# Widespread regulation of gene expression by glucocorticoids in chondrocytes from patients with osteoarthritis as determined by RNA-Seq

**DOI:** 10.1186/s13075-020-02289-7

**Published:** 2020-11-17

**Authors:** Antti Pemmari, Tiina Leppänen, Mari Hämäläinen, Teemu Moilanen, Katriina Vuolteenaho, Eeva Moilanen

**Affiliations:** 1 The Immunopharmacology Research Group, Faculty of Medicine and Health Technology, Tampere University and Tampere University Hospital, Tampere, Finland; 2 Coxa Hospital for Joint Replacement, Tampere, Finland

**Keywords:** Osteoarthritis, Cartilage, Chondrocyte, Glucocorticoid, RNA-Seq

## Abstract

**Background:**

Intra-articular glucocorticoid (GC) injections are widely used as a symptomatic treatment for osteoarthritis (OA). However, there are also concerns about their potentially harmful effects, and their detailed effects on chondrocyte phenotype remain poorly understood.

**Methods:**

We studied the effects of dexamethasone on gene expression in OA chondrocytes with RNA-Seq. Chondrocytes were isolated from the cartilage from OA patients undergoing knee replacement surgery and cultured with or without dexamethasone for 24 h. Total RNA was isolated and sequenced, and functional analysis was performed against the Gene Ontology (GO) database. Results for selected genes were confirmed with RT-PCR. We also investigated genes linked to OA in recent genome-wide expression analysis (GWEA) studies.

**Results:**

Dexamethasone increased the expression of 480 and reduced that of 755 genes with a fold change (FC) 2.0 or greater. Several genes associated with inflammation and cartilage anabolism/catabolism as well as lipid and carbohydrate metabolism were among the most strongly affected genes. In the GO analysis, genes involved in the extracellular matrix organization, cell proliferation and adhesion, inflammation, and collagen synthesis were enriched among the significantly affected genes. In network analysis, NGF, PI3KR1, and VCAM1 were identified as central genes among those most strongly affected by dexamethasone.

**Conclusions:**

This is the first study investigating the genome-wide effects of GCs on the gene expression in OA chondrocytes. In addition to clear anti-inflammatory and anticatabolic effects, GCs affect lipid and glucose metabolism in chondrocytes, an observation that might be particularly important in the metabolic phenotype of OA.

## Introduction

Osteoarthritis (OA) is a disease that affects over 15% of the global population aged 60 or more, causing pain, disability, and reduced quality of life, as well as major costs to healthcare systems [1]. The disease process in the joint is characterized by oxidative stress, low-grade inflammation, and increased catabolism. This eventually results in the breakdown of the articular cartilage and changes in other tissues of the joint, leading to pain and loss of function [2].

Chondrocyte gene expression is markedly altered in osteoarthritis [3]. Some of these changes are thought to be harmful (such as increased expression of proteolytic enzymes and proinflammatory cytokines) and some protective (e.g., increased expression of extracellular matrix [ECM] components) [4]. A couple of genome-wide expression analyses (GWEAs) have previously been performed comparing damaged OA cartilage either to intact OA cartilage or to cartilage from a healthy donor [5–8]. Two larger studies have utilized microarrays and RNA-Seq respectively to compare lesioned and healthy OA cartilage in the same joint, identifying a number of differentially expressed genes involved in inflammation, skeletal system development, cell adhesion, and monosaccharide metabolism [9, 10].

As no proven disease-modifying medications are currently available for OA, all pharmacological treatments are essentially symptomatic [1]. Intra-articular injections with glucocorticoids (GCs) are widely used to combat inflammation and pain and are recommended in the OARSI [11], ACR [12], and NICE [13] treatment guidelines for the management of knee OA. However, their long-term benefits are unclear, and there appears to be a significant variation in the responses between individual patients [14]. GCs are thought to suppress harmful low-grade inflammation present in OA joints. However, there are some concerns about potentially deleterious long-term effects [15, 16].

Glucocorticoids are steroid hormones that are endogenously produced in the adrenal cortex. In target cells, they form a complex with glucocorticoid receptor (GR), which then dimerizes and migrates into the nucleus. After that, GCs cause their anti-inflammatory effects through two main mechanisms. The GR-steroid complex binds to glucocorticoid response elements (GREs) in the promoter region of target genes, promoting the expression of anti-inflammatory genes as well as a number of genes involved in various other functions. In addition, GCs can inhibit the activity of inflammatory transcription factors such as nuclear factor kappa-light-chain-enhancer of activated B cells (NF-κB) and activator protein 1 (AP-1) and sequester their coactivators, leading to suppression of the expression of various inflammatory genes and attenuation of inflammation [17]. In chondrocytes, glucocorticoids have also been shown to reduce chondrocyte viability by inducing oxidative stress and apoptosis [18]. In addition to their effects on inflammation, modulation of glucose and lipid metabolism by glucocorticoids could mediate some of their effects on OA cartilage. Osteoarthritis is linked to metabolic disturbances such as obesity and diabetes, giving rise to the concept of a so-called metabolic phenotype of OA. There is also evidence on metabolic derangements in articular chondrocytes, and impairments in, for example, glycolysis [19], cholesterol metabolism, [20], and mitochondrial respiration [21] have been reported. Due to their established effects on these metabolic pathways in other cell types [22], glucocorticoids could plausibly affect OA pathogenesis via affecting chondrocyte metabolism.

In the present study, we set out to study the effects of the glucocorticoid dexamethasone on gene expression in OA chondrocytes. The aim was to identify significantly modulated pathways and/or functional categories of genes that might be important in the pathogenesis of OA. The results were also compared to those of two previous GWEA studies based on the patient material of the RAAK study [9, 10] to determine whether glucocorticoid treatment might “shift” the expression profile of OA chondrocytes towards the one in healthy cells. In addition, we studied the effects of dexamethasone on potential OA susceptibility genes previously identified in genome-wide association (GWAS) studies [23–25].

## Methods

### Patients and cell culture

Leftover cartilage pieces were collected from OA patients (*n* = 10, BMI 27.3 [5.8] kg/m^2^, age 70.0 [14.8] years, median [IQR]; 4/6 females/males) undergoing total knee replacement surgery in Coxa Hospital for Joint Replacement, Tampere, Finland. All patients fulfilled the American College of Rheumatology classification criteria for knee OA [26] with a mean Kellgren-Lawrence score of 3.5 (SEM 0.22). Patients with diabetes mellitus were excluded from the study.

Chondrocyte isolation and culture were performed as previously described [27]. The articular cartilage was removed aseptically from the subchondral bone using a scalpel and cut into small pieces. The pieces were first washed with phosphate-buffered saline (PBS). After that, they were incubated overnight in the presence of Liberase™ enzyme (Roche Applied Science, Penzberg, Germany, 0.25 mg/mL) diluted in Dulbecco’s modified Eagle’s medium (DMEM, Sigma-Aldrich, St Louis, MO, USA) with GlutaMAX-I containing penicillin (100 units/mL), streptomycin (100 μg/mL), and amphotericin B (250 ng/mL) (all three from Invitrogen, Carlsbad, CA, USA) at 37 °C. The resulting cell suspension was poured through a 70-μm nylon mesh and centrifuged for 5 min at 1500 rpm. The cells were washed and seeded on 24-well plates (0.2 million cells/mL) and incubated for 24 h. Thereafter, the experiments were started by adding dexamethasone (1 μM) in the fresh culture medium (DMEM supplemented with 10% heat-inactivated fetal bovine serum [Lonza] together with the aforementioned substituents) for 24 h.

### Enzyme-linked immunosorbent assay

A separate group of nine cartilage samples was used for ELISA measurements. Cartilage preparation, chondrocyte isolation, and incubation were performed as described above. After 24 h, incubations were terminated by collecting the cell culture media, and culture medium samples were stored at − 20 °C until analyzed. The concentrations of human MMP-1, MMP-13, and CCL2 (MCP-1) were determined by enzyme-linked immunosorbent assay (ELISA) by using reagents from R&D Systems Europe Ltd., 175 Abingdon, UK (catalog nos. DY901, DY511, and DY279, respectively). The detection limits were 39 pg/mL, 31.3 pg/mL, and 7.8 pg/mL, respectively.

### RNA isolation and sample preparation

The culture medium was removed at the indicated time point, and total RNA of the chondrocytes was extracted with GenElute™ Mammalian Total RNA Miniprep kit (Sigma). Total RNA was treated with DNAse I (Qiagen, Hilden, Germany). RNA concentration and integrity were confirmed with the 2100 Bioanalyzer (Agilent Technologies). RNA (150 ng/sample) was reverse-transcribed to cDNA for RT-PCR using TaqMan Reverse Transcription reagents and random hexamers (Applied Biosystems, Foster City, CA, USA).

### Next-generation sequencing and data analysis

Sequencing of RNA samples (500 ng) was performed in the Finnish Institute of Molecular Medicine (FIMM) sequencing core, Helsinki, Finland, using the Illumina HiSeq 2500 sequencing platform. Sequencing depth was 15 million paired-end reads 100 bp in length. Read quality was first assessed using FastQC [28], and the reads were trimmed using Trimmomatic [29]. Trimmed reads were aligned to the full reference human genome with STAR [30]. Count matrices were prepared with the featureCounts program [31]. Differential expression was assessed with DESeq2 using patient number as an additional experimental factor for pairwise comparisons [32]. Gene expression levels are given as DESeq2-normalized counts, and genes with a mean normalized count 5 or less across all samples were excluded from further analysis. For the purposes of further analysis, genes with a minimum of 2.0 fold change (FC) in abundance and false discovery rate (FDR)-corrected *p* value < 0.05 were deemed biologically and statistically significant. Functional analysis was performed against the Gene Ontology (GO) database [33] using ranked list enrichment implemented in the GOrilla tool [34], and protein interactions were studied with STRING [35]. Gene functions were obtained from the NCBI Gene database.

### Quantitative reverse transcription/polymerase chain reaction

cDNA obtained from the reverse transcriptase reaction was diluted 1:20 with RNAse-free water and subjected to quantitative RT-PCR using TaqMan Universal PCR Master Mix and the ABI Prism 7500 Sequence detection system (Applied Biosystems).

Primers and probes for human glyceraldehyde-3-phosphate dehydrogenase (GAPDH), cyclooxygenase-2 (COX-2), MAP kinase phosphatase 1 (MKP-1), matrix metalloproteinases (MMPs) 1 and 13, collagen type II, alpha 1 (COL2A1), and aggrecan (ACAN) were obtained from Metabion International AG (Martinsried, Germany). The primer and probe sequences (Table S1) and concentrations were optimized according to the manufacturer’s instructions in TaqMan Universal PCR Master Mix Protocol part number 4304449 revision C. mRNA levels of other studied genes were determined with TagMan Gene Expression Assays (Applied Biosystems): MMP16 (assay number Hs00234676_m1), collagen type IX, alpha 1 (COL9A1, Hs00932136_g), collagen type XI, alpha 1 (COL11A1, Hs01097664_m1), C-C motif chemokine ligand 2 (CCL2, Hs00234140_m1), MAP kinase phosphatase 2 (MKP-2, Hs01027785_m1), Kruppel like factor 9 (KLF9, Hs00230918_m1), nerve growth factor (NGF, Hs00171458_m1), tumor necrosis factor superfamily member 15 (TNFSF15, Hs00270802_s1), and forkhead box O3 (FOXO3, Hs00818121_m1).

PCR reaction parameters were as follows: incubation at 50 °C for 2 min, incubation at 95 °C for 10 min, and thereafter 40 cycles of denaturation at 95 °C for 15 s and annealing and extension at 60 °C for 1 min. Each experimental reaction was performed in duplicate. The relative mRNA levels of genes listed in Table S1 were quantified using the standard curve method as described in Applied Biosystems User Bulletin number 2. To calculate the relative expression of mRNAs determined with TaqMan assays, the 2^(−ΔΔCT)^ method was used. According to the method, the cycle threshold (C_T_) value for genes of each gene was normalized to the C_T_ value of GAPDH mRNA in the same sample.

### Statistics

For NGS data analysis, normalization was performed and differential expression studied using a negative binomial model implemented in DESeq2. In ELISA and PCR experiments, paired Student’s *t* test was used to assess the statistical significance of differential expression, and multiple testing was addressed using Bonferroni correction. Data is presented as average + standard error of the mean (SEM).

## Results

### Differentially expressed genes

After normalization and correction for multiple testing, 480 genes were upregulated more than 2.0-fold in dexamethasone-treated cells compared to control cells, and 755 downregulated by the same factor (FC < − 2.0). In total, 7371 genes were found to be differentially expressed in dexamethasone-treated versus control cartilage in a statistically significant manner (FDR-corrected *p* value < 0.05). Of these, 3612 were up- and 3759 downregulated. Twelve most strongly up- and downregulated genes are listed in Table 1. A complete list of differentially expressed genes in dexamethasone-treated chondrocytes compared to untreated cells is provided in the Supplementary data in Table S2.

**Table 1:**
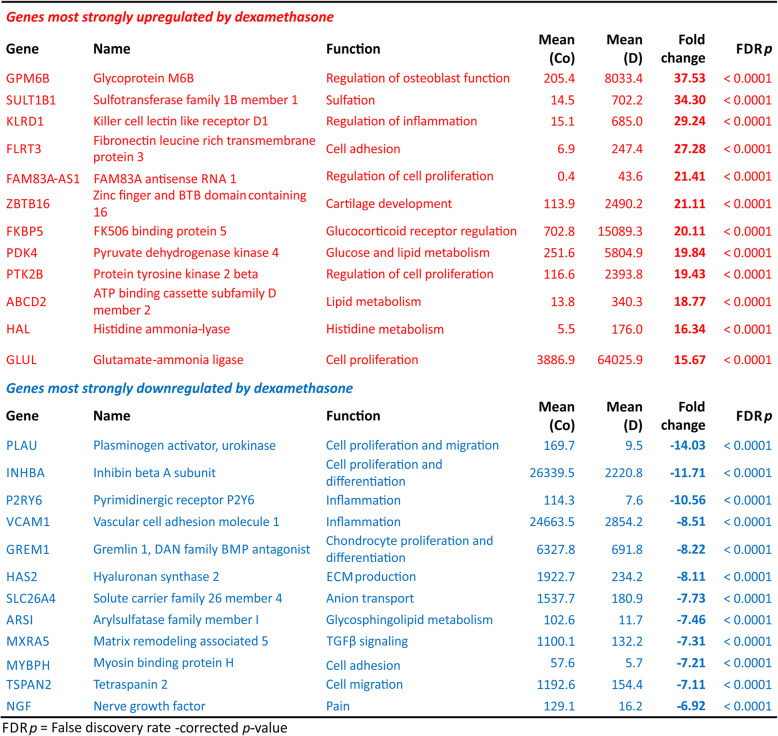
Twelve most strongly up- and downregulated genes in dexamethasone-treated OA chondrocytes (D) relative to control (Co)

The list of the most strongly upregulated genes includes genes involved in the regulation of cell proliferation, inflammation, cartilage development, and carbohydrate and lipid metabolism. Among the most strongly downregulated genes are those linked to cell proliferation and differentiation, extracellular matrix production, and inflammation. Also included was nerve growth factor (NGF), a known mediator of OA pain [36] (Table 1).

Next, we studied, by ranked list enrichment GO analysis, which functional gene categories were enriched among those genes with the largest fold changes into either direction (Table 2). These include, among others, those associated with extracellular matrix organization, regulation of cell proliferation, inflammatory response, cell adhesion, MAP kinase signaling, collagen synthesis, and lipid and carbohydrate metabolism.

**Table 2:** Ranked Gene Ontology (GO) terms significantly affected by dexamethasone

GO term	Description	FDR *p-*value
GO:0048523	Negative regulation of cellular process	3.50E-08
GO:0032502	Developmental process	3.81E-08
GO:0048519	Negative regulation of biological process	4.05E-08
GO:0030334	Regulation of cell migration	5.17E-08
GO:0051239	Regulation of multicellular organismal process	1.69E-07
GO:0032501	Multicellular organismal process	4.37E-07
GO:0007166	Cell surface receptor signaling pathway	4.66E-07
GO:0043062	Extracellular structure organization	6.75E-06
GO:0030198	Extracellular matrix organization	7.34E-06
GO:0048856	Anatomical structure development	7.86E-06
GO:0032879	Regulation of localization	7.91E-06
GO:0009725	Response to hormone	1.25E-05
GO:0042127	Regulation of cell proliferation	1.27E-05
GO:0050896	Response to stimulus	3.12E-05
GO:0040011	Locomotion	0.000137
GO:0048518	Positive regulation of biological process	0.000193
GO:0050794	Regulation of cellular process	0.000197
GO:0040008	Regulation of growth	0.000199
GO:0048583	Regulation of response to stimulus	0.000351
GO:0065008	Regulation of biological quality	0.000584
GO:0043408	Regulation of MAPK cascade	0.00105
GO:0009719	Response to endogenous stimulus	0.00152
GO:0006954	Inflammatory response	0.00235
GO:0006950	Response to stress	0.00241
GO:0065007	Biological regulation	0.00281
GO:0051174	Regulation of phosphorus metabolic process	0.00303
GO:0010273	Detoxification of copper ion	0.00306
GO:0048878	Chemical homeostasis	0.0032
GO:0010941	Regulation of cell death	0.00426
GO:0070482	Response to oxygen levels	0.00506
GO:0061687	Detoxification of inorganic compound	0.0057
GO:0006928	Movement of cell or subcellular component	0.00578
GO:0022610	Biological adhesion	0.00626
GO:0009611	Response to wounding	0.0064
GO:0007155	Cell adhesion	0.00717
GO:0065009	Regulation of molecular function	0.0116
GO:0051338	Regulation of transferase activity	0.023
GO:0006068	Ethanol catabolic process	0.0239
GO:0019216	Regulation of lipid metabolic process	0.0255
GO:0009628	Response to abiotic stimulus	0.0342
GO:0006109	Regulation of carbohydrate metabolic process	0.0375
GO:0051246	Regulation of protein metabolic process	0.039
GO:0002682	Regulation of immune system process	0.0425
GO:0032964	Collagen biosynthetic process	0.0429
GO:0008283	Cell proliferation	0.0454

FDR *p*-value = False discovery rate -corrected *p*-value

### Genes involved in inflammation, oxidative stress, and extracellular matrix production

As low-grade inflammation, oxidative stress, and changes in extracellular matrix production and catabolism are central features in the pathogenesis of OA, we set out to separately study genes linked to these processes (Table 3). Several proinflammatory factors such as cyclooxygenase-2 (COX-2, fold change − 4.29), chemokine (C-C motif) ligand 2 (CCL2, fold change − 5.43), and TNF superfamily member 15 (TNFSF15, fold change − 5.98) were significantly downregulated by dexamethasone, while the anti-inflammatory MAP kinase phosphatases 1 (MKP-1, fold change 10.48) and 2 (MKP-2, fold change 5.50) were upregulated. Genes affecting response to oxidative stress, such as Kruppel-like factor 9 (KLF9, fold change 10.85) and forkhead box O3 (FOXO3, fold change 4.29), were similarly upregulated. Also, the catabolic matrix metalloproteinases 1, 16, and 13 (MMP1 with fold change − 2.85, MMP16 with fold change − 3.12 and MMP13 with fold change − 4.08) were downregulated by dexamethasone. Various collagens were downregulated by dexamethasone, including the most highly expressed collagens COL2A1 (fold change − 2.28) and COL11A1 (fold change − 3.12). However, aggrecan was found to be significantly upregulated (fold change 2.43). The expression of connective tissue growth factor (CTGF, fold change 2.25) was enhanced while fibroblast growth factor 1 (FGF1, fold change − 2.62), transforming growth factor beta 2 (TGFB2, fold change − 2.57), and vascular endothelial growth factor A (VEGFA, fold change − 2.36) were downregulated by dexamethasone. The change in the expression of selected genes was confirmed with RT-PCR (Table S3) and the production of MMPs 1 and 13 as well as CCL2 with ELISA (Figure S1).

**Table 3:**
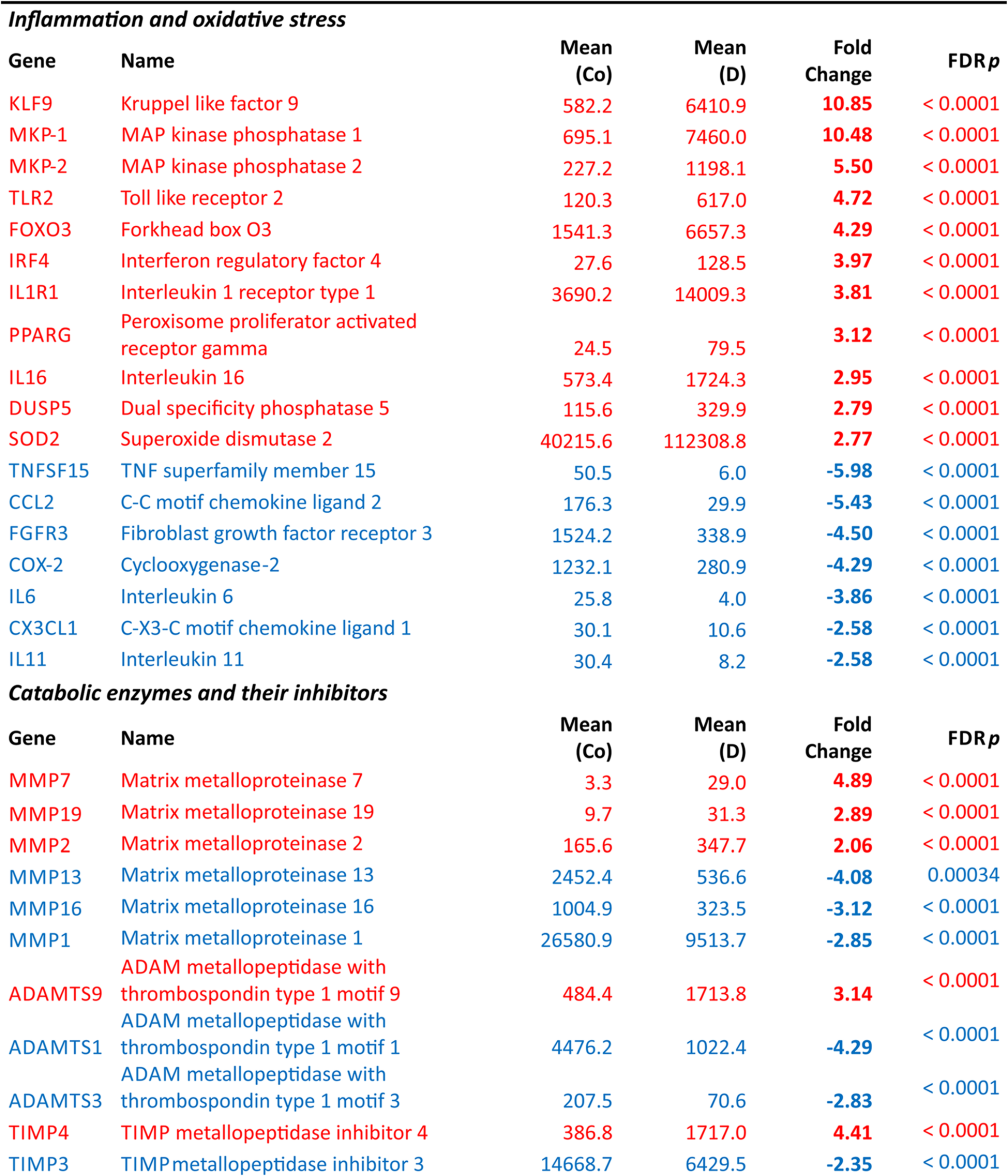

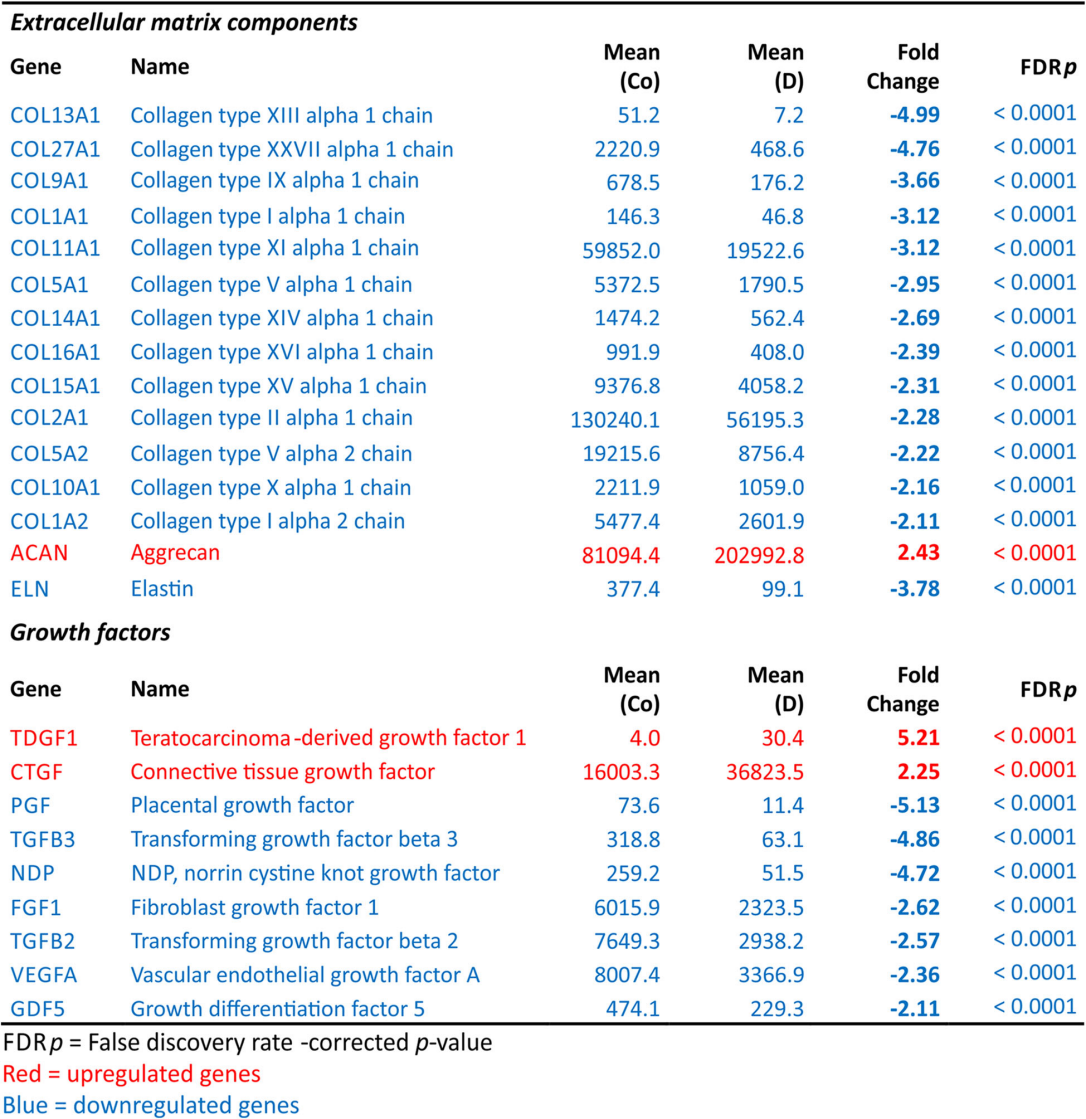
Selected genes linked to inflammation, oxidative stress, catabolic enzymes, and extracellular matrix production as well as growth factors in dexamethasone-treated OA chondrocytes (D) relative to control (Co)

### Carbohydrate and lipid metabolism

As glucocorticoids regulate glucose and lipid metabolism, and OA is known to be associated with metabolic syndrome, we separately studied genes for proteins participating in the main pathways of carbohydrate and lipid metabolism (glycolysis, oxidative phosphorylation, lipolysis, and beta-oxidation) [37]. Dexamethasone did not have a marked (fold change > 2.0) effect on any of these genes, with the sole exception being upregulation of long-chain acyl-CoA dehydrogenase (ACADL) (fold change 2.60) (Table S4).

**Fig. 1:**
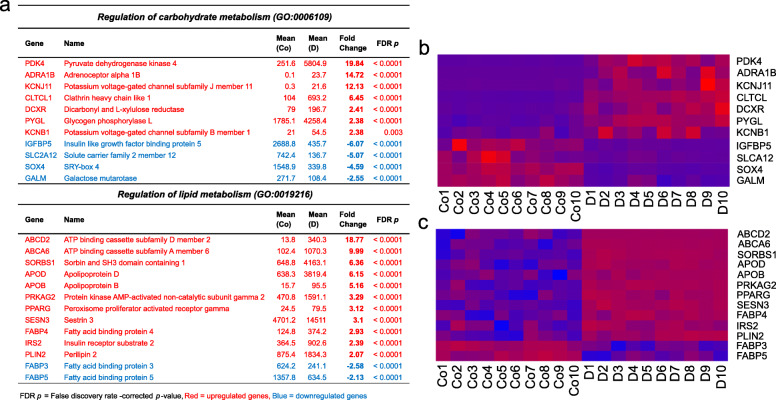
Genes significantly affected by dexamethasone and regulating carbohydrate (GO:0006109) and lipid (GO:0019216) metabolism. **a** Table showing the expression of genes regulating carbohydrate and lipid metabolism in dexamethasone-treated OA chondrocytes (D) compared with control (Co). **b** and **c** Heatmaps showing the expression of differentially expressed genes involved in carbohydrate and lipid metabolism, respectively, in dexamethasone-treated OA cells (D) and controls (Co). Expression values are DESeq2-normalized and row-scaled, with blue signifying lower and red higher expression

However, dexamethasone affected the expression of several other genes regulating lipid and carbohydrate metabolism (those associated with hierarchically high-level GO terms GO:0019216 Regulation of lipid metabolism and GO:0006109 Regulation of carbohydrate metabolism). For example, pyruvate dehydrogenase kinase 4 (PDK4) and glycogen phosphorylase L (PYGL), as well as the redox regulator Sestrin 3 (SESN3), were markedly upregulated by dexamethasone (Fig. 1).

### Integration with previous GWAS and GWEA studies

When the 53 genes previously associated with OA in GWAS studies [23–25] were studied separately, 12 of them were found to be significantly affected by dexamethasone with a FC greater than 2.0 (Table S5). Eleven of them (including COL11A1, COX-2, GDF5, IL6, and VEGFA) were downregulated and only one, IL16, upregulated.

The microarray-based GWEA study by Ramos et al. [9] identified 18 genes that were differentially expressed between degraded and preserved OA cartilage in the same joint, and two of them were affected by dexamethasone in our data (Table S6). Of these, the expression of COL9A1 was found to be lower in degraded cartilage, and the gene was also downregulated by dexamethasone. Nerve growth factor (NGF), in turn, was expressed at higher levels in degraded cartilage and strongly downregulated by dexamethasone.

Almeida et al. [10] analyzed gene expression in degraded and spared OA cartilage with NGS. In their data, 372 genes were differentially expressed with FC > 2.0 into either direction, and 78 of them were significantly affected by dexamethasone (FC > 2.0 into either direction) in the present study. Of these, 19 were upregulated in degraded cartilage compared with spared cartilage, and upregulated by dexamethasone in our data, while 25 were upregulated in degraded cartilage and downregulated by dexamethasone. Seventeen were downregulated in degraded cartilage and by dexamethasone, while another 17 were downregulated in degraded cartilage and upregulated by dexamethasone. Interestingly, NGF was one of the genes whose expression was enhanced in degraded OA cartilage and normalized by dexamethasone (Table S7).

### Interactions between the differentially expressed genes

**Fig. 2:**
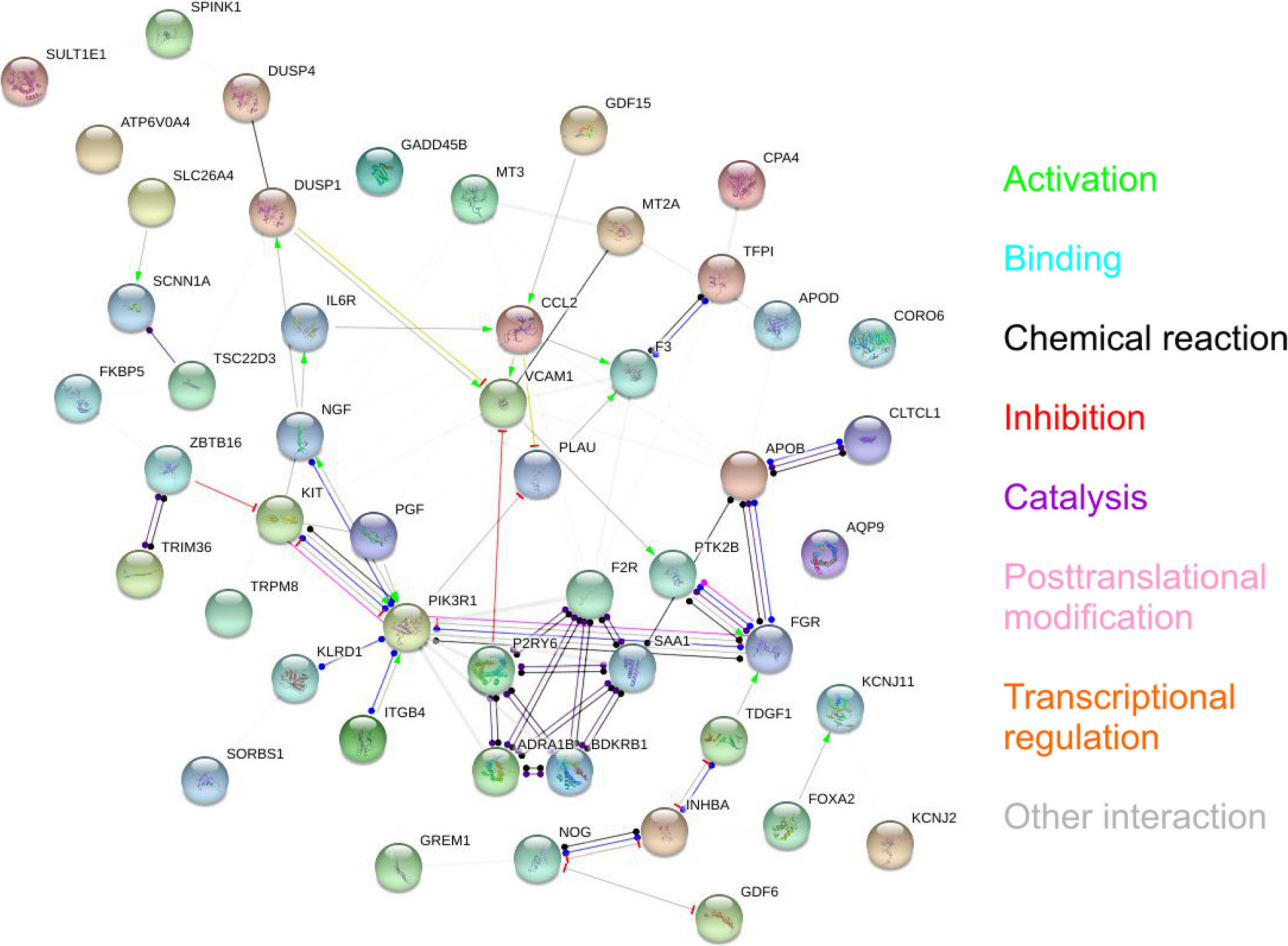
Interactions among the genes that were up- or downregulated by dexamethasone with an FC 5.0 or greater into either direction, as determined with STRING. Genes with no interactions are excluded from the graph. Colors of the edges are as follows: green = activation, blue = binding, black = chemical reaction, red = inhibition, violet = catalysis, pink = posttranslational modification, yellow = transcriptional regulation, and gray = other interaction

Among the genes most strongly affected by dexamethasone (FC > 5.0), several interactions were identified using the STRING database (Fig. 2). Phosphatidylinositol 3-kinase regulatory subunit alpha (PI3KR1), vascular cell adhesion protein 1 (VCAM1), KIT proto-oncogene receptor tyrosine kinase (KIT), FGR proto-oncogene Src family tyrosine kinase (FGR), C-C motif ligand 2 (CCL2), and nerve growth factor (NGF) were found to occupy central positions in the interaction network.

## Discussion

The pathology of OA is characterized by eventual cartilage degradation that is caused by imbalanced gene expression profiles in the cartilage. The effects of GCs used in the treatment of OA on this balance are of interest, and RNA-Seq provides a comprehensive view of gene expression in a tissue. Dexamethasone was found to affect the expression of a large number of genes in OA chondrocytes. Among the most strongly affected genes were several involved in inflammation, extracellular matrix organization, and carbohydrate and lipid metabolism.

Constant low-grade joint inflammation along with inflammatory exacerbations is a central feature of OA. Glucocorticoids were found to inhibit inflammation, and this might explain part of their therapeutic effects on OA exacerbations. In our data, dexamethasone reduced the expression of well-known inflammatory factors such as cyclooxygenase-2 (COX-2) [38], interleukin 6 (IL6), and C-C motif chemokine ligand 2 (CCL2) [39]. In addition, dexamethasone downregulated the expression of cartilage extracellular matrix-degrading matrix metalloproteinases (MMPs) 1, 13, and 16, while various collagens and anabolic factors (including hyaluronan synthase 2 [HAS2], one of the most strongly downregulated genes) were also downregulated. As the relative expression of catabolic and anabolic factors in the cartilage varies during the course of OA, the effects of glucocorticoids on cartilage homeostasis are likely to depend on the phase of the disease process.

Osteoarthritis as well as glucocorticoid treatment has been reported to be associated with increased oxidative stress and chondrocyte apoptosis [18]. Activation of the ROS/Akt/FOXO3 signaling pathway appears to counteract these effects, and particularly, forkhead box O3 (FOXO3) has been shown to protect chondrocytes from apoptosis [40]. Interestingly, the expression of FOXO3 was strongly upregulated by dexamethasone in the present data. Superoxide dismutase 2 (SOD2) inactivates the superoxide anion, reducing oxidative stress [21]. SOD2 was highly expressed in OA cartilage, and its expression was nearly tripled by treatment with dexamethasone. On the other hand, dexamethasone strongly enhanced the expression of KLF9, which has been shown to sensitize cells to oxidative stress [41] and may contribute to the previously reported dexamethasone-induced oxidative stress [18].

Pain in OA is mediated through various intracellular pathways and soluble factors, of which nerve growth factor (NGF) is thought to be of particular importance [42]. Antibodies targeting NGF have been shown to be effective for treating OA pain but may also accelerate joint destruction in a small group of patients [36]. In the present data, the expression of NGF was reduced by dexamethasone indicating that intra-articular GC injections might alleviate OA pain at least partly by reducing the synthesis of NGF. The mechanisms of NGF blocker-induced joint degradation are still largely unknown. Increased use of the OA-affected joint, enabled by analgesia, has been hypothesized to explain the findings. However, accelerated degradation has been observed also in non-OA-affected (and initially painless) joints, casting doubt on this hypothesis [36]. Whether GC-induced downregulation of NGF might have similar deleterious effects remains to be studied. In addition to NGF, dexamethasone treatment also downregulated the expression of prostaglandin-producing COX-2 and vascular endothelial growth factor A (VEGFA). Prostaglandins, particularly PGE_2_, as well as VEGFA are involved in mediating OA pain [43]. Thus, their downregulation by dexamethasone is likely to contribute to the pain-alleviating properties of glucocorticoids in OA.

Dexamethasone markedly affected the expression of several genes involved in glucose and lipid metabolism. An interesting example is pyruvate dehydrogenase kinase 4 (PDK4), which was one of the genes most strongly upregulated by dexamethasone. As this gene inactivates pyruvate dehydrogenase and prevents pyruvate produced in glycolysis from progressing to oxidative phosphorylation [44], this glucocorticoid effect may shift carbohydrate metabolism from mitochondrial respiration towards glycolysis. Several genes promoting lipid synthesis and transport, such as perilipin 2 (PLIN2) [45] and 5′-AMP-activated protein kinase subunit gamma-2 (PRKAG2) [46], were also upregulated. However, genes coding for the enzymes participating in the major pathways of carbohydrate and lipid metabolism itself (glycolysis, oxidative phosphorylation, lipolysis, and beta-oxidation) were not significantly affected. Of the OA-associated lipid metabolism genes, apolipoprotein D (APOD) is a central mediator of steroid and lipoprotein metabolism. Its expression has been shown to be decreased in OA chondrocytes [47], and APOD was found to be upregulated by dexamethasone in the present study. Sestrin 3 (SESN3), which was highly expressed in OA cartilage and upregulated by dexamethasone, is involved in the regulation of carbohydrate and lipid metabolism. Impairment of Sestrin signaling has been implicated in the pathogenesis of OA [48]. These are examples of dexamethasone-induced normalization of the expression of lipid metabolism-related genes in OA chondrocytes. Furthermore, peroxisome proliferator-activated receptor gamma (PPARG), which was likewise upregulated by dexamethasone, is widely regarded as an anti-inflammatory and chondroprotective factor [49] in addition to its significant role in cellular metabolism.

Osteoarthritis has a large heritable component, as up to 50% of the incidence of the disease is thought to be explained by genetics [50, 51]. Several genome-wide association (GWAS) studies have been performed in OA [23–25], identifying at least 53 genes associated with the disease. Many of the identified genes affect extracellular matrix synthesis and skeletal system development, while the effects of some of the genes are largely unknown [23–25]. GWAS studies investigate systemic genomic variation, while expression analyses (such as the present study) directly measure gene expression in the tissue of interest. Nevertheless, as gene polymorphisms often affect the function or activity of the protein coded by the gene, it can be postulated that altered expression or activity of the OA-linked genes might affect the development of the disease and serve as a treatment target. Twelve of the 53 genes previously associated with hip and/or knee OA in GWAS studies were found to be clearly (FC > 2.0) affected by dexamethasone. Examples of those are COL11A1, COX-2, GDF5, IL6, and VEGFA, all of which were downregulated. Our study identified hundreds of differential genes by dexamethasone, which makes it quite probable that some of them should, by chance, be among those previously linked to OA. However, in the case of those genes which have relatively well-established mechanistic links to OA development, we think that highlighting them as genes potentially mediating the effects of glucocorticoids on the development or symptoms of the disease is justified. For example, GDF5 may affect cartilage remodeling and repair [52], COX-2 and IL6 are indicated to promote inflammation in OA [39], and VEGF-induced angiogenesis seems to play a role in OA progression and pain [42]. Further elucidating the relative glucocorticoid-regulated effects of these genes on OA pathophysiology could be an interesting avenue of further study.

The microarray-based genome-wide expression analysis (GWEA) study by Ramos et al. [9] previously compared the gene expression in OA affected and preserved cartilage in the same joint. It identified 18 differentially expressed genes, whose up- or downregulation might therefore be expected to affect the pathogenesis of OA. Two of those, namely NGF and collagen 9 alpha 1 (COL9A1), were markedly affected by dexamethasone in the present study. NGF was upregulated in more severely affected OA cartilage [9], and in the present study, dexamethasone was found to downregulate it. As previously discussed, NGF downregulation might alleviate OA pain but also predispose the cartilage to accelerated destruction [36]. COL9A1 was downregulated in severely affected OA cartilage [9], and dexamethasone further downregulated its expression in our data. This can be regarded as an antianabolic effect with an impact on OA, which is further supported by the finding that COL9A1 deficiency induces osteoarthritis-like pathology in mice [53]. The results of our study were also compared with a recent larger, NGS-based expression analysis based on an extended study population [10]. Of the 372 genes identified in that study with markedly differential expression (FC > 2.0 in either direction) between degraded and preserved OA cartilage, 78 were significantly affected by dexamethasone in our study. While the expression of 42 genes was “normalized” by dexamethasone (i.e., their expression was altered in the direction of preserved cartilage), nearly the same number (34) were altered in the opposite direction. Thus, while glucocorticoids may partially normalize the phenotype of OA chondrocytes, this may be counteracted by increased expression of genes driving the disease process in OA cartilage.

The time course of the effects of glucocorticoids is an important factor to be taken into account when evaluating their potential effects on cartilage. When used at clinically relevant doses (1–3 mg), dexamethasone injected intra-articularly seems to be mostly absorbed from the joint within 24 h [54]. We thus propose that the time point used in the current study (24 h) can be expected to reasonably well capture the effects of glucocorticoids on gene expression in OA chondrocytes, while the effects on protein production are known to occur and last over a longer time frame (several days) [55]. Investigating the time evolution of chondrocyte gene expression in response to glucocorticoid treatment (including direct and secondary effects) would be an interesting avenue of further study.

## Conclusions

In conclusion, dexamethasone was found to cause a major phenotypic switch in OA chondrocytes, while the overall effect on genes linked to OA in GWAS and GWEA studies appeared to be modest. In addition to clear anti-inflammatory, anticatabolic, and extracellular matrix-targeting effects, dexamethasone was found to affect lipid and glucose metabolism-related genes, an observation that might be particularly important in the metabolic phenotype of OA.

## Supplementary information

**Additional file 1: Table S1.** Primers and probes used for quantitative RT-PCR. **Table S3.** Expression of cartilage constituents in dexamethasone-treated OA chondrocytes (D) relative to controls (Co). **Table S4.** Selected genes linked to inflammation, oxidative stress, catabolism and extracellular matrix production in dexamethasone-treated OA chondrocytes (D) relative to controls (Co) as determined by NGS and confirmed with RT-PCR. **Table S5.** Expression of genes belonging to the major pathways of carbohydrate and lipid metabolism in dexamethasone-treated OA chondrocytes (D) relative to controls (Co) [35]. **Table S6.** Effects of dexamethasone on genes linked to OA in previous GWAS studies [21–23]. **Table S7.** Effects of dexamethasone on genes previously linked to OA in the GWEA study by Ramos et al. [9]. **Table S8.** Effects of dexamethasone on genes previously linked to OA cartilage in the GWEA study by Almeida et al. [10]. **Figure S1.** Effects of dexamethasone on the production of catabolic and proinflammatory factors in OA chondrocytes. OA chondrocytes / chondrocytes isolated from OA patients were cultured for 24 h with or without dexamethasone (1 μM). MMP-1 (A), MMP-13 (B) and CCL2 (C) levels in the culture media were determined with ELISA. MMP-1 (D), MMP-13 (E) and CCL2 (F) mRNA expression was studied with quantitative RT-PCR and normalized against GAPDH. The results were compared against control, which was set as 100 %. The results are expressed as mean + SEM, *n* = 9. *: *p* < 0.05, **: *p* < 0.01 and ***: *p* < 0.001, compared to the untreated control.

**Additional file 2: Table S2.** All genes differentially expressed in dexamethasone-treated OA chondrocytes (D) relative to controls (Co).

## Data Availability

The list of all genes significantly affected by dexamethasone is included in Supplementary data (Table S2).

## Abbreviations

ECM: Extracellular matrix
ELISA: Enzyme-linked immunosorbent assay
FC: Fold change
FDR: False discovery rate
GC: Glucocorticoid
GO: Gene Ontology
GWEA: Genome-wide expression analysis
GWAS: Genome-wide association study
MMP: Matrix metalloproteinase
mRNA: Messenger RNA
NGS: Next-generation sequencing
OA: Osteoarthritis
RNA-Seq: RNA sequencing
RT-PCR: Real-time quantitative polymerase chain reaction
SEM: Standard error of the mean
