# Supplementary material for: Widespread regulation of gene expression by glucocorticoids in chondrocytes from patients with osteoarthritis as determined by RNA-Seq
Source: Arthritis Res Ther. 2020 Nov 17;22:271. doi: 10.1186/s13075-020-02289-7 (PMC7670667; doi:10.1186/s13075-020-02289-7)
Supplement: Supplementary file 1 — Additional file 1: Table S1. Primers and probes used for quantitative RT-PCR. Table S3. Expression of cartilage constituents in dexamethasone-treated OA chondrocytes (D) relative to controls (Co). Table S4. Selected genes linked to inflammation, oxidative stress, catabolism and extracellular matrix production in dexamethasone-treated OA chondrocytes (D) relative to controls (Co) as determined by NGS and confirmed with RT-PCR. Table S5. Expression of genes belonging to the major pathways of carbohydrate and lipid metabolism in dexamethasone-treated OA chondrocytes (D) relative to controls (Co) [35]. Table S6. Effects of dexamethasone on genes linked to OA in previous GWAS studies [21–23]. Table S7. Effects of dexamethasone on genes previously linked to OA in the GWEA study by Ramos et al. [9]. Table S8. Effects of dexamethasone on genes previously linked to OA cartilage in the GWEA study by Almeida et al. [10]. Figure S1. Effects of dexamethasone on the production of catabolic and proinflammatory factors in OA chondrocytes. OA chondrocytes / chondrocytes isolated from OA patients were cultured for 24 h with or without dexamethasone (1 μM). MMP-1 (A), MMP-13 (B) and CCL2 (C) levels in the culture media were determined with ELISA. MMP-1 (D), MMP-13 (E) and CCL2 (F) mRNA expression was studied with quantitative RT-PCR and normalized against GAPDH. The results were compared against control, which was set as 100 %. The results are expressed as mean + SEM, n = 9. *: p < 0.05, **: p < 0.01 and ***: p < 0.001, compared to the untreated control. [file 13075_2020_2289_MOESM1_ESM.docx]

**Supplementary data**

**Table S1: Primers and probes used for quantitative RT-PCR**

| **Primer/probe** | **Sequence** |
| --- | --- |
| hGAPDH forward | 5′-AAGGTCGGAGTCAACGGATTT-3′ |
| hGAPDH reverse | 5′-GCAACAATATCCACTTTACCAGAGTTAA-3′ |
| hGAPDH probe | 5′-CGCCTGGTCACCAGGGCTGC-3′ |
| hCOX-2 forward | 5′-CAACTCTATATTGCTGGAACATGGA-3′ |
| hCOX-2 reverse | 5′-TGGAAGCCTGTGATACTTTCTGTACT-3′ |
| hCOX-2 probe | 5′-TCCTACCACCAGCAACCCTGCCA-3′ |
| hMKP-1 forward | 5′-ACGAGGCCATTGACTTCATAGAC-3′ |
| hMKP-1 reverse | 5′-TCGATTAGTCCTCATAAGGTAAGCAA-3′ |
| hMKP-1 probe | 5′-CCACTGCCAGGCAGGCATTTCC-3′ |
| hMMP1 forward | 5′-TTGAAGCTGCTTACGAATTTGC-3′ |
| hMMP1 reverse | 5′-GAAGCCAAAGGAGCTGTAGATGTC-3′ |
| hMMP1 probe | 5′-CAGAGATGAAGTCCGGTTTTTCAAAGGGA-3′ |
| hMMP13 forward | 5′-TGATCTCTTTTGGAATTAAGGAGCAT-3′ |
| hMMP13 reverse | 5′-GGAACTACTTGTCCAGGTTTCATCAT-3′ |
| hMMP13 probe | 5′-CCCTCTGGCCTGCTGGCTCATG-3′ |
| hCOL2A1 forward | 5′-GGCAATAGCAGGTTCACGTACA-3′ |
| hCOL2A1 reverse | 5′-CGATAACAGTCTTGCCCCACTT-3′ |
| hCOL2A1 probe | 5′-CTGAAGGATGGCTGCACGAAACATACC-3′ |
| hACAN forward | 5′-GCCTGCGCTCCAATGACT-3′ |
| hACAN reverse | 5′-TAATGGAACACGATGCCTTTCA-3′ |
| hACAN probe | 5′-CCATGCATCACCTCGCAGCGGTA′ |

**Table S2: All genes differentially expressed in dexamethasone-treated OA chondrocytes (D) relative to controls (Co)**

**(Submitted as a separate file)**

**Table S3: Expression of cartilage constituents in dexamethasone-treated OA chondrocytes (D) relative to controls (Co)**

| **Gene** | **Name** | **Mean (Co)** | **Mean (D)** | **Fold Change** | **FDR *p*** |
| --- | --- | --- | --- | --- | --- |
| COL13A1 | Collagen type XIII alpha 1 chain | 51.2 | 7.2 | **-4.99** | < 0.0001 |
| COL27A1 | Collagen type XXVII alpha 1 chain | 2220.9 | 468.6 | **-4.76** | < 0.0001 |
| COL9A1 | Collagen type IX alpha 1 chain | 678.5 | 176.2 | **-3.66** | < 0.0001 |
| COL1A1 | Collagen type I alpha 1 chain | 146.3 | 46.8 | **-3.12** | < 0.0001 |
| COL11A1 | Collagen type XI alpha 1 chain | 59852 | 19522.6 | **-3.12** | < 0.0001 |
| COL5A1 | Collagen type V alpha 1 chain | 5372.5 | 1790.5 | **-2.95** | < 0.0001 |
| COL14A1 | Collagen type XIV alpha 1 chain | 1474.2 | 562.4 | **-2.69** | < 0.0001 |
| COL16A1 | Collagen type XVI alpha 1 chain | 991.9 | 408 | **-2.39** | < 0.0001 |
| COL15A1 | Collagen type XV alpha 1 chain | 9376.8 | 4058.2 | **-2.31** | < 0.0001 |
| COL2A1 | Collagen type II alpha 1 chain | 130240.1 | 56195.3 | **-2.28** | < 0.0001 |
| COL5A2 | Collagen type V alpha 2 chain | 19215.6 | 8756.4 | **-2.22** | < 0.0001 |
| COL10A1 | Collagen type X alpha 1 chain | 2211.9 | 1059 | **-2.16** | < 0.0001 |
| COL1A2 | Collagen type I alpha 2 chain | 5477.4 | 2601.9 | **-2.11** | < 0.0001 |
| ACAN | Aggrecan | 81094.4 | 202992.8 | **2.43** | < 0.0001 |
| ELN | Elastin | 377.4 | 99.1 | **-3.78** | < 0.0001 |

FDR *p* = False discovery rate -corrected *p*-value

**Table S4: Selected genes linked to inflammation, oxidative stress, catabolism and extracellular matrix production in dexamethasone-treated OA chondrocytes (D) relative to controls (Co) as determined by NGS and confirmed with RT-PCR**

| **Gene** | **Name** | **Mean (Co)** | **Mean (D)** | **FC (NGS)** | **FDR *p* (NGS)** | **FC** | **FDR *p*** |
| --- | --- | --- | --- | --- | --- | --- | --- |
|  |  |  |  |  |  | **(RT-PCR)** | **(RT-PCR)** |
| ***Inflammation and oxidative stress*** | | | | | | | |
| KLF9 | Kruppel like factor 9 | 582.2 | 6410.9 | **10.85** | < 0.0001 | **19.26** | 0.0046 |
| MKP-1 | MAP kinase phosphatase 1 | 695.1 | 7460 | **10.48** | < 0.0001 | **6.26** | < 0.0001 |
| MKP-2 | MAP kinase phosphatase 2 | 227.2 | 1198.1 | **5.5** | < 0.0001 | **6.19** | 0.0012 |
| FOXO3 | Forkhead box O3 | 1541.3 | 6657.3 | **4.29** | < 0.0001 | **10.01** | 0.011 |
| NGF | Nerve growth factor | 129.1 | 16.2 | **-6.92** | < 0.0001 | **-4.46** | 0.0005 |
| TNFSF15 | TNF superfamily member 15 | 50.5 | 6 | **-5.98** | < 0.0001 | **-8.02** | 0.041 |
| CCL2 | C-C motif chemokine ligand 2 | 176.3 | 29.9 | **-5.43** | < 0.0001 | **-2.87** | 0.012 |
| COX-2 | Cyclooxygenase-2 | 1232.1 | 280.9 | **-4.29** | < 0.0001 | **-4.45** | 0.0016 |
| GDF5 | Growth differentiation factor 5 | 474.1 | 229.3 | **-2.11** | < 0.0001 | **-1.44** | 0.034 |
| ***Matrix metalloproteinases*** | | | | | | | |
| MMP13 | Matrix metalloproteinase 13 | 2452.4 | 536.6 | **-4.08** | 0.00034 | **-2.82** | 0.004 |
| MMP16 | Matrix metalloproteinase 16 | 1004.9 | 323.5 | **-3.12** | < 0.0001 | **-3.99** | <0.0001 |
| MMP1 | Matrix metalloproteinase 1 | 26580.9 | 9513.7 | **-2.85** | < 0.0001 | **-2.51** | 0.0042 |
| ***Extracellular matrix components*** | | | | | | | |
| COL9A1 | Collagen type IX alpha 1 chain | 678.5 | 176.2 | **-3.66** | < 0.0001 | **-3.28** | 0.0012 |
| COL11A1 | Collagen type XI alpha 1 chain | 59852 | 19522.6 | **-3.12** | < 0.0001 | **-3.07** | <0.0001 |
| COL2A1 | Collagen type II alpha 1 chain | 130240.1 | 56195.3 | **-2.28** | < 0.0001 | **-1.95** | 0.0002 |
| ACAN | Aggrecan | 81094.4 | 202992.8 | **2.43** | < 0.0001 | **2.34** | 0.0003 |

NGS = Next generation sequencing

FC = Fold change

FDR *p* = False discovery rate -corrected *p*-value

Red = upregulated genes

Blue = downregulated genes

**Table S5: Expression of genes belonging to the major pathways of carbohydrate and lipid metabolism in dexamethasone-treated OA chondrocytes (D) relative to controls (Co) [35]**

| ***Glycolysis*** | | | | | |
| --- | --- | --- | --- | --- | --- |
| **Gene** | **Name** | **Mean (Co)** | **Mean (D)** | **Fold change** | **FDR *p*** |
| HK1 | hexokinase 1 | 8979.6 | 10269.1 | **1.14** | < 0.0001 |
| HK2 | hexokinase 2 | 1150.5 | 812.5 | **-1.42** | < 0.0001 |
| HK3 | hexokinase 3 | 0.8 | 2.4 | **1.57** | 0.52 |
| GPI | glucose-6-phosphate isomerase | 2397.6 | 1791.3 | **-1.34** | < 0.0001 |
| PFKL | phosphofructokinase, liver type | 1329.8 | 971.9 | **-1.37** | < 0.0001 |
| PFKM | phosphofructokinase, muscle | 1075.0 | 811.7 | **-1.33** | < 0.0001 |
| PFKP | phosphofructokinase, platelet | 2894.5 | 3198.5 | **1.09** | 0.22 |
| ALDOA | aldolase, fructose-bisphosphate A | 4484.0 | 3231.3 | **-1.39** | < 0.0001 |
| ALDOC | aldolase, fructose-bisphosphate C | 896.7 | 607.3 | **-1.47** | < 0.0001 |
| TPI1 | triosephosphate isomerase 1 | 9017.1 | 5450.2 | **-1.66** | < 0.0001 |
| GAPDH | glyceraldehyde-3-phosphate dehydrogenase | 29461.7 | 22816.9 | **-1.29** | < 0.0001 |
| PGK1 | phosphoglycerate kinase 1 | 12358.2 | 7736.0 | **-1.59** | < 0.0001 |
| PGK2 | phosphoglycerate kinase 2 | 0.5 | 0.2 | **-1.07** | 0.93 |
| PGAM1 | phosphoglycerate mutase 1 | 1904.1 | 1222.6 | **-1.56** | < 0.0001 |
| PGAM2 | phosphoglycerate mutase 2 | 14.3 | 9.4 | **-1.41** | 0.47 |
| PGAM4 | phosphoglycerate mutase family member 4 | 0.5 | 1.4 | **1.30** | 0.84 |
| PGAM5 | PGAM family member 5, mitochondrial serine/threonine protein phosphatase | 398.9 | 413.3 | **1.04** | 0.87 |
| ENO1 | enolase 1 | 49167.8 | 49473.1 | **1.01** | 0.98 |
| ENO3 | enolase 3 | 325.3 | 287.0 | **-1.13** | 0.11 |
| PKM | pyruvate kinase, muscle | 47215.8 | 32348.1 | **-1.46** | < 0.0001 |
| PKLR | pyruvate kinase, liver and RBC | 0.5 | 1.5 | **1.24** | 0.84 |
| ***Oxidative phosphorylation*** | | | | | |
| **Gene** | **Name** | **Mean (Co)** | **Mean (D)** | **Fold change** | **FDR *p*** |
| CS | citrate synthase | 785.5 | 821.1 | **1.04** | 0.78 |
| ACO1 | aconitase 1 (soluble) | 1428.1 | 1662.7 | **1.16** | 0.001474 |
| ACO2 | aconitase 2 (mitochondrial) | 1281.6 | 1331.1 | **1.04** | 0.84 |
| IDH1 | isocitrate dehydrogenase (NADP(+)) 1, cytosolic | 4134.3 | 3294.5 | **-1.26** | < 0.0001 |
| IDH2 | isocitrate dehydrogenase (NADP(+)) 2 mitochondrial | 583.1 | 466.4 | **-1.25** | 0.0042 |
| IDH3A | isocitrate dehydrogenase 3 (NAD(+)) alpha | 1128.6 | 1120.6 | **-1.01** | 0.97 |
| IDH3B | isocitrate dehydrogenase 3 (NAD(+)) beta | 727.4 | 682.4 | **-1.06** | 0.52 |
| IDH3G | isocitrate dehydrogenase 3 (NAD(+)) gamma | 314.0 | 308.0 | **-1.01** | 0.95 |
| OGDH | oxoglutarate dehydrogenase | 1724.2 | 1717.0 | **-1.01** | 0.99 |
| DLST | dihydrolipoamide S-succinyltransferase | 1070.3 | 1360.6 | **1.27** | < 0.0001 |
| DLD | dihydrolipoamide dehydrogenase | 2534.3 | 2304.7 | **-1.10** | 0.0027 |
| SUCLA2 | succinate-CoA ligase ADP-forming beta subunit | 1686.9 | 1527.1 | **-1.10** | 0.0037 |
| SUCLG1 | succinate-CoA ligase alpha subunit | 1040.6 | 1111.0 | **1.07** | 0.36 |
| SUCLG2 | succinate-CoA ligase GDP-forming beta subunit | 1880.9 | 1844.3 | **-1.02** | 0.89 |
| SDHA | succinate dehydrogenase complex flavoprotein subunit A | 3179.9 | 2181.8 | **-1.42** | < 0.0001 |
| SDHB | succinate dehydrogenase complex iron sulfur subunit B | 1171.6 | 1098.9 | **-1.06** | 0.31 |
| SDHC | succinate dehydrogenase complex subunit C | 666.7 | 776.0 | **1.15** | 0.0035 |
| SDHD | succinate dehydrogenase complex subunit D | 968.2 | 961.0 | **-1.01** | 0.95 |
| FH | fumarate hydratase | 1795.4 | 1572.0 | **-1.14** | 0.014 |
| MDH1 | malate dehydrogenase 1 | 2182.5 | 1853.9 | **-1.17** | < 0.0001 |
| MDH2 | malate dehydrogenase 2 | 1043.1 | 1038.6 | **-1.01** | 0.99 |
| ***Lipolysis*** | | | | | |
| **Gene** | **Name** | **Mean (Co)** | **Mean (D)** | **Fold change** | **FDR *p*** |
| MGLL | monoglyceride lipase | 1521.7 | 2062.5 | **1.35** | < 0.0001 |
| LIPA | lipase A, lysosomal acid type | 542.5 | 639.8 | **1.18** | 0.0025 |
| LIPH | lipase H | 32.0 | 54.0 | **1.61** | 0.014 |
| DAGLA | diacylglycerol lipase alpha | 15.4 | 12.0 | **-1.25** | 0.84 |
| LIPJ | lipase family member J | 6.8 | 4.2 | **-1.34** | 0.84 |
| LIPC | lipase C, hepatic type | 11.7 | 10.0 | **-1.15** | 0.89 |
| DAGLB | diacylglycerol lipase beta | 744.5 | 776.6 | **1.05** | 0.84 |
| LIPG | lipase G, endothelial type | 22.1 | 27.3 | **1.26** | 0.67 |
| LIPF | lipase F, gastric type | 0.7 | 0.3 | **-1.08** | 0.93 |
| LIPI | lipase I | 3.1 | 2.4 | **-1.15** | 0.93 |
| LIPE | lipase E, hormone sensitive type | 13.3 | 13.0 | **-1.02** | 0.99 |
| ***Beta-oxidation*** | | | | | |
| **Gene** | **Name** | **Mean (Co)** | **Mean (D)** | **Fold change** | **FDR *p*** |
| ACADVL | acyl-CoA dehydrogenase, very long chain | 3674.9 | 2904.4 | **-1.27** | < 0.0001 |
| ACADM | acyl-CoA dehydrogenase, C-4 to C-12 straight chain | 1982.2 | 1783.8 | **-1.11** | 0.0040 |
| ACAD8 | acyl-CoA dehydrogenase family member 8 | 485.7 | 485.4 | **1.00** | 1.0 |
| ACAD9 | acyl-CoA dehydrogenase family member 9 | 897.6 | 834.9 | **-1.08** | 0.17 |
| ACAD10 | acyl-CoA dehydrogenase family member 10 | 151.9 | 134.6 | **-1.13** | 0.36 |
| ACAD11 | acyl-CoA dehydrogenase family member 11 | 976.1 | 1010.7 | **1.04** | 0.84 |
| ACADSB | acyl-CoA dehydrogenase, short/branched chain | 464.4 | 484.4 | **1.04** | 0.84 |
| ACADS | acyl-CoA dehydrogenase, C-2 to C-3 short chain | 50.2 | 52.8 | **1.06** | 0.93 |
| **ACADL** | **acyl-CoA dehydrogenase, long chain** | 106.2 | 288.2 | **2.58** | < 0.0001 |
| EHHADH | enoyl-CoA hydratase and 3-hydroxyacyl CoA dehydrogenase | 361.2 | 538.4 | **1.46** | < 0.0001 |
| HADH | hydroxyacyl-CoA dehydrogenase | 584.3 | 619.6 | **1.06** | 0.75 |
| HADHB | hydroxyacyl-CoA dehydrogenase/3-ketoacyl-CoA thiolase/enoyl-CoA hydratase (trifunctional protein), beta subunit | 2070.6 | 2661.3 | **1.28** | < 0.0001 |

FDR *p* = False discovery rate -corrected *p*-value

Red = upregulated genes

**Table S6: Effects of dexamethasone on genes linked to OA in previous GWAS studies [21-23]**

| **Gene** | **Name** | **Function** | **Mean (Co)** | **Mean (D)** | **Fold change** | **FDR *p*-value** |
| --- | --- | --- | --- | --- | --- | --- |
| ADAM12 | **ADAM metalloproteinase domain 12** | ECM catabolism | 1359.7 | 509.3 | **-2.68** | <0.0001 |
| ADAMTS14 | ADAM metalloproteinase with thrombospondin type 1 motif 14 | ECM catabolism | **-** | **-** | **-** | - |
| ASPN | **Asporin** | Regulation of chondrogenesis | 1728.1 | 577.8 | **-2.62** | <0.0001 |
| ASTN2 | Astrotactin 2 | Regulation of bone formation | 27.2 | 16.6 | **-1.55** | 0.090 |
| BAG6 | BCL2 associated athanogene 6 | Regulation of apoptosis | 1228.1 | 1163.6 | **-1.06** | 0.63 |
| BCAP29 | B-cell receptor associated protein 29 | Osteoblast differentiation | 4388.0 | 4130.9 | **-1.06** | 0.22 |
| BTNL2 | Butyrophilin like 2 | Regulation of inflammation | **-** | **-** |  |  |
| C9orf3 | Chromosome 9 open reading frame 3 | ? | 3363.6 | 2059.0 | **-**  **-1.62** | < 0.0001 |
| C6Orf130 = OARD1 | O-acyl-ADP-ribose deacylase 1 | Purine metabolism | **-** | **-** | **-** | - |
| CDC5L | Cell division cycle 5 like | Regulation of cell division | 2523.0 | 3026.8 | **1.20** | < 0.0001 |
| CHST11 | **Carbohydrate sulfotransferase 11** | Proteoglycan synthesis | 771.5 | 274.9 | **-2.69** | < 0.0001 |
| COL9A3 | Collagen type IX alpha 3 chain | ECM component | 2746.7 | 2328.8 | **-1.20** | 0.0025 |
| COL11A1 | **Collagen type XI alpha 1 chain** | ECM component | 59852.0 | 19522.6 | **-3.12** | < 0.0001 |
| COL6A4P1 | Collagen type VI alpha 4 pseudogene 1 | ECM component -like pseudogene | **-** | **-** | **-** | - |
| COG5 | Component of oligomeric golgi complex 5 | Protein processing | 2231.2 | 2223.4 | **-1.01** | 0.96 |
| COX-2 | **Cyclooxygenase-2** | Inflammation | 1232.1 | 280.9 | **-4.29** | < 0.0001 |
| DIO2 | **Iodothyronine deiodinase 2** | Thyroxine metabolism | 1663.5 | 311.0 | **-5.21** | < 0.0001 |
| DOT1L | DOT1 like histone lysine methyltransferase | Regulation of gene expression | 154.3 | 140.7 | **-1.09** | 0.84 |
| DUS4L | Dihydrouridine synthase 4 like | ? | 186.5 | 154.7 | **-1.21** | 0.059 |
| ESR1 | Estrogen receptor 1 | Estrogen signaling | 318.5 | 424.6 | **1.32** | 0.00082 |
| ESR2 | Estrogen receptor 2 | Estrogen signaling | 11.1 | 11.9 | **1.06** | 0.95 |
| FILIP1L | Filamin A interacting protein 1 like | Regulation of ECM catabolism | 62.2 | 32.7 | **-1.88** | < 0.0001 |
| FTO | FTO, alpha-ketoglutarate dependent dioxygenase | Lipid metabolism | 1464.0 | 1369.4 | **-1.07** | 0.23 |
| GDF5 | **Growth differentiation factor 5** | Skeletal system development | 474.1 | 229.3 | **-2.11** | < 0.0001 |
| GLT8D1 | Glycosyltransferase 8 domain containing 1 | ? | 1206.1 | 1561.2 | **1.29** | < 0.0001 |
| GNL3 | G protein nucleolar 3 | Regulation of cell proliferation | 3488.8 | 3389.5 | **-1.03** | 0.84 |
| GPR22 | G protein-coupled receptor 22 | ? | 13.8 | 13.1 | **-1.06** | 0.96 |
| HBP1 | HMG-box transcription factor 1 | Regulation of cell proliferation and apoptosis | 2267.8 | 2609.3 | **1.13** | 0.0067 |
| HIF1A | Hypoxia inducible factor 1 alpha subunit | Response to hypoxia | 28876.9 | 15036.6 | **-1.95** | < 0.0001 |
| HLA-DQB1 | HLA-DQB1 antisense RNA 1 | Regulation of inflammation | 14.1 | 21.0 | **1.33** | 0.75 |
| IGFBP3 | **Insulin like growth factor binding protein 3** | Regulation of cell proliferation | 89825.2 | 24371.5 | **-3.81** | 1.14E-74 |
| IL6 | **Interleukin 6** | Inflammation | 25.8 | 3.9 | **-3.86** | < 0.0001 |
| IL16 | **Interleukin 16** | Inflammation | 573.4 | 1724.3 | **2.95** | < 0.0001 |
| IL17A | Interleukin 17A | Inflammation | **-** | **-** | **-** | - |
| IL1RN | Interleukin 1 receptor antagonist | Regulation of inflammation | **-** | **-** | **-** | - |
| INSR | Insulin receptor | Carbohydrate metabolism | 815.1 | 1153.6 | **1.40** | 9.38E-17 |
| KLHDC5 | Kelch domain containing 5 = Kelch like family member 42 | Cell proliferation | 875.6 | 1134.1 | **1.29** | < 0.0001 |
| =KLHL42 |  |  |  |  |  |  |
| LHCGR | Luteinizing hormone/choriogonadotropin receptor | Sex hormone signaling | 10.3 | 7.7 | **-1.27** | 0.84 |
| LRCH1 | Leucine rich repeats and calponin homology domain containing 1 | Regulation of inflammation | 391.5 | 372.4 | **-1.06** | 0.84 |
| MCF2L | MCF.2 cell line derived transforming sequence like | Regulation of cell proliferation and apoptosis | 6.9 | 4.3 | **-1.43** | 0.66 |
| MICAL3 | Microtubule associateda monooxygenase, calponin and LIM domain containing 3 | Cytoskeleton organization | 496.7 | 435.3 | **-1.15** | 0.022 |
| MMP8 | Matrix metalloproteinase 8 | ECM catabolism | **-** | **-** | **-** | - |
| NCOA3 | nuclear receptor coactivator 3 | Regulation of transcription | 2482.4 | 4651.1 | **1.87** | < 0.0001 |
| PAPPA | Pappalysin 1 | IGF signaling | 1064.4 | 1372.3 | **1.27** | < 0.0001 |
| PTHLH | Parathyroid hormone like hormone | Regulation of chondrocyte differentiation | 31.6 | 42.0 | **1.27** | 0.53 |
| SENP6 | SUMO1/sentrin specific peptidase 6 | Protein sumoylation | 4100.3 | 4182.2 | **1.02** | 0.84 |
| SMAD3 | SMAD family member 3 | Regulation of chondrocyte differentiation | 1007.2 | 839.7 | **-1.20** | 0.00020 |
| SUPT3H | SPT3 homolog, SAGA and STAGA complex component | ? | 146.2 | 155.0 | **1.04** | 0.92 |
| TGFB1 | Transforming growth factor beta 1 | Skeletal system development | 704.9 | 531.5 | **-1.32** | <0.0001 |
| TP63 | Tumor protein p63 | Regulation of autophagy | **-** | **-** | **-** | - |
| VDR | **Vitamin D (1,25- dihydroxyvitamin D3) receptor** | Regulation of transcription | 317.2 | 140.8 | **-2.25** | < 0.0001 |
| VEGFA | **Vascular endothelial growth factor A** | Regulation of chondrocyte viability and ECM production | 8007.4 | 3366.9 | **-2.36** | < 0.0001 |
| VEGFC | Vascular endothelial growth factor C | Regulation of cell proliferation | 158.8 | 80.7 | **-1.92** | < 0.0001 |

Co = Control OA chondrocytes

D = Dexamethasone-treated OA chondrocytes

FDR *p*-value = False discovery rate -corrected *p*-value

Red = upregulated genes

Blue = downregulated genes

**Table S7: Effects of dexamethasone on genes previously linked to OA in the GWEA study by Ramos et al. [9]**

| **Gene** | **Name** | **Mean (Co)** | **Mean (D)** | **Fold change** | **FDR**  ***p-*value** | **FC (Ramos et al.)** | **FDR**  **(Ramos et al.)** |
| --- | --- | --- | --- | --- | --- | --- | --- |
| TNFAIP6 | TNF alpha induced protein 6 | 1054.4 | 772.3 | **-1.35** | < 0.0001 | 4.01 | 0.0004 |
| CRLF1 | Cytokine receptor like factor 1 | 411.4 | 484.2 | **1.17** | 0.029 | 4.01 | < 0.0001 |
| P3H2 | Prolyl 3-hydroxylase 2 | 23211.8 | 16599.2 | **-1.40** | < 0.0001 | 2.88 | 0.0009 |
| CD55 | CD55 molecule (Cromer blood group) | 22856.9 | 14240.9 | **-1.56** | < 0.0001 | 3.00 | < 0.0001 |
| RARRES2 | Retinoic acid receptor responder 2 | 35.0 | 35.4 | **1.01** | 0.99 | -3.32 | 0.0001 |
| NGF | **Nerve growth factor** | 129.1 | 16.2 | **-6.92** | < 0.0001 | 4.54 | < 0.0001 |
| FRZB | Frizzled-related protein | 2615.8 | 2519.3 | **-1.04** | 0.87 | -3.04 | 0.0004 |
| SERPINE1 | Serpin family E member 1 | 14774.0 | 16781.4 | **1.11** | 0.55 | 3.13 | 0.0002 |
| PTGES | Prostaglandin E synthase | 298.9 | 242.1 | **-1.23** | 0.081 | 3.29 | < 0.0001 |
| SPP1 | Secreted phosphoprotein 1 | 52263.3 | 37784.5 | **-1.30** | 0.0012 | 2.56 | 0.0071 |
| CXCL14 | C-X-C motif chemokine ligand 14 | 48.7 | 58.2 | **-1.03** | 0.97 | 4.97 | 0.0001 |
| TNFRSF11B | TNF receptor superfamily member 11b | 71281.8 | 51916.0 | **-1.33** | 0.020 | 2.69 | 0.0007 |
| FN1 | Fibronectin 1 | 1195480.5 | 1570744.5 | **1.33** | < 0.0001 | 1.52 | 0.033 |
| HBA2 | Hemoglobin subunit alpha 2 | 0.6 | 1.8 | **1.39** | 0.78 | 2.97 | 0.0062 |
| HBB | Hemoglobin subunit beta | 3.8 | 5.9 | **1.27** | 0.84 | 2.50 | 0.017 |
| PAPPA | Pappalysin 1 | 1064.4 | 1372.3 | **1.27** | < 0.0001 | 2.25 | 0.0041 |
| CRISPLD1 | **Cysteine rich secretory protein LCCL domain containing 1** | 1014.9 | 408.6 | **-2.39** | < 0.0001 | – | 0.75 |
| COL9A1 | **Collagen, type IX, alpha 1** | 678.5 | 176.2 | **-3.66** | < 0.0001 | -3.14 | 0.0002 |
| CHRDL2 | Chordin like 2 | 42.4 | 46.6 | **1.09** | 0.92 | -5.33 | 0.0006 |

Co = Control OA chondrocytes

D = Dexamethasone-treated OA chondrocytes

FC = Fold change

FDR *p*-value = False discovery rate -corrected *p*-value

Red = upregulated genes

Blue = downregulated genes

**Table S8: Effects of dexamethasone on genes previously linked to OA cartilage in the GWEA study by Almeida et al. [10]**

| **Gene** | **Name** | | **FC (Almeida et al.)** | **FDR *p* (Almeida)** | **Mean (Co** **) (our study)** | **Mean (D)**  **(our study)** | **FC (our study)** | **adj. *p* (our study)** |
| --- | --- | --- | --- | --- | --- | --- | --- | --- |
| ***Genes upregulated in degraded cartilage (Almeida et al. [10]) and upregulated by dexamethasone (our study)*** | | | | | | | | |
| KLRD1 | Killer Cell Lectin Like Receptor D1 | | **2.50** | 2.48E-05 | 15.1 | 685.0 | **29.24** | 3.57E-73 |
| WNT16 | Wnt Family Member 16 | | **8.48** | 1.10E-13 | 11.3 | 134.6 | **8.51** | 2.09E-29 |
| CLVS2 | Clavesin 2 | | **2.15** | 1.34E-07 | 278 | 1731.3 | **6.28** | 0.00E+00 |
| DUSP4 | Dual Specificity Phosphatase 4 | | **2.82** | 4.84E-08 | 227.2 | 1198.1 | **5.50** | 5.08E-46 |
| TRIM36 | Tripartite Motif Containing 36 | | **2.85** | 1.08E-08 | 72.7 | 385.8 | **5.21** | 1.15E-62 |
| SLC7A2 | Solute Carrier Family 7 Member 2 | | **2.10** | 1.02E-08 | 7546.7 | 34380.9 | **4.59** | 1.90E-74 |
| AOC2 | Amine Oxidase, Copper Containing | | **2.23** | 3.57E-10 | 769.4 | 3114.5 | **4.06** | 1.21E-200 |
| DSC3 | Desmocollin 3 | | **2.35** | 1.34E-04 | 1000.2 | 3244.1 | **3.25** | 2.86E-135 |
| AFAP1L1 | Actin Filament Associated Protein 1 Like 1 | | **2.08** | 9.44E-04 | 13.9 | 46.3 | **2.99** | 1.16E-09 |
| AOC3 | Amine Oxidase, Copper Containing | | **2.00** | 2.11E-07 | 141.4 | 429.3 | **2.99** | 8.49E-48 |
| DAW1 | Dynein Assembly Factor With Wd Repeats 1 | | **2.57** | 2.55E-05 | 28.4 | 84.7 | **2.87** | 2.11E-16 |
| OR7E14P | Olfactory Receptor Family 7 Subfamily E Member 14 | | **3.50** | 2.10E-04 | 5.4 | 19.6 | **2.85** | 2.57E-05 |
| ZNF474 | Zinc Finger Protein 474 | | **2.27** | 8.22E-03 | 6.5 | 20.8 | **2.71** | 1.00E-05 |
| DSG3 | Desmoglein 3 | | **7.81** | 1.05E-05 | 3.8 | 13.1 | **2.58** | 5.60E-04 |
| FSTL3 | Follistatin Like 3 | | **2.25** | 2.05E-09 | 98.4 | 263.6 | **2.58** | 3.85E-32 |
| GALNT13 | Polypeptide N-Acetylgalactosaminyltransferase 13 | | **2.10** | 9.15E-05 | 107.4 | 271.1 | **2.51** | 2.64E-33 |
| DNER | Delta/Notch Like Egf Repeat Containing | | **3.37** | 4.24E-11 | 550.9 | 1328.3 | **2.39** | 1.89E-13 |
| ANK3 | Ankyrin | | **2.62** | 6.02E-10 | 3804.7 | 8718.8 | **2.30** | 7.45E-116 |
| CYFIP2 | Cytoplasmic Fmr1 Interacting Protein 2 | | **2.05** | 9.36E-08 | 36.8 | 77.4 | **2.04** | 1.22E-05 |
| ***Genes upregulated in degraded cartilage (Almeida et al. [10]) and downregulated by dexamethasone (our study)*** | | | | | | | | |
| INHBA | Inhibin Subunit Beta A | | **2.36** | 2.13E-08 | 26339.5 | 2220.8 | **-11.71** | 0.00E+00 |
| TSPAN2 | Tetraspanin 2 | | **2.42** | 1.51E-08 | 1192.6 | 154.4 | **-7.11** | 1.66E-66 |
| NGF | Nerve Growth Factor | | **4.91** | 2.53E-14 | 129.1 | 16.2 | **-6.92** | 7.82E-29 |
| NOG | Noggin | | **2.08** | 2.05E-05 | 316.5 | 52.3 | **-5.78** | 8.65E-43 |
| MIR31HG | Mir31 Host Gene | | **2.01** | 1.26E-03 | 104.0 | 17.5 | **-5.31** | 2.78E-20 |
| ATP6V0A4 | | Atpase H+ Transporting V0 Subunit A | **2.59** | 8.26E-04 | 29.8 | 3.8 | **-5.03** | 8.08E-11 |
| IGFBP3 | | Insulin Like Growth Factor Binding Protein 3 | **2.65** | 1.12E-07 | 89825.2 | 24371.5 | **-3.81** | 5.70E-75 |
| MYBL2 | | Myb Proto-Oncogene Like 2 | **2.13** | 1.13E-02 | 38.9 | 8.9 | **-3.61** | 7.08E-09 |
| BTBD16 | | Btb Domain Containing 16 | **2.21** | 9.10E-06 | 23.6 | 4.7 | **-3.53** | 2.83E-06 |
| IGFBP1 | | Insulin Like Growth Factor Binding Protein 1 | **7.59** | 1.32E-09 | 157.1 | 42.8 | **-3.41** | 2.88E-13 |
| LIF | | Lif, Interleukin 6 Family Cytokine | **4.91** | 7.79E-10 | 477.6 | 142.3 | **-3.18** | 7.19E-33 |
| SHC4 | | Shc Adaptor Protein 4 | **2.10** | 5.87E-09 | 902.4 | 328.6 | **-2.81** | 5.71E-44 |
| GRIA2 | | Glutamate Ionotropic Receptor Ampa Type Subunit 2 | **2.03** | 6.05E-04 | 75.7 | 24.9 | **-2.79** | 4.00E-14 |
| SHISA9 | | Shisa Family Member 9 | **2.73** | 4.47E-05 | 46.1 | 15.2 | **-2.71** | 1.29E-08 |
| IL11 | | Interleukin 11 | **22.80** | 1.53E-20 | 30.4 | 8.2 | **-2.58** | 5.76E-05 |
| C15orf48 | | Chromosome 15 Open Reading Frame 48 | **2.01** | 1.56E-02 | 32.1 | 11.4 | **-2.33** | 1.26E-04 |
| S100A3 | | S100 Calcium Binding Protein A3 | **2.10** | 1.25E-05 | 83.6 | 35.7 | **-2.27** | 3.30E-07 |
| PIMREG | | Picalm Interacting Mitotic Regulator | **2.02** | 1.64E-02 | 148.6 | 67.8 | **-2.25** | 5.46E-09 |
| RIPK4 | | Receptor Interacting Serine/Threonine Kinase 4 | **5.22** | 5.45E-13 | 39.8 | 17.9 | **-2.22** | 4.69E-05 |
| UROC1 | | Urocanate Hydratase 1 | **2.41** | 6.74E-08 | 14.3 | 5.2 | **-2.19** | 4.53E-03 |
| SKA1 | | Spindle And Kinetochore Associated Complex Subunit 1 | **2.32** | 4.47E-02 | 195.5 | 91.0 | **-2.19** | 3.11E-15 |
| TGFBI | | Transforming Growth Factor Beta Induced | **2.14** | 1.11E-03 | 3222.9 | 1495.3 | **-2.19** | 3.63E-16 |
| DNAJC22 | | Dnaj Heat Shock Protein Family (Hsp40) Member C22 | **2.02** | 1.90E-08 | 201.1 | 90.9 | **-2.19** | 7.80E-17 |
| BIRC5 | | Baculoviral Iap Repeat Containing | **2.30** | 2.12E-03 | 427.4 | 224.2 | **-2.04** | 1.38E-14 |
| CLIC3 | | Chloride Intracellular Channel 3 | **3.65** | 1.81E-11 | 32.9 | 15.7 | **-2.01** | 2.07E-03 |
| ***Genes downregulated in degraded cartilage (Almeida et al. [10]) and downregulated by dexamethasone (our study)*** | | | | | | | | |
| GREM1 | Gremlin 1, Dan Family Bmp Antagonist | | **-2.65** | 2.57E-05 | 6327.8 | 691.8 | **-8.22** | 1.55E-63 |
| SLC26A4 | Solute Carrier Family 26 Member 4 | | **-2.13** | 5.52E-06 | 1537.7 | 180.9 | **-7.73** | 2.50E-56 |
| RFLNA | Refilin A | | **-2.04** | 6.48E-05 | 356.5 | 87.2 | **-4.00** | 1.09E-72 |
| BMPER | Bmp Binding Endothelial Regulator | | **-2.13** | 9.36E-06 | 447.1 | 110.9 | **-3.84** | 2.59E-41 |
| PIEZO2 | Piezo Type Mechanosensitive Ion Channel Component 2 | | **-2.28** | 3.57E-07 | 2106.9 | 548.9 | **-3.81** | 7.71E-117 |
| COL9A1 | Collagen Type Ix Alpha 1 Chain | | **-2.41** | 4.27E-03 | 678.5 | 176.2 | **-3.66** | 3.90E-71 |
| SHISA3 | Shisa Family Member 3 | | **-3.67** | 3.14E-08 | 485 | 134.5 | **-3.56** | 1.94E-78 |
| SPTSSB | Serine Palmitoyltransferase Small Subunit B | | **-2.49** | 4.08E-07 | 178.8 | 50.1 | **-3.48** | 3.19E-23 |
| LRRTM2 | Leucine Rich Repeat Transmembrane Neuronal 2 | | **-2.25** | 5.42E-04 | 59.3 | 20.5 | **-2.64** | 1.06E-07 |
| ERICH3 | Glutamate Rich 3 | | **-2.56** | 2.32E-02 | 31.3 | 10 | **-2.62** | 2.51E-04 |
| PTGER3 | Prostaglandin E Receptor 3 | | **-3.08** | 4.91E-08 | 485.3 | 183.4 | **-2.62** | 9.54E-41 |
| DACT1 | Dishevelled Binding Antagonist Of Beta Catenin 1 | | **-2.68** | 2.85E-07 | 416.3 | 165.8 | **-2.46** | 5.13E-15 |
| ZNF385C | Zinc Finger Protein 385C | | **-2.32** | 2.30E-06 | 109.7 | 43.5 | **-2.41** | 5.86E-13 |
| CRISPLD1 | Cysteine Rich Secretory Protein Lccl Domain Containing 1 | | **-2.76** | 9.29E-06 | 1014.9 | 408.6 | **-2.39** | 6.13E-25 |
| CNTFR | Ciliary Neurotrophic Factor Receptor | | **-3.24** | 1.56E-09 | 55.8 | 23.5 | **-2.23** | 8.81E-08 |
| RNF150 | Ring Finger Protein 150 | | **-2.11** | 6.85E-04 | 22.5 | 9.6 | **-2.07** | 4.40E-03 |
| ZFHX4-AS1 | Zfhx4 Antisense Rna 1 | | **-2.65** | 2.07E-02 | 21.4 | 8.8 | **-2.07** | 4.90E-03 |
| ***Genes downregulated in degraded cartilage (Almeida et al. [10]) and downregulated by dexamethasone (our study)*** | | | | | | | | |
| PDZRN4 | Pdz Domain Containing Ring Finger 4 | | **-2.58** | 7.76E-04 | 3.3 | 184.8 | **12.55** | 2.82E-18 |
| SAA1 | Serum Amyloid A1 | | **-2.40** | 3.04E-03 | 57 | 1094.9 | **12.13** | 1.25E-27 |
| RSPO3 | R-Spondin 3 | | **-3.62** | 1.48E-06 | 57.3 | 584.8 | **9.58** | 2.35E-165 |
| WISP2 | Wnt1 Inducible Signaling Pathway Protein 2 | | **-2.30** | 3.06E-03 | 2.9 | 45.7 | **9.45** | 2.18E-21 |
| KIT | Kit Proto-Oncogene Receptor Tyrosine Kinase | | **-2.58** | 5.18E-04 | 5.6 | 58.7 | **6.54** | 1.81E-14 |
| APOD | Apolipoprotein | | **-2.98** | 1.70E-10 | 638.3 | 3819.4 | **6.15** | 2.65E-222 |
| METTL7A | Methyltransferase Like 7A | | **-2.15** | 1.54E-06 | 2409.8 | 11859.9 | **4.92** | 0.00E+00 |
| SLC14A2 | Solute Carrier Family 14 Member 2 | | **-2.29** | 6.02E-03 | 5.7 | 31.5 | **4.17** | 2.87E-09 |
| CYP4F22 | Cytochrome P450 Family 4 Subfamily F Member 22 | | **-2.38** | 1.35E-03 | 6.5 | 32.8 | **4.08** | 1.12E-12 |
| TRABD2B | Trab Domain Containing 2B | | **-2.02** | 1.34E-07 | 13.8 | 61.7 | **4.03** | 1.07E-16 |
| PRLR | Prolactin Receptor | | **-2.55** | 1.94E-02 | 8.1 | 37.5 | **3.61** | 9.75E-09 |
| ALDH1L1 | Aldehyde Dehydrogenase 1 Family Member L1 | | **-2.03** | 7.24E-07 | 27.1 | 94.3 | **3.29** | 1.38E-23 |
| HS6ST3 | Heparan Sulfate 6-O-Sulfotransferase 3 | | **-2.16** | 6.80E-03 | 12.7 | 46.2 | **2.97** | 1.40E-06 |
| AQP7P2 | Aquaporin 7 Pseudogene 2 | | **-2.43** | 1.93E-02 | 11.2 | 40.2 | **2.91** | 4.98E-07 |
| ACADL | Acyl-CoA Dehydrogenase Long Chain | | **-2.26** | 2.53E-05 | 106.2 | 288.2 | **2.58** | 2.11E-27 |
| STC2 | Stanniocalcin 2 | | **-2.04** | 1.24E-06 | 1899.6 | 4253.4 | **2.19** | 5.74E-19 |
| FMO2 | Flavin Containing Monooxygenase 2 | | **-2.64** | 2.20E-03 | 4.0 | 11.9 | **2.19** | 1.17E-02 |

D = Dexamethasone-treated OA chondrocytes

Co = Control OA chondrocytes

FC = Fold change

FDR *p* = False discovery rate –corrected *p*-value

Red = upregulated genes

Blue = downregulated genes

**Figures**

**
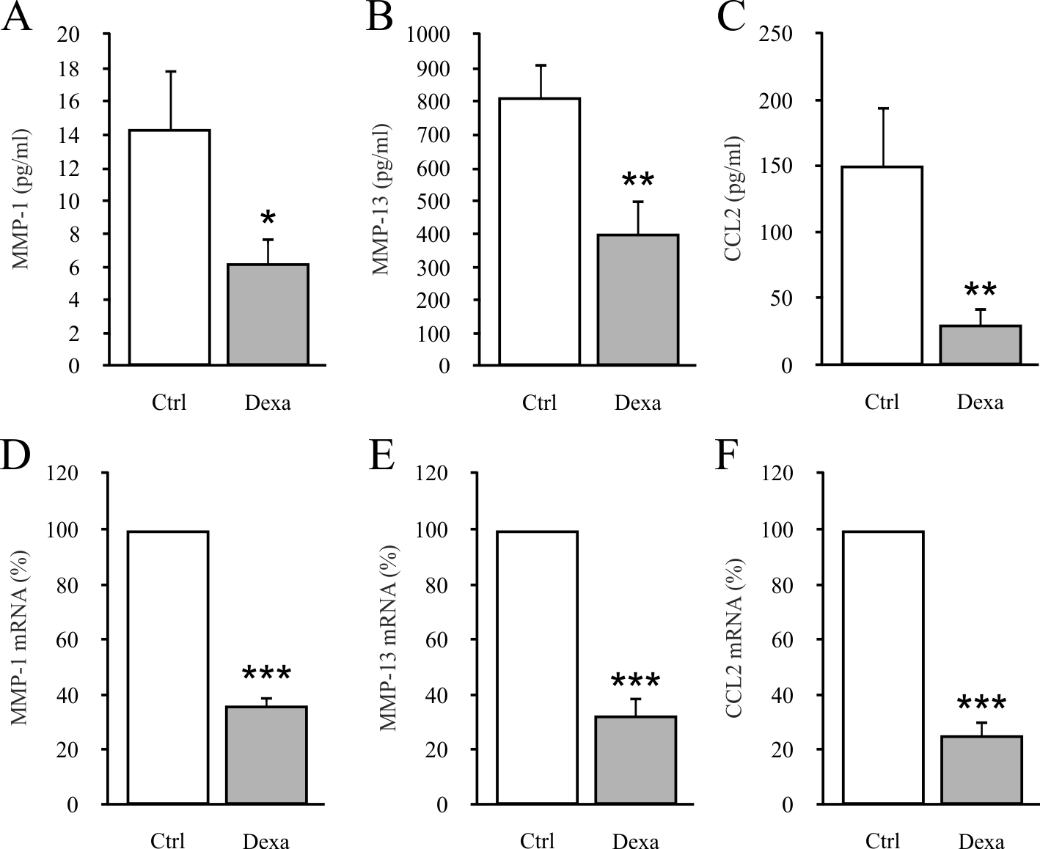
**

**Figure S1: Effects of dexamethasone on the production of catabolic and proinflammatory factors in OA chondrocytes.** OA chondrocytes / chondrocytes isolated from OA patients were cultured for 24 h with or without dexamethasone (1 µM). MMP-1 (A), MMP-13 (B) and CCL2 (C) levels in the culture media were determined with ELISA. MMP-1 (D), MMP-13 (E) and CCL2 (F) mRNA expression was studied with quantitative RT-PCR and normalized against GAPDH. The results were compared against control, which was set as 100 %. The results are expressed as mean + SEM, n = 9. *: p < 0.05, **: *p* < 0.01 and ***: *p* < 0.001, compared to the untreated control.
