# Supplementary material for: Widespread regulation of gene expression by glucocorticoids in chondrocytes from patients with osteoarthritis as determined by RNA-Seq
Source: Arthritis Res Ther. 2020 Nov 17;22:271. doi: 10.1186/s13075-020-02289-7 (PMC7670667; doi:10.1186/s13075-020-02289-7)
Supplement: Supplementary file 2 — Additional file 2: Table S2. All genes differentially expressed in dexamethasone-treated OA chondrocytes (D) relative to controls (Co). [file 13075_2020_2289_MOESM2_ESM.pdf]

Gene	Name	Mean (Co)	Mean (D)	Fold Change	FDR *p*
GPM6B	glycoprotein M6B [Source:HGNC Symbol;Acc:HGNC:4461]	205.4	8033.4	**37.53**	0
SULT1B1	sulfotransferase family 1B member 1 [Source:HGNC Symbol;Acc:HGNC:17845]	14.5	702.2	**34.30**	1.62E-86
KLRD1	killer cell lectin like receptor D1 [Source:HGNC Symbol;Acc:HGNC:6378]	15.1	685.0	**29.24**	3.57E-73
FLRT3	fibronectin leucine rich transmembrane protein 3 [Source:HGNC Symbol;Acc:HGNC:3762]	6.9	247.4	**27.28**	1.06E-89
FAM83A-AS1	FAM83A antisense RNA 1 [Source:HGNC Symbol;Acc:HGNC:48658]	0.4	43.6	**21.41**	2.18E-31
ZBTB16	zinc finger and BTB domain containing 16 [Source:HGNC Symbol;Acc:HGNC:12930]	113.9	2490.2	**21.11**	0
FKBP5	FK506 binding protein 5 [Source:HGNC Symbol;Acc:HGNC:3721]	702.8	15089.3	**20.11**	0
PDK4	pyruvate dehydrogenase kinase 4 [Source:HGNC Symbol;Acc:HGNC:8812]	251.6	5804.9	**19.84**	7.78E-125
PTK2B	protein tyrosine kinase 2 beta [Source:HGNC Symbol;Acc:HGNC:9612]	116.6	2393.8	**19.43**	0
ABCD2	ATP binding cassette subfamily D member 2 [Source:HGNC Symbol;Acc:HGNC:66]	13.8	340.3	**18.77**	3.53E-91
HAL	histidine ammonia-lyase [Source:HGNC Symbol;Acc:HGNC:4806]	5.5	176.0	**16.34**	1.40E-32
GLUL	glutamate-ammonia ligase [Source:HGNC Symbol;Acc:HGNC:4341]	3886.9	64025.9	**15.67**	0
TFCP2L1	transcription factor CP2 like 1 [Source:HGNC Symbol;Acc:HGNC:17925]	68.6	1265.0	**14.93**	3.17E-68
ADRA1B	adrenoceptor alpha 1B [Source:HGNC Symbol;Acc:HGNC:278]	0.1	23.7	**14.72**	7.07E-21
SLC38A4	solute carrier family 38 member 4 [Source:HGNC Symbol;Acc:HGNC:14679]	12.5	220.1	**14.32**	9.87E-76
ANGPTL5	angiopoietin like 5 [Source:HGNC Symbol;Acc:HGNC:19705]	638.2	9994.4	**13.18**	3.59E-80
MAOA	monoamine oxidase A [Source:HGNC Symbol;Acc:HGNC:6833]	19.1	297.4	**13.00**	3.87E-89
ZPLD1	zona pellucida like domain containing 1 [Source:HGNC Symbol;Acc:HGNC:27022]	37.3	523.7	**12.55**	2.32E-146
PDZRN4	PDZ domain containing ring finger 4 [Source:HGNC Symbol;Acc:HGNC:30552]	3.3	184.8	**12.55**	2.82E-18
SAA1	serum amyloid A1 [Source:HGNC Symbol;Acc:HGNC:10513]	57.0	1094.9	**12.13**	1.25E-27
KCNJ11	potassium voltage-gated channel subfamily J member 11 [Source:HGNC Symbol;Acc:HGNC:6257]	0.3	21.6	**12.13**	1.73E-18
CAMK2N1	calcium/calmodulin dependent protein kinase II inhibitor 1 [Source:HGNC Symbol;Acc:HGNC:24190]	37.5	507.7	**11.55**	1.04E-94
SULT1E1	sulfotransferase family 1E member 1 [Source:HGNC Symbol;Acc:HGNC:11377]	0.7	24.4	**11.47**	1.55E-19
KLF9	Kruppel like factor 9 [Source:HGNC Symbol;Acc:HGNC:1123]	582.2	6410.9	**10.85**	0
DUSP1	dual specificity phosphatase 1 [Source:HGNC Symbol;Acc:HGNC:3064]	695.1	7460.0	**10.48**	0
LINC01384	long intergenic non-protein coding RNA 1384 [Source:HGNC Symbol;Acc:HGNC:50656]	7.9	106.5	**10.48**	8.42E-40
ABCA6	ATP binding cassette subfamily A member 6 [Source:HGNC Symbol;Acc:HGNC:36]	102.4	1070.3	**9.99**	1.39E-196
RSPO3	R-spondin 3 [Source:HGNC Symbol;Acc:HGNC:20866]	57.3	584.8	**9.58**	2.35E-165
KLF15	Kruppel like factor 15 [Source:HGNC Symbol;Acc:HGNC:14536]	72.1	717.0	**9.51**	1.10E-183
WISP2	WNT1 inducible signaling pathway protein 2 [Source:HGNC Symbol;Acc:HGNC:12770]	2.9	45.7	**9.45**	2.18E-21
CMTM8	CKLF like MARVEL transmembrane domain containing 8 [Source:HGNC Symbol;Acc:HGNC:19179]	7.8	95.1	**9.38**	1.05E-30
CORO6	coronin 6 [Source:HGNC Symbol;Acc:HGNC:21356]	6.2	86.9	**9.38**	4.86E-26
PCBP3	poly(rC) binding protein 3 [Source:HGNC Symbol;Acc:HGNC:8651]	12.6	139.3	**9.19**	8.29E-42
SAA2	serum amyloid A2 [Source:HGNC Symbol;Acc:HGNC:10514]	5.0	111.5	**8.82**	1.30E-15
SPINK1	serine peptidase inhibitor, Kazal type 1 [Source:HGNC Symbol;Acc:HGNC:11244]	4.0	53.9	**8.75**	2.06E-22
STC1	stanniocalcin 1 [Source:HGNC Symbol;Acc:HGNC:11373]	4.9	68.0	**8.75**	9.02E-21
WNT16	Wnt family member 16 [Source:HGNC Symbol;Acc:HGNC:16267]	11.3	134.6	**8.51**	2.09E-29
SAA2-SAA4	SAA2-SAA4 readthrough [Source:HGNC Symbol;Acc:HGNC:39550]	2.2	48.7	**8.46**	3.50E-15
CAVIN2	caveolae associated protein 2 [Source:HGNC Symbol;Acc:HGNC:10690]	16.5	155.2	**8.34**	2.80E-57
TSC22D3	TSC22 domain family member 3 [Source:HGNC Symbol;Acc:HGNC:3051]	1101.3	8944.3	**7.73**	6.94E-108
LINC02015	long intergenic non-protein coding RNA 2015 [Source:HGNC Symbol;Acc:HGNC:52850]	13.9	121.2	**7.57**	3.75E-43
IL1R2	interleukin 1 receptor type 2 [Source:HGNC Symbol;Acc:HGNC:5994]	2.7	32.8	**7.52**	6.34E-17
PIK3R1	phosphoinositide-3-kinase regulatory subunit 1 [Source:HGNC Symbol;Acc:HGNC:8979]	2018.7	15847.0	**7.36**	1.37E-117
TNFAIP8L3	TNF alpha induced protein 8 like 3 [Source:HGNC Symbol;Acc:HGNC:20620]	278.5	2083.2	**7.26**	4.57E-242
SRPX	sushi repeat containing protein X-linked [Source:HGNC Symbol;Acc:HGNC:11309]	84.5	623.5	**7.16**	7.90E-126
FLVCR2	feline leukemia virus subgroup C cellular receptor family member 2 [Source:HGNC Symbol;Acc:HGNC:20	145.7	1097.1	**7.11**	5.47E-132
PLEKHA6	pleckstrin homology domain containing A6 [Source:HGNC Symbol;Acc:HGNC:17053]	14.4	205.6	**7.01**	9.92E-14
MYOT	myotilin [Source:HGNC Symbol;Acc:HGNC:12399]	49.0	353.5	**6.96**	1.46E-81
SCNN1A	sodium channel epithelial 1 alpha subunit [Source:HGNC Symbol;Acc:HGNC:10599]	22.1	173.9	**6.96**	1.81E-43
CCDC68	coiled-coil domain containing 68 [Source:HGNC Symbol;Acc:HGNC:24350]	72.7	534.7	**6.77**	1.21E-87
F2R	coagulation factor II thrombin receptor [Source:HGNC Symbol;Acc:HGNC:3537]	10.1	88.2	**6.68**	2.33E-23
KCNJ2	potassium voltage-gated channel subfamily J member 2 [Source:HGNC Symbol;Acc:HGNC:6263]	51.7	351.6	**6.54**	3.35E-72
KIT	KIT proto-oncogene receptor tyrosine kinase [Source:HGNC Symbol;Acc:HGNC:6342]	5.6	58.7	**6.54**	1.81E-14
IL6R	interleukin 6 receptor [Source:HGNC Symbol;Acc:HGNC:6019]	489.5	3192.7	**6.45**	0
CLTCL1	clathrin heavy chain like 1 [Source:HGNC Symbol;Acc:HGNC:2093]	104.0	693.2	**6.45**	4.13E-132
SSH2	slingshot protein phosphatase 2 [Source:HGNC Symbol;Acc:HGNC:30580]	226.6	1490.8	**6.41**	1.38E-222
SORBS1	sorbin and SH3 domain containing 1 [Source:HGNC Symbol;Acc:HGNC:14565]	648.8	4163.1	**6.36**	2.30E-113
CLVS2	clavesin 2 [Source:HGNC Symbol;Acc:HGNC:23046]	278.0	1731.3	**6.28**	0
RPGR	retinitis pigmentosa GTPase regulator [Source:HGNC Symbol;Acc:HGNC:10295]	309.7	1978.9	**6.28**	0
F3	coagulation factor III, tissue factor [Source:HGNC Symbol;Acc:HGNC:3541]	28.3	201.2	**6.28**	5.51E-34
AQP9	aquaporin 9 [Source:HGNC Symbol;Acc:HGNC:643]	5.4	47.7	**6.28**	3.41E-16
APOD	apolipoprotein D [Source:HGNC Symbol;Acc:HGNC:612]	638.3	3819.4	**6.15**	2.65E-222
IGSF10	immunoglobulin superfamily member 10 [Source:HGNC Symbol;Acc:HGNC:26384]	8.9	74.9	**5.94**	1.31E-14
MT3	metallothionein 3 [Source:HGNC Symbol;Acc:HGNC:7408]	0.4	15.8	**5.94**	1.37E-08
CEBPD	CCAAT enhancer binding protein delta [Source:HGNC Symbol;Acc:HGNC:1835]	650.8	3826.7	**5.86**	4.49E-264
ETNK2	ethanolamine kinase 2 [Source:HGNC Symbol;Acc:HGNC:25575]	71.2	422.2	**5.82**	1.29E-115
FOXA2	forkhead box A2 [Source:HGNC Symbol;Acc:HGNC:5022]	19.3	122.0	**5.78**	1.39E-35
EMP1	epithelial membrane protein 1 [Source:HGNC Symbol;Acc:HGNC:3333]	7038.6	38770.2	**5.54**	0
TFPI	tissue factor pathway inhibitor [Source:HGNC Symbol;Acc:HGNC:11760]	1133.4	6276.2	**5.54**	9.47E-99
DUSP4	dual specificity phosphatase 4 [Source:HGNC Symbol;Acc:HGNC:3070]	227.2	1198.1	**5.50**	5.08E-46

GADD45B	growth arrest and DNA damage inducible beta [Source:HGNC Symbol;Acc:HGNC:4096]	889.9	4844.8	**5.43**	4.68E-272
NFKBIZ	NFKB inhibitor zeta [Source:HGNC Symbol;Acc:HGNC:29805]	895.0	4920.6	**5.43**	1.02E-173
LINC00968	long intergenic non-protein coding RNA 968 [Source:HGNC Symbol;Acc:HGNC:48727]	63.9	353.0	**5.39**	4.05E-53
SRGN	serglycin [Source:HGNC Symbol;Acc:HGNC:9361]	1343.6	7156.1	**5.35**	5.51E-206
ITGB4	integrin subunit beta 4 [Source:HGNC Symbol;Acc:HGNC:6158]	19.5	124.3	**5.31**	4.55E-27
LRRC2	leucine rich repeat containing 2 [Source:HGNC Symbol;Acc:HGNC:14676]	63.6	336.4	**5.28**	9.68E-62
TRIM36	tripartite motif containing 36 [Source:HGNC Symbol;Acc:HGNC:16280]	72.7	385.8	**5.21**	1.15E-62
TDGF1	teratocarcinoma-derived growth factor 1 [Source:HGNC Symbol;Acc:HGNC:11701]	4.0	30.4	**5.21**	3.50E-12
APOB	apolipoprotein B [Source:HGNC Symbol;Acc:HGNC:603]	15.7	95.5	**5.17**	2.73E-20
GALNT15	polypeptide N-acetylgalactosaminyltransferase 15 [Source:HGNC Symbol;Acc:HGNC:21531]	2621.6	13535.1	**5.13**	9.95E-302
MT2A	metallothionein 2A [Source:HGNC Symbol;Acc:HGNC:7406]	12509.6	63465.7	**5.10**	0
ABLIM3	actin binding LIM protein family member 3 [Source:HGNC Symbol;Acc:HGNC:29132]	121.7	632.4	**5.10**	1.41E-105
CEACAM1	carcinoembryonic antigen related cell adhesion molecule 1 [Source:HGNC Symbol;Acc:HGNC:1814]	4.0	30.8	**5.06**	4.13E-10
MACC1-AS1	MACC1 antisense RNA 1 [Source:HGNC Symbol;Acc:HGNC:41257]	1.3	14.4	**5.06**	2.21E-08
FGR	FGR proto-oncogene, Src family tyrosine kinase [Source:HGNC Symbol;Acc:HGNC:3697]	4.6	34.7	**5.03**	7.41E-12
MT1X	metallothionein 1X [Source:HGNC Symbol;Acc:HGNC:7405]	1905.7	9951.1	**4.99**	1.14E-49
METTL7A	methyltransferase like 7A [Source:HGNC Symbol;Acc:HGNC:24550]	2409.8	11859.9	**4.92**	0
AGFG2	ArfGAP with FG repeats 2 [Source:HGNC Symbol;Acc:HGNC:5177]	290.8	1491.9	**4.92**	4.15E-118
MT1M	metallothionein 1M [Source:HGNC Symbol;Acc:HGNC:14296]	1108.5	5704.4	**4.92**	5.17E-43
MMP7	matrix metallopeptidase 7 [Source:HGNC Symbol;Acc:HGNC:7174]	3.3	29.0	**4.89**	5.00E-09
DEFB1	defensin beta 1 [Source:HGNC Symbol;Acc:HGNC:2766]	47.9	255.2	**4.82**	1.23E-34
NXPE2	neurexophilin and PC-esterase domain family member 2 [Source:HGNC Symbol;Acc:HGNC:26331]	34.8	182.8	**4.82**	3.43E-28
TLR2	toll like receptor 2 [Source:HGNC Symbol;Acc:HGNC:11848]	120.3	617.0	**4.72**	5.68E-28
ITGA9	integrin subunit alpha 9 [Source:HGNC Symbol;Acc:HGNC:6145]	326.6	1599.3	**4.69**	7.20E-102
ABCC9	ATP binding cassette subfamily C member 9 [Source:HGNC Symbol;Acc:HGNC:60]	24.6	139.0	**4.66**	9.03E-18
LINC02289	long intergenic non-protein coding RNA 2289 [Source:HGNC Symbol;Acc:HGNC:53205]	2.2	17.6	**4.66**	2.76E-08
SLC19A2	solute carrier family 19 member 2 [Source:HGNC Symbol;Acc:HGNC:10938]	184.4	853.9	**4.59**	2.01E-163
SLC7A2	solute carrier family 7 member 2 [Source:HGNC Symbol;Acc:HGNC:11060]	7546.7	34380.9	**4.59**	1.90E-74
RAB27B	RAB27B, member RAS oncogene family [Source:HGNC Symbol;Acc:HGNC:9767]	18.1	90.6	**4.59**	2.07E-25
OMD	osteomodulin [Source:HGNC Symbol;Acc:HGNC:8134]	2709.1	12303.8	**4.50**	3.83E-36
PDZK1IP1	PDZK1 interacting protein 1 [Source:HGNC Symbol;Acc:HGNC:16887]	3.2	23.0	**4.50**	4.58E-09
RASGEF1B	RasGEF domain family member 1B [Source:HGNC Symbol;Acc:HGNC:24881]	134.1	615.0	**4.44**	1.80E-71
TIMP4	TIMP metallopeptidase inhibitor 4 [Source:HGNC Symbol;Acc:HGNC:11823]	386.8	1717.0	**4.41**	3.45E-244
HSPB7	heat shock protein family B (small) member 7 [Source:HGNC Symbol;Acc:HGNC:5249]	69.3	312.8	**4.41**	7.60E-54
NYAP1	neuronal tyrosine phosphorylated phosphoinositide-3-kinase adaptor 1 [Source:HGNC Symbol;Acc:HGN	37.7	172.2	**4.41**	4.35E-41
S1PR1	sphingosine-1-phosphate receptor 1 [Source:HGNC Symbol;Acc:HGNC:3165]	7.7	43.5	**4.35**	3.65E-11
TRIM29	tripartite motif containing 29 [Source:HGNC Symbol;Acc:HGNC:17274]	12.3	59.7	**4.32**	2.85E-19
FOXO3	forkhead box O3 [Source:HGNC Symbol;Acc:HGNC:3821]	1541.3	6657.3	**4.29**	6.12E-285
RALGAPA2	Ral GTPase activating protein catalytic alpha subunit 2 [Source:HGNC Symbol;Acc:HGNC:16207]	214.6	925.3	**4.26**	2.54E-197
CLMN	calmin [Source:HGNC Symbol;Acc:HGNC:19972]	50.3	229.3	**4.26**	1.64E-38
CALCR	calcitonin receptor [Source:HGNC Symbol;Acc:HGNC:1440]	1.2	11.6	**4.23**	2.55E-06
AOX1	aldehyde oxidase 1 [Source:HGNC Symbol;Acc:HGNC:553]	338.0	1680.5	**4.20**	7.01E-23
SLC14A2	solute carrier family 14 member 2 [Source:HGNC Symbol;Acc:HGNC:10919]	5.7	31.5	**4.17**	2.87E-09
OSBPL5	oxysterol binding protein like 5 [Source:HGNC Symbol;Acc:HGNC:16392]	724.2	3031.6	**4.14**	9.58E-282
CBLB	Cbl proto-oncogene B [Source:HGNC Symbol;Acc:HGNC:1542]	572.1	2387.0	**4.14**	5.95E-234
HS3ST3B1	heparan sulfate-glucosamine 3-sulfotransferase 3B1 [Source:HGNC Symbol;Acc:HGNC:5198]	256.5	1060.8	**4.14**	2.07E-119
RHOB	ras homolog family member B [Source:HGNC Symbol;Acc:HGNC:668]	944.8	3864.3	**4.08**	1.55E-174
NNMT	nicotinamide N-methyltransferase [Source:HGNC Symbol;Acc:HGNC:7861]	2708.9	10905.2	**4.08**	4.32E-134
TLN2	talin 2 [Source:HGNC Symbol;Acc:HGNC:15447]	333.6	1417.5	**4.08**	4.40E-86
CYP4F22	cytochrome P450 family 4 subfamily F member 22 [Source:HGNC Symbol;Acc:HGNC:26820]	6.5	32.8	**4.08**	1.12E-12
RIPOR2	RHO family interacting cell polarization regulator 2 [Source:HGNC Symbol;Acc:HGNC:13872]	3.9	44.1	**4.08**	9.54E-06
ITGA5	integrin subunit alpha 5 [Source:HGNC Symbol;Acc:HGNC:6141]	5908.8	23991.7	**4.06**	2.04E-269
AOC2	amine oxidase, copper containing 2 [Source:HGNC Symbol;Acc:HGNC:549]	769.4	3114.5	**4.06**	1.21E-200
TRPM3	transient receptor potential cation channel subfamily M member 3 [Source:HGNC Symbol;Acc:HGNC:17	10.3	48.8	**4.06**	9.98E-12
NUDT4	nudix hydrolase 4 [Source:HGNC Symbol;Acc:HGNC:8051]	1439.2	5991.3	**4.03**	1.49E-145
ARL4D	ADP ribosylation factor like GTPase 4D [Source:HGNC Symbol;Acc:HGNC:656]	120.9	508.1	**4.03**	5.78E-83
TRABD2B	TraB domain containing 2B [Source:HGNC Symbol;Acc:HGNC:44200]	13.8	61.7	**4.03**	1.07E-16
RASD1	ras related dexamethasone induced 1 [Source:HGNC Symbol;Acc:HGNC:15828]	149.9	605.4	**4.00**	2.49E-76
FBLN5	fibulin 5 [Source:HGNC Symbol;Acc:HGNC:3602]	35.0	150.3	**4.00**	2.39E-29
RHOXF1-AS1	RHOXF1 antisense RNA 1 [Source:HGNC Symbol;Acc:HGNC:51582]	20.6	93.7	**4.00**	2.08E-19
GCNT3	glucosaminyl (N-acetyl) transferase 3, mucin type [Source:HGNC Symbol;Acc:HGNC:4205]	35.2	154.6	**3.97**	8.45E-31
PTX3	pentraxin 3 [Source:HGNC Symbol;Acc:HGNC:9692]	190.7	874.0	**3.97**	2.84E-28
IRF4	interferon regulatory factor 4 [Source:HGNC Symbol;Acc:HGNC:6119]	27.6	128.5	**3.97**	6.64E-27
ERLNC1	estrogen receptor responsive lncRNA 1 [Source:HGNC Symbol;Acc:HGNC:41109]	6.4	31.6	**3.97**	4.01E-11
PLPP3	phospholipid phosphatase 3 [Source:HGNC Symbol;Acc:HGNC:9229]	1079.4	4280.4	**3.94**	6.01E-205
CD1D	CD1d molecule [Source:HGNC Symbol;Acc:HGNC:1637]	58.5	236.5	**3.94**	8.63E-55
GGT5	gamma-glutamyltransferase 5 [Source:HGNC Symbol;Acc:HGNC:4260]	3.9	23.0	**3.94**	5.65E-07
NEXN	nexilin F-actin binding protein [Source:HGNC Symbol;Acc:HGNC:29557]	202.9	799.4	**3.92**	5.28E-170
OFD1	OFD1, centriole and centriolar satellite protein [Source:HGNC Symbol;Acc:HGNC:2567]	804.7	3176.6	**3.92**	1.55E-151
DCLK1	doublecortin like kinase 1 [Source:HGNC Symbol;Acc:HGNC:2700]	11.0	75.6	**3.86**	3.18E-06
MARCH3	membrane associated ring-CH-type finger 3 [Source:HGNC Symbol;Acc:HGNC:28728]	38.3	158.2	**3.84**	6.74E-34
IL1R1	interleukin 1 receptor type 1 [Source:HGNC Symbol;Acc:HGNC:5993]	3690.2	14009.3	**3.81**	0

ASB13	ankyrin repeat and SOCS box containing 13 [Source:HGNC Symbol;Acc:HGNC:19765]	108.0	424.5	**3.81**	8.47E-59
DNAH5	dynein axonemal heavy chain 5 [Source:HGNC Symbol;Acc:HGNC:2950]	116.8	459.4	**3.81**	3.16E-52
LINC00261	long intergenic non-protein coding RNA 261 [Source:HGNC Symbol;Acc:HGNC:16189]	7.9	37.8	**3.81**	3.47E-10
ABLIM2	actin binding LIM protein family member 2 [Source:HGNC Symbol;Acc:HGNC:19195]	3.0	39.0	**3.81**	3.76E-05
FAXDC2	fatty acid hydroxylase domain containing 2 [Source:HGNC Symbol;Acc:HGNC:1334]	198.8	828.4	**3.73**	7.87E-51
NET1	neuroepithelial cell transforming 1 [Source:HGNC Symbol;Acc:HGNC:14592]	1674.5	6441.3	**3.73**	1.78E-48
PRKAG2-AS1	PRKAG2 antisense RNA 1 [Source:HGNC Symbol;Acc:HGNC:40468]	4.0	20.8	**3.73**	1.37E-07
TSPAN7	tetraspanin 7 [Source:HGNC Symbol;Acc:HGNC:11854]	1.9	11.4	**3.73**	2.63E-06
GLULP4	glutamate-ammonia ligase pseudogene 4 [Source:HGNC Symbol;Acc:HGNC:4345]	1.6	11.6	**3.73**	1.78E-05
MT2P1	metallothionein 2 pseudogene 1 [Source:HGNC Symbol;Acc:HGNC:7407]	1.2	9.9	**3.73**	2.49E-05
LINC00607	long intergenic non-protein coding RNA 607 [Source:HGNC Symbol;Acc:HGNC:43944]	108.0	403.8	**3.68**	8.67E-46
SYNE2	spectrin repeat containing nuclear envelope protein 2 [Source:HGNC Symbol;Acc:HGNC:17084]	944.2	3606.7	**3.68**	6.59E-34
PHLDB2	pleckstrin homology like domain family B member 2 [Source:HGNC Symbol;Acc:HGNC:29573]	64.6	244.6	**3.68**	2.38E-33
ADH1B	alcohol dehydrogenase 1B (class I), beta polypeptide [Source:HGNC Symbol;Acc:HGNC:250]	1.8	11.7	**3.68**	1.03E-05
CLIC6	chloride intracellular channel 6 [Source:HGNC Symbol;Acc:HGNC:2065]	197.9	731.4	**3.63**	3.80E-74
SYN2	synapsin II [Source:HGNC Symbol;Acc:HGNC:11495]	80.4	300.3	**3.63**	7.56E-55
S1PR3	sphingosine-1-phosphate receptor 3 [Source:HGNC Symbol;Acc:HGNC:3167]	110.4	444.6	**3.63**	1.21E-16
SMPX	small muscle protein X-linked [Source:HGNC Symbol;Acc:HGNC:11122]	1.3	9.8	**3.63**	2.35E-05
ANGPTL7	angiopoietin like 7 [Source:HGNC Symbol;Acc:HGNC:24078]	441.4	1708.1	**3.61**	5.37E-27
PRLR	prolactin receptor [Source:HGNC Symbol;Acc:HGNC:9446]	8.1	37.5	**3.61**	9.75E-09
ABCC8	ATP binding cassette subfamily C member 8 [Source:HGNC Symbol;Acc:HGNC:59]	5.9	29.8	**3.61**	1.49E-06
MT1E	metallothionein 1E [Source:HGNC Symbol;Acc:HGNC:7397]	3989.6	14349.2	**3.58**	1.13E-159
B3GNT5	UDP-GlcNAc:betaGal beta-1,3-N-acetylglucosaminyltransferase 5 [Source:HGNC Symbol;Acc:HGNC:156	426.7	1585.7	**3.58**	1.21E-67
LINC00482	long intergenic non-protein coding RNA 482 [Source:HGNC Symbol;Acc:HGNC:26816]	3.9	18.3	**3.58**	2.19E-07
TNC	tenascin C [Source:HGNC Symbol;Acc:HGNC:5318]	34118.7	123319.9	**3.53**	3.59E-49
AKAP12	A-kinase anchoring protein 12 [Source:HGNC Symbol;Acc:HGNC:370]	2361.4	9469.6	**3.53**	1.19E-18
MT1JP	metallothionein 1J, pseudogene [Source:HGNC Symbol;Acc:HGNC:7402]	6.0	27.2	**3.53**	1.90E-07
PSME4	proteasome activator subunit 4 [Source:HGNC Symbol;Acc:HGNC:20635]	1835.8	6495.7	**3.48**	9.53E-160
PER1	period circadian regulator 1 [Source:HGNC Symbol;Acc:HGNC:8845]	106.0	374.5	**3.48**	5.81E-64
ELMO1	engulfment and cell motility 1 [Source:HGNC Symbol;Acc:HGNC:16286]	465.1	1631.9	**3.46**	1.21E-91
ADAMTS7P4	ADAMTS7 pseudogene 4 [Source:HGNC Symbol;Acc:HGNC:49410]	1.8	11.4	**3.46**	4.02E-05
ZFP36	ZFP36 ring finger protein [Source:HGNC Symbol;Acc:HGNC:12862]	146.1	515.3	**3.43**	2.84E-44
LRRN3	leucine rich repeat neuronal 3 [Source:HGNC Symbol;Acc:HGNC:17200]	29.9	109.1	**3.43**	2.19E-24
ADAMTS9-AS1	ADAMTS9 antisense RNA 1 [Source:HGNC Symbol;Acc:HGNC:40625]	32.1	128.3	**3.41**	2.39E-15
OFD1P17	OFD1 pseudogene 17 [Source:HGNC Symbol;Acc:HGNC:1332]	12.0	45.2	**3.41**	3.23E-13
LINC02407	long intergenic non-protein coding RNA 2407 [Source:HGNC Symbol;Acc:HGNC:53336]	6.5	25.8	**3.41**	1.80E-08
JADE1	jade family PHD finger 1 [Source:HGNC Symbol;Acc:HGNC:30027]	762.7	2588.4	**3.34**	8.12E-129
USP53	ubiquitin specific peptidase 53 [Source:HGNC Symbol;Acc:HGNC:29255]	4039.8	13494.0	**3.32**	1.56E-141
RNF157	ring finger protein 157 [Source:HGNC Symbol;Acc:HGNC:29402]	13.9	51.7	**3.32**	2.38E-12
TGFBR2	transforming growth factor beta receptor 2 [Source:HGNC Symbol;Acc:HGNC:11773]	4791.0	15889.1	**3.29**	9.67E-238
GLRX	glutaredoxin [Source:HGNC Symbol;Acc:HGNC:4330]	3078.4	10282.6	**3.29**	5.62E-137
PRKAG2	protein kinase AMP-activated non-catalytic subunit gamma 2 [Source:HGNC Symbol;Acc:HGNC:9386]	470.8	1591.1	**3.29**	4.77E-87
NAV2	neuron navigator 2 [Source:HGNC Symbol;Acc:HGNC:15997]	520.3	1782.0	**3.29**	2.21E-35
ALDH1L1	aldehyde dehydrogenase 1 family member L1 [Source:HGNC Symbol;Acc:HGNC:3978]	27.1	94.3	**3.29**	1.38E-23
NAV2-AS2	NAV2 antisense RNA 2 [Source:HGNC Symbol;Acc:HGNC:40743]	19.9	71.1	**3.29**	6.18E-17
RGCC	regulator of cell cycle [Source:HGNC Symbol;Acc:HGNC:20369]	3660.3	12302.0	**3.27**	6.38E-98
DSC3	desmocollin 3 [Source:HGNC Symbol;Acc:HGNC:3037]	1000.2	3244.1	**3.25**	2.86E-135
MTSS1	MTSS1, I-BAR domain containing [Source:HGNC Symbol;Acc:HGNC:20443]	61.2	200.0	**3.25**	4.70E-21
AZGP1	alpha-2-glycoprotein 1, zinc-binding [Source:HGNC Symbol;Acc:HGNC:910]	1.5	11.0	**3.25**	0.0002511
DPP4	dipeptidyl peptidase 4 [Source:HGNC Symbol;Acc:HGNC:3009]	1424.9	4619.6	**3.23**	4.34E-125
C5AR2	complement component 5a receptor 2 [Source:HGNC Symbol;Acc:HGNC:4527]	502.6	1662.9	**3.23**	1.31E-82
ANGPTL4	angiopoietin like 4 [Source:HGNC Symbol;Acc:HGNC:16039]	251.4	833.6	**3.23**	2.39E-57
FOS	Fos proto-oncogene, AP-1 transcription factor subunit [Source:HGNC Symbol;Acc:HGNC:3796]	238.7	793.5	**3.23**	3.98E-39
ITPKC	inositol-trisphosphate 3-kinase C [Source:HGNC Symbol;Acc:HGNC:14897]	323.2	1037.6	**3.20**	2.35E-133
PHC2	polyhomeotic homolog 2 [Source:HGNC Symbol;Acc:HGNC:3183]	918.3	2977.5	**3.20**	7.58E-98
KDR	kinase insert domain receptor [Source:HGNC Symbol;Acc:HGNC:6307]	3.3	16.4	**3.20**	4.90E-05
SMIM3	small integral membrane protein 3 [Source:HGNC Symbol;Acc:HGNC:30248]	181.3	593.7	**3.18**	1.33E-50
PRUNE2	prune homolog 2 [Source:HGNC Symbol;Acc:HGNC:25209]	2911.2	9795.5	**3.18**	8.88E-18
PLEKHA7	pleckstrin homology domain containing A7 [Source:HGNC Symbol;Acc:HGNC:27049]	77.7	296.6	**3.18**	1.54E-09
CD163	CD163 molecule [Source:HGNC Symbol;Acc:HGNC:1631]	3.4	17.8	**3.18**	0.0001712
TTPAL	alpha tocopherol transfer protein like [Source:HGNC Symbol;Acc:HGNC:16114]	759.1	2416.9	**3.16**	5.65E-132
ANKRD1	ankyrin repeat domain 1 [Source:HGNC Symbol;Acc:HGNC:15819]	6.1	26.7	**3.16**	1.91E-05
PARM1	prostate androgen-regulated mucin-like protein 1 [Source:HGNC Symbol;Acc:HGNC:24536]	799.2	2580.1	**3.14**	8.78E-100
ADAMTS9	ADAM metallopeptidase with thrombospondin type 1 motif 9 [Source:HGNC Symbol;Acc:HGNC:13202]	484.4	1713.8	**3.14**	8.52E-17
PPARG	peroxisome proliferator activated receptor gamma [Source:HGNC Symbol;Acc:HGNC:9236]	24.5	79.5	**3.12**	6.73E-22
CACHD1	cache domain containing 1 [Source:HGNC Symbol;Acc:HGNC:29314]	525.2	1635.1	**3.10**	9.45E-163
ZFP36L2	ZFP36 ring finger protein like 2 [Source:HGNC Symbol;Acc:HGNC:1108]	834.4	2626.1	**3.10**	1.60E-112
SESN3	sestrin 3 [Source:HGNC Symbol;Acc:HGNC:23060]	4701.2	14511.0	**3.10**	1.08E-95
MCF2L2	MCF.2 cell line derived transforming sequence-like 2 [Source:HGNC Symbol;Acc:HGNC:30319]	213.6	678.2	**3.10**	3.40E-47
PTPRG	protein tyrosine phosphatase, receptor type G [Source:HGNC Symbol;Acc:HGNC:9671]	1881.4	5800.2	**3.07**	0
PTPRG-AS1	PTPRG antisense RNA 1 [Source:HGNC Symbol;Acc:HGNC:44638]	264.0	817.6	**3.07**	1.49E-120
IGFBP2	insulin like growth factor binding protein 2 [Source:HGNC Symbol;Acc:HGNC:5471]	108.8	343.3	**3.07**	9.50E-51

ELMO1-AS1	ELMO1 antisense RNA 1 [Source:HGNC Symbol;Acc:HGNC:40765]	43.4	139.2	**3.07**	2.02E-25
MT1L	metallothionein 1L, pseudogene [Source:HGNC Symbol;Acc:HGNC:7404]	486.2	1457.5	**3.05**	3.69E-68
TMEM204	transmembrane protein 204 [Source:HGNC Symbol;Acc:HGNC:14158]	157.7	480.5	**3.03**	3.39E-56
SLC22A3	solute carrier family 22 member 3 [Source:HGNC Symbol;Acc:HGNC:10967]	60.5	188.6	**3.03**	1.96E-31
GABARAPL1	GABA type A receptor associated protein like 1 [Source:HGNC Symbol;Acc:HGNC:4068]	705.5	2304.4	**3.03**	1.30E-28
NAV2-AS1	NAV2 antisense RNA 1 [Source:HGNC Symbol;Acc:HGNC:40744]	28.5	94.8	**3.03**	1.10E-12
PCA3	prostate cancer associated 3 (non-protein coding) [Source:HGNC Symbol;Acc:HGNC:8637]	13.9	47.3	**3.03**	1.34E-10
SESN1	sestrin 1 [Source:HGNC Symbol;Acc:HGNC:21595]	457.5	1408.0	**3.01**	3.56E-105
AOC3	amine oxidase, copper containing 3 [Source:HGNC Symbol;Acc:HGNC:550]	141.4	429.3	**2.99**	8.49E-48
AFAP1L1	actin filament associated protein 1 like 1 [Source:HGNC Symbol;Acc:HGNC:26714]	13.9	46.3	**2.99**	1.16E-09
MTUS2	microtubule associated scaffold protein 2 [Source:HGNC Symbol;Acc:HGNC:20595]	18.0	62.1	**2.99**	1.85E-09
NEXN-AS1	NEXN antisense RNA 1 [Source:HGNC Symbol;Acc:HGNC:31983]	12.8	42.3	**2.99**	2.08E-08
ADGRG1	adhesion G protein-coupled receptor G1 [Source:HGNC Symbol;Acc:HGNC:4512]	57.0	193.0	**2.99**	1.32E-07
MERTK	MER proto-oncogene, tyrosine kinase [Source:HGNC Symbol;Acc:HGNC:7027]	94.7	289.2	**2.97**	2.52E-32
KCNS3	potassium voltage-gated channel modifier subfamily S member 3 [Source:HGNC Symbol;Acc:HGNC:630	19.0	58.8	**2.97**	1.93E-14
HS6ST3	heparan sulfate 6-O-sulfotransferase 3 [Source:HGNC Symbol;Acc:HGNC:19134]	12.7	46.2	**2.97**	1.40E-06
IL16	interleukin 16 [Source:HGNC Symbol;Acc:HGNC:5980]	573.4	1724.3	**2.95**	9.41E-72
PTPRJ	protein tyrosine phosphatase, receptor type J [Source:HGNC Symbol;Acc:HGNC:9673]	435.9	1293.9	**2.93**	2.75E-72
FABP4	fatty acid binding protein 4 [Source:HGNC Symbol;Acc:HGNC:3559]	124.8	374.2	**2.93**	1.11E-23
PRRG4	proline rich and Gla domain 4 [Source:HGNC Symbol;Acc:HGNC:30799]	18.8	59.2	**2.93**	3.87E-12
SPSB1	splA/ryanodine receptor domain and SOCS box containing 1 [Source:HGNC Symbol;Acc:HGNC:30628]	444.7	1309.7	**2.91**	3.60E-58
HSD17B11	hydroxysteroid 17-beta dehydrogenase 11 [Source:HGNC Symbol;Acc:HGNC:22960]	70.8	215.4	**2.91**	3.72E-29
TBC1D8	TBC1 domain family member 8 [Source:HGNC Symbol;Acc:HGNC:17791]	82.7	251.7	**2.91**	5.59E-27
AQP7P2	aquaporin 7 pseudogene 2 [Source:HGNC Symbol;Acc:HGNC:32049]	11.2	40.2	**2.91**	4.98E-07
RPS6KA2	ribosomal protein S6 kinase A2 [Source:HGNC Symbol;Acc:HGNC:10431]	461.5	1361.1	**2.89**	1.40E-82
MMP19	matrix metallopeptidase 19 [Source:HGNC Symbol;Acc:HGNC:7165]	9.7	31.3	**2.89**	4.96E-08
CCNA1	cyclin A1 [Source:HGNC Symbol;Acc:HGNC:1577]	6.5	24.9	**2.89**	6.65E-05
IP6K3	inositol hexakisphosphate kinase 3 [Source:HGNC Symbol;Acc:HGNC:17269]	6.2	25.2	**2.89**	0.0001009
ALDH6A1	aldehyde dehydrogenase 6 family member A1 [Source:HGNC Symbol;Acc:HGNC:7179]	696.4	2027.3	**2.87**	1.56E-141
SQOR	sulfide quinone oxidoreductase [Source:HGNC Symbol;Acc:HGNC:20390]	450.8	1315.6	**2.87**	9.36E-67
DAW1	dynein assembly factor with WD repeats 1 [Source:HGNC Symbol;Acc:HGNC:26383]	28.4	84.7	**2.87**	2.11E-16
GPX3	glutathione peroxidase 3 [Source:HGNC Symbol;Acc:HGNC:4555]	3311.8	11502.0	**2.87**	2.71E-09
OR7E14P	olfactory receptor family 7 subfamily E member 14 pseudogene [Source:HGNC Symbol;Acc:HGNC:8385]	5.4	19.6	**2.85**	2.57E-05
PACSIN2	protein kinase C and casein kinase substrate in neurons 2 [Source:HGNC Symbol;Acc:HGNC:8571]	2885.5	8237.9	**2.81**	1.79E-122
SLC26A6	solute carrier family 26 member 6 [Source:HGNC Symbol;Acc:HGNC:14472]	251.1	706.5	**2.81**	1.12E-96
ARRDC2	arrestin domain containing 2 [Source:HGNC Symbol;Acc:HGNC:25225]	328.0	933.3	**2.81**	3.01E-83
HS3ST3A1	heparan sulfate-glucosamine 3-sulfotransferase 3A1 [Source:HGNC Symbol;Acc:HGNC:5196]	78.3	226.1	**2.81**	8.98E-26
EVA1C	eva-1 homolog C [Source:HGNC Symbol;Acc:HGNC:13239]	36.4	107.2	**2.81**	8.27E-15
ZNF259P1	zinc finger protein 259 pseudogene 1 [Source:HGNC Symbol;Acc:HGNC:13052]	2.9	12.4	**2.81**	0.0005281
ELK3	ELK3, ETS transcription factor [Source:HGNC Symbol;Acc:HGNC:3325]	1171.5	3306.9	**2.79**	6.61E-123
APPL2	adaptor protein, phosphotyrosine interacting with PH domain and leucine zipper 2 [Source:HGNC Symb	1387.2	3931.9	**2.79**	3.80E-106
SERPING1	serpin family G member 1 [Source:HGNC Symbol;Acc:HGNC:1228]	329.2	900.4	**2.79**	1.10E-46
DUSP5	dual specificity phosphatase 5 [Source:HGNC Symbol;Acc:HGNC:3071]	115.6	329.9	**2.79**	1.28E-20
S100P	S100 calcium binding protein P [Source:HGNC Symbol;Acc:HGNC:10504]	13.8	43.3	**2.79**	5.00E-08
ABAT	4-aminobutyrate aminotransferase [Source:HGNC Symbol;Acc:HGNC:23]	183.9	520.9	**2.77**	1.32E-63
SOD2	superoxide dismutase 2 [Source:HGNC Symbol;Acc:HGNC:11180]	40215.6	112308.8	**2.77**	2.85E-38
POU3F3	POU class 3 homeobox 3 [Source:HGNC Symbol;Acc:HGNC:9216]	33.1	95.9	**2.77**	2.54E-18
FGD4	FYVE, RhoGEF and PH domain containing 4 [Source:HGNC Symbol;Acc:HGNC:19125]	2332.6	6460.8	**2.75**	7.49E-216
ANKS1B	ankyrin repeat and sterile alpha motif domain containing 1B [Source:HGNC Symbol;Acc:HGNC:24600]	236.6	653.8	**2.75**	5.96E-95
LONRF1	LON peptidase N-terminal domain and ring finger 1 [Source:HGNC Symbol;Acc:HGNC:26302]	438.0	1241.6	**2.75**	3.68E-63
FGL2	fibrinogen like 2 [Source:HGNC Symbol;Acc:HGNC:3696]	22.9	67.5	**2.75**	1.26E-12
LINC00707	long intergenic non-protein coding RNA 707 [Source:HGNC Symbol;Acc:HGNC:44691]	15.7	46.8	**2.75**	4.82E-07
AGTR1	angiotensin II receptor type 1 [Source:HGNC Symbol;Acc:HGNC:336]	6.9	23.0	**2.75**	4.14E-05
LINC01158	long intergenic non-protein coding RNA 1158 [Source:HGNC Symbol;Acc:HGNC:49513]	2.4	11.1	**2.75**	0.001232
CHAC2	ChaC cation transport regulator homolog 2 [Source:HGNC Symbol;Acc:HGNC:32363]	236.0	660.9	**2.73**	2.11E-74
SLC44A2	solute carrier family 44 member 2 [Source:HGNC Symbol;Acc:HGNC:17292]	2135.3	5851.3	**2.73**	2.86E-71
PMEPA1	prostate transmembrane protein, androgen induced 1 [Source:HGNC Symbol;Acc:HGNC:14107]	1153.3	3315.3	**2.73**	7.52E-21
ABL2	ABL proto-oncogene 2, non-receptor tyrosine kinase [Source:HGNC Symbol;Acc:HGNC:77]	1679.9	4553.6	**2.71**	2.61E-140
STON1	stonin 1 [Source:HGNC Symbol;Acc:HGNC:17003]	553.5	1500.3	**2.71**	3.18E-77
GGTA1P	glycoprotein, alpha-galactosyltransferase 1 pseudogene [Source:HGNC Symbol;Acc:HGNC:4253]	16.9	49.6	**2.71**	4.64E-11
SLC7A8	solute carrier family 7 member 8 [Source:HGNC Symbol;Acc:HGNC:11066]	242.2	736.4	**2.71**	4.59E-09
ORM1	orosomucoid 1 [Source:HGNC Symbol;Acc:HGNC:8498]	35.5	108.3	**2.71**	2.43E-07
NCKAP1L	NCK associated protein 1 like [Source:HGNC Symbol;Acc:HGNC:4862]	10.3	31.5	**2.71**	4.74E-06
ZNF474	zinc finger protein 474 [Source:HGNC Symbol;Acc:HGNC:23245]	6.5	20.8	**2.71**	1.00E-05
TRPC6	transient receptor potential cation channel subfamily C member 6 [Source:HGNC Symbol;Acc:HGNC:12	9.6	29.3	**2.71**	1.62E-05
LNX2	ligand of numb-protein X 2 [Source:HGNC Symbol;Acc:HGNC:20421]	389.8	1061.3	**2.69**	2.03E-111
ARHGEF26	Rho guanine nucleotide exchange factor 26 [Source:HGNC Symbol;Acc:HGNC:24490]	113.0	312.5	**2.69**	4.26E-36
DDIT4	DNA damage inducible transcript 4 [Source:HGNC Symbol;Acc:HGNC:24944]	1542.9	4090.3	**2.69**	1.79E-17
LINC02542	long intergenic non-protein coding RNA 2542 [Source:HGNC Symbol;Acc:HGNC:53576]	11.5	34.7	**2.69**	1.65E-07
HIP1	huntingtin interacting protein 1 [Source:HGNC Symbol;Acc:HGNC:4913]	1135.3	3067.6	**2.68**	1.13E-61
HIPK2	homeodomain interacting protein kinase 2 [Source:HGNC Symbol;Acc:HGNC:14402]	987.1	2753.1	**2.68**	3.42E-28
DDIT4-AS1	DDIT4 antisense RNA 1 [Source:HGNC Symbol;Acc:HGNC:52379]	1182.2	3088.7	**2.66**	4.87E-17

FAM196A	family with sequence similarity 196 member A [Source:HGNC Symbol;Acc:HGNC:33859]	13.7	41.2	**2.66**	1.74E-06
ENPP4	ectonucleotide pyrophosphatase/phosphodiesterase 4 [Source:HGNC Symbol;Acc:HGNC:3359]	289.1	759.0	**2.64**	9.21E-81
ITPRIP	inositol 1,4,5-trisphosphate receptor interacting protein [Source:HGNC Symbol;Acc:HGNC:29370]	224.5	619.4	**2.64**	1.18E-30
SLPI	secretory leukocyte peptidase inhibitor [Source:HGNC Symbol;Acc:HGNC:11092]	68.9	190.7	**2.64**	3.09E-19
ENPP5	ectonucleotide pyrophosphatase/phosphodiesterase 5 (putative) [Source:HGNC Symbol;Acc:HGNC:137	27.7	75.5	**2.64**	2.36E-13
MYPN	myopalladin [Source:HGNC Symbol;Acc:HGNC:23246]	18.9	54.8	**2.64**	1.18E-07
ALCAM	activated leukocyte cell adhesion molecule [Source:HGNC Symbol;Acc:HGNC:400]	335.6	889.7	**2.62**	1.24E-103
KBTBD12	kelch repeat and BTB domain containing 12 [Source:HGNC Symbol;Acc:HGNC:25731]	40.4	111.4	**2.62**	1.61E-13
BNC1	basonuclin 1 [Source:HGNC Symbol;Acc:HGNC:1081]	5.1	16.2	**2.62**	0.0001903
TPRG1-AS1	TPRG1 antisense RNA 1 [Source:HGNC Symbol;Acc:HGNC:42391]	2.1	9.1	**2.62**	0.002699
LINC00702	long intergenic non-protein coding RNA 702 [Source:HGNC Symbol;Acc:HGNC:44676]	456.0	1187.5	**2.60**	6.78E-125
CNKSR3	CNKSR family member 3 [Source:HGNC Symbol;Acc:HGNC:23034]	481.6	1263.7	**2.60**	1.35E-90
CNOT8	CCR4-NOT transcription complex subunit 8 [Source:HGNC Symbol;Acc:HGNC:9207]	1034.0	2810.5	**2.60**	7.96E-59
IL6R-AS1	IL6R antisense RNA 1 [Source:HGNC Symbol;Acc:HGNC:53716]	3.6	12.7	**2.60**	0.0008182
FSTL3	follistatin like 3 [Source:HGNC Symbol;Acc:HGNC:3973]	98.4	263.6	**2.58**	3.85E-32
ACADL	acyl-CoA dehydrogenase long chain [Source:HGNC Symbol;Acc:HGNC:88]	106.2	288.2	**2.58**	2.11E-27
DSG3	desmoglein 3 [Source:HGNC Symbol;Acc:HGNC:3050]	3.8	13.1	**2.58**	0.0005596
IRAK3	interleukin 1 receptor associated kinase 3 [Source:HGNC Symbol;Acc:HGNC:17020]	860.8	2221.1	**2.57**	3.23E-245
MCL1	MCL1, BCL2 family apoptosis regulator [Source:HGNC Symbol;Acc:HGNC:6943]	7685.5	19872.7	**2.57**	5.46E-171
EFHD1	EF-hand domain family member D1 [Source:HGNC Symbol;Acc:HGNC:29556]	310.8	810.6	**2.57**	2.28E-32
SEC14L2	SEC14 like lipid binding 2 [Source:HGNC Symbol;Acc:HGNC:10699]	220.5	584.7	**2.55**	1.04E-36
C1orf87	chromosome 1 open reading frame 87 [Source:HGNC Symbol;Acc:HGNC:28547]	4.3	14.8	**2.55**	0.001184
NFKBIA	NFKB inhibitor alpha [Source:HGNC Symbol;Acc:HGNC:7797]	443.3	1116.5	**2.53**	8.99E-92
TBC1D2B	TBC1 domain family member 2B [Source:HGNC Symbol;Acc:HGNC:29183]	640.9	1642.7	**2.53**	3.20E-88
TBX15	T-box 15 [Source:HGNC Symbol;Acc:HGNC:11594]	2680.6	6867.4	**2.53**	8.05E-88
TRIB1	tribbles pseudokinase 1 [Source:HGNC Symbol;Acc:HGNC:16891]	450.9	1157.0	**2.53**	2.93E-58
CDO1	cysteine dioxygenase type 1 [Source:HGNC Symbol;Acc:HGNC:1795]	2780.6	7851.3	**2.53**	2.09E-15
C11orf91	chromosome 11 open reading frame 91 [Source:HGNC Symbol;Acc:HGNC:34444]	15.5	42.1	**2.53**	2.09E-07
AKR1C2	aldo-keto reductase family 1 member C2 [Source:HGNC Symbol;Acc:HGNC:385]	1447.0	4716.6	**2.53**	4.77E-06
PRELP	proline and arginine rich end leucine rich repeat protein [Source:HGNC Symbol;Acc:HGNC:9357]	6139.4	15609.4	**2.51**	8.67E-65
GALNT13	polypeptide N-acetylgalactosaminyltransferase 13 [Source:HGNC Symbol;Acc:HGNC:23242]	107.4	271.1	**2.51**	2.64E-33
TXNIP	thioredoxin interacting protein [Source:HGNC Symbol;Acc:HGNC:16952]	1956.3	5076.0	**2.51**	7.17E-20
EDEM2	ER degradation enhancing alpha-mannosidase like protein 2 [Source:HGNC Symbol;Acc:HGNC:15877]	277.2	696.4	**2.50**	1.32E-65
SAT1	spermidine/spermine N1-acetyltransferase 1 [Source:HGNC Symbol;Acc:HGNC:10540]	6466.6	16105.5	**2.50**	4.36E-65
SYNPO2	synaptopodin 2 [Source:HGNC Symbol;Acc:HGNC:17732]	38.0	101.0	**2.50**	2.84E-10
AASS	aminoadipate-semialdehyde synthase [Source:HGNC Symbol;Acc:HGNC:17366]	411.1	1030.7	**2.48**	1.24E-96
UGP2	UDP-glucose pyrophosphorylase 2 [Source:HGNC Symbol;Acc:HGNC:12527]	9160.1	22898.7	**2.48**	2.49E-89
MT1A	metallothionein 1A [Source:HGNC Symbol;Acc:HGNC:7393]	50.6	129.5	**2.48**	1.36E-17
ADGRD1	adhesion G protein-coupled receptor D1 [Source:HGNC Symbol;Acc:HGNC:19893]	24.0	63.6	**2.48**	1.53E-07
RN7SKP16	RNA, 7SK small nuclear pseudogene 16 [Source:HGNC Symbol;Acc:HGNC:45740]	2.3	9.4	**2.48**	0.00507
ATOH8	atonal bHLH transcription factor 8 [Source:HGNC Symbol;Acc:HGNC:24126]	646.4	1655.3	**2.46**	6.89E-48
FAM43A	family with sequence similarity 43 member A [Source:HGNC Symbol;Acc:HGNC:26888]	88.0	223.3	**2.46**	1.83E-14
MYOZ2	myozenin 2 [Source:HGNC Symbol;Acc:HGNC:1330]	17.5	45.8	**2.46**	5.00E-08
SNX7	sorting nexin 7 [Source:HGNC Symbol;Acc:HGNC:14971]	625.7	1525.9	**2.45**	1.03E-65
RGS17	regulator of G protein signaling 17 [Source:HGNC Symbol;Acc:HGNC:14088]	115.2	291.6	**2.45**	6.69E-27
DIAPH2	diaphanous related formin 2 [Source:HGNC Symbol;Acc:HGNC:2877]	505.1	1228.9	**2.43**	5.55E-91
ARHGAP12	Rho GTPase activating protein 12 [Source:HGNC Symbol;Acc:HGNC:16348]	1790.9	4469.4	**2.43**	2.49E-86
MYO1E	myosin IE [Source:HGNC Symbol;Acc:HGNC:7599]	1894.1	4670.8	**2.43**	3.42E-81
ACAN	aggrecan [Source:HGNC Symbol;Acc:HGNC:319]	81094.4	202992.8	**2.43**	6.72E-20
PCDH9-AS1	PCDH9 antisense RNA 1 [Source:HGNC Symbol;Acc:HGNC:39897]	3.5	12.3	**2.43**	0.004289
KIF21A	kinesin family member 21A [Source:HGNC Symbol;Acc:HGNC:19349]	625.8	1519.0	**2.41**	3.81E-161
LDHAL6B	lactate dehydrogenase A like 6B [Source:HGNC Symbol;Acc:HGNC:21481]	6.6	19.6	**2.41**	0.0005075
RN7SL600P	RNA, 7SL, cytoplasmic 600, pseudogene [Source:HGNC Symbol;Acc:HGNC:46616]	4.7	13.9	**2.41**	0.0006751
IRS2	insulin receptor substrate 2 [Source:HGNC Symbol;Acc:HGNC:6126]	364.5	902.6	**2.39**	1.50E-34
DNER	delta/notch like EGF repeat containing [Source:HGNC Symbol;Acc:HGNC:24456]	550.9	1328.3	**2.39**	1.89E-13
P2RX5	purinergic receptor P2X 5 [Source:HGNC Symbol;Acc:HGNC:8536]	41.2	102.1	**2.39**	4.93E-09
NEBL-AS1	NEBL antisense RNA 1 [Source:HGNC Symbol;Acc:HGNC:44899]	14.4	37.3	**2.39**	4.30E-07
RGS6	regulator of G protein signaling 6 [Source:HGNC Symbol;Acc:HGNC:10002]	4.8	16.9	**2.39**	0.00323
PYGL	glycogen phosphorylase L [Source:HGNC Symbol;Acc:HGNC:9725]	1785.1	4258.4	**2.38**	3.88E-89
BIN1	bridging integrator 1 [Source:HGNC Symbol;Acc:HGNC:1052]	604.5	1469.6	**2.38**	1.30E-62
MAP7	microtubule associated protein 7 [Source:HGNC Symbol;Acc:HGNC:6869]	152.3	364.8	**2.38**	1.35E-45
DCXR	dicarbonyl and L-xylulose reductase [Source:HGNC Symbol;Acc:HGNC:18985]	79.0	196.7	**2.38**	3.24E-22
BACH2	BTB domain and CNC homolog 2 [Source:HGNC Symbol;Acc:HGNC:14078]	30.2	76.0	**2.38**	2.97E-09
KCNB1	potassium voltage-gated channel subfamily B member 1 [Source:HGNC Symbol;Acc:HGNC:6231]	21.0	54.5	**2.38**	3.07E-06
TIPARP	TCDD inducible poly(ADP-ribose) polymerase [Source:HGNC Symbol;Acc:HGNC:23696]	1448.9	3418.2	**2.36**	1.29E-179
XPR1	xenotropic and polytropic retrovirus receptor 1 [Source:HGNC Symbol;Acc:HGNC:12827]	858.4	2024.1	**2.36**	3.39E-178
PROCR	protein C receptor [Source:HGNC Symbol;Acc:HGNC:9452]	1668.0	3949.0	**2.36**	2.37E-97
MAP3K5	mitogen-activated protein kinase kinase kinase 5 [Source:HGNC Symbol;Acc:HGNC:6857]	1605.8	3820.8	**2.36**	6.31E-95
DSE	dermatan sulfate epimerase [Source:HGNC Symbol;Acc:HGNC:21144]	3692.7	8924.5	**2.36**	1.49E-67
ATP1A1-AS1	ATP1A1 antisense RNA 1 [Source:HGNC Symbol;Acc:HGNC:28262]	2513.8	6086.3	**2.36**	1.90E-65
PIM3	Pim-3 proto-oncogene, serine/threonine kinase [Source:HGNC Symbol;Acc:HGNC:19310]	536.0	1300.1	**2.36**	1.03E-50
PCDH9	protocadherin 9 [Source:HGNC Symbol;Acc:HGNC:8661]	1691.1	4112.7	**2.36**	2.51E-10

MTOR-AS1	MTOR antisense RNA 1 [Source:HGNC Symbol;Acc:HGNC:40242]	25.5	62.9	**2.36**	6.81E-10
PLCXD3	phosphatidylinositol specific phospholipase C X domain containing 3 [Source:HGNC Symbol;Acc:HGNC:3	43.0	104.0	**2.36**	1.43E-08
PTPRM	protein tyrosine phosphatase, receptor type M [Source:HGNC Symbol;Acc:HGNC:9675]	4860.3	11615.0	**2.35**	2.56E-91
ATP1A1	ATPase Na+/K+ transporting subunit alpha 1 [Source:HGNC Symbol;Acc:HGNC:799]	10516.0	25230.1	**2.35**	6.01E-64
STARD5	StAR related lipid transfer domain containing 5 [Source:HGNC Symbol;Acc:HGNC:18065]	221.7	530.0	**2.35**	2.52E-37
SPARCL1	SPARC like 1 [Source:HGNC Symbol;Acc:HGNC:11220]	22.8	74.4	**2.35**	3.75E-05
CLTB	clathrin light chain B [Source:HGNC Symbol;Acc:HGNC:2091]	1213.0	2894.8	**2.33**	3.75E-71
UBALD2	UBA like domain containing 2 [Source:HGNC Symbol;Acc:HGNC:28438]	116.8	289.5	**2.33**	4.78E-15
MAN1A1	mannosidase alpha class 1A member 1 [Source:HGNC Symbol;Acc:HGNC:6821]	5141.5	12096.0	**2.31**	1.21E-133
TMEM100	transmembrane protein 100 [Source:HGNC Symbol;Acc:HGNC:25607]	119.6	288.0	**2.31**	4.02E-22
FOXO1	forkhead box O1 [Source:HGNC Symbol;Acc:HGNC:3819]	1074.5	2483.5	**2.30**	4.33E-151
ANK3	ankyrin 3 [Source:HGNC Symbol;Acc:HGNC:494]	3804.7	8718.8	**2.30**	7.45E-116
STON1-GTF2A1L	STON1-GTF2A1L readthrough [Source:HGNC Symbol;Acc:HGNC:30651]	344.2	775.6	**2.30**	1.79E-39
ITGA9-AS1	ITGA9 antisense RNA 1 [Source:HGNC Symbol;Acc:HGNC:49668]	141.4	334.9	**2.30**	3.01E-20
GIPC2	GIPC PDZ domain containing family member 2 [Source:HGNC Symbol;Acc:HGNC:18177]	26.6	64.0	**2.30**	2.83E-08
TEK	TEK receptor tyrosine kinase [Source:HGNC Symbol;Acc:HGNC:11724]	14.2	35.4	**2.30**	1.86E-05
SSBP2	single stranded DNA binding protein 2 [Source:HGNC Symbol;Acc:HGNC:15831]	481.2	1096.0	**2.28**	1.01E-46
MAP2K6	mitogen-activated protein kinase kinase 6 [Source:HGNC Symbol;Acc:HGNC:6846]	523.7	1276.9	**2.28**	1.31E-12
DIAPH2-AS1	DIAPH2 antisense RNA 1 [Source:HGNC Symbol;Acc:HGNC:16972]	11.5	28.5	**2.28**	6.91E-05
CD38	CD38 molecule [Source:HGNC Symbol;Acc:HGNC:1667]	8.9	23.8	**2.28**	0.0005296
CCND3	cyclin D3 [Source:HGNC Symbol;Acc:HGNC:1585]	406.4	923.4	**2.27**	1.54E-72
HHAT	hedgehog acyltransferase [Source:HGNC Symbol;Acc:HGNC:18270]	162.5	375.6	**2.27**	6.60E-37
STX11	syntaxin 11 [Source:HGNC Symbol;Acc:HGNC:11429]	37.9	87.5	**2.27**	5.72E-09
GRHL1	grainyhead like transcription factor 1 [Source:HGNC Symbol;Acc:HGNC:17923]	37.5	90.3	**2.27**	7.53E-08
EPSTI1	epithelial stromal interaction 1 [Source:HGNC Symbol;Acc:HGNC:16465]	22.6	56.4	**2.27**	8.82E-05
KCNJ2-AS1	KCNJ2 antisense RNA 1 [Source:HGNC Symbol;Acc:HGNC:43720]	2.9	9.5	**2.27**	0.008899
CTGF	connective tissue growth factor [Source:HGNC Symbol;Acc:HGNC:2500]	16003.3	36823.5	**2.25**	3.73E-20
ARHGAP20	Rho GTPase activating protein 20 [Source:HGNC Symbol;Acc:HGNC:18357]	151.4	352.1	**2.25**	8.59E-18
SH3RF3-AS1	SH3RF3 antisense RNA 1 [Source:HGNC Symbol;Acc:HGNC:44168]	15.6	38.5	**2.25**	0.000123
RNU2-28P	RNA, U2 small nuclear 28, pseudogene [Source:HGNC Symbol;Acc:HGNC:48521]	3.3	9.7	**2.25**	0.007103
CMPK1	cytidine/uridine monophosphate kinase 1 [Source:HGNC Symbol;Acc:HGNC:18170]	5405.0	12124.3	**2.23**	7.58E-198
ADGRG6	adhesion G protein-coupled receptor G6 [Source:HGNC Symbol;Acc:HGNC:13841]	3913.1	8727.7	**2.23**	4.45E-78
FOXD2-AS1	FOXD2 adjacent opposite strand RNA 1 [Source:HGNC Symbol;Acc:HGNC:44256]	11.6	28.5	**2.23**	0.0005953
CALHM5	calcium homeostasis modulator family member 5 [Source:HGNC Symbol;Acc:HGNC:21568]	4.9	13.9	**2.23**	0.01027
LAMA1	laminin subunit alpha 1 [Source:HGNC Symbol;Acc:HGNC:6481]	189.2	424.0	**2.22**	8.83E-37
RASSF8-AS1	RASSF8 antisense RNA 1 [Source:HGNC Symbol;Acc:HGNC:48637]	172.9	389.4	**2.22**	7.89E-36
PRKAR2B	protein kinase cAMP-dependent type II regulatory subunit beta [Source:HGNC Symbol;Acc:HGNC:9392]	309.8	710.5	**2.22**	8.85E-25
MYBPC1	myosin binding protein C, slow type [Source:HGNC Symbol;Acc:HGNC:7549]	3.7	11.7	**2.22**	0.01085
GRIA1	glutamate ionotropic receptor AMPA type subunit 1 [Source:HGNC Symbol;Acc:HGNC:4571]	2.3	8.0	**2.22**	0.01699
ITGA10	integrin subunit alpha 10 [Source:HGNC Symbol;Acc:HGNC:6135]	7022.8	15615.3	**2.20**	3.62E-138
BBS2	Bardet-Biedl syndrome 2 [Source:HGNC Symbol;Acc:HGNC:967]	1194.8	2680.8	**2.20**	8.27E-90
RNASE4	ribonuclease A family member 4 [Source:HGNC Symbol;Acc:HGNC:10047]	965.3	2180.5	**2.20**	6.21E-78
FURIN	furin, paired basic amino acid cleaving enzyme [Source:HGNC Symbol;Acc:HGNC:8568]	1446.3	3191.0	**2.20**	5.14E-47
RAPGEF1	Rap guanine nucleotide exchange factor 1 [Source:HGNC Symbol;Acc:HGNC:4568]	528.7	1199.6	**2.20**	4.74E-27
ADAMTSL4-AS1	ADAMTSL4 antisense RNA 1 [Source:HGNC Symbol;Acc:HGNC:32041]	25.8	59.5	**2.20**	1.58E-09
LAMA3	laminin subunit alpha 3 [Source:HGNC Symbol;Acc:HGNC:6483]	21.7	51.7	**2.20**	2.32E-07
MCTP1	multiple C2 and transmembrane domain containing 1 [Source:HGNC Symbol;Acc:HGNC:26183]	9.0	22.2	**2.20**	0.0008371
ODF3L1	outer dense fiber of sperm tails 3 like 1 [Source:HGNC Symbol;Acc:HGNC:28735]	6.0	15.4	**2.20**	0.001454
AL845321.1	monofunctional C1-tetrahydrofolate synthase, mitochondrial-like [Source:NCBI gene;Acc:100996643]	6.1	18.0	**2.20**	0.009421
MAB21L3	mab-21 like 3 [Source:HGNC Symbol;Acc:HGNC:26787]	2.6	8.6	**2.20**	0.01997
STC2	stanniocalcin 2 [Source:HGNC Symbol;Acc:HGNC:11374]	1899.6	4253.4	**2.19**	5.74E-19
LRRK2	leucine rich repeat kinase 2 [Source:HGNC Symbol;Acc:HGNC:18618]	104.8	235.1	**2.19**	5.78E-18
TMEM198B	transmembrane protein 198B (pseudogene) [Source:HGNC Symbol;Acc:HGNC:43629]	22.3	51.3	**2.19**	1.45E-06
FMO2	flavin containing monooxygenase 2 [Source:HGNC Symbol;Acc:HGNC:3770]	4.0	11.9	**2.19**	0.01173
WDR19	WD repeat domain 19 [Source:HGNC Symbol;Acc:HGNC:18340]	671.7	1492.2	**2.17**	5.39E-52
LOX	lysyl oxidase [Source:HGNC Symbol;Acc:HGNC:6664]	1771.3	3928.3	**2.17**	8.55E-15
MTUS2-AS1	MTUS2 antisense RNA 1 [Source:HGNC Symbol;Acc:HGNC:40924]	4.4	12.2	**2.17**	0.01014
ERGIC1	endoplasmic reticulum-golgi intermediate compartment 1 [Source:HGNC Symbol;Acc:HGNC:29205]	3068.8	6606.1	**2.16**	1.01E-219
IGF2BP2	insulin like growth factor 2 mRNA binding protein 2 [Source:HGNC Symbol;Acc:HGNC:28867]	333.3	740.5	**2.16**	1.04E-44
ADAMTSL4	ADAMTS like 4 [Source:HGNC Symbol;Acc:HGNC:19706]	41.2	92.9	**2.16**	1.19E-06
TSC22D4	TSC22 domain family member 4 [Source:HGNC Symbol;Acc:HGNC:21696]	152.4	328.2	**2.14**	4.84E-27
PROSER2	proline and serine rich 2 [Source:HGNC Symbol;Acc:HGNC:23728]	115.8	253.9	**2.14**	1.58E-14
TMEM44	transmembrane protein 44 [Source:HGNC Symbol;Acc:HGNC:25120]	75.9	170.1	**2.14**	7.88E-11
CDSN	corneodesmosin [Source:HGNC Symbol;Acc:HGNC:1802]	29.8	68.2	**2.14**	4.78E-07
NPB	neuropeptide B [Source:HGNC Symbol;Acc:HGNC:30099]	11.8	27.4	**2.14**	0.0002103
WDR93	WD repeat domain 93 [Source:HGNC Symbol;Acc:HGNC:26924]	3.1	8.6	**2.14**	0.01533
ANG	angiogenin [Source:HGNC Symbol;Acc:HGNC:483]	199.1	426.8	**2.13**	3.15E-40
SYBU	syntabulin [Source:HGNC Symbol;Acc:HGNC:26011]	560.7	1179.2	**2.13**	1.31E-28
CNTNAP3B	contactin associated protein like 3B [Source:HGNC Symbol;Acc:HGNC:32035]	50.5	110.2	**2.13**	3.31E-08
FRMD3	FERM domain containing 3 [Source:HGNC Symbol;Acc:HGNC:24125]	27.2	61.1	**2.13**	3.33E-08
PRR16	proline rich 16 [Source:HGNC Symbol;Acc:HGNC:29654]	31.9	70.4	**2.13**	2.02E-06
YBX3P1	Y-box binding protein 3 pseudogene 1 [Source:HGNC Symbol;Acc:HGNC:2429]	31.4	69.6	**2.11**	1.59E-06

TBC1D3L	TBC1 domain family member 3L [Source:HGNC Symbol;Acc:HGNC:51246]	4.6	12.5	**2.11**	0.01192
NUP50-DT	NUP50 divergent transcript [Source:HGNC Symbol;Acc:HGNC:50502]	1799.8	2604.7	**2.10**	9.39E-14
EPB41L4B	erythrocyte membrane protein band 4.1 like 4B [Source:HGNC Symbol;Acc:HGNC:19818]	33.6	74.8	**2.10**	7.86E-08
IL1RL1	interleukin 1 receptor like 1 [Source:HGNC Symbol;Acc:HGNC:5998]	829.6	1775.7	**2.08**	2.35E-28
MKNK2	MAP kinase interacting serine/threonine kinase 2 [Source:HGNC Symbol;Acc:HGNC:7111]	354.7	736.8	**2.08**	2.81E-21
KLF6	Kruppel like factor 6 [Source:HGNC Symbol;Acc:HGNC:2235]	4752.2	10129.4	**2.08**	3.72E-16
PSORS1C1	psoriasis susceptibility 1 candidate 1 [Source:HGNC Symbol;Acc:HGNC:17202]	55.7	121.0	**2.08**	2.72E-08
RAB9A	RAB9A, member RAS oncogene family [Source:HGNC Symbol;Acc:HGNC:9792]	747.1	1554.3	**2.07**	6.36E-104
SLC38A2	solute carrier family 38 member 2 [Source:HGNC Symbol;Acc:HGNC:13448]	17647.1	36259.3	**2.07**	3.42E-89
PLIN2	perilipin 2 [Source:HGNC Symbol;Acc:HGNC:248]	875.4	1834.3	**2.07**	2.58E-35
CFLAR-AS1	CFLAR antisense RNA 1 [Source:HGNC Symbol;Acc:HGNC:14437]	99.3	208.0	**2.07**	1.23E-20
NRBP2	nuclear receptor binding protein 2 [Source:HGNC Symbol;Acc:HGNC:19339]	156.7	325.7	**2.07**	3.86E-17
CP	ceruloplasmin [Source:HGNC Symbol;Acc:HGNC:2295]	3654.0	7486.3	**2.07**	1.11E-16
DDIT4L	DNA damage inducible transcript 4 like [Source:HGNC Symbol;Acc:HGNC:30555]	699.9	1444.0	**2.07**	1.79E-09
CDA	cytidine deaminase [Source:HGNC Symbol;Acc:HGNC:1712]	22.8	50.4	**2.07**	5.37E-06
DPPA2P4	developmental pluripotency associated 2 pseudogene 4 [Source:HGNC Symbol;Acc:HGNC:44629]	3.5	9.5	**2.07**	0.02136
TBC1D3C	TBC1 domain family member 3C [Source:HGNC Symbol;Acc:HGNC:24889]	3.1	8.9	**2.07**	0.02235
RETREG1	reticulophagy regulator 1 [Source:HGNC Symbol;Acc:HGNC:25964]	3447.1	7246.9	**2.06**	8.24E-50
MMP2	matrix metallopeptidase 2 [Source:HGNC Symbol;Acc:HGNC:7166]	165.6	347.7	**2.06**	1.16E-12
PARD6B	par-6 family cell polarity regulator beta [Source:HGNC Symbol;Acc:HGNC:16245]	37.0	81.1	**2.06**	7.58E-07
HMGN2P15	high mobility group nucleosomal binding domain 2 pseudogene 15 [Source:HGNC Symbol;Acc:HGNC:39	3.8	10.2	**2.06**	0.01986
LINC01564	long intergenic non-protein coding RNA 1564 [Source:HGNC Symbol;Acc:HGNC:51361]	4.2	12.4	**2.06**	0.03285
JAK1	Janus kinase 1 [Source:HGNC Symbol;Acc:HGNC:6190]	3647.6	7458.9	**2.04**	4.51E-88
MICAL1	microtubule associated monooxygenase, calponin and LIM domain containing 1 [Source:HGNC Symbol;	277.6	567.2	**2.04**	3.19E-33
DSEL	dermatan sulfate epimerase like [Source:HGNC Symbol;Acc:HGNC:18144]	762.7	1582.9	**2.04**	1.74E-29
CYP1B1-AS1	CYP1B1 antisense RNA 1 [Source:HGNC Symbol;Acc:HGNC:28543]	331.1	693.9	**2.04**	3.50E-17
BATF	basic leucine zipper ATF-like transcription factor [Source:HGNC Symbol;Acc:HGNC:958]	50.1	104.9	**2.04**	2.65E-09
CYP19A1	cytochrome P450 family 19 subfamily A member 1 [Source:HGNC Symbol;Acc:HGNC:2594]	360.4	699.3	**2.04**	1.97E-08
HMGB1P31	high mobility group box 1 pseudogene 31 [Source:HGNC Symbol;Acc:HGNC:39122]	22.0	46.8	**2.04**	2.08E-06
CYFIP2	cytoplasmic FMR1 interacting protein 2 [Source:HGNC Symbol;Acc:HGNC:13760]	36.8	77.4	**2.04**	1.22E-05
CLDN1	claudin 1 [Source:HGNC Symbol;Acc:HGNC:2032]	20.1	46.2	**2.04**	0.0003816
ANKRD28	ankyrin repeat domain 28 [Source:HGNC Symbol;Acc:HGNC:29024]	5083.9	10348.2	**2.03**	4.08E-87
PPP2CB	protein phosphatase 2 catalytic subunit beta [Source:HGNC Symbol;Acc:HGNC:9300]	2302.0	4719.9	**2.03**	6.83E-77
LYVE1	lymphatic vessel endothelial hyaluronan receptor 1 [Source:HGNC Symbol;Acc:HGNC:14687]	749.3	1571.9	**2.03**	2.13E-07
GNG7	G protein subunit gamma 7 [Source:HGNC Symbol;Acc:HGNC:4410]	27.9	60.6	**2.03**	1.21E-05
LINC01094	long intergenic non-protein coding RNA 1094 [Source:HGNC Symbol;Acc:HGNC:49219]	5.9	13.9	**2.03**	0.004669
LINC00921	long intergenic non-protein coding RNA 921 [Source:HGNC Symbol;Acc:HGNC:26830]	6.2	15.4	**2.03**	0.01651
ASB5	ankyrin repeat and SOCS box containing 5 [Source:HGNC Symbol;Acc:HGNC:17180]	3.2	8.7	**2.03**	0.02654
UAP1	UDP-N-acetylglucosamine pyrophosphorylase 1 [Source:HGNC Symbol;Acc:HGNC:12457]	9950.9	20101.3	**2.01**	6.64E-122
CFLAR	CASP8 and FADD like apoptosis regulator [Source:HGNC Symbol;Acc:HGNC:1876]	1026.7	2083.9	**2.01**	1.49E-79
TRIM56	tripartite motif containing 56 [Source:HGNC Symbol;Acc:HGNC:19028]	712.7	1433.3	**2.01**	5.38E-54
DHTKD1	dehydrogenase E1 and transketolase domain containing 1 [Source:HGNC Symbol;Acc:HGNC:23537]	212.7	431.6	**2.01**	2.03E-43
AKAP13	A-kinase anchoring protein 13 [Source:HGNC Symbol;Acc:HGNC:371]	2490.7	5062.0	**2.01**	6.29E-41
WASF3	WAS protein family member 3 [Source:HGNC Symbol;Acc:HGNC:12734]	346.6	710.0	**2.01**	9.62E-38
MGST1	microsomal glutathione S-transferase 1 [Source:HGNC Symbol;Acc:HGNC:7061]	102.3	203.9	**2.01**	6.58E-16
CYP1B1	cytochrome P450 family 1 subfamily B member 1 [Source:HGNC Symbol;Acc:HGNC:2597]	10032.7	20731.6	**2.01**	4.08E-15
IMPDH1P10	inosine monophosphate dehydrogenase 1 pseudogene 10 [Source:HGNC Symbol;Acc:HGNC:33965]	18.3	39.3	**2.01**	0.0003361
DEC1	deleted in esophageal cancer 1 [Source:HGNC Symbol;Acc:HGNC:23658]	4.2	10.8	**2.01**	0.0209
PLXNA2	plexin A2 [Source:HGNC Symbol;Acc:HGNC:9100]	545.0	1116.3	**2.00**	3.40E-33
DNAH11	dynein axonemal heavy chain 11 [Source:HGNC Symbol;Acc:HGNC:2942]	1108.8	2192.6	**2.00**	8.71E-28
RASSF8	Ras association domain family member 8 [Source:HGNC Symbol;Acc:HGNC:13232]	4103.8	8069.0	**1.99**	2.30E-83
EXOSC10	exosome component 10 [Source:HGNC Symbol;Acc:HGNC:9138]	1470.6	2963.2	**1.99**	2.68E-61
GBP2	guanylate binding protein 2 [Source:HGNC Symbol;Acc:HGNC:4183]	548.2	1090.1	**1.99**	5.14E-32
RALGPS2	Ral GEF with PH domain and SH3 binding motif 2 [Source:HGNC Symbol;Acc:HGNC:30279]	142.3	309.7	**1.99**	4.22E-09
FAM222A	family with sequence similarity 222 member A [Source:HGNC Symbol;Acc:HGNC:25915]	48.7	99.2	**1.99**	3.36E-06
RAMP2-AS1	RAMP2 antisense RNA 1 [Source:HGNC Symbol;Acc:HGNC:44358]	13.5	29.7	**1.99**	0.002154
HTR2A	5-hydroxytryptamine receptor 2A [Source:HGNC Symbol;Acc:HGNC:5293]	9.0	20.2	**1.99**	0.003825
ELMO3	engulfment and cell motility 3 [Source:HGNC Symbol;Acc:HGNC:17289]	5.3	12.0	**1.99**	0.02035
LRRC4	leucine rich repeat containing 4 [Source:HGNC Symbol;Acc:HGNC:15586]	4.6	11.5	**1.99**	0.02956
ZHX3	zinc fingers and homeoboxes 3 [Source:HGNC Symbol;Acc:HGNC:15935]	1069.2	2118.4	**1.97**	3.67E-64
YBX3	Y-box binding protein 3 [Source:HGNC Symbol;Acc:HGNC:2428]	2909.6	5827.5	**1.97**	4.28E-58
ADGRG2	adhesion G protein-coupled receptor G2 [Source:HGNC Symbol;Acc:HGNC:4516]	1059.7	2094.2	**1.97**	5.20E-40
ACSL4	acyl-CoA synthetase long chain family member 4 [Source:HGNC Symbol;Acc:HGNC:3571]	5491.3	11015.9	**1.97**	9.96E-38
SLC27A4	solute carrier family 27 member 4 [Source:HGNC Symbol;Acc:HGNC:10998]	276.4	551.7	**1.97**	3.86E-34
MPST	mercaptopyruvate sulfurtransferase [Source:HGNC Symbol;Acc:HGNC:7223]	142.3	290.1	**1.97**	1.07E-20
TREM1	triggering receptor expressed on myeloid cells 1 [Source:HGNC Symbol;Acc:HGNC:17760]	63.7	127.0	**1.97**	1.01E-09
STEAP4	STEAP4 metalloreductase [Source:HGNC Symbol;Acc:HGNC:21923]	1724.5	3780.4	**1.97**	6.57E-07
LINC01554	long intergenic non-protein coding RNA 1554 [Source:HGNC Symbol;Acc:HGNC:24687]	141.9	281.0	**1.96**	2.74E-22
DSG2-AS1	DSG2 antisense RNA 1 [Source:HGNC Symbol;Acc:HGNC:51311]	75.6	149.5	**1.96**	1.10E-13
AQP7P1	aquaporin 7 pseudogene 1 [Source:HGNC Symbol;Acc:HGNC:32048]	64.4	126.5	**1.96**	2.52E-10
ANGPT1	angiopoietin 1 [Source:HGNC Symbol;Acc:HGNC:484]	46.5	96.8	**1.96**	2.45E-07
P4HA3-AS1	P4HA3 antisense RNA 1 [Source:HGNC Symbol;Acc:HGNC:53160]	16.0	33.7	**1.96**	0.000132

XDH	xanthine dehydrogenase [Source:HGNC Symbol;Acc:HGNC:12805]	18.6	38.6	**1.96**	0.0004559
LINC00598	long intergenic non-protein coding RNA 598 [Source:HGNC Symbol;Acc:HGNC:42770]	12.0	26.0	**1.96**	0.003438
DCAF13P1	DDB1 and CUL4 associated factor 13 pseudogene 1 [Source:HGNC Symbol;Acc:HGNC:43866]	3.3	8.5	**1.96**	0.03991
NEBL	nebulette [Source:HGNC Symbol;Acc:HGNC:16932]	16213.9	31499.2	**1.95**	6.51E-133
EZH1	enhancer of zeste 1 polycomb repressive complex 2 subunit [Source:HGNC Symbol;Acc:HGNC:3526]	489.4	965.4	**1.95**	6.12E-50
ADRB2	adrenoceptor beta 2 [Source:HGNC Symbol;Acc:HGNC:286]	559.0	1092.0	**1.95**	8.61E-45
ANKRD6	ankyrin repeat domain 6 [Source:HGNC Symbol;Acc:HGNC:17280]	361.2	715.6	**1.95**	9.61E-37
THSD4	thrombospondin type 1 domain containing 4 [Source:HGNC Symbol;Acc:HGNC:25835]	475.6	927.0	**1.95**	6.48E-29
HMGA2	high mobility group AT-hook 2 [Source:HGNC Symbol;Acc:HGNC:5009]	108.2	212.9	**1.95**	5.47E-12
MARCH2	membrane associated ring-CH-type finger 2 [Source:HGNC Symbol;Acc:HGNC:28038]	82.5	163.7	**1.95**	1.50E-11
LINC01614	long intergenic non-protein coding RNA 1614 [Source:HGNC Symbol;Acc:HGNC:51847]	16.9	36.9	**1.95**	0.0007837
SLC11A1	solute carrier family 11 member 1 [Source:HGNC Symbol;Acc:HGNC:10907]	10.2	22.1	**1.95**	0.003156
LINC01694	long intergenic non-protein coding RNA 1694 [Source:HGNC Symbol;Acc:HGNC:52481]	6.2	14.8	**1.95**	0.02322
MXI1	MAX interactor 1, dimerization protein [Source:HGNC Symbol;Acc:HGNC:7534]	582.4	1130.3	**1.93**	6.87E-45
ALDH1A2	aldehyde dehydrogenase 1 family member A2 [Source:HGNC Symbol;Acc:HGNC:15472]	3110.4	5938.3	**1.93**	1.15E-32
PAG1	phosphoprotein membrane anchor with glycosphingolipid microdomains 1 [Source:HGNC Symbol;Acc:H	157.7	307.0	**1.93**	4.68E-26
CYTH1	cytohesin 1 [Source:HGNC Symbol;Acc:HGNC:9501]	432.1	840.6	**1.93**	6.68E-25
FOXD2	forkhead box D2 [Source:HGNC Symbol;Acc:HGNC:3803]	87.3	170.7	**1.93**	1.11E-09
NKILA	NF-kappaB interacting lncRNA [Source:HGNC Symbol;Acc:HGNC:51599]	48.9	98.3	**1.93**	2.57E-07
FOXC2-AS1	FOXC2 antisense RNA 1 [Source:HGNC Symbol;Acc:HGNC:50665]	17.2	35.1	**1.93**	3.53E-05
NPIPB4	nuclear pore complex interacting protein family member B4 [Source:HGNC Symbol;Acc:HGNC:41985]	14.7	30.3	**1.93**	0.0002216
TSC22D1-AS1	TSC22D1 antisense RNA 1 [Source:HGNC Symbol;Acc:HGNC:43684]	16.0	33.5	**1.93**	0.001115
C9orf47	chromosome 9 open reading frame 47 [Source:HGNC Symbol;Acc:HGNC:23669]	7.1	15.7	**1.93**	0.02699
IQGAP2	IQ motif containing GTPase activating protein 2 [Source:HGNC Symbol;Acc:HGNC:6111]	4.0	9.8	**1.93**	0.04027
MARVELD1	MARVEL domain containing 1 [Source:HGNC Symbol;Acc:HGNC:28674]	1014.3	1972.4	**1.92**	2.59E-27
GFOD1-AS1	GFOD1 antisense RNA 1 [Source:HGNC Symbol;Acc:HGNC:40956]	30.8	64.4	**1.92**	8.45E-05
TMEM92	transmembrane protein 92 [Source:HGNC Symbol;Acc:HGNC:26579]	4.5	10.7	**1.92**	0.0317
OTUD4P1	OTUD4 pseudogene 1 [Source:HGNC Symbol;Acc:HGNC:33912]	3.0	7.6	**1.92**	0.0499
SORT1	sortilin 1 [Source:HGNC Symbol;Acc:HGNC:11186]	2739.1	5177.4	**1.91**	5.78E-80
CAV1	caveolin 1 [Source:HGNC Symbol;Acc:HGNC:1527]	2175.9	4190.4	**1.91**	1.22E-67
TOB2	transducer of ERBB2, 2 [Source:HGNC Symbol;Acc:HGNC:11980]	660.5	1259.9	**1.91**	1.53E-36
PAIP2B	poly(A) binding protein interacting protein 2B [Source:HGNC Symbol;Acc:HGNC:29200]	165.4	318.2	**1.91**	3.79E-27
NPAS2	neuronal PAS domain protein 2 [Source:HGNC Symbol;Acc:HGNC:7895]	269.5	505.5	**1.91**	5.42E-12
TST	thiosulfate sulfurtransferase [Source:HGNC Symbol;Acc:HGNC:12388]	40.8	80.5	**1.91**	5.41E-09
AKNAD1	AKNA domain containing 1 [Source:HGNC Symbol;Acc:HGNC:28398]	21.5	44.0	**1.91**	0.000249
FOXF1	forkhead box F1 [Source:HGNC Symbol;Acc:HGNC:3809]	10.2	21.2	**1.91**	0.002062
C5orf49	chromosome 5 open reading frame 49 [Source:HGNC Symbol;Acc:HGNC:27028]	8.5	18.4	**1.91**	0.007778
CRYAB	crystallin alpha B [Source:HGNC Symbol;Acc:HGNC:2389]	953.2	1846.5	**1.89**	3.12E-42
SPIDR	scaffold protein involved in DNA repair [Source:HGNC Symbol;Acc:HGNC:28971]	933.9	1806.9	**1.89**	3.40E-35
KIAA0513	KIAA0513 [Source:HGNC Symbol;Acc:HGNC:29058]	584.7	1116.7	**1.89**	1.09E-34
HS1BP3	HCLS1 binding protein 3 [Source:HGNC Symbol;Acc:HGNC:24979]	190.2	363.2	**1.89**	6.62E-24
ZNF385B	zinc finger protein 385B [Source:HGNC Symbol;Acc:HGNC:26332]	274.3	534.7	**1.89**	4.95E-22
FGF13	fibroblast growth factor 13 [Source:HGNC Symbol;Acc:HGNC:3670]	101.5	193.1	**1.89**	1.93E-11
ITPRIPL1	ITPRIP like 1 [Source:HGNC Symbol;Acc:HGNC:29371]	54.2	107.2	**1.89**	3.67E-07
SESTD1	SEC14 and spectrin domain containing 1 [Source:HGNC Symbol;Acc:HGNC:18379]	1811.9	3410.3	**1.88**	7.56E-82
UBE2E1	ubiquitin conjugating enzyme E2 E1 [Source:HGNC Symbol;Acc:HGNC:12477]	1149.0	2166.5	**1.88**	4.38E-51
BCR	BCR, RhoGEF and GTPase activating protein [Source:HGNC Symbol;Acc:HGNC:1014]	572.1	1094.8	**1.88**	2.52E-37
JDP2	Jun dimerization protein 2 [Source:HGNC Symbol;Acc:HGNC:17546]	771.8	1433.1	**1.88**	3.83E-35
AHR	aryl hydrocarbon receptor [Source:HGNC Symbol;Acc:HGNC:348]	593.3	1105.6	**1.88**	3.95E-25
PTPN22	protein tyrosine phosphatase, non-receptor type 22 [Source:HGNC Symbol;Acc:HGNC:9652]	1154.2	2109.3	**1.88**	3.71E-23
CEP295NL	CEP295 N-terminal like [Source:HGNC Symbol;Acc:HGNC:44659]	718.6	1370.5	**1.88**	3.99E-20
CD36	CD36 molecule [Source:HGNC Symbol;Acc:HGNC:1663]	16.8	36.6	**1.88**	0.003323
NCOA3	nuclear receptor coactivator 3 [Source:HGNC Symbol;Acc:HGNC:7670]	2482.4	4651.1	**1.87**	1.07E-52
TIMP2	TIMP metallopeptidase inhibitor 2 [Source:HGNC Symbol;Acc:HGNC:11821]	3582.9	6790.3	**1.87**	4.08E-38
TBC1D16	TBC1 domain family member 16 [Source:HGNC Symbol;Acc:HGNC:28356]	344.6	643.1	**1.87**	3.05E-25
SH3BP4	SH3 domain binding protein 4 [Source:HGNC Symbol;Acc:HGNC:10826]	246.5	460.9	**1.87**	6.35E-18
ST6GALNAC5	ST6 N-acetylgalactosaminide alpha-2,6-sialyltransferase 5 [Source:HGNC Symbol;Acc:HGNC:19342]	240.5	462.3	**1.87**	2.36E-17
GAB2	GRB2 associated binding protein 2 [Source:HGNC Symbol;Acc:HGNC:14458]	455.1	887.3	**1.87**	8.85E-17
VSIR	V-set immunoregulatory receptor [Source:HGNC Symbol;Acc:HGNC:30085]	522.3	1010.4	**1.87**	8.00E-16
CHMP4C	charged multivesicular body protein 4C [Source:HGNC Symbol;Acc:HGNC:30599]	113.8	217.0	**1.87**	3.49E-12
PPL	periplakin [Source:HGNC Symbol;Acc:HGNC:9273]	145.9	287.5	**1.87**	1.95E-10
SVEP1	sushi, von Willebrand factor type A, EGF and pentraxin domain containing 1 [Source:HGNC Symbol;Acc:I	150.7	289.5	**1.87**	1.71E-09
MME	membrane metalloendopeptidase [Source:HGNC Symbol;Acc:HGNC:7154]	26.0	49.9	**1.87**	0.0001307
CYCSP24	cytochrome c, somatic pseudogene 24 [Source:HGNC Symbol;Acc:HGNC:24398]	8.6	18.3	**1.87**	0.01083
TMEM105	transmembrane protein 105 [Source:HGNC Symbol;Acc:HGNC:26794]	5.4	11.5	**1.87**	0.01757
LINC02361	long intergenic non-protein coding RNA 2361 [Source:HGNC Symbol;Acc:HGNC:53283]	4.4	10.1	**1.87**	0.03863
NAV3	neuron navigator 3 [Source:HGNC Symbol;Acc:HGNC:15998]	7.1	16.0	**1.87**	0.04159
ORM2	orosomucoid 2 [Source:HGNC Symbol;Acc:HGNC:8499]	5.3	12.0	**1.87**	0.04356
RTN3	reticulon 3 [Source:HGNC Symbol;Acc:HGNC:10469]	1188.3	2218.8	**1.85**	8.75E-54
DSG2	desmoglein 2 [Source:HGNC Symbol;Acc:HGNC:3049]	1268.8	2351.7	**1.85**	1.68E-46
TPST1	tyrosylprotein sulfotransferase 1 [Source:HGNC Symbol;Acc:HGNC:12020]	1170.4	2168.4	**1.85**	1.70E-45
SNED1	sushi, nidogen and EGF like domains 1 [Source:HGNC Symbol;Acc:HGNC:24696]	605.0	1131.8	**1.85**	6.00E-34

TRAFD1	TRAF-type zinc finger domain containing 1 [Source:HGNC Symbol;Acc:HGNC:24808]	1103.9	2124.8	**1.85**	2.46E-32
DIAPH1	diaphanous related formin 1 [Source:HGNC Symbol;Acc:HGNC:2876]	2058.3	3894.2	**1.85**	2.39E-26
FAM241A	family with sequence similarity 241 member A [Source:HGNC Symbol;Acc:HGNC:26813]	139.4	261.1	**1.85**	1.07E-16
RAB3D	RAB3D, member RAS oncogene family [Source:HGNC Symbol;Acc:HGNC:9779]	142.3	267.8	**1.85**	1.21E-16
PRICKLE2-AS1	PRICKLE2 antisense RNA 1 [Source:HGNC Symbol;Acc:HGNC:40916]	574.8	1029.5	**1.85**	2.70E-09
GFOD1	glucose-fructose oxidoreductase domain containing 1 [Source:HGNC Symbol;Acc:HGNC:21096]	882.8	1705.6	**1.85**	2.50E-08
ARID3A	AT-rich interaction domain 3A [Source:HGNC Symbol;Acc:HGNC:3031]	24.8	47.9	**1.85**	4.35E-05
LINC01585	long intergenic non-protein coding RNA 1585 [Source:HGNC Symbol;Acc:HGNC:51432]	8.4	18.3	**1.85**	0.02962
CD109	CD109 molecule [Source:HGNC Symbol;Acc:HGNC:21685]	8821.3	16663.1	**1.84**	1.17E-29
C5orf63	chromosome 5 open reading frame 63 [Source:HGNC Symbol;Acc:HGNC:40051]	113.6	213.8	**1.84**	4.40E-14
PLEKHM1P1	pleckstrin homology and RUN domain containing M1 pseudogene 1 [Source:HGNC Symbol;Acc:HGNC:3!	141.9	266.8	**1.84**	9.12E-14
TRIM9	tripartite motif containing 9 [Source:HGNC Symbol;Acc:HGNC:16288]	39.4	75.4	**1.84**	6.38E-05
CLEC2B	C-type lectin domain family 2 member B [Source:HGNC Symbol;Acc:HGNC:2053]	7.9	17.3	**1.84**	0.01946
SLC38A1	solute carrier family 38 member 1 [Source:HGNC Symbol;Acc:HGNC:13447]	5926.4	10866.6	**1.83**	2.04E-74
MED28	mediator complex subunit 28 [Source:HGNC Symbol;Acc:HGNC:24628]	654.4	1197.9	**1.83**	3.01E-51
SDC4	syndecan 4 [Source:HGNC Symbol;Acc:HGNC:10661]	5128.9	9280.4	**1.83**	1.95E-41
LIFR	LIF receptor alpha [Source:HGNC Symbol;Acc:HGNC:6597]	3531.6	6561.4	**1.83**	5.49E-32
STXBP5	syntaxin binding protein 5 [Source:HGNC Symbol;Acc:HGNC:19665]	1824.5	3427.1	**1.83**	2.48E-25
ACOT7	acyl-CoA thioesterase 7 [Source:HGNC Symbol;Acc:HGNC:24157]	340.0	624.6	**1.83**	1.96E-23
ABCA9	ATP binding cassette subfamily A member 9 [Source:HGNC Symbol;Acc:HGNC:39]	50.7	98.1	**1.83**	8.77E-07
CES1	carboxylesterase 1 [Source:HGNC Symbol;Acc:HGNC:1863]	35.4	65.4	**1.83**	1.39E-06
GMFG	glia maturation factor gamma [Source:HGNC Symbol;Acc:HGNC:4374]	22.1	44.5	**1.83**	0.001112
CD99P1	CD99 molecule pseudogene 1 [Source:HGNC Symbol;Acc:HGNC:7083]	17.4	34.4	**1.83**	0.002391
PTPRR	protein tyrosine phosphatase, receptor type R [Source:HGNC Symbol;Acc:HGNC:9680]	6.0	12.5	**1.83**	0.02561
GATA2-AS1	GATA2 antisense RNA 1 [Source:HGNC Symbol;Acc:HGNC:51108]	6.9	14.5	**1.83**	0.02577
TMEM92-AS1	TMEM92 antisense RNA 1 [Source:HGNC Symbol;Acc:HGNC:50442]	4.4	9.8	**1.83**	0.04562
UBAP1	ubiquitin associated protein 1 [Source:HGNC Symbol;Acc:HGNC:12461]	925.2	1697.0	**1.82**	1.91E-27
RCAN2	regulator of calcineurin 2 [Source:HGNC Symbol;Acc:HGNC:3041]	337.1	611.8	**1.82**	5.55E-15
CDK6	cyclin dependent kinase 6 [Source:HGNC Symbol;Acc:HGNC:1777]	1391.8	2633.7	**1.82**	1.07E-09
NAMPTP1	nicotinamide phosphoribosyltransferase pseudogene 1 [Source:HGNC Symbol;Acc:HGNC:17633]	77.9	147.1	**1.82**	3.34E-09
VGLL3	vestigial like family member 3 [Source:HGNC Symbol;Acc:HGNC:24327]	34.3	63.4	**1.82**	0.0002183
INS-IGF2	INS-IGF2 readthrough [Source:HGNC Symbol;Acc:HGNC:33527]	15.5	30.4	**1.82**	0.00179
HEBP2	heme binding protein 2 [Source:HGNC Symbol;Acc:HGNC:15716]	838.6	1504.5	**1.80**	5.31E-65
HIP1R	huntingtin interacting protein 1 related [Source:HGNC Symbol;Acc:HGNC:18415]	321.5	592.4	**1.80**	4.36E-24
COX10-AS1	COX10 antisense RNA 1 [Source:HGNC Symbol;Acc:HGNC:38873]	115.8	208.8	**1.80**	1.22E-16
AVIL	advillin [Source:HGNC Symbol;Acc:HGNC:14188]	119.8	217.2	**1.80**	2.19E-13
PITPNC1	phosphatidylinositol transfer protein cytoplasmic 1 [Source:HGNC Symbol;Acc:HGNC:21045]	166.1	313.4	**1.80**	3.06E-08
NR2F1	nuclear receptor subfamily 2 group F member 1 [Source:HGNC Symbol;Acc:HGNC:7975]	7.4	15.6	**1.80**	0.0448
SYNE3	spectrin repeat containing nuclear envelope family member 3 [Source:HGNC Symbol;Acc:HGNC:19861]	1202.7	2151.2	**1.79**	1.17E-82
SPTBN1	spectrin beta, non-erythrocytic 1 [Source:HGNC Symbol;Acc:HGNC:11275]	14902.0	27009.8	**1.79**	1.21E-52
OSBPL10	oxysterol binding protein like 10 [Source:HGNC Symbol;Acc:HGNC:16395]	1280.3	2308.3	**1.79**	4.00E-51
P4HA3	prolyl 4-hydroxylase subunit alpha 3 [Source:HGNC Symbol;Acc:HGNC:30135]	689.1	1267.3	**1.79**	9.66E-23
PPM1L	protein phosphatase, Mg2+/Mn2+ dependent 1L [Source:HGNC Symbol;Acc:HGNC:16381]	219.2	414.3	**1.79**	3.94E-12
HSPA8P4	heat shock protein family A (Hsp70) member 8 pseudogene 4 [Source:HGNC Symbol;Acc:HGNC:44919]	31.1	58.9	**1.79**	0.0001026
ZFP36L1	ZFP36 ring finger protein like 1 [Source:HGNC Symbol;Acc:HGNC:1107]	520.1	952.5	**1.79**	0.0007434
MRC1	mannose receptor C-type 1 [Source:HGNC Symbol;Acc:HGNC:7228]	6.1	12.4	**1.79**	0.03675
HRASLS5	HRAS like suppressor family member 5 [Source:HGNC Symbol;Acc:HGNC:24978]	10.8	23.3	**1.79**	0.04635
C10orf113	chromosome 10 open reading frame 113 [Source:HGNC Symbol;Acc:HGNC:31447]	6.2	13.0	**1.79**	0.04662
PHACTR2	phosphatase and actin regulator 2 [Source:HGNC Symbol;Acc:HGNC:20956]	3131.1	5577.9	**1.78**	7.51E-104
ARHGAP10	Rho GTPase activating protein 10 [Source:HGNC Symbol;Acc:HGNC:26099]	1453.5	2611.0	**1.78**	1.33E-65
MOSMO	modulator of smoothened [Source:HGNC Symbol;Acc:HGNC:27087]	684.4	1220.8	**1.78**	4.50E-37
RETSAT	retinol saturase [Source:HGNC Symbol;Acc:HGNC:25991]	525.8	949.0	**1.78**	1.15E-30
TMEM154	transmembrane protein 154 [Source:HGNC Symbol;Acc:HGNC:26489]	497.8	889.3	**1.78**	1.24E-30
GTF2IRD1	GTF2I repeat domain containing 1 [Source:HGNC Symbol;Acc:HGNC:4661]	309.9	558.0	**1.78**	1.28E-19
AVPI1	arginine vasopressin induced 1 [Source:HGNC Symbol;Acc:HGNC:30898]	183.9	332.8	**1.78**	8.92E-19
FMNL2	formin like 2 [Source:HGNC Symbol;Acc:HGNC:18267]	1243.7	2200.0	**1.78**	1.45E-18
ABCC3	ATP binding cassette subfamily C member 3 [Source:HGNC Symbol;Acc:HGNC:54]	75.1	133.1	**1.78**	2.05E-08
EFCAB1	EF-hand calcium binding domain 1 [Source:HGNC Symbol;Acc:HGNC:25678]	35.5	65.1	**1.78**	1.93E-05
ANXA4	annexin A4 [Source:HGNC Symbol;Acc:HGNC:542]	2647.5	4701.5	**1.77**	2.51E-62
MLKL	mixed lineage kinase domain like pseudokinase [Source:HGNC Symbol;Acc:HGNC:26617]	416.9	733.4	**1.77**	2.94E-36
POC1B-GALNT4	POC1B-GALNT4 readthrough [Source:HGNC Symbol;Acc:HGNC:42957]	2936.0	5236.8	**1.77**	2.04E-30
GALNT4	polypeptide N-acetylgalactosaminyltransferase 4 [Source:HGNC Symbol;Acc:HGNC:4126]	2961.5	5284.3	**1.77**	3.08E-30
BBS12	Bardet-Biedl syndrome 12 [Source:HGNC Symbol;Acc:HGNC:26648]	315.1	564.2	**1.77**	3.21E-26
GPR137B	G protein-coupled receptor 137B [Source:HGNC Symbol;Acc:HGNC:11862]	128.5	230.0	**1.77**	6.73E-18
TYMP	thymidine phosphorylase [Source:HGNC Symbol;Acc:HGNC:3148]	236.0	428.4	**1.77**	1.68E-15
DUSP23	dual specificity phosphatase 23 [Source:HGNC Symbol;Acc:HGNC:21480]	133.2	236.0	**1.77**	1.24E-13
CCDC13	coiled-coil domain containing 13 [Source:HGNC Symbol;Acc:HGNC:26358]	21.8	39.3	**1.77**	0.0004658
HDX	highly divergent homeobox [Source:HGNC Symbol;Acc:HGNC:26411]	12.8	24.1	**1.77**	0.008535
ABCA9-AS1	ABCA9 antisense RNA 1 [Source:HGNC Symbol;Acc:HGNC:39983]	8.9	17.7	**1.77**	0.02142
PGAM1P8	phosphoglycerate mutase 1 pseudogene 8 [Source:HGNC Symbol;Acc:HGNC:42455]	7.9	15.6	**1.77**	0.03657
CFAP58-DT	CFAP58 divergent transcript [Source:HGNC Symbol;Acc:HGNC:45243]	32.9	58.4	**1.77**	0.03937
PDE4DIP	phosphodiesterase 4D interacting protein [Source:HGNC Symbol;Acc:HGNC:15580]	5519.8	9720.2	**1.75**	8.37E-80

GPSM2	G protein signaling modulator 2 [Source:HGNC Symbol;Acc:HGNC:29501]	2725.5	4777.4	**1.75**	3.31E-62
RSPRY1	ring finger and SPRY domain containing 1 [Source:HGNC Symbol;Acc:HGNC:29420]	1302.9	2289.5	**1.75**	3.28E-61
ITGBL1	integrin subunit beta like 1 [Source:HGNC Symbol;Acc:HGNC:6164]	2824.3	4962.2	**1.75**	9.22E-52
ZFAND5	zinc finger AN1-type containing 5 [Source:HGNC Symbol;Acc:HGNC:13008]	3968.8	7063.3	**1.75**	1.06E-34
PRICKLE2	prickle planar cell polarity protein 2 [Source:HGNC Symbol;Acc:HGNC:20340]	1297.1	2271.1	**1.75**	1.49E-28
PLEKHM1	pleckstrin homology and RUN domain containing M1 [Source:HGNC Symbol;Acc:HGNC:29017]	257.0	458.4	**1.75**	7.03E-19
PDGFD	platelet derived growth factor D [Source:HGNC Symbol;Acc:HGNC:30620]	573.1	1006.2	**1.75**	1.44E-15
MLYCD	malonyl-CoA decarboxylase [Source:HGNC Symbol;Acc:HGNC:7150]	159.2	284.7	**1.75**	1.66E-10
ST3GAL5	ST3 beta-galactoside alpha-2,3-sialyltransferase 5 [Source:HGNC Symbol;Acc:HGNC:10872]	84.1	146.6	**1.75**	8.60E-09
ZC3H12A	zinc finger CCCH-type containing 12A [Source:HGNC Symbol;Acc:HGNC:26259]	48.9	88.5	**1.75**	1.54E-05
MYOM1	myomesin 1 [Source:HGNC Symbol;Acc:HGNC:7613]	69.4	132.8	**1.75**	1.95E-05
TLL1	tolloid like 1 [Source:HGNC Symbol;Acc:HGNC:11843]	73.5	130.2	**1.75**	4.69E-05
CBS	cystathionine-beta-synthase [Source:HGNC Symbol;Acc:HGNC:1550]	33.4	60.9	**1.75**	5.42E-05
RNF175	ring finger protein 175 [Source:HGNC Symbol;Acc:HGNC:27735]	12.1	22.9	**1.75**	0.01075
ATL3	atlastin GTPase 3 [Source:HGNC Symbol;Acc:HGNC:24526]	10740.3	18756.9	**1.74**	9.27E-121
USP32	ubiquitin specific peptidase 32 [Source:HGNC Symbol;Acc:HGNC:19143]	1545.1	2708.8	**1.74**	4.00E-55
FGF14	fibroblast growth factor 14 [Source:HGNC Symbol;Acc:HGNC:3671]	1467.2	2559.9	**1.74**	1.24E-50
GNPNAT1	glucosamine-phosphate N-acetyltransferase 1 [Source:HGNC Symbol;Acc:HGNC:19980]	1780.6	3181.8	**1.74**	3.81E-30
ARMC9	armadillo repeat containing 9 [Source:HGNC Symbol;Acc:HGNC:20730]	816.4	1466.5	**1.74**	2.20E-28
GCH1	GTP cyclohydrolase 1 [Source:HGNC Symbol;Acc:HGNC:4193]	620.3	1085.9	**1.74**	1.93E-27
DSP	desmoplakin [Source:HGNC Symbol;Acc:HGNC:3052]	1979.4	3399.6	**1.74**	1.34E-23
PRG4	proteoglycan 4 [Source:HGNC Symbol;Acc:HGNC:9364]	1527.0	2831.0	**1.74**	1.72E-20
ABHD6	abhydrolase domain containing 6 [Source:HGNC Symbol;Acc:HGNC:21398]	279.8	499.8	**1.74**	1.13E-14
STAMBPL1	STAM binding protein like 1 [Source:HGNC Symbol;Acc:HGNC:24105]	304.5	536.1	**1.74**	3.69E-13
MKL1	megakaryoblastic leukemia (translocation) 1 [Source:HGNC Symbol;Acc:HGNC:14334]	143.6	253.6	**1.74**	2.61E-12
SLC43A2	solute carrier family 43 member 2 [Source:HGNC Symbol;Acc:HGNC:23087]	119.0	209.9	**1.74**	2.21E-09
CDK2AP2	cyclin dependent kinase 2 associated protein 2 [Source:HGNC Symbol;Acc:HGNC:30833]	120.9	212.7	**1.74**	7.67E-09
NPTN-IT1	NPTN intronic transcript 1 [Source:HGNC Symbol;Acc:HGNC:45091]	41.9	75.1	**1.74**	3.45E-05
KCNJ8	potassium voltage-gated channel subfamily J member 8 [Source:HGNC Symbol;Acc:HGNC:6269]	9.9	19.2	**1.74**	0.03822
IL6ST	interleukin 6 signal transducer [Source:HGNC Symbol;Acc:HGNC:6021]	19551.3	33971.5	**1.73**	6.81E-82
PM20D2	peptidase M20 domain containing 2 [Source:HGNC Symbol;Acc:HGNC:21408]	840.8	1466.8	**1.73**	2.87E-49
WEE1	WEE1 G2 checkpoint kinase [Source:HGNC Symbol;Acc:HGNC:12761]	471.6	816.0	**1.73**	3.38E-43
JUNB	JunB proto-oncogene, AP-1 transcription factor subunit [Source:HGNC Symbol;Acc:HGNC:6205]	593.3	1028.3	**1.73**	2.31E-40
SLC31A2	solute carrier family 31 member 2 [Source:HGNC Symbol;Acc:HGNC:11017]	785.6	1382.7	**1.73**	3.11E-30
SLC35D2	solute carrier family 35 member D2 [Source:HGNC Symbol;Acc:HGNC:20799]	266.7	465.7	**1.73**	4.48E-17
ZNF860	zinc finger protein 860 [Source:HGNC Symbol;Acc:HGNC:34513]	171.9	298.4	**1.73**	2.38E-14
MAP1LC3A	microtubule associated protein 1 light chain 3 alpha [Source:HGNC Symbol;Acc:HGNC:6838]	15.4	29.6	**1.73**	0.01822
ABHD2	abhydrolase domain containing 2 [Source:HGNC Symbol;Acc:HGNC:18717]	7459.7	12893.5	**1.72**	2.23E-66
RIOK3	RIO kinase 3 [Source:HGNC Symbol;Acc:HGNC:11451]	2814.8	4893.8	**1.72**	6.05E-42
CST3	cystatin C [Source:HGNC Symbol;Acc:HGNC:2475]	821.8	1431.5	**1.72**	2.07E-37
EFNA5	ephrin A5 [Source:HGNC Symbol;Acc:HGNC:3225]	481.8	830.5	**1.72**	3.69E-33
AGTRAP	angiotensin II receptor associated protein [Source:HGNC Symbol;Acc:HGNC:13539]	247.6	426.4	**1.72**	2.09E-32
SMPDL3A	sphingomyelin phosphodiesterase acid like 3A [Source:HGNC Symbol;Acc:HGNC:17389]	195.7	343.6	**1.72**	1.55E-20
NAMPT	nicotinamide phosphoribosyltransferase [Source:HGNC Symbol;Acc:HGNC:30092]	9016.4	16135.0	**1.72**	4.20E-13
LINC02246	long intergenic non-protein coding RNA 2246 [Source:HGNC Symbol;Acc:HGNC:53135]	96.8	171.8	**1.72**	3.22E-10
MLPH	melanophilin [Source:HGNC Symbol;Acc:HGNC:29643]	121.2	207.7	**1.72**	6.42E-10
ZIC1	Zic family member 1 [Source:HGNC Symbol;Acc:HGNC:12872]	74.2	126.9	**1.72**	8.54E-09
JUP	junction plakoglobin [Source:HGNC Symbol;Acc:HGNC:6207]	27.2	48.9	**1.72**	0.0003705
ARHGAP45	Rho GTPase activating protein 45 [Source:HGNC Symbol;Acc:HGNC:17102]	25.6	47.4	**1.72**	0.001308
FOXF2	forkhead box F2 [Source:HGNC Symbol;Acc:HGNC:3810]	12.5	22.9	**1.72**	0.01323
CD59	CD59 molecule (CD59 blood group) [Source:HGNC Symbol;Acc:HGNC:1689]	26473.6	45364.8	**1.71**	6.01E-52
ELL2	elongation factor for RNA polymerase II 2 [Source:HGNC Symbol;Acc:HGNC:17064]	12433.2	21297.5	**1.71**	4.88E-46
WBP1L	WW domain binding protein 1 like [Source:HGNC Symbol;Acc:HGNC:23510]	636.4	1087.0	**1.71**	1.19E-37
CALCOCO2	calcium binding and coiled-coil domain 2 [Source:HGNC Symbol;Acc:HGNC:29912]	2117.6	3637.7	**1.71**	2.70E-26
DUSP22	dual specificity phosphatase 22 [Source:HGNC Symbol;Acc:HGNC:16077]	496.5	847.1	**1.71**	1.04E-22
FAM184B	family with sequence similarity 184 member B [Source:HGNC Symbol;Acc:HGNC:29235]	98.5	170.1	**1.71**	5.57E-12
CYP3A5	cytochrome P450 family 3 subfamily A member 5 [Source:HGNC Symbol;Acc:HGNC:2638]	134.4	233.5	**1.71**	7.59E-09
TMSB10	thymosin beta 10 [Source:HGNC Symbol;Acc:HGNC:11879]	6471.7	11218.5	**1.71**	1.73E-08
LONRF3	LON peptidase N-terminal domain and ring finger 3 [Source:HGNC Symbol;Acc:HGNC:21152]	110.3	191.3	**1.71**	5.86E-07
CFAP58	cilia and flagella associated protein 58 [Source:HGNC Symbol;Acc:HGNC:26676]	32.9	58.4	**1.71**	1.77E-05
AC104971.1	uncharacterized LOC101927322 [Source:NCBI gene;Acc:101927322]	14.0	25.7	**1.71**	0.009677
TBXAS1	thromboxane A synthase 1 [Source:HGNC Symbol;Acc:HGNC:11609]	13.5	24.4	**1.71**	0.01354
ST6GALNAC3	ST6 N-acetylgalactosaminide alpha-2,6-sialyltransferase 3 [Source:HGNC Symbol;Acc:HGNC:19343]	6.0	11.6	**1.71**	0.04829
AC245297.1	phosphodiesterase 4D interacting protein-like [Source:NCBI gene;Acc:653513]	721.5	1226.6	**1.69**	4.85E-43
POLD4	DNA polymerase delta 4, accessory subunit [Source:HGNC Symbol;Acc:HGNC:14106]	259.2	440.1	**1.69**	3.81E-21
JCAD	junctional cadherin 5 associated [Source:HGNC Symbol;Acc:HGNC:29283]	770.8	1343.7	**1.69**	2.26E-20
TSPAN14	tetraspanin 14 [Source:HGNC Symbol;Acc:HGNC:23303]	140.3	240.9	**1.69**	3.89E-13
MTERF2	mitochondrial transcription termination factor 2 [Source:HGNC Symbol;Acc:HGNC:30779]	184.8	317.3	**1.69**	6.34E-12
SLC22A15	solute carrier family 22 member 15 [Source:HGNC Symbol;Acc:HGNC:20301]	312.2	533.1	**1.69**	4.49E-11
MARCH1	membrane associated ring-CH-type finger 1 [Source:HGNC Symbol;Acc:HGNC:26077]	178.7	314.2	**1.69**	6.17E-11
PLK3	polo like kinase 3 [Source:HGNC Symbol;Acc:HGNC:2154]	115.2	198.7	**1.69**	2.02E-10
MAP3K7CL	MAP3K7 C-terminal like [Source:HGNC Symbol;Acc:HGNC:16457]	122.5	212.4	**1.69**	6.33E-07

ARMC2	armadillo repeat containing 2 [Source:HGNC Symbol;Acc:HGNC:23045]	72.3	123.8	**1.69**	9.57E-07
RMDN2-AS1	RMDN2 antisense RNA 1 [Source:HGNC Symbol;Acc:HGNC:41150]	22.9	40.8	**1.69**	0.001076
UBE2FP1	ubiquitin conjugating enzyme E2 F (putative) pseudogene 1 [Source:HGNC Symbol;Acc:HGNC:44535]	14.1	25.1	**1.69**	0.007343
CMKLR1	chemerin chemokine-like receptor 1 [Source:HGNC Symbol;Acc:HGNC:2121]	1968.9	3361.8	**1.68**	1.72E-31
THADA	THADA, armadillo repeat containing [Source:HGNC Symbol;Acc:HGNC:19217]	1293.4	2188.8	**1.68**	1.26E-30
BLM	Bloom syndrome RecQ like helicase [Source:HGNC Symbol;Acc:HGNC:1058]	257.7	435.4	**1.68**	1.33E-08
KPNA2P3	karyopherin subunit alpha 2 pseudogene 3 [Source:HGNC Symbol;Acc:HGNC:52870]	70.2	118.7	**1.68**	2.07E-07
GPC5	glypican 5 [Source:HGNC Symbol;Acc:HGNC:4453]	141.8	246.4	**1.68**	2.26E-07
NXPH3	neurexophilin 3 [Source:HGNC Symbol;Acc:HGNC:8077]	37.9	66.7	**1.68**	0.0005002
MYO16	myosin XVI [Source:HGNC Symbol;Acc:HGNC:29822]	9.7	17.7	**1.68**	0.03637
MSI2	musashi RNA binding protein 2 [Source:HGNC Symbol;Acc:HGNC:18585]	4804.8	8025.8	**1.67**	1.07E-107
PMP22	peripheral myelin protein 22 [Source:HGNC Symbol;Acc:HGNC:9118]	3852.2	6457.8	**1.67**	1.20E-67
EMP2	epithelial membrane protein 2 [Source:HGNC Symbol;Acc:HGNC:3334]	3641.7	6099.3	**1.67**	2.51E-64
LTBR	lymphotoxin beta receptor [Source:HGNC Symbol;Acc:HGNC:6718]	1094.3	1832.0	**1.67**	7.98E-60
ATP6V1B2	ATPase H+ transporting V1 subunit B2 [Source:HGNC Symbol;Acc:HGNC:854]	2129.7	3571.6	**1.67**	8.66E-45
PCYT2	phosphate cytidylyltransferase 2, ethanolamine [Source:HGNC Symbol;Acc:HGNC:8756]	419.7	704.6	**1.67**	2.21E-24
F5	coagulation factor V [Source:HGNC Symbol;Acc:HGNC:3542]	167.3	281.6	**1.67**	2.42E-15
CCL20	C-C motif chemokine ligand 20 [Source:HGNC Symbol;Acc:HGNC:10619]	306.3	478.8	**1.67**	1.77E-11
HACD1	3-hydroxyacyl-CoA dehydratase 1 [Source:HGNC Symbol;Acc:HGNC:9639]	143.3	239.3	**1.67**	2.70E-08
TRAF5	TNF receptor associated factor 5 [Source:HGNC Symbol;Acc:HGNC:12035]	88.3	148.2	**1.67**	4.08E-07
COMTD1	catechol-O-methyltransferase domain containing 1 [Source:HGNC Symbol;Acc:HGNC:26309]	47.0	80.6	**1.67**	4.44E-05
AMFR	autocrine motility factor receptor [Source:HGNC Symbol;Acc:HGNC:463]	2569.1	4292.3	**1.66**	1.72E-64
NRIP1	nuclear receptor interacting protein 1 [Source:HGNC Symbol;Acc:HGNC:8001]	3285.3	5491.8	**1.66**	1.27E-53
COPS8	COP9 signalosome subunit 8 [Source:HGNC Symbol;Acc:HGNC:24335]	1090.9	1816.6	**1.66**	1.53E-42
PTPN1	protein tyrosine phosphatase, non-receptor type 1 [Source:HGNC Symbol;Acc:HGNC:9642]	780.4	1305.2	**1.66**	5.41E-42
ZBTB43	zinc finger and BTB domain containing 43 [Source:HGNC Symbol;Acc:HGNC:17908]	732.9	1227.5	**1.66**	4.27E-41
SASH1	SAM and SH3 domain containing 1 [Source:HGNC Symbol;Acc:HGNC:19182]	1882.4	3135.6	**1.66**	2.38E-33
TSPYL2	TSPY like 2 [Source:HGNC Symbol;Acc:HGNC:24358]	1134.0	1913.3	**1.66**	1.70E-30
NGEF	neuronal guanine nucleotide exchange factor [Source:HGNC Symbol;Acc:HGNC:7807]	1425.6	2414.5	**1.66**	9.39E-21
DAAM2	dishevelled associated activator of morphogenesis 2 [Source:HGNC Symbol;Acc:HGNC:18143]	690.3	1153.2	**1.66**	2.20E-17
TMEM150A	transmembrane protein 150A [Source:HGNC Symbol;Acc:HGNC:24677]	43.3	74.6	**1.66**	0.000209
BDH1	3-hydroxybutyrate dehydrogenase 1 [Source:HGNC Symbol;Acc:HGNC:1027]	37.5	63.4	**1.66**	0.001101
RBMS1P1	RNA binding motif single stranded interacting protein 1 pseudogene 1 [Source:HGNC Symbol;Acc:HGNC	16.4	28.0	**1.66**	0.006079
MT-RNR1	mitochondrially encoded 12S RNA [Source:HGNC Symbol;Acc:HGNC:7470]	272.8	484.6	**1.66**	0.01998
NGFR	nerve growth factor receptor [Source:HGNC Symbol;Acc:HGNC:7809]	15.1	27.9	**1.66**	0.03971
SLC7A6	solute carrier family 7 member 6 [Source:HGNC Symbol;Acc:HGNC:11064]	770.0	1266.0	**1.65**	8.26E-52
FUT4	fucosyltransferase 4 [Source:HGNC Symbol;Acc:HGNC:4015]	464.4	767.1	**1.65**	1.46E-26
SELENOP	selenoprotein P [Source:HGNC Symbol;Acc:HGNC:10751]	797.9	1315.9	**1.65**	1.33E-19
MLXIP	MLX interacting protein [Source:HGNC Symbol;Acc:HGNC:17055]	230.0	388.0	**1.65**	2.26E-14
BCAT1	branched chain amino acid transaminase 1 [Source:HGNC Symbol;Acc:HGNC:976]	34210.6	57229.5	**1.65**	2.00E-09
LIPE-AS1	LIPE antisense RNA 1 [Source:HGNC Symbol;Acc:HGNC:48589]	62.8	106.1	**1.65**	5.38E-06
FBLN1	fibulin 1 [Source:HGNC Symbol;Acc:HGNC:3600]	60.3	103.1	**1.65**	9.46E-06
KCNH1	potassium voltage-gated channel subfamily H member 1 [Source:HGNC Symbol;Acc:HGNC:6250]	29.3	51.2	**1.65**	0.003868
RPL37P6	ribosomal protein L37 pseudogene 6 [Source:HGNC Symbol;Acc:HGNC:31080]	13.3	23.1	**1.65**	0.0217
MALT1	MALT1 paracaspase [Source:HGNC Symbol;Acc:HGNC:6819]	1531.0	2521.5	**1.64**	1.81E-40
KLF4	Kruppel like factor 4 [Source:HGNC Symbol;Acc:HGNC:6348]	786.5	1293.2	**1.64**	1.34E-18
PLAG1	PLAG1 zinc finger [Source:HGNC Symbol;Acc:HGNC:9045]	307.1	496.9	**1.64**	4.53E-17
SH3RF3	SH3 domain containing ring finger 3 [Source:HGNC Symbol;Acc:HGNC:24699]	204.3	335.0	**1.64**	1.81E-14
SLC39A14	solute carrier family 39 member 14 [Source:HGNC Symbol;Acc:HGNC:20858]	39362.6	65245.8	**1.64**	2.13E-08
KLF8	Kruppel like factor 8 [Source:HGNC Symbol;Acc:HGNC:6351]	87.1	142.5	**1.64**	4.65E-08
FAM20A	FAM20A, golgi associated secretory pathway pseudokinase [Source:HGNC Symbol;Acc:HGNC:23015]	60.4	101.5	**1.64**	2.29E-06
POC1B-AS1	POC1B antisense RNA 1 [Source:HGNC Symbol;Acc:HGNC:52949]	49.6	82.3	**1.64**	3.47E-06
PRICKLE2-AS3	PRICKLE2 antisense RNA 3 [Source:HGNC Symbol;Acc:HGNC:40918]	54.2	91.5	**1.64**	3.20E-05
JADE3	jade family PHD finger 3 [Source:HGNC Symbol;Acc:HGNC:22982]	39.5	67.0	**1.64**	0.003516
LINC01235	long intergenic non-protein coding RNA 1235 [Source:HGNC Symbol;Acc:HGNC:49769]	24.0	38.1	**1.64**	0.01373
SIK1B	salt inducible kinase 1B (putative) [Source:HGNC Symbol;Acc:HGNC:52389]	13.3	23.2	**1.64**	0.03726
RSPH4A	radial spoke head 4 homolog A [Source:HGNC Symbol;Acc:HGNC:21558]	9.2	16.1	**1.64**	0.04694
IGFBP6	insulin like growth factor binding protein 6 [Source:HGNC Symbol;Acc:HGNC:5475]	4200.6	6924.3	**1.62**	8.41E-37
MTOR	mechanistic target of rapamycin kinase [Source:HGNC Symbol;Acc:HGNC:3942]	1095.8	1791.9	**1.62**	9.50E-32
FADS3	fatty acid desaturase 3 [Source:HGNC Symbol;Acc:HGNC:3576]	682.2	1109.7	**1.62**	2.14E-27
WDR13	WD repeat domain 13 [Source:HGNC Symbol;Acc:HGNC:14352]	394.0	640.3	**1.62**	8.95E-27
LRRFIP1	LRR binding FLII interacting protein 1 [Source:HGNC Symbol;Acc:HGNC:6702]	6488.1	10765.1	**1.62**	9.38E-27
DSC2	desmocollin 2 [Source:HGNC Symbol;Acc:HGNC:3036]	418.2	675.3	**1.62**	1.65E-22
CIPC	CLOCK interacting pacemaker [Source:HGNC Symbol;Acc:HGNC:20365]	308.6	505.3	**1.62**	6.17E-20
ERRFI1	ERBB receptor feedback inhibitor 1 [Source:HGNC Symbol;Acc:HGNC:18185]	17803.9	29284.7	**1.62**	1.01E-19
DCUN1D3	defective in cullin neddylation 1 domain containing 3 [Source:HGNC Symbol;Acc:HGNC:28734]	569.4	929.3	**1.62**	1.40E-17
THRB	thyroid hormone receptor beta [Source:HGNC Symbol;Acc:HGNC:11799]	1157.8	1849.0	**1.62**	3.61E-17
NADK	NAD kinase [Source:HGNC Symbol;Acc:HGNC:29831]	343.8	567.8	**1.62**	1.51E-15
PIK3IP1	phosphoinositide-3-kinase interacting protein 1 [Source:HGNC Symbol;Acc:HGNC:24942]	288.6	482.5	**1.62**	2.40E-13
EFNA1	ephrin A1 [Source:HGNC Symbol;Acc:HGNC:3221]	145.1	237.6	**1.62**	1.70E-10
MTMR7	myotubularin related protein 7 [Source:HGNC Symbol;Acc:HGNC:7454]	86.2	140.9	**1.62**	2.26E-08
SHROOM1	shroom family member 1 [Source:HGNC Symbol;Acc:HGNC:24084]	73.3	122.4	**1.62**	1.61E-06

ECHDC3	enoyl-CoA hydratase domain containing 3 [Source:HGNC Symbol;Acc:HGNC:23489]	101.2	168.1	**1.62**	2.58E-06
TRABD2A	TraB domain containing 2A [Source:HGNC Symbol;Acc:HGNC:27013]	73.1	120.0	**1.62**	5.82E-06
RBPMS2	RNA binding protein, mRNA processing factor 2 [Source:HGNC Symbol;Acc:HGNC:19098]	50.2	86.4	**1.62**	0.002251
ANXA1	annexin A1 [Source:HGNC Symbol;Acc:HGNC:533]	25751.0	41552.7	**1.61**	1.47E-52
SGMS2	sphingomyelin synthase 2 [Source:HGNC Symbol;Acc:HGNC:28395]	3946.8	6444.8	**1.61**	1.42E-32
BAG3	BCL2 associated athanogene 3 [Source:HGNC Symbol;Acc:HGNC:939]	909.8	1479.3	**1.61**	2.50E-23
DGKH	diacylglycerol kinase eta [Source:HGNC Symbol;Acc:HGNC:2854]	1131.3	1848.6	**1.61**	2.50E-23
CCDC152	coiled-coil domain containing 152 [Source:HGNC Symbol;Acc:HGNC:34438]	612.7	996.8	**1.61**	6.95E-17
SOWAHC	sosondowah ankyrin repeat domain family member C [Source:HGNC Symbol;Acc:HGNC:26149]	833.3	1355.3	**1.61**	1.75E-14
ARNTL	aryl hydrocarbon receptor nuclear translocator like [Source:HGNC Symbol;Acc:HGNC:701]	443.4	692.4	**1.61**	6.63E-11
SHTN1	shootin 1 [Source:HGNC Symbol;Acc:HGNC:29319]	244.7	404.4	**1.61**	4.76E-09
ZDHHC23	zinc finger DHHC-type containing 23 [Source:HGNC Symbol;Acc:HGNC:28654]	157.6	259.9	**1.61**	8.90E-09
NALCN	sodium leak channel, non-selective [Source:HGNC Symbol;Acc:HGNC:19082]	25.1	41.7	**1.61**	0.005761
LIPH	lipase H [Source:HGNC Symbol;Acc:HGNC:18483]	32.0	54.0	**1.61**	0.006888
ARHGAP6	Rho GTPase activating protein 6 [Source:HGNC Symbol;Acc:HGNC:676]	14.6	25.2	**1.61**	0.02179
SLC30A2	solute carrier family 30 member 2 [Source:HGNC Symbol;Acc:HGNC:11013]	17.7	31.4	**1.61**	0.03018
KLB	klotho beta [Source:HGNC Symbol;Acc:HGNC:15527]	19.9	34.5	**1.61**	0.04439
SUN2	Sad1 and UNC84 domain containing 2 [Source:HGNC Symbol;Acc:HGNC:14210]	1752.2	2829.5	**1.60**	1.83E-45
PRKD1	protein kinase D1 [Source:HGNC Symbol;Acc:HGNC:9407]	484.5	773.8	**1.60**	2.28E-36
MDM2	MDM2 proto-oncogene [Source:HGNC Symbol;Acc:HGNC:6973]	6658.9	10710.0	**1.60**	1.15E-29
IGF1R	insulin like growth factor 1 receptor [Source:HGNC Symbol;Acc:HGNC:5465]	715.2	1149.4	**1.60**	2.45E-29
MYC	MYC proto-oncogene, bHLH transcription factor [Source:HGNC Symbol;Acc:HGNC:7553]	502.2	802.3	**1.60**	4.28E-29
CPD	carboxypeptidase D [Source:HGNC Symbol;Acc:HGNC:2301]	6592.1	10638.6	**1.60**	9.03E-27
ARID5B	AT-rich interaction domain 5B [Source:HGNC Symbol;Acc:HGNC:17362]	1243.3	1982.4	**1.60**	1.81E-25
LHFPL2	LHFPL tetraspan subfamily member 2 [Source:HGNC Symbol;Acc:HGNC:6588]	4575.0	7347.5	**1.60**	6.60E-25
ACSS2	acyl-CoA synthetase short chain family member 2 [Source:HGNC Symbol;Acc:HGNC:15814]	257.8	413.7	**1.60**	1.41E-18
ZNF699	zinc finger protein 699 [Source:HGNC Symbol;Acc:HGNC:24750]	306.9	495.7	**1.60**	5.74E-18
KLF5	Kruppel like factor 5 [Source:HGNC Symbol;Acc:HGNC:6349]	214.8	348.1	**1.60**	5.05E-11
SEMA4B	semaphorin 4B [Source:HGNC Symbol;Acc:HGNC:10730]	105.8	171.3	**1.60**	8.44E-09
ANPEP	alanyl aminopeptidase, membrane [Source:HGNC Symbol;Acc:HGNC:500]	154.3	237.0	**1.60**	4.88E-06
ESYT3	extended synaptotagmin 3 [Source:HGNC Symbol;Acc:HGNC:24295]	68.5	112.8	**1.60**	1.71E-05
FOXL1	forkhead box L1 [Source:HGNC Symbol;Acc:HGNC:3817]	46.8	75.9	**1.60**	0.0002478
MIR635	microRNA 635 [Source:HGNC Symbol;Acc:HGNC:32891]	19.3	32.9	**1.60**	0.01593
NTF3	neurotrophin 3 [Source:HGNC Symbol;Acc:HGNC:8023]	12.6	21.1	**1.60**	0.03984
PNPLA8	patatin like phospholipase domain containing 8 [Source:HGNC Symbol;Acc:HGNC:28900]	1831.9	2921.4	**1.59**	2.14E-62
TMED5	transmembrane p24 trafficking protein 5 [Source:HGNC Symbol;Acc:HGNC:24251]	1440.9	2294.3	**1.59**	3.69E-43
RNF141	ring finger protein 141 [Source:HGNC Symbol;Acc:HGNC:21159]	1180.0	1878.2	**1.59**	2.15E-42
ITSN1	intersectin 1 [Source:HGNC Symbol;Acc:HGNC:6183]	1496.3	2377.6	**1.59**	1.50E-41
SMARCD2	SWI/SNF related, matrix associated, actin dependent regulator of chromatin, subfamily d, member 2 [Sc	715.8	1147.6	**1.59**	2.49E-25
ATP9A	ATPase phospholipid transporting 9A (putative) [Source:HGNC Symbol;Acc:HGNC:13540]	1311.7	2125.1	**1.59**	2.83E-21
CEBPB	CCAAT enhancer binding protein beta [Source:HGNC Symbol;Acc:HGNC:1834]	728.2	1157.3	**1.59**	7.62E-20
GRK5	G protein-coupled receptor kinase 5 [Source:HGNC Symbol;Acc:HGNC:4544]	638.1	1036.1	**1.59**	6.35E-19
SLC1A1	solute carrier family 1 member 1 [Source:HGNC Symbol;Acc:HGNC:10939]	2167.1	3445.2	**1.59**	5.14E-16
TGFBR3	transforming growth factor beta receptor 3 [Source:HGNC Symbol;Acc:HGNC:11774]	2621.6	4297.5	**1.59**	5.60E-16
SHE	Src homology 2 domain containing E [Source:HGNC Symbol;Acc:HGNC:27004]	141.8	224.1	**1.59**	1.98E-09
GRIP1	glutamate receptor interacting protein 1 [Source:HGNC Symbol;Acc:HGNC:18708]	66.4	108.3	**1.59**	3.26E-06
SAMHD1	SAM and HD domain containing deoxynucleoside triphosphate triphosphohydrolase 1 [Source:HGNC Sy	170.5	292.3	**1.59**	9.15E-06
ODF3B	outer dense fiber of sperm tails 3B [Source:HGNC Symbol;Acc:HGNC:34388]	27.9	46.3	**1.59**	0.003665
LINC01004	long intergenic non-protein coding RNA 1004 [Source:HGNC Symbol;Acc:HGNC:48961]	28.6	46.8	**1.59**	0.003914
ANKRD13B	ankyrin repeat domain 13B [Source:HGNC Symbol;Acc:HGNC:26363]	36.4	59.3	**1.59**	0.00451
MBL1P	mannose binding lectin 1, pseudogene [Source:HGNC Symbol;Acc:HGNC:6921]	30.6	50.2	**1.59**	0.005153
ELL2P1	elongation factor for RNA polymerase II 2 pseudogene 1 [Source:HGNC Symbol;Acc:HGNC:39343]	25.7	41.6	**1.59**	0.01376
PROSER2-AS1	PROSER2 antisense RNA 1 [Source:HGNC Symbol;Acc:HGNC:27343]	15.8	26.8	**1.59**	0.02574
LRRIQ1	leucine rich repeats and IQ motif containing 1 [Source:HGNC Symbol;Acc:HGNC:25708]	13.8	23.5	**1.59**	0.04107
RSRP1	arginine and serine rich protein 1 [Source:HGNC Symbol;Acc:HGNC:25234]	877.1	1389.2	**1.58**	1.24E-40
METTL5	methyltransferase like 5 [Source:HGNC Symbol;Acc:HGNC:25006]	1905.0	3022.8	**1.58**	1.95E-35
OSBPL1A	oxysterol binding protein like 1A [Source:HGNC Symbol;Acc:HGNC:16398]	1040.5	1650.8	**1.58**	9.03E-31
PHKA1	phosphorylase kinase regulatory subunit alpha 1 [Source:HGNC Symbol;Acc:HGNC:8925]	434.6	694.5	**1.58**	6.51E-21
CAPG	capping actin protein, gelsolin like [Source:HGNC Symbol;Acc:HGNC:1474]	1454.8	2300.2	**1.58**	1.63E-19
TNFAIP8	TNF alpha induced protein 8 [Source:HGNC Symbol;Acc:HGNC:17260]	282.7	454.3	**1.58**	6.74E-15
GEMIN5	gem nuclear organelle associated protein 5 [Source:HGNC Symbol;Acc:HGNC:20043]	775.0	1244.8	**1.58**	7.28E-14
CRTC3	CREB regulated transcription coactivator 3 [Source:HGNC Symbol;Acc:HGNC:26148]	250.1	398.1	**1.58**	8.16E-13
CCDC107	coiled-coil domain containing 107 [Source:HGNC Symbol;Acc:HGNC:28465]	251.2	405.7	**1.58**	1.38E-11
YPEL3	yippee like 3 [Source:HGNC Symbol;Acc:HGNC:18327]	121.5	194.4	**1.58**	1.55E-08
TMEM159	transmembrane protein 159 [Source:HGNC Symbol;Acc:HGNC:30136]	148.7	236.8	**1.58**	4.44E-08
IL18R1	interleukin 18 receptor 1 [Source:HGNC Symbol;Acc:HGNC:5988]	64.2	103.2	**1.58**	7.30E-06
GRTP1	growth hormone regulated TBC protein 1 [Source:HGNC Symbol;Acc:HGNC:20310]	33.1	55.3	**1.58**	0.0009445
MCPH1-AS1	MCPH1 antisense RNA 1 [Source:HGNC Symbol;Acc:HGNC:51655]	37.6	62.6	**1.58**	0.002336
MYOSLID	myocardin-induced smooth muscle lncRNA, inducer of differentiation [Source:HGNC Symbol;Acc:HGNC:	25.1	40.9	**1.58**	0.009962
CACNB2	calcium voltage-gated channel auxiliary subunit beta 2 [Source:HGNC Symbol;Acc:HGNC:1402]	18.2	29.9	**1.58**	0.01861
DYNLT3	dynein light chain Tctex-type 3 [Source:HGNC Symbol;Acc:HGNC:11694]	2763.9	4348.0	**1.57**	5.32E-40
SSB	Sjogren syndrome antigen B [Source:HGNC Symbol;Acc:HGNC:11316]	5829.5	9229.0	**1.57**	2.29E-38

STK39	serine/threonine kinase 39 [Source:HGNC Symbol;Acc:HGNC:17717]	2269.7	3587.3	**1.57**	2.27E-36
RBMXL1	RBMX like 1 [Source:HGNC Symbol;Acc:HGNC:25073]	544.2	853.3	**1.57**	3.70E-29
SLC16A7	solute carrier family 16 member 7 [Source:HGNC Symbol;Acc:HGNC:10928]	5341.7	8438.0	**1.57**	2.76E-28
H2AFJ	H2A histone family member J [Source:HGNC Symbol;Acc:HGNC:14456]	705.3	1121.0	**1.57**	1.37E-27
FAM126A	family with sequence similarity 126 member A [Source:HGNC Symbol;Acc:HGNC:24587]	1447.6	2290.2	**1.57**	3.10E-26
ZC3H12C	zinc finger CCCH-type containing 12C [Source:HGNC Symbol;Acc:HGNC:29362]	661.7	1041.7	**1.57**	1.08E-25
CD58	CD58 molecule [Source:HGNC Symbol;Acc:HGNC:1688]	444.8	702.6	**1.57**	3.74E-21
RYBP	RING1 and YY1 binding protein [Source:HGNC Symbol;Acc:HGNC:10480]	785.7	1259.9	**1.57**	1.51E-15
PPTC7	PTC7 protein phosphatase homolog [Source:HGNC Symbol;Acc:HGNC:30695]	185.2	293.4	**1.57**	3.14E-14
NEK3	NIMA related kinase 3 [Source:HGNC Symbol;Acc:HGNC:7746]	201.5	316.6	**1.57**	6.39E-14
STRADB	STE20-related kinase adaptor beta [Source:HGNC Symbol;Acc:HGNC:13205]	204.9	324.1	**1.57**	1.49E-12
ABLIM1	actin binding LIM protein 1 [Source:HGNC Symbol;Acc:HGNC:78]	406.5	650.9	**1.57**	2.26E-12
AHCYL2	adenosylhomocysteinase like 2 [Source:HGNC Symbol;Acc:HGNC:22204]	449.6	720.6	**1.57**	8.41E-12
NBPF19	NBPF member 19 [Source:HGNC Symbol;Acc:HGNC:31999]	174.2	279.0	**1.57**	2.00E-10
EML6	echinoderm microtubule associated protein like 6 [Source:HGNC Symbol;Acc:HGNC:35412]	111.9	179.5	**1.57**	3.57E-08
CYTL1	cytokine like 1 [Source:HGNC Symbol;Acc:HGNC:24435]	3064.0	5053.7	**1.57**	3.45E-07
C19orf44	chromosome 19 open reading frame 44 [Source:HGNC Symbol;Acc:HGNC:26141]	110.7	173.6	**1.57**	4.62E-06
TPT1P9	tumor protein, translationally-controlled 1 pseudogene 9 [Source:HGNC Symbol;Acc:HGNC:49299]	47.4	75.4	**1.57**	2.54E-05
MIDN	midnolin [Source:HGNC Symbol;Acc:HGNC:16298]	130.6	204.9	**1.57**	0.0001004
NUS1P1	NUS1 pseudogene 1 [Source:HGNC Symbol;Acc:HGNC:38472]	16.5	27.8	**1.57**	0.03956
DENND4C	DENN domain containing 4C [Source:HGNC Symbol;Acc:HGNC:26079]	1685.5	2631.1	**1.56**	2.72E-45
COL6A3	collagen type VI alpha 3 chain [Source:HGNC Symbol;Acc:HGNC:2213]	77705.1	122455.3	**1.56**	1.20E-40
FERMT2	fermitin family member 2 [Source:HGNC Symbol;Acc:HGNC:15767]	4549.7	7158.8	**1.56**	1.08E-29
CAV2	caveolin 2 [Source:HGNC Symbol;Acc:HGNC:1528]	1512.5	2380.9	**1.56**	2.69E-19
ORAI3	ORAI calcium release-activated calcium modulator 3 [Source:HGNC Symbol;Acc:HGNC:28185]	139.3	221.3	**1.56**	6.45E-11
ARHGAP18	Rho GTPase activating protein 18 [Source:HGNC Symbol;Acc:HGNC:21035]	273.4	435.0	**1.56**	2.09E-08
COL4A1	collagen type IV alpha 1 chain [Source:HGNC Symbol;Acc:HGNC:2202]	194.8	304.1	**1.56**	5.61E-08
SEMA5A	semaphorin 5A [Source:HGNC Symbol;Acc:HGNC:10736]	109.5	169.2	**1.56**	3.03E-07
MSRB1	methionine sulfoxide reductase B1 [Source:HGNC Symbol;Acc:HGNC:14133]	87.1	137.1	**1.56**	4.95E-06
GLIPR2	GLI pathogenesis related 2 [Source:HGNC Symbol;Acc:HGNC:18007]	42.5	69.1	**1.56**	0.001258
LINC01290	long intergenic non-protein coding RNA 1290 [Source:HGNC Symbol;Acc:HGNC:50356]	12.9	21.4	**1.56**	0.04497
CAST	calpastatin [Source:HGNC Symbol;Acc:HGNC:1515]	4898.4	7584.6	**1.55**	1.08E-49
EIF4EBP2	eukaryotic translation initiation factor 4E binding protein 2 [Source:HGNC Symbol;Acc:HGNC:3289]	2371.7	3700.4	**1.55**	9.39E-41
MAVS	mitochondrial antiviral signaling protein [Source:HGNC Symbol;Acc:HGNC:29233]	1464.0	2268.9	**1.55**	4.83E-40
RGS10	regulator of G protein signaling 10 [Source:HGNC Symbol;Acc:HGNC:9992]	512.8	799.3	**1.55**	5.45E-24
TAF8	TATA-box binding protein associated factor 8 [Source:HGNC Symbol;Acc:HGNC:17300]	443.8	688.3	**1.55**	7.16E-22
POLR3GL	RNA polymerase III subunit G like [Source:HGNC Symbol;Acc:HGNC:28466]	424.6	657.3	**1.55**	4.46E-21
CEP126	centrosomal protein 126 [Source:HGNC Symbol;Acc:HGNC:29264]	521.9	810.6	**1.55**	4.48E-19
NEDD4	neural precursor cell expressed, developmentally down-regulated 4, E3 ubiquitin protein ligase [Source:	1567.3	2474.6	**1.55**	7.58E-19
UBE2D1	ubiquitin conjugating enzyme E2 D1 [Source:HGNC Symbol;Acc:HGNC:12474]	302.3	471.2	**1.55**	1.75E-17
COL18A1	collagen type XVIII alpha 1 chain [Source:HGNC Symbol;Acc:HGNC:2195]	68.9	109.4	**1.55**	9.15E-06
NAPRT	nicotinate phosphoribosyltransferase [Source:HGNC Symbol;Acc:HGNC:30450]	32.7	50.4	**1.55**	0.002363
C1QTNF1	C1q and TNF related 1 [Source:HGNC Symbol;Acc:HGNC:14324]	49.3	76.1	**1.55**	0.002445
NXPE1	neurexophilin and PC-esterase domain family member 1 [Source:HGNC Symbol;Acc:HGNC:28527]	33.7	52.6	**1.55**	0.006865
MUC20	mucin 20, cell surface associated [Source:HGNC Symbol;Acc:HGNC:23282]	14.9	23.3	**1.55**	0.04016
FADS1	fatty acid desaturase 1 [Source:HGNC Symbol;Acc:HGNC:3574]	2366.0	3671.8	**1.54**	6.71E-22
TP53BP2	tumor protein p53 binding protein 2 [Source:HGNC Symbol;Acc:HGNC:12000]	587.5	915.1	**1.54**	3.31E-19
INPP5K	inositol polyphosphate-5-phosphatase K [Source:HGNC Symbol;Acc:HGNC:33882]	318.6	494.1	**1.54**	1.69E-17
HOMEZ	homeobox and leucine zipper encoding [Source:HGNC Symbol;Acc:HGNC:20164]	158.1	244.9	**1.54**	3.17E-12
SNN	stannin [Source:HGNC Symbol;Acc:HGNC:11149]	183.3	288.0	**1.54**	1.38E-09
CCDC65	coiled-coil domain containing 65 [Source:HGNC Symbol;Acc:HGNC:29937]	31.4	48.7	**1.54**	0.002438
GALNT9	polypeptide N-acetylgalactosaminyltransferase 9 [Source:HGNC Symbol;Acc:HGNC:4131]	26.9	42.7	**1.54**	0.01185
MASP2	mannan binding lectin serine peptidase 2 [Source:HGNC Symbol;Acc:HGNC:6902]	17.6	27.8	**1.54**	0.0245
FUT8-AS1	FUT8 antisense RNA 1 [Source:HGNC Symbol;Acc:HGNC:44294]	17.5	27.7	**1.54**	0.02884
MRVI1	murine retrovirus integration site 1 homolog [Source:HGNC Symbol;Acc:HGNC:7237]	20.2	33.4	**1.54**	0.03796
KANSL1L	KAT8 regulatory NSL complex subunit 1 like [Source:HGNC Symbol;Acc:HGNC:26310]	1266.6	1937.3	**1.53**	2.05E-39
AFF1	AF4/FMR2 family member 1 [Source:HGNC Symbol;Acc:HGNC:7135]	1659.8	2546.9	**1.53**	9.82E-37
SDCBP	syndecan binding protein [Source:HGNC Symbol;Acc:HGNC:10662]	2396.9	3678.4	**1.53**	5.07E-36
PNRC1	proline rich nuclear receptor coactivator 1 [Source:HGNC Symbol;Acc:HGNC:17278]	1239.6	1907.0	**1.53**	4.64E-33
PTPDC1	protein tyrosine phosphatase domain containing 1 [Source:HGNC Symbol;Acc:HGNC:30184]	654.3	1002.5	**1.53**	2.16E-30
SPIRE1	spire type actin nucleation factor 1 [Source:HGNC Symbol;Acc:HGNC:30622]	1806.2	2785.4	**1.53**	5.90E-26
SOAT1	sterol O-acyltransferase 1 [Source:HGNC Symbol;Acc:HGNC:11177]	1515.2	2365.4	**1.53**	1.06E-21
ST6GAL1	ST6 beta-galactoside alpha-2,6-sialyltransferase 1 [Source:HGNC Symbol;Acc:HGNC:10860]	1181.1	1815.5	**1.53**	7.48E-18
BCL6	B cell CLL/lymphoma 6 [Source:HGNC Symbol;Acc:HGNC:1001]	1243.7	1923.6	**1.53**	7.71E-18
ZDHHC7	zinc finger DHHC-type containing 7 [Source:HGNC Symbol;Acc:HGNC:18459]	743.1	1138.7	**1.53**	1.57E-16
KLF12	Kruppel like factor 12 [Source:HGNC Symbol;Acc:HGNC:6346]	565.7	867.1	**1.53**	1.08E-14
RAB11FIP3	RAB11 family interacting protein 3 [Source:HGNC Symbol;Acc:HGNC:17224]	303.5	464.0	**1.53**	1.47E-14
A4GALT	alpha 1,4-galactosyltransferase (P blood group) [Source:HGNC Symbol;Acc:HGNC:18149]	203.5	310.7	**1.53**	2.70E-14
PPP1R3C	protein phosphatase 1 regulatory subunit 3C [Source:HGNC Symbol;Acc:HGNC:9293]	12594.1	18830.9	**1.53**	3.07E-14
SACS	sacsin molecular chaperone [Source:HGNC Symbol;Acc:HGNC:10519]	2919.0	4551.8	**1.53**	1.10E-11
SERPINB1	serpin family B member 1 [Source:HGNC Symbol;Acc:HGNC:3311]	1344.4	2070.5	**1.53**	3.25E-11
PLA2G16	phospholipase A2 group XVI [Source:HGNC Symbol;Acc:HGNC:17825]	227.8	360.2	**1.53**	7.43E-10

UBE2E2	ubiquitin conjugating enzyme E2 E2 [Source:HGNC Symbol;Acc:HGNC:12478]	244.2	375.2	**1.53**	3.73E-08
ARHGAP27P1-BPT	ARHGAP27P1-BPTFP1-KPNA2P3 readthrough, transcribed pseudogene [Source:HGNC Symbol;Acc:HGNC	134.0	203.2	**1.53**	1.36E-05
SLC7A5P2	solute carrier family 7 member 5 pseudogene 2 [Source:HGNC Symbol;Acc:HGNC:24951]	32.6	51.0	**1.53**	0.00424
FOXO3B	forkhead box O3B pseudogene [Source:HGNC Symbol;Acc:HGNC:3822]	24.7	38.7	**1.53**	0.005703
CASTOR2	cytosolic arginine sensor for mTORC1 subunit 2 [Source:HGNC Symbol;Acc:HGNC:37073]	27.5	43.7	**1.53**	0.03563
MBNL1	muscleblind like splicing regulator 1 [Source:HGNC Symbol;Acc:HGNC:6923]	12345.7	18704.9	**1.52**	7.70E-42
MTMR2	myotubularin related protein 2 [Source:HGNC Symbol;Acc:HGNC:7450]	2265.8	3446.1	**1.52**	2.30E-38
MET	MET proto-oncogene, receptor tyrosine kinase [Source:HGNC Symbol;Acc:HGNC:7029]	5495.6	8430.2	**1.52**	3.06E-22
CCNYL1	cyclin Y like 1 [Source:HGNC Symbol;Acc:HGNC:26868]	369.0	563.8	**1.52**	6.21E-21
CORO1B	coronin 1B [Source:HGNC Symbol;Acc:HGNC:2253]	361.6	548.4	**1.52**	1.19E-17
PLEKHM2	pleckstrin homology and RUN domain containing M2 [Source:HGNC Symbol;Acc:HGNC:29131]	548.8	836.5	**1.52**	1.43E-16
NANS	N-acetylneuraminate synthase [Source:HGNC Symbol;Acc:HGNC:19237]	1927.0	2908.2	**1.52**	1.85E-16
CSTB	cystatin B [Source:HGNC Symbol;Acc:HGNC:2482]	418.8	634.0	**1.52**	5.66E-11
SH3PXD2B	SH3 and PX domains 2B [Source:HGNC Symbol;Acc:HGNC:29242]	254.2	390.5	**1.52**	7.72E-11
TLNRD1	talin rod domain containing 1 [Source:HGNC Symbol;Acc:HGNC:13519]	97.5	151.7	**1.52**	0.0001542
TNFAIP3	TNF alpha induced protein 3 [Source:HGNC Symbol;Acc:HGNC:11896]	88.8	142.3	**1.52**	0.001049
TTC9	tetratricopeptide repeat domain 9 [Source:HGNC Symbol;Acc:HGNC:20267]	81.0	126.3	**1.52**	0.002569
SMG1P5	SMG1 pseudogene 5 [Source:HGNC Symbol;Acc:HGNC:49862]	31.3	48.1	**1.52**	0.003908
CHD5	chromodomain helicase DNA binding protein 5 [Source:HGNC Symbol;Acc:HGNC:16816]	27.6	43.1	**1.52**	0.005311
LAMC3	laminin subunit gamma 3 [Source:HGNC Symbol;Acc:HGNC:6494]	35.3	55.2	**1.52**	0.01161
MBD2	methyl-CpG binding domain protein 2 [Source:HGNC Symbol;Acc:HGNC:6917]	2797.6	4203.8	**1.51**	3.41E-50
MKLN1	muskelin 1 [Source:HGNC Symbol;Acc:HGNC:7109]	2811.8	4238.1	**1.51**	3.25E-49
PPA1	pyrophosphatase (inorganic) 1 [Source:HGNC Symbol;Acc:HGNC:9226]	2871.8	4322.7	**1.51**	1.32E-29
TNFRSF1A	TNF receptor superfamily member 1A [Source:HGNC Symbol;Acc:HGNC:11916]	1782.9	2685.8	**1.51**	2.56E-29
ARNT	aryl hydrocarbon receptor nuclear translocator [Source:HGNC Symbol;Acc:HGNC:700]	967.5	1452.8	**1.51**	1.45E-27
UQCRC1	ubiquinol-cytochrome c reductase core protein 1 [Source:HGNC Symbol;Acc:HGNC:12585]	1129.5	1713.9	**1.51**	2.77E-27
SLC30A4	solute carrier family 30 member 4 [Source:HGNC Symbol;Acc:HGNC:11015]	939.6	1423.0	**1.51**	4.19E-19
CASP4	caspase 4 [Source:HGNC Symbol;Acc:HGNC:1505]	751.6	1120.7	**1.51**	9.54E-19
GCNT1	glucosaminyl (N-acetyl) transferase 1, core 2 [Source:HGNC Symbol;Acc:HGNC:4203]	2720.7	4068.9	**1.51**	1.16E-18
SLC44A1	solute carrier family 44 member 1 [Source:HGNC Symbol;Acc:HGNC:18798]	1105.1	1678.6	**1.51**	3.06E-18
RBP4	retinol binding protein 4 [Source:HGNC Symbol;Acc:HGNC:9922]	447.2	664.0	**1.51**	9.11E-16
TANC2	tetratricopeptide repeat, ankyrin repeat and coiled-coil containing 2 [Source:HGNC Symbol;Acc:HGNC:3	243.1	368.5	**1.51**	2.07E-10
CPM	carboxypeptidase M [Source:HGNC Symbol;Acc:HGNC:2311]	749.2	1146.6	**1.51**	4.27E-10
NCR3LG1	natural killer cell cytotoxicity receptor 3 ligand 1 [Source:HGNC Symbol;Acc:HGNC:42400]	174.4	264.7	**1.51**	6.77E-10
SLC6A8	solute carrier family 6 member 8 [Source:HGNC Symbol;Acc:HGNC:11055]	195.3	293.0	**1.51**	5.67E-09
HMGB3	high mobility group box 3 [Source:HGNC Symbol;Acc:HGNC:5004]	500.1	762.5	**1.51**	2.66E-08
FBXO17	F-box protein 17 [Source:HGNC Symbol;Acc:HGNC:18754]	175.8	263.6	**1.51**	9.17E-07
IGBP1-AS2	IGBP1 antisense RNA 2 [Source:HGNC Symbol;Acc:HGNC:40294]	110.1	167.1	**1.51**	3.22E-06
SLC38A7	solute carrier family 38 member 7 [Source:HGNC Symbol;Acc:HGNC:25582]	116.7	180.1	**1.51**	8.99E-06
POPDC3	popeye domain containing 3 [Source:HGNC Symbol;Acc:HGNC:17649]	117.6	181.5	**1.51**	0.0001002
TMEM63C	transmembrane protein 63C [Source:HGNC Symbol;Acc:HGNC:23787]	42.1	65.5	**1.51**	0.005838
C3	complement C3 [Source:HGNC Symbol;Acc:HGNC:1318]	23.8	38.1	**1.51**	0.0234
HHIP-AS1	HHIP antisense RNA 1 [Source:HGNC Symbol;Acc:HGNC:44182]	39.9	62.3	**1.51**	0.03419
ATP6V0E1	ATPase H+ transporting V0 subunit e1 [Source:HGNC Symbol;Acc:HGNC:863]	1918.3	2880.7	**1.49**	1.75E-52
C1orf21	chromosome 1 open reading frame 21 [Source:HGNC Symbol;Acc:HGNC:15494]	1972.7	2974.4	**1.49**	8.21E-34
PTPN3	protein tyrosine phosphatase, non-receptor type 3 [Source:HGNC Symbol;Acc:HGNC:9655]	568.4	849.9	**1.49**	2.21E-28
EPAS1	endothelial PAS domain protein 1 [Source:HGNC Symbol;Acc:HGNC:3374]	2963.3	4486.6	**1.49**	2.11E-22
APBB2	amyloid beta precursor protein binding family B member 2 [Source:HGNC Symbol;Acc:HGNC:582]	1456.4	2161.4	**1.49**	8.39E-18
NECAB1	N-terminal EF-hand calcium binding protein 1 [Source:HGNC Symbol;Acc:HGNC:20983]	397.0	598.8	**1.49**	9.81E-18
CITED2	Cbp/p300 interacting transactivator with Glu/Asp rich carboxy-terminal domain 2 [Source:HGNC Symbo	1241.8	1921.4	**1.49**	9.72E-13
ARHGAP42	Rho GTPase activating protein 42 [Source:HGNC Symbol;Acc:HGNC:26545]	821.7	1252.9	**1.49**	4.34E-12
FBXO31	F-box protein 31 [Source:HGNC Symbol;Acc:HGNC:16510]	349.1	531.0	**1.49**	7.57E-11
EPB41L5	erythrocyte membrane protein band 4.1 like 5 [Source:HGNC Symbol;Acc:HGNC:19819]	362.4	537.8	**1.49**	1.55E-09
TLR4	toll like receptor 4 [Source:HGNC Symbol;Acc:HGNC:11850]	218.9	323.8	**1.49**	6.35E-09
ACVR1B	activin A receptor type 1B [Source:HGNC Symbol;Acc:HGNC:172]	191.3	289.3	**1.49**	1.08E-08
CYP27A1	cytochrome P450 family 27 subfamily A member 1 [Source:HGNC Symbol;Acc:HGNC:2605]	285.1	434.2	**1.49**	3.37E-08
EBPL	emopamil binding protein like [Source:HGNC Symbol;Acc:HGNC:18061]	201.9	304.8	**1.49**	3.39E-08
PABPC5	poly(A) binding protein cytoplasmic 5 [Source:HGNC Symbol;Acc:HGNC:13629]	145.6	218.9	**1.49**	7.98E-07
FAM222B	family with sequence similarity 222 member B [Source:HGNC Symbol;Acc:HGNC:25563]	91.4	137.8	**1.49**	1.11E-06
NUDT16P1	nudix hydrolase 16 pseudogene 1 [Source:HGNC Symbol;Acc:HGNC:27189]	84.8	126.9	**1.49**	1.44E-05
C15orf65	chromosome 15 open reading frame 65 [Source:HGNC Symbol;Acc:HGNC:44654]	67.4	101.8	**1.49**	1.87E-05
KIAA0408	KIAA0408 [Source:HGNC Symbol;Acc:HGNC:21636]	119.0	182.0	**1.49**	5.38E-05
SMPD2	sphingomyelin phosphodiesterase 2 [Source:HGNC Symbol;Acc:HGNC:11121]	63.5	95.8	**1.49**	0.0001248
FOXC2	forkhead box C2 [Source:HGNC Symbol;Acc:HGNC:3801]	102.2	159.3	**1.49**	0.000713
ARFGEF2	ADP ribosylation factor guanine nucleotide exchange factor 2 [Source:HGNC Symbol;Acc:HGNC:15853]	5229.2	7800.0	**1.48**	1.88E-39
ITGA1	integrin subunit alpha 1 [Source:HGNC Symbol;Acc:HGNC:6134]	2478.7	3680.7	**1.48**	1.24E-32
NOTCH2	notch 2 [Source:HGNC Symbol;Acc:HGNC:7882]	10986.4	16345.1	**1.48**	3.71E-30
AKT3	AKT serine/threonine kinase 3 [Source:HGNC Symbol;Acc:HGNC:393]	6747.6	10077.0	**1.48**	4.44E-28
STAT2	signal transducer and activator of transcription 2 [Source:HGNC Symbol;Acc:HGNC:11363]	1042.0	1560.2	**1.48**	1.90E-26
YWHAQ	tyrosine 3-monooxygenase/tryptophan 5-monooxygenase activation protein theta [Source:HGNC Symb	7392.4	10997.7	**1.48**	3.32E-26
CLASP1	cytoplasmic linker associated protein 1 [Source:HGNC Symbol;Acc:HGNC:17088]	1456.0	2175.7	**1.48**	8.86E-25
OPTN	optineurin [Source:HGNC Symbol;Acc:HGNC:17142]	3221.6	4816.4	**1.48**	1.31E-22

MAT2A	methionine adenosyltransferase 2A [Source:HGNC Symbol;Acc:HGNC:6904]	3691.4	5522.9	**1.48**	1.31E-22
RCL1	RNA terminal phosphate cyclase like 1 [Source:HGNC Symbol;Acc:HGNC:17687]	852.1	1274.3	**1.48**	3.20E-21
CARMIL1	capping protein regulator and myosin 1 linker 1 [Source:HGNC Symbol;Acc:HGNC:21581]	1272.5	1891.3	**1.48**	2.04E-15
TUBA1A	tubulin alpha 1a [Source:HGNC Symbol;Acc:HGNC:20766]	1536.8	2273.6	**1.48**	1.64E-11
CAPN1	calpain 1 [Source:HGNC Symbol;Acc:HGNC:1476]	340.8	503.9	**1.48**	2.19E-10
ARHGAP24	Rho GTPase activating protein 24 [Source:HGNC Symbol;Acc:HGNC:25361]	155.0	234.2	**1.48**	1.48E-09
HSD11B1	hydroxysteroid 11-beta dehydrogenase 1 [Source:HGNC Symbol;Acc:HGNC:5208]	481.3	735.8	**1.48**	2.85E-09
TMEM120A	transmembrane protein 120A [Source:HGNC Symbol;Acc:HGNC:21697]	180.5	269.7	**1.48**	1.24E-07
AHNAK	AHNAK nucleoprotein [Source:HGNC Symbol;Acc:HGNC:347]	69048.6	102610.7	**1.48**	6.53E-06
PXMP2	peroxisomal membrane protein 2 [Source:HGNC Symbol;Acc:HGNC:9716]	57.9	86.9	**1.48**	0.000217
VAMP2	vesicle associated membrane protein 2 [Source:HGNC Symbol;Acc:HGNC:12643]	63.6	96.3	**1.48**	0.0003948
SMIM29	small integral membrane protein 29 [Source:HGNC Symbol;Acc:HGNC:1340]	70.7	106.3	**1.48**	0.001939
EEPD1	endonuclease/exonuclease/phosphatase family domain containing 1 [Source:HGNC Symbol;Acc:HGNC:2	43.6	66.8	**1.48**	0.01877
LZTS3	leucine zipper tumor suppressor family member 3 [Source:HGNC Symbol;Acc:HGNC:30139]	20.0	31.0	**1.48**	0.03345
LHX4	LIM homeobox 4 [Source:HGNC Symbol;Acc:HGNC:21734]	25.8	40.6	**1.48**	0.04658
UBC	ubiquitin C [Source:HGNC Symbol;Acc:HGNC:12468]	14876.2	21905.0	**1.47**	8.79E-62
PIK3CA	phosphatidylinositol-4,5-bisphosphate 3-kinase catalytic subunit alpha [Source:HGNC Symbol;Acc:HGNC	1632.4	2411.8	**1.47**	6.32E-46
REXO2	RNA exonuclease 2 [Source:HGNC Symbol;Acc:HGNC:17851]	4691.6	6937.0	**1.47**	5.63E-45
SLK	STE20 like kinase [Source:HGNC Symbol;Acc:HGNC:11088]	5435.6	8049.2	**1.47**	3.41E-44
ARMC8	armadillo repeat containing 8 [Source:HGNC Symbol;Acc:HGNC:24999]	1128.4	1669.2	**1.47**	2.29E-33
KIF1B	kinesin family member 1B [Source:HGNC Symbol;Acc:HGNC:16636]	1565.5	2321.8	**1.47**	6.05E-30
UBTD2	ubiquitin domain containing 2 [Source:HGNC Symbol;Acc:HGNC:24463]	1667.0	2458.7	**1.47**	2.12E-28
SNAP23	synaptosome associated protein 23 [Source:HGNC Symbol;Acc:HGNC:11131]	1732.6	2573.4	**1.47**	2.33E-28
SLC33A1	solute carrier family 33 member 1 [Source:HGNC Symbol;Acc:HGNC:95]	1392.9	2058.5	**1.47**	2.62E-28
ARHGAP21	Rho GTPase activating protein 21 [Source:HGNC Symbol;Acc:HGNC:23725]	4277.8	6275.3	**1.47**	7.78E-25
CTDSP2	CTD small phosphatase 2 [Source:HGNC Symbol;Acc:HGNC:17077]	2424.5	3581.4	**1.47**	4.31E-24
AGO4	argonaute 4, RISC catalytic component [Source:HGNC Symbol;Acc:HGNC:18424]	499.5	736.6	**1.47**	5.12E-19
LPP	LIM domain containing preferred translocation partner in lipoma [Source:HGNC Symbol;Acc:HGNC:6679	4293.7	6302.4	**1.47**	1.75E-16
ENTPD6	ectonucleoside triphosphate diphosphohydrolase 6 (putative) [Source:HGNC Symbol;Acc:HGNC:3368]	1178.2	1722.6	**1.47**	2.77E-16
ZBTB7B	zinc finger and BTB domain containing 7B [Source:HGNC Symbol;Acc:HGNC:18668]	227.6	340.8	**1.47**	5.08E-09
IGF2	insulin like growth factor 2 [Source:HGNC Symbol;Acc:HGNC:5466]	307.2	460.9	**1.47**	1.32E-06
SIRT7	sirtuin 7 [Source:HGNC Symbol;Acc:HGNC:14935]	106.3	159.3	**1.47**	1.76E-05
ISCA2	iron-sulfur cluster assembly 2 [Source:HGNC Symbol;Acc:HGNC:19857]	85.5	127.1	**1.47**	8.94E-05
PAWR	pro-apoptotic WT1 regulator [Source:HGNC Symbol;Acc:HGNC:8614]	77.9	115.3	**1.47**	0.0002239
DPYD-AS1	DPYD antisense RNA 1 [Source:HGNC Symbol;Acc:HGNC:40195]	25.8	39.0	**1.47**	0.02268
MAPK14	mitogen-activated protein kinase 14 [Source:HGNC Symbol;Acc:HGNC:6876]	787.2	1152.3	**1.46**	2.29E-33
LRRFIP2	LRR binding FLII interacting protein 2 [Source:HGNC Symbol;Acc:HGNC:6703]	2594.9	3810.7	**1.46**	2.26E-32
SLC7A6OS	solute carrier family 7 member 6 opposite strand [Source:HGNC Symbol;Acc:HGNC:25807]	639.9	936.9	**1.46**	2.17E-31
ZDHHC9	zinc finger DHHC-type containing 9 [Source:HGNC Symbol;Acc:HGNC:18475]	795.6	1168.8	**1.46**	2.54E-22
ZDHHC3	zinc finger DHHC-type containing 3 [Source:HGNC Symbol;Acc:HGNC:18470]	481.7	705.1	**1.46**	1.50E-21
MAP3K8	mitogen-activated protein kinase kinase kinase 8 [Source:HGNC Symbol;Acc:HGNC:6860]	527.5	777.2	**1.46**	6.17E-20
BBOF1	basal body orientation factor 1 [Source:HGNC Symbol;Acc:HGNC:19855]	408.9	600.5	**1.46**	1.66E-16
GSTO1	glutathione S-transferase omega 1 [Source:HGNC Symbol;Acc:HGNC:13312]	1879.8	2732.8	**1.46**	3.98E-16
PPM1K	protein phosphatase, Mg2+/Mn2+ dependent 1K [Source:HGNC Symbol;Acc:HGNC:25415]	669.6	977.5	**1.46**	2.42E-15
CNN2	calponin 2 [Source:HGNC Symbol;Acc:HGNC:2156]	413.0	600.6	**1.46**	1.11E-13
PABPC4	poly(A) binding protein cytoplasmic 4 [Source:HGNC Symbol;Acc:HGNC:8557]	1799.5	2664.8	**1.46**	1.22E-13
HIVEP2	human immunodeficiency virus type I enhancer binding protein 2 [Source:HGNC Symbol;Acc:HGNC:492	1984.0	2954.9	**1.46**	4.90E-13
EHHADH	enoyl-CoA hydratase and 3-hydroxyacyl CoA dehydrogenase [Source:HGNC Symbol;Acc:HGNC:3247]	361.2	538.4	**1.46**	5.85E-12
VAMP8	vesicle associated membrane protein 8 [Source:HGNC Symbol;Acc:HGNC:12647]	254.6	371.2	**1.46**	3.63E-11
GPAT3	glycerol-3-phosphate acyltransferase 3 [Source:HGNC Symbol;Acc:HGNC:28157]	370.0	543.8	**1.46**	8.43E-11
CHKA	choline kinase alpha [Source:HGNC Symbol;Acc:HGNC:1937]	187.0	275.6	**1.46**	1.07E-09
WASF2	WAS protein family member 2 [Source:HGNC Symbol;Acc:HGNC:12733]	1109.9	1654.0	**1.46**	8.91E-09
IL17RC	interleukin 17 receptor C [Source:HGNC Symbol;Acc:HGNC:18358]	297.2	445.2	**1.46**	4.01E-08
RTN4RL1	reticulon 4 receptor like 1 [Source:HGNC Symbol;Acc:HGNC:21329]	211.1	330.1	**1.46**	4.44E-06
CTTNBP2	cortactin binding protein 2 [Source:HGNC Symbol;Acc:HGNC:15679]	91.1	141.7	**1.46**	0.001005
EIF4BP6	eukaryotic translation initiation factor 4B pseudogene 6 [Source:HGNC Symbol;Acc:HGNC:37939]	63.7	94.7	**1.46**	0.002196
BPTFP1	bromodomain PHD finger transcription factor pseudogene 1 [Source:HGNC Symbol;Acc:HGNC:52869]	44.1	65.6	**1.46**	0.01693
RAD51-AS1	RAD51 antisense RNA 1 (head to head) [Source:HGNC Symbol;Acc:HGNC:48621]	30.4	45.8	**1.46**	0.03855
ELF1	E74 like ETS transcription factor 1 [Source:HGNC Symbol;Acc:HGNC:3316]	2157.2	3143.5	**1.45**	7.09E-36
YAP1	Yes associated protein 1 [Source:HGNC Symbol;Acc:HGNC:16262]	5971.9	8717.7	**1.45**	1.79E-24
PRR13	proline rich 13 [Source:HGNC Symbol;Acc:HGNC:24528]	695.8	1015.4	**1.45**	1.27E-21
SAP30L	SAP30 like [Source:HGNC Symbol;Acc:HGNC:25663]	512.3	743.4	**1.45**	1.51E-18
CDC37L1	cell division cycle 37 like 1 [Source:HGNC Symbol;Acc:HGNC:17179]	1074.8	1559.9	**1.45**	3.62E-18
FAM135A	family with sequence similarity 135 member A [Source:HGNC Symbol;Acc:HGNC:21084]	1087.7	1584.3	**1.45**	4.83E-17
LATS2	large tumor suppressor kinase 2 [Source:HGNC Symbol;Acc:HGNC:6515]	979.0	1419.6	**1.45**	1.12E-15
NABP1	nucleic acid binding protein 1 [Source:HGNC Symbol;Acc:HGNC:26232]	901.7	1332.6	**1.45**	2.99E-15
BCL2L1	BCL2 like 1 [Source:HGNC Symbol;Acc:HGNC:992]	892.8	1324.0	**1.45**	1.01E-11
FAM160A1	family with sequence similarity 160 member A1 [Source:HGNC Symbol;Acc:HGNC:34237]	314.2	459.9	**1.45**	1.68E-10
KCNMB3	potassium calcium-activated channel subfamily M regulatory beta subunit 3 [Source:HGNC Symbol;Acc:	215.9	314.2	**1.45**	2.00E-10
LRRC1	leucine rich repeat containing 1 [Source:HGNC Symbol;Acc:HGNC:14307]	377.6	552.3	**1.45**	3.68E-10
SHROOM4	shroom family member 4 [Source:HGNC Symbol;Acc:HGNC:29215]	238.9	348.4	**1.45**	7.83E-10
SRD5A3	steroid 5 alpha-reductase 3 [Source:HGNC Symbol;Acc:HGNC:25812]	278.9	408.4	**1.45**	2.59E-09

KCTD21	potassium channel tetramerization domain containing 21 [Source:HGNC Symbol;Acc:HGNC:27452]	174.1	254.9	**1.45**	2.22E-08
ARHGEF39	Rho guanine nucleotide exchange factor 39 [Source:HGNC Symbol;Acc:HGNC:25909]	283.8	419.0	**1.45**	8.38E-08
LAMA2	laminin subunit alpha 2 [Source:HGNC Symbol;Acc:HGNC:6482]	155.8	233.3	**1.45**	8.14E-05
ETHE1	ETHE1, persulfide dioxygenase [Source:HGNC Symbol;Acc:HGNC:23287]	64.3	94.3	**1.45**	0.0001619
EHHADH-AS1	EHHADH antisense RNA 1 [Source:HGNC Symbol;Acc:HGNC:44133]	28.7	42.9	**1.45**	0.01358
PCLO	piccolo presynaptic cytomatrix protein [Source:HGNC Symbol;Acc:HGNC:13406]	96.2	143.5	**1.45**	0.01437
MYH11	myosin heavy chain 11 [Source:HGNC Symbol;Acc:HGNC:7569]	25.6	40.4	**1.45**	0.03907
REEP3	receptor accessory protein 3 [Source:HGNC Symbol;Acc:HGNC:23711]	5257.2	7581.6	**1.44**	9.29E-46
CAP1	cyclase associated actin cytoskeleton regulatory protein 1 [Source:HGNC Symbol;Acc:HGNC:20040]	5207.3	7549.3	**1.44**	1.38E-36
ZPR1	ZPR1 zinc finger [Source:HGNC Symbol;Acc:HGNC:13051]	1063.5	1543.3	**1.44**	2.35E-22
DHRS7	dehydrogenase/reductase 7 [Source:HGNC Symbol;Acc:HGNC:21524]	1342.1	1937.2	**1.44**	6.37E-21
NUP50	nucleoporin 50 [Source:HGNC Symbol;Acc:HGNC:8065]	1799.8	2604.7	**1.44**	1.92E-20
LDLRAD3	low density lipoprotein receptor class A domain containing 3 [Source:HGNC Symbol;Acc:HGNC:27046]	1079.5	1558.4	**1.44**	7.74E-19
RAB31	RAB31, member RAS oncogene family [Source:HGNC Symbol;Acc:HGNC:9771]	1118.5	1619.7	**1.44**	1.07E-17
VPS13C	vacuolar protein sorting 13 homolog C [Source:HGNC Symbol;Acc:HGNC:23594]	3910.0	5672.6	**1.44**	2.72E-15
PPM1B	protein phosphatase, Mg2+/Mn2+ dependent 1B [Source:HGNC Symbol;Acc:HGNC:9276]	823.0	1194.4	**1.44**	1.46E-13
C8orf88	chromosome 8 open reading frame 88 [Source:HGNC Symbol;Acc:HGNC:44672]	301.0	435.7	**1.44**	1.47E-13
SNX8	sorting nexin 8 [Source:HGNC Symbol;Acc:HGNC:14972]	496.4	730.7	**1.44**	1.56E-07
ALDH3B1	aldehyde dehydrogenase 3 family member B1 [Source:HGNC Symbol;Acc:HGNC:410]	161.1	237.2	**1.44**	1.18E-06
TECPR1	tectonin beta-propeller repeat containing 1 [Source:HGNC Symbol;Acc:HGNC:22214]	116.7	169.4	**1.44**	0.0001093
BCAT2	branched chain amino acid transaminase 2 [Source:HGNC Symbol;Acc:HGNC:977]	92.6	135.1	**1.44**	0.0003093
NCKIPSD	NCK interacting protein with SH3 domain [Source:HGNC Symbol;Acc:HGNC:15486]	96.4	140.3	**1.44**	0.000653
BTBD8	BTB domain containing 8 [Source:HGNC Symbol;Acc:HGNC:21019]	76.0	111.0	**1.44**	0.001628
FBXO6	F-box protein 6 [Source:HGNC Symbol;Acc:HGNC:13585]	34.4	50.3	**1.44**	0.007209
CACNB4	calcium voltage-gated channel auxiliary subunit beta 4 [Source:HGNC Symbol;Acc:HGNC:1404]	24.4	37.8	**1.44**	0.03975
TMEM43	transmembrane protein 43 [Source:HGNC Symbol;Acc:HGNC:28472]	5385.1	7719.8	**1.43**	6.02E-40
SBDS	SBDS, ribosome maturation factor [Source:HGNC Symbol;Acc:HGNC:19440]	3724.9	5376.0	**1.43**	2.45E-32
RHEB	Ras homolog, mTORC1 binding [Source:HGNC Symbol;Acc:HGNC:10011]	3058.9	4393.8	**1.43**	5.47E-26
PERP	PERP, TP53 apoptosis effector [Source:HGNC Symbol;Acc:HGNC:17637]	710.3	1023.7	**1.43**	8.40E-22
UTRN	utrophin [Source:HGNC Symbol;Acc:HGNC:12635]	6835.9	9776.3	**1.43**	1.23E-21
CD9	CD9 molecule [Source:HGNC Symbol;Acc:HGNC:1709]	5811.3	8375.1	**1.43**	2.52E-20
MYCBP2	MYC binding protein 2, E3 ubiquitin protein ligase [Source:HGNC Symbol;Acc:HGNC:23386]	3474.3	5038.0	**1.43**	9.90E-18
PTPN12	protein tyrosine phosphatase, non-receptor type 12 [Source:HGNC Symbol;Acc:HGNC:9645]	915.0	1316.5	**1.43**	4.88E-16
BZW2	basic leucine zipper and W2 domains 2 [Source:HGNC Symbol;Acc:HGNC:18808]	728.5	1058.2	**1.43**	3.55E-15
TOM1L1	target of myb1 like 1 membrane trafficking protein [Source:HGNC Symbol;Acc:HGNC:11983]	634.3	906.4	**1.43**	6.50E-14
MBNL1-AS1	MBNL1 antisense RNA 1 [Source:HGNC Symbol;Acc:HGNC:44584]	1007.4	1440.3	**1.43**	6.35E-12
HSF1	heat shock transcription factor 1 [Source:HGNC Symbol;Acc:HGNC:5224]	267.9	386.5	**1.43**	1.64E-07
TP53I3	tumor protein p53 inducible protein 3 [Source:HGNC Symbol;Acc:HGNC:19373]	177.8	255.6	**1.43**	2.94E-06
GSTO2	glutathione S-transferase omega 2 [Source:HGNC Symbol;Acc:HGNC:23064]	113.8	163.5	**1.43**	5.84E-06
MROH1	maestro heat like repeat family member 1 [Source:HGNC Symbol;Acc:HGNC:26958]	180.3	263.9	**1.43**	1.37E-05
CDH23	cadherin related 23 [Source:HGNC Symbol;Acc:HGNC:13733]	196.9	286.8	**1.43**	3.59E-05
SELENBP1	selenium binding protein 1 [Source:HGNC Symbol;Acc:HGNC:10719]	197.4	289.9	**1.43**	0.0001957
HMGN2P46	high mobility group nucleosomal binding domain 2 pseudogene 46 [Source:HGNC Symbol;Acc:HGNC:26	57.5	83.8	**1.43**	0.0004649
CPAMD8	C3 and PZP like, alpha-2-macroglobulin domain containing 8 [Source:HGNC Symbol;Acc:HGNC:23228]	203.9	296.0	**1.43**	0.0007699
DNAH7	dynein axonemal heavy chain 7 [Source:HGNC Symbol;Acc:HGNC:18661]	62.2	90.2	**1.43**	0.001419
EPS15	epidermal growth factor receptor pathway substrate 15 [Source:HGNC Symbol;Acc:HGNC:3419]	1723.0	2458.6	**1.42**	1.74E-28
NCK2	NCK adaptor protein 2 [Source:HGNC Symbol;Acc:HGNC:7665]	2053.2	2932.8	**1.42**	2.28E-27
UBE2J1	ubiquitin conjugating enzyme E2 J1 [Source:HGNC Symbol;Acc:HGNC:17598]	1493.2	2123.0	**1.42**	4.51E-26
SYS1	SYS1, golgi trafficking protein [Source:HGNC Symbol;Acc:HGNC:16162]	374.3	535.7	**1.42**	1.16E-17
UBE2R2	ubiquitin conjugating enzyme E2 R2 [Source:HGNC Symbol;Acc:HGNC:19907]	869.3	1239.9	**1.42**	1.18E-17
DTX3L	deltex E3 ubiquitin ligase 3L [Source:HGNC Symbol;Acc:HGNC:30323]	906.1	1293.0	**1.42**	1.83E-16
BBS10	Bardet-Biedl syndrome 10 [Source:HGNC Symbol;Acc:HGNC:26291]	1085.5	1554.5	**1.42**	5.86E-16
ZCCHC2	zinc finger CCHC-type containing 2 [Source:HGNC Symbol;Acc:HGNC:22916]	348.0	495.9	**1.42**	6.07E-14
WIPF1	WAS/WASL interacting protein family member 1 [Source:HGNC Symbol;Acc:HGNC:12736]	721.8	1023.5	**1.42**	2.07E-13
DOCK9	dedicator of cytokinesis 9 [Source:HGNC Symbol;Acc:HGNC:14132]	951.4	1359.7	**1.42**	4.93E-11
MTMR14	myotubularin related protein 14 [Source:HGNC Symbol;Acc:HGNC:26190]	330.7	473.9	**1.42**	2.62E-09
ABALON	apoptotic BCL2L1-antisense long non-coding RNA [Source:HGNC Symbol;Acc:HGNC:49667]	470.8	685.5	**1.42**	9.86E-09
PRR4	proline rich 4 [Source:HGNC Symbol;Acc:HGNC:18020]	274.6	397.9	**1.42**	1.64E-08
EPHA4	EPH receptor A4 [Source:HGNC Symbol;Acc:HGNC:3388]	321.1	460.0	**1.42**	3.89E-08
COQ8B	coenzyme Q8B [Source:HGNC Symbol;Acc:HGNC:19041]	159.8	229.7	**1.42**	5.07E-08
WDFY2	WD repeat and FYVE domain containing 2 [Source:HGNC Symbol;Acc:HGNC:20482]	212.1	306.1	**1.42**	2.23E-07
MCRIP1	MAPK regulated corepressor interacting protein 1 [Source:HGNC Symbol;Acc:HGNC:28007]	134.2	192.4	**1.42**	1.28E-06
CTC1	CST telomere replication complex component 1 [Source:HGNC Symbol;Acc:HGNC:26169]	123.4	179.0	**1.42**	0.0001472
RAC1P2	Rac family small GTPase 1 pseudogene 2 [Source:HGNC Symbol;Acc:HGNC:31414]	100.7	143.0	**1.42**	0.004965
MT1H	metallothionein 1H [Source:HGNC Symbol;Acc:HGNC:7400]	469.0	711.9	**1.42**	0.01195
NLGN1	neuroligin 1 [Source:HGNC Symbol;Acc:HGNC:14291]	41.3	60.3	**1.42**	0.01352
CORIN	corin, serine peptidase [Source:HGNC Symbol;Acc:HGNC:19012]	34.2	49.6	**1.42**	0.01566
TLK2P1	tousled like kinase 2 pseudogene 1 [Source:HGNC Symbol;Acc:HGNC:18048]	32.7	47.1	**1.42**	0.0231
TMBIM1	transmembrane BAX inhibitor motif containing 1 [Source:HGNC Symbol;Acc:HGNC:23410]	5228.4	7374.1	**1.41**	1.05E-26
NUDT16	nudix hydrolase 16 [Source:HGNC Symbol;Acc:HGNC:26442]	680.9	965.4	**1.41**	1.18E-25
SEPT10	septin 10 [Source:HGNC Symbol;Acc:HGNC:14349]	3416.8	4814.8	**1.41**	8.86E-25
MYOF	myoferlin [Source:HGNC Symbol;Acc:HGNC:3656]	7765.3	11003.9	**1.41**	8.82E-22

RECK	reversion inducing cysteine rich protein with kazal motifs [Source:HGNC Symbol;Acc:HGNC:11345]	1036.5	1467.6	**1.41**	2.33E-21
DDRGK1	DDRGK domain containing 1 [Source:HGNC Symbol;Acc:HGNC:16110]	596.2	845.3	**1.41**	1.51E-18
FKBP15	FK506 binding protein 15 [Source:HGNC Symbol;Acc:HGNC:23397]	1698.2	2425.6	**1.41**	1.38E-17
CLIP1	CAP-Gly domain containing linker protein 1 [Source:HGNC Symbol;Acc:HGNC:10461]	5701.5	8102.0	**1.41**	2.09E-17
MINDY2	MINDY lysine 48 deubiquitinase 2 [Source:HGNC Symbol;Acc:HGNC:26954]	2222.9	3194.3	**1.41**	4.13E-17
CARS2	cysteinyl-tRNA synthetase 2, mitochondrial [Source:HGNC Symbol;Acc:HGNC:25695]	569.6	807.3	**1.41**	5.13E-15
ASL	argininosuccinate lyase [Source:HGNC Symbol;Acc:HGNC:746]	468.3	664.7	**1.41**	4.76E-14
RRAGC	Ras related GTP binding C [Source:HGNC Symbol;Acc:HGNC:19902]	597.1	854.1	**1.41**	8.37E-14
IP6K2	inositol hexakisphosphate kinase 2 [Source:HGNC Symbol;Acc:HGNC:17313]	431.1	612.7	**1.41**	2.12E-12
CYB5R3	cytochrome b5 reductase 3 [Source:HGNC Symbol;Acc:HGNC:2873]	1812.1	2555.0	**1.41**	4.98E-11
POC1B	POC1 centriolar protein B [Source:HGNC Symbol;Acc:HGNC:30836]	581.8	833.2	**1.41**	9.28E-11
KAT8	lysine acetyltransferase 8 [Source:HGNC Symbol;Acc:HGNC:17933]	357.0	507.9	**1.41**	2.05E-09
DBNDD2	dysbindin domain containing 2 [Source:HGNC Symbol;Acc:HGNC:15881]	234.7	337.0	**1.41**	8.05E-09
SYS1-DBNDD2	SYS1-DBNDD2 readthrough (NMD candidate) [Source:HGNC Symbol;Acc:HGNC:33535]	242.7	347.6	**1.41**	1.26E-08
FOSL2	FOS like 2, AP-1 transcription factor subunit [Source:HGNC Symbol;Acc:HGNC:3798]	1467.3	2082.2	**1.41**	2.11E-07
STX1A	syntaxin 1A [Source:HGNC Symbol;Acc:HGNC:11433]	142.7	202.6	**1.41**	7.67E-06
KLHL3	kelch like family member 3 [Source:HGNC Symbol;Acc:HGNC:6354]	166.6	241.1	**1.41**	0.0001149
COX7A1	cytochrome c oxidase subunit 7A1 [Source:HGNC Symbol;Acc:HGNC:2287]	117.1	166.7	**1.41**	0.0001869
EEF1A1P13	eukaryotic translation elongation factor 1 alpha 1 pseudogene 13 [Source:HGNC Symbol;Acc:HGNC:319(	81.5	116.7	**1.41**	0.0004192
OSCP1	organic solute carrier partner 1 [Source:HGNC Symbol;Acc:HGNC:29971]	110.5	158.0	**1.41**	0.001276
NINJ1	ninjurin 1 [Source:HGNC Symbol;Acc:HGNC:7824]	244.0	357.9	**1.41**	0.001472
TMCO6	transmembrane and coiled-coil domains 6 [Source:HGNC Symbol;Acc:HGNC:28814]	60.6	85.5	**1.41**	0.00326
WASH3P	WAS protein family homolog 3 pseudogene [Source:HGNC Symbol;Acc:HGNC:24362]	44.7	64.0	**1.41**	0.003826
SLC44A3	solute carrier family 44 member 3 [Source:HGNC Symbol;Acc:HGNC:28689]	75.0	108.9	**1.41**	0.004351
HTR5BP	5-hydroxytryptamine receptor 5B, pseudogene [Source:HGNC Symbol;Acc:HGNC:16291]	29.0	42.1	**1.41**	0.03845
DIXDC1	DIX domain containing 1 [Source:HGNC Symbol;Acc:HGNC:23695]	3000.2	4216.5	**1.40**	6.60E-29
LARP1B	La ribonucleoprotein domain family member 1B [Source:HGNC Symbol;Acc:HGNC:24704]	921.2	1296.7	**1.40**	2.83E-21
ADH5	alcohol dehydrogenase 5 (class III), chi polypeptide [Source:HGNC Symbol;Acc:HGNC:253]	3319.2	4714.6	**1.40**	3.45E-21
OSER1	oxidative stress responsive serine rich 1 [Source:HGNC Symbol;Acc:HGNC:16105]	741.2	1046.7	**1.40**	1.96E-19
TNIP2	TNFAIP3 interacting protein 2 [Source:HGNC Symbol;Acc:HGNC:19118]	634.2	891.3	**1.40**	3.16E-19
ZNF532	zinc finger protein 532 [Source:HGNC Symbol;Acc:HGNC:30940]	2161.2	3045.0	**1.40**	2.83E-18
KRAS	KRAS proto-oncogene, GTPase [Source:HGNC Symbol;Acc:HGNC:6407]	1416.6	2007.5	**1.40**	2.96E-18
CLDN12	claudin 12 [Source:HGNC Symbol;Acc:HGNC:2034]	2073.9	2917.9	**1.40**	6.52E-18
INSR	insulin receptor [Source:HGNC Symbol;Acc:HGNC:6091]	815.1	1153.6	**1.40**	4.70E-17
AP1M1	adaptor related protein complex 1 subunit mu 1 [Source:HGNC Symbol;Acc:HGNC:13667]	536.6	756.7	**1.40**	3.35E-15
MYCBP2-AS1	MYCBP2 antisense RNA 1 [Source:HGNC Symbol;Acc:HGNC:41023]	494.8	701.1	**1.40**	1.23E-13
MIOS	meiosis regulator for oocyte development [Source:HGNC Symbol;Acc:HGNC:21905]	828.6	1167.1	**1.40**	4.06E-11
RNF19B	ring finger protein 19B [Source:HGNC Symbol;Acc:HGNC:26886]	736.0	1044.1	**1.40**	1.63E-09
OSBPL6	oxysterol binding protein like 6 [Source:HGNC Symbol;Acc:HGNC:16388]	180.9	254.0	**1.40**	3.52E-06
KCNK5	potassium two pore domain channel subfamily K member 5 [Source:HGNC Symbol;Acc:HGNC:6280]	873.7	1288.8	**1.40**	6.84E-06
CTH	cystathionine gamma-lyase [Source:HGNC Symbol;Acc:HGNC:2501]	136.2	191.6	**1.40**	0.0001458
NOS2	nitric oxide synthase 2 [Source:HGNC Symbol;Acc:HGNC:7873]	183.4	274.6	**1.40**	0.0002959
SLC25A28	solute carrier family 25 member 28 [Source:HGNC Symbol;Acc:HGNC:23472]	135.0	192.3	**1.40**	0.002985
AC090673.1	uncharacterized LOC100129940 [Source:NCBI gene;Acc:100129940]	43.0	60.2	**1.40**	0.01804
FUK	fucokinase [Source:HGNC Symbol;Acc:HGNC:29500]	39.7	55.9	**1.40**	0.02308
AP3S1	adaptor related protein complex 3 subunit sigma 1 [Source:HGNC Symbol;Acc:HGNC:2013]	3693.0	5153.4	**1.39**	1.91E-39
TMEM165	transmembrane protein 165 [Source:HGNC Symbol;Acc:HGNC:30760]	3700.0	5174.8	**1.39**	1.06E-33
TMEM123	transmembrane protein 123 [Source:HGNC Symbol;Acc:HGNC:30138]	4225.5	5932.7	**1.39**	4.09E-33
STYX	serine/threonine/tyrosine interacting protein [Source:HGNC Symbol;Acc:HGNC:11447]	1393.3	1943.9	**1.39**	5.23E-26
AVL9	AVL9 cell migration associated [Source:HGNC Symbol;Acc:HGNC:28994]	802.0	1122.2	**1.39**	3.06E-22
HIGD1A	HIG1 hypoxia inducible domain family member 1A [Source:HGNC Symbol;Acc:HGNC:29527]	2773.7	3846.1	**1.39**	4.45E-21
SLC35E3	solute carrier family 35 member E3 [Source:HGNC Symbol;Acc:HGNC:20864]	551.1	770.9	**1.39**	4.79E-18
MON1B	MON1 homolog B, secretory trafficking associated [Source:HGNC Symbol;Acc:HGNC:25020]	731.5	1024.5	**1.39**	1.10E-16
TNKS1BP1	tankyrase 1 binding protein 1 [Source:HGNC Symbol;Acc:HGNC:19081]	1621.8	2274.0	**1.39**	3.95E-16
MCUB	mitochondrial calcium uniporter dominant negative beta subunit [Source:HGNC Symbol;Acc:HGNC:2607	659.1	917.3	**1.39**	6.07E-14
KDM1B	lysine demethylase 1B [Source:HGNC Symbol;Acc:HGNC:21577]	460.3	645.2	**1.39**	2.12E-13
CALCOCO1	calcium binding and coiled-coil domain 1 [Source:HGNC Symbol;Acc:HGNC:29306]	1032.2	1474.3	**1.39**	1.67E-12
SKAP2	src kinase associated phosphoprotein 2 [Source:HGNC Symbol;Acc:HGNC:15687]	429.1	600.6	**1.39**	7.44E-12
KATNA1	katanin catalytic subunit A1 [Source:HGNC Symbol;Acc:HGNC:6216]	533.0	747.0	**1.39**	2.35E-11
SMS	spermine synthase [Source:HGNC Symbol;Acc:HGNC:11123]	2946.6	4181.8	**1.39**	1.17E-10
ZBED1	zinc finger BED-type containing 1 [Source:HGNC Symbol;Acc:HGNC:447]	336.1	481.3	**1.39**	1.12E-09
AKAP1	A-kinase anchoring protein 1 [Source:HGNC Symbol;Acc:HGNC:367]	662.3	930.4	**1.39**	3.10E-08
SIK3	SIK family kinase 3 [Source:HGNC Symbol;Acc:HGNC:29165]	410.4	573.7	**1.39**	6.34E-08
LCN2	lipocalin 2 [Source:HGNC Symbol;Acc:HGNC:6526]	330.3	458.5	**1.39**	7.85E-08
GCLC	glutamate-cysteine ligase catalytic subunit [Source:HGNC Symbol;Acc:HGNC:4311]	806.1	1164.7	**1.39**	8.23E-08
FNDC4	fibronectin type III domain containing 4 [Source:HGNC Symbol;Acc:HGNC:20239]	213.5	302.2	**1.39**	9.57E-07
STEAP1	STEAP family member 1 [Source:HGNC Symbol;Acc:HGNC:11378]	944.6	1347.0	**1.39**	2.08E-06
PRR34-AS1	PRR34 antisense RNA 1 [Source:HGNC Symbol;Acc:HGNC:50499]	113.7	158.5	**1.39**	7.71E-06
PAPPA2	pappalysin 2 [Source:HGNC Symbol;Acc:HGNC:14615]	148.6	209.0	**1.39**	9.66E-05
ZMIZ2	zinc finger MIZ-type containing 2 [Source:HGNC Symbol;Acc:HGNC:22229]	88.0	125.0	**1.39**	0.004546
TWIST1	twist family bHLH transcription factor 1 [Source:HGNC Symbol;Acc:HGNC:12428]	78.6	111.9	**1.39**	0.004586
PABPC5-AS1	PABPC5 antisense RNA 1 [Source:HGNC Symbol;Acc:HGNC:31845]	61.3	87.3	**1.39**	0.007045

RPL34P31	ribosomal protein L34 pseudogene 31 [Source:HGNC Symbol;Acc:HGNC:36899]	47.7	66.6	**1.39**	0.01255
ARRDC1-AS1	ARRDC1 antisense RNA 1 [Source:HGNC Symbol;Acc:HGNC:23395]	42.3	59.6	**1.39**	0.01437
KCTD3	potassium channel tetramerization domain containing 3 [Source:HGNC Symbol;Acc:HGNC:21305]	1422.9	1976.8	**1.39**	2.45E-28
KPNA3	karyopherin subunit alpha 3 [Source:HGNC Symbol;Acc:HGNC:6396]	979.6	1364.5	**1.39**	4.11E-26
GTF2E2	general transcription factor IIE subunit 2 [Source:HGNC Symbol;Acc:HGNC:4651]	1064.6	1474.0	**1.39**	1.50E-23
EXOC1	exocyst complex component 1 [Source:HGNC Symbol;Acc:HGNC:30380]	2332.3	3243.3	**1.39**	4.01E-22
CDS2	CDP-diacylglycerol synthase 2 [Source:HGNC Symbol;Acc:HGNC:1801]	1171.3	1626.7	**1.39**	5.80E-22
SLC35A1	solute carrier family 35 member A1 [Source:HGNC Symbol;Acc:HGNC:11021]	848.5	1173.8	**1.39**	1.16E-19
RAB5B	RAB5B, member RAS oncogene family [Source:HGNC Symbol;Acc:HGNC:9784]	1150.5	1600.3	**1.39**	5.78E-19
GTPBP1	GTP binding protein 1 [Source:HGNC Symbol;Acc:HGNC:4669]	621.5	866.7	**1.39**	1.21E-18
PHF20	PHD finger protein 20 [Source:HGNC Symbol;Acc:HGNC:16098]	1669.0	2317.9	**1.39**	3.74E-17
RASAL2	RAS protein activator like 2 [Source:HGNC Symbol;Acc:HGNC:9874]	918.8	1271.2	**1.39**	9.05E-17
WIPI1	WD repeat domain, phosphoinositide interacting 1 [Source:HGNC Symbol;Acc:HGNC:25471]	936.3	1312.0	**1.39**	3.72E-14
TLDC1	TBC/LysM-associated domain containing 1 [Source:HGNC Symbol;Acc:HGNC:29325]	518.1	724.0	**1.39**	4.94E-13
PKN1	protein kinase N1 [Source:HGNC Symbol;Acc:HGNC:9405]	613.1	854.4	**1.39**	2.77E-11
TOM1	target of myb1 membrane trafficking protein [Source:HGNC Symbol;Acc:HGNC:11982]	473.1	660.2	**1.39**	4.14E-11
GAN	gigaxonin [Source:HGNC Symbol;Acc:HGNC:4137]	869.6	1215.8	**1.39**	7.41E-11
GALC	galactosylceramidase [Source:HGNC Symbol;Acc:HGNC:4115]	298.4	415.3	**1.39**	1.06E-10
RHOU	ras homolog family member U [Source:HGNC Symbol;Acc:HGNC:17794]	644.1	908.6	**1.39**	3.61E-10
DYNC1I1	dynein cytoplasmic 1 intermediate chain 1 [Source:HGNC Symbol;Acc:HGNC:2963]	472.5	665.2	**1.39**	9.77E-10
KCNK6	potassium two pore domain channel subfamily K member 6 [Source:HGNC Symbol;Acc:HGNC:6281]	410.1	571.9	**1.39**	5.71E-09
TAF4B	TATA-box binding protein associated factor 4b [Source:HGNC Symbol;Acc:HGNC:11538]	197.3	273.5	**1.39**	6.42E-09
ZMYND8	zinc finger MYND-type containing 8 [Source:HGNC Symbol;Acc:HGNC:9397]	610.1	858.8	**1.39**	7.52E-09
PPP1R12C	protein phosphatase 1 regulatory subunit 12C [Source:HGNC Symbol;Acc:HGNC:14947]	276.7	384.4	**1.39**	1.03E-07
CCNG2	cyclin G2 [Source:HGNC Symbol;Acc:HGNC:1593]	1151.2	1595.7	**1.39**	4.29E-06
S100A16	S100 calcium binding protein A16 [Source:HGNC Symbol;Acc:HGNC:20441]	431.4	614.8	**1.39**	2.84E-05
OSER1-DT	OSER1 divergent transcript [Source:HGNC Symbol;Acc:HGNC:48585]	741.2	1046.7	**1.39**	6.39E-05
LPIN3	lipin 3 [Source:HGNC Symbol;Acc:HGNC:14451]	139.3	194.1	**1.39**	9.07E-05
ZNRF1	zinc and ring finger 1 [Source:HGNC Symbol;Acc:HGNC:18452]	76.9	107.7	**1.39**	0.003498
HORMAD2-AS1	HORMAD2 and MTMR3 antisense RNA 1 [Source:HGNC Symbol;Acc:HGNC:50729]	28.7	40.4	**1.39**	0.04614
KIDINS220	kinase D interacting substrate 220 [Source:HGNC Symbol;Acc:HGNC:29508]	5553.6	7644.9	**1.38**	9.61E-28
EMC3	ER membrane protein complex subunit 3 [Source:HGNC Symbol;Acc:HGNC:23999]	1391.3	1922.9	**1.38**	4.75E-19
RAB29	RAB29, member RAS oncogene family [Source:HGNC Symbol;Acc:HGNC:9789]	653.7	903.9	**1.38**	1.25E-18
CORO1C	coronin 1C [Source:HGNC Symbol;Acc:HGNC:2254]	2401.8	3315.7	**1.38**	1.42E-18
TRAK2	trafficking kinesin protein 2 [Source:HGNC Symbol;Acc:HGNC:13206]	1897.9	2627.1	**1.38**	2.57E-18
DPH3	diphthamide biosynthesis 3 [Source:HGNC Symbol;Acc:HGNC:27717]	1010.6	1393.1	**1.38**	9.85E-18
AGL	amylo-alpha-1, 6-glucosidase, 4-alpha-glucanotransferase [Source:HGNC Symbol;Acc:HGNC:321]	839.2	1162.4	**1.38**	2.10E-16
NUMB	NUMB, endocytic adaptor protein [Source:HGNC Symbol;Acc:HGNC:8060]	1468.6	2031.4	**1.38**	3.25E-16
RMC1	regulator of MON1-CCZ1 [Source:HGNC Symbol;Acc:HGNC:24326]	681.2	939.8	**1.38**	1.97E-14
PTPN9	protein tyrosine phosphatase, non-receptor type 9 [Source:HGNC Symbol;Acc:HGNC:9661]	778.3	1075.4	**1.38**	5.00E-14
HAPLN1	hyaluronan and proteoglycan link protein 1 [Source:HGNC Symbol;Acc:HGNC:2380]	52202.3	72001.5	**1.38**	8.42E-12
STK10	serine/threonine kinase 10 [Source:HGNC Symbol;Acc:HGNC:11388]	443.8	620.1	**1.38**	3.27E-10
FTSJ1	FtsJ RNA methyltransferase homolog 1 [Source:HGNC Symbol;Acc:HGNC:13254]	389.6	540.3	**1.38**	4.27E-10
TTC7A	tetratricopeptide repeat domain 7A [Source:HGNC Symbol;Acc:HGNC:19750]	311.1	430.6	**1.38**	6.62E-10
ZNF33B	zinc finger protein 33B [Source:HGNC Symbol;Acc:HGNC:13097]	406.5	563.6	**1.38**	3.40E-08
JAGN1	jagunal homolog 1 [Source:HGNC Symbol;Acc:HGNC:26926]	278.2	382.9	**1.38**	3.78E-08
MIR29B2CHG	MIR29B2 and MIR29C host gene [Source:HGNC Symbol;Acc:HGNC:32018]	283.2	391.7	**1.38**	2.61E-07
SPATA5L1	spermatogenesis associated 5 like 1 [Source:HGNC Symbol;Acc:HGNC:28762]	173.1	238.2	**1.38**	4.19E-07
KDM7A-DT	KDM7A divergent transcript [Source:HGNC Symbol;Acc:HGNC:48959]	2347.7	3014.0	**1.38**	8.11E-07
ABHD3	abhydrolase domain containing 3 [Source:HGNC Symbol;Acc:HGNC:18718]	192.4	264.8	**1.38**	5.89E-06
SDR39U1	short chain dehydrogenase/reductase family 39U member 1 [Source:HGNC Symbol;Acc:HGNC:20275]	235.1	329.6	**1.38**	1.86E-05
TMX2P1	thioredoxin related transmembrane protein 2 pseudogene 1 [Source:HGNC Symbol;Acc:HGNC:49916]	138.6	193.2	**1.38**	2.94E-05
NKX3-1	NK3 homeobox 1 [Source:HGNC Symbol;Acc:HGNC:7838]	287.2	405.5	**1.38**	0.0001019
PNPLA2	patatin like phospholipase domain containing 2 [Source:HGNC Symbol;Acc:HGNC:30802]	77.2	108.3	**1.38**	0.002609
TPT1P4	tumor protein, translationally-controlled 1 pseudogene 4 [Source:HGNC Symbol;Acc:HGNC:49298]	69.6	96.2	**1.38**	0.003446
IRX5	iroquois homeobox 5 [Source:HGNC Symbol;Acc:HGNC:14361]	33.7	47.0	**1.38**	0.03346
MARVELD2	MARVEL domain containing 2 [Source:HGNC Symbol;Acc:HGNC:26401]	45.8	64.9	**1.38**	0.03453
IQGAP1	IQ motif containing GTPase activating protein 1 [Source:HGNC Symbol;Acc:HGNC:6110]	26841.9	36731.5	**1.37**	2.71E-38
TRAPPC11	trafficking protein particle complex 11 [Source:HGNC Symbol;Acc:HGNC:25751]	1436.5	1961.2	**1.37**	5.33E-29
NUFIP2	NUFIP2, FMR1 interacting protein 2 [Source:HGNC Symbol;Acc:HGNC:17634]	5406.9	7413.8	**1.37**	3.57E-28
PSME3	proteasome activator subunit 3 [Source:HGNC Symbol;Acc:HGNC:9570]	1150.9	1577.2	**1.37**	1.52E-22
SWAP70	switching B cell complex subunit SWAP70 [Source:HGNC Symbol;Acc:HGNC:17070]	5028.5	6864.9	**1.37**	3.18E-21
STK4	serine/threonine kinase 4 [Source:HGNC Symbol;Acc:HGNC:11408]	1475.7	2025.7	**1.37**	1.36E-18
MUT	methylmalonyl-CoA mutase [Source:HGNC Symbol;Acc:HGNC:7526]	1135.9	1552.8	**1.37**	2.64E-18
CC2D1B	coiled-coil and C2 domain containing 1B [Source:HGNC Symbol;Acc:HGNC:29386]	1064.7	1452.9	**1.37**	4.53E-17
CNTRL	centriolin [Source:HGNC Symbol;Acc:HGNC:1858]	698.6	953.7	**1.37**	1.65E-16
PTPN2	protein tyrosine phosphatase, non-receptor type 2 [Source:HGNC Symbol;Acc:HGNC:9650]	902.9	1235.2	**1.37**	2.97E-15
ASCC1	activating signal cointegrator 1 complex subunit 1 [Source:HGNC Symbol;Acc:HGNC:24268]	690.3	940.6	**1.37**	4.28E-15
RWDD4	RWD domain containing 4 [Source:HGNC Symbol;Acc:HGNC:23750]	677.6	932.7	**1.37**	2.77E-12
SCLT1	sodium channel and clathrin linker 1 [Source:HGNC Symbol;Acc:HGNC:26406]	540.1	737.4	**1.37**	5.26E-12
PATJ	PATJ, crumbs cell polarity complex component [Source:HGNC Symbol;Acc:HGNC:28881]	1112.1	1532.2	**1.37**	1.82E-11
POLR1D	RNA polymerase I and III subunit D [Source:HGNC Symbol;Acc:HGNC:20422]	474.4	648.0	**1.37**	9.31E-11

CHST15	carbohydrate sulfotransferase 15 [Source:HGNC Symbol;Acc:HGNC:18137]	454.1	631.5	**1.37**	2.41E-09
ANKRD49	ankyrin repeat domain 49 [Source:HGNC Symbol;Acc:HGNC:25970]	273.2	372.5	**1.37**	5.44E-08
MTURN	maturin, neural progenitor differentiation regulator homolog [Source:HGNC Symbol;Acc:HGNC:25457]	1617.2	2186.0	**1.37**	1.09E-07
CHEK2	checkpoint kinase 2 [Source:HGNC Symbol;Acc:HGNC:16627]	263.0	361.2	**1.37**	2.06E-07
ARSG	arylsulfatase G [Source:HGNC Symbol;Acc:HGNC:24102]	478.2	659.1	**1.37**	3.94E-07
IGSF3	immunoglobulin superfamily member 3 [Source:HGNC Symbol;Acc:HGNC:5950]	285.7	388.6	**1.37**	8.61E-07
BTG2	BTG anti-proliferation factor 2 [Source:HGNC Symbol;Acc:HGNC:1131]	513.6	692.8	**1.37**	2.73E-06
MINDY1	MINDY lysine 48 deubiquitinase 1 [Source:HGNC Symbol;Acc:HGNC:25648]	285.8	389.3	**1.37**	5.12E-06
FOXO4	forkhead box O4 [Source:HGNC Symbol;Acc:HGNC:7139]	170.6	236.9	**1.37**	2.87E-05
ABHD1	abhydrolase domain containing 1 [Source:HGNC Symbol;Acc:HGNC:17553]	115.7	159.1	**1.37**	8.68E-05
SPSB3	splA/ryanodine receptor domain and SOCS box containing 3 [Source:HGNC Symbol;Acc:HGNC:30629]	109.3	150.5	**1.37**	0.001026
LPCAT2	lysophosphatidylcholine acyltransferase 2 [Source:HGNC Symbol;Acc:HGNC:26032]	128.5	181.2	**1.37**	0.001131
NKD1	naked cuticle homolog 1 [Source:HGNC Symbol;Acc:HGNC:17045]	176.7	246.5	**1.37**	0.00386
PIH1D2	PIH1 domain containing 2 [Source:HGNC Symbol;Acc:HGNC:25210]	72.6	100.0	**1.37**	0.004858
NUMBL	NUMB like, endocytic adaptor protein [Source:HGNC Symbol;Acc:HGNC:8061]	84.5	117.2	**1.37**	0.01094
AMDHD1	amidohydrolase domain containing 1 [Source:HGNC Symbol;Acc:HGNC:28577]	53.1	72.7	**1.37**	0.01388
BEND7	BEN domain containing 7 [Source:HGNC Symbol;Acc:HGNC:23514]	59.5	83.2	**1.37**	0.02163
HERC6	HECT and RLD domain containing E3 ubiquitin protein ligase family member 6 [Source:HGNC Symbol;Ac	41.7	57.3	**1.37**	0.03772
WASF1	WAS protein family member 1 [Source:HGNC Symbol;Acc:HGNC:12732]	41.3	57.0	**1.37**	0.04516
PTPN11	protein tyrosine phosphatase, non-receptor type 11 [Source:HGNC Symbol;Acc:HGNC:9644]	7615.0	10302.3	**1.36**	7.92E-34
CTNNB1	catenin beta 1 [Source:HGNC Symbol;Acc:HGNC:2514]	9142.1	12389.8	**1.36**	2.95E-25
NCKAP1	NCK associated protein 1 [Source:HGNC Symbol;Acc:HGNC:7666]	7547.6	10240.6	**1.36**	1.14E-23
SAP30BP	SAP30 binding protein [Source:HGNC Symbol;Acc:HGNC:30785]	1182.3	1604.7	**1.36**	2.22E-21
RAB7A	RAB7A, member RAS oncogene family [Source:HGNC Symbol;Acc:HGNC:9788]	1884.4	2563.3	**1.36**	1.82E-20
DPY19L3	dpy-19 like C-mannosyltransferase 3 [Source:HGNC Symbol;Acc:HGNC:27120]	2539.4	3456.3	**1.36**	6.65E-20
MFGE8	milk fat globule-EGF factor 8 protein [Source:HGNC Symbol;Acc:HGNC:7036]	6460.5	8867.5	**1.36**	5.13E-19
BLVRA	biliverdin reductase A [Source:HGNC Symbol;Acc:HGNC:1062]	748.1	1012.5	**1.36**	2.27E-18
ROCK1	Rho associated coiled-coil containing protein kinase 1 [Source:HGNC Symbol;Acc:HGNC:10251]	5076.2	6905.0	**1.36**	8.52E-18
PSEN1	presenilin 1 [Source:HGNC Symbol;Acc:HGNC:9508]	1589.3	2158.8	**1.36**	8.19E-17
ACOT9	acyl-CoA thioesterase 9 [Source:HGNC Symbol;Acc:HGNC:17152]	1499.7	2040.5	**1.36**	8.60E-17
SLC30A1	solute carrier family 30 member 1 [Source:HGNC Symbol;Acc:HGNC:11012]	4481.1	6055.4	**1.36**	1.60E-16
NPC1	NPC intracellular cholesterol transporter 1 [Source:HGNC Symbol;Acc:HGNC:7897]	1836.6	2505.1	**1.36**	1.94E-16
DBT	dihydrolipoamide branched chain transacylase E2 [Source:HGNC Symbol;Acc:HGNC:2698]	619.5	843.4	**1.36**	7.67E-14
CCNDBP1	cyclin D1 binding protein 1 [Source:HGNC Symbol;Acc:HGNC:1587]	820.8	1120.3	**1.36**	2.23E-13
FCHO2	FCH domain only 2 [Source:HGNC Symbol;Acc:HGNC:25180]	1074.7	1458.7	**1.36**	2.62E-13
AACS	acetoacetyl-CoA synthetase [Source:HGNC Symbol;Acc:HGNC:21298]	787.1	1071.0	**1.36**	1.81E-12
SCUBE1	signal peptide, CUB domain and EGF like domain containing 1 [Source:HGNC Symbol;Acc:HGNC:13441]	952.9	1299.4	**1.36**	1.89E-11
AGO2	argonaute 2, RISC catalytic component [Source:HGNC Symbol;Acc:HGNC:3263]	1778.3	2456.5	**1.36**	2.09E-11
LINC00511	long intergenic non-protein coding RNA 511 [Source:HGNC Symbol;Acc:HGNC:43564]	726.9	995.4	**1.36**	8.32E-11
PARP6	poly(ADP-ribose) polymerase family member 6 [Source:HGNC Symbol;Acc:HGNC:26921]	333.9	451.8	**1.36**	3.32E-10
PYGB	glycogen phosphorylase B [Source:HGNC Symbol;Acc:HGNC:9723]	7219.5	9753.1	**1.36**	5.70E-09
FCGRT	Fc fragment of IgG receptor and transporter [Source:HGNC Symbol;Acc:HGNC:3621]	302.0	413.0	**1.36**	8.30E-09
PC	pyruvate carboxylase [Source:HGNC Symbol;Acc:HGNC:8636]	687.2	938.9	**1.36**	8.72E-09
LACTB	lactamase beta [Source:HGNC Symbol;Acc:HGNC:16468]	386.8	528.1	**1.36**	7.24E-08
CLCN5	chloride voltage-gated channel 5 [Source:HGNC Symbol;Acc:HGNC:2023]	282.6	384.0	**1.36**	1.85E-07
LRRK1	leucine rich repeat kinase 1 [Source:HGNC Symbol;Acc:HGNC:18608]	224.7	303.8	**1.36**	3.61E-07
MAP3K6	mitogen-activated protein kinase kinase kinase 6 [Source:HGNC Symbol;Acc:HGNC:6858]	250.6	343.9	**1.36**	5.56E-07
TMEM35B	transmembrane protein 35B [Source:HGNC Symbol;Acc:HGNC:40021]	221.8	301.1	**1.36**	9.51E-07
CCDC39	coiled-coil domain containing 39 [Source:HGNC Symbol;Acc:HGNC:25244]	213.6	290.6	**1.36**	1.58E-05
PGS1	phosphatidylglycerophosphate synthase 1 [Source:HGNC Symbol;Acc:HGNC:30029]	182.0	248.7	**1.36**	2.54E-05
FAM206A	family with sequence similarity 206 member A [Source:HGNC Symbol;Acc:HGNC:1364]	139.4	190.3	**1.36**	5.39E-05
NBPF10	NBPF member 10 [Source:HGNC Symbol;Acc:HGNC:31992]	180.8	252.6	**1.36**	0.0004137
MNT	MAX network transcriptional repressor [Source:HGNC Symbol;Acc:HGNC:7188]	86.7	119.7	**1.36**	0.005311
PGGHG	protein-glucosylgalactosylhydroxylysine glucosidase [Source:HGNC Symbol;Acc:HGNC:26210]	73.3	101.8	**1.36**	0.005356
RWDD4P2	RWD domain containing 4 pseudogene 2 [Source:HGNC Symbol;Acc:HGNC:23754]	70.8	97.3	**1.36**	0.007337
RNPEPL1	arginyl aminopeptidase like 1 [Source:HGNC Symbol;Acc:HGNC:10079]	53.5	74.2	**1.36**	0.01101
GEMIN7	gem nuclear organelle associated protein 7 [Source:HGNC Symbol;Acc:HGNC:20045]	38.2	52.0	**1.36**	0.02033
SETD7	SET domain containing lysine methyltransferase 7 [Source:HGNC Symbol;Acc:HGNC:30412]	8996.1	12138.3	**1.35**	3.94E-62
TMCO3	transmembrane and coiled-coil domains 3 [Source:HGNC Symbol;Acc:HGNC:20329]	6814.4	9211.0	**1.35**	1.19E-32
FNDC3A	fibronectin type III domain containing 3A [Source:HGNC Symbol;Acc:HGNC:20296]	4213.9	5696.9	**1.35**	5.27E-31
ANXA2	annexin A2 [Source:HGNC Symbol;Acc:HGNC:537]	34873.6	47191.1	**1.35**	1.78E-25
HIPK3	homeodomain interacting protein kinase 3 [Source:HGNC Symbol;Acc:HGNC:4915]	5906.3	7949.6	**1.35**	1.14E-23
SPAG9	sperm associated antigen 9 [Source:HGNC Symbol;Acc:HGNC:14524]	9238.7	12484.3	**1.35**	8.93E-21
KHNYN	KH and NYN domain containing [Source:HGNC Symbol;Acc:HGNC:20166]	961.0	1298.8	**1.35**	7.50E-16
FAM50A	family with sequence similarity 50 member A [Source:HGNC Symbol;Acc:HGNC:18786]	1235.6	1667.7	**1.35**	2.15E-15
PINK1	PTEN induced putative kinase 1 [Source:HGNC Symbol;Acc:HGNC:14581]	1715.1	2325.8	**1.35**	1.36E-14
MGLL	monoglyceride lipase [Source:HGNC Symbol;Acc:HGNC:17038]	1521.7	2062.5	**1.35**	1.05E-13
GBE1	1,4-alpha-glucan branching enzyme 1 [Source:HGNC Symbol;Acc:HGNC:4180]	8795.8	11914.4	**1.35**	3.56E-13
RRAGD	Ras related GTP binding D [Source:HGNC Symbol;Acc:HGNC:19903]	806.4	1096.0	**1.35**	5.05E-13
PAPSS2	3'-phosphoadenosine 5'-phosphosulfate synthase 2 [Source:HGNC Symbol;Acc:HGNC:8604]	8567.4	11568.3	**1.35**	2.38E-12
TATDN3	TatD DNase domain containing 3 [Source:HGNC Symbol;Acc:HGNC:27010]	507.6	682.4	**1.35**	4.38E-12
THRA	thyroid hormone receptor alpha [Source:HGNC Symbol;Acc:HGNC:11796]	474.0	640.5	**1.35**	5.45E-12

TTLL5	tubulin tyrosine ligase like 5 [Source:HGNC Symbol;Acc:HGNC:19963]	539.0	726.2	**1.35**	5.83E-12
EVI5	ecotropic viral integration site 5 [Source:HGNC Symbol;Acc:HGNC:3501]	1440.2	1948.9	**1.35**	1.82E-11
ZNF765	zinc finger protein 765 [Source:HGNC Symbol;Acc:HGNC:25092]	455.7	616.1	**1.35**	4.51E-11
B4GALT1	beta-1,4-galactosyltransferase 1 [Source:HGNC Symbol;Acc:HGNC:924]	1189.4	1596.5	**1.35**	6.11E-10
GOLPH3L	golgi phosphoprotein 3 like [Source:HGNC Symbol;Acc:HGNC:24882]	765.7	1038.8	**1.35**	1.12E-09
MLLT1	MLLT1, super elongation complex subunit [Source:HGNC Symbol;Acc:HGNC:7134]	723.4	978.7	**1.35**	1.88E-09
SIPA1L2	signal induced proliferation associated 1 like 2 [Source:HGNC Symbol;Acc:HGNC:23800]	1403.2	1885.5	**1.35**	6.23E-09
MRPS36	mitochondrial ribosomal protein S36 [Source:HGNC Symbol;Acc:HGNC:16631]	368.3	494.0	**1.35**	8.29E-09
ABL1	ABL proto-oncogene 1, non-receptor tyrosine kinase [Source:HGNC Symbol;Acc:HGNC:76]	414.7	561.0	**1.35**	2.24E-08
SNX29	sorting nexin 29 [Source:HGNC Symbol;Acc:HGNC:30542]	702.7	951.0	**1.35**	3.27E-08
LPIN1	lipin 1 [Source:HGNC Symbol;Acc:HGNC:13345]	954.0	1289.5	**1.35**	1.33E-07
IL17RA	interleukin 17 receptor A [Source:HGNC Symbol;Acc:HGNC:5985]	241.4	325.8	**1.35**	2.59E-07
TBC1D19	TBC1 domain family member 19 [Source:HGNC Symbol;Acc:HGNC:25624]	249.0	336.8	**1.35**	2.52E-06
MEF2D	myocyte enhancer factor 2D [Source:HGNC Symbol;Acc:HGNC:6997]	522.1	715.1	**1.35**	3.52E-06
CLCN6	chloride voltage-gated channel 6 [Source:HGNC Symbol;Acc:HGNC:2024]	213.9	289.1	**1.35**	5.21E-06
ANXA2P2	annexin A2 pseudogene 2 [Source:HGNC Symbol;Acc:HGNC:539]	306.6	417.3	**1.35**	8.54E-06
AMTN	amelotin [Source:HGNC Symbol;Acc:HGNC:33188]	351.9	473.8	**1.35**	1.31E-05
KCNQ3	potassium voltage-gated channel subfamily Q member 3 [Source:HGNC Symbol;Acc:HGNC:6297]	140.9	189.5	**1.35**	6.09E-05
NPR3	natriuretic peptide receptor 3 [Source:HGNC Symbol;Acc:HGNC:7945]	160.9	219.2	**1.35**	0.0006517
L3MBTL4	L3MBTL4, histone methyl-lysine binding protein [Source:HGNC Symbol;Acc:HGNC:26677]	158.1	217.8	**1.35**	0.0009031
DDTL	D-dopachrome tautomerase like [Source:HGNC Symbol;Acc:HGNC:33446]	98.9	133.6	**1.35**	0.001574
DNAL4	dynein axonemal light chain 4 [Source:HGNC Symbol;Acc:HGNC:2955]	129.8	179.1	**1.35**	0.002019
CDK11A	cyclin dependent kinase 11A [Source:HGNC Symbol;Acc:HGNC:1730]	113.3	155.3	**1.35**	0.002065
PKP4-AS1	PKP4 antisense RNA 1 [Source:HGNC Symbol;Acc:HGNC:52580]	66.3	89.4	**1.35**	0.002639
SAMD15	sterile alpha motif domain containing 15 [Source:HGNC Symbol;Acc:HGNC:18631]	103.1	140.6	**1.35**	0.003817
LPP-AS2	LPP antisense RNA 2 [Source:HGNC Symbol;Acc:HGNC:27952]	67.5	91.9	**1.35**	0.006919
KMT2B	lysine methyltransferase 2B [Source:HGNC Symbol;Acc:HGNC:15840]	86.4	116.1	**1.35**	0.008464
FAM19A2	family with sequence similarity 19 member A2, C-C motif chemokine like [Source:HGNC Symbol;Acc:HGI	93.7	127.1	**1.35**	0.009971
TRIM7	tripartite motif containing 7 [Source:HGNC Symbol;Acc:HGNC:16278]	87.9	119.1	**1.35**	0.01068
LOXL4	lysyl oxidase like 4 [Source:HGNC Symbol;Acc:HGNC:17171]	2993.8	4084.8	**1.35**	0.01538
EBF1	early B cell factor 1 [Source:HGNC Symbol;Acc:HGNC:3126]	110.4	149.3	**1.35**	0.01616
ACTA2	actin, alpha 2, smooth muscle, aorta [Source:HGNC Symbol;Acc:HGNC:130]	76.4	131.1	**1.35**	0.03544
HMSD	histocompatibility minor serpin domain containing [Source:HGNC Symbol;Acc:HGNC:23037]	37.6	51.7	**1.35**	0.04147
KIF1BP	KIF1 binding protein [Source:HGNC Symbol;Acc:HGNC:23419]	2481.8	3322.9	**1.34**	7.00E-37
TMEM50B	transmembrane protein 50B [Source:HGNC Symbol;Acc:HGNC:1280]	1349.9	1807.9	**1.34**	2.14E-17
SPTLC2	serine palmitoyltransferase long chain base subunit 2 [Source:HGNC Symbol;Acc:HGNC:11278]	1395.9	1869.1	**1.34**	1.42E-16
WAC-AS1	WAC antisense RNA 1 (head to head) [Source:HGNC Symbol;Acc:HGNC:27347]	615.0	823.5	**1.34**	1.46E-14
H6PD	hexose-6-phosphate dehydrogenase/glucose 1-dehydrogenase [Source:HGNC Symbol;Acc:HGNC:4795]	1225.5	1651.4	**1.34**	3.70E-12
RTL8C	retrotransposon Gag like 8C [Source:HGNC Symbol;Acc:HGNC:2569]	410.0	548.3	**1.34**	8.53E-12
SLC38A9	solute carrier family 38 member 9 [Source:HGNC Symbol;Acc:HGNC:26907]	446.5	600.4	**1.34**	9.95E-12
DCN	decorin [Source:HGNC Symbol;Acc:HGNC:2705]	120103.2	159795.7	**1.34**	4.53E-11
LRRC57	leucine rich repeat containing 57 [Source:HGNC Symbol;Acc:HGNC:26719]	582.8	785.4	**1.34**	5.10E-11
KLHL36	kelch like family member 36 [Source:HGNC Symbol;Acc:HGNC:17844]	509.8	685.2	**1.34**	1.66E-09
FARP2	FERM, ARH/RhoGEF and pleckstrin domain protein 2 [Source:HGNC Symbol;Acc:HGNC:16460]	551.0	738.1	**1.34**	1.83E-09
TMOD2	tropomodulin 2 [Source:HGNC Symbol;Acc:HGNC:11872]	378.2	508.2	**1.34**	2.31E-09
SIAH2	siah E3 ubiquitin protein ligase 2 [Source:HGNC Symbol;Acc:HGNC:10858]	382.7	511.9	**1.34**	2.53E-09
PHF8	PHD finger protein 8 [Source:HGNC Symbol;Acc:HGNC:20672]	357.2	480.6	**1.34**	2.94E-09
NAAA	N-acylethanolamine acid amidase [Source:HGNC Symbol;Acc:HGNC:736]	308.0	410.3	**1.34**	4.78E-07
DHRSX	dehydrogenase/reductase X-linked [Source:HGNC Symbol;Acc:HGNC:18399]	198.9	267.8	**1.34**	6.50E-07
ARF5	ADP ribosylation factor 5 [Source:HGNC Symbol;Acc:HGNC:658]	243.0	327.2	**1.34**	4.33E-06
FZD5	frizzled class receptor 5 [Source:HGNC Symbol;Acc:HGNC:4043]	209.3	281.8	**1.34**	1.47E-05
ZNF708	zinc finger protein 708 [Source:HGNC Symbol;Acc:HGNC:12945]	195.0	263.0	**1.34**	4.02E-05
SINHCAF	SIN3-HDAC complex associated factor [Source:HGNC Symbol;Acc:HGNC:30702]	138.9	186.0	**1.34**	0.0001657
MKLN1-AS	MKLN1 antisense RNA [Source:HGNC Symbol;Acc:HGNC:40374]	82.9	111.3	**1.34**	0.002306
LRRFIP1P1	LRR binding FLII interacting protein 1 pseudogene 1 [Source:HGNC Symbol;Acc:HGNC:32937]	86.6	116.5	**1.34**	0.003199
ARHGAP19-SLIT1	ARHGAP19-SLIT1 readthrough (NMD candidate) [Source:HGNC Symbol;Acc:HGNC:48348]	75.2	101.2	**1.34**	0.003729
ZYX	zyxin [Source:HGNC Symbol;Acc:HGNC:13200]	123.1	166.4	**1.34**	0.004228
ZNF57	zinc finger protein 57 [Source:HGNC Symbol;Acc:HGNC:13125]	82.2	111.0	**1.34**	0.005644
N4BP2L1	NEDD4 binding protein 2 like 1 [Source:HGNC Symbol;Acc:HGNC:25037]	89.1	121.2	**1.34**	0.007711
H1F0	H1 histone family member 0 [Source:HGNC Symbol;Acc:HGNC:4714]	1476.8	2026.3	**1.34**	0.009251
NEDD9	neural precursor cell expressed, developmentally down-regulated 9 [Source:HGNC Symbol;Acc:HGNC:7	102.1	137.7	**1.34**	0.009921
BARX2	BARX homeobox 2 [Source:HGNC Symbol;Acc:HGNC:956]	119.5	167.1	**1.34**	0.01
LMBR1L	limb development membrane protein 1 like [Source:HGNC Symbol;Acc:HGNC:18268]	107.2	144.7	**1.34**	0.01088
SLC25A27	solute carrier family 25 member 27 [Source:HGNC Symbol;Acc:HGNC:21065]	84.7	114.1	**1.34**	0.01277
TTC25	tetratricopeptide repeat domain 25 [Source:HGNC Symbol;Acc:HGNC:25280]	72.5	97.1	**1.34**	0.01445
CCDC50	coiled-coil domain containing 50 [Source:HGNC Symbol;Acc:HGNC:18111]	2453.3	3253.5	**1.33**	2.65E-33
ARL8B	ADP ribosylation factor like GTPase 8B [Source:HGNC Symbol;Acc:HGNC:25564]	2219.0	2946.3	**1.33**	1.09E-27
CRK	CRK proto-oncogene, adaptor protein [Source:HGNC Symbol;Acc:HGNC:2362]	2606.2	3473.8	**1.33**	3.69E-27
PDCD6IP	programmed cell death 6 interacting protein [Source:HGNC Symbol;Acc:HGNC:8766]	3011.0	4000.8	**1.33**	5.48E-27
SERINC3	serine incorporator 3 [Source:HGNC Symbol;Acc:HGNC:11699]	7720.5	10309.8	**1.33**	1.55E-26
LTA4H	leukotriene A4 hydrolase [Source:HGNC Symbol;Acc:HGNC:6710]	1766.6	2356.1	**1.33**	1.33E-25
CDV3	CDV3 homolog [Source:HGNC Symbol;Acc:HGNC:26928]	6575.1	8764.5	**1.33**	7.04E-22

JOSD1	Josephin domain containing 1 [Source:HGNC Symbol;Acc:HGNC:28953]	1167.4	1554.0	**1.33**	9.19E-20
KIF1C	kinesin family member 1C [Source:HGNC Symbol;Acc:HGNC:6317]	2359.3	3135.9	**1.33**	2.65E-18
USP15	ubiquitin specific peptidase 15 [Source:HGNC Symbol;Acc:HGNC:12613]	2414.2	3215.2	**1.33**	8.06E-18
TANK	TRAF family member associated NFKB activator [Source:HGNC Symbol;Acc:HGNC:11562]	1321.4	1756.0	**1.33**	1.89E-16
SPRYD3	SPRY domain containing 3 [Source:HGNC Symbol;Acc:HGNC:25920]	1545.0	2070.2	**1.33**	4.10E-16
PLS3	plastin 3 [Source:HGNC Symbol;Acc:HGNC:9091]	8415.7	11114.8	**1.33**	7.72E-16
FLT1	fms related tyrosine kinase 1 [Source:HGNC Symbol;Acc:HGNC:3763]	878.6	1164.2	**1.33**	7.95E-16
FEM1B	fem-1 homolog B [Source:HGNC Symbol;Acc:HGNC:3649]	2492.4	3318.7	**1.33**	1.68E-15
FN1	fibronectin 1 [Source:HGNC Symbol;Acc:HGNC:3778]	1195480.5	1570744.5	**1.33**	2.54E-15
PTPRD	protein tyrosine phosphatase, receptor type D [Source:HGNC Symbol;Acc:HGNC:9668]	1148.6	1539.6	**1.33**	1.35E-14
CCNL2	cyclin L2 [Source:HGNC Symbol;Acc:HGNC:20570]	944.3	1252.4	**1.33**	1.49E-14
TEX2	testis expressed 2 [Source:HGNC Symbol;Acc:HGNC:30884]	980.7	1304.7	**1.33**	5.95E-14
TMEM192	transmembrane protein 192 [Source:HGNC Symbol;Acc:HGNC:26775]	832.5	1111.9	**1.33**	2.91E-13
ZFYVE21	zinc finger FYVE-type containing 21 [Source:HGNC Symbol;Acc:HGNC:20760]	785.5	1046.3	**1.33**	3.70E-13
RBL2	RB transcriptional corepressor like 2 [Source:HGNC Symbol;Acc:HGNC:9894]	2124.3	2861.0	**1.33**	1.14E-12
VPS26A	VPS26, retromer complex component A [Source:HGNC Symbol;Acc:HGNC:12711]	1695.4	2274.5	**1.33**	2.20E-12
GXYLT1	glucoside xylosyltransferase 1 [Source:HGNC Symbol;Acc:HGNC:27482]	1087.0	1446.4	**1.33**	2.21E-12
RNF13	ring finger protein 13 [Source:HGNC Symbol;Acc:HGNC:10057]	3337.2	4441.9	**1.33**	1.91E-11
JMJD6	arginine demethylase and lysine hydroxylase [Source:HGNC Symbol;Acc:HGNC:19355]	541.3	724.1	**1.33**	1.21E-10
HMBOX1	homeobox containing 1 [Source:HGNC Symbol;Acc:HGNC:26137]	459.1	611.0	**1.33**	8.96E-10
SDHAF2	succinate dehydrogenase complex assembly factor 2 [Source:HGNC Symbol;Acc:HGNC:26034]	369.1	492.4	**1.33**	1.57E-09
ZNF845	zinc finger protein 845 [Source:HGNC Symbol;Acc:HGNC:25112]	377.7	500.8	**1.33**	2.92E-09
C1RL	complement C1r subcomponent like [Source:HGNC Symbol;Acc:HGNC:21265]	961.5	1281.1	**1.33**	9.34E-09
FYN	FYN proto-oncogene, Src family tyrosine kinase [Source:HGNC Symbol;Acc:HGNC:4037]	275.6	364.1	**1.33**	2.48E-06
WHAMM	WAS protein homolog associated with actin, golgi membranes and microtubules [Source:HGNC Symbol;	455.3	609.6	**1.33**	2.57E-06
GRAMD2B	GRAM domain containing 2B [Source:HGNC Symbol;Acc:HGNC:24911]	315.4	421.4	**1.33**	4.42E-06
MFSD6	major facilitator superfamily domain containing 6 [Source:HGNC Symbol;Acc:HGNC:24711]	260.2	345.9	**1.33**	4.54E-06
DHX37	DEAH-box helicase 37 [Source:HGNC Symbol;Acc:HGNC:17210]	207.2	276.2	**1.33**	1.17E-05
MAN2A2	mannosidase alpha class 2A member 2 [Source:HGNC Symbol;Acc:HGNC:6825]	525.8	710.4	**1.33**	1.50E-05
TTLL4	tubulin tyrosine ligase like 4 [Source:HGNC Symbol;Acc:HGNC:28976]	153.8	204.4	**1.33**	2.59E-05
TTC39C	tetratricopeptide repeat domain 39C [Source:HGNC Symbol;Acc:HGNC:26595]	199.1	265.6	**1.33**	4.82E-05
C9orf72	chromosome 9 open reading frame 72 [Source:HGNC Symbol;Acc:HGNC:28337]	250.9	337.3	**1.33**	6.06E-05
WDR81	WD repeat domain 81 [Source:HGNC Symbol;Acc:HGNC:26600]	115.2	153.9	**1.33**	0.0002821
PEX16	peroxisomal biogenesis factor 16 [Source:HGNC Symbol;Acc:HGNC:8857]	126.8	170.1	**1.33**	0.0126
DNAH6	dynein axonemal heavy chain 6 [Source:HGNC Symbol;Acc:HGNC:2951]	75.3	100.9	**1.33**	0.01971
CFAP126	cilia and flagella associated protein 126 [Source:HGNC Symbol;Acc:HGNC:32325]	92.1	126.1	**1.33**	0.02159
FANK1	fibronectin type III and ankyrin repeat domains 1 [Source:HGNC Symbol;Acc:HGNC:23527]	41.8	56.5	**1.33**	0.03085
SNCA	synuclein alpha [Source:HGNC Symbol;Acc:HGNC:11138]	50.8	70.3	**1.33**	0.03226
GPR75	G protein-coupled receptor 75 [Source:HGNC Symbol;Acc:HGNC:4526]	54.7	75.2	**1.33**	0.03748
PCYT1A	phosphate cytidylyltransferase 1, choline, alpha [Source:HGNC Symbol;Acc:HGNC:8754]	3402.7	4494.3	**1.32**	3.23E-32
MVP	major vault protein [Source:HGNC Symbol;Acc:HGNC:7531]	2458.7	3260.2	**1.32**	9.33E-29
CUL4A	cullin 4A [Source:HGNC Symbol;Acc:HGNC:2554]	3024.9	4003.6	**1.32**	6.18E-25
SEC23IP	SEC23 interacting protein [Source:HGNC Symbol;Acc:HGNC:17018]	2513.5	3317.7	**1.32**	8.48E-22
SNX13	sorting nexin 13 [Source:HGNC Symbol;Acc:HGNC:21335]	2493.7	3298.2	**1.32**	2.34E-21
RDX	radixin [Source:HGNC Symbol;Acc:HGNC:9944]	5159.0	6821.5	**1.32**	4.90E-21
ZNF622	zinc finger protein 622 [Source:HGNC Symbol;Acc:HGNC:30958]	1121.3	1484.1	**1.32**	9.62E-19
MGAT1	mannosyl (alpha-1,3-)-glycoprotein beta-1,2-N-acetylglucosaminyltransferase [Source:HGNC Symbol;Ac	966.6	1276.9	**1.32**	5.04E-18
CCNY	cyclin Y [Source:HGNC Symbol;Acc:HGNC:23354]	2134.5	2829.6	**1.32**	8.28E-17
SGK3	serum/glucocorticoid regulated kinase family member 3 [Source:HGNC Symbol;Acc:HGNC:10812]	1013.0	1336.7	**1.32**	8.85E-17
TSC1	TSC complex subunit 1 [Source:HGNC Symbol;Acc:HGNC:12362]	1042.6	1384.4	**1.32**	1.58E-16
NPEPPS	aminopeptidase puromycin sensitive [Source:HGNC Symbol;Acc:HGNC:7900]	1526.9	2024.2	**1.32**	1.96E-14
DPYD	dihydropyrimidine dehydrogenase [Source:HGNC Symbol;Acc:HGNC:3012]	1163.3	1535.6	**1.32**	5.32E-14
TJP1	tight junction protein 1 [Source:HGNC Symbol;Acc:HGNC:11827]	1441.8	1913.6	**1.32**	6.54E-14
ARPC4	actin related protein 2/3 complex subunit 4 [Source:HGNC Symbol;Acc:HGNC:707]	1174.3	1552.3	**1.32**	2.89E-13
UBE2B	ubiquitin conjugating enzyme E2 B [Source:HGNC Symbol;Acc:HGNC:12473]	619.6	822.3	**1.32**	7.70E-12
CDKN1A	cyclin dependent kinase inhibitor 1A [Source:HGNC Symbol;Acc:HGNC:1784]	833.1	1106.5	**1.32**	2.15E-10
ATP10D	ATPase phospholipid transporting 10D (putative) [Source:HGNC Symbol;Acc:HGNC:13549]	1025.4	1359.1	**1.32**	3.91E-10
SGPL1	sphingosine-1-phosphate lyase 1 [Source:HGNC Symbol;Acc:HGNC:10817]	702.0	927.7	**1.32**	1.67E-09
RAB35	RAB35, member RAS oncogene family [Source:HGNC Symbol;Acc:HGNC:9774]	480.3	635.6	**1.32**	2.67E-09
USP31	ubiquitin specific peptidase 31 [Source:HGNC Symbol;Acc:HGNC:20060]	451.2	597.0	**1.32**	8.73E-09
HECTD4	HECT domain E3 ubiquitin protein ligase 4 [Source:HGNC Symbol;Acc:HGNC:26611]	446.3	594.6	**1.32**	3.13E-08
ROMO1	reactive oxygen species modulator 1 [Source:HGNC Symbol;Acc:HGNC:16185]	394.0	519.2	**1.32**	1.07E-07
AIDA	axin interactor, dorsalization associated [Source:HGNC Symbol;Acc:HGNC:25761]	776.1	1032.3	**1.32**	2.47E-06
LIN52	lin-52 DREAM MuvB core complex component [Source:HGNC Symbol;Acc:HGNC:19856]	325.4	430.5	**1.32**	0.0001149
SAMD9	sterile alpha motif domain containing 9 [Source:HGNC Symbol;Acc:HGNC:1348]	663.0	863.5	**1.32**	0.0002813
ESR1	estrogen receptor 1 [Source:HGNC Symbol;Acc:HGNC:3467]	318.5	424.6	**1.32**	0.0004129
INTS9	integrator complex subunit 9 [Source:HGNC Symbol;Acc:HGNC:25592]	128.3	170.5	**1.32**	0.0009423
RNF135	ring finger protein 135 [Source:HGNC Symbol;Acc:HGNC:21158]	152.5	204.2	**1.32**	0.002313
GCAT	glycine C-acetyltransferase [Source:HGNC Symbol;Acc:HGNC:4188]	129.5	171.9	**1.32**	0.002652
STAG3L5P	stromal antigen 3-like 5 pseudogene [Source:HGNC Symbol;Acc:HGNC:48896]	114.9	151.9	**1.32**	0.00319
GBP1	guanylate binding protein 1 [Source:HGNC Symbol;Acc:HGNC:4182]	82.0	106.8	**1.32**	0.01296
SATB2	SATB homeobox 2 [Source:HGNC Symbol;Acc:HGNC:21637]	79.3	104.2	**1.32**	0.0254

PLAGL2	PLAG1 like zinc finger 2 [Source:HGNC Symbol;Acc:HGNC:9047]	51.0	67.8	**1.32**	0.04787
SLA2	Src like adaptor 2 [Source:HGNC Symbol;Acc:HGNC:17329]	44.3	58.8	**1.32**	0.04904
TRAPPC8	trafficking protein particle complex 8 [Source:HGNC Symbol;Acc:HGNC:29169]	1607.6	2110.2	**1.31**	1.26E-23
HLA-E	major histocompatibility complex, class I, E [Source:HGNC Symbol;Acc:HGNC:4962]	3334.5	4384.1	**1.31**	3.31E-23
VAPA	VAMP associated protein A [Source:HGNC Symbol;Acc:HGNC:12648]	3005.7	3947.8	**1.31**	8.39E-21
TMEM184C	transmembrane protein 184C [Source:HGNC Symbol;Acc:HGNC:25587]	1233.8	1624.9	**1.31**	2.33E-17
TPT1	tumor protein, translationally-controlled 1 [Source:HGNC Symbol;Acc:HGNC:12022]	22087.8	29020.0	**1.31**	2.99E-15
ITSN2	intersectin 2 [Source:HGNC Symbol;Acc:HGNC:6184]	2015.6	2650.5	**1.31**	8.35E-15
CRLS1	cardiolipin synthase 1 [Source:HGNC Symbol;Acc:HGNC:16148]	1192.5	1567.4	**1.31**	2.34E-14
OXSR1	oxidative stress responsive 1 [Source:HGNC Symbol;Acc:HGNC:8508]	2101.9	2767.7	**1.31**	1.19E-13
CREBRF	CREB3 regulatory factor [Source:HGNC Symbol;Acc:HGNC:24050]	2100.7	2789.5	**1.31**	8.99E-12
GABPB2	GA binding protein transcription factor subunit beta 2 [Source:HGNC Symbol;Acc:HGNC:28441]	866.6	1138.5	**1.31**	3.53E-11
KAT2B	lysine acetyltransferase 2B [Source:HGNC Symbol;Acc:HGNC:8638]	854.9	1122.4	**1.31**	1.36E-10
IGBP1	immunoglobulin binding protein 1 [Source:HGNC Symbol;Acc:HGNC:5461]	979.2	1295.1	**1.31**	1.97E-10
ATG16L1	autophagy related 16 like 1 [Source:HGNC Symbol;Acc:HGNC:21498]	590.0	775.1	**1.31**	8.03E-10
RNF149	ring finger protein 149 [Source:HGNC Symbol;Acc:HGNC:23137]	764.3	1001.9	**1.31**	2.14E-09
SLC9B2	solute carrier family 9 member B2 [Source:HGNC Symbol;Acc:HGNC:25143]	735.2	965.7	**1.31**	3.22E-09
USP4	ubiquitin specific peptidase 4 [Source:HGNC Symbol;Acc:HGNC:12627]	595.0	786.1	**1.31**	3.89E-08
TRAF3IP2	TRAF3 interacting protein 2 [Source:HGNC Symbol;Acc:HGNC:1343]	563.1	743.8	**1.31**	1.29E-07
APBB1	amyloid beta precursor protein binding family B member 1 [Source:HGNC Symbol;Acc:HGNC:581]	371.1	489.4	**1.31**	6.68E-07
GYG1	glycogenin 1 [Source:HGNC Symbol;Acc:HGNC:4699]	896.7	1185.8	**1.31**	1.18E-06
DUSP16	dual specificity phosphatase 16 [Source:HGNC Symbol;Acc:HGNC:17909]	272.4	360.0	**1.31**	1.73E-06
SYT11	synaptotagmin 11 [Source:HGNC Symbol;Acc:HGNC:19239]	286.9	381.4	**1.31**	2.28E-06
STARD13	StAR related lipid transfer domain containing 13 [Source:HGNC Symbol;Acc:HGNC:19164]	955.5	1265.3	**1.31**	1.03E-05
FPGS	folylpolyglutamate synthase [Source:HGNC Symbol;Acc:HGNC:3824]	250.9	329.3	**1.31**	5.22E-05
ARHGEF28	Rho guanine nucleotide exchange factor 28 [Source:HGNC Symbol;Acc:HGNC:30322]	424.7	572.6	**1.31**	8.55E-05
ZNF816	zinc finger protein 816 [Source:HGNC Symbol;Acc:HGNC:26995]	184.5	241.5	**1.31**	0.0001037
AMMECR1	Alport syndrome, mental retardation, midface hypoplasia and elliptocytosis chromosomal region gene 1	211.2	277.3	**1.31**	0.0002584
GIPC1	GIPC PDZ domain containing family member 1 [Source:HGNC Symbol;Acc:HGNC:1226]	239.7	312.2	**1.31**	0.0002765
RPL10P16	ribosomal protein L10 pseudogene 16 [Source:HGNC Symbol;Acc:HGNC:36882]	107.4	141.5	**1.31**	0.001245
SYNGR2	synaptogyrin 2 [Source:HGNC Symbol;Acc:HGNC:11499]	188.2	243.6	**1.31**	0.00128
ACSS3	acyl-CoA synthetase short chain family member 3 [Source:HGNC Symbol;Acc:HGNC:24723]	185.8	245.7	**1.31**	0.003536
GPX1P1	glutathione peroxidase pseudogene 1 [Source:HGNC Symbol;Acc:HGNC:4560]	134.5	179.2	**1.31**	0.004736
C4orf36	chromosome 4 open reading frame 36 [Source:HGNC Symbol;Acc:HGNC:28386]	112.4	148.3	**1.31**	0.004936
FJX1	four jointed box 1 [Source:HGNC Symbol;Acc:HGNC:17166]	111.8	148.6	**1.31**	0.005184
SDF2L1	stromal cell derived factor 2 like 1 [Source:HGNC Symbol;Acc:HGNC:10676]	146.4	192.3	**1.31**	0.006865
AGT	angiotensinogen [Source:HGNC Symbol;Acc:HGNC:333]	141.0	191.5	**1.31**	0.007069
SFXN2	sideroflexin 2 [Source:HGNC Symbol;Acc:HGNC:16086]	121.3	161.9	**1.31**	0.007651
MTATP6P1	mitochondrially encoded ATP synthase 6 pseudogene 1 [Source:HGNC Symbol;Acc:HGNC:44575]	98.6	131.2	**1.31**	0.01065
CMYA5	cardiomyopathy associated 5 [Source:HGNC Symbol;Acc:HGNC:14305]	139.5	189.1	**1.31**	0.01838
GTF2A1L	general transcription factor IIA subunit 1 like [Source:HGNC Symbol;Acc:HGNC:30727]	76.2	100.3	**1.31**	0.02839
MTCL1	microtubule crosslinking factor 1 [Source:HGNC Symbol;Acc:HGNC:29121]	130.6	182.8	**1.31**	0.02865
ADGRV1	adhesion G protein-coupled receptor V1 [Source:HGNC Symbol;Acc:HGNC:17416]	76.2	101.6	**1.31**	0.02878
FAM221A	family with sequence similarity 221 member A [Source:HGNC Symbol;Acc:HGNC:27977]	45.8	60.9	**1.31**	0.03468
ARID5A	AT-rich interaction domain 5A [Source:HGNC Symbol;Acc:HGNC:17361]	52.2	69.1	**1.31**	0.04568
TMEM50A	transmembrane protein 50A [Source:HGNC Symbol;Acc:HGNC:30590]	2250.0	2934.4	**1.30**	4.66E-22
SRI	sorcin [Source:HGNC Symbol;Acc:HGNC:11292]	1607.2	2094.7	**1.30**	1.80E-19
RPL36AL	ribosomal protein L36a like [Source:HGNC Symbol;Acc:HGNC:10346]	3338.6	4351.8	**1.30**	3.39E-19
FAM208B	family with sequence similarity 208 member B [Source:HGNC Symbol;Acc:HGNC:23484]	3310.9	4334.5	**1.30**	1.27E-17
RALBP1	ralA binding protein 1 [Source:HGNC Symbol;Acc:HGNC:9841]	3415.4	4485.4	**1.30**	1.14E-15
VPS4B	vacuolar protein sorting 4 homolog B [Source:HGNC Symbol;Acc:HGNC:10895]	1915.5	2494.7	**1.30**	1.66E-14
SEC23B	Sec23 homolog B, coat complex II component [Source:HGNC Symbol;Acc:HGNC:10702]	2328.1	3036.2	**1.30**	7.21E-14
CARHSP1	calcium regulated heat stable protein 1 [Source:HGNC Symbol;Acc:HGNC:17150]	718.8	943.0	**1.30**	1.24E-13
SLC11A2	solute carrier family 11 member 2 [Source:HGNC Symbol;Acc:HGNC:10908]	1172.2	1533.9	**1.30**	4.03E-13
P2RX5-TAX1BP3	P2RX5-TAX1BP3 readthrough (NMD candidate) [Source:HGNC Symbol;Acc:HGNC:49191]	664.1	865.8	**1.30**	9.60E-12
PREB	prolactin regulatory element binding [Source:HGNC Symbol;Acc:HGNC:9356]	728.2	953.1	**1.30**	1.00E-11
AATF	apoptosis antagonizing transcription factor [Source:HGNC Symbol;Acc:HGNC:19235]	1339.9	1730.0	**1.30**	1.78E-11
ABCC4	ATP binding cassette subfamily C member 4 [Source:HGNC Symbol;Acc:HGNC:55]	1256.4	1641.6	**1.30**	1.03E-10
MTMR4	myotubularin related protein 4 [Source:HGNC Symbol;Acc:HGNC:7452]	593.3	775.4	**1.30**	1.52E-10
C22orf39	chromosome 22 open reading frame 39 [Source:HGNC Symbol;Acc:HGNC:27012]	425.9	554.7	**1.30**	1.01E-09
AGTPBP1	ATP/GTP binding protein 1 [Source:HGNC Symbol;Acc:HGNC:17258]	808.4	1058.4	**1.30**	8.93E-09
PRCP	prolylcarboxypeptidase [Source:HGNC Symbol;Acc:HGNC:9344]	1179.4	1550.3	**1.30**	1.38E-08
IRF2	interferon regulatory factor 2 [Source:HGNC Symbol;Acc:HGNC:6117]	410.9	535.1	**1.30**	3.28E-08
CCND1	cyclin D1 [Source:HGNC Symbol;Acc:HGNC:1582]	13088.5	16459.3	**1.30**	4.34E-08
TRIM35	tripartite motif containing 35 [Source:HGNC Symbol;Acc:HGNC:16285]	503.2	656.7	**1.30**	2.89E-07
LYRM1	LYR motif containing 1 [Source:HGNC Symbol;Acc:HGNC:25074]	398.9	517.3	**1.30**	2.94E-07
CPSF7	cleavage and polyadenylation specific factor 7 [Source:HGNC Symbol;Acc:HGNC:30098]	470.8	617.9	**1.30**	4.13E-07
C20orf194	chromosome 20 open reading frame 194 [Source:HGNC Symbol;Acc:HGNC:17721]	381.1	497.5	**1.30**	9.88E-07
GTF2IRD1P1	GTF2I repeat domain containing 1 pseudogene 1 [Source:HGNC Symbol;Acc:HGNC:44136]	395.9	514.6	**1.30**	1.07E-06
KIAA1324L	KIAA1324 like [Source:HGNC Symbol;Acc:HGNC:21945]	455.3	587.7	**1.30**	1.81E-06
ZNF277	zinc finger protein 277 [Source:HGNC Symbol;Acc:HGNC:13070]	489.5	644.6	**1.30**	4.20E-06
ZBED4	zinc finger BED-type containing 4 [Source:HGNC Symbol;Acc:HGNC:20721]	311.9	407.9	**1.30**	4.74E-06

TRIM14	tripartite motif containing 14 [Source:HGNC Symbol;Acc:HGNC:16283]	535.3	691.9	**1.30**	5.55E-06
CABIN1	calcineurin binding protein 1 [Source:HGNC Symbol;Acc:HGNC:24187]	414.0	539.4	**1.30**	6.54E-06
BAG1	BCL2 associated athanogene 1 [Source:HGNC Symbol;Acc:HGNC:937]	775.5	1014.0	**1.30**	7.55E-06
CTPS2	CTP synthase 2 [Source:HGNC Symbol;Acc:HGNC:2520]	223.6	290.0	**1.30**	2.95E-05
TMEM30B	transmembrane protein 30B [Source:HGNC Symbol;Acc:HGNC:27254]	492.0	643.1	**1.30**	3.44E-05
TRMT9B	tRNA methyltransferase 9B (putative) [Source:HGNC Symbol;Acc:HGNC:26725]	257.3	341.1	**1.30**	4.46E-05
LBR	lamin B receptor [Source:HGNC Symbol;Acc:HGNC:6518]	369.4	481.1	**1.30**	6.70E-05
ELMOD3	ELMO domain containing 3 [Source:HGNC Symbol;Acc:HGNC:26158]	220.7	286.9	**1.30**	0.0002922
TOB1	transducer of ERBB2, 1 [Source:HGNC Symbol;Acc:HGNC:11979]	409.0	539.1	**1.30**	0.0006323
TRAPPC9	trafficking protein particle complex 9 [Source:HGNC Symbol;Acc:HGNC:30832]	146.0	190.2	**1.30**	0.0006977
KIAA0825	KIAA0825 [Source:HGNC Symbol;Acc:HGNC:28532]	166.6	218.3	**1.30**	0.001745
DDT	D-dopachrome tautomerase [Source:HGNC Symbol;Acc:HGNC:2732]	118.7	153.6	**1.30**	0.00198
FAM234B	family with sequence similarity 234 member B [Source:HGNC Symbol;Acc:HGNC:29288]	131.3	173.2	**1.30**	0.002721
MIGA2	mitoguardin 2 [Source:HGNC Symbol;Acc:HGNC:23621]	122.5	160.9	**1.30**	0.003922
EIF4BP7	eukaryotic translation initiation factor 4B pseudogene 7 [Source:HGNC Symbol;Acc:HGNC:37940]	80.7	104.5	**1.30**	0.004974
TACSTD2	tumor associated calcium signal transducer 2 [Source:HGNC Symbol;Acc:HGNC:11530]	96.8	127.9	**1.30**	0.006836
PLEKHJ1	pleckstrin homology domain containing J1 [Source:HGNC Symbol;Acc:HGNC:18211]	103.5	134.5	**1.30**	0.007055
NME5	NME/NM23 family member 5 [Source:HGNC Symbol;Acc:HGNC:7853]	99.9	132.0	**1.30**	0.01693
ARRDC1	arrestin domain containing 1 [Source:HGNC Symbol;Acc:HGNC:28633]	91.5	120.0	**1.30**	0.01712
KCND3	potassium voltage-gated channel subfamily D member 3 [Source:HGNC Symbol;Acc:HGNC:6239]	58.3	77.9	**1.30**	0.01947
BMS1P4	BMS1, ribosome biogenesis factor pseudogene 4 [Source:HGNC Symbol;Acc:HGNC:23652]	125.6	150.5	**1.30**	0.02508
USP35	ubiquitin specific peptidase 35 [Source:HGNC Symbol;Acc:HGNC:20061]	102.5	129.8	**1.30**	0.02899
ZNF763	zinc finger protein 763 [Source:HGNC Symbol;Acc:HGNC:27614]	68.6	90.8	**1.30**	0.02971
DNAH12	dynein axonemal heavy chain 12 [Source:HGNC Symbol;Acc:HGNC:2943]	53.5	71.2	**1.30**	0.0403
CYBRD1	cytochrome b reductase 1 [Source:HGNC Symbol;Acc:HGNC:20797]	12400.4	16067.7	**1.29**	1.75E-32
ETF1	eukaryotic translation termination factor 1 [Source:HGNC Symbol;Acc:HGNC:3477]	4028.9	5201.8	**1.29**	1.63E-28
TRIP12	thyroid hormone receptor interactor 12 [Source:HGNC Symbol;Acc:HGNC:12306]	7442.9	9659.7	**1.29**	1.69E-27
GLT8D1	glycosyltransferase 8 domain containing 1 [Source:HGNC Symbol;Acc:HGNC:24870]	1206.1	1561.2	**1.29**	1.46E-20
NAP1L1	nucleosome assembly protein 1 like 1 [Source:HGNC Symbol;Acc:HGNC:7637]	10576.6	13715.7	**1.29**	1.77E-20
AMD1	adenosylmethionine decarboxylase 1 [Source:HGNC Symbol;Acc:HGNC:457]	3546.9	4599.4	**1.29**	2.57E-19
CAMSAP2	calmodulin regulated spectrin associated protein family member 2 [Source:HGNC Symbol;Acc:HGNC:29	3078.3	3965.2	**1.29**	1.27E-17
PARP4	poly(ADP-ribose) polymerase family member 4 [Source:HGNC Symbol;Acc:HGNC:271]	2230.3	2889.9	**1.29**	2.75E-17
MYL6	myosin light chain 6 [Source:HGNC Symbol;Acc:HGNC:7587]	14998.2	19315.0	**1.29**	3.44E-17
PPM1A	protein phosphatase, Mg2+/Mn2+ dependent 1A [Source:HGNC Symbol;Acc:HGNC:9275]	1800.7	2334.1	**1.29**	1.34E-16
DICER1	dicer 1, ribonuclease III [Source:HGNC Symbol;Acc:HGNC:17098]	2629.8	3398.9	**1.29**	2.74E-16
DDX27	DEAD-box helicase 27 [Source:HGNC Symbol;Acc:HGNC:15837]	1098.2	1425.6	**1.29**	1.28E-13
ATP11B	ATPase phospholipid transporting 11B (putative) [Source:HGNC Symbol;Acc:HGNC:13553]	3116.3	4031.1	**1.29**	1.52E-13
TEAD1	TEA domain transcription factor 1 [Source:HGNC Symbol;Acc:HGNC:11714]	1770.4	2293.1	**1.29**	2.57E-13
CACUL1	CDK2 associated cullin domain 1 [Source:HGNC Symbol;Acc:HGNC:23727]	1356.3	1755.6	**1.29**	4.49E-13
RABGEF1	RAB guanine nucleotide exchange factor 1 [Source:HGNC Symbol;Acc:HGNC:17676]	2183.1	2834.4	**1.29**	2.60E-12
ARPC4-TTLL3	ARPC4-TTLL3 readthrough [Source:HGNC Symbol;Acc:HGNC:38830]	968.1	1257.6	**1.29**	1.16E-11
DHCR24	24-dehydrocholesterol reductase [Source:HGNC Symbol;Acc:HGNC:2859]	3077.8	3988.9	**1.29**	2.92E-11
TRIM13	tripartite motif containing 13 [Source:HGNC Symbol;Acc:HGNC:9976]	1107.9	1436.4	**1.29**	4.84E-10
BAZ2B	bromodomain adjacent to zinc finger domain 2B [Source:HGNC Symbol;Acc:HGNC:963]	758.8	981.1	**1.29**	5.33E-10
TAX1BP3	Tax1 binding protein 3 [Source:HGNC Symbol;Acc:HGNC:30684]	474.1	610.3	**1.29**	3.38E-09
COMP	cartilage oligomeric matrix protein [Source:HGNC Symbol;Acc:HGNC:2227]	13676.8	17180.4	**1.29**	1.29E-08
SLC51A	solute carrier family 51 alpha subunit [Source:HGNC Symbol;Acc:HGNC:29955]	378.4	492.0	**1.29**	1.69E-08
SLC25A51	solute carrier family 25 member 51 [Source:HGNC Symbol;Acc:HGNC:23323]	338.0	435.0	**1.29**	2.19E-08
ZNF347	zinc finger protein 347 [Source:HGNC Symbol;Acc:HGNC:16447]	440.7	571.2	**1.29**	2.59E-08
TBC1D12	TBC1 domain family member 12 [Source:HGNC Symbol;Acc:HGNC:29082]	590.2	762.9	**1.29**	4.22E-08
KLHL42	kelch like family member 42 [Source:HGNC Symbol;Acc:HGNC:29252]	875.6	1134.1	**1.29**	5.57E-08
SBF1	SET binding factor 1 [Source:HGNC Symbol;Acc:HGNC:10542]	544.7	702.3	**1.29**	3.70E-07
HPS1	HPS1, biogenesis of lysosomal organelles complex 3 subunit 1 [Source:HGNC Symbol;Acc:HGNC:5163]	253.9	327.4	**1.29**	5.77E-06
MED20	mediator complex subunit 20 [Source:HGNC Symbol;Acc:HGNC:16840]	213.9	276.7	**1.29**	9.68E-06
CHD7	chromodomain helicase DNA binding protein 7 [Source:HGNC Symbol;Acc:HGNC:20626]	330.9	426.0	**1.29**	1.29E-05
L3MBTL3	L3MBTL3, histone methyl-lysine binding protein [Source:HGNC Symbol;Acc:HGNC:23035]	278.6	358.4	**1.29**	1.53E-05
DPP9	dipeptidyl peptidase 9 [Source:HGNC Symbol;Acc:HGNC:18648]	387.5	500.8	**1.29**	1.65E-05
SETD6	SET domain containing 6 [Source:HGNC Symbol;Acc:HGNC:26116]	348.1	452.3	**1.29**	2.76E-05
SYNJ1	synaptojanin 1 [Source:HGNC Symbol;Acc:HGNC:11503]	254.4	331.3	**1.29**	5.66E-05
CCDC32	coiled-coil domain containing 32 [Source:HGNC Symbol;Acc:HGNC:28295]	298.7	386.3	**1.29**	5.89E-05
ZNF836	zinc finger protein 836 [Source:HGNC Symbol;Acc:HGNC:34333]	241.5	311.7	**1.29**	5.99E-05
IFITM3	interferon induced transmembrane protein 3 [Source:HGNC Symbol;Acc:HGNC:5414]	410.7	534.4	**1.29**	6.95E-05
WDR37	WD repeat domain 37 [Source:HGNC Symbol;Acc:HGNC:31406]	321.8	415.1	**1.29**	8.43E-05
COL4A2	collagen type IV alpha 2 chain [Source:HGNC Symbol;Acc:HGNC:2203]	308.0	401.5	**1.29**	0.0002431
ZNF783	zinc finger family member 783 [Source:HGNC Symbol;Acc:HGNC:27222]	174.5	227.3	**1.29**	0.0002557
TRIP4	thyroid hormone receptor interactor 4 [Source:HGNC Symbol;Acc:HGNC:12310]	290.2	374.3	**1.29**	0.0002852
ASMTL	acetylserotonin O-methyltransferase like [Source:HGNC Symbol;Acc:HGNC:751]	238.9	313.8	**1.29**	0.0006035
FAM234A	family with sequence similarity 234 member A [Source:HGNC Symbol;Acc:HGNC:14163]	229.9	297.7	**1.29**	0.0007656
IMPDH1	inosine monophosphate dehydrogenase 1 [Source:HGNC Symbol;Acc:HGNC:6052]	332.9	431.7	**1.29**	0.001125
CYB561D1	cytochrome b561 family member D1 [Source:HGNC Symbol;Acc:HGNC:26804]	134.8	175.5	**1.29**	0.001851
CFAP69	cilia and flagella associated protein 69 [Source:HGNC Symbol;Acc:HGNC:26107]	110.2	144.0	**1.29**	0.004031
KBTBD3	kelch repeat and BTB domain containing 3 [Source:HGNC Symbol;Acc:HGNC:22934]	146.5	191.9	**1.29**	0.004692

ZBTB48	zinc finger and BTB domain containing 48 [Source:HGNC Symbol;Acc:HGNC:4930]	70.3	91.1	**1.29**	0.01286
CHP1	calcineurin like EF-hand protein 1 [Source:HGNC Symbol;Acc:HGNC:17433]	1375.3	1771.2	**1.28**	4.84E-19
NMT1	N-myristoyltransferase 1 [Source:HGNC Symbol;Acc:HGNC:7857]	2182.3	2798.4	**1.28**	3.69E-18
FNTA	farnesyltransferase, CAAX box, alpha [Source:HGNC Symbol;Acc:HGNC:3782]	1549.7	1987.3	**1.28**	2.75E-15
PLPBP	pyridoxal phosphate binding protein [Source:HGNC Symbol;Acc:HGNC:9457]	1009.4	1297.3	**1.28**	1.04E-14
ATP6V1D	ATPase H+ transporting V1 subunit D [Source:HGNC Symbol;Acc:HGNC:13527]	1138.1	1461.1	**1.28**	9.93E-14
OGFOD1	2-oxoglutarate and iron dependent oxygenase domain containing 1 [Source:HGNC Symbol;Acc:HGNC:25	1023.2	1310.9	**1.28**	4.81E-12
GMFB	glia maturation factor beta [Source:HGNC Symbol;Acc:HGNC:4373]	3247.0	4168.2	**1.28**	5.19E-12
RBM7	RNA binding motif protein 7 [Source:HGNC Symbol;Acc:HGNC:9904]	755.9	967.8	**1.28**	2.03E-11
PAQR3	progestin and adipoQ receptor family member 3 [Source:HGNC Symbol;Acc:HGNC:30130]	703.5	902.4	**1.28**	3.61E-11
HADHB	hydroxyacyl-CoA dehydrogenase trifunctional multienzyme complex subunit beta [Source:HGNC Symbo	2070.6	2661.3	**1.28**	5.00E-11
RIC1	RIC1 homolog, RAB6A GEF complex partner 1 [Source:HGNC Symbol;Acc:HGNC:17686]	684.7	883.8	**1.28**	6.26E-10
VPS26C	VPS26 endosomal protein sorting factor C [Source:HGNC Symbol;Acc:HGNC:3044]	1164.6	1497.9	**1.28**	7.39E-10
VTI1A	vesicle transport through interaction with t-SNAREs 1A [Source:HGNC Symbol;Acc:HGNC:17792]	739.0	951.4	**1.28**	8.69E-10
CIZ1	CDKN1A interacting zinc finger protein 1 [Source:HGNC Symbol;Acc:HGNC:16744]	812.5	1043.1	**1.28**	1.88E-09
FNIP2	folliculin interacting protein 2 [Source:HGNC Symbol;Acc:HGNC:29280]	2104.2	2696.7	**1.28**	3.06E-09
MGAT4B	mannosyl (alpha-1,3-)-glycoprotein beta-1,4-N-acetylglucosaminyltransferase, isozyme B [Source:HGNC	721.8	936.5	**1.28**	9.97E-08
TMEM246	transmembrane protein 246 [Source:HGNC Symbol;Acc:HGNC:28180]	360.4	463.1	**1.28**	3.82E-07
HSPA1A	heat shock protein family A (Hsp70) member 1A [Source:HGNC Symbol;Acc:HGNC:5232]	1034.1	1326.3	**1.28**	8.21E-07
SMYD2	SET and MYND domain containing 2 [Source:HGNC Symbol;Acc:HGNC:20982]	462.5	594.5	**1.28**	1.44E-06
BRMS1	breast cancer metastasis suppressor 1 [Source:HGNC Symbol;Acc:HGNC:17262]	389.6	501.3	**1.28**	2.06E-06
CHFR	checkpoint with forkhead and ring finger domains [Source:HGNC Symbol;Acc:HGNC:20455]	370.5	474.9	**1.28**	4.08E-06
MOB3C	MOB kinase activator 3C [Source:HGNC Symbol;Acc:HGNC:29800]	232.0	298.7	**1.28**	5.53E-06
ZNF641	zinc finger protein 641 [Source:HGNC Symbol;Acc:HGNC:31834]	518.8	667.2	**1.28**	7.55E-06
PIWIL4	piwi like RNA-mediated gene silencing 4 [Source:HGNC Symbol;Acc:HGNC:18444]	446.4	575.5	**1.28**	2.16E-05
CPEB2	cytoplasmic polyadenylation element binding protein 2 [Source:HGNC Symbol;Acc:HGNC:21745]	371.8	487.4	**1.28**	6.96E-05
TSEN2	tRNA splicing endonuclease subunit 2 [Source:HGNC Symbol;Acc:HGNC:28422]	285.5	370.3	**1.28**	7.85E-05
ZFYVE1	zinc finger FYVE-type containing 1 [Source:HGNC Symbol;Acc:HGNC:13180]	238.6	305.4	**1.28**	9.77E-05
FAH	fumarylacetoacetate hydrolase [Source:HGNC Symbol;Acc:HGNC:3579]	231.4	298.9	**1.28**	0.0001609
PGM2L1	phosphoglucomutase 2 like 1 [Source:HGNC Symbol;Acc:HGNC:20898]	1036.0	1354.7	**1.28**	0.0001872
CTIF	cap binding complex dependent translation initiation factor [Source:HGNC Symbol;Acc:HGNC:23925]	295.7	380.0	**1.28**	0.0002576
BCAS3	BCAS3, microtubule associated cell migration factor [Source:HGNC Symbol;Acc:HGNC:14347]	247.6	322.9	**1.28**	0.0002654
SNAI2	snail family transcriptional repressor 2 [Source:HGNC Symbol;Acc:HGNC:11094]	1702.7	2227.1	**1.28**	0.0002785
DIRAS1	DIRAS family GTPase 1 [Source:HGNC Symbol;Acc:HGNC:19127]	146.0	186.9	**1.28**	0.001021
CUTALP	cutA divalent cation tolerance homolog-like, pseudogene [Source:HGNC Symbol;Acc:HGNC:27367]	223.3	288.6	**1.28**	0.001115
SERAC1	serine active site containing 1 [Source:HGNC Symbol;Acc:HGNC:21061]	221.7	283.1	**1.28**	0.002044
TRIM3	tripartite motif containing 3 [Source:HGNC Symbol;Acc:HGNC:10064]	99.3	127.7	**1.28**	0.007276
NOTCH2NL	notch 2 N-terminal like [Source:HGNC Symbol;Acc:HGNC:31862]	150.2	194.8	**1.28**	0.01352
MIF4GD	MIF4G domain containing [Source:HGNC Symbol;Acc:HGNC:24030]	86.7	112.2	**1.28**	0.01927
ADD1	adducin 1 [Source:HGNC Symbol;Acc:HGNC:243]	3442.8	4381.5	**1.27**	7.68E-26
SPART	spartin [Source:HGNC Symbol;Acc:HGNC:18514]	3493.5	4462.5	**1.27**	1.46E-24
PHF3	PHD finger protein 3 [Source:HGNC Symbol;Acc:HGNC:8921]	4979.4	6348.0	**1.27**	8.34E-20
VAMP3	vesicle associated membrane protein 3 [Source:HGNC Symbol;Acc:HGNC:12644]	2157.4	2753.8	**1.27**	2.07E-19
ZC3HAV1	zinc finger CCCH-type containing, antiviral 1 [Source:HGNC Symbol;Acc:HGNC:23721]	2143.8	2738.2	**1.27**	8.81E-16
CLCC1	chloride channel CLIC like 1 [Source:HGNC Symbol;Acc:HGNC:29675]	2789.8	3553.8	**1.27**	5.80E-15
PCNX4	pecanex homolog 4 [Source:HGNC Symbol;Acc:HGNC:20349]	2685.7	3432.3	**1.27**	5.57E-14
SENP2	SUMO specific peptidase 2 [Source:HGNC Symbol;Acc:HGNC:23116]	1276.5	1634.3	**1.27**	1.06E-13
RBM19	RNA binding motif protein 19 [Source:HGNC Symbol;Acc:HGNC:29098]	758.7	964.7	**1.27**	5.11E-13
AUP1	AUP1, lipid droplet regulating VLDL assembly factor [Source:HGNC Symbol;Acc:HGNC:891]	831.7	1062.5	**1.27**	1.10E-10
HECTD2	HECT domain E3 ubiquitin protein ligase 2 [Source:HGNC Symbol;Acc:HGNC:26736]	1011.3	1292.0	**1.27**	3.97E-10
KHDC4	KH domain containing 4, pre-mRNA splicing factor [Source:HGNC Symbol;Acc:HGNC:29145]	616.6	787.5	**1.27**	5.79E-10
LDHA	lactate dehydrogenase A [Source:HGNC Symbol;Acc:HGNC:6535]	31343.9	40239.1	**1.27**	2.56E-09
MTMR3	myotubularin related protein 3 [Source:HGNC Symbol;Acc:HGNC:7451]	893.4	1141.6	**1.27**	6.96E-09
MED21	mediator complex subunit 21 [Source:HGNC Symbol;Acc:HGNC:11473]	1004.5	1282.4	**1.27**	1.19E-08
SMURF1	SMAD specific E3 ubiquitin protein ligase 1 [Source:HGNC Symbol;Acc:HGNC:16807]	660.1	839.9	**1.27**	1.94E-08
LZTR1	leucine zipper like transcription regulator 1 [Source:HGNC Symbol;Acc:HGNC:6742]	572.4	728.7	**1.27**	1.99E-08
UNC13B	unc-13 homolog B [Source:HGNC Symbol;Acc:HGNC:12566]	957.9	1220.7	**1.27**	2.36E-08
FAM129B	family with sequence similarity 129 member B [Source:HGNC Symbol;Acc:HGNC:25282]	5632.8	7215.4	**1.27**	6.42E-08
PDP1	pyruvate dehyrogenase phosphatase catalytic subunit 1 [Source:HGNC Symbol;Acc:HGNC:9279]	998.4	1270.0	**1.27**	6.83E-08
AAED1	AhpC/TSA antioxidant enzyme domain containing 1 [Source:HGNC Symbol;Acc:HGNC:16881]	429.1	548.8	**1.27**	3.52E-07
SLC38A6	solute carrier family 38 member 6 [Source:HGNC Symbol;Acc:HGNC:19863]	950.0	1213.3	**1.27**	4.28E-07
SDF2	stromal cell derived factor 2 [Source:HGNC Symbol;Acc:HGNC:10675]	490.3	624.9	**1.27**	5.96E-07
ZFHX3	zinc finger homeobox 3 [Source:HGNC Symbol;Acc:HGNC:777]	697.1	892.0	**1.27**	1.63E-06
RFLNB	refilin B [Source:HGNC Symbol;Acc:HGNC:28705]	674.3	871.1	**1.27**	2.32E-06
IKBKB	inhibitor of nuclear factor kappa B kinase subunit beta [Source:HGNC Symbol;Acc:HGNC:5960]	365.1	468.2	**1.27**	4.56E-06
ZBTB34	zinc finger and BTB domain containing 34 [Source:HGNC Symbol;Acc:HGNC:31446]	269.3	344.0	**1.27**	7.54E-06
TECPR2	tectonin beta-propeller repeat containing 2 [Source:HGNC Symbol;Acc:HGNC:19957]	319.0	411.2	**1.27**	2.91E-05
ZNF600	zinc finger protein 600 [Source:HGNC Symbol;Acc:HGNC:30951]	289.2	367.2	**1.27**	3.39E-05
PAPPA	pappalysin 1 [Source:HGNC Symbol;Acc:HGNC:8602]	1064.4	1372.3	**1.27**	4.21E-05
STAT5A	signal transducer and activator of transcription 5A [Source:HGNC Symbol;Acc:HGNC:11366]	474.0	613.5	**1.27**	5.72E-05
NOA1	nitric oxide associated 1 [Source:HGNC Symbol;Acc:HGNC:28473]	276.1	355.2	**1.27**	0.0001255
SS18L2	SS18 like 2 [Source:HGNC Symbol;Acc:HGNC:15593]	182.9	234.4	**1.27**	0.0001266

SLC35F6	solute carrier family 35 member F6 [Source:HGNC Symbol;Acc:HGNC:26055]	469.9	598.9	**1.27**	0.0001355
TMEM164	transmembrane protein 164 [Source:HGNC Symbol;Acc:HGNC:26217]	228.6	294.2	**1.27**	0.000153
NFKBIB	NFKB inhibitor beta [Source:HGNC Symbol;Acc:HGNC:7798]	184.6	234.8	**1.27**	0.0001608
BAHD1	bromo adjacent homology domain containing 1 [Source:HGNC Symbol;Acc:HGNC:29153]	175.8	226.7	**1.27**	0.002897
TMEM186	transmembrane protein 186 [Source:HGNC Symbol;Acc:HGNC:24530]	135.9	173.2	**1.27**	0.003136
SGSM2	small G protein signaling modulator 2 [Source:HGNC Symbol;Acc:HGNC:29026]	203.0	265.2	**1.27**	0.004318
TRANK1	tetratricopeptide repeat and ankyrin repeat containing 1 [Source:HGNC Symbol;Acc:HGNC:29011]	262.2	336.6	**1.27**	0.005575
WDFY3-AS1	WDFY3 antisense RNA 1 [Source:HGNC Symbol;Acc:HGNC:40935]	83.4	107.1	**1.27**	0.01853
KRT8P12	keratin 8 pseudogene 12 [Source:HGNC Symbol;Acc:HGNC:28057]	94.7	120.5	**1.27**	0.02375
IPO5P1	importin 5 pseudogene 1 [Source:HGNC Symbol;Acc:HGNC:49687]	84.7	108.5	**1.27**	0.04114
DDX21	DExD-box helicase 21 [Source:HGNC Symbol;Acc:HGNC:2744]	5487.2	6963.0	**1.27**	4.15E-19
ESYT2	extended synaptotagmin 2 [Source:HGNC Symbol;Acc:HGNC:22211]	5952.3	7548.0	**1.27**	2.24E-15
GULP1	GULP, engulfment adaptor PTB domain containing 1 [Source:HGNC Symbol;Acc:HGNC:18649]	2012.3	2552.0	**1.27**	4.90E-13
MYL6B	myosin light chain 6B [Source:HGNC Symbol;Acc:HGNC:29823]	5131.3	6469.3	**1.27**	6.00E-13
RPL13	ribosomal protein L13 [Source:HGNC Symbol;Acc:HGNC:10303]	4375.5	5563.0	**1.27**	1.00E-12
PTPRA	protein tyrosine phosphatase, receptor type A [Source:HGNC Symbol;Acc:HGNC:9664]	1750.0	2216.4	**1.27**	4.43E-12
RNF115	ring finger protein 115 [Source:HGNC Symbol;Acc:HGNC:18154]	1289.6	1635.8	**1.27**	6.89E-12
NDEL1	nudE neurodevelopment protein 1 like 1 [Source:HGNC Symbol;Acc:HGNC:17620]	1788.8	2264.0	**1.27**	7.50E-12
SPDYA	speedy/RINGO cell cycle regulator family member A [Source:HGNC Symbol;Acc:HGNC:30613]	4006.7	5069.4	**1.27**	7.90E-12
QKI	QKI, KH domain containing RNA binding [Source:HGNC Symbol;Acc:HGNC:21100]	3243.3	4116.7	**1.27**	1.39E-11
KCTD7	potassium channel tetramerization domain containing 7 [Source:HGNC Symbol;Acc:HGNC:21957]	2334.3	2974.4	**1.27**	1.91E-11
KIF16B	kinesin family member 16B [Source:HGNC Symbol;Acc:HGNC:15869]	911.7	1155.6	**1.27**	2.28E-10
GSR	glutathione-disulfide reductase [Source:HGNC Symbol;Acc:HGNC:4623]	840.0	1066.6	**1.27**	2.89E-10
AFDN	afadin, adherens junction formation factor [Source:HGNC Symbol;Acc:HGNC:7137]	2656.4	3368.1	**1.27**	4.87E-10
LTN1	listerin E3 ubiquitin protein ligase 1 [Source:HGNC Symbol;Acc:HGNC:13082]	2258.6	2859.2	**1.27**	6.77E-10
PLEC	plectin [Source:HGNC Symbol;Acc:HGNC:9069]	16296.3	20885.6	**1.27**	6.81E-10
DDAH1	dimethylarginine dimethylaminohydrolase 1 [Source:HGNC Symbol;Acc:HGNC:2715]	4597.3	5843.5	**1.27**	7.86E-10
CNOT1	CCR4-NOT transcription complex subunit 1 [Source:HGNC Symbol;Acc:HGNC:7877]	2820.4	3585.1	**1.27**	2.53E-09
SLC25A24	solute carrier family 25 member 24 [Source:HGNC Symbol;Acc:HGNC:20662]	2004.0	2531.4	**1.27**	5.02E-09
RASA2	RAS p21 protein activator 2 [Source:HGNC Symbol;Acc:HGNC:9872]	638.1	808.9	**1.27**	7.56E-09
EOGT	EGF domain specific O-linked N-acetylglucosamine transferase [Source:HGNC Symbol;Acc:HGNC:28526]	835.8	1056.3	**1.27**	7.94E-09
CADPS2	calcium dependent secretion activator 2 [Source:HGNC Symbol;Acc:HGNC:16018]	565.8	720.1	**1.27**	8.37E-09
CDC42SE1	CDC42 small effector 1 [Source:HGNC Symbol;Acc:HGNC:17719]	2117.7	2688.8	**1.27**	9.13E-09
DLST	dihydrolipoamide S-succinyltransferase [Source:HGNC Symbol;Acc:HGNC:2911]	1070.3	1360.6	**1.27**	2.95E-08
DNAJB9	DnaJ heat shock protein family (Hsp40) member B9 [Source:HGNC Symbol;Acc:HGNC:6968]	2552.9	3255.5	**1.27**	5.53E-08
PARD3	par-3 family cell polarity regulator [Source:HGNC Symbol;Acc:HGNC:16051]	688.4	878.6	**1.27**	6.87E-08
AREL1	apoptosis resistant E3 ubiquitin protein ligase 1 [Source:HGNC Symbol;Acc:HGNC:20363]	579.0	732.2	**1.27**	8.44E-08
WIPI2	WD repeat domain, phosphoinositide interacting 2 [Source:HGNC Symbol;Acc:HGNC:32225]	650.2	824.9	**1.27**	9.35E-08
DCAF10	DDB1 and CUL4 associated factor 10 [Source:HGNC Symbol;Acc:HGNC:23686]	691.3	877.0	**1.27**	2.19E-07
MT-CO2	mitochondrially encoded cytochrome c oxidase II [Source:HGNC Symbol;Acc:HGNC:7421]	1983.3	2500.3	**1.27**	3.52E-07
TRIO	trio Rho guanine nucleotide exchange factor [Source:HGNC Symbol;Acc:HGNC:12303]	2729.0	3490.7	**1.27**	5.07E-07
TCTN2	tectonic family member 2 [Source:HGNC Symbol;Acc:HGNC:25774]	560.2	713.6	**1.27**	1.40E-06
IP6K1	inositol hexakisphosphate kinase 1 [Source:HGNC Symbol;Acc:HGNC:18360]	462.7	587.3	**1.27**	1.67E-06
ORAOV1	oral cancer overexpressed 1 [Source:HGNC Symbol;Acc:HGNC:17589]	1553.7	1920.9	**1.27**	2.00E-06
ABHD18	abhydrolase domain containing 18 [Source:HGNC Symbol;Acc:HGNC:26111]	278.7	352.7	**1.27**	2.29E-06
SGPP1	sphingosine-1-phosphate phosphatase 1 [Source:HGNC Symbol;Acc:HGNC:17720]	960.4	1217.8	**1.27**	4.64E-06
GPATCH8	G-patch domain containing 8 [Source:HGNC Symbol;Acc:HGNC:29066]	697.1	887.0	**1.27**	4.83E-06
STIM1	stromal interaction molecule 1 [Source:HGNC Symbol;Acc:HGNC:11386]	494.2	628.7	**1.27**	9.71E-06
PRRG1	proline rich and Gla domain 1 [Source:HGNC Symbol;Acc:HGNC:9469]	362.8	458.6	**1.27**	1.05E-05
KANK2	KN motif and ankyrin repeat domains 2 [Source:HGNC Symbol;Acc:HGNC:29300]	1521.7	1978.8	**1.27**	1.28E-05
CHMP1A	charged multivesicular body protein 1A [Source:HGNC Symbol;Acc:HGNC:8740]	356.2	453.6	**1.27**	1.53E-05
DCAF16	DDB1 and CUL4 associated factor 16 [Source:HGNC Symbol;Acc:HGNC:25987]	428.9	548.8	**1.27**	2.03E-05
LINC00963	long intergenic non-protein coding RNA 963 [Source:HGNC Symbol;Acc:HGNC:48716]	342.0	434.7	**1.27**	3.44E-05
KDM7A	lysine demethylase 7A [Source:HGNC Symbol;Acc:HGNC:22224]	2347.7	3014.0	**1.27**	4.69E-05
RRAGB	Ras related GTP binding B [Source:HGNC Symbol;Acc:HGNC:19901]	233.4	295.0	**1.27**	4.81E-05
ARHGAP23	Rho GTPase activating protein 23 [Source:HGNC Symbol;Acc:HGNC:29293]	373.1	475.3	**1.27**	5.47E-05
ZNF287	zinc finger protein 287 [Source:HGNC Symbol;Acc:HGNC:13502]	180.0	228.5	**1.27**	7.11E-05
PLAGL1	PLAG1 like zinc finger 1 [Source:HGNC Symbol;Acc:HGNC:9046]	604.2	760.4	**1.27**	0.0001003
GON7	GON7, KEOPS complex subunit [Source:HGNC Symbol;Acc:HGNC:20356]	209.5	265.2	**1.27**	0.000126
SCARNA7	small Cajal body-specific RNA 7 [Source:HGNC Symbol;Acc:HGNC:32563]	635.1	801.1	**1.27**	0.0001537
LDHAP4	lactate dehydrogenase A pseudogene 4 [Source:HGNC Symbol;Acc:HGNC:6539]	296.7	383.0	**1.27**	0.0002238
GTPBP6	GTP binding protein 6 (putative) [Source:HGNC Symbol;Acc:HGNC:30189]	331.9	420.6	**1.27**	0.0002918
ZSCAN21	zinc finger and SCAN domain containing 21 [Source:HGNC Symbol;Acc:HGNC:13104]	232.3	296.7	**1.27**	0.0005006
R3HDM4	R3H domain containing 4 [Source:HGNC Symbol;Acc:HGNC:28270]	130.5	165.3	**1.27**	0.00132
ZNF69	zinc finger protein 69 [Source:HGNC Symbol;Acc:HGNC:13138]	131.0	165.5	**1.27**	0.007702
TBC1D25	TBC1 domain family member 25 [Source:HGNC Symbol;Acc:HGNC:8092]	82.4	104.5	**1.27**	0.009742
DENND2C	DENN domain containing 2C [Source:HGNC Symbol;Acc:HGNC:24748]	149.8	191.6	**1.27**	0.01078
AP5Z1	adaptor related protein complex 5 subunit zeta 1 [Source:HGNC Symbol;Acc:HGNC:22197]	110.6	141.3	**1.27**	0.01156
EPS15L1	epidermal growth factor receptor pathway substrate 15 like 1 [Source:HGNC Symbol;Acc:HGNC:24634]	190.0	241.2	**1.27**	0.01292
TYW1B	tRNA-yW synthesizing protein 1 homolog B [Source:HGNC Symbol;Acc:HGNC:33908]	116.9	142.7	**1.27**	0.01997
RPL22P1	ribosomal protein L22 pseudogene 1 [Source:HGNC Symbol;Acc:HGNC:17998]	61.0	78.0	**1.27**	0.04984
PIGN	phosphatidylinositol glycan anchor biosynthesis class N [Source:HGNC Symbol;Acc:HGNC:8967]	1808.5	2278.9	**1.26**	2.35E-15

ANKRD27	ankyrin repeat domain 27 [Source:HGNC Symbol;Acc:HGNC:25310]	1149.1	1444.0	**1.26**	8.02E-15
BAZ1A	bromodomain adjacent to zinc finger domain 1A [Source:HGNC Symbol;Acc:HGNC:960]	1331.1	1671.0	**1.26**	1.97E-14
DCAF6	DDB1 and CUL4 associated factor 6 [Source:HGNC Symbol;Acc:HGNC:30002]	2459.8	3102.7	**1.26**	3.50E-14
SNX12	sorting nexin 12 [Source:HGNC Symbol;Acc:HGNC:14976]	993.4	1245.1	**1.26**	3.54E-14
WDR11	WD repeat domain 11 [Source:HGNC Symbol;Acc:HGNC:13831]	1871.5	2365.1	**1.26**	3.64E-14
MAP3K4	mitogen-activated protein kinase kinase kinase 4 [Source:HGNC Symbol;Acc:HGNC:6856]	1512.8	1908.2	**1.26**	2.73E-12
LGALS3	galectin 3 [Source:HGNC Symbol;Acc:HGNC:6563]	4973.2	6270.2	**1.26**	1.33E-11
SLC26A2	solute carrier family 26 member 2 [Source:HGNC Symbol;Acc:HGNC:10994]	5863.6	7419.7	**1.26**	1.61E-11
LARP1	La ribonucleoprotein domain family member 1 [Source:HGNC Symbol;Acc:HGNC:29531]	2594.3	3254.1	**1.26**	1.54E-10
LETM1	leucine zipper and EF-hand containing transmembrane protein 1 [Source:HGNC Symbol;Acc:HGNC:6556	1000.3	1257.8	**1.26**	1.84E-10
RABGAP1L	RAB GTPase activating protein 1 like [Source:HGNC Symbol;Acc:HGNC:24663]	1480.8	1856.0	**1.26**	5.94E-10
HMGN4	high mobility group nucleosomal binding domain 4 [Source:HGNC Symbol;Acc:HGNC:4989]	870.7	1090.5	**1.26**	8.33E-10
SMAD4	SMAD family member 4 [Source:HGNC Symbol;Acc:HGNC:6770]	2179.8	2753.9	**1.26**	1.20E-09
MPC1	mitochondrial pyruvate carrier 1 [Source:HGNC Symbol;Acc:HGNC:21606]	622.1	781.1	**1.26**	2.41E-09
SVIL	supervillin [Source:HGNC Symbol;Acc:HGNC:11480]	2865.1	3599.0	**1.26**	8.25E-09
ATXN1L	ataxin 1 like [Source:HGNC Symbol;Acc:HGNC:33279]	715.3	899.2	**1.26**	9.36E-09
EIF2B4	eukaryotic translation initiation factor 2B subunit delta [Source:HGNC Symbol;Acc:HGNC:3260]	519.8	656.7	**1.26**	4.58E-08
TRIM38	tripartite motif containing 38 [Source:HGNC Symbol;Acc:HGNC:10059]	696.8	876.0	**1.26**	6.41E-08
SVIL-AS1	SVIL antisense RNA 1 [Source:HGNC Symbol;Acc:HGNC:51219]	759.9	954.6	**1.26**	7.10E-08
TOP1	DNA topoisomerase I [Source:HGNC Symbol;Acc:HGNC:11986]	762.6	962.8	**1.26**	8.03E-08
WDR78	WD repeat domain 78 [Source:HGNC Symbol;Acc:HGNC:26252]	996.9	1245.9	**1.26**	2.51E-07
CTSB	cathepsin B [Source:HGNC Symbol;Acc:HGNC:2527]	8160.8	10392.0	**1.26**	5.65E-07
TRAPPC10	trafficking protein particle complex 10 [Source:HGNC Symbol;Acc:HGNC:11868]	389.6	490.4	**1.26**	6.21E-07
RRAS2	RAS related 2 [Source:HGNC Symbol;Acc:HGNC:17271]	1139.4	1438.6	**1.26**	1.08E-06
DOCK11	dedicator of cytokinesis 11 [Source:HGNC Symbol;Acc:HGNC:23483]	641.3	808.1	**1.26**	1.29E-06
MARCH5	membrane associated ring-CH-type finger 5 [Source:HGNC Symbol;Acc:HGNC:26025]	802.0	1015.0	**1.26**	1.38E-06
C5orf22	chromosome 5 open reading frame 22 [Source:HGNC Symbol;Acc:HGNC:25639]	462.8	581.7	**1.26**	1.74E-05
PQLC3	PQ loop repeat containing 3 [Source:HGNC Symbol;Acc:HGNC:28503]	396.0	498.3	**1.26**	1.90E-05
ZNF281	zinc finger protein 281 [Source:HGNC Symbol;Acc:HGNC:13075]	463.8	587.2	**1.26**	2.05E-05
CDIPT	CDP-diacylglycerol--inositol 3-phosphatidyltransferase [Source:HGNC Symbol;Acc:HGNC:1769]	448.8	565.6	**1.26**	2.50E-05
NOP53	NOP53 ribosome biogenesis factor [Source:HGNC Symbol;Acc:HGNC:4333]	878.1	1121.7	**1.26**	2.82E-05
KLHL29	kelch like family member 29 [Source:HGNC Symbol;Acc:HGNC:29404]	370.7	467.0	**1.26**	2.90E-05
TPRA1	transmembrane protein adipocyte associated 1 [Source:HGNC Symbol;Acc:HGNC:30413]	374.6	471.1	**1.26**	3.70E-05
RNF38	ring finger protein 38 [Source:HGNC Symbol;Acc:HGNC:18052]	330.2	415.3	**1.26**	4.51E-05
ZNF28	zinc finger protein 28 [Source:HGNC Symbol;Acc:HGNC:13073]	477.0	601.4	**1.26**	5.64E-05
KLHL21	kelch like family member 21 [Source:HGNC Symbol;Acc:HGNC:29041]	927.2	1156.1	**1.26**	6.51E-05
BAZ2A	bromodomain adjacent to zinc finger domain 2A [Source:HGNC Symbol;Acc:HGNC:962]	676.6	853.9	**1.26**	6.62E-05
MYO6	myosin VI [Source:HGNC Symbol;Acc:HGNC:7605]	2004.3	2559.0	**1.26**	6.84E-05
TLE3	transducin like enhancer of split 3 [Source:HGNC Symbol;Acc:HGNC:11839]	459.0	582.6	**1.26**	0.0001193
GPX1	glutathione peroxidase 1 [Source:HGNC Symbol;Acc:HGNC:4553]	433.2	557.2	**1.26**	0.0001814
RUBCN	RUN and cysteine rich domain containing beclin 1 interacting protein [Source:HGNC Symbol;Acc:HGNC:2	242.8	306.7	**1.26**	3.00E-04
NADSYN1	NAD synthetase 1 [Source:HGNC Symbol;Acc:HGNC:29832]	300.7	381.7	**1.26**	0.0003769
MORN2	MORN repeat containing 2 [Source:HGNC Symbol;Acc:HGNC:30166]	337.6	427.8	**1.26**	0.0003923
C1orf56	chromosome 1 open reading frame 56 [Source:HGNC Symbol;Acc:HGNC:26045]	376.7	475.8	**1.26**	0.0005005
TFE3	transcription factor binding to IGHM enhancer 3 [Source:HGNC Symbol;Acc:HGNC:11752]	360.1	453.4	**1.26**	0.0005955
PDLIM4	PDZ and LIM domain 4 [Source:HGNC Symbol;Acc:HGNC:16501]	263.4	333.5	**1.26**	0.0008286
TRIM52	tripartite motif containing 52 [Source:HGNC Symbol;Acc:HGNC:19024]	206.2	260.8	**1.26**	0.001293
GIT1	GIT ArfGAP 1 [Source:HGNC Symbol;Acc:HGNC:4272]	246.8	309.5	**1.26**	0.00261
NBPF14	NBPF member 14 [Source:HGNC Symbol;Acc:HGNC:25232]	302.8	381.4	**1.26**	0.00279
PGLS	6-phosphogluconolactonase [Source:HGNC Symbol;Acc:HGNC:8903]	163.4	204.5	**1.26**	0.006115
GCDH	glutaryl-CoA dehydrogenase [Source:HGNC Symbol;Acc:HGNC:4189]	193.2	243.9	**1.26**	0.00801
FAM53B	family with sequence similarity 53 member B [Source:HGNC Symbol;Acc:HGNC:28968]	168.6	211.5	**1.26**	0.00907
CAMKMT	calmodulin-lysine N-methyltransferase [Source:HGNC Symbol;Acc:HGNC:26276]	98.2	126.3	**1.26**	0.0173
GRK6	G protein-coupled receptor kinase 6 [Source:HGNC Symbol;Acc:HGNC:4545]	119.8	150.9	**1.26**	0.02707
RPL26P19	ribosomal protein L26 pseudogene 19 [Source:HGNC Symbol;Acc:HGNC:36393]	66.2	84.3	**1.26**	0.03664
RAB1A	RAB1A, member RAS oncogene family [Source:HGNC Symbol;Acc:HGNC:9758]	6767.3	8460.9	**1.25**	5.64E-29
DNAJC21	DnaJ heat shock protein family (Hsp40) member C21 [Source:HGNC Symbol;Acc:HGNC:27030]	2107.5	2638.2	**1.25**	2.57E-22
BNIP2	BCL2 interacting protein 2 [Source:HGNC Symbol;Acc:HGNC:1083]	4425.2	5528.5	**1.25**	2.24E-19
TMED10	transmembrane p24 trafficking protein 10 [Source:HGNC Symbol;Acc:HGNC:16998]	13490.4	16844.3	**1.25**	1.51E-17
ICE1	interactor of little elongation complex ELL subunit 1 [Source:HGNC Symbol;Acc:HGNC:29154]	1756.6	2190.1	**1.25**	5.29E-15
LAMP1	lysosomal associated membrane protein 1 [Source:HGNC Symbol;Acc:HGNC:6499]	6381.8	7971.7	**1.25**	5.34E-15
CEP350	centrosomal protein 350 [Source:HGNC Symbol;Acc:HGNC:24238]	3689.5	4597.2	**1.25**	1.19E-14
PPP1CB	protein phosphatase 1 catalytic subunit beta [Source:HGNC Symbol;Acc:HGNC:9282]	12106.8	15134.3	**1.25**	1.97E-14
SPCS3	signal peptidase complex subunit 3 [Source:HGNC Symbol;Acc:HGNC:26212]	4829.8	6022.1	**1.25**	6.69E-14
SLC17A5	solute carrier family 17 member 5 [Source:HGNC Symbol;Acc:HGNC:10933]	1297.2	1621.1	**1.25**	1.84E-13
UTP3	UTP3, small subunit processome component [Source:HGNC Symbol;Acc:HGNC:24477]	828.2	1035.7	**1.25**	4.93E-12
RAF1	Raf-1 proto-oncogene, serine/threonine kinase [Source:HGNC Symbol;Acc:HGNC:9829]	2340.1	2943.3	**1.25**	7.88E-12
TXN	thioredoxin [Source:HGNC Symbol;Acc:HGNC:12435]	3938.8	4910.9	**1.25**	8.51E-12
HERC3	HECT and RLD domain containing E3 ubiquitin protein ligase 3 [Source:HGNC Symbol;Acc:HGNC:4876]	2231.9	2785.9	**1.25**	2.76E-11
PKP4	plakophilin 4 [Source:HGNC Symbol;Acc:HGNC:9026]	1009.9	1261.2	**1.25**	1.03E-10
HEATR5B	HEAT repeat containing 5B [Source:HGNC Symbol;Acc:HGNC:29273]	1023.2	1276.2	**1.25**	1.46E-10
ZNF106	zinc finger protein 106 [Source:HGNC Symbol;Acc:HGNC:12886]	3282.2	4107.0	**1.25**	2.80E-10

C6orf106	chromosome 6 open reading frame 106 [Source:HGNC Symbol;Acc:HGNC:21215]	1421.4	1773.4	**1.25**	4.93E-10
RCAN3	RCAN family member 3 [Source:HGNC Symbol;Acc:HGNC:3042]	1001.8	1254.1	**1.25**	2.09E-09
MKRN2	makorin ring finger protein 2 [Source:HGNC Symbol;Acc:HGNC:7113]	1098.5	1378.6	**1.25**	2.61E-09
BBS7	Bardet-Biedl syndrome 7 [Source:HGNC Symbol;Acc:HGNC:18758]	1039.4	1302.8	**1.25**	4.19E-09
STX12	syntaxin 12 [Source:HGNC Symbol;Acc:HGNC:11430]	1182.5	1473.6	**1.25**	8.03E-09
RAC1	Rac family small GTPase 1 [Source:HGNC Symbol;Acc:HGNC:9801]	1981.1	2468.4	**1.25**	1.12E-08
ITPR2	inositol 1,4,5-trisphosphate receptor type 2 [Source:HGNC Symbol;Acc:HGNC:6181]	5450.8	6864.6	**1.25**	1.61E-08
HGS	hepatocyte growth factor-regulated tyrosine kinase substrate [Source:HGNC Symbol;Acc:HGNC:4897]	1057.9	1327.9	**1.25**	2.48E-07
AKAP8L	A-kinase anchoring protein 8 like [Source:HGNC Symbol;Acc:HGNC:29857]	630.5	790.3	**1.25**	1.08E-06
PON2	paraoxonase 2 [Source:HGNC Symbol;Acc:HGNC:9205]	719.7	903.9	**1.25**	3.08E-06
TTC26	tetratricopeptide repeat domain 26 [Source:HGNC Symbol;Acc:HGNC:21882]	515.6	645.7	**1.25**	3.18E-06
ZNF274	zinc finger protein 274 [Source:HGNC Symbol;Acc:HGNC:13068]	366.6	460.9	**1.25**	3.32E-06
SMG1	SMG1, nonsense mediated mRNA decay associated PI3K related kinase [Source:HGNC Symbol;Acc:HGN(	2594.1	3227.7	**1.25**	3.80E-06
ZNF331	zinc finger protein 331 [Source:HGNC Symbol;Acc:HGNC:15489]	471.0	588.5	**1.25**	4.49E-06
APAF1	apoptotic peptidase activating factor 1 [Source:HGNC Symbol;Acc:HGNC:576]	887.7	1104.8	**1.25**	4.82E-06
C1R	complement C1r [Source:HGNC Symbol;Acc:HGNC:1246]	2876.7	3594.9	**1.25**	8.97E-06
HMCES	5-hydroxymethylcytosine binding, ES cell specific [Source:HGNC Symbol;Acc:HGNC:24446]	443.4	552.6	**1.25**	1.31E-05
TMED8	transmembrane p24 trafficking protein family member 8 [Source:HGNC Symbol;Acc:HGNC:18633]	942.4	1173.0	**1.25**	2.43E-05
GORASP1	golgi reassembly stacking protein 1 [Source:HGNC Symbol;Acc:HGNC:16769]	269.6	338.1	**1.25**	4.90E-05
CYB561	cytochrome b561 [Source:HGNC Symbol;Acc:HGNC:2571]	272.6	339.8	**1.25**	0.0001124
RPS3AP26	ribosomal protein S3a pseudogene 26 [Source:HGNC Symbol;Acc:HGNC:36513]	421.9	529.4	**1.25**	0.0001454
ZNF471	zinc finger protein 471 [Source:HGNC Symbol;Acc:HGNC:23226]	209.3	261.5	**1.25**	0.0002526
EIF3J-DT	EIF3J divergent transcript [Source:HGNC Symbol;Acc:HGNC:48616]	1606.2	1645.1	**1.25**	0.0005048
WDTC1	WD and tetratricopeptide repeats 1 [Source:HGNC Symbol;Acc:HGNC:29175]	330.5	416.4	**1.25**	0.0006337
PSEN2	presenilin 2 [Source:HGNC Symbol;Acc:HGNC:9509]	266.0	336.8	**1.25**	0.0008692
ZER1	zyg-11 related cell cycle regulator [Source:HGNC Symbol;Acc:HGNC:30960]	227.3	286.1	**1.25**	0.001656
ARHGAP17	Rho GTPase activating protein 17 [Source:HGNC Symbol;Acc:HGNC:18239]	208.2	260.2	**1.25**	0.001908
PAPPA-AS1	PAPPA antisense RNA 1 [Source:HGNC Symbol;Acc:HGNC:35152]	318.9	396.8	**1.25**	0.002431
MKNK1	MAP kinase interacting serine/threonine kinase 1 [Source:HGNC Symbol;Acc:HGNC:7110]	203.8	252.9	**1.25**	0.002452
MAML1	mastermind like transcriptional coactivator 1 [Source:HGNC Symbol;Acc:HGNC:13632]	281.7	349.9	**1.25**	0.002995
XG	Xg glycoprotein (Xg blood group) [Source:HGNC Symbol;Acc:HGNC:12806]	170.9	216.9	**1.25**	0.003098
ARHGEF19	Rho guanine nucleotide exchange factor 19 [Source:HGNC Symbol;Acc:HGNC:26604]	148.6	187.0	**1.25**	0.005579
PQLC1	PQ loop repeat containing 1 [Source:HGNC Symbol;Acc:HGNC:26188]	156.0	195.7	**1.25**	0.005596
ZNF526	zinc finger protein 526 [Source:HGNC Symbol;Acc:HGNC:29415]	186.6	234.0	**1.25**	0.006063
ZNF34	zinc finger protein 34 [Source:HGNC Symbol;Acc:HGNC:13098]	109.6	137.3	**1.25**	0.01073
GTF2IP4	general transcription factor IIi pseudogene 4 [Source:HGNC Symbol;Acc:HGNC:51716]	170.9	216.5	**1.25**	0.01486
SLC16A10	solute carrier family 16 member 10 [Source:HGNC Symbol;Acc:HGNC:17027]	173.7	218.6	**1.25**	0.01561
MOB3A	MOB kinase activator 3A [Source:HGNC Symbol;Acc:HGNC:29802]	124.7	157.6	**1.25**	0.02512
SNAPC2	small nuclear RNA activating complex polypeptide 2 [Source:HGNC Symbol;Acc:HGNC:11135]	104.6	130.7	**1.25**	0.03069
FZD3	frizzled class receptor 3 [Source:HGNC Symbol;Acc:HGNC:4041]	124.0	152.9	**1.25**	0.04022
SMG1P3	SMG1 pseudogene 3 [Source:HGNC Symbol;Acc:HGNC:49860]	120.6	153.0	**1.25**	0.04178
XRN2	5'-3' exoribonuclease 2 [Source:HGNC Symbol;Acc:HGNC:12836]	3044.7	3785.6	**1.24**	2.15E-17
PITRM1	pitrilysin metallopeptidase 1 [Source:HGNC Symbol;Acc:HGNC:17663]	2573.6	3191.4	**1.24**	3.53E-15
SNX3	sorting nexin 3 [Source:HGNC Symbol;Acc:HGNC:11174]	3447.2	4276.3	**1.24**	2.91E-14
SSR3	signal sequence receptor subunit 3 [Source:HGNC Symbol;Acc:HGNC:11325]	12565.7	15529.5	**1.24**	3.50E-14
MAPK1	mitogen-activated protein kinase 1 [Source:HGNC Symbol;Acc:HGNC:6871]	2996.9	3729.5	**1.24**	5.91E-14
RNF40	ring finger protein 40 [Source:HGNC Symbol;Acc:HGNC:16867]	1106.5	1372.9	**1.24**	1.04E-12
DDHD2	DDHD domain containing 2 [Source:HGNC Symbol;Acc:HGNC:29106]	1578.0	1950.8	**1.24**	2.68E-12
SAV1	salvador family WW domain containing protein 1 [Source:HGNC Symbol;Acc:HGNC:17795]	1649.2	2038.7	**1.24**	3.45E-12
RANBP9	RAN binding protein 9 [Source:HGNC Symbol;Acc:HGNC:13727]	3292.7	4082.6	**1.24**	8.80E-12
SMC6	structural maintenance of chromosomes 6 [Source:HGNC Symbol;Acc:HGNC:20466]	1280.2	1593.3	**1.24**	1.03E-11
SLTM	SAFB like transcription modulator [Source:HGNC Symbol;Acc:HGNC:20709]	3746.0	4658.8	**1.24**	1.81E-11
FNIP1	folliculin interacting protein 1 [Source:HGNC Symbol;Acc:HGNC:29418]	2083.7	2575.4	**1.24**	2.93E-11
UHRF1BP1L	UHRF1 binding protein 1 like [Source:HGNC Symbol;Acc:HGNC:29102]	3145.2	3899.1	**1.24**	3.73E-11
SCYL2	SCY1 like pseudokinase 2 [Source:HGNC Symbol;Acc:HGNC:19286]	1539.7	1911.4	**1.24**	7.01E-11
PRDX6	peroxiredoxin 6 [Source:HGNC Symbol;Acc:HGNC:16753]	3031.8	3768.7	**1.24**	2.31E-10
USP25	ubiquitin specific peptidase 25 [Source:HGNC Symbol;Acc:HGNC:12624]	1885.7	2351.6	**1.24**	1.90E-09
USP9X	ubiquitin specific peptidase 9 X-linked [Source:HGNC Symbol;Acc:HGNC:12632]	6565.6	8139.1	**1.24**	6.01E-09
WASHC2C	WASH complex subunit 2C [Source:HGNC Symbol;Acc:HGNC:23414]	901.8	1122.9	**1.24**	7.03E-09
ALG2	ALG2, alpha-1,3/1,6-mannosyltransferase [Source:HGNC Symbol;Acc:HGNC:23159]	970.6	1208.5	**1.24**	8.22E-09
NTN4	netrin 4 [Source:HGNC Symbol;Acc:HGNC:13658]	14273.3	17886.1	**1.24**	9.02E-09
MYO1C	myosin IC [Source:HGNC Symbol;Acc:HGNC:7597]	2888.1	3568.0	**1.24**	2.02E-08
RCBTB1	RCC1 and BTB domain containing protein 1 [Source:HGNC Symbol;Acc:HGNC:18243]	626.4	778.4	**1.24**	2.55E-08
CD2AP	CD2 associated protein [Source:HGNC Symbol;Acc:HGNC:14258]	1360.1	1689.3	**1.24**	2.62E-08
DNAJB4	DnaJ heat shock protein family (Hsp40) member B4 [Source:HGNC Symbol;Acc:HGNC:14886]	1968.5	2444.1	**1.24**	4.04E-08
KIAA0355	KIAA0355 [Source:HGNC Symbol;Acc:HGNC:29016]	595.1	734.9	**1.24**	5.23E-08
MAP3K3	mitogen-activated protein kinase kinase kinase 3 [Source:HGNC Symbol;Acc:HGNC:6855]	817.6	1017.6	**1.24**	9.78E-08
ABCA5	ATP binding cassette subfamily A member 5 [Source:HGNC Symbol;Acc:HGNC:35]	2422.8	3014.3	**1.24**	2.38E-07
MR1	major histocompatibility complex, class I-related [Source:HGNC Symbol;Acc:HGNC:4975]	936.5	1161.3	**1.24**	3.07E-07
RAB33B	RAB33B, member RAS oncogene family [Source:HGNC Symbol;Acc:HGNC:16075]	769.1	955.5	**1.24**	3.58E-07
C12orf49	chromosome 12 open reading frame 49 [Source:HGNC Symbol;Acc:HGNC:26128]	850.1	1054.3	**1.24**	4.01E-07
RAB5C	RAB5C, member RAS oncogene family [Source:HGNC Symbol;Acc:HGNC:9785]	713.3	885.1	**1.24**	6.57E-07

ZNF480	zinc finger protein 480 [Source:HGNC Symbol;Acc:HGNC:23305]	546.1	675.0	**1.24**	8.01E-07
TAOK3	TAO kinase 3 [Source:HGNC Symbol;Acc:HGNC:18133]	811.2	1008.6	**1.24**	1.09E-06
FLOT2	flotillin 2 [Source:HGNC Symbol;Acc:HGNC:3758]	738.3	918.0	**1.24**	1.16E-06
MAML2	mastermind like transcriptional coactivator 2 [Source:HGNC Symbol;Acc:HGNC:16259]	657.2	821.0	**1.24**	1.19E-06
ZFC3H1	zinc finger C3H1-type containing [Source:HGNC Symbol;Acc:HGNC:28328]	832.4	1030.1	**1.24**	2.54E-06
HIST2H2AC	histone cluster 2 H2A family member c [Source:HGNC Symbol;Acc:HGNC:4738]	1449.6	1821.3	**1.24**	2.87E-06
STXBP4	syntaxin binding protein 4 [Source:HGNC Symbol;Acc:HGNC:19694]	393.6	488.7	**1.24**	6.61E-06
UBLCP1	ubiquitin like domain containing CTD phosphatase 1 [Source:HGNC Symbol;Acc:HGNC:28110]	597.7	738.7	**1.24**	8.36E-06
RARB	retinoic acid receptor beta [Source:HGNC Symbol;Acc:HGNC:9865]	1325.2	1662.6	**1.24**	1.60E-05
RNF185	ring finger protein 185 [Source:HGNC Symbol;Acc:HGNC:26783]	523.0	649.4	**1.24**	2.77E-05
SPEN	spen family transcriptional repressor [Source:HGNC Symbol;Acc:HGNC:17575]	1215.3	1504.8	**1.24**	3.37E-05
NOP16	NOP16 nucleolar protein [Source:HGNC Symbol;Acc:HGNC:26934]	312.9	387.1	**1.24**	4.03E-05
MMP10	matrix metallopeptidase 10 [Source:HGNC Symbol;Acc:HGNC:7156]	698.2	843.8	**1.24**	6.20E-05
GRB10	growth factor receptor bound protein 10 [Source:HGNC Symbol;Acc:HGNC:4564]	451.7	561.3	**1.24**	0.0001726
IGF2R	insulin like growth factor 2 receptor [Source:HGNC Symbol;Acc:HGNC:5467]	6766.2	8415.8	**1.24**	0.0001744
OTUB1	OTU deubiquitinase, ubiquitin aldehyde binding 1 [Source:HGNC Symbol;Acc:HGNC:23077]	308.9	383.5	**1.24**	0.000226
DDHD1	DDHD domain containing 1 [Source:HGNC Symbol;Acc:HGNC:19714]	370.9	459.0	**1.24**	0.000251
FOXD1	forkhead box D1 [Source:HGNC Symbol;Acc:HGNC:3802]	238.1	296.0	**1.24**	0.0004224
BBS1	Bardet-Biedl syndrome 1 [Source:HGNC Symbol;Acc:HGNC:966]	245.6	305.4	**1.24**	0.0005302
SPATA7	spermatogenesis associated 7 [Source:HGNC Symbol;Acc:HGNC:20423]	339.9	419.1	**1.24**	0.0005751
MT-ND4	mitochondrially encoded NADH:ubiquinone oxidoreductase core subunit 4 [Source:HGNC Symbol;Acc:H	1640.0	2090.4	**1.24**	0.0006159
GPR176	G protein-coupled receptor 176 [Source:HGNC Symbol;Acc:HGNC:32370]	383.9	481.9	**1.24**	0.0006213
SMNDC1	survival motor neuron domain containing 1 [Source:HGNC Symbol;Acc:HGNC:16900]	312.7	391.1	**1.24**	0.000897
YIPF1	Yip1 domain family member 1 [Source:HGNC Symbol;Acc:HGNC:25231]	263.8	329.1	**1.24**	0.001024
PLCD4	phospholipase C delta 4 [Source:HGNC Symbol;Acc:HGNC:9062]	200.5	248.9	**1.24**	0.001061
TBRG4	transforming growth factor beta regulator 4 [Source:HGNC Symbol;Acc:HGNC:17443]	243.1	299.1	**1.24**	0.001146
C1RL-AS1	C1RL antisense RNA 1 [Source:HGNC Symbol;Acc:HGNC:27461]	226.4	275.3	**1.24**	0.001577
CEP164P1	centrosomal protein 164 pseudogene 1 [Source:HGNC Symbol;Acc:HGNC:44988]	277.0	344.2	**1.24**	0.001717
TMEM115	transmembrane protein 115 [Source:HGNC Symbol;Acc:HGNC:30055]	191.1	236.8	**1.24**	0.00462
STAG3L5P-PVRIG2	STAG3L5P-PVRIG2P-PILRB readthrough [Source:HGNC Symbol;Acc:HGNC:48898]	219.5	270.6	**1.24**	0.004701
EME2	essential meiotic structure-specific endonuclease subunit 2 [Source:HGNC Symbol;Acc:HGNC:27289]	166.8	206.9	**1.24**	0.006402
ADM	adrenomedullin [Source:HGNC Symbol;Acc:HGNC:259]	1013.5	1283.1	**1.24**	0.007724
PARD3B	par-3 family cell polarity regulator beta [Source:HGNC Symbol;Acc:HGNC:14446]	204.9	258.9	**1.24**	0.01508
ZXDB	zinc finger X-linked duplicated B [Source:HGNC Symbol;Acc:HGNC:13199]	146.4	182.2	**1.24**	0.01598
PPDPF	pancreatic progenitor cell differentiation and proliferation factor [Source:HGNC Symbol;Acc:HGNC:1614	212.6	259.6	**1.24**	0.01607
CD14	CD14 molecule [Source:HGNC Symbol;Acc:HGNC:1628]	198.1	253.2	**1.24**	0.03373
BEX5	brain expressed X-linked 5 [Source:HGNC Symbol;Acc:HGNC:27990]	96.7	120.4	**1.24**	0.03764
MAATS1	MYCBP associated and testis expressed 1 [Source:HGNC Symbol;Acc:HGNC:24010]	86.7	106.7	**1.24**	0.04083
PBLD	phenazine biosynthesis like protein domain containing [Source:HGNC Symbol;Acc:HGNC:23301]	79.6	99.5	**1.24**	0.0448
ECPAS	Ecm29 proteasome adaptor and scaffold [Source:HGNC Symbol;Acc:HGNC:29020]	5580.7	6860.9	**1.23**	4.88E-17
MSL1	male specific lethal 1 homolog [Source:HGNC Symbol;Acc:HGNC:27905]	1305.2	1609.1	**1.23**	4.53E-15
RPS27	ribosomal protein S27 [Source:HGNC Symbol;Acc:HGNC:10416]	6039.2	7467.6	**1.23**	9.37E-14
EIF3G	eukaryotic translation initiation factor 3 subunit G [Source:HGNC Symbol;Acc:HGNC:3274]	1677.6	2068.1	**1.23**	3.61E-13
DDX17	DEAD-box helicase 17 [Source:HGNC Symbol;Acc:HGNC:2740]	10472.5	12984.4	**1.23**	3.78E-13
ALDH3A2	aldehyde dehydrogenase 3 family member A2 [Source:HGNC Symbol;Acc:HGNC:403]	2450.0	3017.0	**1.23**	3.81E-13
ARFGEF1	ADP ribosylation factor guanine nucleotide exchange factor 1 [Source:HGNC Symbol;Acc:HGNC:15772]	3200.8	3948.5	**1.23**	7.48E-13
SRP54	signal recognition particle 54 [Source:HGNC Symbol;Acc:HGNC:11301]	3928.3	4833.8	**1.23**	1.79E-12
DCTN1	dynactin subunit 1 [Source:HGNC Symbol;Acc:HGNC:2711]	3934.9	4865.8	**1.23**	4.19E-11
AGFG1	ArfGAP with FG repeats 1 [Source:HGNC Symbol;Acc:HGNC:5175]	1537.4	1896.7	**1.23**	4.39E-11
SHOC2	SHOC2, leucine rich repeat scaffold protein [Source:HGNC Symbol;Acc:HGNC:15454]	2908.7	3587.2	**1.23**	5.92E-11
ANKRD13C	ankyrin repeat domain 13C [Source:HGNC Symbol;Acc:HGNC:25374]	1514.2	1871.6	**1.23**	6.83E-11
RALB	RAS like proto-oncogene B [Source:HGNC Symbol;Acc:HGNC:9840]	2321.2	2859.6	**1.23**	9.11E-11
CTSZ	cathepsin Z [Source:HGNC Symbol;Acc:HGNC:2547]	1878.5	2322.7	**1.23**	1.44E-10
MYL12A	myosin light chain 12A [Source:HGNC Symbol;Acc:HGNC:16701]	7023.5	8607.2	**1.23**	1.54E-10
GPCPD1	glycerophosphocholine phosphodiesterase 1 [Source:HGNC Symbol;Acc:HGNC:26957]	1370.9	1688.9	**1.23**	1.73E-10
MAPK1IP1L	mitogen-activated protein kinase 1 interacting protein 1 like [Source:HGNC Symbol;Acc:HGNC:19840]	1415.5	1740.6	**1.23**	2.32E-10
PIGG	phosphatidylinositol glycan anchor biosynthesis class G [Source:HGNC Symbol;Acc:HGNC:25985]	1004.7	1232.8	**1.23**	7.40E-10
ATP13A3	ATPase 13A3 [Source:HGNC Symbol;Acc:HGNC:24113]	5194.2	6355.1	**1.23**	2.50E-09
PAK2	p21 (RAC1) activated kinase 2 [Source:HGNC Symbol;Acc:HGNC:8591]	1480.7	1829.6	**1.23**	3.81E-09
ANKRD12	ankyrin repeat domain 12 [Source:HGNC Symbol;Acc:HGNC:29135]	4555.1	5647.7	**1.23**	4.40E-09
VPS4A	vacuolar protein sorting 4 homolog A [Source:HGNC Symbol;Acc:HGNC:13488]	1199.7	1474.2	**1.23**	8.79E-09
MKRN1	makorin ring finger protein 1 [Source:HGNC Symbol;Acc:HGNC:7112]	1618.8	2000.9	**1.23**	9.26E-09
PAIP2	poly(A) binding protein interacting protein 2 [Source:HGNC Symbol;Acc:HGNC:17970]	2363.8	2921.0	**1.23**	1.43E-08
ZDHHC6	zinc finger DHHC-type containing 6 [Source:HGNC Symbol;Acc:HGNC:19160]	830.4	1021.9	**1.23**	1.78E-08
SNX25	sorting nexin 25 [Source:HGNC Symbol;Acc:HGNC:21883]	893.1	1096.8	**1.23**	3.19E-08
ABCF2	ATP binding cassette subfamily F member 2 [Source:HGNC Symbol;Acc:HGNC:71]	1681.4	2090.5	**1.23**	6.37E-08
DAZAP2	DAZ associated protein 2 [Source:HGNC Symbol;Acc:HGNC:2684]	1020.2	1257.1	**1.23**	1.62E-07
ZFAND6	zinc finger AN1-type containing 6 [Source:HGNC Symbol;Acc:HGNC:30164]	1343.9	1665.5	**1.23**	2.01E-07
RETREG3	reticulophagy regulator family member 3 [Source:HGNC Symbol;Acc:HGNC:27258]	1169.4	1447.4	**1.23**	4.14E-07
FOXP1	forkhead box P1 [Source:HGNC Symbol;Acc:HGNC:3823]	1902.7	2348.9	**1.23**	4.21E-07
FUT8	fucosyltransferase 8 [Source:HGNC Symbol;Acc:HGNC:4019]	935.4	1159.9	**1.23**	9.92E-07
SRFBP1	serum response factor binding protein 1 [Source:HGNC Symbol;Acc:HGNC:26333]	1060.0	1311.7	**1.23**	1.22E-06

STX2	syntaxin 2 [Source:HGNC Symbol;Acc:HGNC:3403]	1101.7	1358.5	**1.23**	1.23E-06
DGCR2	DiGeorge syndrome critical region gene 2 [Source:HGNC Symbol;Acc:HGNC:2845]	610.0	754.4	**1.23**	2.48E-06
ZNF267	zinc finger protein 267 [Source:HGNC Symbol;Acc:HGNC:13060]	492.5	605.1	**1.23**	7.70E-06
CAMKK2	calcium/calmodulin dependent protein kinase kinase 2 [Source:HGNC Symbol;Acc:HGNC:1470]	370.5	457.5	**1.23**	1.75E-05
GSTK1	glutathione S-transferase kappa 1 [Source:HGNC Symbol;Acc:HGNC:16906]	449.6	553.1	**1.23**	1.80E-05
RILPL2	Rab interacting lysosomal protein like 2 [Source:HGNC Symbol;Acc:HGNC:28787]	542.6	668.5	**1.23**	1.93E-05
PPP3CC	protein phosphatase 3 catalytic subunit gamma [Source:HGNC Symbol;Acc:HGNC:9316]	516.2	640.7	**1.23**	5.15E-05
CD302	CD302 molecule [Source:HGNC Symbol;Acc:HGNC:30843]	725.8	900.4	**1.23**	8.66E-05
TRAF6	TNF receptor associated factor 6 [Source:HGNC Symbol;Acc:HGNC:12036]	352.6	436.3	**1.23**	0.0001351
CARD6	caspase recruitment domain family member 6 [Source:HGNC Symbol;Acc:HGNC:16394]	365.8	449.7	**1.23**	0.0001521
NCOR2	nuclear receptor corepressor 2 [Source:HGNC Symbol;Acc:HGNC:7673]	557.8	693.3	**1.23**	0.0001546
SYCP3	synaptonemal complex protein 3 [Source:HGNC Symbol;Acc:HGNC:18130]	275.4	338.6	**1.23**	0.0002067
ZNF701	zinc finger protein 701 [Source:HGNC Symbol;Acc:HGNC:25597]	232.4	286.7	**1.23**	0.0005336
SRGAP2	SLIT-ROBO Rho GTPase activating protein 2 [Source:HGNC Symbol;Acc:HGNC:19751]	400.6	493.7	**1.23**	0.00055
IER5	immediate early response 5 [Source:HGNC Symbol;Acc:HGNC:5393]	425.1	526.4	**1.23**	0.000643
AKAP17A	A-kinase anchoring protein 17A [Source:HGNC Symbol;Acc:HGNC:18783]	422.0	518.1	**1.23**	0.000644
CIB1	calcium and integrin binding 1 [Source:HGNC Symbol;Acc:HGNC:16920]	298.2	371.2	**1.23**	0.001049
ENOX2	ecto-NOX disulfide-thiol exchanger 2 [Source:HGNC Symbol;Acc:HGNC:2259]	280.1	346.1	**1.23**	0.001256
NBPF8	NBPF member 8 [Source:HGNC Symbol;Acc:HGNC:31990]	380.4	473.9	**1.23**	0.002624
SERTAD3	SERTA domain containing 3 [Source:HGNC Symbol;Acc:HGNC:17931]	152.1	186.5	**1.23**	0.005946
KCNRG	potassium channel regulator [Source:HGNC Symbol;Acc:HGNC:18893]	155.7	192.9	**1.23**	0.006176
PLPP6	phospholipid phosphatase 6 [Source:HGNC Symbol;Acc:HGNC:23682]	188.4	232.2	**1.23**	0.006254
SMAD1	SMAD family member 1 [Source:HGNC Symbol;Acc:HGNC:6767]	198.2	244.3	**1.23**	0.007624
TPCN2	two pore segment channel 2 [Source:HGNC Symbol;Acc:HGNC:20820]	170.1	210.3	**1.23**	0.01111
MED30	mediator complex subunit 30 [Source:HGNC Symbol;Acc:HGNC:23032]	132.4	163.6	**1.23**	0.0113
UPP1	uridine phosphorylase 1 [Source:HGNC Symbol;Acc:HGNC:12576]	418.2	500.3	**1.23**	0.0194
ENDOG	endonuclease G [Source:HGNC Symbol;Acc:HGNC:3346]	103.2	127.0	**1.23**	0.02102
CEP19	centrosomal protein 19 [Source:HGNC Symbol;Acc:HGNC:28209]	135.4	166.8	**1.23**	0.02273
DLGAP4	DLG associated protein 4 [Source:HGNC Symbol;Acc:HGNC:24476]	159.1	198.1	**1.23**	0.02602
LRFN4	leucine rich repeat and fibronectin type III domain containing 4 [Source:HGNC Symbol;Acc:HGNC:28456	136.9	168.5	**1.23**	0.02645
PCSK1	proprotein convertase subtilisin/kexin type 1 [Source:HGNC Symbol;Acc:HGNC:8743]	171.1	209.8	**1.23**	0.02833
FBXL3	F-box and leucine rich repeat protein 3 [Source:HGNC Symbol;Acc:HGNC:13599]	3884.7	4765.5	**1.22**	4.21E-13
LMAN2	lectin, mannose binding 2 [Source:HGNC Symbol;Acc:HGNC:16986]	3834.8	4689.5	**1.22**	1.15E-12
MFN1	mitofusin 1 [Source:HGNC Symbol;Acc:HGNC:18262]	2272.1	2782.2	**1.22**	1.63E-12
SH3GLB1	SH3 domain containing GRB2 like, endophilin B1 [Source:HGNC Symbol;Acc:HGNC:10833]	3783.7	4637.9	**1.22**	2.44E-12
EIF3F	eukaryotic translation initiation factor 3 subunit F [Source:HGNC Symbol;Acc:HGNC:3275]	1121.0	1375.3	**1.22**	3.97E-12
TRAM1	translocation associated membrane protein 1 [Source:HGNC Symbol;Acc:HGNC:20568]	7290.9	8890.1	**1.22**	5.26E-12
PPP1R2	protein phosphatase 1 regulatory inhibitor subunit 2 [Source:HGNC Symbol;Acc:HGNC:9288]	1271.9	1555.2	**1.22**	8.86E-11
CHPT1	choline phosphotransferase 1 [Source:HGNC Symbol;Acc:HGNC:17852]	1619.5	1974.7	**1.22**	2.17E-10
CD99	CD99 molecule (Xg blood group) [Source:HGNC Symbol;Acc:HGNC:7082]	1866.3	2297.5	**1.22**	2.34E-10
ALS2	ALS2, alsin Rho guanine nucleotide exchange factor [Source:HGNC Symbol;Acc:HGNC:443]	800.1	982.1	**1.22**	3.72E-09
PITPNA	phosphatidylinositol transfer protein alpha [Source:HGNC Symbol;Acc:HGNC:9001]	1173.0	1438.2	**1.22**	3.76E-09
NECTIN3	nectin cell adhesion molecule 3 [Source:HGNC Symbol;Acc:HGNC:17664]	1103.1	1355.2	**1.22**	3.93E-09
PMM2	phosphomannomutase 2 [Source:HGNC Symbol;Acc:HGNC:9115]	945.9	1160.5	**1.22**	4.14E-09
DNAJC1	DnaJ heat shock protein family (Hsp40) member C1 [Source:HGNC Symbol;Acc:HGNC:20090]	2172.2	2666.5	**1.22**	8.95E-09
CELF1	CUGBP Elav-like family member 1 [Source:HGNC Symbol;Acc:HGNC:2549]	1717.3	2114.8	**1.22**	1.90E-08
RFFL	ring finger and FYVE like domain containing E3 ubiquitin protein ligase [Source:HGNC Symbol;Acc:HGNC	817.7	1005.3	**1.22**	2.28E-08
WWP1	WW domain containing E3 ubiquitin protein ligase 1 [Source:HGNC Symbol;Acc:HGNC:17004]	1170.7	1429.5	**1.22**	2.62E-08
MAP3K2	mitogen-activated protein kinase kinase kinase 2 [Source:HGNC Symbol;Acc:HGNC:6854]	4282.3	5214.5	**1.22**	2.70E-08
PRR14L	proline rich 14 like [Source:HGNC Symbol;Acc:HGNC:28738]	1214.0	1485.9	**1.22**	2.91E-08
MAP4K3	mitogen-activated protein kinase kinase kinase kinase 3 [Source:HGNC Symbol;Acc:HGNC:6865]	753.3	921.8	**1.22**	6.94E-08
KYAT3	kynurenine aminotransferase 3 [Source:HGNC Symbol;Acc:HGNC:33238]	902.5	1103.7	**1.22**	1.08E-07
NEK4	NIMA related kinase 4 [Source:HGNC Symbol;Acc:HGNC:11399]	844.8	1032.8	**1.22**	1.12E-07
UEVLD	UEV and lactate/malate dehyrogenase domains [Source:HGNC Symbol;Acc:HGNC:30866]	1186.2	1451.7	**1.22**	1.18E-07
WBP4	WW domain binding protein 4 [Source:HGNC Symbol;Acc:HGNC:12739]	1196.7	1466.0	**1.22**	1.65E-07
C5orf51	chromosome 5 open reading frame 51 [Source:HGNC Symbol;Acc:HGNC:27750]	1333.5	1635.5	**1.22**	1.69E-07
DENND6A	DENN domain containing 6A [Source:HGNC Symbol;Acc:HGNC:26635]	535.9	654.1	**1.22**	3.24E-07
SMARCC2	SWI/SNF related, matrix associated, actin dependent regulator of chromatin subfamily c member 2 [Sou	1427.9	1750.9	**1.22**	4.82E-07
IRAK4	interleukin 1 receptor associated kinase 4 [Source:HGNC Symbol;Acc:HGNC:17967]	687.8	844.2	**1.22**	5.42E-07
MCCC2	methylcrotonoyl-CoA carboxylase 2 [Source:HGNC Symbol;Acc:HGNC:6937]	741.9	905.8	**1.22**	6.98E-07
TARSL2	threonyl-tRNA synthetase like 2 [Source:HGNC Symbol;Acc:HGNC:24728]	536.9	659.7	**1.22**	7.55E-07
IFT74	intraflagellar transport 74 [Source:HGNC Symbol;Acc:HGNC:21424]	874.9	1073.3	**1.22**	1.27E-06
STK38	serine/threonine kinase 38 [Source:HGNC Symbol;Acc:HGNC:17847]	854.3	1050.0	**1.22**	1.30E-06
IFNGR1	interferon gamma receptor 1 [Source:HGNC Symbol;Acc:HGNC:5439]	1378.9	1685.5	**1.22**	2.42E-06
SIRT1	sirtuin 1 [Source:HGNC Symbol;Acc:HGNC:14929]	701.1	856.4	**1.22**	4.79E-06
RAI14	retinoic acid induced 14 [Source:HGNC Symbol;Acc:HGNC:14873]	858.1	1048.6	**1.22**	5.22E-06
PPP6C	protein phosphatase 6 catalytic subunit [Source:HGNC Symbol;Acc:HGNC:9323]	1095.0	1339.6	**1.22**	7.35E-06
MTRF1L	mitochondrial translational release factor 1 like [Source:HGNC Symbol;Acc:HGNC:21051]	556.3	679.9	**1.22**	7.70E-06
ERICH1	glutamate rich 1 [Source:HGNC Symbol;Acc:HGNC:27234]	834.5	1025.6	**1.22**	1.30E-05
SMIM12	small integral membrane protein 12 [Source:HGNC Symbol;Acc:HGNC:25154]	563.7	685.0	**1.22**	3.34E-05
WNK1	WNK lysine deficient protein kinase 1 [Source:HGNC Symbol;Acc:HGNC:14540]	2832.9	3460.4	**1.22**	4.56E-05
WDFY3	WD repeat and FYVE domain containing 3 [Source:HGNC Symbol;Acc:HGNC:20751]	1820.3	2221.3	**1.22**	8.76E-05

IQCE	IQ motif containing E [Source:HGNC Symbol;Acc:HGNC:29171]	437.9	535.3	**1.22**	8.96E-05
GAK	cyclin G associated kinase [Source:HGNC Symbol;Acc:HGNC:4113]	535.2	657.1	**1.22**	0.0001065
PCCA	propionyl-CoA carboxylase subunit alpha [Source:HGNC Symbol;Acc:HGNC:8653]	394.5	482.2	**1.22**	0.000129
GFOD2	glucose-fructose oxidoreductase domain containing 2 [Source:HGNC Symbol;Acc:HGNC:28159]	322.6	394.0	**1.22**	0.0001317
VPS25	vacuolar protein sorting 25 homolog [Source:HGNC Symbol;Acc:HGNC:28122]	392.2	478.2	**1.22**	0.0004579
BEST1	bestrophin 1 [Source:HGNC Symbol;Acc:HGNC:12703]	7170.3	8875.3	**1.22**	0.0007749
EVC2	EvC ciliary complex subunit 2 [Source:HGNC Symbol;Acc:HGNC:19747]	406.1	499.8	**1.22**	0.0008187
MTMR1	myotubularin related protein 1 [Source:HGNC Symbol;Acc:HGNC:7449]	317.0	389.5	**1.22**	0.0009391
TRIM26	tripartite motif containing 26 [Source:HGNC Symbol;Acc:HGNC:12962]	455.9	556.5	**1.22**	0.001006
AP2A1	adaptor related protein complex 2 subunit alpha 1 [Source:HGNC Symbol;Acc:HGNC:561]	384.0	468.9	**1.22**	0.001227
ZNF442	zinc finger protein 442 [Source:HGNC Symbol;Acc:HGNC:20877]	244.6	298.0	**1.22**	0.001455
FBXW5	F-box and WD repeat domain containing 5 [Source:HGNC Symbol;Acc:HGNC:13613]	379.2	461.4	**1.22**	0.001678
CSNK1E	casein kinase 1 epsilon [Source:HGNC Symbol;Acc:HGNC:2453]	352.5	432.8	**1.22**	0.00187
BDH2	3-hydroxybutyrate dehydrogenase 2 [Source:HGNC Symbol;Acc:HGNC:32389]	427.8	521.2	**1.22**	0.002273
TMEM216	transmembrane protein 216 [Source:HGNC Symbol;Acc:HGNC:25018]	220.7	270.1	**1.22**	0.003059
TPP1	tripeptidyl peptidase 1 [Source:HGNC Symbol;Acc:HGNC:2073]	217.8	269.7	**1.22**	0.003357
HSD17B7	hydroxysteroid 17-beta dehydrogenase 7 [Source:HGNC Symbol;Acc:HGNC:5215]	299.1	366.3	**1.22**	0.004362
CHAD	chondroadherin [Source:HGNC Symbol;Acc:HGNC:1909]	275.5	337.4	**1.22**	0.005322
CDC34	cell division cycle 34 [Source:HGNC Symbol;Acc:HGNC:1734]	268.1	328.4	**1.22**	0.006736
CRNDE	colorectal neoplasia differentially expressed [Source:HGNC Symbol;Acc:HGNC:37078]	326.6	396.6	**1.22**	0.007999
CCDC130	coiled-coil domain containing 130 [Source:HGNC Symbol;Acc:HGNC:28118]	159.3	195.2	**1.22**	0.008266
RUSC2	RUN and SH3 domain containing 2 [Source:HGNC Symbol;Acc:HGNC:23625]	453.8	559.3	**1.22**	0.008318
CCNB1IP1	cyclin B1 interacting protein 1 [Source:HGNC Symbol;Acc:HGNC:19437]	339.7	428.2	**1.22**	0.009719
CRYBG1	crystallin beta-gamma domain containing 1 [Source:HGNC Symbol;Acc:HGNC:356]	259.2	304.2	**1.22**	0.009999
HDAC11	histone deacetylase 11 [Source:HGNC Symbol;Acc:HGNC:19086]	135.5	166.6	**1.22**	0.01552
CCDC97	coiled-coil domain containing 97 [Source:HGNC Symbol;Acc:HGNC:28289]	179.1	219.1	**1.22**	0.01569
UBIAD1	UbiA prenyltransferase domain containing 1 [Source:HGNC Symbol;Acc:HGNC:30791]	151.0	185.2	**1.22**	0.01621
FAM13A-AS1	FAM13A antisense RNA 1 [Source:HGNC Symbol;Acc:HGNC:19370]	110.2	135.9	**1.22**	0.04332
GPR89A	G protein-coupled receptor 89A [Source:HGNC Symbol;Acc:HGNC:31984]	107.8	132.8	**1.22**	0.04887
UBE2D3	ubiquitin conjugating enzyme E2 D3 [Source:HGNC Symbol;Acc:HGNC:12476]	7358.9	8954.2	**1.21**	5.97E-23
LEPROT	leptin receptor overlapping transcript [Source:HGNC Symbol;Acc:HGNC:29477]	3141.3	3823.2	**1.21**	9.60E-18
WASHC4	WASH complex subunit 4 [Source:HGNC Symbol;Acc:HGNC:29174]	4705.5	5733.9	**1.21**	3.78E-16
SPAST	spastin [Source:HGNC Symbol;Acc:HGNC:11233]	1020.5	1244.4	**1.21**	3.18E-11
UHMK1	U2AF homology motif kinase 1 [Source:HGNC Symbol;Acc:HGNC:19683]	5131.1	6204.1	**1.21**	5.24E-11
RBM12	RNA binding motif protein 12 [Source:HGNC Symbol;Acc:HGNC:9898]	2399.4	2907.8	**1.21**	5.74E-11
MANBA	mannosidase beta [Source:HGNC Symbol;Acc:HGNC:6831]	2729.9	3323.7	**1.21**	6.36E-11
ASNSD1	asparagine synthetase domain containing 1 [Source:HGNC Symbol;Acc:HGNC:24910]	1568.0	1900.0	**1.21**	6.97E-11
MPHOSPH8	M-phase phosphoprotein 8 [Source:HGNC Symbol;Acc:HGNC:29810]	1660.9	2014.1	**1.21**	8.93E-11
UBE2Q2	ubiquitin conjugating enzyme E2 Q2 [Source:HGNC Symbol;Acc:HGNC:19248]	2881.5	3491.6	**1.21**	1.43E-10
EIF3D	eukaryotic translation initiation factor 3 subunit D [Source:HGNC Symbol;Acc:HGNC:3278]	2681.6	3264.7	**1.21**	5.09E-10
BACH1	BTB domain and CNC homolog 1 [Source:HGNC Symbol;Acc:HGNC:935]	1912.7	2330.0	**1.21**	2.22E-09
PTBP3	polypyrimidine tract binding protein 3 [Source:HGNC Symbol;Acc:HGNC:10253]	2638.2	3205.9	**1.21**	4.66E-09
LNPEP	leucyl and cystinyl aminopeptidase [Source:HGNC Symbol;Acc:HGNC:6656]	6969.5	8495.0	**1.21**	1.13E-08
CTBP2	C-terminal binding protein 2 [Source:HGNC Symbol;Acc:HGNC:2495]	1482.4	1795.8	**1.21**	3.17E-08
HERC4	HECT and RLD domain containing E3 ubiquitin protein ligase 4 [Source:HGNC Symbol;Acc:HGNC:24521]	2348.7	2845.1	**1.21**	4.52E-08
SEC22C	SEC22 homolog C, vesicle trafficking protein [Source:HGNC Symbol;Acc:HGNC:16828]	1150.3	1398.0	**1.21**	5.98E-08
ZNF800	zinc finger protein 800 [Source:HGNC Symbol;Acc:HGNC:27267]	791.7	962.3	**1.21**	6.05E-08
NFKB1	nuclear factor kappa B subunit 1 [Source:HGNC Symbol;Acc:HGNC:7794]	1161.2	1407.9	**1.21**	7.18E-08
ACAT1	acetyl-CoA acetyltransferase 1 [Source:HGNC Symbol;Acc:HGNC:93]	1588.7	1939.5	**1.21**	7.85E-08
RBM3	RNA binding motif protein 3 [Source:HGNC Symbol;Acc:HGNC:9900]	6601.2	7950.4	**1.21**	1.05E-07
CEP162	centrosomal protein 162 [Source:HGNC Symbol;Acc:HGNC:21107]	779.6	945.1	**1.21**	1.77E-07
MBD1	methyl-CpG binding domain protein 1 [Source:HGNC Symbol;Acc:HGNC:6916]	593.2	720.3	**1.21**	2.19E-07
SLAIN2	SLAIN motif family member 2 [Source:HGNC Symbol;Acc:HGNC:29282]	1913.3	2335.1	**1.21**	2.53E-07
MEAF6	MYST/Esa1 associated factor 6 [Source:HGNC Symbol;Acc:HGNC:25674]	1017.2	1243.8	**1.21**	2.73E-07
KLHL2	kelch like family member 2 [Source:HGNC Symbol;Acc:HGNC:6353]	1281.7	1555.5	**1.21**	3.00E-07
MCMBP	minichromosome maintenance complex binding protein [Source:HGNC Symbol;Acc:HGNC:25782]	1590.2	1934.7	**1.21**	3.87E-07
GNPAT	glyceronephosphate O-acyltransferase [Source:HGNC Symbol;Acc:HGNC:4416]	1241.2	1512.2	**1.21**	8.40E-07
CCNI	cyclin I [Source:HGNC Symbol;Acc:HGNC:1595]	2095.9	2544.9	**1.21**	9.51E-07
MT-CO1	mitochondrially encoded cytochrome c oxidase I [Source:HGNC Symbol;Acc:HGNC:7419]	8002.1	9717.7	**1.21**	1.05E-06
TRPC4AP	transient receptor potential cation channel subfamily C member 4 associated protein [Source:HGNC Syr	1532.4	1873.9	**1.21**	1.16E-06
USP10	ubiquitin specific peptidase 10 [Source:HGNC Symbol;Acc:HGNC:12608]	613.6	747.2	**1.21**	1.44E-06
EMG1	EMG1, N1-specific pseudouridine methyltransferase [Source:NCBI gene;Acc:10436]	924.3	1120.5	**1.21**	1.73E-06
PCMTD2	protein-L-isoaspartate (D-aspartate) O-methyltransferase domain containing 2 [Source:HGNC Symbol;A	534.3	645.5	**1.21**	8.97E-06
NBEAL1	neurobeachin like 1 [Source:HGNC Symbol;Acc:HGNC:20681]	1579.7	1915.7	**1.21**	8.97E-06
HERPUD2	HERPUD family member 2 [Source:HGNC Symbol;Acc:HGNC:21915]	623.9	756.9	**1.21**	1.02E-05
VPS13D	vacuolar protein sorting 13 homolog D [Source:HGNC Symbol;Acc:HGNC:23595]	2572.8	3119.1	**1.21**	1.29E-05
ZNF791	zinc finger protein 791 [Source:HGNC Symbol;Acc:HGNC:26895]	839.6	1022.8	**1.21**	1.49E-05
FDX1	ferredoxin 1 [Source:HGNC Symbol;Acc:HGNC:3638]	408.1	496.2	**1.21**	1.57E-05
SMG6	SMG6, nonsense mediated mRNA decay factor [Source:HGNC Symbol;Acc:HGNC:17809]	624.9	757.6	**1.21**	2.18E-05
MT-CO3	mitochondrially encoded cytochrome c oxidase III [Source:HGNC Symbol;Acc:HGNC:7422]	2647.9	3263.9	**1.21**	3.52E-05
CPT1A	carnitine palmitoyltransferase 1A [Source:HGNC Symbol;Acc:HGNC:2328]	867.3	1053.9	**1.21**	5.93E-05
FASTKD5	FAST kinase domains 5 [Source:HGNC Symbol;Acc:HGNC:25790]	385.4	470.5	**1.21**	0.0001009

KCTD12	potassium channel tetramerization domain containing 12 [Source:HGNC Symbol;Acc:HGNC:14678]	1059.7	1303.8	**1.21**	0.0001251
FPGT	fucose-1-phosphate guanylyltransferase [Source:HGNC Symbol;Acc:HGNC:3825]	578.9	700.5	**1.21**	0.0001328
ETFRF1	electron transfer flavoprotein regulatory factor 1 [Source:HGNC Symbol;Acc:HGNC:27052]	341.6	413.5	**1.21**	0.0002431
POR	cytochrome p450 oxidoreductase [Source:HGNC Symbol;Acc:HGNC:9208]	380.9	461.9	**1.21**	0.0002999
ADCY9	adenylate cyclase 9 [Source:HGNC Symbol;Acc:HGNC:240]	376.4	456.1	**1.21**	0.0004867
SEC14L1	SEC14 like lipid binding 1 [Source:HGNC Symbol;Acc:HGNC:10698]	870.3	1062.8	**1.21**	0.0006035
BRD4	bromodomain containing 4 [Source:HGNC Symbol;Acc:HGNC:13575]	336.4	410.0	**1.21**	0.0007823
IFT22	intraflagellar transport 22 [Source:HGNC Symbol;Acc:HGNC:21895]	325.2	397.3	**1.21**	0.0009453
MAP3K13	mitogen-activated protein kinase kinase kinase 13 [Source:HGNC Symbol;Acc:HGNC:6852]	347.8	423.5	**1.21**	0.001073
SNX24	sorting nexin 24 [Source:HGNC Symbol;Acc:HGNC:21533]	629.3	760.4	**1.21**	0.001119
CSNK1G2	casein kinase 1 gamma 2 [Source:HGNC Symbol;Acc:HGNC:2455]	374.5	454.5	**1.21**	0.001631
FASN	fatty acid synthase [Source:HGNC Symbol;Acc:HGNC:3594]	494.3	602.5	**1.21**	0.00167
PEX11B	peroxisomal biogenesis factor 11 beta [Source:HGNC Symbol;Acc:HGNC:8853]	289.1	351.4	**1.21**	0.002665
RSC1A1	regulator of solute carriers 1 [Source:HGNC Symbol;Acc:HGNC:10458]	255.1	308.6	**1.21**	0.002731
NFS1	NFS1, cysteine desulfurase [Source:HGNC Symbol;Acc:HGNC:15910]	232.6	282.1	**1.21**	0.002847
ZNF595	zinc finger protein 595 [Source:HGNC Symbol;Acc:HGNC:27196]	267.1	324.4	**1.21**	0.002922
STX3	syntaxin 3 [Source:HGNC Symbol;Acc:HGNC:11438]	224.9	275.4	**1.21**	0.004158
TMEM254	transmembrane protein 254 [Source:HGNC Symbol;Acc:HGNC:25804]	177.9	216.4	**1.21**	0.004765
CHIC2	cysteine rich hydrophobic domain 2 [Source:HGNC Symbol;Acc:HGNC:1935]	327.4	399.2	**1.21**	0.006528
NT5DC3	5'-nucleotidase domain containing 3 [Source:HGNC Symbol;Acc:HGNC:30826]	750.7	940.3	**1.21**	0.006788
MTHFR	methylenetetrahydrofolate reductase [Source:HGNC Symbol;Acc:HGNC:7436]	264.7	321.3	**1.21**	0.007885
KIAA1614	KIAA1614 [Source:HGNC Symbol;Acc:HGNC:29327]	101.7	125.1	**1.21**	0.02718
GK	glycerol kinase [Source:HGNC Symbol;Acc:HGNC:4289]	128.3	156.5	**1.21**	0.03019
NUDT11	nudix hydrolase 11 [Source:HGNC Symbol;Acc:HGNC:18011]	96.0	116.4	**1.21**	0.0383
LRRC37A2	leucine rich repeat containing 37 member A2 [Source:HGNC Symbol;Acc:HGNC:32404]	114.4	141.4	**1.21**	0.04627
TMBIM6	transmembrane BAX inhibitor motif containing 6 [Source:HGNC Symbol;Acc:HGNC:11723]	8100.4	9795.5	**1.21**	6.47E-17
EIF3A	eukaryotic translation initiation factor 3 subunit A [Source:HGNC Symbol;Acc:HGNC:3271]	9104.3	11007.8	**1.21**	1.92E-16
KIF3B	kinesin family member 3B [Source:HGNC Symbol;Acc:HGNC:6320]	2017.7	2428.2	**1.21**	5.87E-14
PDS5A	PDS5 cohesin associated factor A [Source:HGNC Symbol;Acc:HGNC:29088]	4890.2	5908.9	**1.21**	1.26E-13
BECN1	beclin 1 [Source:HGNC Symbol;Acc:HGNC:1034]	2458.3	2958.7	**1.21**	3.81E-12
ITCH	itchy E3 ubiquitin protein ligase [Source:HGNC Symbol;Acc:HGNC:13890]	4005.3	4830.2	**1.21**	1.07E-11
DCTN5	dynactin subunit 5 [Source:HGNC Symbol;Acc:HGNC:24594]	1645.7	1987.9	**1.21**	5.65E-11
RYK	receptor-like tyrosine kinase [Source:HGNC Symbol;Acc:HGNC:10481]	2879.9	3480.8	**1.21**	1.12E-10
EIF4B	eukaryotic translation initiation factor 4B [Source:HGNC Symbol;Acc:HGNC:3285]	16562.3	20131.1	**1.21**	2.81E-10
SH3BGRL	SH3 domain binding glutamate rich protein like [Source:HGNC Symbol;Acc:HGNC:10823]	1276.5	1540.4	**1.21**	3.31E-10
MSANTD4	Myb/SANT DNA binding domain containing 4 with coiled-coils [Source:HGNC Symbol;Acc:HGNC:29383]	1234.1	1492.9	**1.21**	4.25E-10
RALGAPB	Ral GTPase activating protein non-catalytic beta subunit [Source:HGNC Symbol;Acc:HGNC:29221]	1443.7	1742.6	**1.21**	5.99E-10
ZYG11B	zyg-11 family member B, cell cycle regulator [Source:HGNC Symbol;Acc:HGNC:25820]	1855.5	2241.4	**1.21**	2.95E-09
EXOC8	exocyst complex component 8 [Source:HGNC Symbol;Acc:HGNC:24659]	1052.9	1271.8	**1.21**	2.24E-08
KDM3B	lysine demethylase 3B [Source:HGNC Symbol;Acc:HGNC:1337]	1228.5	1482.1	**1.21**	2.48E-08
TFIP11	tuftelin interacting protein 11 [Source:HGNC Symbol;Acc:HGNC:17165]	1180.3	1425.0	**1.21**	3.17E-08
ANKRD42	ankyrin repeat domain 42 [Source:HGNC Symbol;Acc:HGNC:26752]	995.8	1199.6	**1.21**	3.78E-08
KPNA4	karyopherin subunit alpha 4 [Source:HGNC Symbol;Acc:HGNC:6397]	2568.4	3121.3	**1.21**	5.28E-08
OTUD4	OTU deubiquitinase 4 [Source:HGNC Symbol;Acc:HGNC:24949]	1370.9	1659.3	**1.21**	8.12E-08
PDCD10	programmed cell death 10 [Source:HGNC Symbol;Acc:HGNC:8761]	1534.9	1851.4	**1.21**	8.30E-08
FAM120A	family with sequence similarity 120A [Source:HGNC Symbol;Acc:HGNC:13247]	2183.0	2638.7	**1.21**	1.86E-07
ENTPD4	ectonucleoside triphosphate diphosphohydrolase 4 [Source:HGNC Symbol;Acc:HGNC:14573]	1028.5	1240.3	**1.21**	2.15E-07
NPC2	NPC intracellular cholesterol transporter 2 [Source:HGNC Symbol;Acc:HGNC:14537]	1639.6	1984.8	**1.21**	5.59E-07
LSG1	large 60S subunit nuclear export GTPase 1 [Source:HGNC Symbol;Acc:HGNC:25652]	1247.7	1500.7	**1.21**	8.92E-07
NSMAF	neutral sphingomyelinase activation associated factor [Source:HGNC Symbol;Acc:HGNC:8017]	818.2	987.5	**1.21**	1.46E-06
WASHC2A	WASH complex subunit 2A [Source:HGNC Symbol;Acc:HGNC:23416]	972.8	1175.9	**1.21**	1.47E-06
PDXK	pyridoxal kinase [Source:HGNC Symbol;Acc:HGNC:8819]	825.9	998.6	**1.21**	1.72E-06
SNIP1	Smad nuclear interacting protein 1 [Source:HGNC Symbol;Acc:HGNC:30587]	663.3	802.5	**1.21**	2.93E-06
PRKCI	protein kinase C iota [Source:HGNC Symbol;Acc:HGNC:9404]	1053.9	1269.9	**1.21**	5.61E-06
ZNF525	zinc finger protein 525 [Source:HGNC Symbol;Acc:HGNC:29423]	544.3	659.4	**1.21**	7.76E-06
TTC14	tetratricopeptide repeat domain 14 [Source:HGNC Symbol;Acc:HGNC:24697]	1189.6	1434.5	**1.21**	8.40E-06
NAA20	N(alpha)-acetyltransferase 20, NatB catalytic subunit [Source:HGNC Symbol;Acc:HGNC:15908]	1129.4	1355.1	**1.21**	8.46E-06
CDK7	cyclin dependent kinase 7 [Source:HGNC Symbol;Acc:HGNC:1778]	566.1	682.3	**1.21**	9.11E-06
CYTH3	cytohesin 3 [Source:HGNC Symbol;Acc:HGNC:9504]	792.2	960.5	**1.21**	1.17E-05
NOP10	NOP10 ribonucleoprotein [Source:HGNC Symbol;Acc:HGNC:14378]	649.5	783.7	**1.21**	1.23E-05
LEPR	leptin receptor [Source:HGNC Symbol;Acc:HGNC:6554]	613.3	744.5	**1.21**	1.87E-05
LAMTOR5	late endosomal/lysosomal adaptor, MAPK and MTOR activator 5 [Source:HGNC Symbol;Acc:HGNC:1795	776.1	939.5	**1.21**	2.57E-05
SPTSSA	serine palmitoyltransferase small subunit A [Source:HGNC Symbol;Acc:HGNC:20361]	534.9	643.9	**1.21**	2.87E-05
ISCA1	iron-sulfur cluster assembly 1 [Source:HGNC Symbol;Acc:HGNC:28660]	831.0	1002.5	**1.21**	3.16E-05
NELFB	negative elongation factor complex member B [Source:HGNC Symbol;Acc:HGNC:24324]	501.8	606.7	**1.21**	3.21E-05
NCOA1	nuclear receptor coactivator 1 [Source:HGNC Symbol;Acc:HGNC:7668]	1054.3	1278.9	**1.21**	5.03E-05
C6orf120	chromosome 6 open reading frame 120 [Source:HGNC Symbol;Acc:HGNC:21247]	695.8	843.1	**1.21**	6.89E-05
ETNK1	ethanolamine kinase 1 [Source:HGNC Symbol;Acc:HGNC:24649]	1345.3	1613.8	**1.21**	8.26E-05
HECTD3	HECT domain E3 ubiquitin protein ligase 3 [Source:HGNC Symbol;Acc:HGNC:26117]	470.5	568.8	**1.21**	0.000109
RFTN2	raftlin family member 2 [Source:HGNC Symbol;Acc:HGNC:26402]	1174.8	1443.4	**1.21**	0.0001156
MT-ND3	mitochondrially encoded NADH:ubiquinone oxidoreductase core subunit 3 [Source:HGNC Symbol;Acc:H	1198.7	1447.5	**1.21**	0.0002031
NUP85	nucleoporin 85 [Source:HGNC Symbol;Acc:HGNC:8734]	381.3	459.2	**1.21**	0.0002262

ST6GALNAC2	ST6 N-acetylgalactosaminide alpha-2,6-sialyltransferase 2 [Source:HGNC Symbol;Acc:HGNC:10867]	592.6	712.8	**1.21**	0.0002588
ZNF720	zinc finger protein 720 [Source:HGNC Symbol;Acc:HGNC:26987]	415.5	504.5	**1.21**	0.0003985
SLC25A4	solute carrier family 25 member 4 [Source:HGNC Symbol;Acc:HGNC:10990]	335.2	407.1	**1.21**	0.0004485
LRRC49	leucine rich repeat containing 49 [Source:HGNC Symbol;Acc:HGNC:25965]	268.4	323.7	**1.21**	0.000584
AAMDC	adipogenesis associated Mth938 domain containing [Source:HGNC Symbol;Acc:HGNC:30205]	312.4	377.3	**1.21**	0.0006383
METTL23	methyltransferase like 23 [Source:HGNC Symbol;Acc:HGNC:26988]	249.7	302.8	**1.21**	0.0006841
TOMM34	translocase of outer mitochondrial membrane 34 [Source:HGNC Symbol;Acc:HGNC:15746]	522.9	634.4	**1.21**	0.0007013
GUSB	glucuronidase beta [Source:HGNC Symbol;Acc:HGNC:4696]	291.5	352.2	**1.21**	0.0007248
HERC2P2	hect domain and RLD 2 pseudogene 2 [Source:HGNC Symbol;Acc:HGNC:4870]	360.7	434.7	**1.21**	0.0007267
PRDM2	PR/SET domain 2 [Source:HGNC Symbol;Acc:HGNC:9347]	765.9	931.0	**1.21**	0.0009316
OPHN1	oligophrenin 1 [Source:HGNC Symbol;Acc:HGNC:8148]	606.6	732.7	**1.21**	0.001232
CACTIN	cactin, spliceosome C complex subunit [Source:HGNC Symbol;Acc:HGNC:29938]	244.9	295.5	**1.21**	0.001308
PKNOX1	PBX/knotted 1 homeobox 1 [Source:HGNC Symbol;Acc:HGNC:9022]	374.9	451.7	**1.21**	0.002121
PRKAB1	protein kinase AMP-activated non-catalytic subunit beta 1 [Source:HGNC Symbol;Acc:HGNC:9378]	233.9	283.0	**1.21**	0.002522
UBE2O	ubiquitin conjugating enzyme E2 O [Source:HGNC Symbol;Acc:HGNC:29554]	482.3	586.8	**1.21**	0.002984
ZNF782	zinc finger protein 782 [Source:HGNC Symbol;Acc:HGNC:33110]	262.0	316.3	**1.21**	0.002994
ECE1	endothelin converting enzyme 1 [Source:HGNC Symbol;Acc:HGNC:3146]	450.5	545.4	**1.21**	0.003046
NCOA6	nuclear receptor coactivator 6 [Source:HGNC Symbol;Acc:HGNC:15936]	238.0	287.3	**1.21**	0.003433
CRAT	carnitine O-acetyltransferase [Source:HGNC Symbol;Acc:HGNC:2342]	367.5	452.7	**1.21**	0.005346
C1orf115	chromosome 1 open reading frame 115 [Source:HGNC Symbol;Acc:HGNC:25873]	276.0	334.4	**1.21**	0.007318
MTF1	metal regulatory transcription factor 1 [Source:HGNC Symbol;Acc:HGNC:7428]	286.9	346.0	**1.21**	0.009095
KIAA0556	KIAA0556 [Source:HGNC Symbol;Acc:HGNC:29068]	301.3	361.8	**1.21**	0.009611
SF3B5	splicing factor 3b subunit 5 [Source:HGNC Symbol;Acc:HGNC:21083]	279.4	335.2	**1.21**	0.01062
CTDP1	CTD phosphatase subunit 1 [Source:HGNC Symbol;Acc:HGNC:2498]	210.5	256.2	**1.21**	0.01085
ZNF225	zinc finger protein 225 [Source:HGNC Symbol;Acc:HGNC:13018]	164.0	196.9	**1.21**	0.01106
TJAP1	tight junction associated protein 1 [Source:HGNC Symbol;Acc:HGNC:17949]	181.6	218.0	**1.21**	0.01219
PEX11A	peroxisomal biogenesis factor 11 alpha [Source:HGNC Symbol;Acc:HGNC:8852]	214.9	262.2	**1.21**	0.01586
ISG20	interferon stimulated exonuclease gene 20 [Source:HGNC Symbol;Acc:HGNC:6130]	235.9	287.9	**1.21**	0.01807
LRRC37A17P	leucine rich repeat containing 37 member A17, pseudogene [Source:HGNC Symbol;Acc:HGNC:48365]	174.1	210.6	**1.21**	0.01951
ZNF214	zinc finger protein 214 [Source:HGNC Symbol;Acc:HGNC:13006]	175.7	214.0	**1.21**	0.02086
NUTM2A-AS1	NUTM2A antisense RNA 1 [Source:HGNC Symbol;Acc:HGNC:45161]	171.3	205.9	**1.21**	0.02854
SND1	staphylococcal nuclease and tudor domain containing 1 [Source:HGNC Symbol;Acc:HGNC:30646]	5481.6	6578.3	**1.20**	4.44E-15
ARFGAP3	ADP ribosylation factor GTPase activating protein 3 [Source:HGNC Symbol;Acc:HGNC:661]	5240.7	6272.9	**1.20**	2.07E-12
CDC5L	cell division cycle 5 like [Source:HGNC Symbol;Acc:HGNC:1743]	2523.0	3026.8	**1.20**	1.36E-11
RPL10	ribosomal protein L10 [Source:HGNC Symbol;Acc:HGNC:10298]	3760.0	4495.8	**1.20**	1.45E-11
USP33	ubiquitin specific peptidase 33 [Source:HGNC Symbol;Acc:HGNC:20059]	1829.1	2193.8	**1.20**	2.86E-10
SLMAP	sarcolemma associated protein [Source:HGNC Symbol;Acc:HGNC:16643]	1722.6	2062.7	**1.20**	4.05E-10
MPRIP	myosin phosphatase Rho interacting protein [Source:HGNC Symbol;Acc:HGNC:30321]	2681.6	3213.8	**1.20**	1.07E-09
SUMF2	sulfatase modifying factor 2 [Source:HGNC Symbol;Acc:HGNC:20415]	2145.4	2578.0	**1.20**	2.05E-09
CNTD1	cyclin N-terminal domain containing 1 [Source:HGNC Symbol;Acc:HGNC:26847]	1223.8	1471.0	**1.20**	2.26E-09
ATP6AP2	ATPase H+ transporting accessory protein 2 [Source:HGNC Symbol;Acc:HGNC:18305]	3474.3	4189.1	**1.20**	2.43E-09
CUL1	cullin 1 [Source:HGNC Symbol;Acc:HGNC:2551]	1850.3	2212.3	**1.20**	9.39E-09
CRNKL1	crooked neck pre-mRNA splicing factor 1 [Source:HGNC Symbol;Acc:HGNC:15762]	1735.7	2076.6	**1.20**	9.45E-09
HELZ	helicase with zinc finger [Source:HGNC Symbol;Acc:HGNC:16878]	1311.0	1572.1	**1.20**	4.83E-08
STAT3	signal transducer and activator of transcription 3 [Source:HGNC Symbol;Acc:HGNC:11364]	4149.5	4972.6	**1.20**	1.39E-07
FUNDC2	FUN14 domain containing 2 [Source:HGNC Symbol;Acc:HGNC:24925]	1679.4	2006.2	**1.20**	1.73E-07
UTP18	UTP18, small subunit processome component [Source:HGNC Symbol;Acc:HGNC:24274]	1114.6	1337.5	**1.20**	2.04E-07
LAMTOR3	late endosomal/lysosomal adaptor, MAPK and MTOR activator 3 [Source:HGNC Symbol;Acc:HGNC:1560	1135.4	1360.5	**1.20**	2.21E-07
WDR44	WD repeat domain 44 [Source:HGNC Symbol;Acc:HGNC:30512]	909.9	1090.7	**1.20**	7.03E-07
UBE3B	ubiquitin protein ligase E3B [Source:HGNC Symbol;Acc:HGNC:13478]	885.5	1059.3	**1.20**	1.26E-06
TSPAN31	tetraspanin 31 [Source:HGNC Symbol;Acc:HGNC:10539]	1167.2	1396.8	**1.20**	1.35E-06
KIAA0391	KIAA0391 [Source:HGNC Symbol;Acc:HGNC:19958]	1114.2	1336.0	**1.20**	1.57E-06
CTNNBL1	catenin beta like 1 [Source:HGNC Symbol;Acc:HGNC:15879]	684.8	823.2	**1.20**	1.93E-06
LYSMD3	LysM domain containing 3 [Source:HGNC Symbol;Acc:HGNC:26969]	1445.4	1726.8	**1.20**	2.28E-06
CD2BP2	CD2 cytoplasmic tail binding protein 2 [Source:HGNC Symbol;Acc:HGNC:1656]	1017.9	1215.1	**1.20**	3.50E-06
GBA	glucosylceramidase beta [Source:HGNC Symbol;Acc:HGNC:4177]	966.4	1161.5	**1.20**	4.82E-06
ZMYM6	zinc finger MYM-type containing 6 [Source:HGNC Symbol;Acc:HGNC:13050]	814.3	975.6	**1.20**	5.89E-06
ANKHD1	ankyrin repeat and KH domain containing 1 [Source:HGNC Symbol;Acc:HGNC:24714]	2243.6	2690.5	**1.20**	6.64E-06
ANKHD1-EIF4EBP3	ANKHD1-EIF4EBP3 readthrough [Source:HGNC Symbol;Acc:HGNC:33530]	2269.4	2713.5	**1.20**	7.81E-06
STAT6	signal transducer and activator of transcription 6 [Source:HGNC Symbol;Acc:HGNC:11368]	2290.9	2765.4	**1.20**	7.92E-06
CASC3	CASC3, exon junction complex subunit [Source:HGNC Symbol;Acc:HGNC:17040]	1380.2	1653.9	**1.20**	9.75E-06
EXOC3	exocyst complex component 3 [Source:HGNC Symbol;Acc:HGNC:30378]	1048.3	1255.0	**1.20**	1.10E-05
DNAJB2	DnaJ heat shock protein family (Hsp40) member B2 [Source:HGNC Symbol;Acc:HGNC:5228]	848.6	1019.7	**1.20**	1.88E-05
TYW1	tRNA-yW synthesizing protein 1 homolog [Source:HGNC Symbol;Acc:HGNC:25598]	885.1	1064.7	**1.20**	1.92E-05
DYNC2LI1	dynein cytoplasmic 2 light intermediate chain 1 [Source:HGNC Symbol;Acc:HGNC:24595]	458.3	548.5	**1.20**	2.84E-05
HDAC8	histone deacetylase 8 [Source:HGNC Symbol;Acc:HGNC:13315]	489.3	587.8	**1.20**	2.99E-05
MBOAT2	membrane bound O-acyltransferase domain containing 2 [Source:HGNC Symbol;Acc:HGNC:25193]	934.9	1121.4	**1.20**	3.64E-05
EHD4	EH domain containing 4 [Source:HGNC Symbol;Acc:HGNC:3245]	508.1	607.6	**1.20**	4.07E-05
GPATCH4	G-patch domain containing 4 [Source:HGNC Symbol;Acc:HGNC:25982]	774.2	932.3	**1.20**	4.49E-05
CEP85L	centrosomal protein 85 like [Source:HGNC Symbol;Acc:HGNC:21638]	627.2	752.7	**1.20**	4.59E-05
TAF1B	TATA-box binding protein associated factor, RNA polymerase I subunit B [Source:HGNC Symbol;Acc:HGN	556.4	667.4	**1.20**	6.13E-05
VPS13B	vacuolar protein sorting 13 homolog B [Source:HGNC Symbol;Acc:HGNC:2183]	1349.6	1621.8	**1.20**	6.41E-05

HSD17B10	hydroxysteroid 17-beta dehydrogenase 10 [Source:HGNC Symbol;Acc:HGNC:4800]	667.3	803.2	**1.20**	7.70E-05
LYST	lysosomal trafficking regulator [Source:HGNC Symbol;Acc:HGNC:1968]	761.1	906.5	**1.20**	7.85E-05
SLC38A10	solute carrier family 38 member 10 [Source:HGNC Symbol;Acc:HGNC:28237]	1465.7	1754.6	**1.20**	8.63E-05
E2F4	E2F transcription factor 4 [Source:HGNC Symbol;Acc:HGNC:3118]	419.5	505.8	**1.20**	8.99E-05
CSGALNACT2	chondroitin sulfate N-acetylgalactosaminyltransferase 2 [Source:HGNC Symbol;Acc:HGNC:24292]	3119.7	3777.2	**1.20**	9.13E-05
UBN2	ubinuclein 2 [Source:HGNC Symbol;Acc:HGNC:21931]	573.2	687.0	**1.20**	9.81E-05
VHL	von Hippel-Lindau tumor suppressor [Source:HGNC Symbol;Acc:HGNC:12687]	567.5	680.3	**1.20**	0.0001027
PTPN18	protein tyrosine phosphatase, non-receptor type 18 [Source:HGNC Symbol;Acc:HGNC:9649]	400.1	480.6	**1.20**	0.000128
CDC16	cell division cycle 16 [Source:HGNC Symbol;Acc:HGNC:1720]	717.0	860.8	**1.20**	0.0001719
ASCC2	activating signal cointegrator 1 complex subunit 2 [Source:HGNC Symbol;Acc:HGNC:24103]	458.5	549.7	**1.20**	0.0002407
MCU	mitochondrial calcium uniporter [Source:HGNC Symbol;Acc:HGNC:23526]	576.7	690.4	**1.20**	0.0003352
NAA10	N(alpha)-acetyltransferase 10, NatA catalytic subunit [Source:HGNC Symbol;Acc:HGNC:18704]	363.4	434.0	**1.20**	0.0006102
CCDC126	coiled-coil domain containing 126 [Source:HGNC Symbol;Acc:HGNC:22398]	362.8	436.3	**1.20**	0.0006184
CHTF8	chromosome transmission fidelity factor 8 [Source:HGNC Symbol;Acc:HGNC:24353]	242.0	288.3	**1.20**	0.002244
TUBGCP6	tubulin gamma complex associated protein 6 [Source:HGNC Symbol;Acc:HGNC:18127]	263.9	319.6	**1.20**	0.003081
SYNE1	spectrin repeat containing nuclear envelope protein 1 [Source:HGNC Symbol;Acc:HGNC:17089]	7370.8	8890.7	**1.20**	0.003346
GRN	granulin precursor [Source:HGNC Symbol;Acc:HGNC:4601]	777.4	929.1	**1.20**	0.004066
DRAM1	DNA damage regulated autophagy modulator 1 [Source:HGNC Symbol;Acc:HGNC:25645]	357.7	424.0	**1.20**	0.005481
MOAP1	modulator of apoptosis 1 [Source:HGNC Symbol;Acc:HGNC:16658]	365.0	445.6	**1.20**	0.007745
ZNF823	zinc finger protein 823 [Source:HGNC Symbol;Acc:HGNC:30936]	228.2	272.9	**1.20**	0.01147
KLF7	Kruppel like factor 7 [Source:HGNC Symbol;Acc:HGNC:6350]	281.0	334.4	**1.20**	0.01225
SNORA32	small nucleolar RNA, H/ACA box 32 [Source:HGNC Symbol;Acc:HGNC:32622]	207.1	250.9	**1.20**	0.01645
EMD	emerin [Source:HGNC Symbol;Acc:HGNC:3331]	259.5	310.2	**1.20**	0.02123
EFCAB7	EF-hand calcium binding domain 7 [Source:HGNC Symbol;Acc:HGNC:29379]	219.0	264.2	**1.20**	0.02147
HPS6	HPS6, biogenesis of lysosomal organelles complex 2 subunit 3 [Source:HGNC Symbol;Acc:HGNC:18817]	162.0	194.5	**1.20**	0.02148
TTC38	tetratricopeptide repeat domain 38 [Source:HGNC Symbol;Acc:HGNC:26082]	244.7	299.0	**1.20**	0.024
TYRO3	TYRO3 protein tyrosine kinase [Source:HGNC Symbol;Acc:HGNC:12446]	302.7	362.6	**1.20**	0.0243
ABHD15	abhydrolase domain containing 15 [Source:HGNC Symbol;Acc:HGNC:26971]	215.4	258.8	**1.20**	0.02456
PKIG	cAMP-dependent protein kinase inhibitor gamma [Source:HGNC Symbol;Acc:HGNC:9019]	411.2	503.1	**1.20**	0.02763
ADHFE1	alcohol dehydrogenase, iron containing 1 [Source:HGNC Symbol;Acc:HGNC:16354]	141.5	169.3	**1.20**	0.03314
TRAF7	TNF receptor associated factor 7 [Source:HGNC Symbol;Acc:HGNC:20456]	239.0	285.4	**1.20**	0.03436
HDAC7	histone deacetylase 7 [Source:HGNC Symbol;Acc:HGNC:14067]	165.9	199.6	**1.20**	0.04242
TRABD	TraB domain containing [Source:HGNC Symbol;Acc:HGNC:28805]	121.6	146.1	**1.20**	0.04253
FAM3A	family with sequence similarity 3 member A [Source:HGNC Symbol;Acc:HGNC:13749]	159.6	191.5	**1.20**	0.04411
BMS1P10	BMS1, ribosome biogenesis factor pseudogene 10 [Source:HGNC Symbol;Acc:HGNC:49154]	162.1	196.1	**1.20**	0.04771
HADHA	hydroxyacyl-CoA dehydrogenase trifunctional multienzyme complex subunit alpha [Source:HGNC Symb	5386.4	6411.3	**1.19**	2.32E-13
RPS25	ribosomal protein S25 [Source:HGNC Symbol;Acc:HGNC:10413]	9752.8	11616.4	**1.19**	3.27E-12
RPL37	ribosomal protein L37 [Source:HGNC Symbol;Acc:HGNC:10347]	6819.0	8140.6	**1.19**	9.33E-12
STAU1	staufen double-stranded RNA binding protein 1 [Source:HGNC Symbol;Acc:HGNC:11370]	1967.8	2336.7	**1.19**	7.88E-11
C6orf89	chromosome 6 open reading frame 89 [Source:HGNC Symbol;Acc:HGNC:21114]	2932.7	3491.8	**1.19**	9.60E-11
RALGAPA1	Ral GTPase activating protein catalytic alpha subunit 1 [Source:HGNC Symbol;Acc:HGNC:17770]	1296.3	1543.4	**1.19**	3.73E-10
TXNDC9	thioredoxin domain containing 9 [Source:HGNC Symbol;Acc:HGNC:24110]	1698.0	2027.1	**1.19**	5.39E-10
OSBPL8	oxysterol binding protein like 8 [Source:HGNC Symbol;Acc:HGNC:16396]	5683.5	6790.0	**1.19**	6.12E-10
PRPF4B	pre-mRNA processing factor 4B [Source:HGNC Symbol;Acc:HGNC:17346]	2760.7	3275.6	**1.19**	1.71E-09
ATP5PD	ATP synthase peripheral stalk subunit d [Source:HGNC Symbol;Acc:HGNC:845]	2594.5	3093.9	**1.19**	6.68E-09
TOGARAM1	TOG array regulator of axonemal microtubules 1 [Source:HGNC Symbol;Acc:HGNC:19959]	1187.6	1407.5	**1.19**	7.99E-09
IFNAR1	interferon alpha and beta receptor subunit 1 [Source:HGNC Symbol;Acc:HGNC:5432]	2308.4	2756.4	**1.19**	8.54E-09
SERPINA3	serpin family A member 3 [Source:HGNC Symbol;Acc:HGNC:16]	19875.5	23313.0	**1.19**	1.47E-08
MORC3	MORC family CW-type zinc finger 3 [Source:HGNC Symbol;Acc:HGNC:23572]	1594.4	1898.8	**1.19**	2.03E-08
ACTR1A	ARP1 actin related protein 1 homolog A [Source:HGNC Symbol;Acc:HGNC:167]	1658.6	1981.0	**1.19**	2.78E-08
QSOX1	quiescin sulfhydryl oxidase 1 [Source:HGNC Symbol;Acc:HGNC:9756]	3051.9	3621.8	**1.19**	3.59E-08
KMT2E	lysine methyltransferase 2E [Source:HGNC Symbol;Acc:HGNC:18541]	2223.1	2639.3	**1.19**	4.47E-08
PINK1-AS	PINK1 antisense RNA [Source:HGNC Symbol;Acc:HGNC:38872]	2736.9	3265.7	**1.19**	8.76E-08
CHMP4A	charged multivesicular body protein 4A [Source:HGNC Symbol;Acc:HGNC:20274]	1087.5	1299.4	**1.19**	2.69E-07
AP3D1	adaptor related protein complex 3 subunit delta 1 [Source:HGNC Symbol;Acc:HGNC:568]	1892.9	2257.7	**1.19**	3.04E-07
FAM210B	family with sequence similarity 210 member B [Source:HGNC Symbol;Acc:HGNC:16102]	809.5	966.4	**1.19**	4.43E-07
RPL23	ribosomal protein L23 [Source:HGNC Symbol;Acc:HGNC:10316]	8119.9	9658.3	**1.19**	5.05E-07
LLPH	LLP homolog, long-term synaptic facilitation factor [Source:HGNC Symbol;Acc:HGNC:28229]	1038.7	1233.2	**1.19**	6.43E-07
CRTAC1	cartilage acidic protein 1 [Source:HGNC Symbol;Acc:HGNC:14882]	2722.0	3210.7	**1.19**	9.17E-07
ZRANB1	zinc finger RANBP2-type containing 1 [Source:HGNC Symbol;Acc:HGNC:18224]	1636.6	1947.0	**1.19**	1.57E-06
GALNT11	polypeptide N-acetylgalactosaminyltransferase 11 [Source:HGNC Symbol;Acc:HGNC:19875]	1968.9	2328.0	**1.19**	2.66E-06
CMTM6	CKLF like MARVEL transmembrane domain containing 6 [Source:HGNC Symbol;Acc:HGNC:19177]	1440.5	1722.1	**1.19**	2.82E-06
PAPOLG	poly(A) polymerase gamma [Source:HGNC Symbol;Acc:HGNC:14982]	631.4	751.5	**1.19**	5.49E-06
SPAG16	sperm associated antigen 16 [Source:HGNC Symbol;Acc:HGNC:23225]	710.9	842.5	**1.19**	5.95E-06
NBR1	NBR1, autophagy cargo receptor [Source:HGNC Symbol;Acc:HGNC:6746]	5211.6	6199.4	**1.19**	6.61E-06
PAN3	poly(A) specific ribonuclease subunit PAN3 [Source:HGNC Symbol;Acc:HGNC:29991]	706.6	841.9	**1.19**	6.61E-06
CLUAP1	clusterin associated protein 1 [Source:HGNC Symbol;Acc:HGNC:19009]	979.7	1162.3	**1.19**	1.12E-05
CWC25	CWC25 spliceosome associated protein homolog [Source:HGNC Symbol;Acc:HGNC:25989]	539.0	643.7	**1.19**	1.52E-05
CSPP1	centrosome and spindle pole associated protein 1 [Source:HGNC Symbol;Acc:HGNC:26193]	671.1	798.6	**1.19**	2.36E-05
ADI1	acireductone dioxygenase 1 [Source:HGNC Symbol;Acc:HGNC:30576]	890.9	1071.8	**1.19**	2.78E-05
DERA	deoxyribose-phosphate aldolase [Source:HGNC Symbol;Acc:HGNC:24269]	851.6	1014.9	**1.19**	3.72E-05
TF	transferrin [Source:HGNC Symbol;Acc:HGNC:11740]	1101.7	1331.6	**1.19**	3.73E-05

EIF2B1	eukaryotic translation initiation factor 2B subunit alpha [Source:HGNC Symbol;Acc:HGNC:3257]	861.3	1029.2	**1.19**	9.36E-05
DDX19B	DEAD-box helicase 19B [Source:HGNC Symbol;Acc:HGNC:2742]	501.4	594.6	**1.19**	0.0001072
KATNBL1	katanin regulatory subunit B1 like 1 [Source:HGNC Symbol;Acc:HGNC:26199]	363.4	432.0	**1.19**	0.0001345
IRGQ	immunity related GTPase Q [Source:HGNC Symbol;Acc:HGNC:24868]	874.6	1041.9	**1.19**	0.0002082
UNC45A	unc-45 myosin chaperone A [Source:HGNC Symbol;Acc:HGNC:30594]	764.5	911.7	**1.19**	0.0002615
POP4	POP4 homolog, ribonuclease P/MRP subunit [Source:HGNC Symbol;Acc:HGNC:30081]	737.2	871.3	**1.19**	0.0002933
SLC25A33	solute carrier family 25 member 33 [Source:HGNC Symbol;Acc:HGNC:29681]	343.9	410.9	**1.19**	0.0002979
POLR3E	RNA polymerase III subunit E [Source:HGNC Symbol;Acc:HGNC:30347]	503.9	598.1	**1.19**	0.0003392
CYSTM1	cysteine rich transmembrane module containing 1 [Source:HGNC Symbol;Acc:HGNC:30239]	612.9	730.8	**1.19**	0.0004538
AK3	adenylate kinase 3 [Source:HGNC Symbol;Acc:HGNC:17376]	815.0	977.9	**1.19**	0.0004634
LPCAT3	lysophosphatidylcholine acyltransferase 3 [Source:HGNC Symbol;Acc:HGNC:30244]	387.9	463.3	**1.19**	0.0005239
ZNF841	zinc finger protein 841 [Source:HGNC Symbol;Acc:HGNC:27611]	684.5	815.9	**1.19**	0.0005509
PANX1	pannexin 1 [Source:HGNC Symbol;Acc:HGNC:8599]	526.6	630.8	**1.19**	0.0008732
S100A11	S100 calcium binding protein A11 [Source:HGNC Symbol;Acc:HGNC:10488]	543.5	643.9	**1.19**	0.0009675
UTP14A	UTP14A, small subunit processome component [Source:HGNC Symbol;Acc:HGNC:10665]	666.4	792.5	**1.19**	0.00101
RSBN1	round spermatid basic protein 1 [Source:HGNC Symbol;Acc:HGNC:25642]	683.9	818.0	**1.19**	0.001155
TBCCD1	TBCC domain containing 1 [Source:HGNC Symbol;Acc:HGNC:25546]	320.6	380.5	**1.19**	0.001162
VSIG10	V-set and immunoglobulin domain containing 10 [Source:HGNC Symbol;Acc:HGNC:26078]	561.1	667.4	**1.19**	0.00186
ZNF22	zinc finger protein 22 [Source:HGNC Symbol;Acc:HGNC:13012]	627.5	752.4	**1.19**	0.001924
TRIM69	tripartite motif containing 69 [Source:HGNC Symbol;Acc:HGNC:17857]	477.9	570.1	**1.19**	0.002062
CEP170B	centrosomal protein 170B [Source:HGNC Symbol;Acc:HGNC:20362]	308.7	371.0	**1.19**	0.002221
ZFAND2A	zinc finger AN1-type containing 2A [Source:HGNC Symbol;Acc:HGNC:28073]	335.5	405.4	**1.19**	0.002956
MAN2B2	mannosidase alpha class 2B member 2 [Source:HGNC Symbol;Acc:HGNC:29623]	898.9	1078.7	**1.19**	0.003109
TRNAU1AP	tRNA selenocysteine 1 associated protein 1 [Source:HGNC Symbol;Acc:HGNC:30813]	426.2	506.1	**1.19**	0.003944
AAR2	AAR2 splicing factor homolog [Source:HGNC Symbol;Acc:HGNC:15886]	279.8	333.4	**1.19**	0.004024
SIRT5	sirtuin 5 [Source:HGNC Symbol;Acc:HGNC:14933]	440.0	528.1	**1.19**	0.004845
SPRYD7	SPRY domain containing 7 [Source:HGNC Symbol;Acc:HGNC:14297]	582.0	691.3	**1.19**	0.004901
RAP1A	RAP1A, member of RAS oncogene family [Source:HGNC Symbol;Acc:HGNC:9855]	1880.3	2237.0	**1.19**	0.005382
CHRAC1	chromatin accessibility complex 1 [Source:HGNC Symbol;Acc:HGNC:13544]	425.9	512.0	**1.19**	0.005606
LRRC6	leucine rich repeat containing 6 [Source:HGNC Symbol;Acc:HGNC:16725]	247.0	294.5	**1.19**	0.006095
MTHFD2L	methylenetetrahydrofolate dehydrogenase (NADP+ dependent) 2 like [Source:HGNC Symbol;Acc:HGNC	287.4	341.5	**1.19**	0.008302
USP19	ubiquitin specific peptidase 19 [Source:HGNC Symbol;Acc:HGNC:12617]	380.5	455.4	**1.19**	0.0086
URM1	ubiquitin related modifier 1 [Source:HGNC Symbol;Acc:HGNC:28378]	255.2	304.8	**1.19**	0.008721
MOCS1	molybdenum cofactor synthesis 1 [Source:HGNC Symbol;Acc:HGNC:7190]	278.0	332.5	**1.19**	0.009689
UBQLN2	ubiquilin 2 [Source:HGNC Symbol;Acc:HGNC:12509]	368.8	438.0	**1.19**	0.01151
PIK3CB	phosphatidylinositol-4,5-bisphosphate 3-kinase catalytic subunit beta [Source:HGNC Symbol;Acc:HGNC:	285.2	339.4	**1.19**	0.01169
LINC00294	long intergenic non-protein coding RNA 294 [Source:HGNC Symbol;Acc:HGNC:27456]	257.4	305.7	**1.19**	0.01179
FOXD1-AS1	FOXD1 antisense RNA 1 [Source:HGNC Symbol;Acc:HGNC:50658]	154.8	185.6	**1.19**	0.01455
EGFR	epidermal growth factor receptor [Source:HGNC Symbol;Acc:HGNC:3236]	863.5	1011.4	**1.19**	0.01757
ALS2CL	ALS2 C-terminal like [Source:HGNC Symbol;Acc:HGNC:20605]	284.2	338.1	**1.19**	0.01967
WDCP	WD repeat and coiled coil containing [Source:HGNC Symbol;Acc:HGNC:26157]	244.8	292.4	**1.19**	0.02303
IFNAR2	interferon alpha and beta receptor subunit 2 [Source:HGNC Symbol;Acc:HGNC:5433]	140.5	167.5	**1.19**	0.02981
ZBED8	zinc finger BED-type containing 8 [Source:HGNC Symbol;Acc:HGNC:30804]	194.5	231.1	**1.19**	0.03081
ZXDC	ZXD family zinc finger C [Source:HGNC Symbol;Acc:HGNC:28160]	390.6	462.5	**1.19**	0.0347
VRK3	vaccinia related kinase 3 [Source:HGNC Symbol;Acc:HGNC:18996]	148.6	178.1	**1.19**	0.04424
CXorf40A	chromosome X open reading frame 40A [Source:HGNC Symbol;Acc:HGNC:28089]	114.4	137.2	**1.19**	0.04904
CHD9	chromodomain helicase DNA binding protein 9 [Source:HGNC Symbol;Acc:HGNC:25701]	7412.0	8789.8	**1.18**	1.42E-14
RAB10	RAB10, member RAS oncogene family [Source:HGNC Symbol;Acc:HGNC:9759]	3654.6	4325.5	**1.18**	5.75E-12
RPL26	ribosomal protein L26 [Source:HGNC Symbol;Acc:HGNC:10327]	10275.1	12176.0	**1.18**	7.39E-12
WTAP	WT1 associated protein [Source:HGNC Symbol;Acc:HGNC:16846]	2316.1	2732.2	**1.18**	1.19E-11
ROCK2	Rho associated coiled-coil containing protein kinase 2 [Source:HGNC Symbol;Acc:HGNC:10252]	6660.3	7885.2	**1.18**	1.04E-10
CLPX	caseinolytic mitochondrial matrix peptidase chaperone subunit [Source:HGNC Symbol;Acc:HGNC:2088]	1639.6	1943.3	**1.18**	5.96E-10
BDP1	B double prime 1, subunit of RNA polymerase III transcription initiation factor IIIB [Source:HGNC Symbo	3390.0	4015.0	**1.18**	1.06E-09
GGNBP2	gametogenetin binding protein 2 [Source:HGNC Symbol;Acc:HGNC:19357]	1883.1	2232.0	**1.18**	1.36E-09
VPS29	VPS29, retromer complex component [Source:HGNC Symbol;Acc:HGNC:14340]	1715.7	2034.1	**1.18**	3.14E-09
NPTN	neuroplastin [Source:HGNC Symbol;Acc:HGNC:17867]	7680.9	9056.4	**1.18**	1.15E-08
ZNFX1	zinc finger NFX1-type containing 1 [Source:HGNC Symbol;Acc:HGNC:29271]	2796.4	3312.1	**1.18**	1.61E-08
USP47	ubiquitin specific peptidase 47 [Source:HGNC Symbol;Acc:HGNC:20076]	4400.0	5198.5	**1.18**	2.61E-08
NAE1	NEDD8 activating enzyme E1 subunit 1 [Source:HGNC Symbol;Acc:HGNC:621]	1683.8	1985.2	**1.18**	4.35E-08
EWSR1	EWS RNA binding protein 1 [Source:HGNC Symbol;Acc:HGNC:3508]	4368.7	5169.1	**1.18**	4.81E-08
STXBP3	syntaxin binding protein 3 [Source:HGNC Symbol;Acc:HGNC:11446]	2329.1	2759.1	**1.18**	8.26E-08
GPAT4	glycerol-3-phosphate acyltransferase 4 [Source:HGNC Symbol;Acc:HGNC:20880]	1365.1	1616.6	**1.18**	9.98E-08
TLN1	talin 1 [Source:HGNC Symbol;Acc:HGNC:11845]	7854.7	9333.5	**1.18**	1.12E-07
TNRC6A	trinucleotide repeat containing 6A [Source:HGNC Symbol;Acc:HGNC:11969]	1674.0	1977.9	**1.18**	3.07E-07
SPG11	SPG11, spatacsin vesicle trafficking associated [Source:HGNC Symbol;Acc:HGNC:11226]	2067.8	2447.0	**1.18**	6.88E-07
MFAP1	microfibril associated protein 1 [Source:HGNC Symbol;Acc:HGNC:7032]	1425.8	1684.9	**1.18**	1.14E-06
CDC42BPB	CDC42 binding protein kinase beta [Source:HGNC Symbol;Acc:HGNC:1738]	2010.0	2370.5	**1.18**	1.97E-06
GTF2H5	general transcription factor IIH subunit 5 [Source:HGNC Symbol;Acc:HGNC:21157]	751.5	888.3	**1.18**	2.44E-06
AP2B1	adaptor related protein complex 2 subunit beta 1 [Source:HGNC Symbol;Acc:HGNC:563]	2909.5	3429.8	**1.18**	3.95E-06
PSAP	prosaposin [Source:HGNC Symbol;Acc:HGNC:9498]	20051.7	23920.2	**1.18**	4.53E-06
DIP2B	disco interacting protein 2 homolog B [Source:HGNC Symbol;Acc:HGNC:29284]	917.3	1084.3	**1.18**	5.09E-06
LRCH3	leucine rich repeats and calponin homology domain containing 3 [Source:HGNC Symbol;Acc:HGNC:2863	1124.7	1329.7	**1.18**	5.41E-06

SPOP	speckle type BTB/POZ protein [Source:HGNC Symbol;Acc:HGNC:11254]	1170.1	1390.3	**1.18**	5.74E-06
MOB1A	MOB kinase activator 1A [Source:HGNC Symbol;Acc:HGNC:16015]	2700.0	3185.4	**1.18**	5.89E-06
RORA	RAR related orphan receptor A [Source:HGNC Symbol;Acc:HGNC:10258]	2465.7	2925.4	**1.18**	7.90E-06
FBXO21	F-box protein 21 [Source:HGNC Symbol;Acc:HGNC:13592]	849.6	1002.5	**1.18**	1.05E-05
DCTN2	dynactin subunit 2 [Source:HGNC Symbol;Acc:HGNC:2712]	1271.3	1512.4	**1.18**	1.07E-05
DCP2	decapping mRNA 2 [Source:HGNC Symbol;Acc:HGNC:24452]	1207.9	1431.2	**1.18**	1.19E-05
MGAT2	mannosyl (alpha-1,6-)-glycoprotein beta-1,2-N-acetylglucosaminyltransferase [Source:HGNC Symbol;Ac	1552.6	1835.3	**1.18**	1.60E-05
CDC26	cell division cycle 26 [Source:HGNC Symbol;Acc:HGNC:17839]	805.6	950.7	**1.18**	1.76E-05
RC3H1	ring finger and CCCH-type domains 1 [Source:HGNC Symbol;Acc:HGNC:29434]	1158.9	1372.8	**1.18**	2.02E-05
PLOD3	procollagen-lysine,2-oxoglutarate 5-dioxygenase 3 [Source:HGNC Symbol;Acc:HGNC:9083]	1496.2	1773.8	**1.18**	3.39E-05
DNAJC19	DnaJ heat shock protein family (Hsp40) member C19 [Source:HGNC Symbol;Acc:HGNC:30528]	871.6	1031.1	**1.18**	3.99E-05
FBXO33	F-box protein 33 [Source:HGNC Symbol;Acc:HGNC:19833]	766.3	909.3	**1.18**	6.26E-05
RNF34	ring finger protein 34 [Source:HGNC Symbol;Acc:HGNC:17297]	708.3	835.2	**1.18**	7.70E-05
RPS19BP1	ribosomal protein S19 binding protein 1 [Source:HGNC Symbol;Acc:HGNC:28749]	649.6	763.6	**1.18**	7.80E-05
ZNF397	zinc finger protein 397 [Source:HGNC Symbol;Acc:HGNC:18818]	1140.6	1351.5	**1.18**	0.0001112
ORMDL2	ORMDL sphingolipid biosynthesis regulator 2 [Source:HGNC Symbol;Acc:HGNC:16037]	670.3	793.2	**1.18**	0.0001143
KIAA1109	KIAA1109 [Source:HGNC Symbol;Acc:HGNC:26953]	2771.3	3268.9	**1.18**	0.0001347
RERE	arginine-glutamic acid dipeptide repeats [Source:HGNC Symbol;Acc:HGNC:9965]	601.4	710.0	**1.18**	0.0001396
ARL13B	ADP ribosylation factor like GTPase 13B [Source:HGNC Symbol;Acc:HGNC:25419]	578.9	685.4	**1.18**	0.0001604
HIRA	histone cell cycle regulator [Source:HGNC Symbol;Acc:HGNC:4916]	340.2	404.0	**1.18**	0.000248
PHYH	phytanoyl-CoA 2-hydroxylase [Source:HGNC Symbol;Acc:HGNC:8940]	468.3	554.2	**1.18**	0.0002636
ZNF143	zinc finger protein 143 [Source:HGNC Symbol;Acc:HGNC:12928]	622.5	736.6	**1.18**	0.0003139
CAMLG	calcium modulating ligand [Source:HGNC Symbol;Acc:HGNC:1471]	964.4	1141.7	**1.18**	0.0003249
SLC12A6	solute carrier family 12 member 6 [Source:HGNC Symbol;Acc:HGNC:10914]	2069.9	2439.8	**1.18**	0.0003278
GID8	GID complex subunit 8 homolog [Source:HGNC Symbol;Acc:HGNC:15857]	1032.8	1228.5	**1.18**	0.0005437
TDP2	tyrosyl-DNA phosphodiesterase 2 [Source:HGNC Symbol;Acc:HGNC:17768]	1216.7	1451.1	**1.18**	0.000644
CCDC113	coiled-coil domain containing 113 [Source:HGNC Symbol;Acc:HGNC:25002]	359.8	425.3	**1.18**	0.0006888
DCAF12	DDB1 and CUL4 associated factor 12 [Source:HGNC Symbol;Acc:HGNC:19911]	556.9	656.2	**1.18**	0.0008282
MYO9B	myosin IXB [Source:HGNC Symbol;Acc:HGNC:7609]	1021.5	1216.0	**1.18**	0.0009432
LIPA	lipase A, lysosomal acid type [Source:HGNC Symbol;Acc:HGNC:6617]	542.5	639.8	**1.18**	0.001273
ARFGAP2	ADP ribosylation factor GTPase activating protein 2 [Source:HGNC Symbol;Acc:HGNC:13504]	562.7	664.0	**1.18**	0.001419
FZD6	frizzled class receptor 6 [Source:HGNC Symbol;Acc:HGNC:4044]	711.8	841.0	**1.18**	0.001593
AC008878.3	Rho/Rac guanine nucleotide exchange factor 18 [Source:NCBI gene;Acc:23370]	426.2	505.2	**1.18**	0.001862
LSM6	LSM6 homolog, U6 small nuclear RNA and mRNA degradation associated [Source:HGNC Symbol;Acc:HG	421.8	499.9	**1.18**	0.002412
NLK	nemo like kinase [Source:HGNC Symbol;Acc:HGNC:29858]	322.3	382.5	**1.18**	0.002976
VPS51	VPS51, GARP complex subunit [Source:HGNC Symbol;Acc:HGNC:1172]	300.0	356.8	**1.18**	0.003958
CLEC16A	C-type lectin domain containing 16A [Source:HGNC Symbol;Acc:HGNC:29013]	261.5	308.5	**1.18**	0.004631
NPHP1	nephrocystin 1 [Source:HGNC Symbol;Acc:HGNC:7905]	229.2	270.5	**1.18**	0.00824
GMDS-DT	GMDS divergent transcript [Source:HGNC Symbol;Acc:HGNC:48993]	796.9	866.4	**1.18**	0.008243
RNPC3	RNA binding region (RNP1, RRM) containing 3 [Source:HGNC Symbol;Acc:HGNC:18666]	287.0	341.0	**1.18**	0.009032
SPOUT1	SPOUT domain containing methyltransferase 1 [Source:HGNC Symbol;Acc:HGNC:26933]	342.1	401.8	**1.18**	0.01226
SERPINB8	serpin family B member 8 [Source:HGNC Symbol;Acc:HGNC:8952]	267.2	315.7	**1.18**	0.01674
GPR157	G protein-coupled receptor 157 [Source:HGNC Symbol;Acc:HGNC:23687]	221.2	262.1	**1.18**	0.01821
LINC-PINT	long intergenic non-protein coding RNA, p53 induced transcript [Source:HGNC Symbol;Acc:HGNC:26885	316.9	377.8	**1.18**	0.01935
NHEJ1	non-homologous end joining factor 1 [Source:HGNC Symbol;Acc:HGNC:25737]	203.1	241.6	**1.18**	0.02508
FAM160B2	family with sequence similarity 160 member B2 [Source:HGNC Symbol;Acc:HGNC:16492]	192.4	227.2	**1.18**	0.02726
PDP2	pyruvate dehyrogenase phosphatase catalytic subunit 2 [Source:HGNC Symbol;Acc:HGNC:30263]	257.9	305.8	**1.18**	0.02748
MXD4	MAX dimerization protein 4 [Source:HGNC Symbol;Acc:HGNC:13906]	370.7	444.1	**1.18**	0.0376
HINFP	histone H4 transcription factor [Source:HGNC Symbol;Acc:HGNC:17850]	149.5	178.4	**1.18**	0.04927
GOLGA4	golgin A4 [Source:HGNC Symbol;Acc:HGNC:4427]	9344.6	10994.2	**1.17**	1.06E-11
SYPL1	synaptophysin like 1 [Source:HGNC Symbol;Acc:HGNC:11507]	1563.9	1833.0	**1.17**	6.62E-10
UBR2	ubiquitin protein ligase E3 component n-recognin 2 [Source:HGNC Symbol;Acc:HGNC:21289]	2350.4	2762.3	**1.17**	6.80E-10
MPDZ	multiple PDZ domain crumbs cell polarity complex component [Source:HGNC Symbol;Acc:HGNC:7208]	1550.1	1822.9	**1.17**	1.29E-09
TPD52L2	tumor protein D52 like 2 [Source:HGNC Symbol;Acc:HGNC:12007]	2437.3	2861.8	**1.17**	5.49E-09
TPR	translocated promoter region, nuclear basket protein [Source:HGNC Symbol;Acc:HGNC:12017]	7426.1	8707.4	**1.17**	7.51E-09
APMAP	adipocyte plasma membrane associated protein [Source:HGNC Symbol;Acc:HGNC:13238]	3373.2	3953.3	**1.17**	6.31E-08
SERP1	stress associated endoplasmic reticulum protein 1 [Source:HGNC Symbol;Acc:HGNC:10759]	3310.9	3882.3	**1.17**	7.43E-08
PSMA7	proteasome subunit alpha 7 [Source:HGNC Symbol;Acc:HGNC:9536]	2799.9	3278.3	**1.17**	9.00E-08
OSTF1	osteoclast stimulating factor 1 [Source:HGNC Symbol;Acc:HGNC:8510]	1023.7	1203.3	**1.17**	1.21E-07
SEC23A	Sec23 homolog A, coat complex II component [Source:HGNC Symbol;Acc:HGNC:10701]	9035.7	10581.4	**1.17**	1.44E-07
PIK3C2A	phosphatidylinositol-4-phosphate 3-kinase catalytic subunit type 2 alpha [Source:HGNC Symbol;Acc:HG	2505.7	2952.8	**1.17**	1.51E-07
CCAR1	cell division cycle and apoptosis regulator 1 [Source:HGNC Symbol;Acc:HGNC:24236]	2088.9	2452.1	**1.17**	2.18E-07
TBK1	TANK binding kinase 1 [Source:HGNC Symbol;Acc:HGNC:11584]	1350.0	1581.8	**1.17**	3.10E-07
PDCD2	programmed cell death 2 [Source:HGNC Symbol;Acc:HGNC:8762]	1160.8	1366.8	**1.17**	9.68E-07
RPL9	ribosomal protein L9 [Source:HGNC Symbol;Acc:HGNC:10369]	8603.9	10185.6	**1.17**	1.01E-06
MYL12B	myosin light chain 12B [Source:HGNC Symbol;Acc:HGNC:29827]	5887.1	6890.2	**1.17**	1.17E-06
ATG2B	autophagy related 2B [Source:HGNC Symbol;Acc:HGNC:20187]	1047.7	1231.2	**1.17**	1.28E-06
LRRC40	leucine rich repeat containing 40 [Source:HGNC Symbol;Acc:HGNC:26004]	849.6	999.4	**1.17**	1.61E-06
WDR47	WD repeat domain 47 [Source:HGNC Symbol;Acc:HGNC:29141]	1099.0	1283.8	**1.17**	2.01E-06
PIBF1	progesterone immunomodulatory binding factor 1 [Source:HGNC Symbol;Acc:HGNC:23352]	1483.1	1742.3	**1.17**	3.05E-06
NDUFV2	NADH:ubiquinone oxidoreductase core subunit V2 [Source:HGNC Symbol;Acc:HGNC:7717]	2756.0	3238.4	**1.17**	3.26E-06
VMP1	vacuole membrane protein 1 [Source:HGNC Symbol;Acc:HGNC:29559]	3294.2	3869.2	**1.17**	4.02E-06

TSG101	tumor susceptibility 101 [Source:HGNC Symbol;Acc:HGNC:15971]	1282.4	1512.8	**1.17**	7.37E-06
COG3	component of oligomeric golgi complex 3 [Source:HGNC Symbol;Acc:HGNC:18619]	1309.6	1536.5	**1.17**	7.85E-06
DROSHA	drosha ribonuclease III [Source:HGNC Symbol;Acc:HGNC:17904]	697.0	820.4	**1.17**	1.22E-05
MYO9A	myosin IXA [Source:HGNC Symbol;Acc:HGNC:7608]	3593.8	4213.8	**1.17**	1.27E-05
FAM126B	family with sequence similarity 126 member B [Source:HGNC Symbol;Acc:HGNC:28593]	645.8	757.8	**1.17**	1.36E-05
CEP70	centrosomal protein 70 [Source:HGNC Symbol;Acc:HGNC:29972]	1023.5	1199.6	**1.17**	1.73E-05
DNAJB12	DnaJ heat shock protein family (Hsp40) member B12 [Source:HGNC Symbol;Acc:HGNC:14891]	770.4	907.3	**1.17**	2.50E-05
PSMF1	proteasome inhibitor subunit 1 [Source:HGNC Symbol;Acc:HGNC:9571]	868.8	1018.2	**1.17**	3.27E-05
CDKN1B	cyclin dependent kinase inhibitor 1B [Source:HGNC Symbol;Acc:HGNC:1785]	713.9	839.5	**1.17**	3.75E-05
SNX4	sorting nexin 4 [Source:HGNC Symbol;Acc:HGNC:11175]	1435.4	1687.9	**1.17**	4.22E-05
RAPGEF6	Rap guanine nucleotide exchange factor 6 [Source:HGNC Symbol;Acc:HGNC:20655]	867.0	1021.3	**1.17**	4.78E-05
DMTF1	cyclin D binding myb like transcription factor 1 [Source:HGNC Symbol;Acc:HGNC:14603]	1315.8	1545.6	**1.17**	5.51E-05
RBM33	RNA binding motif protein 33 [Source:HGNC Symbol;Acc:HGNC:27223]	1613.9	1892.1	**1.17**	6.03E-05
ZDHHC16	zinc finger DHHC-type containing 16 [Source:HGNC Symbol;Acc:HGNC:20714]	523.5	614.8	**1.17**	6.70E-05
ATAD2B	ATPase family, AAA domain containing 2B [Source:HGNC Symbol;Acc:HGNC:29230]	766.9	902.0	**1.17**	6.75E-05
YWHAE	tyrosine 3-monooxygenase/tryptophan 5-monooxygenase activation protein epsilon [Source:HGNC Sym	11022.3	13055.8	**1.17**	7.21E-05
GTF2B	general transcription factor IIB [Source:HGNC Symbol;Acc:HGNC:4648]	1060.8	1239.9	**1.17**	7.21E-05
DAD1	defender against cell death 1 [Source:HGNC Symbol;Acc:HGNC:2664]	2827.9	3313.2	**1.17**	7.30E-05
SMPD1	sphingomyelin phosphodiesterase 1 [Source:HGNC Symbol;Acc:HGNC:11120]	896.5	1052.0	**1.17**	8.63E-05
AGO3	argonaute 3, RISC catalytic component [Source:HGNC Symbol;Acc:HGNC:18421]	1013.9	1192.4	**1.17**	9.01E-05
OTULIN	OTU deubiquitinase with linear linkage specificity [Source:HGNC Symbol;Acc:HGNC:25118]	1171.8	1378.2	**1.17**	9.32E-05
LMBRD1	LMBR1 domain containing 1 [Source:HGNC Symbol;Acc:HGNC:23038]	2125.2	2506.5	**1.17**	9.92E-05
STX8	syntaxin 8 [Source:HGNC Symbol;Acc:HGNC:11443]	588.8	692.9	**1.17**	0.0001055
GMCL1	germ cell-less, spermatogenesis associated 1 [Source:HGNC Symbol;Acc:HGNC:23843]	1170.0	1381.0	**1.17**	0.0001172
RNF10	ring finger protein 10 [Source:HGNC Symbol;Acc:HGNC:10055]	1297.0	1524.8	**1.17**	0.0001292
C14orf119	chromosome 14 open reading frame 119 [Source:HGNC Symbol;Acc:HGNC:20270]	680.3	794.8	**1.17**	0.0001308
SNX33	sorting nexin 33 [Source:HGNC Symbol;Acc:HGNC:28468]	714.4	839.1	**1.17**	0.000145
AGPAT3	1-acylglycerol-3-phosphate O-acyltransferase 3 [Source:HGNC Symbol;Acc:HGNC:326]	641.8	754.4	**1.17**	0.0002553
DYNC2H1	dynein cytoplasmic 2 heavy chain 1 [Source:HGNC Symbol;Acc:HGNC:2962]	2671.7	3148.5	**1.17**	0.0004048
COX6B1	cytochrome c oxidase subunit 6B1 [Source:HGNC Symbol;Acc:HGNC:2280]	679.7	794.1	**1.17**	0.0004531
EIF4ENIF1	eukaryotic translation initiation factor 4E nuclear import factor 1 [Source:HGNC Symbol;Acc:HGNC:1668	408.0	478.9	**1.17**	0.0005228
KRTCAP2	keratinocyte associated protein 2 [Source:HGNC Symbol;Acc:HGNC:28942]	855.1	1005.6	**1.17**	0.0005618
CDC42SE2	CDC42 small effector 2 [Source:HGNC Symbol;Acc:HGNC:18547]	783.4	917.8	**1.17**	0.0005826
WRN	Werner syndrome RecQ like helicase [Source:HGNC Symbol;Acc:HGNC:12791]	846.6	994.2	**1.17**	0.00065
UBE2F	ubiquitin conjugating enzyme E2 F (putative) [Source:HGNC Symbol;Acc:HGNC:12480]	478.4	562.0	**1.17**	0.0007211
UBXN2B	UBX domain protein 2B [Source:HGNC Symbol;Acc:HGNC:27035]	755.5	883.9	**1.17**	0.0008642
CUX1	cut like homeobox 1 [Source:HGNC Symbol;Acc:HGNC:2557]	1364.5	1610.3	**1.17**	0.0008652
MID1IP1	MID1 interacting protein 1 [Source:HGNC Symbol;Acc:HGNC:20715]	656.8	777.2	**1.17**	0.0009288
LSM14B	LSM family member 14B [Source:HGNC Symbol;Acc:HGNC:15887]	553.5	646.0	**1.17**	0.0009933
RNF220	ring finger protein 220 [Source:HGNC Symbol;Acc:HGNC:25552]	633.8	742.3	**1.17**	0.001037
ANKUB1	ankyrin repeat and ubiquitin domain containing 1 [Source:HGNC Symbol;Acc:HGNC:29642]	623.4	731.7	**1.17**	0.001127
ATL1	atlastin GTPase 1 [Source:HGNC Symbol;Acc:HGNC:11231]	602.1	702.9	**1.17**	0.001377
ARHGEF18	Rho/Rac guanine nucleotide exchange factor 18 [Source:HGNC Symbol;Acc:HGNC:17090]	456.7	537.3	**1.17**	0.001383
CHML	CHM like, Rab escort protein 2 [Source:HGNC Symbol;Acc:HGNC:1941]	783.2	924.8	**1.17**	0.001532
DYNC1H1	dynein cytoplasmic 1 heavy chain 1 [Source:HGNC Symbol;Acc:HGNC:2961]	9894.1	11658.4	**1.17**	0.001563
CDK11B	cyclin dependent kinase 11B [Source:HGNC Symbol;Acc:HGNC:1729]	361.8	424.8	**1.17**	0.00174
GMPPA	GDP-mannose pyrophosphorylase A [Source:HGNC Symbol;Acc:HGNC:22923]	438.2	514.4	**1.17**	0.001792
CNOT11	CCR4-NOT transcription complex subunit 11 [Source:HGNC Symbol;Acc:HGNC:25217]	610.8	719.8	**1.17**	0.001893
LIG4	DNA ligase 4 [Source:HGNC Symbol;Acc:HGNC:6601]	813.3	956.9	**1.17**	0.001997
ENTPD7	ectonucleoside triphosphate diphosphohydrolase 7 [Source:HGNC Symbol;Acc:HGNC:19745]	695.6	818.4	**1.17**	0.002017
ARMCX3-AS1	ARMCX3 antisense RNA 1 [Source:HGNC Symbol;Acc:HGNC:41038]	326.0	382.3	**1.17**	0.002538
MED29	mediator complex subunit 29 [Source:HGNC Symbol;Acc:HGNC:23074]	495.4	585.3	**1.17**	0.00254
CHI3L2	chitinase 3 like 2 [Source:HGNC Symbol;Acc:HGNC:1933]	14655.2	17652.5	**1.17**	0.002558
MCCC1	methylcrotonoyl-CoA carboxylase 1 [Source:HGNC Symbol;Acc:HGNC:6936]	591.7	699.3	**1.17**	0.003043
SEC16A	SEC16 homolog A, endoplasmic reticulum export factor [Source:HGNC Symbol;Acc:HGNC:29006]	839.3	984.3	**1.17**	0.003697
PPP1R21	protein phosphatase 1 regulatory subunit 21 [Source:HGNC Symbol;Acc:HGNC:30595]	403.4	473.9	**1.17**	0.004443
SH3GL1	SH3 domain containing GRB2 like 1, endophilin A2 [Source:HGNC Symbol;Acc:HGNC:10830]	462.8	545.7	**1.17**	0.007129
FAM214A	family with sequence similarity 214 member A [Source:HGNC Symbol;Acc:HGNC:25609]	331.3	390.0	**1.17**	0.009156
SAT2	spermidine/spermine N1-acetyltransferase family member 2 [Source:HGNC Symbol;Acc:HGNC:23160]	249.6	294.2	**1.17**	0.00921
ACSF3	acyl-CoA synthetase family member 3 [Source:HGNC Symbol;Acc:HGNC:27288]	205.7	241.0	**1.17**	0.01093
CDK8	cyclin dependent kinase 8 [Source:HGNC Symbol;Acc:HGNC:1779]	364.3	427.3	**1.17**	0.01402
CRLF1	cytokine receptor like factor 1 [Source:HGNC Symbol;Acc:HGNC:2364]	411.4	484.2	**1.17**	0.01454
KIAA1328	KIAA1328 [Source:HGNC Symbol;Acc:HGNC:29248]	224.0	261.5	**1.17**	0.03459
PHKG1	phosphorylase kinase catalytic subunit gamma 1 [Source:HGNC Symbol;Acc:HGNC:8930]	178.6	209.9	**1.17**	0.03497
SUSD1	sushi domain containing 1 [Source:HGNC Symbol;Acc:HGNC:25413]	200.2	235.8	**1.17**	0.03781
RNF39	ring finger protein 39 [Source:HGNC Symbol;Acc:HGNC:18064]	189.7	222.2	**1.17**	0.04208
TLCD2	TLC domain containing 2 [Source:HGNC Symbol;Acc:HGNC:33522]	316.1	362.7	**1.17**	0.04438
RBM39	RNA binding motif protein 39 [Source:HGNC Symbol;Acc:HGNC:15923]	8036.9	9352.8	**1.16**	3.36E-11
OS9	OS9, endoplasmic reticulum lectin [Source:HGNC Symbol;Acc:HGNC:16994]	7600.2	8894.9	**1.16**	4.70E-10
SPECC1L	sperm antigen with calponin homology and coiled-coil domains 1 like [Source:HGNC Symbol;Acc:HGNC::	2611.1	3046.3	**1.16**	6.50E-10
ATF6	activating transcription factor 6 [Source:HGNC Symbol;Acc:HGNC:791]	2115.0	2463.7	**1.16**	2.13E-09
AP3B1	adaptor related protein complex 3 subunit beta 1 [Source:HGNC Symbol;Acc:HGNC:566]	4078.1	4742.6	**1.16**	3.37E-09

SPECC1L-ADORA2/	SPECC1L-ADORA2A readthrough (NMD candidate) [Source:HGNC Symbol;Acc:HGNC:49185]	2254.2	2628.9	**1.16**	1.78E-08
CHMP2A	charged multivesicular body protein 2A [Source:HGNC Symbol;Acc:HGNC:30216]	1629.2	1898.3	**1.16**	2.37E-08
RPL38	ribosomal protein L38 [Source:HGNC Symbol;Acc:HGNC:10349]	7011.7	8155.0	**1.16**	2.72E-08
NCOA4	nuclear receptor coactivator 4 [Source:HGNC Symbol;Acc:HGNC:7671]	3651.1	4251.6	**1.16**	3.29E-08
EIF4E2	eukaryotic translation initiation factor 4E family member 2 [Source:HGNC Symbol;Acc:HGNC:3293]	2116.5	2468.0	**1.16**	4.73E-08
LSM14A	LSM14A, mRNA processing body assembly factor [Source:HGNC Symbol;Acc:HGNC:24489]	2599.5	3040.6	**1.16**	5.10E-08
ZNF207	zinc finger protein 207 [Source:HGNC Symbol;Acc:HGNC:12998]	2903.3	3397.1	**1.16**	6.03E-08
VPS39	VPS39, HOPS complex subunit [Source:HGNC Symbol;Acc:HGNC:20593]	1185.2	1384.9	**1.16**	6.45E-08
CHURC1	churchill domain containing 1 [Source:HGNC Symbol;Acc:HGNC:20099]	2282.3	2669.4	**1.16**	8.89E-08
SMC5	structural maintenance of chromosomes 5 [Source:HGNC Symbol;Acc:HGNC:20465]	2285.0	2654.4	**1.16**	1.21E-07
RPL5	ribosomal protein L5 [Source:HGNC Symbol;Acc:HGNC:10360]	13938.8	16302.5	**1.16**	1.50E-07
PRPF39	pre-mRNA processing factor 39 [Source:HGNC Symbol;Acc:HGNC:20314]	1363.6	1587.8	**1.16**	1.53E-07
UTP6	UTP6, small subunit processome component [Source:HGNC Symbol;Acc:HGNC:18279]	2196.3	2554.8	**1.16**	1.56E-07
SEC11C	SEC11 homolog C, signal peptidase complex subunit [Source:HGNC Symbol;Acc:HGNC:23400]	2657.7	3093.2	**1.16**	2.25E-07
SNW1	SNW domain containing 1 [Source:HGNC Symbol;Acc:HGNC:16696]	2120.9	2471.5	**1.16**	4.12E-07
RAB3GAP2	RAB3 GTPase activating non-catalytic protein subunit 2 [Source:HGNC Symbol;Acc:HGNC:17168]	2850.5	3308.4	**1.16**	8.78E-07
UBAP2L	ubiquitin associated protein 2 like [Source:HGNC Symbol;Acc:HGNC:29877]	1679.5	1954.0	**1.16**	1.65E-06
RBM5	RNA binding motif protein 5 [Source:HGNC Symbol;Acc:HGNC:9902]	2595.5	3019.1	**1.16**	1.71E-06
DDX52	DExD-box helicase 52 [Source:HGNC Symbol;Acc:HGNC:20038]	1084.9	1262.1	**1.16**	1.73E-06
ADK	adenosine kinase [Source:HGNC Symbol;Acc:HGNC:257]	2540.7	2972.2	**1.16**	1.96E-06
RPL34	ribosomal protein L34 [Source:HGNC Symbol;Acc:HGNC:10340]	8463.8	9843.0	**1.16**	2.31E-06
CAPN7	calpain 7 [Source:HGNC Symbol;Acc:HGNC:1484]	1526.8	1780.2	**1.16**	5.40E-06
WDFY1	WD repeat and FYVE domain containing 1 [Source:HGNC Symbol;Acc:HGNC:20451]	3502.1	4088.8	**1.16**	5.58E-06
RRN3	RRN3 homolog, RNA polymerase I transcription factor [Source:HGNC Symbol;Acc:HGNC:30346]	1733.4	2023.0	**1.16**	6.50E-06
PARVA	parvin alpha [Source:HGNC Symbol;Acc:HGNC:14652]	2355.5	2742.2	**1.16**	1.26E-05
FBXO3	F-box protein 3 [Source:HGNC Symbol;Acc:HGNC:13582]	1286.4	1506.8	**1.16**	1.39E-05
PLLP	plasmolipin [Source:HGNC Symbol;Acc:HGNC:18553]	1813.5	2108.3	**1.16**	1.42E-05
ATXN7L3B	ataxin 7 like 3B [Source:HGNC Symbol;Acc:HGNC:37931]	1482.8	1722.6	**1.16**	2.18E-05
PUM1	pumilio RNA binding family member 1 [Source:HGNC Symbol;Acc:HGNC:14957]	1411.9	1643.6	**1.16**	2.27E-05
YWHAB	tyrosine 3-monooxygenase/tryptophan 5-monooxygenase activation protein beta [Source:HGNC Symbc	4970.2	5827.3	**1.16**	3.11E-05
ACTR2	ARP2 actin related protein 2 homolog [Source:HGNC Symbol;Acc:HGNC:169]	7109.7	8330.5	**1.16**	3.75E-05
SYF2	SYF2 pre-mRNA splicing factor [Source:HGNC Symbol;Acc:HGNC:19824]	1928.4	2261.4	**1.16**	3.88E-05
SPATA13	spermatogenesis associated 13 [Source:HGNC Symbol;Acc:HGNC:23222]	1182.8	1379.4	**1.16**	4.99E-05
COL6A2	collagen type VI alpha 2 chain [Source:HGNC Symbol;Acc:HGNC:2212]	18723.6	21812.7	**1.16**	5.15E-05
BICC1	BicC family RNA binding protein 1 [Source:HGNC Symbol;Acc:HGNC:19351]	1311.7	1527.6	**1.16**	6.36E-05
WASHC5	WASH complex subunit 5 [Source:HGNC Symbol;Acc:HGNC:28984]	1375.7	1600.2	**1.16**	7.13E-05
TAF1	TATA-box binding protein associated factor 1 [Source:HGNC Symbol;Acc:HGNC:11535]	1357.0	1578.1	**1.16**	0.0001053
FAM199X	family with sequence similarity 199, X-linked [Source:HGNC Symbol;Acc:HGNC:25195]	1311.9	1525.8	**1.16**	0.0001199
TMEM9B	TMEM9 domain family member B [Source:HGNC Symbol;Acc:HGNC:1168]	1186.4	1378.1	**1.16**	0.0001265
CCAR2	cell cycle and apoptosis regulator 2 [Source:HGNC Symbol;Acc:HGNC:23360]	817.9	952.6	**1.16**	0.0001506
CCDC117	coiled-coil domain containing 117 [Source:HGNC Symbol;Acc:HGNC:26599]	759.1	883.1	**1.16**	0.0001797
DBI	diazepam binding inhibitor, acyl-CoA binding protein [Source:HGNC Symbol;Acc:HGNC:2690]	2265.5	2653.4	**1.16**	0.0001803
CD81	CD81 molecule [Source:HGNC Symbol;Acc:HGNC:1701]	1022.5	1208.8	**1.16**	0.0001816
CDK14	cyclin dependent kinase 14 [Source:HGNC Symbol;Acc:HGNC:8883]	1328.1	1540.1	**1.16**	0.0002162
ZBTB10	zinc finger and BTB domain containing 10 [Source:HGNC Symbol;Acc:HGNC:30953]	1675.2	1940.3	**1.16**	0.0002671
TRIM4	tripartite motif containing 4 [Source:HGNC Symbol;Acc:HGNC:16275]	1237.6	1441.2	**1.16**	0.0003808
AKIRIN1	akirin 1 [Source:HGNC Symbol;Acc:HGNC:25744]	774.1	901.1	**1.16**	0.0004506
NCOR1	nuclear receptor corepressor 1 [Source:HGNC Symbol;Acc:HGNC:7672]	2962.0	3460.0	**1.16**	0.0004513
ZBTB33	zinc finger and BTB domain containing 33 [Source:HGNC Symbol;Acc:HGNC:16682]	701.3	818.2	**1.16**	0.0005238
FAM228B	family with sequence similarity 228 member B [Source:HGNC Symbol;Acc:HGNC:24736]	592.5	687.7	**1.16**	0.0005545
CHD2	chromodomain helicase DNA binding protein 2 [Source:HGNC Symbol;Acc:HGNC:1917]	4011.9	4687.0	**1.16**	0.0005935
DAZAP1	DAZ associated protein 1 [Source:HGNC Symbol;Acc:HGNC:2683]	965.8	1123.7	**1.16**	0.0006035
PITRM1-AS1	PITRM1 antisense RNA 1 [Source:HGNC Symbol;Acc:HGNC:44675]	503.6	589.5	**1.16**	0.0006358
PABPC1	poly(A) binding protein cytoplasmic 1 [Source:HGNC Symbol;Acc:HGNC:8554]	3183.5	3706.0	**1.16**	0.0006381
ARHGEF11	Rho guanine nucleotide exchange factor 11 [Source:HGNC Symbol;Acc:HGNC:14580]	892.3	1041.9	**1.16**	0.0006913
UVRAG	UV radiation resistance associated [Source:HGNC Symbol;Acc:HGNC:12640]	462.4	537.1	**1.16**	0.0007082
LRP10	LDL receptor related protein 10 [Source:HGNC Symbol;Acc:HGNC:14553]	3916.4	4616.2	**1.16**	0.0007415
MEX3C	mex-3 RNA binding family member C [Source:HGNC Symbol;Acc:HGNC:28040]	1451.8	1696.5	**1.16**	0.0007835
ADD3	adducin 3 [Source:HGNC Symbol;Acc:HGNC:245]	1035.6	1201.1	**1.16**	0.0008107
TMEM243	transmembrane protein 243 [Source:HGNC Symbol;Acc:HGNC:21707]	438.6	512.6	**1.16**	0.0008572
SPCS2	signal peptidase complex subunit 2 [Source:HGNC Symbol;Acc:HGNC:28962]	1959.7	2298.4	**1.16**	0.0009784
UBXN1	UBX domain protein 1 [Source:HGNC Symbol;Acc:HGNC:18402]	738.4	865.5	**1.16**	0.00112
TMEM126A	transmembrane protein 126A [Source:HGNC Symbol;Acc:HGNC:25382]	403.7	470.9	**1.16**	0.001446
ARID2	AT-rich interaction domain 2 [Source:HGNC Symbol;Acc:HGNC:18037]	604.7	706.4	**1.16**	0.001479
FAM120B	family with sequence similarity 120B [Source:HGNC Symbol;Acc:HGNC:21109]	829.3	967.7	**1.16**	0.001839
SH3TC1	SH3 domain and tetratricopeptide repeats 1 [Source:HGNC Symbol;Acc:HGNC:26009]	430.5	501.6	**1.16**	0.001847
DHRS7B	dehydrogenase/reductase 7B [Source:HGNC Symbol;Acc:HGNC:24547]	336.3	391.4	**1.16**	0.002319
MTFR1	mitochondrial fission regulator 1 [Source:HGNC Symbol;Acc:HGNC:29510]	768.4	897.5	**1.16**	0.002481
NTAN1	N-terminal asparagine amidase [Source:HGNC Symbol;Acc:HGNC:29909]	831.4	974.5	**1.16**	0.002552
AIFM1	apoptosis inducing factor mitochondria associated 1 [Source:HGNC Symbol;Acc:HGNC:8768]	468.9	549.5	**1.16**	0.002665
ATP6V0A1	ATPase H+ transporting V0 subunit a1 [Source:HGNC Symbol;Acc:HGNC:865]	868.4	1015.8	**1.16**	0.002779
GK5	glycerol kinase 5 (putative) [Source:HGNC Symbol;Acc:HGNC:28635]	483.2	565.5	**1.16**	0.002863

ARMCX5	armadillo repeat containing X-linked 5 [Source:HGNC Symbol;Acc:HGNC:25772]	386.8	450.4	**1.16**	0.003549
MCC	mutated in colorectal cancers [Source:HGNC Symbol;Acc:HGNC:6935]	617.1	719.4	**1.16**	0.003922
HPS3	HPS3, biogenesis of lysosomal organelles complex 2 subunit 1 [Source:HGNC Symbol;Acc:HGNC:15597]	1820.2	2165.7	**1.16**	0.004394
EMG1	EMG1, N1-specific pseudouridine methyltransferase [Source:HGNC Symbol;Acc:HGNC:16912]	924.3	1120.5	**1.16**	0.004592
DHX57	DExH-box helicase 57 [Source:HGNC Symbol;Acc:HGNC:20086]	602.0	703.8	**1.16**	0.004883
VPS18	VPS18, CORVET/HOPS core subunit [Source:HGNC Symbol;Acc:HGNC:15972]	372.2	433.6	**1.16**	0.00586
MINK1	misshapen like kinase 1 [Source:HGNC Symbol;Acc:HGNC:17565]	402.1	467.8	**1.16**	0.007902
C11orf98	chromosome 11 open reading frame 98 [Source:HGNC Symbol;Acc:HGNC:51238]	349.7	408.2	**1.16**	0.008265
GALNS	galactosamine (N-acetyl)-6-sulfatase [Source:HGNC Symbol;Acc:HGNC:4122]	278.5	324.7	**1.16**	0.0114
MIPEP	mitochondrial intermediate peptidase [Source:HGNC Symbol;Acc:HGNC:7104]	332.7	386.5	**1.16**	0.01226
TOMM40L	translocase of outer mitochondrial membrane 40 like [Source:HGNC Symbol;Acc:HGNC:25756]	352.7	410.3	**1.16**	0.01352
RRAS	RAS related [Source:HGNC Symbol;Acc:HGNC:10447]	254.0	295.8	**1.16**	0.01466
DRG2	developmentally regulated GTP binding protein 2 [Source:HGNC Symbol;Acc:HGNC:3030]	290.5	341.3	**1.16**	0.01551
MFSD12	major facilitator superfamily domain containing 12 [Source:HGNC Symbol;Acc:HGNC:28299]	220.4	258.3	**1.16**	0.01618
CRAMP1	cramped chromatin regulator homolog 1 [Source:HGNC Symbol;Acc:HGNC:14122]	239.9	280.1	**1.16**	0.01751
SPATS2	spermatogenesis associated serine rich 2 [Source:HGNC Symbol;Acc:HGNC:18650]	295.5	347.5	**1.16**	0.01925
PIGA	phosphatidylinositol glycan anchor biosynthesis class A [Source:HGNC Symbol;Acc:HGNC:8957]	309.1	359.0	**1.16**	0.02325
DMWD	DM1 locus, WD repeat containing [Source:HGNC Symbol;Acc:HGNC:2936]	269.9	316.1	**1.16**	0.02341
ERCC6	ERCC excision repair 6, chromatin remodeling factor [Source:HGNC Symbol;Acc:HGNC:3438]	427.9	500.1	**1.16**	0.02367
TPM3P9	tropomyosin 3 pseudogene 9 [Source:HGNC Symbol;Acc:HGNC:44142]	275.8	324.8	**1.16**	0.02558
RPAP1	RNA polymerase II associated protein 1 [Source:HGNC Symbol;Acc:HGNC:24567]	207.0	241.8	**1.16**	0.0268
FYCO1	FYVE and coiled-coil domain containing 1 [Source:HGNC Symbol;Acc:HGNC:14673]	511.9	598.2	**1.16**	0.02833
MAST2	microtubule associated serine/threonine kinase 2 [Source:HGNC Symbol;Acc:HGNC:19035]	336.5	388.6	**1.16**	0.03276
GAS8	growth arrest specific 8 [Source:HGNC Symbol;Acc:HGNC:4166]	214.8	249.8	**1.16**	0.03361
MAU2	MAU2 sister chromatid cohesion factor [Source:HGNC Symbol;Acc:HGNC:29140]	294.5	344.6	**1.16**	0.03497
STRADA	STE20-related kinase adaptor alpha [Source:HGNC Symbol;Acc:HGNC:30172]	206.5	239.4	**1.16**	0.0359
KDM4B	lysine demethylase 4B [Source:HGNC Symbol;Acc:HGNC:29136]	268.3	314.0	**1.16**	0.03863
WDR27	WD repeat domain 27 [Source:HGNC Symbol;Acc:HGNC:21248]	200.9	233.0	**1.16**	0.04033
TMEM41A	transmembrane protein 41A [Source:HGNC Symbol;Acc:HGNC:30544]	233.6	272.7	**1.16**	0.0408
C8orf76	chromosome 8 open reading frame 76 [Source:HGNC Symbol;Acc:HGNC:25924]	207.4	241.9	**1.16**	0.04435
STK36	serine/threonine kinase 36 [Source:HGNC Symbol;Acc:HGNC:17209]	151.9	177.7	**1.16**	0.04664
MESD	mesoderm development LRP chaperone [Source:HGNC Symbol;Acc:HGNC:13520]	2357.0	2738.6	**1.16**	2.12E-10
EFCAB14	EF-hand calcium binding domain 14 [Source:HGNC Symbol;Acc:HGNC:29051]	2383.1	2764.3	**1.16**	4.11E-10
EFR3A	EFR3 homolog A [Source:HGNC Symbol;Acc:HGNC:28970]	2757.6	3202.8	**1.16**	6.92E-10
GIGYF2	GRB10 interacting GYF protein 2 [Source:HGNC Symbol;Acc:HGNC:11960]	3887.2	4490.0	**1.16**	1.78E-09
BTAF1	B-TFIID TATA-box binding protein associated factor 1 [Source:HGNC Symbol;Acc:HGNC:17307]	1986.7	2298.9	**1.16**	6.57E-09
RPL32	ribosomal protein L32 [Source:HGNC Symbol;Acc:HGNC:10336]	7220.6	8353.3	**1.16**	1.67E-08
FGD5-AS1	FGD5 antisense RNA 1 [Source:HGNC Symbol;Acc:HGNC:40410]	4377.4	5089.9	**1.16**	1.73E-08
ARPC3	actin related protein 2/3 complex subunit 3 [Source:HGNC Symbol;Acc:HGNC:706]	5609.9	6492.5	**1.16**	2.63E-08
CSDE1	cold shock domain containing E1 [Source:HGNC Symbol;Acc:HGNC:29905]	21037.6	24413.4	**1.16**	6.26E-08
ANKLE2	ankyrin repeat and LEM domain containing 2 [Source:HGNC Symbol;Acc:HGNC:29101]	2219.3	2564.7	**1.16**	1.61E-07
CRKL	CRK like proto-oncogene, adaptor protein [Source:HGNC Symbol;Acc:HGNC:2363]	2112.7	2443.8	**1.16**	1.73E-07
CCDC186	coiled-coil domain containing 186 [Source:HGNC Symbol;Acc:HGNC:24349]	1920.3	2231.0	**1.16**	2.22E-07
PTK2	protein tyrosine kinase 2 [Source:HGNC Symbol;Acc:HGNC:9611]	3389.4	3941.6	**1.16**	3.07E-07
KARS	lysyl-tRNA synthetase [Source:HGNC Symbol;Acc:HGNC:6215]	3399.1	3933.3	**1.16**	3.35E-07
PHF20L1	PHD finger protein 20 like 1 [Source:HGNC Symbol;Acc:HGNC:24280]	1812.0	2091.8	**1.16**	4.54E-07
KRIT1	KRIT1, ankyrin repeat containing [Source:HGNC Symbol;Acc:HGNC:1573]	1779.5	2065.3	**1.16**	6.25E-07
SH3D19	SH3 domain containing 19 [Source:HGNC Symbol;Acc:HGNC:30418]	11340.7	13127.6	**1.16**	1.02E-06
AQR	aquarius intron-binding spliceosomal factor [Source:HGNC Symbol;Acc:HGNC:29513]	1864.1	2157.1	**1.16**	1.44E-06
SOCS4	suppressor of cytokine signaling 4 [Source:HGNC Symbol;Acc:HGNC:19392]	1626.4	1890.7	**1.16**	1.50E-06
HSD17B4	hydroxysteroid 17-beta dehydrogenase 4 [Source:HGNC Symbol;Acc:HGNC:5213]	3335.7	3867.2	**1.16**	5.36E-06
CLDND1	claudin domain containing 1 [Source:HGNC Symbol;Acc:HGNC:1322]	1719.1	1989.8	**1.16**	6.91E-06
AAGAB	alpha and gamma adaptin binding protein [Source:HGNC Symbol;Acc:HGNC:25662]	1441.6	1668.5	**1.16**	1.06E-05
SLC25A46	solute carrier family 25 member 46 [Source:HGNC Symbol;Acc:HGNC:25198]	2016.8	2327.6	**1.16**	1.10E-05
ARL2BP	ADP ribosylation factor like GTPase 2 binding protein [Source:HGNC Symbol;Acc:HGNC:17146]	1939.8	2243.5	**1.16**	1.10E-05
ZBTB38	zinc finger and BTB domain containing 38 [Source:HGNC Symbol;Acc:HGNC:26636]	2474.5	2858.7	**1.16**	1.11E-05
SMARCC1	SWI/SNF related, matrix associated, actin dependent regulator of chromatin subfamily c member 1 [Sou	1416.4	1638.1	**1.16**	1.24E-05
PRPF3	pre-mRNA processing factor 3 [Source:HGNC Symbol;Acc:HGNC:17348]	1737.3	2007.9	**1.16**	1.24E-05
PRPF38B	pre-mRNA processing factor 38B [Source:HGNC Symbol;Acc:HGNC:25512]	1110.2	1285.7	**1.16**	1.34E-05
ECHDC1	ethylmalonyl-CoA decarboxylase 1 [Source:HGNC Symbol;Acc:HGNC:21489]	1390.2	1607.8	**1.16**	1.55E-05
EIF4A1	eukaryotic translation initiation factor 4A1 [Source:HGNC Symbol;Acc:HGNC:3282]	13953.8	16142.5	**1.16**	1.91E-05
TMOD3	tropomodulin 3 [Source:HGNC Symbol;Acc:HGNC:11873]	4977.3	5765.5	**1.16**	2.00E-05
RUFY3	RUN and FYVE domain containing 3 [Source:HGNC Symbol;Acc:HGNC:30285]	965.2	1115.5	**1.16**	2.26E-05
SENP3-EIF4A1	SENP3-EIF4A1 readthrough (NMD candidate) [Source:HGNC Symbol;Acc:HGNC:49182]	14042.2	16203.7	**1.16**	2.42E-05
ACAA2	acetyl-CoA acyltransferase 2 [Source:HGNC Symbol;Acc:HGNC:83]	823.4	957.4	**1.16**	3.45E-05
DRG1	developmentally regulated GTP binding protein 1 [Source:HGNC Symbol;Acc:HGNC:3029]	1272.6	1469.6	**1.16**	4.50E-05
GALK2	galactokinase 2 [Source:HGNC Symbol;Acc:HGNC:4119]	1139.7	1315.5	**1.16**	6.60E-05
EDEM3	ER degradation enhancing alpha-mannosidase like protein 3 [Source:HGNC Symbol;Acc:HGNC:16787]	6687.7	7707.4	**1.16**	9.78E-05
TRIOBP	TRIO and F-actin binding protein [Source:HGNC Symbol;Acc:HGNC:17009]	874.6	1014.7	**1.16**	0.0001117
RB1CC1	RB1 inducible coiled-coil 1 [Source:HGNC Symbol;Acc:HGNC:15574]	3311.2	3862.7	**1.16**	0.0001329
MMGT1	membrane magnesium transporter 1 [Source:HGNC Symbol;Acc:HGNC:28100]	1065.7	1231.3	**1.16**	0.0001407
MCFD2	multiple coagulation factor deficiency 2 [Source:HGNC Symbol;Acc:HGNC:18451]	10987.8	12587.9	**1.16**	0.0001436

OSBPL9	oxysterol binding protein like 9 [Source:HGNC Symbol;Acc:HGNC:16386]	3522.7	4067.8	**1.16**	0.0001459
LGALS8	galectin 8 [Source:HGNC Symbol;Acc:HGNC:6569]	1788.6	2067.8	**1.16**	0.0001844
VKORC1L1	vitamin K epoxide reductase complex subunit 1 like 1 [Source:HGNC Symbol;Acc:HGNC:21492]	804.8	934.4	**1.16**	0.0001873
TRAF3IP1	TRAF3 interacting protein 1 [Source:HGNC Symbol;Acc:HGNC:17861]	492.5	570.1	**1.16**	0.0001937
DYNC1LI1	dynein cytoplasmic 1 light intermediate chain 1 [Source:HGNC Symbol;Acc:HGNC:18745]	1438.6	1674.7	**1.16**	0.0002312
HECA	hdc homolog, cell cycle regulator [Source:HGNC Symbol;Acc:HGNC:21041]	1685.6	1947.8	**1.16**	0.0002431
USP38	ubiquitin specific peptidase 38 [Source:HGNC Symbol;Acc:HGNC:20067]	682.4	789.2	**1.16**	0.0002464
BCL2L13	BCL2 like 13 [Source:HGNC Symbol;Acc:HGNC:17164]	1137.5	1318.4	**1.16**	0.0002637
BRAF	B-Raf proto-oncogene, serine/threonine kinase [Source:HGNC Symbol;Acc:HGNC:1097]	1408.3	1635.4	**1.16**	0.0002731
NLN	neurolysin [Source:HGNC Symbol;Acc:HGNC:16058]	1178.4	1366.3	**1.16**	0.0003298
SSR2	signal sequence receptor subunit 2 [Source:HGNC Symbol;Acc:HGNC:11324]	2478.1	2868.6	**1.16**	0.0003581
IFT57	intraflagellar transport 57 [Source:HGNC Symbol;Acc:HGNC:17367]	1754.5	2039.4	**1.16**	0.0003885
IMPA1	inositol monophosphatase 1 [Source:HGNC Symbol;Acc:HGNC:6050]	1435.4	1668.5	**1.16**	0.0003987
DNASE1L1	deoxyribonuclease 1 like 1 [Source:HGNC Symbol;Acc:HGNC:2957]	793.4	918.4	**1.16**	0.0004082
KCMF1	potassium channel modulatory factor 1 [Source:HGNC Symbol;Acc:HGNC:20589]	1779.6	2066.4	**1.16**	0.0005182
ACO1	aconitase 1 [Source:HGNC Symbol;Acc:HGNC:117]	1428.1	1662.7	**1.16**	0.0007382
NIPBL	NIPBL, cohesin loading factor [Source:HGNC Symbol;Acc:HGNC:28862]	1691.1	1962.0	**1.16**	0.0007973
VPS33A	VPS33A, CORVET/HOPS core subunit [Source:HGNC Symbol;Acc:HGNC:18179]	684.3	787.1	**1.16**	0.0008458
PDCD5	programmed cell death 5 [Source:HGNC Symbol;Acc:HGNC:8764]	1923.0	2250.6	**1.16**	0.0008676
CPNE8	copine 8 [Source:HGNC Symbol;Acc:HGNC:23498]	882.5	1018.9	**1.16**	0.00102
MAF1	MAF1 homolog, negative regulator of RNA polymerase III [Source:HGNC Symbol;Acc:HGNC:24966]	713.4	830.2	**1.16**	0.00108
AMMECR1L	AMMECR1 like [Source:HGNC Symbol;Acc:HGNC:28658]	621.2	720.0	**1.16**	0.001088
MDN1	midasin AAA ATPase 1 [Source:HGNC Symbol;Acc:HGNC:18302]	1921.5	2223.5	**1.16**	0.001232
ACSL1	acyl-CoA synthetase long chain family member 1 [Source:HGNC Symbol;Acc:HGNC:3569]	934.6	1087.4	**1.16**	0.001421
NCSTN	nicastrin [Source:HGNC Symbol;Acc:HGNC:17091]	959.7	1111.8	**1.16**	0.001556
FAM76B	family with sequence similarity 76 member B [Source:HGNC Symbol;Acc:HGNC:28492]	507.0	587.3	**1.16**	0.001569
ZNF398	zinc finger protein 398 [Source:HGNC Symbol;Acc:HGNC:18373]	439.9	509.3	**1.16**	0.001821
ZADH2	zinc binding alcohol dehydrogenase domain containing 2 [Source:HGNC Symbol;Acc:HGNC:28697]	591.4	683.6	**1.16**	0.001856
NDUFA1	NADH:ubiquinone oxidoreductase subunit A1 [Source:HGNC Symbol;Acc:HGNC:7683]	1086.5	1255.5	**1.16**	0.001899
BRAP	BRCA1 associated protein [Source:HGNC Symbol;Acc:HGNC:1099]	972.3	1122.7	**1.16**	0.00198
LAMTOR1	late endosomal/lysosomal adaptor, MAPK and MTOR activator 1 [Source:HGNC Symbol;Acc:HGNC:2606	681.9	787.0	**1.16**	0.00202
FBXO16	F-box protein 16 [Source:HGNC Symbol;Acc:HGNC:13618]	734.6	850.3	**1.16**	0.002283
SLC25A38	solute carrier family 25 member 38 [Source:HGNC Symbol;Acc:HGNC:26054]	648.1	748.0	**1.16**	0.003059
ZNF343	zinc finger protein 343 [Source:HGNC Symbol;Acc:HGNC:16017]	380.6	443.3	**1.16**	0.003137
BRI3	brain protein I3 [Source:HGNC Symbol;Acc:HGNC:1109]	772.7	897.8	**1.16**	0.003844
PHLPP2	PH domain and leucine rich repeat protein phosphatase 2 [Source:HGNC Symbol;Acc:HGNC:29149]	577.3	665.5	**1.16**	0.004343
CCDC73	coiled-coil domain containing 73 [Source:HGNC Symbol;Acc:HGNC:23261]	499.0	576.4	**1.16**	0.00451
TADA2B	transcriptional adaptor 2B [Source:HGNC Symbol;Acc:HGNC:30781]	414.9	483.3	**1.16**	0.004945
VIPAS39	VPS33B interacting protein, apical-basolateral polarity regulator, spe-39 homolog [Source:HGNC Symbol	432.3	496.7	**1.16**	0.004959
C2CD2	C2 calcium dependent domain containing 2 [Source:HGNC Symbol;Acc:HGNC:1266]	894.5	1032.4	**1.16**	0.005483
ZNF432	zinc finger protein 432 [Source:HGNC Symbol;Acc:HGNC:20810]	361.2	418.7	**1.16**	0.005897
ZMAT3	zinc finger matrin-type 3 [Source:HGNC Symbol;Acc:HGNC:29983]	675.2	790.7	**1.16**	0.006139
ACVR2A	activin A receptor type 2A [Source:HGNC Symbol;Acc:HGNC:173]	360.5	418.3	**1.16**	0.006791
TAB3	TGF-beta activated kinase 1 (MAP3K7) binding protein 3 [Source:HGNC Symbol;Acc:HGNC:30681]	513.1	595.1	**1.16**	0.007668
NDUFAF1	NADH:ubiquinone oxidoreductase complex assembly factor 1 [Source:HGNC Symbol;Acc:HGNC:18828]	589.3	674.5	**1.16**	0.008198
TNRC6C	trinucleotide repeat containing 6C [Source:HGNC Symbol;Acc:HGNC:29318]	600.8	695.5	**1.16**	0.009156
HTATIP2	HIV-1 Tat interactive protein 2 [Source:HGNC Symbol;Acc:HGNC:16637]	594.0	692.0	**1.16**	0.009157
MDC1	mediator of DNA damage checkpoint 1 [Source:HGNC Symbol;Acc:HGNC:21163]	378.5	439.7	**1.16**	0.009333
PID1	phosphotyrosine interaction domain containing 1 [Source:HGNC Symbol;Acc:HGNC:26084]	1618.9	1848.2	**1.16**	0.009591
SNORA25	small nucleolar RNA, H/ACA box 25 [Source:HGNC Symbol;Acc:HGNC:32615]	341.2	396.5	**1.16**	0.01597
ARSD	arylsulfatase D [Source:HGNC Symbol;Acc:HGNC:717]	317.7	368.2	**1.16**	0.01985
TXNDC16	thioredoxin domain containing 16 [Source:HGNC Symbol;Acc:HGNC:19965]	370.9	429.3	**1.16**	0.02139
NAXE	NAD(P)HX epimerase [Source:HGNC Symbol;Acc:HGNC:18453]	262.6	307.3	**1.16**	0.0214
PREX1	phosphatidylinositol-3,4,5-trisphosphate dependent Rac exchange factor 1 [Source:HGNC Symbol;Acc:H	1344.4	1565.2	**1.16**	0.02245
SEMA3F-AS1	SEMA3F antisense RNA 1 [Source:HGNC Symbol;Acc:HGNC:40518]	281.4	325.2	**1.16**	0.02421
CMTM7	CKLF like MARVEL transmembrane domain containing 7 [Source:HGNC Symbol;Acc:HGNC:19178]	370.1	429.3	**1.16**	0.02868
DMKN	dermokine [Source:HGNC Symbol;Acc:HGNC:25063]	346.5	404.0	**1.16**	0.03192
KIAA1147	KIAA1147 [Source:HGNC Symbol;Acc:HGNC:29472]	315.0	363.9	**1.16**	0.03234
UPF3B	UPF3B, regulator of nonsense mediated mRNA decay [Source:HGNC Symbol;Acc:HGNC:20439]	237.7	274.9	**1.16**	0.03254
TGIF2-RAB5IF	TGIF2-RAB5IF readthrough [Source:HGNC Symbol;Acc:HGNC:44664]	141.4	117.5	**1.16**	0.03557
RPS6KA6	ribosomal protein S6 kinase A6 [Source:HGNC Symbol;Acc:HGNC:10435]	187.2	215.7	**1.16**	0.04019
ULK4	unc-51 like kinase 4 [Source:HGNC Symbol;Acc:HGNC:15784]	228.0	261.8	**1.16**	0.04054
FASTKD3	FAST kinase domains 3 [Source:HGNC Symbol;Acc:HGNC:28758]	191.0	221.9	**1.16**	0.04159
TTLL12	tubulin tyrosine ligase like 12 [Source:HGNC Symbol;Acc:HGNC:28974]	248.6	289.5	**1.16**	0.04635
DDB1	damage specific DNA binding protein 1 [Source:HGNC Symbol;Acc:HGNC:2717]	5086.0	5837.1	**1.15**	1.63E-09
GDI2	GDP dissociation inhibitor 2 [Source:HGNC Symbol;Acc:HGNC:4227]	13189.1	15124.4	**1.15**	1.92E-07
ERP44	endoplasmic reticulum protein 44 [Source:HGNC Symbol;Acc:HGNC:18311]	2294.1	2644.6	**1.15**	3.35E-07
SMARCA5	SWI/SNF related, matrix associated, actin dependent regulator of chromatin, subfamily a, member 5 [Sc	3548.5	4074.4	**1.15**	6.04E-07
BTBD1	BTB domain containing 1 [Source:HGNC Symbol;Acc:HGNC:1120]	2225.6	2562.5	**1.15**	7.55E-07
CCDC93	coiled-coil domain containing 93 [Source:HGNC Symbol;Acc:HGNC:25611]	1471.4	1688.5	**1.15**	1.24E-06
ODR4	odr-4 GPCR localization factor homolog [Source:HGNC Symbol;Acc:HGNC:24299]	1495.1	1719.0	**1.15**	1.36E-06
CLTA	clathrin light chain A [Source:HGNC Symbol;Acc:HGNC:2090]	2446.3	2809.6	**1.15**	1.38E-06

RNF14	ring finger protein 14 [Source:HGNC Symbol;Acc:HGNC:10058]	2288.2	2635.9	**1.15**	1.40E-06
PNRC2	proline rich nuclear receptor coactivator 2 [Source:HGNC Symbol;Acc:HGNC:23158]	3676.7	4212.3	**1.15**	1.42E-06
MAPKAP1	mitogen-activated protein kinase associated protein 1 [Source:HGNC Symbol;Acc:HGNC:18752]	1686.4	1943.4	**1.15**	2.35E-06
MAN2A1	mannosidase alpha class 2A member 1 [Source:HGNC Symbol;Acc:HGNC:6824]	3712.8	4273.1	**1.15**	2.40E-06
ELF2	E74 like ETS transcription factor 2 [Source:HGNC Symbol;Acc:HGNC:3317]	2090.9	2412.2	**1.15**	2.59E-06
BMI1	BMI1 proto-oncogene, polycomb ring finger [Source:HGNC Symbol;Acc:HGNC:1066]	3269.6	3759.4	**1.15**	2.75E-06
FYTTD1	forty-two-three domain containing 1 [Source:HGNC Symbol;Acc:HGNC:25407]	3544.3	4050.7	**1.15**	5.46E-06
EEF2	eukaryotic translation elongation factor 2 [Source:HGNC Symbol;Acc:HGNC:3214]	19030.7	21979.8	**1.15**	5.68E-06
GCN1	GCN1, eIF2 alpha kinase activator homolog [Source:HGNC Symbol;Acc:HGNC:4199]	1998.6	2304.9	**1.15**	5.96E-06
ITGAV	integrin subunit alpha V [Source:HGNC Symbol;Acc:HGNC:6150]	11868.9	13524.0	**1.15**	6.75E-06
CPNE1	copine 1 [Source:HGNC Symbol;Acc:HGNC:2314]	1068.2	1225.4	**1.15**	8.74E-06
TLK2	tousled like kinase 2 [Source:HGNC Symbol;Acc:HGNC:11842]	1493.7	1716.9	**1.15**	9.03E-06
ANAPC16	anaphase promoting complex subunit 16 [Source:HGNC Symbol;Acc:HGNC:26976]	1427.8	1640.7	**1.15**	1.53E-05
ZNF655	zinc finger protein 655 [Source:HGNC Symbol;Acc:HGNC:30899]	1348.0	1553.1	**1.15**	1.75E-05
ZNF395	zinc finger protein 395 [Source:HGNC Symbol;Acc:HGNC:18737]	1721.5	1988.5	**1.15**	5.01E-05
FAM32A	family with sequence similarity 32 member A [Source:HGNC Symbol;Acc:HGNC:24563]	1099.8	1266.4	**1.15**	7.17E-05
ASH1L	ASH1 like histone lysine methyltransferase [Source:HGNC Symbol;Acc:HGNC:19088]	3399.5	3907.2	**1.15**	9.65E-05
ERO1A	endoplasmic reticulum oxidoreductase 1 alpha [Source:HGNC Symbol;Acc:HGNC:13280]	3779.2	4333.2	**1.15**	0.000102
ACBD5	acyl-CoA binding domain containing 5 [Source:HGNC Symbol;Acc:HGNC:23338]	1374.9	1583.1	**1.15**	0.0001219
UBE4B	ubiquitination factor E4B [Source:HGNC Symbol;Acc:HGNC:12500]	1184.9	1367.1	**1.15**	0.0001353
ZNF394	zinc finger protein 394 [Source:HGNC Symbol;Acc:HGNC:18832]	978.6	1133.6	**1.15**	0.000172
NOP14	NOP14 nucleolar protein [Source:HGNC Symbol;Acc:HGNC:16821]	1446.0	1670.4	**1.15**	0.0001766
CFAP36	cilia and flagella associated protein 36 [Source:HGNC Symbol;Acc:HGNC:30540]	1473.4	1701.2	**1.15**	0.00018
CFH	complement factor H [Source:HGNC Symbol;Acc:HGNC:4883]	32312.9	37219.0	**1.15**	0.0001886
HERC2	HECT and RLD domain containing E3 ubiquitin protein ligase 2 [Source:HGNC Symbol;Acc:HGNC:4868]	1888.9	2182.6	**1.15**	0.0003346
NT5C3B	5'-nucleotidase, cytosolic IIIB [Source:HGNC Symbol;Acc:HGNC:28300]	651.6	751.5	**1.15**	0.0003635
NDUFV3	NADH:ubiquinone oxidoreductase subunit V3 [Source:HGNC Symbol;Acc:HGNC:7719]	669.8	768.9	**1.15**	0.0003962
DNAJB1	DnaJ heat shock protein family (Hsp40) member B1 [Source:HGNC Symbol;Acc:HGNC:5270]	1195.5	1375.2	**1.15**	0.000416
TMEM168	transmembrane protein 168 [Source:HGNC Symbol;Acc:HGNC:25826]	714.7	824.9	**1.15**	0.0006595
POLG	DNA polymerase gamma, catalytic subunit [Source:HGNC Symbol;Acc:HGNC:9179]	698.1	799.7	**1.15**	0.0006678
CFB	complement factor B [Source:HGNC Symbol;Acc:HGNC:1037]	2508.5	2851.5	**1.15**	0.0007215
WBP11	WW domain binding protein 11 [Source:HGNC Symbol;Acc:HGNC:16461]	786.6	901.9	**1.15**	0.0007274
RPL18A	ribosomal protein L18a [Source:HGNC Symbol;Acc:HGNC:10311]	1194.3	1383.5	**1.15**	0.0007584
SNAP29	synaptosome associated protein 29 [Source:HGNC Symbol;Acc:HGNC:11133]	671.9	771.2	**1.15**	0.0009853
KMT2C	lysine methyltransferase 2C [Source:HGNC Symbol;Acc:HGNC:13726]	2089.2	2398.8	**1.15**	0.0009957
RRP1B	ribosomal RNA processing 1B [Source:HGNC Symbol;Acc:HGNC:23818]	871.1	997.7	**1.15**	0.001053
SETDB2	SET domain bifurcated 2 [Source:HGNC Symbol;Acc:HGNC:20263]	636.7	735.4	**1.15**	0.001124
HARS2	histidyl-tRNA synthetase 2, mitochondrial [Source:HGNC Symbol;Acc:HGNC:4817]	771.6	886.8	**1.15**	0.001157
CEP104	centrosomal protein 104 [Source:HGNC Symbol;Acc:HGNC:24866]	783.7	899.1	**1.15**	0.001387
POLR1A	RNA polymerase I subunit A [Source:HGNC Symbol;Acc:HGNC:17264]	950.0	1092.1	**1.15**	0.001412
RBBP5	RB binding protein 5, histone lysine methyltransferase complex subunit [Source:HGNC Symbol;Acc:HGN	891.0	1023.6	**1.15**	0.001418
SDHC	succinate dehydrogenase complex subunit C [Source:HGNC Symbol;Acc:HGNC:10682]	666.7	776.0	**1.15**	0.001754
ATP5MD	ATP synthase membrane subunit DAPIT [Source:HGNC Symbol;Acc:HGNC:30889]	1858.8	2140.8	**1.15**	0.00176
DAXX	death domain associated protein [Source:HGNC Symbol;Acc:HGNC:2681]	456.7	527.2	**1.15**	0.001889
NMT2	N-myristoyltransferase 2 [Source:HGNC Symbol;Acc:HGNC:7858]	639.1	736.3	**1.15**	0.002194
ENTPD5	ectonucleoside triphosphate diphosphohydrolase 5 [Source:HGNC Symbol;Acc:HGNC:3367]	784.4	898.1	**1.15**	0.002688
ZDHHC17	zinc finger DHHC-type containing 17 [Source:HGNC Symbol;Acc:HGNC:18412]	913.1	1042.8	**1.15**	0.002867
GTF2A2	general transcription factor IIA subunit 2 [Source:HGNC Symbol;Acc:HGNC:4647]	995.2	1142.6	**1.15**	0.003498
VCPIP1	valosin containing protein interacting protein 1 [Source:HGNC Symbol;Acc:HGNC:30897]	2479.6	2855.5	**1.15**	0.003837
RBBP6	RB binding protein 6, ubiquitin ligase [Source:HGNC Symbol;Acc:HGNC:9889]	861.6	994.2	**1.15**	0.004697
TET1	tet methylcytosine dioxygenase 1 [Source:HGNC Symbol;Acc:HGNC:29484]	935.3	1066.4	**1.15**	0.005052
TRIM24	tripartite motif containing 24 [Source:HGNC Symbol;Acc:HGNC:11812]	411.1	474.2	**1.15**	0.005104
BRWD3	bromodomain and WD repeat domain containing 3 [Source:HGNC Symbol;Acc:HGNC:17342]	750.0	864.0	**1.15**	0.005308
GIT2	GIT ArfGAP 2 [Source:HGNC Symbol;Acc:HGNC:4273]	609.2	700.2	**1.15**	0.005399
DDX20	DEAD-box helicase 20 [Source:HGNC Symbol;Acc:HGNC:2743]	654.9	756.8	**1.15**	0.006798
KLHL24	kelch like family member 24 [Source:HGNC Symbol;Acc:HGNC:25947]	1668.9	1955.0	**1.15**	0.007041
ZBTB24	zinc finger and BTB domain containing 24 [Source:HGNC Symbol;Acc:HGNC:21143]	479.2	552.6	**1.15**	0.007562
TSPO	translocator protein [Source:HGNC Symbol;Acc:HGNC:1158]	415.8	480.7	**1.15**	0.009931
KLHDC3	kelch domain containing 3 [Source:HGNC Symbol;Acc:HGNC:20704]	494.8	569.0	**1.15**	0.01115
ARID4A	AT-rich interaction domain 4A [Source:HGNC Symbol;Acc:HGNC:9885]	813.1	939.5	**1.15**	0.01482
MAPKAPK2	mitogen-activated protein kinase-activated protein kinase 2 [Source:HGNC Symbol;Acc:HGNC:6887]	810.9	940.9	**1.15**	0.01589
SLC10A3	solute carrier family 10 member 3 [Source:HGNC Symbol;Acc:HGNC:22979]	375.0	433.4	**1.15**	0.01666
ZNF91	zinc finger protein 91 [Source:HGNC Symbol;Acc:HGNC:13166]	548.6	631.2	**1.15**	0.01794
SBNO2	strawberry notch homolog 2 [Source:HGNC Symbol;Acc:HGNC:29158]	682.5	788.0	**1.15**	0.01896
AES	amino-terminal enhancer of split [Source:HGNC Symbol;Acc:HGNC:307]	568.1	655.5	**1.15**	0.01927
LY75-CD302	LY75-CD302 readthrough [Source:HGNC Symbol;Acc:HGNC:38828]	501.4	572.8	**1.15**	0.01981
TES	testin LIM domain protein [Source:HGNC Symbol;Acc:HGNC:14620]	2458.1	2812.9	**1.15**	0.0211
TRAPPC2	trafficking protein particle complex 2 [Source:HGNC Symbol;Acc:HGNC:23068]	298.1	343.3	**1.15**	0.0211
SIRT2	sirtuin 2 [Source:HGNC Symbol;Acc:HGNC:10886]	262.5	300.8	**1.15**	0.02443
INTS11	integrator complex subunit 11 [Source:HGNC Symbol;Acc:HGNC:26052]	362.1	418.1	**1.15**	0.02514
C2orf68	chromosome 2 open reading frame 68 [Source:HGNC Symbol;Acc:HGNC:34353]	413.1	474.6	**1.15**	0.02586
SSH1	slingshot protein phosphatase 1 [Source:HGNC Symbol;Acc:HGNC:30579]	1144.6	1315.8	**1.15**	0.0278

SYMPK	symplekin [Source:HGNC Symbol;Acc:HGNC:22935]	263.1	302.0	**1.15**	0.02833
RREB1	ras responsive element binding protein 1 [Source:HGNC Symbol;Acc:HGNC:10449]	704.0	809.9	**1.15**	0.02883
PHF11	PHD finger protein 11 [Source:HGNC Symbol;Acc:HGNC:17024]	570.4	655.2	**1.15**	0.02893
ANKRD44	ankyrin repeat domain 44 [Source:HGNC Symbol;Acc:HGNC:25259]	258.9	298.1	**1.15**	0.02992
JAZF1	JAZF zinc finger 1 [Source:HGNC Symbol;Acc:HGNC:28917]	371.5	422.7	**1.15**	0.03141
SH2D4A	SH2 domain containing 4A [Source:HGNC Symbol;Acc:HGNC:26102]	741.0	847.1	**1.15**	0.03206
ZNF101	zinc finger protein 101 [Source:HGNC Symbol;Acc:HGNC:12881]	252.2	289.4	**1.15**	0.0335
SCARNA2	small Cajal body-specific RNA 2 [Source:HGNC Symbol;Acc:HGNC:32558]	504.6	578.3	**1.15**	0.04628
UBA52	ubiquitin A-52 residue ribosomal protein fusion product 1 [Source:HGNC Symbol;Acc:HGNC:12458]	3373.6	3856.7	**1.14**	6.34E-10
SLC25A3	solute carrier family 25 member 3 [Source:HGNC Symbol;Acc:HGNC:10989]	5418.5	6211.3	**1.14**	9.92E-10
SEC31A	SEC31 homolog A, COPII coat complex component [Source:HGNC Symbol;Acc:HGNC:17052]	8670.9	9877.3	**1.14**	2.33E-08
DIS3	DIS3 homolog, exosome endoribonuclease and 3'-5' exoribonuclease [Source:HGNC Symbol;Acc:HGNC:2	2919.7	3339.9	**1.14**	5.58E-08
TOP2B	DNA topoisomerase II beta [Source:HGNC Symbol;Acc:HGNC:11990]	6787.2	7761.6	**1.14**	1.06E-07
SMU1	SMU1, DNA replication regulator and spliceosomal factor [Source:HGNC Symbol;Acc:HGNC:18247]	2797.7	3193.1	**1.14**	1.25E-07
CNBP	CCHC-type zinc finger nucleic acid binding protein [Source:HGNC Symbol;Acc:HGNC:13164]	11359.1	12965.4	**1.14**	1.53E-07
USP8	ubiquitin specific peptidase 8 [Source:HGNC Symbol;Acc:HGNC:12631]	2368.2	2707.7	**1.14**	1.63E-06
HK1	hexokinase 1 [Source:HGNC Symbol;Acc:HGNC:4922]	8979.6	10269.1	**1.14**	3.57E-06
RPS7	ribosomal protein S7 [Source:HGNC Symbol;Acc:HGNC:10440]	4647.5	5294.2	**1.14**	4.68E-06
PAFAH1B2	platelet activating factor acetylhydrolase 1b catalytic subunit 2 [Source:HGNC Symbol;Acc:HGNC:8575]	4518.2	5135.2	**1.14**	6.07E-06
IK	IK cytokine [Source:HGNC Symbol;Acc:HGNC:5958]	3465.7	3938.6	**1.14**	7.78E-06
KDM5A	lysine demethylase 5A [Source:HGNC Symbol;Acc:HGNC:9886]	2317.9	2639.6	**1.14**	1.01E-05
UBAP2	ubiquitin associated protein 2 [Source:HGNC Symbol;Acc:HGNC:14185]	1736.2	1977.0	**1.14**	1.11E-05
SNX14	sorting nexin 14 [Source:HGNC Symbol;Acc:HGNC:14977]	2746.1	3142.9	**1.14**	1.16E-05
GOLPH3	golgi phosphoprotein 3 [Source:HGNC Symbol;Acc:HGNC:15452]	3535.2	4051.0	**1.14**	1.27E-05
C1S	complement C1s [Source:HGNC Symbol;Acc:HGNC:1247]	12408.7	14094.8	**1.14**	2.00E-05
SEM1	SEM1, 26S proteasome complex subunit [Source:HGNC Symbol;Acc:HGNC:10845]	2191.2	2499.8	**1.14**	2.39E-05
RPLP0	ribosomal protein lateral stalk subunit P0 [Source:HGNC Symbol;Acc:HGNC:10371]	15885.4	18158.9	**1.14**	2.50E-05
CD68	CD68 molecule [Source:HGNC Symbol;Acc:HGNC:1693]	5118.0	5852.7	**1.14**	2.61E-05
PROS1	protein S [Source:HGNC Symbol;Acc:HGNC:9456]	1449.5	1645.1	**1.14**	3.03E-05
RPL14	ribosomal protein L14 [Source:HGNC Symbol;Acc:HGNC:10305]	7979.7	9099.9	**1.14**	3.33E-05
RSL1D1	ribosomal L1 domain containing 1 [Source:HGNC Symbol;Acc:HGNC:24534]	3089.0	3538.1	**1.14**	4.50E-05
ATG12	autophagy related 12 [Source:HGNC Symbol;Acc:HGNC:588]	2328.7	2666.9	**1.14**	4.69E-05
CPSF6	cleavage and polyadenylation specific factor 6 [Source:HGNC Symbol;Acc:HGNC:13871]	1286.4	1466.2	**1.14**	6.21E-05
TRIM33	tripartite motif containing 33 [Source:HGNC Symbol;Acc:HGNC:16290]	1215.2	1387.0	**1.14**	0.0001306
FBXW7	F-box and WD repeat domain containing 7 [Source:HGNC Symbol;Acc:HGNC:16712]	1651.2	1885.1	**1.14**	0.0001334
KLHL28	kelch like family member 28 [Source:HGNC Symbol;Acc:HGNC:19741]	991.1	1128.8	**1.14**	0.0001485
TOX4	TOX high mobility group box family member 4 [Source:HGNC Symbol;Acc:HGNC:20161]	1332.5	1524.4	**1.14**	0.0001651
AP3M1	adaptor related protein complex 3 subunit mu 1 [Source:HGNC Symbol;Acc:HGNC:569]	1205.1	1378.0	**1.14**	0.0001853
RNPS1	RNA binding protein with serine rich domain 1 [Source:HGNC Symbol;Acc:HGNC:10080]	893.8	1024.7	**1.14**	0.0001925
METTL14	methyltransferase like 14 [Source:HGNC Symbol;Acc:HGNC:29330]	1045.5	1190.9	**1.14**	0.0001933
CLK1	CDC like kinase 1 [Source:HGNC Symbol;Acc:HGNC:2068]	3121.7	3560.4	**1.14**	0.0002025
N4BP1	NEDD4 binding protein 1 [Source:HGNC Symbol;Acc:HGNC:29850]	963.0	1100.5	**1.14**	0.0002272
PPP4R1	protein phosphatase 4 regulatory subunit 1 [Source:HGNC Symbol;Acc:HGNC:9320]	2561.6	2926.5	**1.14**	0.0002418
POGK	pogo transposable element derived with KRAB domain [Source:HGNC Symbol;Acc:HGNC:18800]	1010.2	1157.2	**1.14**	0.0002456
IFNGR2	interferon gamma receptor 2 [Source:HGNC Symbol;Acc:HGNC:5440]	1238.5	1418.3	**1.14**	0.0002586
ARMCX1	armadillo repeat containing X-linked 1 [Source:HGNC Symbol;Acc:HGNC:18073]	2012.6	2304.4	**1.14**	0.000312
SNRK	SNF related kinase [Source:HGNC Symbol;Acc:HGNC:30598]	680.3	776.9	**1.14**	0.000313
ODF2	outer dense fiber of sperm tails 2 [Source:HGNC Symbol;Acc:HGNC:8114]	1173.9	1341.4	**1.14**	0.0004073
MTCH1	mitochondrial carrier 1 [Source:HGNC Symbol;Acc:HGNC:17586]	1189.7	1358.0	**1.14**	0.0004243
PRPF6	pre-mRNA processing factor 6 [Source:HGNC Symbol;Acc:HGNC:15860]	1612.9	1841.7	**1.14**	0.0004636
SELENOK	selenoprotein K [Source:HGNC Symbol;Acc:HGNC:30394]	1083.6	1237.8	**1.14**	0.0004702
ERAP1	endoplasmic reticulum aminopeptidase 1 [Source:HGNC Symbol;Acc:HGNC:18173]	1175.6	1349.5	**1.14**	0.0006662
EIF3M	eukaryotic translation initiation factor 3 subunit M [Source:HGNC Symbol;Acc:HGNC:24460]	2209.9	2523.6	**1.14**	0.0007293
GOLGB1	golgin B1 [Source:HGNC Symbol;Acc:HGNC:4429]	9063.7	10368.9	**1.14**	0.0007637
MTMR9	myotubularin related protein 9 [Source:HGNC Symbol;Acc:HGNC:14596]	939.7	1071.8	**1.14**	0.0007709
NEK1	NIMA related kinase 1 [Source:HGNC Symbol;Acc:HGNC:7744]	1458.8	1666.4	**1.14**	0.0008513
ACTB	actin beta [Source:HGNC Symbol;Acc:HGNC:132]	15670.5	17926.9	**1.14**	0.001259
UIMC1	ubiquitin interaction motif containing 1 [Source:HGNC Symbol;Acc:HGNC:30298]	833.3	952.9	**1.14**	0.001269
PDE12	phosphodiesterase 12 [Source:HGNC Symbol;Acc:HGNC:25386]	847.8	971.2	**1.14**	0.001375
FNBP4	formin binding protein 4 [Source:HGNC Symbol;Acc:HGNC:19752]	1068.0	1225.8	**1.14**	0.0018
SP100	SP100 nuclear antigen [Source:HGNC Symbol;Acc:HGNC:11206]	1672.3	1921.5	**1.14**	0.001899
STAT5B	signal transducer and activator of transcription 5B [Source:HGNC Symbol;Acc:HGNC:11367]	873.0	997.9	**1.14**	0.00199
RNF139	ring finger protein 139 [Source:HGNC Symbol;Acc:HGNC:17023]	1009.3	1156.8	**1.14**	0.002035
DDI2	DNA damage inducible 1 homolog 2 [Source:HGNC Symbol;Acc:HGNC:24578]	1022.9	1161.5	**1.14**	0.002194
NCOA2	nuclear receptor coactivator 2 [Source:HGNC Symbol;Acc:HGNC:7669]	1072.8	1225.2	**1.14**	0.002481
MAGI2-AS3	MAGI2 antisense RNA 3 [Source:HGNC Symbol;Acc:HGNC:40862]	1965.9	2247.0	**1.14**	0.002668
NPHP3	nephrocystin 3 [Source:HGNC Symbol;Acc:HGNC:7907]	869.3	998.4	**1.14**	0.003313
GATAD1	GATA zinc finger domain containing 1 [Source:HGNC Symbol;Acc:HGNC:29941]	727.9	829.2	**1.14**	0.003528
NFE2L2	nuclear factor, erythroid 2 like 2 [Source:HGNC Symbol;Acc:HGNC:7782]	1086.6	1235.5	**1.14**	0.004596
MAP2K1	mitogen-activated protein kinase kinase 1 [Source:HGNC Symbol;Acc:HGNC:6840]	1100.0	1263.7	**1.14**	0.004821
EEF1A1P5	eukaryotic translation elongation factor 1 alpha 1 pseudogene 5 [Source:HGNC Symbol;Acc:HGNC:3200]	1004.0	1145.9	**1.14**	0.006072
RPL28	ribosomal protein L28 [Source:HGNC Symbol;Acc:HGNC:10330]	678.5	773.9	**1.14**	0.006767

FAM160B1	family with sequence similarity 160 member B1 [Source:HGNC Symbol;Acc:HGNC:29320]	981.8	1120.5	**1.14**	0.006865
KIAA1468	KIAA1468 [Source:HGNC Symbol;Acc:HGNC:29289]	822.1	936.1	**1.14**	0.00766
HABP4	hyaluronan binding protein 4 [Source:HGNC Symbol;Acc:HGNC:17062]	1313.4	1505.8	**1.14**	0.009079
PAFAH2	platelet activating factor acetylhydrolase 2 [Source:HGNC Symbol;Acc:HGNC:8579]	269.6	308.3	**1.14**	0.01299
TDRD7	tudor domain containing 7 [Source:HGNC Symbol;Acc:HGNC:30831]	449.3	514.7	**1.14**	0.01337
EIF4E3	eukaryotic translation initiation factor 4E family member 3 [Source:HGNC Symbol;Acc:HGNC:31837]	462.6	530.0	**1.14**	0.01431
STAU2	staufen double-stranded RNA binding protein 2 [Source:HGNC Symbol;Acc:HGNC:11371]	457.5	525.2	**1.14**	0.01503
TATDN2	TatD DNase domain containing 2 [Source:HGNC Symbol;Acc:HGNC:28988]	494.5	565.6	**1.14**	0.01512
PSMG2	proteasome assembly chaperone 2 [Source:HGNC Symbol;Acc:HGNC:24929]	484.5	555.3	**1.14**	0.01672
ZNF44	zinc finger protein 44 [Source:HGNC Symbol;Acc:HGNC:13110]	388.5	445.1	**1.14**	0.01691
WDR5	WD repeat domain 5 [Source:HGNC Symbol;Acc:HGNC:12757]	427.0	488.3	**1.14**	0.0176
MTFR1L	mitochondrial fission regulator 1 like [Source:HGNC Symbol;Acc:HGNC:28836]	538.7	615.0	**1.14**	0.01941
ZNF557	zinc finger protein 557 [Source:HGNC Symbol;Acc:HGNC:28632]	433.4	495.8	**1.14**	0.01971
GBF1	golgi brefeldin A resistant guanine nucleotide exchange factor 1 [Source:HGNC Symbol;Acc:HGNC:4181]	1455.0	1662.6	**1.14**	0.01985
RUNDC1	RUN domain containing 1 [Source:HGNC Symbol;Acc:HGNC:25418]	415.1	474.5	**1.14**	0.01985
ZFYVE26	zinc finger FYVE-type containing 26 [Source:HGNC Symbol;Acc:HGNC:20761]	445.3	511.8	**1.14**	0.02228
CLK4	CDC like kinase 4 [Source:HGNC Symbol;Acc:HGNC:13659]	461.4	528.8	**1.14**	0.02569
GCC1	GRIP and coiled-coil domain containing 1 [Source:HGNC Symbol;Acc:HGNC:19095]	745.2	847.5	**1.14**	0.03383
AVEN	apoptosis and caspase activation inhibitor [Source:HGNC Symbol;Acc:HGNC:13509]	418.3	478.5	**1.14**	0.03541
NAPA	NSF attachment protein alpha [Source:HGNC Symbol;Acc:HGNC:7641]	580.6	662.3	**1.14**	0.04225
ZBTB5	zinc finger and BTB domain containing 5 [Source:HGNC Symbol;Acc:HGNC:23836]	262.0	299.2	**1.14**	0.04242
HDAC5	histone deacetylase 5 [Source:HGNC Symbol;Acc:HGNC:14068]	602.7	693.1	**1.14**	0.04295
STYXL1	serine/threonine/tyrosine interacting like 1 [Source:HGNC Symbol;Acc:HGNC:18165]	248.9	283.3	**1.14**	0.04344
HNRNPH2	heterogeneous nuclear ribonucleoprotein H2 [Source:HGNC Symbol;Acc:HGNC:5042]	5176.9	5881.1	**1.13**	2.15E-09
HNRNPU	heterogeneous nuclear ribonucleoprotein U [Source:HGNC Symbol;Acc:HGNC:5048]	16173.0	18301.7	**1.13**	5.75E-08
DYRK1A	dual specificity tyrosine phosphorylation regulated kinase 1A [Source:HGNC Symbol;Acc:HGNC:3091]	2569.0	2919.4	**1.13**	3.93E-07
COMMD3-BMI1	COMMD3-BMI1 readthrough [Source:HGNC Symbol;Acc:HGNC:48326]	2706.0	3064.0	**1.13**	6.56E-06
RPL4	ribosomal protein L4 [Source:HGNC Symbol;Acc:HGNC:10353]	18447.9	20945.9	**1.13**	1.42E-05
PCOLCE2	procollagen C-endopeptidase enhancer 2 [Source:HGNC Symbol;Acc:HGNC:8739]	24950.9	28278.0	**1.13**	1.54E-05
TMCO1	transmembrane and coiled-coil domains 1 [Source:HGNC Symbol;Acc:HGNC:18188]	5325.4	6028.8	**1.13**	2.93E-05
DNTTIP2	deoxynucleotidyltransferase terminal interacting protein 2 [Source:HGNC Symbol;Acc:HGNC:24013]	2797.2	3161.7	**1.13**	3.06E-05
RPS8	ribosomal protein S8 [Source:HGNC Symbol;Acc:HGNC:10441]	9655.3	10944.5	**1.13**	3.11E-05
RPL27	ribosomal protein L27 [Source:HGNC Symbol;Acc:HGNC:10328]	5796.1	6621.3	**1.13**	3.16E-05
PRKAR2A	protein kinase cAMP-dependent type II regulatory subunit alpha [Source:HGNC Symbol;Acc:HGNC:9391	1865.4	2112.1	**1.13**	3.43E-05
RPL24	ribosomal protein L24 [Source:HGNC Symbol;Acc:HGNC:10325]	11221.9	12719.9	**1.13**	4.52E-05
MTIF2	mitochondrial translational initiation factor 2 [Source:HGNC Symbol;Acc:HGNC:7441]	1448.5	1635.3	**1.13**	4.57E-05
ANKRD13A	ankyrin repeat domain 13A [Source:HGNC Symbol;Acc:HGNC:21268]	1071.0	1216.4	**1.13**	5.55E-05
UBR1	ubiquitin protein ligase E3 component n-recognin 1 [Source:HGNC Symbol;Acc:HGNC:16808]	2433.7	2754.5	**1.13**	6.27E-05
FAM114A2	family with sequence similarity 114 member A2 [Source:HGNC Symbol;Acc:HGNC:1333]	1233.2	1396.2	**1.13**	0.0001059
RNF19A	ring finger protein 19A, RBR E3 ubiquitin protein ligase [Source:HGNC Symbol;Acc:HGNC:13432]	1601.6	1823.7	**1.13**	0.0001148
ANXA11	annexin A11 [Source:HGNC Symbol;Acc:HGNC:535]	2075.3	2364.4	**1.13**	0.0001265
BAG5	BCL2 associated athanogene 5 [Source:HGNC Symbol;Acc:HGNC:941]	2466.9	2789.5	**1.13**	0.0001954
INTS6	integrator complex subunit 6 [Source:HGNC Symbol;Acc:HGNC:14879]	1591.1	1798.0	**1.13**	0.0002376
ZFYVE9	zinc finger FYVE-type containing 9 [Source:HGNC Symbol;Acc:HGNC:6775]	1553.5	1765.1	**1.13**	0.0002686
C5orf24	chromosome 5 open reading frame 24 [Source:HGNC Symbol;Acc:HGNC:26746]	2089.9	2367.4	**1.13**	0.0002768
SEC24C	SEC24 homolog C, COPII coat complex component [Source:HGNC Symbol;Acc:HGNC:10705]	1745.2	1982.7	**1.13**	0.0002844
RPL37A	ribosomal protein L37a [Source:HGNC Symbol;Acc:HGNC:10348]	6995.1	7925.8	**1.13**	0.000319
HOOK3	hook microtubule tethering protein 3 [Source:HGNC Symbol;Acc:HGNC:23576]	3298.4	3751.9	**1.13**	0.0003358
NIFK	nucleolar protein interacting with the FHA domain of MKI67 [Source:HGNC Symbol;Acc:HGNC:17838]	1644.4	1865.5	**1.13**	0.0004067
KPNA6	karyopherin subunit alpha 6 [Source:HGNC Symbol;Acc:HGNC:6399]	1883.2	2130.2	**1.13**	0.0004264
PCMTD1	protein-L-isoaspartate (D-aspartate) O-methyltransferase domain containing 1 [Source:HGNC Symbol;A	2147.1	2441.9	**1.13**	0.0004653
TAF9	TATA-box binding protein associated factor 9 [Source:HGNC Symbol;Acc:HGNC:11542]	1154.2	1310.4	**1.13**	0.0004735
SRSF4	serine and arginine rich splicing factor 4 [Source:HGNC Symbol;Acc:HGNC:10786]	1476.9	1670.3	**1.13**	0.0004736
MRPS5	mitochondrial ribosomal protein S5 [Source:HGNC Symbol;Acc:HGNC:14498]	1016.0	1151.6	**1.13**	0.0005266
DHX38	DEAH-box helicase 38 [Source:HGNC Symbol;Acc:HGNC:17211]	1322.4	1500.7	**1.13**	0.0006109
DDX18	DEAD-box helicase 18 [Source:HGNC Symbol;Acc:HGNC:2741]	2682.8	3033.0	**1.13**	0.0006364
FBXO7	F-box protein 7 [Source:HGNC Symbol;Acc:HGNC:13586]	1162.9	1322.9	**1.13**	0.0006508
SEC24B	SEC24 homolog B, COPII coat complex component [Source:HGNC Symbol;Acc:HGNC:10704]	1145.4	1299.1	**1.13**	0.0007482
RBM4	RNA binding motif protein 4 [Source:HGNC Symbol;Acc:HGNC:9901]	944.8	1070.1	**1.13**	0.0008063
TRAPPC4	trafficking protein particle complex 4 [Source:HGNC Symbol;Acc:HGNC:19943]	639.4	725.1	**1.13**	0.0009032
PRMT5	protein arginine methyltransferase 5 [Source:HGNC Symbol;Acc:HGNC:10894]	1098.9	1243.2	**1.13**	0.0009573
SSFA2	sperm specific antigen 2 [Source:HGNC Symbol;Acc:HGNC:11319]	3815.9	4308.7	**1.13**	0.0009582
CFDP1	craniofacial development protein 1 [Source:HGNC Symbol;Acc:HGNC:1873]	1395.8	1588.8	**1.13**	0.001006
ATP11C	ATPase phospholipid transporting 11C [Source:HGNC Symbol;Acc:HGNC:13554]	827.8	935.7	**1.13**	0.001341
HEATR1	HEAT repeat containing 1 [Source:HGNC Symbol;Acc:HGNC:25517]	2319.7	2640.2	**1.13**	0.001536
RAP1GDS1	Rap1 GTPase-GDP dissociation stimulator 1 [Source:HGNC Symbol;Acc:HGNC:9859]	1415.6	1601.8	**1.13**	0.001564
PLSCR1	phospholipid scramblase 1 [Source:HGNC Symbol;Acc:HGNC:9092]	708.0	806.3	**1.13**	0.001857
PPP2R3C	protein phosphatase 2 regulatory subunit B''gamma [Source:HGNC Symbol;Acc:HGNC:17485]	760.0	862.2	**1.13**	0.001978
WDR1	WD repeat domain 1 [Source:HGNC Symbol;Acc:HGNC:12754]	4101.8	4675.6	**1.13**	0.001995
DMXL2	Dmx like 2 [Source:HGNC Symbol;Acc:HGNC:2938]	1267.9	1436.1	**1.13**	0.002323
C1orf216	chromosome 1 open reading frame 216 [Source:HGNC Symbol;Acc:HGNC:26800]	611.3	691.7	**1.13**	0.00261
BICD2	BICD cargo adaptor 2 [Source:HGNC Symbol;Acc:HGNC:17208]	759.1	858.4	**1.13**	0.00272

PCID2	PCI domain containing 2 [Source:HGNC Symbol;Acc:HGNC:25653]	784.6	888.3	**1.13**	0.003088
HBP1	HMG-box transcription factor 1 [Source:HGNC Symbol;Acc:HGNC:23200]	2267.8	2609.3	**1.13**	0.003353
TNKS2	tankyrase 2 [Source:HGNC Symbol;Acc:HGNC:15677]	2335.7	2679.8	**1.13**	0.00366
VPS36	vacuolar protein sorting 36 homolog [Source:HGNC Symbol;Acc:HGNC:20312]	936.4	1061.6	**1.13**	0.003918
SECISBP2	SECIS binding protein 2 [Source:HGNC Symbol;Acc:HGNC:30972]	841.6	949.7	**1.13**	0.00451
PAN2	poly(A) specific ribonuclease subunit PAN2 [Source:HGNC Symbol;Acc:HGNC:20074]	477.4	545.1	**1.13**	0.004555
AHSA2P	activator of HSP90 ATPase homolog 2, pseudogene [Source:HGNC Symbol;Acc:HGNC:20437]	738.4	838.2	**1.13**	0.004874
C1GALT1C1	C1GALT1 specific chaperone 1 [Source:HGNC Symbol;Acc:HGNC:24338]	1226.3	1395.0	**1.13**	0.004928
ZKSCAN1	zinc finger with KRAB and SCAN domains 1 [Source:HGNC Symbol;Acc:HGNC:13101]	2776.9	3158.8	**1.13**	0.006284
LZTFL1	leucine zipper transcription factor like 1 [Source:HGNC Symbol;Acc:HGNC:6741]	763.0	866.1	**1.13**	0.006753
USP28	ubiquitin specific peptidase 28 [Source:HGNC Symbol;Acc:HGNC:12625]	488.8	555.7	**1.13**	0.006882
ALDH2	aldehyde dehydrogenase 2 family member [Source:HGNC Symbol;Acc:HGNC:404]	984.3	1128.8	**1.13**	0.007366
FAM161B	FAM161B, centrosomal protein [Source:HGNC Symbol;Acc:HGNC:19854]	650.2	738.6	**1.13**	0.008302
TRIP10	thyroid hormone receptor interactor 10 [Source:HGNC Symbol;Acc:HGNC:12304]	586.8	665.3	**1.13**	0.009257
CRELD2	cysteine rich with EGF like domains 2 [Source:HGNC Symbol;Acc:HGNC:28150]	1347.1	1533.2	**1.13**	0.01116
UBE2F-SCLY	UBE2F-SCLY readthrough (NMD candidate) [Source:HGNC Symbol;Acc:HGNC:48339]	373.8	423.8	**1.13**	0.01255
KLF13	Kruppel like factor 13 [Source:HGNC Symbol;Acc:HGNC:13672]	1173.2	1329.9	**1.13**	0.01723
C12orf76	chromosome 12 open reading frame 76 [Source:HGNC Symbol;Acc:HGNC:33790]	372.6	424.2	**1.13**	0.01746
TOLLIP	toll interacting protein [Source:HGNC Symbol;Acc:HGNC:16476]	461.8	523.4	**1.13**	0.01747
LSS	lanosterol synthase [Source:HGNC Symbol;Acc:HGNC:6708]	911.3	1029.8	**1.13**	0.01812
ERVK3-1	endogenous retrovirus group K3 member 1 [Source:HGNC Symbol;Acc:HGNC:30466]	412.8	469.4	**1.13**	0.01855
BRD9	bromodomain containing 9 [Source:HGNC Symbol;Acc:HGNC:25818]	586.4	666.7	**1.13**	0.01864
METTL3	methyltransferase like 3 [Source:HGNC Symbol;Acc:HGNC:17563]	663.0	751.7	**1.13**	0.01975
MID2	midline 2 [Source:HGNC Symbol;Acc:HGNC:7096]	381.3	435.4	**1.13**	0.01979
SLC5A6	solute carrier family 5 member 6 [Source:HGNC Symbol;Acc:HGNC:11041]	465.9	528.3	**1.13**	0.02002
EP300	E1A binding protein p300 [Source:HGNC Symbol;Acc:HGNC:3373]	731.7	830.1	**1.13**	0.0201
ZNF654	zinc finger protein 654 [Source:HGNC Symbol;Acc:HGNC:25612]	892.6	1018.4	**1.13**	0.02197
COA7	cytochrome c oxidase assembly factor 7 (putative) [Source:HGNC Symbol;Acc:HGNC:25716]	508.0	579.4	**1.13**	0.02262
PIK3R4	phosphoinositide-3-kinase regulatory subunit 4 [Source:HGNC Symbol;Acc:HGNC:8982]	764.5	868.7	**1.13**	0.02437
TGFBRAP1	transforming growth factor beta receptor associated protein 1 [Source:HGNC Symbol;Acc:HGNC:16836]	392.7	446.3	**1.13**	0.02466
NAT9	N-acetyltransferase 9 (putative) [Source:HGNC Symbol;Acc:HGNC:23133]	472.6	534.3	**1.13**	0.02507
BVES	blood vessel epicardial substance [Source:HGNC Symbol;Acc:HGNC:1152]	521.9	591.1	**1.13**	0.02611
SLC25A36	solute carrier family 25 member 36 [Source:HGNC Symbol;Acc:HGNC:25554]	1475.6	1691.3	**1.13**	0.02623
C3orf38	chromosome 3 open reading frame 38 [Source:HGNC Symbol;Acc:HGNC:28384]	661.6	747.8	**1.13**	0.02727
ZZEF1	zinc finger ZZ-type and EF-hand domain containing 1 [Source:HGNC Symbol;Acc:HGNC:29027]	862.5	975.8	**1.13**	0.02929
KMT2A	lysine methyltransferase 2A [Source:HGNC Symbol;Acc:HGNC:7132]	1156.5	1318.0	**1.13**	0.02954
HAUS3	HAUS augmin like complex subunit 3 [Source:HGNC Symbol;Acc:HGNC:28719]	540.9	613.8	**1.13**	0.03331
ATG14	autophagy related 14 [Source:HGNC Symbol;Acc:HGNC:19962]	462.6	522.2	**1.13**	0.03579
ZNF721	zinc finger protein 721 [Source:HGNC Symbol;Acc:HGNC:29425]	386.4	440.0	**1.13**	0.0359
MUS81	MUS81 structure-specific endonuclease subunit [Source:HGNC Symbol;Acc:HGNC:29814]	317.3	359.3	**1.13**	0.03815
SLC10A7	solute carrier family 10 member 7 [Source:HGNC Symbol;Acc:HGNC:23088]	350.6	400.5	**1.13**	0.03845
JADE2	jade family PHD finger 2 [Source:HGNC Symbol;Acc:HGNC:22984]	548.2	627.3	**1.13**	0.04034
KLHL18	kelch like family member 18 [Source:HGNC Symbol;Acc:HGNC:29120]	392.4	447.2	**1.13**	0.04188
ZHX1-C8orf76	ZHX1-C8orf76 readthrough [Source:HGNC Symbol;Acc:HGNC:42975]	257.8	293.2	**1.13**	0.0445
PRKAA1	protein kinase AMP-activated catalytic subunit alpha 1 [Source:HGNC Symbol;Acc:HGNC:9376]	3142.1	3540.6	**1.13**	3.33E-06
WDR82	WD repeat domain 82 [Source:HGNC Symbol;Acc:HGNC:28826]	2601.8	2921.6	**1.13**	8.58E-06
ARPP19	cAMP regulated phosphoprotein 19 [Source:HGNC Symbol;Acc:HGNC:16967]	4797.6	5401.4	**1.13**	1.72E-05
TNPO3	transportin 3 [Source:HGNC Symbol;Acc:HGNC:17103]	1955.7	2197.3	**1.13**	1.78E-05
EIF2S3	eukaryotic translation initiation factor 2 subunit gamma [Source:HGNC Symbol;Acc:HGNC:3267]	5794.9	6544.1	**1.13**	2.06E-05
XRN1	5'-3' exoribonuclease 1 [Source:HGNC Symbol;Acc:HGNC:30654]	2569.5	2902.7	**1.13**	2.35E-05
RPS15A	ribosomal protein S15a [Source:HGNC Symbol;Acc:HGNC:10389]	8830.0	9931.0	**1.13**	2.50E-05
SETX	senataxin [Source:HGNC Symbol;Acc:HGNC:445]	4593.8	5179.7	**1.13**	3.07E-05
ITPRIPL2	ITPRIP like 2 [Source:HGNC Symbol;Acc:HGNC:27257]	3972.6	4488.1	**1.13**	3.36E-05
GNG12	G protein subunit gamma 12 [Source:HGNC Symbol;Acc:HGNC:19663]	5453.2	6132.1	**1.13**	4.52E-05
BRWD1	bromodomain and WD repeat domain containing 1 [Source:HGNC Symbol;Acc:HGNC:12760]	2806.6	3171.9	**1.13**	4.65E-05
LAMP2	lysosomal associated membrane protein 2 [Source:HGNC Symbol;Acc:HGNC:6501]	5664.9	6371.5	**1.13**	4.73E-05
SMAD2	SMAD family member 2 [Source:HGNC Symbol;Acc:HGNC:6768]	3746.0	4201.7	**1.13**	4.87E-05
DCTN4	dynactin subunit 4 [Source:HGNC Symbol;Acc:HGNC:15518]	2146.2	2407.3	**1.13**	6.63E-05
SERINC1	serine incorporator 1 [Source:HGNC Symbol;Acc:HGNC:13464]	20371.5	22936.2	**1.13**	6.91E-05
TMF1	TATA element modulatory factor 1 [Source:HGNC Symbol;Acc:HGNC:11870]	5350.6	6010.4	**1.13**	7.06E-05
CFAP97	cilia and flagella associated protein 97 [Source:HGNC Symbol;Acc:HGNC:29276]	3126.9	3528.8	**1.13**	7.26E-05
TACC1	transforming acidic coiled-coil containing protein 1 [Source:HGNC Symbol;Acc:HGNC:11522]	3366.0	3801.4	**1.13**	7.57E-05
DNAJC14	DnaJ heat shock protein family (Hsp40) member C14 [Source:HGNC Symbol;Acc:HGNC:24581]	1212.7	1366.7	**1.13**	0.000141
RPS3A	ribosomal protein S3A [Source:HGNC Symbol;Acc:HGNC:10421]	24854.4	27990.2	**1.13**	0.0001558
DDX3X	DEAD-box helicase 3 X-linked [Source:HGNC Symbol;Acc:HGNC:2745]	13064.3	14596.3	**1.13**	0.0001701
SCAF11	SR-related CTD associated factor 11 [Source:HGNC Symbol;Acc:HGNC:10784]	3688.0	4158.7	**1.13**	0.0001798
CCDC7	coiled-coil domain containing 7 [Source:HGNC Symbol;Acc:HGNC:26533]	1516.2	1719.1	**1.13**	0.0002274
UPF1	UPF1, RNA helicase and ATPase [Source:HGNC Symbol;Acc:HGNC:9962]	1072.2	1209.3	**1.13**	0.0002497
SELENOS	selenoprotein S [Source:HGNC Symbol;Acc:HGNC:30396]	3007.1	3403.5	**1.13**	0.0002739
PIK3C3	phosphatidylinositol 3-kinase catalytic subunit type 3 [Source:HGNC Symbol;Acc:HGNC:8974]	1720.2	1937.0	**1.13**	0.0003278
MED13	mediator complex subunit 13 [Source:HGNC Symbol;Acc:HGNC:22474]	2800.0	3148.2	**1.13**	0.0003581
MGA	MGA, MAX dimerization protein [Source:HGNC Symbol;Acc:HGNC:14010]	5112.3	5757.0	**1.13**	0.0003921

CNN3	calponin 3 [Source:HGNC Symbol;Acc:HGNC:2157]	5854.9	6613.0	**1.13**	0.0004044
CYP20A1	cytochrome P450 family 20 subfamily A member 1 [Source:HGNC Symbol;Acc:HGNC:20576]	2697.2	3030.7	**1.13**	0.0006278
SNX6	sorting nexin 6 [Source:HGNC Symbol;Acc:HGNC:14970]	2543.1	2872.2	**1.13**	0.0006286
SETD2	SET domain containing 2 [Source:HGNC Symbol;Acc:HGNC:18420]	2035.2	2296.7	**1.13**	0.0007747
RPS5	ribosomal protein S5 [Source:HGNC Symbol;Acc:HGNC:10426]	3047.3	3438.5	**1.13**	0.001009
TROVE2	TROVE domain family member 2 [Source:HGNC Symbol;Acc:HGNC:11313]	6029.7	6808.6	**1.13**	0.001168
MUC20-OT1	MUC20 overlapping transcript [Source:HGNC Symbol;Acc:HGNC:53807]	1029.2	1155.6	**1.13**	0.001174
NUP133	nucleoporin 133 [Source:HGNC Symbol;Acc:HGNC:18016]	1381.0	1557.5	**1.13**	0.00132
FGFR1OP2	FGFR1 oncogene partner 2 [Source:HGNC Symbol;Acc:HGNC:23098]	1777.2	2007.7	**1.13**	0.00133
AHSA1	activator of HSP90 ATPase activity 1 [Source:HGNC Symbol;Acc:HGNC:1189]	2137.6	2419.2	**1.13**	0.001385
FRA10AC1	FRA10A associated CGG repeat 1 [Source:HGNC Symbol;Acc:HGNC:1162]	996.8	1117.8	**1.13**	0.001408
CRCP	CGRP receptor component [Source:HGNC Symbol;Acc:HGNC:17888]	1048.8	1182.5	**1.13**	0.001659
RBM14-RBM4	RBM14-RBM4 readthrough [Source:HGNC Symbol;Acc:HGNC:38840]	893.8	1007.5	**1.13**	0.001681
RPS15	ribosomal protein S15 [Source:HGNC Symbol;Acc:HGNC:10388]	1139.1	1282.1	**1.13**	0.001732
FLII	FLII, actin remodeling protein [Source:HGNC Symbol;Acc:HGNC:3750]	4881.2	5511.5	**1.13**	0.001841
MXRA7	matrix remodeling associated 7 [Source:HGNC Symbol;Acc:HGNC:7541]	2674.3	3030.4	**1.13**	0.002462
BCL2L2	BCL2 like 2 [Source:HGNC Symbol;Acc:HGNC:995]	814.1	920.5	**1.13**	0.002716
SMIM14	small integral membrane protein 14 [Source:HGNC Symbol;Acc:HGNC:27321]	14208.0	15978.8	**1.13**	0.002716
RSRC1	arginine and serine rich coiled-coil 1 [Source:HGNC Symbol;Acc:HGNC:24152]	1198.5	1346.7	**1.13**	0.00321
GPN1	GPN-loop GTPase 1 [Source:HGNC Symbol;Acc:HGNC:17030]	768.9	866.9	**1.13**	0.003301
ALG5	ALG5, dolichyl-phosphate beta-glucosyltransferase [Source:HGNC Symbol;Acc:HGNC:20266]	908.0	1024.9	**1.13**	0.003419
CSNK1G1	casein kinase 1 gamma 1 [Source:HGNC Symbol;Acc:HGNC:2454]	680.8	767.1	**1.13**	0.003553
PTPN21	protein tyrosine phosphatase, non-receptor type 21 [Source:HGNC Symbol;Acc:HGNC:9651]	731.3	825.1	**1.13**	0.004151
GART	phosphoribosylglycinamide formyltransferase, phosphoribosylglycinamide synthetase, phosphoribosyla	2147.5	2420.7	**1.13**	0.004583
XPO5	exportin 5 [Source:HGNC Symbol;Acc:HGNC:17675]	720.6	807.8	**1.13**	0.004873
EFCAB13	EF-hand calcium binding domain 13 [Source:HGNC Symbol;Acc:HGNC:26864]	510.7	574.2	**1.13**	0.004974
ZDHHC2	zinc finger DHHC-type containing 2 [Source:HGNC Symbol;Acc:HGNC:18469]	1779.9	1984.5	**1.13**	0.006516
ACIN1	apoptotic chromatin condensation inducer 1 [Source:HGNC Symbol;Acc:HGNC:17066]	2235.0	2519.6	**1.13**	0.006518
AP4E1	adaptor related protein complex 4 subunit epsilon 1 [Source:HGNC Symbol;Acc:HGNC:573]	1099.2	1238.7	**1.13**	0.006551
UBE2K	ubiquitin conjugating enzyme E2 K [Source:HGNC Symbol;Acc:HGNC:4914]	2224.5	2518.0	**1.13**	0.007669
KXD1	KxDL motif containing 1 [Source:HGNC Symbol;Acc:HGNC:28420]	732.9	823.3	**1.13**	0.007681
UBASH3B	ubiquitin associated and SH3 domain containing B [Source:HGNC Symbol;Acc:HGNC:29884]	1610.6	1823.7	**1.13**	0.00811
SPTAN1	spectrin alpha, non-erythrocytic 1 [Source:HGNC Symbol;Acc:HGNC:11273]	6398.0	7200.0	**1.13**	0.008228
TMEM127	transmembrane protein 127 [Source:HGNC Symbol;Acc:HGNC:26038]	745.8	839.3	**1.13**	0.00897
GNA13	G protein subunit alpha 13 [Source:HGNC Symbol;Acc:HGNC:4381]	1266.4	1431.7	**1.13**	0.009858
JPT2	Jupiter microtubule associated homolog 2 [Source:HGNC Symbol;Acc:HGNC:14137]	1357.0	1528.6	**1.13**	0.01013
RPS14	ribosomal protein S14 [Source:HGNC Symbol;Acc:HGNC:10387]	1867.4	2106.2	**1.13**	0.0105
PPP1R11	protein phosphatase 1 regulatory inhibitor subunit 11 [Source:HGNC Symbol;Acc:HGNC:9285]	774.4	867.5	**1.13**	0.01064
STAM	signal transducing adaptor molecule [Source:HGNC Symbol;Acc:HGNC:11357]	700.4	789.9	**1.13**	0.01096
MRPS21	mitochondrial ribosomal protein S21 [Source:HGNC Symbol;Acc:HGNC:14046]	893.4	1006.4	**1.13**	0.01155
HUWE1	HECT, UBA and WWE domain containing 1, E3 ubiquitin protein ligase [Source:HGNC Symbol;Acc:HGNC:	5490.4	6177.3	**1.13**	0.01198
YIPF3	Yip1 domain family member 3 [Source:HGNC Symbol;Acc:HGNC:21023]	1132.8	1276.8	**1.13**	0.01233
OMA1	OMA1 zinc metallopeptidase [Source:HGNC Symbol;Acc:HGNC:29661]	1079.0	1229.6	**1.13**	0.01522
CCDC82	coiled-coil domain containing 82 [Source:HGNC Symbol;Acc:HGNC:26282]	778.0	880.0	**1.13**	0.01589
NIT2	nitrilase family member 2 [Source:HGNC Symbol;Acc:HGNC:29878]	571.4	642.2	**1.13**	0.01658
NGLY1	N-glycanase 1 [Source:HGNC Symbol;Acc:HGNC:17646]	1024.7	1151.4	**1.13**	0.01713
ZSCAN29	zinc finger and SCAN domain containing 29 [Source:HGNC Symbol;Acc:HGNC:26673]	480.0	540.3	**1.13**	0.01757
EHMT1	euchromatic histone lysine methyltransferase 1 [Source:HGNC Symbol;Acc:HGNC:24650]	648.8	728.7	**1.13**	0.01816
NDUFS5	NADH:ubiquinone oxidoreductase subunit S5 [Source:HGNC Symbol;Acc:HGNC:7712]	1709.7	1915.7	**1.13**	0.01832
RCHY1	ring finger and CHY zinc finger domain containing 1 [Source:HGNC Symbol;Acc:HGNC:17479]	463.1	518.7	**1.13**	0.01912
SRRD	SRR1 domain containing [Source:HGNC Symbol;Acc:HGNC:33910]	559.0	628.3	**1.13**	0.02
MMS19	MMS19 homolog, cytosolic iron-sulfur assembly component [Source:HGNC Symbol;Acc:HGNC:13824]	539.3	606.8	**1.13**	0.02002
ZKSCAN5	zinc finger with KRAB and SCAN domains 5 [Source:HGNC Symbol;Acc:HGNC:12867]	430.5	484.2	**1.13**	0.02044
MRPS18A	mitochondrial ribosomal protein S18A [Source:HGNC Symbol;Acc:HGNC:14515]	337.9	380.0	**1.13**	0.02256
PIGS	phosphatidylinositol glycan anchor biosynthesis class S [Source:HGNC Symbol;Acc:HGNC:14937]	581.5	651.3	**1.13**	0.02283
NEAT1	nuclear paraspeckle assembly transcript 1 (non-protein coding) [Source:HGNC Symbol;Acc:HGNC:30815	7818.1	8663.7	**1.13**	0.02398
ABCB7	ATP binding cassette subfamily B member 7 [Source:HGNC Symbol;Acc:HGNC:48]	505.2	568.0	**1.13**	0.02443
TULP3	tubby like protein 3 [Source:HGNC Symbol;Acc:HGNC:12425]	810.9	916.6	**1.13**	0.02457
IMP4	IMP4, U3 small nucleolar ribonucleoprotein [Source:HGNC Symbol;Acc:HGNC:30856]	608.1	681.8	**1.13**	0.0248
TSC2	TSC complex subunit 2 [Source:HGNC Symbol;Acc:HGNC:12363]	406.9	457.6	**1.13**	0.02717
MRPS31	mitochondrial ribosomal protein S31 [Source:HGNC Symbol;Acc:HGNC:16632]	501.7	564.4	**1.13**	0.03356
HAUS4	HAUS augmin like complex subunit 4 [Source:HGNC Symbol;Acc:HGNC:20163]	507.0	569.9	**1.13**	0.03385
ACBD6	acyl-CoA binding domain containing 6 [Source:HGNC Symbol;Acc:HGNC:23339]	538.2	605.5	**1.13**	0.03482
CDC25B	cell division cycle 25B [Source:HGNC Symbol;Acc:HGNC:1726]	312.7	352.2	**1.13**	0.03634
FASTKD1	FAST kinase domains 1 [Source:HGNC Symbol;Acc:HGNC:26150]	469.6	529.8	**1.13**	0.03658
DNAJC3	DnaJ heat shock protein family (Hsp40) member C3 [Source:HGNC Symbol;Acc:HGNC:9439]	10557.0	11816.7	**1.12**	4.09E-07
ARID4B	AT-rich interaction domain 4B [Source:HGNC Symbol;Acc:HGNC:15550]	3950.3	4410.4	**1.12**	6.36E-06
NUP98	nucleoporin 98 [Source:HGNC Symbol;Acc:HGNC:8068]	2422.4	2715.5	**1.12**	7.65E-06
YME1L1	YME1 like 1 ATPase [Source:HGNC Symbol;Acc:HGNC:12843]	6557.3	7323.6	**1.12**	8.56E-06
ERCC5	ERCC excision repair 5, endonuclease [Source:HGNC Symbol;Acc:HGNC:3437]	1846.2	2058.3	**1.12**	1.09E-05
RPS4X	ribosomal protein S4 X-linked [Source:HGNC Symbol;Acc:HGNC:10424]	17144.7	19203.4	**1.12**	2.59E-05
KLHL9	kelch like family member 9 [Source:HGNC Symbol;Acc:HGNC:18732]	3310.1	3706.4	**1.12**	2.80E-05

RAD50	RAD50 double strand break repair protein [Source:HGNC Symbol;Acc:HGNC:9816]	3054.0	3418.2	**1.12**	3.20E-05
RBM27	RNA binding motif protein 27 [Source:HGNC Symbol;Acc:HGNC:29243]	2277.0	2552.2	**1.12**	3.39E-05
BIVM-ERCC5	BIVM-ERCC5 readthrough [Source:HGNC Symbol;Acc:HGNC:43690]	1775.2	1978.2	**1.12**	5.60E-05
ZNF451	zinc finger protein 451 [Source:HGNC Symbol;Acc:HGNC:21091]	2759.7	3090.8	**1.12**	7.75E-05
NEMF	nuclear export mediator factor [Source:HGNC Symbol;Acc:HGNC:10663]	2773.9	3091.7	**1.12**	8.03E-05
ODC1	ornithine decarboxylase 1 [Source:HGNC Symbol;Acc:HGNC:8109]	6219.5	6946.8	**1.12**	9.27E-05
ATP1B3	ATPase Na+/K+ transporting subunit beta 3 [Source:HGNC Symbol;Acc:HGNC:806]	2180.0	2437.6	**1.12**	9.27E-05
RHOA	ras homolog family member A [Source:HGNC Symbol;Acc:HGNC:667]	9871.7	11003.9	**1.12**	0.0001019
YWHAZ	tyrosine 3-monooxygenase/tryptophan 5-monooxygenase activation protein zeta [Source:HGNC Symbo	13140.5	14680.2	**1.12**	0.0001111
RACK1	receptor for activated C kinase 1 [Source:HGNC Symbol;Acc:HGNC:4399]	8508.7	9555.6	**1.12**	0.0001129
ABCF1	ATP binding cassette subfamily F member 1 [Source:HGNC Symbol;Acc:HGNC:70]	1751.9	1967.3	**1.12**	0.0001258
DNM1L	dynamin 1 like [Source:HGNC Symbol;Acc:HGNC:2973]	1908.1	2136.6	**1.12**	0.0001259
BTF3	basic transcription factor 3 [Source:HGNC Symbol;Acc:HGNC:1125]	3887.1	4353.6	**1.12**	0.0001345
NRBP1	nuclear receptor binding protein 1 [Source:HGNC Symbol;Acc:HGNC:7993]	1886.4	2107.9	**1.12**	0.0001937
SF3B2	splicing factor 3b subunit 2 [Source:HGNC Symbol;Acc:HGNC:10769]	3456.1	3877.6	**1.12**	0.0002256
REEP5	receptor accessory protein 5 [Source:HGNC Symbol;Acc:HGNC:30077]	4893.8	5484.2	**1.12**	0.0002376
RAD23B	RAD23 homolog B, nucleotide excision repair protein [Source:HGNC Symbol;Acc:HGNC:9813]	7169.6	8008.1	**1.12**	0.0003425
PIKFYVE	phosphoinositide kinase, FYVE-type zinc finger containing [Source:HGNC Symbol;Acc:HGNC:23785]	1442.9	1614.4	**1.12**	0.0005197
ACOX1	acyl-CoA oxidase 1 [Source:HGNC Symbol;Acc:HGNC:119]	2182.0	2446.9	**1.12**	0.000549
SDF4	stromal cell derived factor 4 [Source:HGNC Symbol;Acc:HGNC:24188]	3759.4	4226.3	**1.12**	0.0005935
YTHDC1	YTH domain containing 1 [Source:HGNC Symbol;Acc:HGNC:30626]	2038.6	2275.8	**1.12**	0.000675
TULP4	tubby like protein 4 [Source:HGNC Symbol;Acc:HGNC:15530]	2778.9	3095.9	**1.12**	0.0007843
RHOBTB3	Rho related BTB domain containing 3 [Source:HGNC Symbol;Acc:HGNC:18757]	8302.2	9366.6	**1.12**	0.000868
EBAG9	estrogen receptor binding site associated, antigen, 9 [Source:HGNC Symbol;Acc:HGNC:3123]	1185.0	1323.7	**1.12**	0.0009167
CSTF2T	cleavage stimulation factor subunit 2 tau variant [Source:HGNC Symbol;Acc:HGNC:17086]	1174.3	1315.5	**1.12**	0.0009937
FAM120AOS	family with sequence similarity 120A opposite strand [Source:HGNC Symbol;Acc:HGNC:23389]	1130.5	1264.9	**1.12**	0.001058
GNL2	G protein nucleolar 2 [Source:HGNC Symbol;Acc:HGNC:29925]	1805.6	2014.4	**1.12**	0.0011
JMJD1C	jumonji domain containing 1C [Source:HGNC Symbol;Acc:HGNC:12313]	3361.8	3768.3	**1.12**	0.001245
ARF1	ADP ribosylation factor 1 [Source:HGNC Symbol;Acc:HGNC:652]	4533.5	5097.4	**1.12**	0.001427
VEZF1	vascular endothelial zinc finger 1 [Source:HGNC Symbol;Acc:HGNC:12949]	1354.5	1514.3	**1.12**	0.00158
HINT1	histidine triad nucleotide binding protein 1 [Source:HGNC Symbol;Acc:HGNC:4912]	1688.8	1893.1	**1.12**	0.001631
DNAJC5	DnaJ heat shock protein family (Hsp40) member C5 [Source:HGNC Symbol;Acc:HGNC:16235]	1386.7	1555.0	**1.12**	0.001945
BCAP31	B cell receptor associated protein 31 [Source:HGNC Symbol;Acc:HGNC:16695]	2337.3	2609.5	**1.12**	0.001949
PDLIM5	PDZ and LIM domain 5 [Source:HGNC Symbol;Acc:HGNC:17468]	10454.2	11587.0	**1.12**	0.002072
NT5C2	5'-nucleotidase, cytosolic II [Source:HGNC Symbol;Acc:HGNC:8022]	2124.3	2374.5	**1.12**	0.002183
RNH1	ribonuclease/angiogenin inhibitor 1 [Source:HGNC Symbol;Acc:HGNC:10074]	2571.8	2878.0	**1.12**	0.002303
MIGA1	mitoguardin 1 [Source:HGNC Symbol;Acc:HGNC:24741]	1312.3	1461.8	**1.12**	0.002339
CD99L2	CD99 molecule like 2 [Source:HGNC Symbol;Acc:HGNC:18237]	1838.4	2063.2	**1.12**	0.002702
EEA1	early endosome antigen 1 [Source:HGNC Symbol;Acc:HGNC:3185]	2947.0	3287.8	**1.12**	0.002863
PCGF5	polycomb group ring finger 5 [Source:HGNC Symbol;Acc:HGNC:28264]	2471.1	2763.1	**1.12**	0.002941
RANGAP1	Ran GTPase activating protein 1 [Source:HGNC Symbol;Acc:HGNC:9854]	832.0	929.9	**1.12**	0.00404
NDUFAF2	NADH:ubiquinone oxidoreductase complex assembly factor 2 [Source:HGNC Symbol;Acc:HGNC:28086]	1032.8	1155.2	**1.12**	0.004411
DDX10	DEAD-box helicase 10 [Source:HGNC Symbol;Acc:HGNC:2735]	980.2	1095.1	**1.12**	0.004606
VPS37A	VPS37A, ESCRT-I subunit [Source:HGNC Symbol;Acc:HGNC:24928]	992.1	1112.7	**1.12**	0.004728
SBF2	SET binding factor 2 [Source:HGNC Symbol;Acc:HGNC:2135]	1809.2	2027.3	**1.12**	0.004736
TPMT	thiopurine S-methyltransferase [Source:HGNC Symbol;Acc:HGNC:12014]	762.1	852.7	**1.12**	0.004952
PUM2	pumilio RNA binding family member 2 [Source:HGNC Symbol;Acc:HGNC:14958]	2247.0	2517.5	**1.12**	0.00499
AKAP9	A-kinase anchoring protein 9 [Source:HGNC Symbol;Acc:HGNC:379]	9153.6	10300.7	**1.12**	0.005126
PCBP2	poly(rC) binding protein 2 [Source:HGNC Symbol;Acc:HGNC:8648]	823.5	920.5	**1.12**	0.006281
GOLGA1	golgin A1 [Source:HGNC Symbol;Acc:HGNC:4424]	852.3	952.5	**1.12**	0.006296
FAM219B	family with sequence similarity 219 member B [Source:HGNC Symbol;Acc:HGNC:24695]	771.7	863.6	**1.12**	0.006872
GUCD1	guanylyl cyclase domain containing 1 [Source:HGNC Symbol;Acc:HGNC:14237]	714.7	799.4	**1.12**	0.007276
CNIH4	cornichon family AMPA receptor auxiliary protein 4 [Source:HGNC Symbol;Acc:HGNC:25013]	1288.1	1440.7	**1.12**	0.007607
C14orf132	chromosome 14 open reading frame 132 [Source:HGNC Symbol;Acc:HGNC:20346]	616.0	685.5	**1.12**	0.007959
EIF4A2	eukaryotic translation initiation factor 4A2 [Source:HGNC Symbol;Acc:HGNC:3284]	10722.7	12147.9	**1.12**	0.008243
WASL	Wiskott-Aldrich syndrome like [Source:HGNC Symbol;Acc:HGNC:12735]	1066.9	1196.0	**1.12**	0.008788
C9orf78	chromosome 9 open reading frame 78 [Source:HGNC Symbol;Acc:HGNC:24932]	1526.0	1702.3	**1.12**	0.008924
PPARA	peroxisome proliferator activated receptor alpha [Source:HGNC Symbol;Acc:HGNC:9232]	1001.1	1116.1	**1.12**	0.01018
CHCHD7	coiled-coil-helix-coiled-coil-helix domain containing 7 [Source:HGNC Symbol;Acc:HGNC:28314]	862.6	960.0	**1.12**	0.01056
TEP1	telomerase associated protein 1 [Source:HGNC Symbol;Acc:HGNC:11726]	875.0	976.0	**1.12**	0.01086
DUSP11	dual specificity phosphatase 11 [Source:HGNC Symbol;Acc:HGNC:3066]	662.9	739.7	**1.12**	0.01201
PPP1CA	protein phosphatase 1 catalytic subunit alpha [Source:HGNC Symbol;Acc:HGNC:9281]	825.3	924.4	**1.12**	0.01202
RBMS1	RNA binding motif single stranded interacting protein 1 [Source:HGNC Symbol;Acc:HGNC:9907]	4155.9	4663.5	**1.12**	0.01309
KPNA1	karyopherin subunit alpha 1 [Source:HGNC Symbol;Acc:HGNC:6394]	1340.4	1502.0	**1.12**	0.01326
LYPLA1	lysophospholipase I [Source:HGNC Symbol;Acc:HGNC:6737]	1363.1	1528.1	**1.12**	0.01378
BMPR2	bone morphogenetic protein receptor type 2 [Source:HGNC Symbol;Acc:HGNC:1078]	1806.2	2019.7	**1.12**	0.01492
DARS2	aspartyl-tRNA synthetase 2, mitochondrial [Source:HGNC Symbol;Acc:HGNC:25538]	557.2	623.1	**1.12**	0.01561
ZNF252P	zinc finger protein 252, pseudogene [Source:HGNC Symbol;Acc:HGNC:13046]	1154.0	1281.6	**1.12**	0.01704
C12orf29	chromosome 12 open reading frame 29 [Source:HGNC Symbol;Acc:HGNC:25322]	741.2	829.6	**1.12**	0.01714
SGTB	small glutamine rich tetratricopeptide repeat containing beta [Source:HGNC Symbol;Acc:HGNC:23567]	1404.4	1563.4	**1.12**	0.01896
ME2	malic enzyme 2 [Source:HGNC Symbol;Acc:HGNC:6984]	873.8	978.2	**1.12**	0.02156
PET100	PET100 homolog [Source:HGNC Symbol;Acc:HGNC:40038]	536.3	596.8	**1.12**	0.02281

SLC1A4	solute carrier family 1 member 4 [Source:HGNC Symbol;Acc:HGNC:10942]	2868.5	3213.8	**1.12**	0.0229
C11orf54	chromosome 11 open reading frame 54 [Source:HGNC Symbol;Acc:HGNC:30204]	862.0	966.5	**1.12**	0.02319
KDM4C	lysine demethylase 4C [Source:HGNC Symbol;Acc:HGNC:17071]	893.7	999.0	**1.12**	0.02348
NSMCE2	NSE2 (MMS21) homolog, SMC5-SMC6 complex SUMO ligase [Source:HGNC Symbol;Acc:HGNC:26513]	543.1	608.6	**1.12**	0.02449
LRIF1	ligand dependent nuclear receptor interacting factor 1 [Source:HGNC Symbol;Acc:HGNC:30299]	589.7	660.1	**1.12**	0.02752
MIPOL1	mirror-image polydactyly 1 [Source:HGNC Symbol;Acc:HGNC:21460]	791.0	886.4	**1.12**	0.02938
TDG	thymine DNA glycosylase [Source:HGNC Symbol;Acc:HGNC:11700]	436.5	487.9	**1.12**	0.02991
TBC1D20	TBC1 domain family member 20 [Source:HGNC Symbol;Acc:HGNC:16133]	730.6	817.7	**1.12**	0.03571
PLCB3	phospholipase C beta 3 [Source:HGNC Symbol;Acc:HGNC:9056]	307.4	344.7	**1.12**	0.03846
TNFAIP1	TNF alpha induced protein 1 [Source:HGNC Symbol;Acc:HGNC:11894]	419.8	472.4	**1.12**	0.04067
FOXK2	forkhead box K2 [Source:HGNC Symbol;Acc:HGNC:6036]	574.8	646.1	**1.12**	0.04142
SHISA5	shisa family member 5 [Source:HGNC Symbol;Acc:HGNC:30376]	669.6	750.8	**1.12**	0.04234
ASB7	ankyrin repeat and SOCS box containing 7 [Source:HGNC Symbol;Acc:HGNC:17182]	425.2	476.2	**1.12**	0.04503
MSRB3	methionine sulfoxide reductase B3 [Source:HGNC Symbol;Acc:HGNC:27375]	1735.9	1929.9	**1.12**	0.04688
ZNF844	zinc finger protein 844 [Source:HGNC Symbol;Acc:HGNC:25932]	578.2	644.2	**1.12**	0.04868
ACER3	alkaline ceramidase 3 [Source:HGNC Symbol;Acc:HGNC:16066]	379.3	420.9	**1.12**	0.04927
PIGT	phosphatidylinositol glycan anchor biosynthesis class T [Source:HGNC Symbol;Acc:HGNC:14938]	722.8	807.7	**1.12**	0.04987
ARMCX3	armadillo repeat containing X-linked 3 [Source:HGNC Symbol;Acc:HGNC:24065]	2468.7	2745.4	**1.11**	6.98E-06
EIF4G1	eukaryotic translation initiation factor 4 gamma 1 [Source:HGNC Symbol;Acc:HGNC:3296]	7977.3	8859.3	**1.11**	3.00E-05
VCL	vinculin [Source:HGNC Symbol;Acc:HGNC:12665]	4439.1	4911.3	**1.11**	3.17E-05
RPL31	ribosomal protein L31 [Source:HGNC Symbol;Acc:HGNC:10334]	13929.7	15476.5	**1.11**	4.70E-05
ATF4	activating transcription factor 4 [Source:HGNC Symbol;Acc:HGNC:786]	8634.1	9580.3	**1.11**	4.92E-05
GNS	glucosamine (N-acetyl)-6-sulfatase [Source:HGNC Symbol;Acc:HGNC:4422]	5679.2	6338.1	**1.11**	7.21E-05
KIF13A	kinesin family member 13A [Source:HGNC Symbol;Acc:HGNC:14566]	1957.7	2182.0	**1.11**	9.39E-05
ASCC3	activating signal cointegrator 1 complex subunit 3 [Source:HGNC Symbol;Acc:HGNC:18697]	3521.5	3919.2	**1.11**	0.0001503
EEF1A1	eukaryotic translation elongation factor 1 alpha 1 [Source:HGNC Symbol;Acc:HGNC:3189]	133693.9	148677.9	**1.11**	0.0002025
STAM2	signal transducing adaptor molecule 2 [Source:HGNC Symbol;Acc:HGNC:11358]	2710.6	2999.6	**1.11**	0.0003497
RSU1	Ras suppressor protein 1 [Source:HGNC Symbol;Acc:HGNC:10464]	2571.2	2853.9	**1.11**	0.0004479
DR1	down-regulator of transcription 1 [Source:HGNC Symbol;Acc:HGNC:3017]	1988.6	2207.3	**1.11**	0.0004806
AP1G1	adaptor related protein complex 1 subunit gamma 1 [Source:HGNC Symbol;Acc:HGNC:555]	2395.5	2657.1	**1.11**	0.0004971
RPL11	ribosomal protein L11 [Source:HGNC Symbol;Acc:HGNC:10301]	9348.2	10416.9	**1.11**	0.0005453
OIP5-AS1	OIP5 antisense RNA 1 [Source:HGNC Symbol;Acc:HGNC:43563]	4433.1	4926.4	**1.11**	0.000549
UBE2H	ubiquitin conjugating enzyme E2 H [Source:HGNC Symbol;Acc:HGNC:12484]	2085.3	2319.2	**1.11**	0.0005776
RPS13	ribosomal protein S13 [Source:HGNC Symbol;Acc:HGNC:10386]	3255.2	3629.2	**1.11**	0.0007197
IPO7	importin 7 [Source:HGNC Symbol;Acc:HGNC:9852]	5948.0	6608.1	**1.11**	0.0007225
PKN2	protein kinase N2 [Source:HGNC Symbol;Acc:HGNC:9406]	1913.2	2126.2	**1.11**	0.000937
SSU72	SSU72 homolog, RNA polymerase II CTD phosphatase [Source:HGNC Symbol;Acc:HGNC:25016]	1579.1	1756.5	**1.11**	0.001129
SOS2	SOS Ras/Rho guanine nucleotide exchange factor 2 [Source:HGNC Symbol;Acc:HGNC:11188]	1760.9	1947.5	**1.11**	0.001246
CAPZA2	capping actin protein of muscle Z-line subunit alpha 2 [Source:HGNC Symbol;Acc:HGNC:1490]	5604.3	6207.2	**1.11**	0.001522
RPL17	ribosomal protein L17 [Source:HGNC Symbol;Acc:HGNC:10307]	12464.5	13830.5	**1.11**	0.001579
CD164	CD164 molecule [Source:HGNC Symbol;Acc:HGNC:1632]	12314.0	13627.9	**1.11**	0.001815
BSG	basigin (Ok blood group) [Source:HGNC Symbol;Acc:HGNC:1116]	1509.6	1680.9	**1.11**	0.001885
HNRNPF	heterogeneous nuclear ribonucleoprotein F [Source:HGNC Symbol;Acc:HGNC:5039]	5596.4	6200.3	**1.11**	0.002037
RUFY2	RUN and FYVE domain containing 2 [Source:HGNC Symbol;Acc:HGNC:19761]	1401.0	1562.0	**1.11**	0.002061
HEXA	hexosaminidase subunit alpha [Source:HGNC Symbol;Acc:HGNC:4878]	1653.8	1842.6	**1.11**	0.002328
NACA	nascent polypeptide associated complex subunit alpha [Source:HGNC Symbol;Acc:HGNC:7629]	14776.0	16510.2	**1.11**	0.002581
RAB22A	RAB22A, member RAS oncogene family [Source:HGNC Symbol;Acc:HGNC:9764]	1749.1	1951.5	**1.11**	0.002619
ETFA	electron transfer flavoprotein subunit alpha [Source:HGNC Symbol;Acc:HGNC:3481]	2055.3	2281.0	**1.11**	0.003041
RPL36A-HNRNPH2	RPL36A-HNRNPH2 readthrough [Source:HGNC Symbol;Acc:HGNC:48349]	3584.1	3987.4	**1.11**	0.003758
BTBD7	BTB domain containing 7 [Source:HGNC Symbol;Acc:HGNC:18269]	1306.0	1445.6	**1.11**	0.004608
UTP23	UTP23, small subunit processome component [Source:HGNC Symbol;Acc:HGNC:28224]	977.3	1082.3	**1.11**	0.00494
DCUN1D1	defective in cullin neddylation 1 domain containing 1 [Source:HGNC Symbol;Acc:HGNC:18184]	1874.4	2074.6	**1.11**	0.005109
CCNL1	cyclin L1 [Source:HGNC Symbol;Acc:HGNC:20569]	955.0	1060.9	**1.11**	0.005716
MAX	MYC associated factor X [Source:HGNC Symbol;Acc:HGNC:6913]	1057.0	1174.4	**1.11**	0.005844
RARS2	arginyl-tRNA synthetase 2, mitochondrial [Source:HGNC Symbol;Acc:HGNC:21406]	1289.2	1433.1	**1.11**	0.006156
COX11	COX11, cytochrome c oxidase copper chaperone [Source:HGNC Symbol;Acc:HGNC:2261]	1248.5	1386.9	**1.11**	0.006516
DENND4A	DENN domain containing 4A [Source:HGNC Symbol;Acc:HGNC:24321]	1344.1	1492.3	**1.11**	0.007328
WDR75	WD repeat domain 75 [Source:HGNC Symbol;Acc:HGNC:25725]	1633.4	1810.4	**1.11**	0.008136
NRDC	nardilysin convertase [Source:HGNC Symbol;Acc:HGNC:7995]	2781.6	3092.2	**1.11**	0.008455
ABR	ABR, RhoGEF and GTPase activating protein [Source:HGNC Symbol;Acc:HGNC:81]	2284.8	2550.9	**1.11**	0.008546
PSMA3-AS1	PSMA3 antisense RNA 1 [Source:HGNC Symbol;Acc:HGNC:26445]	809.2	901.7	**1.11**	0.009231
UNC50	unc-50 inner nuclear membrane RNA binding protein [Source:HGNC Symbol;Acc:HGNC:16046]	1170.5	1300.2	**1.11**	0.009545
RNF130	ring finger protein 130 [Source:HGNC Symbol;Acc:HGNC:18280]	1975.9	2185.5	**1.11**	0.009573
EI24	EI24, autophagy associated transmembrane protein [Source:HGNC Symbol;Acc:HGNC:13276]	1034.0	1150.6	**1.11**	0.01083
ESCO1	establishment of sister chromatid cohesion N-acetyltransferase 1 [Source:HGNC Symbol;Acc:HGNC:246	1235.2	1370.7	**1.11**	0.01166
HPS5	HPS5, biogenesis of lysosomal organelles complex 2 subunit 2 [Source:HGNC Symbol;Acc:HGNC:17022]	1273.1	1411.3	**1.11**	0.01191
CHUK	conserved helix-loop-helix ubiquitous kinase [Source:HGNC Symbol;Acc:HGNC:1974]	889.4	983.1	**1.11**	0.01198
TIMM10B	translocase of inner mitochondrial membrane 10B [Source:HGNC Symbol;Acc:HGNC:4022]	845.1	940.6	**1.11**	0.01232
TTC19	tetratricopeptide repeat domain 19 [Source:HGNC Symbol;Acc:HGNC:26006]	1256.9	1399.9	**1.11**	0.01268
XPC	XPC complex subunit, DNA damage recognition and repair factor [Source:HGNC Symbol;Acc:HGNC:1281	1089.9	1210.0	**1.11**	0.0134
MRPS23	mitochondrial ribosomal protein S23 [Source:HGNC Symbol;Acc:HGNC:14509]	1032.9	1138.9	**1.11**	0.01487
CEP95	centrosomal protein 95 [Source:HGNC Symbol;Acc:HGNC:25141]	528.5	587.5	**1.11**	0.01489

ZNF263	zinc finger protein 263 [Source:HGNC Symbol;Acc:HGNC:13056]	660.2	731.7	**1.11**	0.01635
PGM3	phosphoglucomutase 3 [Source:HGNC Symbol;Acc:HGNC:8907]	4164.7	4650.4	**1.11**	0.01715
IFT46	intraflagellar transport 46 [Source:HGNC Symbol;Acc:HGNC:26146]	969.7	1078.4	**1.11**	0.01841
TWSG1	twisted gastrulation BMP signaling modulator 1 [Source:HGNC Symbol;Acc:HGNC:12429]	7645.2	8522.7	**1.11**	0.01869
INPP5B	inositol polyphosphate-5-phosphatase B [Source:HGNC Symbol;Acc:HGNC:6077]	681.7	758.2	**1.11**	0.0194
PRKAB2	protein kinase AMP-activated non-catalytic subunit beta 2 [Source:HGNC Symbol;Acc:HGNC:9379]	558.0	622.0	**1.11**	0.02285
RTCA	RNA 3'-terminal phosphate cyclase [Source:HGNC Symbol;Acc:HGNC:17981]	1411.9	1570.4	**1.11**	0.02331
GON4L	gon-4 like [Source:HGNC Symbol;Acc:HGNC:25973]	983.6	1099.2	**1.11**	0.02402
TMEM19	transmembrane protein 19 [Source:HGNC Symbol;Acc:HGNC:25605]	1214.2	1349.9	**1.11**	0.02523
SLC30A7	solute carrier family 30 member 7 [Source:HGNC Symbol;Acc:HGNC:19306]	2115.0	2355.4	**1.11**	0.02552
METTL13	methyltransferase like 13 [Source:HGNC Symbol;Acc:HGNC:24248]	467.9	516.8	**1.11**	0.02986
CEP89	centrosomal protein 89 [Source:HGNC Symbol;Acc:HGNC:25907]	636.1	707.0	**1.11**	0.03231
ASB14	ankyrin repeat and SOCS box containing 14 [Source:HGNC Symbol;Acc:HGNC:19766]	513.3	568.7	**1.11**	0.03683
DBR1	debranching RNA lariats 1 [Source:HGNC Symbol;Acc:HGNC:15594]	760.4	842.9	**1.11**	0.03865
GBA2	glucosylceramidase beta 2 [Source:HGNC Symbol;Acc:HGNC:18986]	524.8	584.3	**1.11**	0.04852
HCG11	HLA complex group 11 (non-protein coding) [Source:HGNC Symbol;Acc:HGNC:17707]	681.8	763.1	**1.11**	0.04932
MTDH	metadherin [Source:HGNC Symbol;Acc:HGNC:29608]	7909.0	8693.1	**1.10**	3.32E-05
EXT2	exostosin glycosyltransferase 2 [Source:HGNC Symbol;Acc:HGNC:3513]	4748.3	5232.6	**1.10**	5.15E-05
TAF7	TATA-box binding protein associated factor 7 [Source:HGNC Symbol;Acc:HGNC:11541]	4193.6	4627.4	**1.10**	5.87E-05
DYNC1I2	dynein cytoplasmic 1 intermediate chain 2 [Source:HGNC Symbol;Acc:HGNC:2964]	5532.7	6087.1	**1.10**	6.53E-05
RPLP2	ribosomal protein lateral stalk subunit P2 [Source:HGNC Symbol;Acc:HGNC:10377]	4694.3	5192.9	**1.10**	8.33E-05
SBNO1	strawberry notch homolog 1 [Source:HGNC Symbol;Acc:HGNC:22973]	4064.6	4485.9	**1.10**	9.45E-05
TOR1AIP2	torsin 1A interacting protein 2 [Source:HGNC Symbol;Acc:HGNC:24055]	5767.3	6368.5	**1.10**	9.81E-05
CAPNS1	calpain small subunit 1 [Source:HGNC Symbol;Acc:HGNC:1481]	4586.6	5063.3	**1.10**	0.0001328
USP34	ubiquitin specific peptidase 34 [Source:HGNC Symbol;Acc:HGNC:20066]	4386.0	4842.7	**1.10**	0.0002837
RAB6A	RAB6A, member RAS oncogene family [Source:HGNC Symbol;Acc:HGNC:9786]	4587.3	5064.6	**1.10**	0.0003362
SEPT7	septin 7 [Source:HGNC Symbol;Acc:HGNC:1717]	10212.2	11257.2	**1.10**	0.0005155
TMEM59	transmembrane protein 59 [Source:HGNC Symbol;Acc:HGNC:1239]	7815.8	8656.4	**1.10**	0.0005897
RPS3	ribosomal protein S3 [Source:HGNC Symbol;Acc:HGNC:10420]	6047.6	6697.9	**1.10**	0.000876
APPL1	adaptor protein, phosphotyrosine interacting with PH domain and leucine zipper 1 [Source:HGNC Symb	4970.6	5484.5	**1.10**	0.0009211
GLYR1	glyoxylate reductase 1 homolog [Source:HGNC Symbol;Acc:HGNC:24434]	1335.3	1473.6	**1.10**	0.001069
TAX1BP1	Tax1 binding protein 1 [Source:HGNC Symbol;Acc:HGNC:11575]	5301.8	5866.1	**1.10**	0.001199
B2M	beta-2-microglobulin [Source:HGNC Symbol;Acc:HGNC:914]	10650.1	11742.1	**1.10**	0.001539
SLU7	SLU7 homolog, splicing factor [Source:HGNC Symbol;Acc:HGNC:16939]	2461.5	2723.4	**1.10**	0.001584
PPP4R3B	protein phosphatase 4 regulatory subunit 3B [Source:HGNC Symbol;Acc:HGNC:29267]	3539.7	3902.8	**1.10**	0.001843
TAF1D	TATA-box binding protein associated factor, RNA polymerase I subunit D [Source:HGNC Symbol;Acc:HGI	2869.4	3171.8	**1.10**	0.002062
RPS6	ribosomal protein S6 [Source:HGNC Symbol;Acc:HGNC:10429]	23513.3	25905.8	**1.10**	0.002297
GDE1	glycerophosphodiester phosphodiesterase 1 [Source:HGNC Symbol;Acc:HGNC:29644]	1236.2	1365.5	**1.10**	0.002382
ELP1	elongator complex protein 1 [Source:HGNC Symbol;Acc:HGNC:5959]	1782.2	1957.5	**1.10**	0.002479
TMBIM4	transmembrane BAX inhibitor motif containing 4 [Source:HGNC Symbol;Acc:HGNC:24257]	3320.6	3644.9	**1.10**	0.002549
SNX9	sorting nexin 9 [Source:HGNC Symbol;Acc:HGNC:14973]	2337.4	2574.3	**1.10**	0.002714
PPFIA1	PTPRF interacting protein alpha 1 [Source:HGNC Symbol;Acc:HGNC:9245]	2360.2	2597.4	**1.10**	0.002716
EEF1B2	eukaryotic translation elongation factor 1 beta 2 [Source:HGNC Symbol;Acc:HGNC:3208]	12585.4	13897.1	**1.10**	0.002746
RANBP2	RAN binding protein 2 [Source:HGNC Symbol;Acc:HGNC:9848]	8499.2	9382.7	**1.10**	0.002839
EIF2S1	eukaryotic translation initiation factor 2 subunit alpha [Source:HGNC Symbol;Acc:HGNC:3265]	3769.7	4165.2	**1.10**	0.003059
WAC	WW domain containing adaptor with coiled-coil [Source:HGNC Symbol;Acc:HGNC:17327]	3250.1	3580.6	**1.10**	0.003164
SDAD1	SDA1 domain containing 1 [Source:HGNC Symbol;Acc:HGNC:25537]	1740.9	1922.5	**1.10**	0.003391
CILP	cartilage intermediate layer protein [Source:HGNC Symbol;Acc:HGNC:1980]	9258.2	10270.3	**1.10**	0.003612
FBXW11	F-box and WD repeat domain containing 11 [Source:HGNC Symbol;Acc:HGNC:13607]	1843.8	2034.6	**1.10**	0.003662
MRFAP1L1	Morf4 family associated protein 1 like 1 [Source:HGNC Symbol;Acc:HGNC:28796]	1803.9	1987.5	**1.10**	0.004179
RNF146	ring finger protein 146 [Source:HGNC Symbol;Acc:HGNC:21336]	1075.7	1187.2	**1.10**	0.004362
ATXN3	ataxin 3 [Source:HGNC Symbol;Acc:HGNC:7106]	1659.3	1828.0	**1.10**	0.00479
LARP7	La ribonucleoprotein domain family member 7 [Source:HGNC Symbol;Acc:HGNC:24912]	1614.3	1784.7	**1.10**	0.004959
DNAJB6	DnaJ heat shock protein family (Hsp40) member B6 [Source:HGNC Symbol;Acc:HGNC:14888]	1592.8	1760.8	**1.10**	0.005461
CTR9	CTR9 homolog, Paf1/RNA polymerase II complex component [Source:HGNC Symbol;Acc:HGNC:16850]	1989.8	2192.7	**1.10**	0.005577
DHX8	DEAH-box helicase 8 [Source:HGNC Symbol;Acc:HGNC:2749]	1265.7	1398.7	**1.10**	0.005868
YWHAH	tyrosine 3-monooxygenase/tryptophan 5-monooxygenase activation protein eta [Source:HGNC Symbol;	3539.6	3893.0	**1.10**	0.006615
HIPK1	homeodomain interacting protein kinase 1 [Source:HGNC Symbol;Acc:HGNC:19006]	2047.0	2261.8	**1.10**	0.007171
ARFIP1	ADP ribosylation factor interacting protein 1 [Source:HGNC Symbol;Acc:HGNC:21496]	1549.3	1707.5	**1.10**	0.008179
TXNDC11	thioredoxin domain containing 11 [Source:HGNC Symbol;Acc:HGNC:28030]	1363.5	1506.8	**1.10**	0.008191
SPOPL	speckle type BTB/POZ protein like [Source:HGNC Symbol;Acc:HGNC:27934]	1422.4	1575.1	**1.10**	0.008451
ATG7	autophagy related 7 [Source:HGNC Symbol;Acc:HGNC:16935]	1324.7	1462.6	**1.10**	0.009486
YY1AP1	YY1 associated protein 1 [Source:HGNC Symbol;Acc:HGNC:30935]	1092.1	1206.3	**1.10**	0.009644
UBXN2A	UBX domain protein 2A [Source:HGNC Symbol;Acc:HGNC:27265]	1081.9	1192.0	**1.10**	0.009644
ZMAT2	zinc finger matrin-type 2 [Source:HGNC Symbol;Acc:HGNC:26433]	2259.0	2489.5	**1.10**	0.009913
TINF2	TERF1 interacting nuclear factor 2 [Source:HGNC Symbol;Acc:HGNC:11824]	825.7	910.7	**1.10**	0.0101
PRPF38A	pre-mRNA processing factor 38A [Source:HGNC Symbol;Acc:HGNC:25930]	1264.6	1403.7	**1.10**	0.01071
RPL7A	ribosomal protein L7a [Source:HGNC Symbol;Acc:HGNC:10364]	7670.7	8431.9	**1.10**	0.01085
PRDM4	PR/SET domain 4 [Source:HGNC Symbol;Acc:HGNC:9348]	819.7	900.9	**1.10**	0.0112
BPTF	bromodomain PHD finger transcription factor [Source:HGNC Symbol;Acc:HGNC:3581]	2223.7	2452.6	**1.10**	0.01236
EAF1	ELL associated factor 1 [Source:HGNC Symbol;Acc:HGNC:20907]	936.5	1035.3	**1.10**	0.01655
MFSD14A	major facilitator superfamily domain containing 14A [Source:HGNC Symbol;Acc:HGNC:23363]	1363.2	1499.3	**1.10**	0.01656

EIF5B	eukaryotic translation initiation factor 5B [Source:HGNC Symbol;Acc:HGNC:30793]	2431.2	2700.1	**1.10**	0.01715
RNF103-CHMP3	RNF103-CHMP3 readthrough [Source:HGNC Symbol;Acc:HGNC:38847]	2457.7	2719.1	**1.10**	0.01732
DDX23	DEAD-box helicase 23 [Source:HGNC Symbol;Acc:HGNC:17347]	2317.4	2548.5	**1.10**	0.01747
NUCB1	nucleobindin 1 [Source:HGNC Symbol;Acc:HGNC:8043]	2721.5	3014.0	**1.10**	0.01775
HSDL2	hydroxysteroid dehydrogenase like 2 [Source:HGNC Symbol;Acc:HGNC:18572]	1508.9	1664.2	**1.10**	0.01821
FTSJ3	FtsJ RNA methyltransferase homolog 3 [Source:HGNC Symbol;Acc:HGNC:17136]	2129.2	2336.9	**1.10**	0.01956
TTLL7	tubulin tyrosine ligase like 7 [Source:HGNC Symbol;Acc:HGNC:26242]	1229.9	1348.7	**1.10**	0.01979
NEK9	NIMA related kinase 9 [Source:HGNC Symbol;Acc:HGNC:18591]	1035.5	1146.6	**1.10**	0.01987
DFFA	DNA fragmentation factor subunit alpha [Source:HGNC Symbol;Acc:HGNC:2772]	997.1	1098.9	**1.10**	0.02049
NCBP3	nuclear cap binding subunit 3 [Source:HGNC Symbol;Acc:HGNC:24612]	1080.9	1193.5	**1.10**	0.02303
SEC24A	SEC24 homolog A, COPII coat complex component [Source:HGNC Symbol;Acc:HGNC:10703]	1850.5	2043.5	**1.10**	0.02348
CDR2	cerebellar degeneration related protein 2 [Source:HGNC Symbol;Acc:HGNC:1799]	1283.2	1420.2	**1.10**	0.02555
EIF2B5	eukaryotic translation initiation factor 2B subunit epsilon [Source:HGNC Symbol;Acc:HGNC:3261]	803.9	882.7	**1.10**	0.0259
DHX16	DEAH-box helicase 16 [Source:HGNC Symbol;Acc:HGNC:2739]	832.6	917.2	**1.10**	0.02714
PRMT5-AS1	PRMT5 antisense RNA 1 [Source:HGNC Symbol;Acc:HGNC:40533]	742.9	819.2	**1.10**	0.02782
UBN1	ubinuclein 1 [Source:HGNC Symbol;Acc:HGNC:12506]	1045.6	1149.4	**1.10**	0.0284
DCAF5	DDB1 and CUL4 associated factor 5 [Source:HGNC Symbol;Acc:HGNC:20224]	727.9	806.5	**1.10**	0.02884
LRRC59	leucine rich repeat containing 59 [Source:HGNC Symbol;Acc:HGNC:28817]	4475.0	4986.0	**1.10**	0.03043
GPS2	G protein pathway suppressor 2 [Source:HGNC Symbol;Acc:HGNC:4550]	918.5	1015.5	**1.10**	0.0313
TTC4	tetratricopeptide repeat domain 4 [Source:HGNC Symbol;Acc:HGNC:12394]	734.4	808.4	**1.10**	0.03207
NOP14-AS1	NOP14 antisense RNA 1 [Source:HGNC Symbol;Acc:HGNC:20205]	497.2	550.6	**1.10**	0.03667
NUCB1-AS1	NUCB1 antisense RNA 1 [Source:HGNC Symbol;Acc:HGNC:40419]	717.8	796.7	**1.10**	0.04184
DNAJC2	DnaJ heat shock protein family (Hsp40) member C2 [Source:HGNC Symbol;Acc:HGNC:13192]	945.4	1045.2	**1.10**	0.04301
TM2D1	TM2 domain containing 1 [Source:HGNC Symbol;Acc:HGNC:24142]	1151.0	1276.3	**1.10**	0.0445
SLC35D1	solute carrier family 35 member D1 [Source:HGNC Symbol;Acc:HGNC:20800]	1533.8	1686.1	**1.10**	0.04523
TANGO6	transport and golgi organization 6 homolog [Source:HGNC Symbol;Acc:HGNC:25749]	516.6	570.3	**1.10**	0.04691
LRRC47	leucine rich repeat containing 47 [Source:HGNC Symbol;Acc:HGNC:29207]	541.7	597.4	**1.10**	0.04846
HECTD1	HECT domain E3 ubiquitin protein ligase 1 [Source:HGNC Symbol;Acc:HGNC:20157]	10309.1	11292.5	**1.09**	0.0003085
SRSF11	serine and arginine rich splicing factor 11 [Source:HGNC Symbol;Acc:HGNC:10782]	3746.4	4101.5	**1.09**	0.0004514
PAFAH1B1	platelet activating factor acetylhydrolase 1b regulatory subunit 1 [Source:HGNC Symbol;Acc:HGNC:8574	4295.5	4702.1	**1.09**	0.000827
PPP1R12A	protein phosphatase 1 regulatory subunit 12A [Source:HGNC Symbol;Acc:HGNC:7618]	3398.4	3725.6	**1.09**	0.001058
SP1	Sp1 transcription factor [Source:HGNC Symbol;Acc:HGNC:11205]	1880.0	2060.5	**1.09**	0.001237
EPS8	epidermal growth factor receptor pathway substrate 8 [Source:HGNC Symbol;Acc:HGNC:3420]	14894.6	16340.0	**1.09**	0.001593
ENSA	endosulfine alpha [Source:HGNC Symbol;Acc:HGNC:3360]	2403.4	2634.3	**1.09**	0.001631
SNX22	sorting nexin 22 [Source:HGNC Symbol;Acc:HGNC:16315]	8598.4	9452.0	**1.09**	0.001831
C18orf32	chromosome 18 open reading frame 32 [Source:HGNC Symbol;Acc:HGNC:31690]	13525.1	14874.7	**1.09**	0.002445
ZNF638	zinc finger protein 638 [Source:HGNC Symbol;Acc:HGNC:17894]	5207.4	5700.2	**1.09**	0.002608
MPP6	membrane palmitoylated protein 6 [Source:HGNC Symbol;Acc:HGNC:18167]	6518.2	7165.4	**1.09**	0.002609
VTI1B	vesicle transport through interaction with t-SNAREs 1B [Source:HGNC Symbol;Acc:HGNC:17793]	1763.6	1930.1	**1.09**	0.002657
PPIB	peptidylprolyl isomerase B [Source:HGNC Symbol;Acc:HGNC:9255]	14624.4	16093.3	**1.09**	0.002692
RPL17-C18orf32	RPL17-C18orf32 readthrough [Source:HGNC Symbol;Acc:HGNC:44661]	13458.9	14793.8	**1.09**	0.002704
RPS20	ribosomal protein S20 [Source:HGNC Symbol;Acc:HGNC:10405]	9985.7	10904.8	**1.09**	0.002786
USP7	ubiquitin specific peptidase 7 [Source:HGNC Symbol;Acc:HGNC:12630]	3773.0	4141.1	**1.09**	0.002969
ANXA7	annexin A7 [Source:HGNC Symbol;Acc:HGNC:545]	7154.1	7842.9	**1.09**	0.003098
RAB21	RAB21, member RAS oncogene family [Source:HGNC Symbol;Acc:HGNC:18263]	4299.1	4714.1	**1.09**	0.003167
KCTD2	potassium channel tetramerization domain containing 2 [Source:HGNC Symbol;Acc:HGNC:21294]	1512.2	1654.2	**1.09**	0.004416
ABCD3	ATP binding cassette subfamily D member 3 [Source:HGNC Symbol;Acc:HGNC:67]	2570.6	2817.2	**1.09**	0.004693
SACM1L	SAC1 like phosphatidylinositide phosphatase [Source:HGNC Symbol;Acc:HGNC:17059]	1700.3	1868.5	**1.09**	0.005837
SMG5	SMG5, nonsense mediated mRNA decay factor [Source:HGNC Symbol;Acc:HGNC:24644]	1282.1	1409.4	**1.09**	0.007276
RPS9	ribosomal protein S9 [Source:HGNC Symbol;Acc:HGNC:10442]	3671.3	4008.8	**1.09**	0.008516
HMGB2	high mobility group box 2 [Source:HGNC Symbol;Acc:HGNC:5000]	3081.3	3354.0	**1.09**	0.009216
APLF	aprataxin and PNKP like factor [Source:HGNC Symbol;Acc:HGNC:28724]	1304.8	1431.6	**1.09**	0.009463
RPL36	ribosomal protein L36 [Source:HGNC Symbol;Acc:HGNC:13631]	2736.5	2984.5	**1.09**	0.009551
KIF2A	kinesin family member 2A [Source:HGNC Symbol;Acc:HGNC:6318]	1138.6	1246.1	**1.09**	0.009881
ENDOD1	endonuclease domain containing 1 [Source:HGNC Symbol;Acc:HGNC:29129]	3066.6	3337.9	**1.09**	0.01288
OSBP	oxysterol binding protein [Source:HGNC Symbol;Acc:HGNC:8503]	1586.3	1747.0	**1.09**	0.01426
PLA2R1	phospholipase A2 receptor 1 [Source:HGNC Symbol;Acc:HGNC:9042]	1033.7	1131.6	**1.09**	0.01434
DOPEY1	dopey family member 1 [Source:HGNC Symbol;Acc:HGNC:21194]	1685.6	1845.3	**1.09**	0.01489
NPHP3-ACAD11	NPHP3-ACAD11 readthrough (NMD candidate) [Source:HGNC Symbol;Acc:HGNC:48351]	1560.4	1705.6	**1.09**	0.01538
GNE	glucosamine (UDP-N-acetyl)-2-epimerase/N-acetylmannosamine kinase [Source:HGNC Symbol;Acc:HGN	1392.0	1522.3	**1.09**	0.01542
SCAF8	SR-related CTD associated factor 8 [Source:HGNC Symbol;Acc:HGNC:20959]	1878.8	2046.5	**1.09**	0.01632
RPS18	ribosomal protein S18 [Source:HGNC Symbol;Acc:HGNC:10401]	9589.6	10504.5	**1.09**	0.01675
ZBTB44	zinc finger and BTB domain containing 44 [Source:HGNC Symbol;Acc:HGNC:25001]	1419.2	1550.4	**1.09**	0.01864
RAB4A	RAB4A, member RAS oncogene family [Source:HGNC Symbol;Acc:HGNC:9781]	841.2	922.3	**1.09**	0.01945
UFM1	ubiquitin fold modifier 1 [Source:HGNC Symbol;Acc:HGNC:20597]	4159.1	4579.9	**1.09**	0.01975
BFAR	bifunctional apoptosis regulator [Source:HGNC Symbol;Acc:HGNC:17613]	961.8	1054.7	**1.09**	0.02115
TNIP1	TNFAIP3 interacting protein 1 [Source:HGNC Symbol;Acc:HGNC:16903]	1159.9	1270.6	**1.09**	0.02278
TAOK1	TAO kinase 1 [Source:HGNC Symbol;Acc:HGNC:29259]	4480.3	4908.3	**1.09**	0.02322
ADAM10	ADAM metallopeptidase domain 10 [Source:HGNC Symbol;Acc:HGNC:188]	3281.0	3590.6	**1.09**	0.02348
RPL29	ribosomal protein L29 [Source:HGNC Symbol;Acc:HGNC:10331]	1931.6	2122.2	**1.09**	0.02484
HLA-C	major histocompatibility complex, class I, C [Source:HGNC Symbol;Acc:HGNC:4933]	2972.9	3236.3	**1.09**	0.02575
MARCH6	membrane associated ring-CH-type finger 6 [Source:HGNC Symbol;Acc:HGNC:30550]	1560.6	1706.9	**1.09**	0.02717

RPL39	ribosomal protein L39 [Source:HGNC Symbol;Acc:HGNC:10350]	3061.6	3346.9	**1.09**	0.02723
RPF2	ribosome production factor 2 homolog [Source:HGNC Symbol;Acc:HGNC:20870]	1423.9	1563.3	**1.09**	0.02776
CNNM2	cyclin and CBS domain divalent metal cation transport mediator 2 [Source:HGNC Symbol;Acc:HGNC:103	1192.5	1303.3	**1.09**	0.03271
NCOA5	nuclear receptor coactivator 5 [Source:HGNC Symbol;Acc:HGNC:15909]	515.9	565.9	**1.09**	0.03385
MAP2K4	mitogen-activated protein kinase kinase 4 [Source:HGNC Symbol;Acc:HGNC:6844]	800.5	877.9	**1.09**	0.0374
BOD1L1	biorientation of chromosomes in cell division 1 like 1 [Source:HGNC Symbol;Acc:HGNC:31792]	2601.2	2856.9	**1.09**	0.03823
CPOX	coproporphyrinogen oxidase [Source:HGNC Symbol;Acc:HGNC:2321]	1005.9	1096.4	**1.09**	0.04138
DHX32	DEAH-box helicase 32 (putative) [Source:HGNC Symbol;Acc:HGNC:16717]	867.6	949.9	**1.09**	0.04208
MTRR	5-methyltetrahydrofolate-homocysteine methyltransferase reductase [Source:HGNC Symbol;Acc:HGNC	754.7	828.0	**1.09**	0.04303
FRYL	FRY like transcription coactivator [Source:HGNC Symbol;Acc:HGNC:29127]	2082.0	2272.3	**1.09**	0.04495
FAM20C	FAM20C, golgi associated secretory pathway kinase [Source:HGNC Symbol;Acc:HGNC:22140]	2556.7	2803.7	**1.09**	0.04554
GABARAPL2	GABA type A receptor associated protein like 2 [Source:HGNC Symbol;Acc:HGNC:13291]	1291.2	1413.7	**1.09**	0.04734
MROH7-TTC4	MROH7-TTC4 readthrough (NMD candidate) [Source:HGNC Symbol;Acc:HGNC:49180]	725.8	792.8	**1.09**	0.04891
STRAP	serine/threonine kinase receptor associated protein [Source:HGNC Symbol;Acc:HGNC:30796]	2780.7	3039.2	**1.09**	0.0007861
ZMYND11	zinc finger MYND-type containing 11 [Source:HGNC Symbol;Acc:HGNC:16966]	3867.9	4214.2	**1.09**	0.001101
G3BP2	G3BP stress granule assembly factor 2 [Source:HGNC Symbol;Acc:HGNC:30291]	5620.9	6095.9	**1.09**	0.00119
CTTN	cortactin [Source:HGNC Symbol;Acc:HGNC:3338]	6522.7	7106.1	**1.09**	0.001419
RPN1	ribophorin I [Source:HGNC Symbol;Acc:HGNC:10381]	8596.7	9368.7	**1.09**	0.001486
EXOC5	exocyst complex component 5 [Source:HGNC Symbol;Acc:HGNC:10696]	2840.2	3089.9	**1.09**	0.002113
DYNC1LI2	dynein cytoplasmic 1 light intermediate chain 2 [Source:HGNC Symbol;Acc:HGNC:2966]	3061.3	3335.2	**1.09**	0.002271
DNAJC13	DnaJ heat shock protein family (Hsp40) member C13 [Source:HGNC Symbol;Acc:HGNC:30343]	3640.9	3946.6	**1.09**	0.002846
COX4I1	cytochrome c oxidase subunit 4I1 [Source:HGNC Symbol;Acc:HGNC:2265]	2218.3	2412.3	**1.09**	0.00321
RBM6	RNA binding motif protein 6 [Source:HGNC Symbol;Acc:HGNC:9903]	2820.0	3062.0	**1.09**	0.003455
AZIN1	antizyme inhibitor 1 [Source:HGNC Symbol;Acc:HGNC:16432]	6485.3	7050.7	**1.09**	0.003558
UPF2	UPF2, regulator of nonsense mediated mRNA decay [Source:HGNC Symbol;Acc:HGNC:17854]	2161.6	2348.9	**1.09**	0.003758
RPL12	ribosomal protein L12 [Source:HGNC Symbol;Acc:HGNC:10302]	9726.9	10605.0	**1.09**	0.00407
RPS27A	ribosomal protein S27a [Source:HGNC Symbol;Acc:HGNC:10417]	9236.6	10054.4	**1.09**	0.005612
RTF2	replication termination factor 2 [Source:HGNC Symbol;Acc:HGNC:15890]	2782.2	3033.5	**1.09**	0.005724
SS18	SS18, nBAF chromatin remodeling complex subunit [Source:HGNC Symbol;Acc:HGNC:11340]	2770.7	3002.7	**1.09**	0.00637
PCF11	PCF11, cleavage and polyadenylation factor subunit [Source:HGNC Symbol;Acc:HGNC:30097]	2289.4	2495.2	**1.09**	0.007755
ATP5F1E	ATP synthase F1 subunit epsilon [Source:HGNC Symbol;Acc:HGNC:838]	5393.0	5861.1	**1.09**	0.009331
NUDT21	nudix hydrolase 21 [Source:HGNC Symbol;Acc:HGNC:13870]	3881.1	4213.3	**1.09**	0.009556
CCNT1	cyclin T1 [Source:HGNC Symbol;Acc:HGNC:1599]	1631.7	1771.4	**1.09**	0.009686
APLP2	amyloid beta precursor like protein 2 [Source:HGNC Symbol;Acc:HGNC:598]	20026.0	21779.5	**1.09**	0.0113
CHD1	chromodomain helicase DNA binding protein 1 [Source:HGNC Symbol;Acc:HGNC:1915]	1991.9	2165.9	**1.09**	0.0118
RAP1B	RAP1B, member of RAS oncogene family [Source:HGNC Symbol;Acc:HGNC:9857]	2811.8	3072.5	**1.09**	0.01418
EIF3B	eukaryotic translation initiation factor 3 subunit B [Source:HGNC Symbol;Acc:HGNC:3280]	3177.8	3456.4	**1.09**	0.01663
PARG	poly(ADP-ribose) glycohydrolase [Source:HGNC Symbol;Acc:HGNC:8605]	1141.1	1237.1	**1.09**	0.01669
FAU	FAU, ubiquitin like and ribosomal protein S30 fusion [Source:HGNC Symbol;Acc:HGNC:3597]	1513.1	1650.7	**1.09**	0.01724
MPZL1	myelin protein zero like 1 [Source:HGNC Symbol;Acc:HGNC:7226]	2820.0	3070.9	**1.09**	0.01847
GRB2	growth factor receptor bound protein 2 [Source:HGNC Symbol;Acc:HGNC:4566]	1221.4	1330.0	**1.09**	0.01884
SEC63	SEC63 homolog, protein translocation regulator [Source:HGNC Symbol;Acc:HGNC:21082]	2061.0	2230.7	**1.09**	0.0214
OSTC	oligosaccharyltransferase complex non-catalytic subunit [Source:HGNC Symbol;Acc:HGNC:24448]	6703.7	7305.8	**1.09**	0.02504
SEC24D	SEC24 homolog D, COPII coat complex component [Source:HGNC Symbol;Acc:HGNC:10706]	3929.4	4275.6	**1.09**	0.02623
ATP6AP1	ATPase H+ transporting accessory protein 1 [Source:HGNC Symbol;Acc:HGNC:868]	939.0	1022.0	**1.09**	0.02718
GATC	glutamyl-tRNA amidotransferase subunit C [Source:HGNC Symbol;Acc:HGNC:25068]	1429.1	1558.7	**1.09**	0.02847
GNPTAB	N-acetylglucosamine-1-phosphate transferase subunits alpha and beta [Source:HGNC Symbol;Acc:HGN(	1212.8	1325.0	**1.09**	0.02957
ATP6V1C1	ATPase H+ transporting V1 subunit C1 [Source:HGNC Symbol;Acc:HGNC:856]	1846.6	1994.6	**1.09**	0.02972
TUG1	taurine up-regulated 1 (non-protein coding) [Source:HGNC Symbol;Acc:HGNC:26066]	1911.4	2083.5	**1.09**	0.0306
ZCCHC7	zinc finger CCHC-type containing 7 [Source:HGNC Symbol;Acc:HGNC:26209]	1252.0	1367.8	**1.09**	0.03152
COG8	component of oligomeric golgi complex 8 [Source:HGNC Symbol;Acc:HGNC:18623]	856.4	929.6	**1.09**	0.03471
PEBP1	phosphatidylethanolamine binding protein 1 [Source:HGNC Symbol;Acc:HGNC:8630]	3104.7	3387.5	**1.09**	0.03497
RPS6KC1	ribosomal protein S6 kinase C1 [Source:HGNC Symbol;Acc:HGNC:10439]	1148.9	1246.7	**1.09**	0.03515
CREBZF	CREB/ATF bZIP transcription factor [Source:HGNC Symbol;Acc:HGNC:24905]	908.0	988.8	**1.09**	0.03575
ZNF330	zinc finger protein 330 [Source:HGNC Symbol;Acc:HGNC:15462]	940.3	1019.7	**1.09**	0.03622
MCM3AP	minichromosome maintenance complex component 3 associated protein [Source:HGNC Symbol;Acc:HG	1221.2	1333.4	**1.09**	0.0411
WSB2	WD repeat and SOCS box containing 2 [Source:HGNC Symbol;Acc:HGNC:19222]	2027.5	2209.9	**1.09**	0.04288
TAGLN2	transgelin 2 [Source:HGNC Symbol;Acc:HGNC:11554]	2266.1	2472.3	**1.09**	0.04398
COPZ1	coatomer protein complex subunit zeta 1 [Source:HGNC Symbol;Acc:HGNC:2243]	1452.7	1583.3	**1.09**	0.04873
RALY	RALY heterogeneous nuclear ribonucleoprotein [Source:HGNC Symbol;Acc:HGNC:15921]	935.9	1022.5	**1.09**	0.04891
MAPK6	mitogen-activated protein kinase 6 [Source:HGNC Symbol;Acc:HGNC:6879]	5255.4	5738.4	**1.09**	0.04956
PPID	peptidylprolyl isomerase D [Source:HGNC Symbol;Acc:HGNC:9257]	1217.1	1323.1	**1.09**	0.04968
RPS11	ribosomal protein S11 [Source:HGNC Symbol;Acc:HGNC:10384]	11068.6	11971.7	**1.08**	0.000308
PDIA3	protein disulfide isomerase family A member 3 [Source:HGNC Symbol;Acc:HGNC:4606]	17226.0	18651.1	**1.08**	0.001275
RPN2	ribophorin II [Source:HGNC Symbol;Acc:HGNC:10382]	7006.1	7558.5	**1.08**	0.002702
WAPL	WAPL cohesin release factor [Source:HGNC Symbol;Acc:HGNC:23293]	3013.9	3259.7	**1.08**	0.003237
RAB18	RAB18, member RAS oncogene family [Source:HGNC Symbol;Acc:HGNC:14244]	3509.0	3791.3	**1.08**	0.004487
ARPC5	actin related protein 2/3 complex subunit 5 [Source:HGNC Symbol;Acc:HGNC:708]	3019.2	3255.5	**1.08**	0.004568
KCTD20	potassium channel tetramerization domain containing 20 [Source:HGNC Symbol;Acc:HGNC:21052]	2373.7	2571.9	**1.08**	0.005457
ERGIC3	ERGIC and golgi 3 [Source:HGNC Symbol;Acc:HGNC:15927]	2424.4	2616.7	**1.08**	0.005461
RAB2A	RAB2A, member RAS oncogene family [Source:HGNC Symbol;Acc:HGNC:9763]	4880.4	5254.8	**1.08**	0.006119
GNAS	GNAS complex locus [Source:HGNC Symbol;Acc:HGNC:4392]	4021.9	4346.6	**1.08**	0.007434

IST1	IST1, ESCRT-III associated factor [Source:HGNC Symbol;Acc:HGNC:28977]	2620.5	2830.9	**1.08**	0.007942
THOC2	THO complex 2 [Source:HGNC Symbol;Acc:HGNC:19073]	2559.1	2762.5	**1.08**	0.008347
NCBP2	nuclear cap binding protein subunit 2 [Source:HGNC Symbol;Acc:HGNC:7659]	2143.5	2318.9	**1.08**	0.008573
BROX	BRO1 domain and CAAX motif containing [Source:HGNC Symbol;Acc:HGNC:26512]	3003.6	3249.5	**1.08**	0.008807
EIF1AX	eukaryotic translation initiation factor 1A X-linked [Source:HGNC Symbol;Acc:HGNC:3250]	3323.5	3624.2	**1.08**	0.009004
UBA1	ubiquitin like modifier activating enzyme 1 [Source:HGNC Symbol;Acc:HGNC:12469]	1931.1	2088.1	**1.08**	0.009274
ATP6V1E1	ATPase H+ transporting V1 subunit E1 [Source:HGNC Symbol;Acc:HGNC:857]	3063.1	3308.5	**1.08**	0.01083
MAN1A2	mannosidase alpha class 1A member 2 [Source:HGNC Symbol;Acc:HGNC:6822]	7598.9	8181.0	**1.08**	0.01088
GLTP	glycolipid transfer protein [Source:HGNC Symbol;Acc:HGNC:24867]	1865.1	2010.8	**1.08**	0.01136
KDM1A	lysine demethylase 1A [Source:HGNC Symbol;Acc:HGNC:29079]	1738.6	1877.3	**1.08**	0.01279
NORAD	non-coding RNA activated by DNA damage [Source:HGNC Symbol;Acc:HGNC:44311]	27226.7	29338.1	**1.08**	0.01326
DDOST	dolichyl-diphosphooligosaccharide--protein glycosyltransferase non-catalytic subunit [Source:HGNC Syn	4631.5	4983.7	**1.08**	0.01482
DNAJA2	DnaJ heat shock protein family (Hsp40) member A2 [Source:HGNC Symbol;Acc:HGNC:14884]	3336.4	3608.7	**1.08**	0.01589
HEXB	hexosaminidase subunit beta [Source:HGNC Symbol;Acc:HGNC:4879]	4354.5	4707.7	**1.08**	0.01901
CREB1	cAMP responsive element binding protein 1 [Source:HGNC Symbol;Acc:HGNC:2345]	1872.3	2015.6	**1.08**	0.01902
ARF3	ADP ribosylation factor 3 [Source:HGNC Symbol;Acc:HGNC:654]	1649.8	1781.7	**1.08**	0.01921
THAP12	THAP domain containing 12 [Source:HGNC Symbol;Acc:HGNC:9440]	2692.9	2907.3	**1.08**	0.01951
RIOX2	ribosomal oxygenase 2 [Source:HGNC Symbol;Acc:HGNC:19441]	1962.0	2109.1	**1.08**	0.0214
TRMT5	tRNA methyltransferase 5 [Source:HGNC Symbol;Acc:HGNC:23141]	1067.6	1152.1	**1.08**	0.02148
RPL7	ribosomal protein L7 [Source:HGNC Symbol;Acc:HGNC:10363]	20926.5	22568.1	**1.08**	0.02176
COMMD3	COMM domain containing 3 [Source:HGNC Symbol;Acc:HGNC:23332]	1393.0	1498.6	**1.08**	0.0223
ATF2	activating transcription factor 2 [Source:HGNC Symbol;Acc:HGNC:784]	1430.5	1548.4	**1.08**	0.0229
WDR33	WD repeat domain 33 [Source:HGNC Symbol;Acc:HGNC:25651]	1571.9	1694.0	**1.08**	0.02514
PDCD11	programmed cell death 11 [Source:HGNC Symbol;Acc:HGNC:13408]	1490.5	1612.6	**1.08**	0.02521
DIDO1	death inducer-obliterator 1 [Source:HGNC Symbol;Acc:HGNC:2680]	1575.5	1702.9	**1.08**	0.02606
USP16	ubiquitin specific peptidase 16 [Source:HGNC Symbol;Acc:HGNC:12614]	2816.8	3035.9	**1.08**	0.02812
CPSF2	cleavage and polyadenylation specific factor 2 [Source:HGNC Symbol;Acc:HGNC:2325]	3267.8	3530.0	**1.08**	0.02831
RPS10	ribosomal protein S10 [Source:HGNC Symbol;Acc:HGNC:10383]	5191.2	5595.6	**1.08**	0.03121
EIF3I	eukaryotic translation initiation factor 3 subunit I [Source:HGNC Symbol;Acc:HGNC:3272]	2033.3	2196.1	**1.08**	0.03346
DDX39B	DExD-box helicase 39B [Source:HGNC Symbol;Acc:HGNC:13917]	1649.1	1774.0	**1.08**	0.03497
RUFY1	RUN and FYVE domain containing 1 [Source:HGNC Symbol;Acc:HGNC:19760]	2892.6	3135.8	**1.08**	0.03622
PPP4R3A	protein phosphatase 4 regulatory subunit 3A [Source:HGNC Symbol;Acc:HGNC:20219]	2031.8	2198.4	**1.08**	0.03631
SNX19	sorting nexin 19 [Source:HGNC Symbol;Acc:HGNC:21532]	1447.8	1566.5	**1.08**	0.03753
GNAI3	G protein subunit alpha i3 [Source:HGNC Symbol;Acc:HGNC:4387]	4894.5	5296.0	**1.08**	0.03925
CDYL	chromodomain Y like [Source:HGNC Symbol;Acc:HGNC:1811]	1344.8	1456.1	**1.08**	0.03968
ATP6V1G2-DDX39	ATP6V1G2-DDX39B readthrough (NMD candidate) [Source:HGNC Symbol;Acc:HGNC:41999]	1410.1	1519.4	**1.08**	0.04107
RPL10A	ribosomal protein L10a [Source:HGNC Symbol;Acc:HGNC:10299]	5184.6	5615.5	**1.08**	0.0416
CDC42	cell division cycle 42 [Source:HGNC Symbol;Acc:HGNC:1736]	6189.1	6656.8	**1.08**	0.04196
ELOA	elongin A [Source:HGNC Symbol;Acc:HGNC:11620]	1736.2	1868.3	**1.08**	0.04234
PTCD3	pentatricopeptide repeat domain 3 [Source:HGNC Symbol;Acc:HGNC:24717]	1527.8	1644.5	**1.08**	0.04295
RAB3GAP1	RAB3 GTPase activating protein catalytic subunit 1 [Source:HGNC Symbol;Acc:HGNC:17063]	3774.4	4070.1	**1.08**	0.04305
GLUD1	glutamate dehydrogenase 1 [Source:HGNC Symbol;Acc:HGNC:4335]	3103.4	3368.0	**1.08**	0.04557
NFX1	nuclear transcription factor, X-box binding 1 [Source:HGNC Symbol;Acc:HGNC:7803]	1322.3	1432.7	**1.08**	0.04568
CALM2	calmodulin 2 [Source:HGNC Symbol;Acc:HGNC:1445]	24129.4	26030.8	**1.08**	0.04628
UGGT1	UDP-glucose glycoprotein glucosyltransferase 1 [Source:HGNC Symbol;Acc:HGNC:15663]	6929.7	7430.6	**1.07**	0.0005298
SKP1	S-phase kinase associated protein 1 [Source:HGNC Symbol;Acc:HGNC:10899]	4615.9	4947.1	**1.07**	0.002351
USO1	USO1 vesicle transport factor [Source:HGNC Symbol;Acc:HGNC:30904]	8915.8	9577.1	**1.07**	0.003357
CDC37	cell division cycle 37 [Source:HGNC Symbol;Acc:HGNC:1735]	2615.1	2803.7	**1.07**	0.01093
SMARCA2	SWI/SNF related, matrix associated, actin dependent regulator of chromatin, subfamily a, member 2 [Sc	3771.3	4049.0	**1.07**	0.01376
MIER1	MIER1 transcriptional regulator [Source:HGNC Symbol;Acc:HGNC:29657]	2810.9	3014.4	**1.07**	0.01407
RPL3	ribosomal protein L3 [Source:HGNC Symbol;Acc:HGNC:10332]	9920.1	10640.4	**1.07**	0.01821
EIF3L	eukaryotic translation initiation factor 3 subunit L [Source:HGNC Symbol;Acc:HGNC:18138]	2926.0	3145.7	**1.07**	0.01938
ACTR3	ARP3 actin related protein 3 homolog [Source:HGNC Symbol;Acc:HGNC:170]	11444.7	12332.6	**1.07**	0.02617
CUL3	cullin 3 [Source:HGNC Symbol;Acc:HGNC:2553]	2309.0	2472.9	**1.07**	0.02857
PPIA	peptidylprolyl isomerase A [Source:HGNC Symbol;Acc:HGNC:9253]	8255.9	8881.8	**1.07**	0.02906
VDAC2	voltage dependent anion channel 2 [Source:HGNC Symbol;Acc:HGNC:12672]	1917.7	2054.5	**1.07**	0.0292
RPL21	ribosomal protein L21 [Source:HGNC Symbol;Acc:HGNC:10313]	6482.6	6944.8	**1.07**	0.02944
YES1	YES proto-oncogene 1, Src family tyrosine kinase [Source:HGNC Symbol;Acc:HGNC:12841]	3038.9	3280.9	**1.07**	0.03112
YBX1	Y-box binding protein 1 [Source:HGNC Symbol;Acc:HGNC:8014]	18086.4	19373.2	**1.07**	0.03236
UBE4A	ubiquitination factor E4A [Source:HGNC Symbol;Acc:HGNC:12499]	1783.4	1912.8	**1.07**	0.03563
MMP3	matrix metallopeptidase 3 [Source:HGNC Symbol;Acc:HGNC:7173]	183544.9	197330.6	**1.07**	0.03678
NSA2	NSA2, ribosome biogenesis homolog [Source:HGNC Symbol;Acc:HGNC:30728]	2693.1	2881.7	**1.07**	0.03775
CUL5	cullin 5 [Source:HGNC Symbol;Acc:HGNC:2556]	2145.5	2306.6	**1.07**	0.03847
ANXA5	annexin A5 [Source:HGNC Symbol;Acc:HGNC:543]	21020.8	22601.7	**1.07**	0.03919
ATRAID	all-trans retinoic acid induced differentiation factor [Source:HGNC Symbol;Acc:HGNC:24090]	1775.7	1902.0	**1.07**	0.03976
ARF4	ADP ribosylation factor 4 [Source:HGNC Symbol;Acc:HGNC:655]	4684.1	5014.9	**1.07**	0.04035
ERLEC1	endoplasmic reticulum lectin 1 [Source:HGNC Symbol;Acc:HGNC:25222]	5824.2	6267.8	**1.07**	0.04183
ZMYM4	zinc finger MYM-type containing 4 [Source:HGNC Symbol;Acc:HGNC:13055]	2188.3	2336.3	**1.07**	0.0424
EXOC4	exocyst complex component 4 [Source:HGNC Symbol;Acc:HGNC:30389]	2174.6	2332.2	**1.07**	0.04651
SRP72	signal recognition particle 72 [Source:HGNC Symbol;Acc:HGNC:11303]	3592.9	3830.6	**1.06**	0.007752
CTNNA1	catenin alpha 1 [Source:HGNC Symbol;Acc:HGNC:2509]	13921.5	14834.4	**1.06**	0.009065
U2SURP	U2 snRNP associated SURP domain containing [Source:HGNC Symbol;Acc:HGNC:30855]	2864.0	3051.4	**1.06**	0.01986

ACBD3	acyl-CoA binding domain containing 3 [Source:HGNC Symbol;Acc:HGNC:15453]	4595.6	4906.4	**1.06**	0.02105
RPL6	ribosomal protein L6 [Source:HGNC Symbol;Acc:HGNC:10362]	10476.2	11137.4	**1.06**	0.02542
PDIA6	protein disulfide isomerase family A member 6 [Source:HGNC Symbol;Acc:HGNC:30168]	19734.6	21041.2	**1.06**	0.03046
APP	amyloid beta precursor protein [Source:HGNC Symbol;Acc:HGNC:620]	9903.7	10581.8	**1.06**	0.04241
NMD3	NMD3 ribosome export adaptor [Source:HGNC Symbol;Acc:HGNC:24250]	3141.0	3342.4	**1.06**	0.04471
DDX46	DEAD-box helicase 46 [Source:HGNC Symbol;Acc:HGNC:18681]	2617.8	2780.9	**1.06**	0.04544
TMEM14C	transmembrane protein 14C [Source:HGNC Symbol;Acc:HGNC:20952]	1856.8	1982.2	**1.06**	0.04709
HNRNPC	heterogeneous nuclear ribonucleoprotein C (C1/C2) [Source:HGNC Symbol;Acc:HGNC:5035]	12085.1	12854.7	**1.06**	0.04776
DESI2	desumoylating isopeptidase 2 [Source:HGNC Symbol;Acc:HGNC:24264]	1764.5	1884.2	**1.06**	0.04968
HNRNPK	heterogeneous nuclear ribonucleoprotein K [Source:HGNC Symbol;Acc:HGNC:5044]	18325.0	19359.9	**1.06**	0.00838
COPA	coatomer protein complex subunit alpha [Source:HGNC Symbol;Acc:HGNC:2230]	9836.7	10441.8	**1.06**	0.009393
ANKIB1	ankyrin repeat and IBR domain containing 1 [Source:HGNC Symbol;Acc:HGNC:22215]	2786.7	2951.4	**1.06**	0.03275
DDX5	DEAD-box helicase 5 [Source:HGNC Symbol;Acc:HGNC:2746]	15395.3	16326.9	**1.06**	0.0402
EEF1G	eukaryotic translation elongation factor 1 gamma [Source:HGNC Symbol;Acc:HGNC:3213]	12108.1	12736.3	**1.05**	0.01402
GOLIM4	golgi integral membrane protein 4 [Source:HGNC Symbol;Acc:HGNC:15448]	12042.9	11418.7	**-1.06**	0.03769
HNRNPH1	heterogeneous nuclear ribonucleoprotein H1 [Source:HGNC Symbol;Acc:HGNC:5041]	9003.7	8494.8	**-1.06**	0.04721
AIMP1	aminoacyl tRNA synthetase complex interacting multifunctional protein 1 [Source:HGNC Symbol;Acc:HG	2475.1	2342.4	**-1.06**	0.04846
SP3	Sp3 transcription factor [Source:HGNC Symbol;Acc:HGNC:11208]	3784.0	3584.7	**-1.06**	0.04991
EPRS	glutamyl-prolyl-tRNA synthetase [Source:HGNC Symbol;Acc:HGNC:3418]	13645.8	12785.6	**-1.06**	0.001141
PPIP5K2	diphosphoinositol pentakisphosphate kinase 2 [Source:HGNC Symbol;Acc:HGNC:29035]	2844.6	2665.5	**-1.06**	0.01182
BAZ1B	bromodomain adjacent to zinc finger domain 1B [Source:HGNC Symbol;Acc:HGNC:961]	4473.1	4204.6	**-1.06**	0.02439
CD46	CD46 molecule [Source:HGNC Symbol;Acc:HGNC:6953]	6953.0	6516.5	**-1.06**	0.0346
PRNP	prion protein [Source:HGNC Symbol;Acc:HGNC:9449]	4700.5	4406.5	**-1.06**	0.03792
TMEM167A	transmembrane protein 167A [Source:HGNC Symbol;Acc:HGNC:28330]	5915.4	5552.9	**-1.06**	0.04099
KIAA0232	KIAA0232 [Source:HGNC Symbol;Acc:HGNC:28992]	3128.4	2935.6	**-1.06**	0.04664
SMARCE1	SWI/SNF related, matrix associated, actin dependent regulator of chromatin, subfamily e, member 1 [Sc	3471.6	3249.3	**-1.07**	0.01521
YWHAG	tyrosine 3-monooxygenase/tryptophan 5-monooxygenase activation protein gamma [Source:HGNC Syn	3925.4	3677.5	**-1.07**	0.0282
CWC27	CWC27 spliceosome associated protein homolog [Source:HGNC Symbol;Acc:HGNC:10664]	1174.6	1094.4	**-1.07**	0.02948
APOLD1	apolipoprotein L domain containing 1 [Source:HGNC Symbol;Acc:HGNC:25268]	1348.1	1254.4	**-1.07**	0.04183
KPNB1	karyopherin subunit beta 1 [Source:HGNC Symbol;Acc:HGNC:6400]	8891.1	8304.2	**-1.07**	0.0434
ERBIN	erbb2 interacting protein [Source:HGNC Symbol;Acc:HGNC:15842]	4013.8	3713.4	**-1.08**	0.001378
PSMB7	proteasome subunit beta 7 [Source:HGNC Symbol;Acc:HGNC:9544]	2790.2	2580.7	**-1.08**	0.003248
VCP	valosin containing protein [Source:HGNC Symbol;Acc:HGNC:12666]	9093.4	8420.2	**-1.08**	0.003643
PPIG	peptidylprolyl isomerase G [Source:HGNC Symbol;Acc:HGNC:14650]	3102.7	2878.6	**-1.08**	0.003733
NARS	asparaginyl-tRNA synthetase [Source:HGNC Symbol;Acc:HGNC:7643]	5253.0	4870.5	**-1.08**	0.003817
HBS1L	HBS1 like translational GTPase [Source:HGNC Symbol;Acc:HGNC:4834]	3130.6	2892.2	**-1.08**	0.005469
RBFOX2	RNA binding fox-1 homolog 2 [Source:HGNC Symbol;Acc:HGNC:9906]	2911.9	2691.2	**-1.08**	0.008072
EIF4G3	eukaryotic translation initiation factor 4 gamma 3 [Source:HGNC Symbol;Acc:HGNC:3298]	2816.3	2612.3	**-1.08**	0.009481
MORF4L2	mortality factor 4 like 2 [Source:HGNC Symbol;Acc:HGNC:16849]	7995.1	7390.9	**-1.08**	0.01759
PSMD13	proteasome 26S subunit, non-ATPase 13 [Source:HGNC Symbol;Acc:HGNC:9558]	1957.7	1806.3	**-1.08**	0.02233
IMPACT	impact RWD domain protein [Source:HGNC Symbol;Acc:HGNC:20387]	1829.9	1692.1	**-1.08**	0.0288
PTTG1IP	PTTG1 interacting protein [Source:HGNC Symbol;Acc:HGNC:13524]	24265.5	22284.3	**-1.08**	0.03193
PRKD3	protein kinase D3 [Source:HGNC Symbol;Acc:HGNC:9408]	1891.2	1758.1	**-1.08**	0.03385
TCF25	transcription factor 25 [Source:HGNC Symbol;Acc:HGNC:29181]	1339.1	1240.2	**-1.08**	0.03617
CCT7	chaperonin containing TCP1 subunit 7 [Source:HGNC Symbol;Acc:HGNC:1622]	4179.6	3862.7	**-1.08**	0.03651
NHLRC3	NHL repeat containing 3 [Source:HGNC Symbol;Acc:HGNC:33751]	883.4	819.8	**-1.08**	0.0448
GGPS1	geranylgeranyl diphosphate synthase 1 [Source:HGNC Symbol;Acc:HGNC:4249]	1085.7	1004.5	**-1.08**	0.04664
ATP5F1A	ATP synthase F1 subunit alpha [Source:HGNC Symbol;Acc:HGNC:823]	7949.4	7360.5	**-1.08**	0.04757
SPECC1	sperm antigen with calponin homology and coiled-coil domains 1 [Source:HGNC Symbol;Acc:HGNC:3061	4875.9	4480.9	**-1.09**	0.0001607
ACP1	acid phosphatase 1 [Source:HGNC Symbol;Acc:HGNC:122]	2175.1	1995.9	**-1.09**	0.001768
BBX	BBX, HMG-box containing [Source:HGNC Symbol;Acc:HGNC:14422]	3623.6	3340.5	**-1.09**	0.001937
AP2M1	adaptor related protein complex 2 subunit mu 1 [Source:HGNC Symbol;Acc:HGNC:564]	5082.8	4683.7	**-1.09**	0.00272
PSMD2	proteasome 26S subunit, non-ATPase 2 [Source:HGNC Symbol;Acc:HGNC:9559]	3207.1	2963.6	**-1.09**	0.004931
RBMX	RNA binding motif protein X-linked [Source:HGNC Symbol;Acc:HGNC:9910]	3971.6	3667.8	**-1.09**	0.006091
PSMB1	proteasome subunit beta 1 [Source:HGNC Symbol;Acc:HGNC:9537]	3401.7	3135.8	**-1.09**	0.0101
MARS	methionyl-tRNA synthetase [Source:HGNC Symbol;Acc:HGNC:6898]	3257.6	3001.8	**-1.09**	0.01106
HNRNPA3	heterogeneous nuclear ribonucleoprotein A3 [Source:HGNC Symbol;Acc:HGNC:24941]	2692.3	2473.0	**-1.09**	0.01326
RASA1	RAS p21 protein activator 1 [Source:HGNC Symbol;Acc:HGNC:9871]	2953.9	2709.9	**-1.09**	0.01847
SNRNP70	small nuclear ribonucleoprotein U1 subunit 70 [Source:HGNC Symbol;Acc:HGNC:11150]	1596.8	1469.0	**-1.09**	0.02666
BCL2L2-PABPN1	BCL2L2-PABPN1 readthrough [Source:HGNC Symbol;Acc:HGNC:42959]	1179.1	1085.7	**-1.09**	0.02721
SMC1A	structural maintenance of chromosomes 1A [Source:HGNC Symbol;Acc:HGNC:11111]	2535.1	2339.8	**-1.09**	0.0297
PDS5B	PDS5 cohesin associated factor B [Source:HGNC Symbol;Acc:HGNC:20418]	1792.8	1652.3	**-1.09**	0.02992
CSRP1	cysteine and glycine rich protein 1 [Source:HGNC Symbol;Acc:HGNC:2469]	1402.1	1286.8	**-1.09**	0.03342
TOMM20	translocase of outer mitochondrial membrane 20 [Source:HGNC Symbol;Acc:HGNC:20947]	3963.4	3638.5	**-1.09**	0.03678
VPS8	VPS8, CORVET complex subunit [Source:HGNC Symbol;Acc:HGNC:29122]	1315.0	1214.0	**-1.09**	0.03765
SCAMP1	secretory carrier membrane protein 1 [Source:HGNC Symbol;Acc:HGNC:10563]	3376.7	3086.5	**-1.09**	0.03784
ATM	ATM serine/threonine kinase [Source:HGNC Symbol;Acc:HGNC:795]	4619.2	4234.4	**-1.09**	0.0404
ORMDL3	ORMDL sphingolipid biosynthesis regulator 3 [Source:HGNC Symbol;Acc:HGNC:16038]	1077.9	993.5	**-1.09**	0.04446
RNPEP	arginyl aminopeptidase [Source:HGNC Symbol;Acc:HGNC:10078]	722.1	665.0	**-1.09**	0.04721
DDX1	DEAD-box helicase 1 [Source:HGNC Symbol;Acc:HGNC:2734]	5358.0	4915.3	**-1.09**	0.0001712
SSR1	signal sequence receptor subunit 1 [Source:HGNC Symbol;Acc:HGNC:11323]	9050.5	8307.9	**-1.09**	0.001069
METAP2	methionyl aminopeptidase 2 [Source:HGNC Symbol;Acc:HGNC:16672]	5247.2	4790.7	**-1.09**	0.001308

NSD1	nuclear receptor binding SET domain protein 1 [Source:HGNC Symbol;Acc:HGNC:14234]	2163.4	1969.3	**-1.09**	0.001508
MICU2	mitochondrial calcium uptake 2 [Source:HGNC Symbol;Acc:HGNC:31830]	1987.0	1813.8	**-1.09**	0.002029
PSMC2	proteasome 26S subunit, ATPase 2 [Source:HGNC Symbol;Acc:HGNC:9548]	5271.4	4840.0	**-1.09**	0.002518
TCEAL4	transcription elongation factor A like 4 [Source:HGNC Symbol;Acc:HGNC:26121]	2532.2	2315.8	**-1.09**	0.004287
EML1	echinoderm microtubule associated protein like 1 [Source:HGNC Symbol;Acc:HGNC:3330]	1810.7	1656.5	**-1.09**	0.004822
LMAN1	lectin, mannose binding 1 [Source:HGNC Symbol;Acc:HGNC:6631]	13646.8	12431.3	**-1.09**	0.007901
COX6A1	cytochrome c oxidase subunit 6A1 [Source:HGNC Symbol;Acc:HGNC:2277]	1349.6	1230.6	**-1.09**	0.008763
ATPAF1	ATP synthase mitochondrial F1 complex assembly factor 1 [Source:HGNC Symbol;Acc:HGNC:18803]	1338.6	1223.9	**-1.09**	0.009477
FDFT1	farnesyl-diphosphate farnesyltransferase 1 [Source:HGNC Symbol;Acc:HGNC:3629]	2978.9	2727.5	**-1.09**	0.01019
POLR2D	RNA polymerase II subunit D [Source:HGNC Symbol;Acc:HGNC:9191]	899.1	819.3	**-1.09**	0.01051
PSMA3	proteasome subunit alpha 3 [Source:HGNC Symbol;Acc:HGNC:9532]	2209.4	2021.0	**-1.09**	0.01105
TNPO1	transportin 1 [Source:HGNC Symbol;Acc:HGNC:6401]	6750.9	6185.0	**-1.09**	0.0126
HDHD2	haloacid dehalogenase like hydrolase domain containing 2 [Source:HGNC Symbol;Acc:HGNC:25364]	1023.0	931.1	**-1.09**	0.01626
ARF6	ADP ribosylation factor 6 [Source:HGNC Symbol;Acc:HGNC:659]	3189.9	2929.9	**-1.09**	0.01878
SUB1	SUB1 homolog, transcriptional regulator [Source:HGNC Symbol;Acc:HGNC:19985]	3089.0	2840.0	**-1.09**	0.01942
TOR1A	torsin family 1 member A [Source:HGNC Symbol;Acc:HGNC:3098]	830.0	758.0	**-1.09**	0.01965
COPS3	COP9 signalosome subunit 3 [Source:HGNC Symbol;Acc:HGNC:2239]	1838.2	1681.9	**-1.09**	0.02331
KIAA1191	KIAA1191 [Source:HGNC Symbol;Acc:HGNC:29209]	1661.5	1512.2	**-1.09**	0.02437
MTMR10	myotubularin related protein 10 [Source:HGNC Symbol;Acc:HGNC:25999]	1129.0	1037.1	**-1.09**	0.02451
FAM208A	family with sequence similarity 208 member A [Source:HGNC Symbol;Acc:HGNC:30314]	3639.4	3329.3	**-1.09**	0.02547
AGO1	argonaute 1, RISC catalytic component [Source:HGNC Symbol;Acc:HGNC:3262]	1151.6	1051.1	**-1.09**	0.02854
GMPS	guanine monophosphate synthase [Source:HGNC Symbol;Acc:HGNC:4378]	2340.5	2143.6	**-1.09**	0.03038
TTC3	tetratricopeptide repeat domain 3 [Source:HGNC Symbol;Acc:HGNC:12393]	10738.0	9798.5	**-1.09**	0.0326
TXNL4A	thioredoxin like 4A [Source:HGNC Symbol;Acc:HGNC:30551]	952.6	870.1	**-1.09**	0.03277
PGM1	phosphoglucomutase 1 [Source:HGNC Symbol;Acc:HGNC:8905]	3344.7	3068.1	**-1.09**	0.03385
DIABLO	diablo IAP-binding mitochondrial protein [Source:HGNC Symbol;Acc:HGNC:21528]	868.1	794.7	**-1.09**	0.03507
TMEM131	transmembrane protein 131 [Source:HGNC Symbol;Acc:HGNC:30366]	1530.7	1403.2	**-1.09**	0.03769
PSMA2	proteasome subunit alpha 2 [Source:HGNC Symbol;Acc:HGNC:9531]	2375.8	2171.3	**-1.09**	0.04739
RPS27L	ribosomal protein S27 like [Source:HGNC Symbol;Acc:HGNC:18476]	3332.2	3055.3	**-1.09**	0.04964
ST13	ST13, Hsp70 interacting protein [Source:HGNC Symbol;Acc:HGNC:11343]	6101.3	5552.4	**-1.10**	0.0002286
SARAF	store-operated calcium entry associated regulatory factor [Source:HGNC Symbol;Acc:HGNC:28789]	6085.5	5537.0	**-1.10**	0.001126
DLD	dihydrolipoamide dehydrogenase [Source:HGNC Symbol;Acc:HGNC:2898]	2534.3	2304.7	**-1.10**	0.001355
NDUFA5	NADH:ubiquinone oxidoreductase subunit A5 [Source:HGNC Symbol;Acc:HGNC:7688]	2967.0	2691.8	**-1.10**	0.001606
CKAP5	cytoskeleton associated protein 5 [Source:HGNC Symbol;Acc:HGNC:28959]	4997.5	4514.5	**-1.10**	0.001684
SUCLA2	succinate-CoA ligase ADP-forming beta subunit [Source:HGNC Symbol;Acc:HGNC:11448]	1686.9	1527.1	**-1.10**	0.001878
UTP14C	UTP14C, small subunit processome component [Source:HGNC Symbol;Acc:HGNC:20321]	2267.8	2057.3	**-1.10**	0.002243
PARK7	Parkinsonism associated deglycase [Source:HGNC Symbol;Acc:HGNC:16369]	6524.5	5915.6	**-1.10**	0.002273
COQ10B	coenzyme Q10B [Source:HGNC Symbol;Acc:HGNC:25819]	2081.3	1886.8	**-1.10**	0.002863
ILF2	interleukin enhancer binding factor 2 [Source:HGNC Symbol;Acc:HGNC:6037]	3483.8	3159.2	**-1.10**	0.003353
PANK2	pantothenate kinase 2 [Source:HGNC Symbol;Acc:HGNC:15894]	1635.6	1482.6	**-1.10**	0.003589
CNOT7	CCR4-NOT transcription complex subunit 7 [Source:HGNC Symbol;Acc:HGNC:14101]	2286.5	2082.9	**-1.10**	0.003812
VBP1	VHL binding protein 1 [Source:HGNC Symbol;Acc:HGNC:12662]	1802.0	1632.1	**-1.10**	0.004119
G3BP1	G3BP stress granule assembly factor 1 [Source:HGNC Symbol;Acc:HGNC:30292]	8590.0	7808.3	**-1.10**	0.004883
ITM2C	integral membrane protein 2C [Source:HGNC Symbol;Acc:HGNC:6175]	2220.1	2020.5	**-1.10**	0.004889
ZFP91-CNTF	ZFP91-CNTF readthrough (NMD candidate) [Source:HGNC Symbol;Acc:HGNC:33441]	1590.3	1441.2	**-1.10**	0.005322
SLC39A9	solute carrier family 39 member 9 [Source:HGNC Symbol;Acc:HGNC:20182]	3563.3	3251.0	**-1.10**	0.005677
AGPAT5	1-acylglycerol-3-phosphate O-acyltransferase 5 [Source:HGNC Symbol;Acc:HGNC:20886]	1461.7	1334.2	**-1.10**	0.006232
RMND5A	required for meiotic nuclear division 5 homolog A [Source:HGNC Symbol;Acc:HGNC:25850]	2121.9	1924.3	**-1.10**	0.006853
TIPRL	TOR signaling pathway regulator [Source:HGNC Symbol;Acc:HGNC:30231]	1633.3	1481.8	**-1.10**	0.007069
MRPL3	mitochondrial ribosomal protein L3 [Source:HGNC Symbol;Acc:HGNC:10379]	2504.3	2281.2	**-1.10**	0.008173
NDUFC2	NADH:ubiquinone oxidoreductase subunit C2 [Source:HGNC Symbol;Acc:HGNC:7706]	1095.3	993.6	**-1.10**	0.008377
CALM1	calmodulin 1 [Source:HGNC Symbol;Acc:HGNC:1442]	9825.4	8949.9	**-1.10**	0.009594
HRH1	histamine receptor H1 [Source:HGNC Symbol;Acc:HGNC:5182]	2479.1	2251.2	**-1.10**	0.01085
ELOVL5	ELOVL fatty acid elongase 5 [Source:HGNC Symbol;Acc:HGNC:21308]	2773.6	2509.1	**-1.10**	0.01104
LMBRD2	LMBR1 domain containing 2 [Source:HGNC Symbol;Acc:HGNC:25287]	1513.5	1370.8	**-1.10**	0.01111
ATF7IP	activating transcription factor 7 interacting protein [Source:HGNC Symbol;Acc:HGNC:20092]	1053.2	956.9	**-1.10**	0.01225
GDI1	GDP dissociation inhibitor 1 [Source:HGNC Symbol;Acc:HGNC:4226]	1437.5	1305.6	**-1.10**	0.01246
FOPNL	FGFR1OP N-terminal like [Source:HGNC Symbol;Acc:HGNC:26435]	1034.2	942.9	**-1.10**	0.01423
NDUFB5	NADH:ubiquinone oxidoreductase subunit B5 [Source:HGNC Symbol;Acc:HGNC:7700]	1261.7	1142.6	**-1.10**	0.01465
GANAB	glucosidase II alpha subunit [Source:HGNC Symbol;Acc:HGNC:4138]	5272.6	4782.4	**-1.10**	0.015
CTBS	chitobiase [Source:HGNC Symbol;Acc:HGNC:2496]	1224.3	1114.1	**-1.10**	0.01611
ALG11	ALG11, alpha-1,2-mannosyltransferase [Source:HGNC Symbol;Acc:HGNC:32456]	1285.9	1165.1	**-1.10**	0.01716
PFN1	profilin 1 [Source:HGNC Symbol;Acc:HGNC:8881]	2115.6	1910.3	**-1.10**	0.01719
ACTL6A	actin like 6A [Source:HGNC Symbol;Acc:HGNC:24124]	2329.6	2117.8	**-1.10**	0.02047
TRAPPC3	trafficking protein particle complex 3 [Source:HGNC Symbol;Acc:HGNC:19942]	1006.4	912.9	**-1.10**	0.02322
NUMA1	nuclear mitotic apparatus protein 1 [Source:HGNC Symbol;Acc:HGNC:8059]	4009.8	3652.0	**-1.10**	0.02567
CTDSPL2	CTD small phosphatase like 2 [Source:HGNC Symbol;Acc:HGNC:26936]	1434.8	1307.2	**-1.10**	0.02648
SRSF1	serine and arginine rich splicing factor 1 [Source:HGNC Symbol;Acc:HGNC:10780]	8112.3	7398.4	**-1.10**	0.02839
UCHL5	ubiquitin C-terminal hydrolase L5 [Source:HGNC Symbol;Acc:HGNC:19678]	1132.7	1034.2	**-1.10**	0.02901
NEDD1	neural precursor cell expressed, developmentally down-regulated 1 [Source:HGNC Symbol;Acc:HGNC:7	929.8	844.5	**-1.10**	0.03075
CDK4	cyclin dependent kinase 4 [Source:HGNC Symbol;Acc:HGNC:1773]	1024.9	930.1	**-1.10**	0.03164
LPCAT1	lysophosphatidylcholine acyltransferase 1 [Source:HGNC Symbol;Acc:HGNC:25718]	1336.2	1210.2	**-1.10**	0.0398

S100PBP	S100P binding protein [Source:HGNC Symbol;Acc:HGNC:25768]	752.9	686.5	**-1.10**	0.04164
NACC2	NACC family member 2 [Source:HGNC Symbol;Acc:HGNC:23846]	651.9	593.7	**-1.10**	0.04932
USP1	ubiquitin specific peptidase 1 [Source:HGNC Symbol;Acc:HGNC:12607]	3672.2	3317.7	**-1.11**	0.0002245
ACLY	ATP citrate lyase [Source:HGNC Symbol;Acc:HGNC:115]	6332.4	5733.5	**-1.11**	0.0002397
NUCB2	nucleobindin 2 [Source:HGNC Symbol;Acc:HGNC:8044]	8151.8	7377.6	**-1.11**	0.0002936
TSPYL1	TSPY like 1 [Source:HGNC Symbol;Acc:HGNC:12382]	3149.0	2841.3	**-1.11**	0.0003307
HDGF	heparin binding growth factor [Source:HGNC Symbol;Acc:HGNC:4856]	5044.2	4559.5	**-1.11**	0.0003321
RPRD1A	regulation of nuclear pre-mRNA domain containing 1A [Source:HGNC Symbol;Acc:HGNC:25560]	2385.6	2150.5	**-1.11**	0.001882
ACADM	acyl-CoA dehydrogenase medium chain [Source:HGNC Symbol;Acc:HGNC:89]	1982.2	1783.8	**-1.11**	0.001998
XPO7	exportin 7 [Source:HGNC Symbol;Acc:HGNC:14108]	1094.8	990.5	**-1.11**	0.002277
ILF3	interleukin enhancer binding factor 3 [Source:HGNC Symbol;Acc:HGNC:6038]	2579.1	2326.5	**-1.11**	0.00233
TCAF1	TRPM8 channel associated factor 1 [Source:HGNC Symbol;Acc:HGNC:22201]	1105.1	997.5	**-1.11**	0.003189
SELENOT	selenoprotein T [Source:HGNC Symbol;Acc:HGNC:18136]	3506.0	3159.5	**-1.11**	0.004498
PABPN1	poly(A) binding protein nuclear 1 [Source:HGNC Symbol;Acc:HGNC:8565]	1386.2	1250.6	**-1.11**	0.004638
LDLR	low density lipoprotein receptor [Source:HGNC Symbol;Acc:HGNC:6547]	1618.6	1455.0	**-1.11**	0.004683
CCDC59	coiled-coil domain containing 59 [Source:HGNC Symbol;Acc:HGNC:25005]	1061.3	957.3	**-1.11**	0.004952
IER3IP1	immediate early response 3 interacting protein 1 [Source:HGNC Symbol;Acc:HGNC:18550]	2139.8	1923.8	**-1.11**	0.00556
DTD1	D-tyrosyl-tRNA deacylase 1 [Source:HGNC Symbol;Acc:HGNC:16219]	1395.8	1257.1	**-1.11**	0.007133
RNF187	ring finger protein 187 [Source:HGNC Symbol;Acc:HGNC:27146]	910.0	822.7	**-1.11**	0.007359
PSPC1	paraspeckle component 1 [Source:HGNC Symbol;Acc:HGNC:20320]	1332.7	1201.6	**-1.11**	0.0078
GNG10	G protein subunit gamma 10 [Source:HGNC Symbol;Acc:HGNC:4402]	1570.8	1415.6	**-1.11**	0.008514
KCTD14	potassium channel tetramerization domain containing 14 [Source:HGNC Symbol;Acc:HGNC:23295]	860.8	777.1	**-1.11**	0.01009
SRSF3	serine and arginine rich splicing factor 3 [Source:HGNC Symbol;Acc:HGNC:10785]	8172.6	7410.7	**-1.11**	0.0105
DNAJC25-GNG10	DNAJC25-GNG10 readthrough [Source:HGNC Symbol;Acc:HGNC:37501]	1632.4	1479.2	**-1.11**	0.01191
RSBN1L	round spermatid basic protein 1 like [Source:HGNC Symbol;Acc:HGNC:24765]	901.4	814.9	**-1.11**	0.01204
NDUFC2-KCTD14	NDUFC2-KCTD14 readthrough [Source:HGNC Symbol;Acc:HGNC:42956]	785.6	708.0	**-1.11**	0.01335
PRDX5	peroxiredoxin 5 [Source:HGNC Symbol;Acc:HGNC:9355]	2186.0	1975.0	**-1.11**	0.01499
SCOC	short coiled-coil protein [Source:HGNC Symbol;Acc:HGNC:20335]	2328.3	2097.7	**-1.11**	0.01535
NUP205	nucleoporin 205 [Source:HGNC Symbol;Acc:HGNC:18658]	1855.2	1677.0	**-1.11**	0.01731
PEX2	peroxisomal biogenesis factor 2 [Source:HGNC Symbol;Acc:HGNC:9717]	764.8	688.1	**-1.11**	0.01807
PJA1	praja ring finger ubiquitin ligase 1 [Source:HGNC Symbol;Acc:HGNC:16648]	892.1	807.1	**-1.11**	0.01831
NXPE3	neurexophilin and PC-esterase domain family member 3 [Source:HGNC Symbol;Acc:HGNC:28238]	1082.1	979.9	**-1.11**	0.01837
TBC1D1	TBC1 domain family member 1 [Source:HGNC Symbol;Acc:HGNC:11578]	3598.0	3259.3	**-1.11**	0.0211
MRNIP	MRN complex interacting protein [Source:HGNC Symbol;Acc:HGNC:30817]	4953.8	4470.7	**-1.11**	0.02264
GNAQ	G protein subunit alpha q [Source:HGNC Symbol;Acc:HGNC:4390]	1356.2	1226.1	**-1.11**	0.02421
SRPRA	SRP receptor subunit alpha [Source:HGNC Symbol;Acc:HGNC:11307]	2268.7	2041.8	**-1.11**	0.02832
WDR46	WD repeat domain 46 [Source:HGNC Symbol;Acc:HGNC:13923]	1071.1	963.6	**-1.11**	0.02958
HMGN3	high mobility group nucleosomal binding domain 3 [Source:HGNC Symbol;Acc:HGNC:12312]	999.5	899.7	**-1.11**	0.03261
MRPL19	mitochondrial ribosomal protein L19 [Source:HGNC Symbol;Acc:HGNC:14052]	1075.0	971.5	**-1.11**	0.03314
ST6GALNAC6	ST6 N-acetylgalactosaminide alpha-2,6-sialyltransferase 6 [Source:HGNC Symbol;Acc:HGNC:23364]	841.2	763.1	**-1.11**	0.03431
KBTBD6	kelch repeat and BTB domain containing 6 [Source:HGNC Symbol;Acc:HGNC:25340]	711.4	643.2	**-1.11**	0.03997
MRPL24	mitochondrial ribosomal protein L24 [Source:HGNC Symbol;Acc:HGNC:14037]	1495.5	1348.5	**-1.11**	0.04053
WDR61	WD repeat domain 61 [Source:HGNC Symbol;Acc:HGNC:30300]	1094.0	989.2	**-1.11**	0.04067
DDX55	DEAD-box helicase 55 [Source:HGNC Symbol;Acc:HGNC:20085]	524.8	473.3	**-1.11**	0.04443
PRRC1	proline rich coiled-coil 1 [Source:HGNC Symbol;Acc:HGNC:28164]	2915.5	2613.8	**-1.12**	3.46E-05
DHX15	DEAH-box helicase 15 [Source:HGNC Symbol;Acc:HGNC:2738]	4648.2	4151.4	**-1.12**	9.58E-05
GABPA	GA binding protein transcription factor subunit alpha [Source:HGNC Symbol;Acc:HGNC:4071]	2140.9	1918.7	**-1.12**	0.0001096
ATE1	arginyltransferase 1 [Source:HGNC Symbol;Acc:HGNC:782]	1445.5	1296.5	**-1.12**	0.0001328
SUMO1	small ubiquitin-like modifier 1 [Source:HGNC Symbol;Acc:HGNC:12502]	2397.9	2139.9	**-1.12**	0.0001837
DSTN	destrin, actin depolymerizing factor [Source:HGNC Symbol;Acc:HGNC:15750]	27213.9	24360.2	**-1.12**	0.0002382
NGRN	neugrin, neurite outgrowth associated [Source:HGNC Symbol;Acc:HGNC:18077]	3374.3	3017.1	**-1.12**	0.0004445
KLF3	Kruppel like factor 3 [Source:HGNC Symbol;Acc:HGNC:16516]	2870.8	2582.3	**-1.12**	0.0006314
VEZT	vezatin, adherens junctions transmembrane protein [Source:HGNC Symbol;Acc:HGNC:18258]	2438.2	2175.7	**-1.12**	0.0006852
PDCL	phosducin like [Source:HGNC Symbol;Acc:HGNC:8770]	1311.2	1173.5	**-1.12**	0.0006933
ZCRB1	zinc finger CCHC-type and RNA binding motif containing 1 [Source:HGNC Symbol;Acc:HGNC:29620]	1784.6	1599.4	**-1.12**	0.000915
ZNF770	zinc finger protein 770 [Source:HGNC Symbol;Acc:HGNC:26061]	2930.4	2630.4	**-1.12**	0.001094
TSNAX	translin associated factor X [Source:HGNC Symbol;Acc:HGNC:12380]	1911.5	1710.8	**-1.12**	0.00119
MBTPS1	membrane bound transcription factor peptidase, site 1 [Source:HGNC Symbol;Acc:HGNC:15456]	4044.1	3612.7	**-1.12**	0.001278
CYCS	cytochrome c, somatic [Source:HGNC Symbol;Acc:HGNC:19986]	4224.9	3778.9	**-1.12**	0.001572
COX20	COX20, cytochrome c oxidase assembly factor [Source:HGNC Symbol;Acc:HGNC:26970]	2386.1	2135.5	**-1.12**	0.001605
KIFAP3	kinesin associated protein 3 [Source:HGNC Symbol;Acc:HGNC:17060]	2030.6	1813.1	**-1.12**	0.002037
GNAI2	G protein subunit alpha i2 [Source:HGNC Symbol;Acc:HGNC:4385]	4906.2	4381.5	**-1.12**	0.002374
NME7	NME/NM23 family member 7 [Source:HGNC Symbol;Acc:HGNC:20461]	2119.8	1912.5	**-1.12**	0.002735
BOD1	biorientation of chromosomes in cell division 1 [Source:HGNC Symbol;Acc:HGNC:25114]	886.9	793.2	**-1.12**	0.002931
SET	SET nuclear proto-oncogene [Source:HGNC Symbol;Acc:HGNC:10760]	15614.5	14036.7	**-1.12**	0.00351
SDE2	SDE2 telomere maintenance homolog [Source:HGNC Symbol;Acc:HGNC:26643]	1397.4	1249.4	**-1.12**	0.003785
NSF	N-ethylmaleimide sensitive factor, vesicle fusing ATPase [Source:HGNC Symbol;Acc:HGNC:8016]	1192.0	1063.9	**-1.12**	0.003836
AK2	adenylate kinase 2 [Source:HGNC Symbol;Acc:HGNC:362]	1921.1	1718.4	**-1.12**	0.004058
SCCPDH	saccharopine dehydrogenase (putative) [Source:HGNC Symbol;Acc:HGNC:24275]	1192.1	1063.8	**-1.12**	0.004291
PGM2	phosphoglucomutase 2 [Source:HGNC Symbol;Acc:HGNC:8906]	1701.8	1517.5	**-1.12**	0.005318
PSMA1	proteasome subunit alpha 1 [Source:HGNC Symbol;Acc:HGNC:9530]	2204.8	1973.8	**-1.12**	0.005636
GOLT1B	golgi transport 1B [Source:HGNC Symbol;Acc:HGNC:20175]	1500.5	1337.3	**-1.12**	0.005727

POLR3A	RNA polymerase III subunit A [Source:HGNC Symbol;Acc:HGNC:30074]	747.2	667.1	**-1.12**	0.006079
HNRNPAB	heterogeneous nuclear ribonucleoprotein A/B [Source:HGNC Symbol;Acc:HGNC:5034]	2106.2	1894.8	**-1.12**	0.006165
ERH	ERH, mRNA splicing and mitosis factor [Source:HGNC Symbol;Acc:HGNC:3447]	2341.0	2100.7	**-1.12**	0.007144
CDK17	cyclin dependent kinase 17 [Source:HGNC Symbol;Acc:HGNC:8750]	1853.1	1656.9	**-1.12**	0.008
QSER1	glutamine and serine rich 1 [Source:HGNC Symbol;Acc:HGNC:26154]	1091.1	978.5	**-1.12**	0.008093
SQSTM1	sequestosome 1 [Source:HGNC Symbol;Acc:HGNC:11280]	8786.6	7898.5	**-1.12**	0.008699
NDUFA6	NADH:ubiquinone oxidoreductase subunit A6 [Source:HGNC Symbol;Acc:HGNC:7690]	1541.3	1378.0	**-1.12**	0.00972
HNRNPR	heterogeneous nuclear ribonucleoprotein R [Source:HGNC Symbol;Acc:HGNC:5047]	6249.9	5642.7	**-1.12**	0.01094
INTS13	integrator complex subunit 13 [Source:HGNC Symbol;Acc:HGNC:20174]	1310.3	1174.8	**-1.12**	0.0131
RAD23A	RAD23 homolog A, nucleotide excision repair protein [Source:HGNC Symbol;Acc:HGNC:9812]	765.0	684.2	**-1.12**	0.01441
DCK	deoxycytidine kinase [Source:HGNC Symbol;Acc:HGNC:2704]	1206.9	1079.1	**-1.12**	0.01482
PIGB	phosphatidylinositol glycan anchor biosynthesis class B [Source:HGNC Symbol;Acc:HGNC:8959]	829.7	746.9	**-1.12**	0.01876
CBWD1	COBW domain containing 1 [Source:HGNC Symbol;Acc:HGNC:17134]	701.9	625.4	**-1.12**	0.01912
IFT172	intraflagellar transport 172 [Source:HGNC Symbol;Acc:HGNC:30391]	732.6	656.7	**-1.12**	0.02237
CRLF3	cytokine receptor like factor 3 [Source:HGNC Symbol;Acc:HGNC:17177]	686.3	613.8	**-1.12**	0.02296
ZNF286A	zinc finger protein 286A [Source:HGNC Symbol;Acc:HGNC:13501]	565.1	505.1	**-1.12**	0.02373
RPL26L1	ribosomal protein L26 like 1 [Source:HGNC Symbol;Acc:HGNC:17050]	574.2	513.7	**-1.12**	0.02688
RPP14	ribonuclease P/MRP subunit p14 [Source:HGNC Symbol;Acc:HGNC:30327]	599.6	536.9	**-1.12**	0.02691
NAB2	NGFI-A binding protein 2 [Source:HGNC Symbol;Acc:HGNC:7627]	1031.0	924.9	**-1.12**	0.02711
NDUFAB1	NADH:ubiquinone oxidoreductase subunit AB1 [Source:HGNC Symbol;Acc:HGNC:7694]	1392.6	1241.5	**-1.12**	0.02718
LSM5	LSM5 homolog, U6 small nuclear RNA and mRNA degradation associated [Source:HGNC Symbol;Acc:HG	945.2	850.0	**-1.12**	0.02742
SLC20A2	solute carrier family 20 member 2 [Source:HGNC Symbol;Acc:HGNC:10947]	634.1	569.2	**-1.12**	0.02831
ALG9	ALG9, alpha-1,2-mannosyltransferase [Source:HGNC Symbol;Acc:HGNC:15672]	905.0	811.3	**-1.12**	0.03
DDX41	DEAD-box helicase 41 [Source:HGNC Symbol;Acc:HGNC:18674]	549.8	491.4	**-1.12**	0.03385
CCL28	C-C motif chemokine ligand 28 [Source:HGNC Symbol;Acc:HGNC:17700]	338.3	302.9	**-1.12**	0.03993
TPM4	tropomyosin 4 [Source:HGNC Symbol;Acc:HGNC:12013]	9137.6	8319.3	**-1.12**	0.04252
PLEKHA8	pleckstrin homology domain containing A8 [Source:HGNC Symbol;Acc:HGNC:30037]	486.2	436.2	**-1.12**	0.04313
RMND1	required for meiotic nuclear division 1 homolog [Source:HGNC Symbol;Acc:HGNC:21176]	571.8	515.0	**-1.12**	0.04815
EID1	EP300 interacting inhibitor of differentiation 1 [Source:HGNC Symbol;Acc:HGNC:1191]	11117.0	9849.7	**-1.13**	1.01E-06
TRPM7	transient receptor potential cation channel subfamily M member 7 [Source:HGNC Symbol;Acc:HGNC:17	7419.1	6614.7	**-1.13**	1.04E-05
HDDC2	HD domain containing 2 [Source:HGNC Symbol;Acc:HGNC:21078]	2540.1	2258.6	**-1.13**	1.36E-05
DHX9	DExH-box helicase 9 [Source:HGNC Symbol;Acc:HGNC:2750]	5999.9	5325.7	**-1.13**	6.51E-05
ARHGAP5	Rho GTPase activating protein 5 [Source:HGNC Symbol;Acc:HGNC:675]	9015.0	7964.4	**-1.13**	7.62E-05
SRSF10	serine and arginine rich splicing factor 10 [Source:HGNC Symbol;Acc:HGNC:16713]	3040.2	2706.0	**-1.13**	0.0002828
UQCRC2	ubiquinol-cytochrome c reductase core protein 2 [Source:HGNC Symbol;Acc:HGNC:12586]	1785.5	1586.6	**-1.13**	0.0004532
SEPT9	septin 9 [Source:HGNC Symbol;Acc:HGNC:7323]	2185.3	1951.7	**-1.13**	0.0005047
NUDT3	nudix hydrolase 3 [Source:HGNC Symbol;Acc:HGNC:8050]	3385.5	3018.5	**-1.13**	0.0005206
YIPF2	Yip1 domain family member 2 [Source:HGNC Symbol;Acc:HGNC:28476]	1038.8	925.7	**-1.13**	0.0008651
PTRH2	peptidyl-tRNA hydrolase 2 [Source:HGNC Symbol;Acc:HGNC:24265]	1386.2	1231.3	**-1.13**	0.001077
TBCA	tubulin folding cofactor A [Source:HGNC Symbol;Acc:HGNC:11579]	1640.6	1453.7	**-1.13**	0.001238
SLF2	SMC5-SMC6 complex localization factor 2 [Source:HGNC Symbol;Acc:HGNC:17814]	1963.1	1749.6	**-1.13**	0.00146
LMBR1	limb development membrane protein 1 [Source:HGNC Symbol;Acc:HGNC:13243]	1397.0	1236.5	**-1.13**	0.001514
MAP3K20	mitogen-activated protein kinase kinase kinase 20 [Source:HGNC Symbol;Acc:HGNC:17797]	3185.2	2850.7	**-1.13**	0.001563
U2AF2	U2 small nuclear RNA auxiliary factor 2 [Source:HGNC Symbol;Acc:HGNC:23156]	1548.0	1378.2	**-1.13**	0.002445
UQCRQ	ubiquinol-cytochrome c reductase complex III subunit VII [Source:HGNC Symbol;Acc:HGNC:29594]	1168.7	1039.6	**-1.13**	0.002448
FSTL1	follistatin like 1 [Source:HGNC Symbol;Acc:HGNC:3972]	21788.0	19354.2	**-1.13**	0.002742
MTX2	metaxin 2 [Source:HGNC Symbol;Acc:HGNC:7506]	762.0	677.4	**-1.13**	0.002895
SERPINA1	serpin family A member 1 [Source:HGNC Symbol;Acc:HGNC:8941]	23454.7	20864.8	**-1.13**	0.00309
POLI	DNA polymerase iota [Source:HGNC Symbol;Acc:HGNC:9182]	1063.8	941.0	**-1.13**	0.003226
ARL5B	ADP ribosylation factor like GTPase 5B [Source:HGNC Symbol;Acc:HGNC:23052]	1033.2	919.4	**-1.13**	0.003773
UTP11	UTP11, small subunit processome component [Source:HGNC Symbol;Acc:HGNC:24329]	1003.4	896.4	**-1.13**	0.005419
CDC23	cell division cycle 23 [Source:HGNC Symbol;Acc:HGNC:1724]	787.1	703.9	**-1.13**	0.006178
KDM4A	lysine demethylase 4A [Source:HGNC Symbol;Acc:HGNC:22978]	759.3	672.6	**-1.13**	0.006479
CSE1L	chromosome segregation 1 like [Source:HGNC Symbol;Acc:HGNC:2431]	2995.1	2697.2	**-1.13**	0.006994
PIP4K2C	phosphatidylinositol-5-phosphate 4-kinase type 2 gamma [Source:HGNC Symbol;Acc:HGNC:23786]	786.3	698.3	**-1.13**	0.009927
PWWP2A	PWWP domain containing 2A [Source:HGNC Symbol;Acc:HGNC:29406]	548.3	488.3	**-1.13**	0.01067
FAM47E-STBD1	FAM47E-STBD1 readthrough [Source:HGNC Symbol;Acc:HGNC:44667]	999.4	887.9	**-1.13**	0.01088
ST3GAL1	ST3 beta-galactoside alpha-2,3-sialyltransferase 1 [Source:HGNC Symbol;Acc:HGNC:10862]	1673.5	1490.7	**-1.13**	0.01101
FAM210A	family with sequence similarity 210 member A [Source:HGNC Symbol;Acc:HGNC:28346]	674.4	598.1	**-1.13**	0.0126
H3F3A	H3 histone family member 3A [Source:HGNC Symbol;Acc:HGNC:4764]	759.6	675.1	**-1.13**	0.01309
POLR2A	RNA polymerase II subunit A [Source:HGNC Symbol;Acc:HGNC:9187]	620.5	549.9	**-1.13**	0.01378
MFSD8	major facilitator superfamily domain containing 8 [Source:HGNC Symbol;Acc:HGNC:28486]	657.5	584.0	**-1.13**	0.01453
STBD1	starch binding domain 1 [Source:HGNC Symbol;Acc:HGNC:24854]	973.9	863.4	**-1.13**	0.01488
ATP5MC3	ATP synthase membrane subunit c locus 3 [Source:HGNC Symbol;Acc:HGNC:843]	678.4	605.8	**-1.13**	0.01517
NUP155	nucleoporin 155 [Source:HGNC Symbol;Acc:HGNC:8063]	1587.6	1422.8	**-1.13**	0.01557
ZNF326	zinc finger protein 326 [Source:HGNC Symbol;Acc:HGNC:14104]	614.7	542.7	**-1.13**	0.01621
CHID1	chitinase domain containing 1 [Source:HGNC Symbol;Acc:HGNC:28474]	510.7	454.0	**-1.13**	0.01691
PEX10	peroxisomal biogenesis factor 10 [Source:HGNC Symbol;Acc:HGNC:8851]	582.7	515.8	**-1.13**	0.01739
TIMM21	translocase of inner mitochondrial membrane 21 [Source:HGNC Symbol;Acc:HGNC:25010]	797.7	709.6	**-1.13**	0.01765
WDR92	WD repeat domain 92 [Source:HGNC Symbol;Acc:HGNC:25176]	399.7	355.1	**-1.13**	0.02062
FGFR1OP	FGFR1 oncogene partner [Source:HGNC Symbol;Acc:HGNC:17012]	722.3	642.9	**-1.13**	0.02433
POLR2H	RNA polymerase II subunit H [Source:HGNC Symbol;Acc:HGNC:9195]	498.5	441.9	**-1.13**	0.02777

TPRKB	TP53RK binding protein [Source:HGNC Symbol;Acc:HGNC:24259]	536.3	478.4	**-1.13**	0.02789
PFDN2	prefoldin subunit 2 [Source:HGNC Symbol;Acc:HGNC:8867]	699.4	621.8	**-1.13**	0.02853
HNRNPH3	heterogeneous nuclear ribonucleoprotein H3 [Source:HGNC Symbol;Acc:HGNC:5043]	2027.3	1831.8	**-1.13**	0.02963
MACF1	microtubule-actin crosslinking factor 1 [Source:HGNC Symbol;Acc:HGNC:13664]	9783.6	8720.7	**-1.13**	0.03138
CNOT9	CCR4-NOT transcription complex subunit 9 [Source:HGNC Symbol;Acc:HGNC:10445]	1029.2	922.2	**-1.13**	0.0319
IL1RAP	interleukin 1 receptor accessory protein [Source:HGNC Symbol;Acc:HGNC:5995]	576.6	509.9	**-1.13**	0.03526
PPRC1	peroxisome proliferator-activated receptor gamma, coactivator-related 1 [Source:HGNC Symbol;Acc:HG	392.0	348.8	**-1.13**	0.03888
BORCS7	BLOC-1 related complex subunit 7 [Source:HGNC Symbol;Acc:HGNC:23516]	503.7	449.5	**-1.13**	0.04158
ZNF140	zinc finger protein 140 [Source:HGNC Symbol;Acc:HGNC:12925]	384.9	340.9	**-1.13**	0.04431
VARS2	valyl-tRNA synthetase 2, mitochondrial [Source:HGNC Symbol;Acc:HGNC:21642]	335.0	297.8	**-1.13**	0.04528
PRKAR1A	protein kinase cAMP-dependent type I regulatory subunit alpha [Source:HGNC Symbol;Acc:HGNC:9388]	19360.7	17118.6	**-1.13**	8.54E-08
TGFBR1	transforming growth factor beta receptor 1 [Source:HGNC Symbol;Acc:HGNC:11772]	6699.4	5916.2	**-1.13**	2.17E-06
PGRMC1	progesterone receptor membrane component 1 [Source:HGNC Symbol;Acc:HGNC:16090]	2828.7	2499.6	**-1.13**	3.58E-05
PCYOX1	prenylcysteine oxidase 1 [Source:HGNC Symbol;Acc:HGNC:20588]	4027.8	3550.7	**-1.13**	0.000143
TMED3	transmembrane p24 trafficking protein 3 [Source:HGNC Symbol;Acc:HGNC:28889]	2426.0	2138.5	**-1.13**	0.0001702
PPP2R5A	protein phosphatase 2 regulatory subunit B'alpha [Source:HGNC Symbol;Acc:HGNC:9309]	1465.2	1289.3	**-1.13**	0.0002844
HSPH1	heat shock protein family H (Hsp110) member 1 [Source:HGNC Symbol;Acc:HGNC:16969]	3080.8	2717.4	**-1.13**	0.0005768
AKT1	AKT serine/threonine kinase 1 [Source:HGNC Symbol;Acc:HGNC:391]	1925.8	1705.2	**-1.13**	0.0006227
KLHDC10	kelch domain containing 10 [Source:HGNC Symbol;Acc:HGNC:22194]	1968.6	1735.0	**-1.13**	0.0007144
TOMM22	translocase of outer mitochondrial membrane 22 [Source:HGNC Symbol;Acc:HGNC:18002]	1229.9	1080.8	**-1.13**	0.0009014
COPS4	COP9 signalosome subunit 4 [Source:HGNC Symbol;Acc:HGNC:16702]	1672.0	1472.5	**-1.13**	0.00112
ZACN	zinc activated ion channel [Source:HGNC Symbol;Acc:HGNC:29504]	751.7	663.0	**-1.13**	0.001729
GTF3A	general transcription factor IIIA [Source:HGNC Symbol;Acc:HGNC:4662]	2077.0	1827.9	**-1.13**	0.002022
MTHFD1	methylenetetrahydrofolate dehydrogenase, cyclohydrolase and formyltetrahydrofolate synthetase 1 [So	2423.2	2148.9	**-1.13**	0.002647
H2AFY	H2A histone family member Y [Source:HGNC Symbol;Acc:HGNC:4740]	1638.4	1450.6	**-1.13**	0.00403
SCAMP3	secretory carrier membrane protein 3 [Source:HGNC Symbol;Acc:HGNC:10565]	617.0	542.7	**-1.13**	0.004235
SLC35B3	solute carrier family 35 member B3 [Source:HGNC Symbol;Acc:HGNC:21601]	938.5	832.1	**-1.13**	0.00451
SPINT2	serine peptidase inhibitor, Kunitz type 2 [Source:HGNC Symbol;Acc:HGNC:11247]	1439.3	1272.3	**-1.13**	0.009437
RAB28	RAB28, member RAS oncogene family [Source:HGNC Symbol;Acc:HGNC:9768]	570.6	503.3	**-1.13**	0.009898
NDUFC1	NADH:ubiquinone oxidoreductase subunit C1 [Source:HGNC Symbol;Acc:HGNC:7705]	480.0	423.3	**-1.13**	0.01164
HS2ST1	heparan sulfate 2-O-sulfotransferase 1 [Source:HGNC Symbol;Acc:HGNC:5193]	1173.6	1033.4	**-1.13**	0.01214
TBC1D15	TBC1 domain family member 15 [Source:HGNC Symbol;Acc:HGNC:25694]	1827.7	1616.3	**-1.13**	0.01318
SPAG7	sperm associated antigen 7 [Source:HGNC Symbol;Acc:HGNC:11216]	578.1	510.8	**-1.13**	0.01587
B3GNT4	UDP-GlcNAc:betaGal beta-1,3-N-acetylglucosaminyltransferase 4 [Source:HGNC Symbol;Acc:HGNC:1568	424.4	375.1	**-1.13**	0.02088
NDUFB7	NADH:ubiquinone oxidoreductase subunit B7 [Source:HGNC Symbol;Acc:HGNC:7702]	490.0	432.8	**-1.13**	0.02521
ARHGEF1	Rho guanine nucleotide exchange factor 1 [Source:HGNC Symbol;Acc:HGNC:681]	1070.8	949.5	**-1.13**	0.02737
YEATS4	YEATS domain containing 4 [Source:HGNC Symbol;Acc:HGNC:24859]	455.9	403.5	**-1.13**	0.03177
FKBP7	FK506 binding protein 7 [Source:HGNC Symbol;Acc:HGNC:3723]	694.7	605.2	**-1.13**	0.03557
TFB2M	transcription factor B2, mitochondrial [Source:HGNC Symbol;Acc:HGNC:18559]	500.0	441.3	**-1.13**	0.03579
WDR83OS	WD repeat domain 83 opposite strand [Source:HGNC Symbol;Acc:HGNC:30203]	460.1	406.3	**-1.13**	0.03603
TVP23C-CDRT4	TVP23C-CDRT4 readthrough [Source:HGNC Symbol;Acc:HGNC:42961]	670.4	594.1	**-1.13**	0.03737
KANK1	KN motif and ankyrin repeat domains 1 [Source:HGNC Symbol;Acc:HGNC:19309]	5044.2	4464.6	**-1.13**	0.03753
ZNF445	zinc finger protein 445 [Source:HGNC Symbol;Acc:HGNC:21018]	471.7	416.8	**-1.13**	0.04147
TMEM135	transmembrane protein 135 [Source:HGNC Symbol;Acc:HGNC:26167]	487.1	427.4	**-1.13**	0.04912
KRT8	keratin 8 [Source:HGNC Symbol;Acc:HGNC:6446]	462.3	407.5	**-1.13**	0.04914
TERF1	telomeric repeat binding factor 1 [Source:HGNC Symbol;Acc:HGNC:11728]	626.8	554.7	**-1.13**	0.04936
RCN1	reticulocalbin 1 [Source:HGNC Symbol;Acc:HGNC:9934]	7302.9	6382.4	**-1.14**	9.33E-09
ZFP91	ZFP91 zinc finger protein [Source:HGNC Symbol;Acc:HGNC:14983]	4128.6	3612.2	**-1.14**	9.36E-08
BRD2	bromodomain containing 2 [Source:HGNC Symbol;Acc:HGNC:1103]	2890.0	2526.6	**-1.14**	2.64E-07
MAN1B1	mannosidase alpha class 1B member 1 [Source:HGNC Symbol;Acc:HGNC:6823]	1997.1	1753.4	**-1.14**	5.29E-07
CALM3	calmodulin 3 [Source:HGNC Symbol;Acc:HGNC:1449]	4612.9	4030.1	**-1.14**	1.81E-05
ALDH9A1	aldehyde dehydrogenase 9 family member A1 [Source:HGNC Symbol;Acc:HGNC:412]	1782.5	1567.2	**-1.14**	2.58E-05
GPC6	glypican 6 [Source:HGNC Symbol;Acc:HGNC:4454]	14580.2	12739.0	**-1.14**	4.69E-05
LASP1	LIM and SH3 protein 1 [Source:HGNC Symbol;Acc:HGNC:6513]	2392.0	2101.9	**-1.14**	6.27E-05
PELO	pelota mRNA surveillance and ribosome rescue factor [Source:HGNC Symbol;Acc:HGNC:8829]	1239.5	1088.7	**-1.14**	9.17E-05
HNRNPL	heterogeneous nuclear ribonucleoprotein L [Source:HGNC Symbol;Acc:HGNC:5045]	3078.9	2711.0	**-1.14**	0.0001298
BCAS2	BCAS2, pre-mRNA processing factor [Source:HGNC Symbol;Acc:HGNC:975]	2124.0	1863.5	**-1.14**	0.0001334
XPO1	exportin 1 [Source:HGNC Symbol;Acc:HGNC:12825]	7216.0	6346.3	**-1.14**	0.0001821
DNAJA3	DnaJ heat shock protein family (Hsp40) member A3 [Source:HGNC Symbol;Acc:HGNC:11808]	940.8	825.2	**-1.14**	0.0003572
PSMA4	proteasome subunit alpha 4 [Source:HGNC Symbol;Acc:HGNC:9533]	3414.2	3018.3	**-1.14**	0.0004823
GFPT1	glutamine--fructose-6-phosphate transaminase 1 [Source:HGNC Symbol;Acc:HGNC:4241]	14524.9	12728.2	**-1.14**	0.0005088
UQCC2	ubiquinol-cytochrome c reductase complex assembly factor 2 [Source:HGNC Symbol;Acc:HGNC:21237]	1084.3	952.7	**-1.14**	0.0008084
TRA2B	transformer 2 beta homolog [Source:HGNC Symbol;Acc:HGNC:10781]	3830.3	3380.7	**-1.14**	0.00112
LUZP1	leucine zipper protein 1 [Source:HGNC Symbol;Acc:HGNC:14985]	1253.4	1094.2	**-1.14**	0.001125
ASXL2	additional sex combs like 2, transcriptional regulator [Source:HGNC Symbol;Acc:HGNC:23805]	928.9	815.5	**-1.14**	0.001482
UQCC1	ubiquinol-cytochrome c reductase complex assembly factor 1 [Source:HGNC Symbol;Acc:HGNC:15891]	584.5	514.0	**-1.14**	0.002263
MRAS	muscle RAS oncogene homolog [Source:HGNC Symbol;Acc:HGNC:7227]	988.4	865.3	**-1.14**	0.002454
TMEM47	transmembrane protein 47 [Source:HGNC Symbol;Acc:HGNC:18515]	3605.9	3132.5	**-1.14**	0.002748
LRRCC1	leucine rich repeat and coiled-coil centrosomal protein 1 [Source:HGNC Symbol;Acc:HGNC:29373]	959.5	848.1	**-1.14**	0.002807
SLC39A10	solute carrier family 39 member 10 [Source:HGNC Symbol;Acc:HGNC:20861]	1000.8	880.8	**-1.14**	0.003486
ICMT	isoprenylcysteine carboxyl methyltransferase [Source:HGNC Symbol;Acc:HGNC:5350]	2556.0	2253.6	**-1.14**	0.00355
EEF1AKMT2	EEF1A lysine methyltransferase 2 [Source:HGNC Symbol;Acc:HGNC:33787]	747.1	651.1	**-1.14**	0.003739

SNRNP48	small nuclear ribonucleoprotein U11/U12 subunit 48 [Source:HGNC Symbol;Acc:HGNC:21368]	801.0	702.9	**-1.14**	0.004736
RCOR1	REST corepressor 1 [Source:HGNC Symbol;Acc:HGNC:17441]	545.3	477.2	**-1.14**	0.005415
SNRPD1	small nuclear ribonucleoprotein D1 polypeptide [Source:HGNC Symbol;Acc:HGNC:11158]	1736.3	1518.6	**-1.14**	0.00651
FH	fumarate hydratase [Source:HGNC Symbol;Acc:HGNC:3700]	1795.4	1572.0	**-1.14**	0.006904
BRIX1	BRX1, biogenesis of ribosomes [Source:HGNC Symbol;Acc:HGNC:24170]	1154.3	1005.7	**-1.14**	0.007627
PPP1R9B	protein phosphatase 1 regulatory subunit 9B [Source:HGNC Symbol;Acc:HGNC:9298]	602.6	530.8	**-1.14**	0.008212
BSDC1	BSD domain containing 1 [Source:HGNC Symbol;Acc:HGNC:25501]	1007.9	888.4	**-1.14**	0.008703
SIGMAR1	sigma non-opioid intracellular receptor 1 [Source:HGNC Symbol;Acc:HGNC:8157]	650.4	566.9	**-1.14**	0.009163
RAPH1	Ras association (RalGDS/AF-6) and pleckstrin homology domains 1 [Source:HGNC Symbol;Acc:HGNC:144	2990.9	2614.9	**-1.14**	0.009671
ARHGAP35	Rho GTPase activating protein 35 [Source:HGNC Symbol;Acc:HGNC:4591]	4734.4	4118.3	**-1.14**	0.01007
BMP1	bone morphogenetic protein 1 [Source:HGNC Symbol;Acc:HGNC:1067]	398.7	348.8	**-1.14**	0.01107
RFT1	RFT1 homolog [Source:HGNC Symbol;Acc:HGNC:30220]	578.7	505.8	**-1.14**	0.01114
SRA1	steroid receptor RNA activator 1 [Source:HGNC Symbol;Acc:HGNC:11281]	582.5	511.2	**-1.14**	0.01202
ZNF470	zinc finger protein 470 [Source:HGNC Symbol;Acc:HGNC:22220]	409.6	355.8	**-1.14**	0.01242
MBIP	MAP3K12 binding inhibitory protein 1 [Source:HGNC Symbol;Acc:HGNC:20427]	450.3	395.8	**-1.14**	0.01284
MRFAP1	Morf4 family associated protein 1 [Source:HGNC Symbol;Acc:HGNC:24549]	7348.3	6513.8	**-1.14**	0.01459
TMEM97	transmembrane protein 97 [Source:HGNC Symbol;Acc:HGNC:28106]	642.7	564.7	**-1.14**	0.01485
TMEM184B	transmembrane protein 184B [Source:HGNC Symbol;Acc:HGNC:1310]	548.8	482.1	**-1.14**	0.01758
SMURF2	SMAD specific E3 ubiquitin protein ligase 2 [Source:HGNC Symbol;Acc:HGNC:16809]	1064.5	931.7	**-1.14**	0.01807
GSKIP	GSK3B interacting protein [Source:HGNC Symbol;Acc:HGNC:20343]	573.3	504.5	**-1.14**	0.01871
SLBP	stem-loop binding protein [Source:HGNC Symbol;Acc:HGNC:10904]	1370.7	1210.7	**-1.14**	0.01961
POLR3F	RNA polymerase III subunit F [Source:HGNC Symbol;Acc:HGNC:15763]	551.4	483.7	**-1.14**	0.02382
NSUN3	NOP2/Sun RNA methyltransferase family member 3 [Source:HGNC Symbol;Acc:HGNC:26208]	364.3	318.6	**-1.14**	0.03237
ABCD4	ATP binding cassette subfamily D member 4 [Source:HGNC Symbol;Acc:HGNC:68]	364.7	318.1	**-1.14**	0.03453
RAB30	RAB30, member RAS oncogene family [Source:HGNC Symbol;Acc:HGNC:9770]	365.7	319.3	**-1.14**	0.04004
IFRD2	interferon related developmental regulator 2 [Source:HGNC Symbol;Acc:HGNC:5457]	385.9	339.4	**-1.14**	0.0417
VAV3	vav guanine nucleotide exchange factor 3 [Source:HGNC Symbol;Acc:HGNC:12659]	269.2	235.4	**-1.14**	0.04201
PRDM10	PR/SET domain 10 [Source:HGNC Symbol;Acc:HGNC:13995]	299.9	264.2	**-1.14**	0.04352
MRPL58	mitochondrial ribosomal protein L58 [Source:HGNC Symbol;Acc:HGNC:5359]	411.8	362.1	**-1.14**	0.04545
LYAR	Ly1 antibody reactive [Source:HGNC Symbol;Acc:HGNC:26021]	863.9	760.0	**-1.14**	0.04846
FKBP10	FK506 binding protein 10 [Source:HGNC Symbol;Acc:HGNC:18169]	5062.9	4417.6	**-1.15**	5.12E-09
EMC1	ER membrane protein complex subunit 1 [Source:HGNC Symbol;Acc:HGNC:28957]	3876.1	3389.9	**-1.15**	5.65E-07
CCDC91	coiled-coil domain containing 91 [Source:HGNC Symbol;Acc:HGNC:24855]	1082.9	939.1	**-1.15**	9.11E-06
SMIM15	small integral membrane protein 15 [Source:HGNC Symbol;Acc:HGNC:33861]	2255.5	1971.9	**-1.15**	1.44E-05
LZIC	leucine zipper and CTNNBIP1 domain containing [Source:HGNC Symbol;Acc:HGNC:17497]	1365.4	1190.5	**-1.15**	3.72E-05
COPS7A	COP9 signalosome subunit 7A [Source:HGNC Symbol;Acc:HGNC:16758]	1027.4	897.3	**-1.15**	4.07E-05
BBIP1	BBSome interacting protein 1 [Source:HGNC Symbol;Acc:HGNC:28093]	1238.0	1075.0	**-1.15**	4.32E-05
FAM3C	family with sequence similarity 3 member C [Source:HGNC Symbol;Acc:HGNC:18664]	5007.7	4386.7	**-1.15**	8.50E-05
PPP3CA	protein phosphatase 3 catalytic subunit alpha [Source:HGNC Symbol;Acc:HGNC:9314]	10019.1	8736.7	**-1.15**	0.0001645
SEPHS1	selenophosphate synthetase 1 [Source:HGNC Symbol;Acc:HGNC:19685]	1270.0	1104.7	**-1.15**	0.0002071
ADPGK	ADP dependent glucokinase [Source:HGNC Symbol;Acc:HGNC:25250]	1955.5	1700.8	**-1.15**	0.0002146
SLC41A2	solute carrier family 41 member 2 [Source:HGNC Symbol;Acc:HGNC:31045]	1086.1	941.8	**-1.15**	0.0002584
MYH10	myosin heavy chain 10 [Source:HGNC Symbol;Acc:HGNC:7568]	1183.2	1024.1	**-1.15**	0.0004653
CEBPG	CCAAT enhancer binding protein gamma [Source:HGNC Symbol;Acc:HGNC:1837]	1751.0	1517.2	**-1.15**	0.0007714
RABL3	RAB, member of RAS oncogene family like 3 [Source:HGNC Symbol;Acc:HGNC:18072]	746.2	650.4	**-1.15**	0.0008357
EPB41L3	erythrocyte membrane protein band 4.1 like 3 [Source:HGNC Symbol;Acc:HGNC:3380]	813.6	705.2	**-1.15**	0.0008578
TMX4	thioredoxin related transmembrane protein 4 [Source:HGNC Symbol;Acc:HGNC:25237]	7728.3	6717.0	**-1.15**	0.0009994
ISLR	immunoglobulin superfamily containing leucine rich repeat [Source:HGNC Symbol;Acc:HGNC:6133]	3490.2	3078.3	**-1.15**	0.00102
PCCB	propionyl-CoA carboxylase subunit beta [Source:HGNC Symbol;Acc:HGNC:8654]	857.3	746.8	**-1.15**	0.001037
SCRN3	secernin 3 [Source:HGNC Symbol;Acc:HGNC:30382]	717.0	626.9	**-1.15**	0.001045
CRYZ	crystallin zeta [Source:HGNC Symbol;Acc:HGNC:2419]	1881.5	1651.6	**-1.15**	0.001105
IKBIP	IKBKB interacting protein [Source:HGNC Symbol;Acc:HGNC:26430]	3667.8	3200.1	**-1.15**	0.001583
RBM18	RNA binding motif protein 18 [Source:HGNC Symbol;Acc:HGNC:28413]	895.8	781.0	**-1.15**	0.001619
TIA1	TIA1 cytotoxic granule associated RNA binding protein [Source:HGNC Symbol;Acc:HGNC:11802]	1080.5	940.8	**-1.15**	0.001999
CROT	carnitine O-octanoyltransferase [Source:HGNC Symbol;Acc:HGNC:2366]	690.3	598.9	**-1.15**	0.002218
MORC4	MORC family CW-type zinc finger 4 [Source:HGNC Symbol;Acc:HGNC:23485]	1131.8	983.5	**-1.15**	0.003012
AKAP2	A-kinase anchoring protein 2 [Source:HGNC Symbol;Acc:HGNC:372]	5251.9	4550.9	**-1.15**	0.003041
BSCL2	BSCL2, seipin lipid droplet biogenesis associated [Source:HGNC Symbol;Acc:HGNC:15832]	640.8	555.0	**-1.15**	0.003746
ZNF260	zinc finger protein 260 [Source:HGNC Symbol;Acc:HGNC:13499]	705.4	616.3	**-1.15**	0.003746
MBD3	methyl-CpG binding domain protein 3 [Source:HGNC Symbol;Acc:HGNC:6918]	541.7	469.4	**-1.15**	0.003812
INTS7	integrator complex subunit 7 [Source:HGNC Symbol;Acc:HGNC:24484]	552.1	482.0	**-1.15**	0.005385
TMEM267	transmembrane protein 267 [Source:HGNC Symbol;Acc:HGNC:26139]	739.1	645.7	**-1.15**	0.005414
NSMCE3	NSE3 homolog, SMC5-SMC6 complex component [Source:HGNC Symbol;Acc:HGNC:7677]	471.5	410.4	**-1.15**	0.007687
CARF	calcium responsive transcription factor [Source:HGNC Symbol;Acc:HGNC:14435]	432.2	377.0	**-1.15**	0.008902
RBBP8	RB binding protein 8, endonuclease [Source:HGNC Symbol;Acc:HGNC:9891]	1057.3	921.7	**-1.15**	0.01029
MICAL3	microtubule associated monooxygenase, calponin and LIM domain containing 3 [Source:HGNC Symbol;/	496.7	435.3	**-1.15**	0.011
DYRK4	dual specificity tyrosine phosphorylation regulated kinase 4 [Source:HGNC Symbol;Acc:HGNC:3095]	473.5	411.7	**-1.15**	0.01576
INO80E	INO80 complex subunit E [Source:HGNC Symbol;Acc:HGNC:26905]	464.4	405.5	**-1.15**	0.01709
CUEDC2	CUE domain containing 2 [Source:HGNC Symbol;Acc:HGNC:28352]	441.7	384.2	**-1.15**	0.01813
EPDR1	ependymin related 1 [Source:HGNC Symbol;Acc:HGNC:17572]	545.2	477.5	**-1.15**	0.01847
NDUFA8	NADH:ubiquinone oxidoreductase subunit A8 [Source:HGNC Symbol;Acc:HGNC:7692]	548.4	478.6	**-1.15**	0.0194
C5orf66	chromosome 5 open reading frame 66 [Source:HGNC Symbol;Acc:HGNC:48332]	300.8	261.3	**-1.15**	0.02048

DUSP7	dual specificity phosphatase 7 [Source:HGNC Symbol;Acc:HGNC:3073]	474.3	414.2	**-1.15**	0.02177
MCEE	methylmalonyl-CoA epimerase [Source:HGNC Symbol;Acc:HGNC:16732]	325.0	282.9	**-1.15**	0.02399
MRPL52	mitochondrial ribosomal protein L52 [Source:HGNC Symbol;Acc:HGNC:16655]	473.1	408.8	**-1.15**	0.02431
DBF4	DBF4 zinc finger [Source:HGNC Symbol;Acc:HGNC:17364]	395.6	345.0	**-1.15**	0.0275
NDUFAF5	NADH:ubiquinone oxidoreductase complex assembly factor 5 [Source:HGNC Symbol;Acc:HGNC:15899]	371.1	324.2	**-1.15**	0.02786
FN3KRP	fructosamine 3 kinase related protein [Source:HGNC Symbol;Acc:HGNC:25700]	345.3	300.4	**-1.15**	0.02795
MFHAS1	malignant fibrous histiocytoma amplified sequence 1 [Source:HGNC Symbol;Acc:HGNC:16982]	435.5	377.1	**-1.15**	0.02861
ZNF559	zinc finger protein 559 [Source:HGNC Symbol;Acc:HGNC:28197]	362.8	316.7	**-1.15**	0.04262
SPPL3	signal peptide peptidase like 3 [Source:HGNC Symbol;Acc:HGNC:30424]	284.6	247.8	**-1.15**	0.04275
ZNF568	zinc finger protein 568 [Source:HGNC Symbol;Acc:HGNC:25392]	364.1	316.6	**-1.15**	0.04765
EMC2	ER membrane protein complex subunit 2 [Source:HGNC Symbol;Acc:HGNC:28963]	2039.6	1756.7	**-1.16**	7.30E-07
SOCS5	suppressor of cytokine signaling 5 [Source:HGNC Symbol;Acc:HGNC:16852]	2639.7	2280.7	**-1.16**	7.55E-07
PTEN	phosphatase and tensin homolog [Source:HGNC Symbol;Acc:HGNC:9588]	4242.8	3648.0	**-1.16**	1.74E-06
OAT	ornithine aminotransferase [Source:HGNC Symbol;Acc:HGNC:8091]	5339.6	4603.0	**-1.16**	4.66E-06
CCDC90B	coiled-coil domain containing 90B [Source:HGNC Symbol;Acc:HGNC:28108]	2587.8	2244.8	**-1.16**	6.03E-06
RAPGEF2	Rap guanine nucleotide exchange factor 2 [Source:HGNC Symbol;Acc:HGNC:16854]	2371.6	2044.8	**-1.16**	8.30E-06
ADAM9	ADAM metallopeptidase domain 9 [Source:HGNC Symbol;Acc:HGNC:216]	3829.7	3316.1	**-1.16**	9.98E-06
SYNJ2BP	synaptojanin 2 binding protein [Source:HGNC Symbol;Acc:HGNC:18955]	2518.2	2175.4	**-1.16**	1.14E-05
CALU	calumenin [Source:HGNC Symbol;Acc:HGNC:1458]	26287.9	22762.4	**-1.16**	1.78E-05
CAB39	calcium binding protein 39 [Source:HGNC Symbol;Acc:HGNC:20292]	7391.6	6358.9	**-1.16**	2.58E-05
GLOD4	glyoxalase domain containing 4 [Source:HGNC Symbol;Acc:HGNC:14111]	1421.0	1229.9	**-1.16**	5.43E-05
CBX3	chromobox 3 [Source:HGNC Symbol;Acc:HGNC:1553]	4333.1	3747.0	**-1.16**	5.92E-05
SARS	seryl-tRNA synthetase [Source:HGNC Symbol;Acc:HGNC:10537]	7018.4	6054.9	**-1.16**	6.34E-05
CYB5B	cytochrome b5 type B [Source:HGNC Symbol;Acc:HGNC:24374]	2358.1	2031.8	**-1.16**	6.71E-05
SEC13	SEC13 homolog, nuclear pore and COPII coat complex component [Source:HGNC Symbol;Acc:HGNC:106	2964.9	2568.0	**-1.16**	0.0001175
TRAPPC13	trafficking protein particle complex 13 [Source:HGNC Symbol;Acc:HGNC:25828]	792.8	684.7	**-1.16**	0.0001469
BMPR1A	bone morphogenetic protein receptor type 1A [Source:HGNC Symbol;Acc:HGNC:1076]	1671.8	1453.4	**-1.16**	0.0001524
SLC12A4	solute carrier family 12 member 4 [Source:HGNC Symbol;Acc:HGNC:10913]	1009.5	870.9	**-1.16**	0.000251
CBWD2	COBW domain containing 2 [Source:HGNC Symbol;Acc:HGNC:17907]	795.3	683.6	**-1.16**	0.0005729
S100A1	S100 calcium binding protein A1 [Source:HGNC Symbol;Acc:HGNC:10486]	4017.2	3559.2	**-1.16**	0.0006215
LIX1L-AS1	LIX1L antisense RNA 1 [Source:HGNC Symbol;Acc:HGNC:41210]	1118.0	969.8	**-1.16**	0.0007411
DIP2C	disco interacting protein 2 homolog C [Source:HGNC Symbol;Acc:HGNC:29150]	1185.7	1028.7	**-1.16**	0.001052
UROD	uroporphyrinogen decarboxylase [Source:HGNC Symbol;Acc:HGNC:12591]	701.5	606.6	**-1.16**	0.001264
BLOC1S2	biogenesis of lysosomal organelles complex 1 subunit 2 [Source:HGNC Symbol;Acc:HGNC:20984]	1028.2	883.6	**-1.16**	0.001945
PSMD14	proteasome 26S subunit, non-ATPase 14 [Source:HGNC Symbol;Acc:HGNC:16889]	2969.5	2587.2	**-1.16**	0.002416
FARSA	phenylalanyl-tRNA synthetase subunit alpha [Source:HGNC Symbol;Acc:HGNC:3592]	558.0	483.6	**-1.16**	0.002511
ZNF626	zinc finger protein 626 [Source:HGNC Symbol;Acc:HGNC:30461]	528.9	459.4	**-1.16**	0.002957
PLA2G12A	phospholipase A2 group XIIA [Source:HGNC Symbol;Acc:HGNC:18554]	682.0	587.5	**-1.16**	0.003589
PAAF1	proteasomal ATPase associated factor 1 [Source:HGNC Symbol;Acc:HGNC:25687]	358.8	309.5	**-1.16**	0.003787
SAYSD1	SAYSVFN motif domain containing 1 [Source:HGNC Symbol;Acc:HGNC:21025]	596.8	516.5	**-1.16**	0.004251
PEX5	peroxisomal biogenesis factor 5 [Source:HGNC Symbol;Acc:HGNC:9719]	532.3	462.3	**-1.16**	0.005531
GRK2	G protein-coupled receptor kinase 2 [Source:HGNC Symbol;Acc:HGNC:289]	445.0	382.6	**-1.16**	0.005602
TPBG	trophoblast glycoprotein [Source:HGNC Symbol;Acc:HGNC:12004]	675.6	586.9	**-1.16**	0.007314
SH3KBP1	SH3 domain containing kinase binding protein 1 [Source:HGNC Symbol;Acc:HGNC:13867]	793.8	681.8	**-1.16**	0.008517
WDR12	WD repeat domain 12 [Source:HGNC Symbol;Acc:HGNC:14098]	1182.8	1018.3	**-1.16**	0.008519
SCYL3	SCY1 like pseudokinase 3 [Source:HGNC Symbol;Acc:HGNC:19285]	542.6	469.6	**-1.16**	0.009207
DGCR8	DGCR8, microprocessor complex subunit [Source:HGNC Symbol;Acc:HGNC:2847]	676.3	588.4	**-1.16**	0.009213
RANBP3	RAN binding protein 3 [Source:HGNC Symbol;Acc:HGNC:9850]	409.3	353.2	**-1.16**	0.01187
ZNF569	zinc finger protein 569 [Source:HGNC Symbol;Acc:HGNC:24737]	313.7	271.0	**-1.16**	0.01195
PSME1	proteasome activator subunit 1 [Source:HGNC Symbol;Acc:HGNC:9568]	396.6	343.7	**-1.16**	0.0149
HIRIP3	HIRA interacting protein 3 [Source:HGNC Symbol;Acc:HGNC:4917]	354.4	307.9	**-1.16**	0.015
GGCT	gamma-glutamylcyclotransferase [Source:HGNC Symbol;Acc:HGNC:21705]	690.6	602.5	**-1.16**	0.01525
CGREF1	cell growth regulator with EF-hand domain 1 [Source:HGNC Symbol;Acc:HGNC:16962]	417.6	362.0	**-1.16**	0.01825
PIAS4	protein inhibitor of activated STAT 4 [Source:HGNC Symbol;Acc:HGNC:17002]	243.7	211.5	**-1.16**	0.01835
GNAL	G protein subunit alpha L [Source:HGNC Symbol;Acc:HGNC:4388]	371.8	322.4	**-1.16**	0.01958
FNBP1L	formin binding protein 1 like [Source:HGNC Symbol;Acc:HGNC:20851]	460.5	393.8	**-1.16**	0.01974
ZFAND2B	zinc finger AN1-type containing 2B [Source:HGNC Symbol;Acc:HGNC:25206]	331.7	285.8	**-1.16**	0.0207
HDAC4	histone deacetylase 4 [Source:HGNC Symbol;Acc:HGNC:14063]	497.0	427.6	**-1.16**	0.02276
NFATC1	nuclear factor of activated T cells 1 [Source:HGNC Symbol;Acc:HGNC:7775]	287.2	248.7	**-1.16**	0.02523
C10orf88	chromosome 10 open reading frame 88 [Source:HGNC Symbol;Acc:HGNC:25822]	249.5	215.1	**-1.16**	0.02586
HOMER3	homer scaffold protein 3 [Source:HGNC Symbol;Acc:HGNC:17514]	344.4	297.2	**-1.16**	0.02696
ZNF625	zinc finger protein 625 [Source:HGNC Symbol;Acc:HGNC:30571]	250.4	215.8	**-1.16**	0.02786
ING1	inhibitor of growth family member 1 [Source:HGNC Symbol;Acc:HGNC:6062]	365.6	315.1	**-1.16**	0.02857
NAGLU	N-acetyl-alpha-glucosaminidase [Source:HGNC Symbol;Acc:HGNC:7632]	316.5	275.5	**-1.16**	0.04498
DOLPP1	dolichyldiphosphatase 1 [Source:HGNC Symbol;Acc:HGNC:29565]	171.2	148.1	**-1.16**	0.04912
TXNL1	thioredoxin like 1 [Source:HGNC Symbol;Acc:HGNC:12436]	2393.5	2058.3	**-1.16**	3.22E-09
SUCO	SUN domain containing ossification factor [Source:HGNC Symbol;Acc:HGNC:1240]	4257.2	3637.9	**-1.16**	8.32E-09
LRP6	LDL receptor related protein 6 [Source:HGNC Symbol;Acc:HGNC:6698]	5407.0	4639.6	**-1.16**	3.22E-07
CEP63	centrosomal protein 63 [Source:HGNC Symbol;Acc:HGNC:25815]	2623.5	2248.3	**-1.16**	8.47E-07
GOLM1	golgi membrane protein 1 [Source:HGNC Symbol;Acc:HGNC:15451]	19701.1	16915.2	**-1.16**	9.97E-07
SNX5	sorting nexin 5 [Source:HGNC Symbol;Acc:HGNC:14969]	2358.4	2030.5	**-1.16**	1.64E-06
DENR	density regulated re-initiation and release factor [Source:HGNC Symbol;Acc:HGNC:2769]	2217.8	1911.2	**-1.16**	1.05E-05

VOPP1	VOPP1, WBP1/VOPP1 family member [Source:HGNC Symbol;Acc:HGNC:34518]	1777.9	1523.1	**-1.16**	2.17E-05
ANP32E	acidic nuclear phosphoprotein 32 family member E [Source:HGNC Symbol;Acc:HGNC:16673]	3003.0	2594.2	**-1.16**	3.09E-05
CKAP4	cytoskeleton associated protein 4 [Source:HGNC Symbol;Acc:HGNC:16991]	4041.4	3461.2	**-1.16**	3.39E-05
NAGK	N-acetylglucosamine kinase [Source:HGNC Symbol;Acc:HGNC:17174]	1244.7	1066.4	**-1.16**	0.0001088
PRDX3	peroxiredoxin 3 [Source:HGNC Symbol;Acc:HGNC:9354]	2621.5	2256.0	**-1.16**	0.0001372
IGFBP7	insulin like growth factor binding protein 7 [Source:HGNC Symbol;Acc:HGNC:5476]	1597.8	1361.9	**-1.16**	0.0006958
PALM2-AKAP2	PALM2-AKAP2 readthrough [Source:HGNC Symbol;Acc:HGNC:33529]	5458.9	4671.1	**-1.16**	0.0007012
BEX3	brain expressed X-linked 3 [Source:HGNC Symbol;Acc:HGNC:13388]	1032.6	891.6	**-1.16**	0.0008138
VARS	valyl-tRNA synthetase [Source:HGNC Symbol;Acc:HGNC:12651]	570.7	489.1	**-1.16**	0.0009397
HSDL1	hydroxysteroid dehydrogenase like 1 [Source:HGNC Symbol;Acc:HGNC:16475]	717.6	615.2	**-1.16**	0.000961
RXYLT1	ribitol xylosyltransferase 1 [Source:HGNC Symbol;Acc:HGNC:13530]	958.7	819.4	**-1.16**	0.001034
ALYREF	Aly/REF export factor [Source:HGNC Symbol;Acc:HGNC:19071]	1147.5	989.0	**-1.16**	0.001325
FAN1	FANCD2 and FANCI associated nuclease 1 [Source:HGNC Symbol;Acc:HGNC:29170]	1221.5	1054.0	**-1.16**	0.001392
CCP110	centriolar coiled-coil protein 110 [Source:HGNC Symbol;Acc:HGNC:24342]	815.5	700.0	**-1.16**	0.00191
TAF6	TATA-box binding protein associated factor 6 [Source:HGNC Symbol;Acc:HGNC:11540]	440.3	377.2	**-1.16**	0.002363
HMGXB4	HMG-box containing 4 [Source:HGNC Symbol;Acc:HGNC:5003]	596.5	511.6	**-1.16**	0.002732
ARL6	ADP ribosylation factor like GTPase 6 [Source:HGNC Symbol;Acc:HGNC:13210]	573.2	490.2	**-1.16**	0.003565
KIAA0930	KIAA0930 [Source:HGNC Symbol;Acc:HGNC:1314]	591.0	508.9	**-1.16**	0.003579
MLF1	myeloid leukemia factor 1 [Source:HGNC Symbol;Acc:HGNC:7125]	343.3	295.5	**-1.16**	0.004543
CAVIN3	caveolae associated protein 3 [Source:HGNC Symbol;Acc:HGNC:9400]	484.8	417.1	**-1.16**	0.004602
ZNF484	zinc finger protein 484 [Source:HGNC Symbol;Acc:HGNC:23385]	580.0	501.5	**-1.16**	0.007454
INO80B-WBP1	INO80B-WBP1 readthrough (NMD candidate) [Source:HGNC Symbol;Acc:HGNC:49199]	512.0	441.2	**-1.16**	0.009898
C17orf75	chromosome 17 open reading frame 75 [Source:HGNC Symbol;Acc:HGNC:30173]	450.1	384.7	**-1.16**	0.01141
NFYA	nuclear transcription factor Y subunit alpha [Source:HGNC Symbol;Acc:HGNC:7804]	795.1	681.4	**-1.16**	0.01211
ZNF542P	zinc finger protein 542, pseudogene [Source:HGNC Symbol;Acc:HGNC:25393]	421.3	360.4	**-1.16**	0.01349
NDNF	neuron derived neurotrophic factor [Source:HGNC Symbol;Acc:HGNC:26256]	1118.8	983.4	**-1.16**	0.01421
AC093323.1	Putative MORF4 family-associated protein 1-like protein UPP [Source:UniProtKB/Swiss-Prot;Acc:B2RBV	320.1	275.1	**-1.16**	0.01685
FAM57A	family with sequence similarity 57 member A [Source:HGNC Symbol;Acc:HGNC:29646]	290.2	248.2	**-1.16**	0.02308
ZNF736	zinc finger protein 736 [Source:HGNC Symbol;Acc:HGNC:32467]	295.4	255.9	**-1.16**	0.02348
HSCB	HscB mitochondrial iron-sulfur cluster cochaperone [Source:HGNC Symbol;Acc:HGNC:28913]	236.1	203.7	**-1.16**	0.03032
HPF1	histone PARylation factor 1 [Source:HGNC Symbol;Acc:HGNC:26051]	464.1	399.5	**-1.16**	0.03089
PACS1	phosphofurin acidic cluster sorting protein 1 [Source:HGNC Symbol;Acc:HGNC:30032]	285.9	245.7	**-1.16**	0.03112
PPIF	peptidylprolyl isomerase F [Source:HGNC Symbol;Acc:HGNC:9259]	347.4	298.4	**-1.16**	0.03117
ZNF486	zinc finger protein 486 [Source:HGNC Symbol;Acc:HGNC:20807]	280.5	240.6	**-1.16**	0.0315
KREMEN1	kringle containing transmembrane protein 1 [Source:HGNC Symbol;Acc:HGNC:17550]	263.8	222.8	**-1.16**	0.03282
RAD52	RAD52 homolog, DNA repair protein [Source:HGNC Symbol;Acc:HGNC:9824]	247.6	210.6	**-1.16**	0.04127
LMF2	lipase maturation factor 2 [Source:HGNC Symbol;Acc:HGNC:25096]	271.1	232.7	**-1.16**	0.04157
RASSF1	Ras association domain family member 1 [Source:HGNC Symbol;Acc:HGNC:9882]	397.0	339.9	**-1.16**	0.04484
SAE1	SUMO1 activating enzyme subunit 1 [Source:HGNC Symbol;Acc:HGNC:30660]	2450.2	2090.9	**-1.17**	1.89E-09
HTATSF1	HIV-1 Tat specific factor 1 [Source:HGNC Symbol;Acc:HGNC:5276]	3491.3	2981.4	**-1.17**	1.73E-08
IMPAD1	inositol monophosphatase domain containing 1 [Source:HGNC Symbol;Acc:HGNC:26019]	9864.1	8389.9	**-1.17**	7.44E-08
GPR107	G protein-coupled receptor 107 [Source:HGNC Symbol;Acc:HGNC:17830]	3036.8	2598.4	**-1.17**	1.24E-07
HDAC2	histone deacetylase 2 [Source:HGNC Symbol;Acc:HGNC:4853]	1702.9	1459.8	**-1.17**	2.67E-07
YIPF6	Yip1 domain family member 6 [Source:HGNC Symbol;Acc:HGNC:28304]	2317.8	1973.0	**-1.17**	3.60E-07
MDH1	malate dehydrogenase 1 [Source:HGNC Symbol;Acc:HGNC:6970]	2182.5	1853.9	**-1.17**	8.49E-07
ERLIN2	ER lipid raft associated 2 [Source:HGNC Symbol;Acc:HGNC:1356]	3001.4	2552.5	**-1.17**	8.90E-07
MBNL2	muscleblind like splicing regulator 2 [Source:HGNC Symbol;Acc:HGNC:16746]	2882.3	2462.8	**-1.17**	1.91E-06
GAS5	growth arrest specific 5 (non-protein coding) [Source:HGNC Symbol;Acc:HGNC:16355]	1171.8	995.0	**-1.17**	2.31E-06
MRPS10	mitochondrial ribosomal protein S10 [Source:HGNC Symbol;Acc:HGNC:14502]	1066.8	908.7	**-1.17**	2.44E-06
ERCC6L2	ERCC excision repair 6 like 2 [Source:HGNC Symbol;Acc:HGNC:26922]	1132.2	967.2	**-1.17**	7.18E-06
TAPBP	TAP binding protein [Source:HGNC Symbol;Acc:HGNC:11566]	1234.8	1048.5	**-1.17**	1.68E-05
MTX3	metaxin 3 [Source:HGNC Symbol;Acc:HGNC:24812]	1569.9	1339.8	**-1.17**	2.43E-05
MOB4	MOB family member 4, phocein [Source:HGNC Symbol;Acc:HGNC:17261]	1689.3	1436.3	**-1.17**	3.28E-05
SETBP1	SET binding protein 1 [Source:HGNC Symbol;Acc:HGNC:15573]	920.0	785.9	**-1.17**	4.93E-05
GPR180	G protein-coupled receptor 180 [Source:HGNC Symbol;Acc:HGNC:28899]	1732.7	1480.0	**-1.17**	6.89E-05
SNURF	SNRPN upstream reading frame [Source:HGNC Symbol;Acc:HGNC:11171]	1258.1	1076.3	**-1.17**	7.22E-05
GNPDA1	glucosamine-6-phosphate deaminase 1 [Source:HGNC Symbol;Acc:HGNC:4417]	1138.3	971.2	**-1.17**	0.0001009
RPAIN	RPA interacting protein [Source:HGNC Symbol;Acc:HGNC:28641]	603.9	514.3	**-1.17**	0.0001372
NUP107	nucleoporin 107 [Source:HGNC Symbol;Acc:HGNC:29914]	1605.7	1382.5	**-1.17**	0.0001699
TCEAL8	transcription elongation factor A like 8 [Source:HGNC Symbol;Acc:HGNC:28683]	2579.5	2194.0	**-1.17**	0.0001777
TRIQK	triple QxxK/R motif containing [Source:HGNC Symbol;Acc:HGNC:27828]	1441.1	1227.5	**-1.17**	0.0001952
EDIL3	EGF like repeats and discoidin domains 3 [Source:HGNC Symbol;Acc:HGNC:3173]	24686.4	21062.2	**-1.17**	0.0002334
KLF10	Kruppel like factor 10 [Source:HGNC Symbol;Acc:HGNC:11810]	1446.5	1237.6	**-1.17**	0.0002388
MFAP3L	microfibril associated protein 3 like [Source:HGNC Symbol;Acc:HGNC:29083]	1046.9	893.0	**-1.17**	0.0002505
KMT5A	lysine methyltransferase 5A [Source:HGNC Symbol;Acc:HGNC:29489]	1146.1	980.5	**-1.17**	0.0002532
PAF1	PAF1 homolog, Paf1/RNA polymerase II complex component [Source:HGNC Symbol;Acc:HGNC:25459]	1195.4	1018.8	**-1.17**	0.0003156
ABI2	abl interactor 2 [Source:HGNC Symbol;Acc:HGNC:24011]	3425.2	2917.4	**-1.17**	0.0003515
NOP56	NOP56 ribonucleoprotein [Source:HGNC Symbol;Acc:HGNC:15911]	2280.5	1932.4	**-1.17**	0.0003898
SLC25A13	solute carrier family 25 member 13 [Source:HGNC Symbol;Acc:HGNC:10983]	782.7	666.4	**-1.17**	0.0004506
PARP1	poly(ADP-ribose) polymerase 1 [Source:HGNC Symbol;Acc:HGNC:270]	2323.5	1990.6	**-1.17**	0.0004706
TUBB4B	tubulin beta 4B class IVb [Source:HGNC Symbol;Acc:HGNC:20771]	3653.0	3151.3	**-1.17**	0.0005269
GLI3	GLI family zinc finger 3 [Source:HGNC Symbol;Acc:HGNC:4319]	654.7	554.4	**-1.17**	0.0005755

DLAT	dihydrolipoamide S-acetyltransferase [Source:HGNC Symbol;Acc:HGNC:2896]	1269.8	1081.1	**-1.17**	0.0006194
SERTAD2	SERTA domain containing 2 [Source:HGNC Symbol;Acc:HGNC:30784]	1478.2	1266.6	**-1.17**	0.0007021
TMA16	translation machinery associated 16 homolog [Source:HGNC Symbol;Acc:HGNC:25638]	839.8	716.9	**-1.17**	0.0007745
LSM4	LSM4 homolog, U6 small nuclear RNA and mRNA degradation associated [Source:HGNC Symbol;Acc:HG	825.1	706.4	**-1.17**	0.001213
TRA2A	transformer 2 alpha homolog [Source:HGNC Symbol;Acc:HGNC:16645]	735.6	632.7	**-1.17**	0.001457
COMMD2	COMM domain containing 2 [Source:HGNC Symbol;Acc:HGNC:24993]	1248.5	1063.1	**-1.17**	0.001523
SAP30	Sin3A associated protein 30 [Source:HGNC Symbol;Acc:HGNC:10532]	841.7	719.5	**-1.17**	0.001655
FAM76A	family with sequence similarity 76 member A [Source:HGNC Symbol;Acc:HGNC:28530]	491.9	418.3	**-1.17**	0.001714
ZNRF2	zinc and ring finger 2 [Source:HGNC Symbol;Acc:HGNC:22316]	397.1	338.9	**-1.17**	0.002425
TMEM116	transmembrane protein 116 [Source:HGNC Symbol;Acc:HGNC:25084]	399.8	342.2	**-1.17**	0.002546
PRPSAP1	phosphoribosyl pyrophosphate synthetase associated protein 1 [Source:HGNC Symbol;Acc:HGNC:9466]	634.8	542.0	**-1.17**	0.002864
DHCR7	7-dehydrocholesterol reductase [Source:HGNC Symbol;Acc:HGNC:2860]	690.1	587.8	**-1.17**	0.002986
POLD2	DNA polymerase delta 2, accessory subunit [Source:HGNC Symbol;Acc:HGNC:9176]	872.9	744.7	**-1.17**	0.003106
MRPL43	mitochondrial ribosomal protein L43 [Source:HGNC Symbol;Acc:HGNC:14517]	357.6	304.1	**-1.17**	0.003926
LMAN2L	lectin, mannose binding 2 like [Source:HGNC Symbol;Acc:HGNC:19263]	686.0	586.1	**-1.17**	0.004116
GPNMB	glycoprotein nmb [Source:HGNC Symbol;Acc:HGNC:4462]	704.9	629.2	**-1.17**	0.004463
MRPS34	mitochondrial ribosomal protein S34 [Source:HGNC Symbol;Acc:HGNC:16618]	463.9	395.7	**-1.17**	0.00509
RNASEK-C17orf49	RNASEK-C17orf49 readthrough [Source:HGNC Symbol;Acc:HGNC:44419]	565.9	486.0	**-1.17**	0.007182
SH3BP5-AS1	SH3BP5 antisense RNA 1 [Source:HGNC Symbol;Acc:HGNC:44501]	1572.2	1325.6	**-1.17**	0.007268
SELENOW	selenoprotein W [Source:HGNC Symbol;Acc:HGNC:10752]	431.2	366.7	**-1.17**	0.008714
SNAP47	synaptosome associated protein 47 [Source:HGNC Symbol;Acc:HGNC:30669]	590.4	501.6	**-1.17**	0.01021
NUPR1	nuclear protein 1, transcriptional regulator [Source:HGNC Symbol;Acc:HGNC:29990]	2602.8	2227.4	**-1.17**	0.01021
TMEM128	transmembrane protein 128 [Source:HGNC Symbol;Acc:HGNC:28201]	427.1	361.7	**-1.17**	0.01088
MPZL2	myelin protein zero like 2 [Source:HGNC Symbol;Acc:HGNC:3496]	747.7	650.2	**-1.17**	0.01147
NAV1	neuron navigator 1 [Source:HGNC Symbol;Acc:HGNC:15989]	1644.9	1405.6	**-1.17**	0.01294
TRAPPC2L	trafficking protein particle complex 2 like [Source:HGNC Symbol;Acc:HGNC:30887]	317.7	269.3	**-1.17**	0.01383
SFXN5	sideroflexin 5 [Source:HGNC Symbol;Acc:HGNC:16073]	200.4	169.9	**-1.17**	0.01607
RILPL1	Rab interacting lysosomal protein like 1 [Source:HGNC Symbol;Acc:HGNC:26814]	445.9	380.1	**-1.17**	0.01652
RGS12	regulator of G protein signaling 12 [Source:HGNC Symbol;Acc:HGNC:9994]	224.6	190.5	**-1.17**	0.01674
MITF	melanogenesis associated transcription factor [Source:HGNC Symbol;Acc:HGNC:7105]	344.1	296.1	**-1.17**	0.01707
PDIK1L	PDLIM1 interacting kinase 1 like [Source:HGNC Symbol;Acc:HGNC:18981]	316.2	268.8	**-1.17**	0.03002
LRSAM1	leucine rich repeat and sterile alpha motif containing 1 [Source:HGNC Symbol;Acc:HGNC:25135]	266.8	226.0	**-1.17**	0.03078
TRIM21	tripartite motif containing 21 [Source:HGNC Symbol;Acc:HGNC:11312]	268.8	229.3	**-1.17**	0.03418
LINC00662	long intergenic non-protein coding RNA 662 [Source:HGNC Symbol;Acc:HGNC:27122]	340.3	288.8	**-1.17**	0.04104
METTL21A	methyltransferase like 21A [Source:HGNC Symbol;Acc:HGNC:30476]	214.1	182.1	**-1.17**	0.04325
USP30	ubiquitin specific peptidase 30 [Source:HGNC Symbol;Acc:HGNC:20065]	178.4	153.3	**-1.17**	0.04328
HKR1	HKR1, GLI-Kruppel zinc finger family member [Source:HGNC Symbol;Acc:HGNC:4928]	313.5	267.3	**-1.17**	0.04381
PTPN20	protein tyrosine phosphatase, non-receptor type 20 [Source:HGNC Symbol;Acc:HGNC:23423]	223.4	189.7	**-1.17**	0.0465
PSMD11	proteasome 26S subunit, non-ATPase 11 [Source:HGNC Symbol;Acc:HGNC:9556]	2620.9	2221.8	**-1.18**	2.61E-10
AHCYL1	adenosylhomocysteinase like 1 [Source:HGNC Symbol;Acc:HGNC:344]	1925.3	1626.4	**-1.18**	2.74E-08
TPD52L1	tumor protein D52 like 1 [Source:HGNC Symbol;Acc:HGNC:12006]	3180.8	2712.5	**-1.18**	1.47E-07
UBFD1	ubiquitin family domain containing 1 [Source:HGNC Symbol;Acc:HGNC:30565]	1945.0	1642.9	**-1.18**	2.40E-07
CEP57	centrosomal protein 57 [Source:HGNC Symbol;Acc:HGNC:30794]	1531.1	1301.4	**-1.18**	4.06E-07
CNPY2	canopy FGF signaling regulator 2 [Source:HGNC Symbol;Acc:HGNC:13529]	1561.6	1316.1	**-1.18**	5.29E-06
CERK	ceramide kinase [Source:HGNC Symbol;Acc:HGNC:19256]	2206.9	1866.3	**-1.18**	5.84E-06
PGD	phosphogluconate dehydrogenase [Source:HGNC Symbol;Acc:HGNC:8891]	1816.8	1562.7	**-1.18**	6.43E-06
TP53BP1	tumor protein p53 binding protein 1 [Source:HGNC Symbol;Acc:HGNC:11999]	2723.3	2310.1	**-1.18**	8.68E-06
ATP7A	ATPase copper transporting alpha [Source:HGNC Symbol;Acc:HGNC:869]	1345.8	1137.2	**-1.18**	1.24E-05
GRPEL2	GrpE like 2, mitochondrial [Source:HGNC Symbol;Acc:HGNC:21060]	960.0	817.2	**-1.18**	2.30E-05
COX17	COX17, cytochrome c oxidase copper chaperone [Source:HGNC Symbol;Acc:HGNC:2264]	1181.2	996.3	**-1.18**	3.31E-05
ARHGAP31	Rho GTPase activating protein 31 [Source:HGNC Symbol;Acc:HGNC:29216]	930.9	787.1	**-1.18**	4.15E-05
MPC2	mitochondrial pyruvate carrier 2 [Source:HGNC Symbol;Acc:HGNC:24515]	775.1	657.2	**-1.18**	4.27E-05
GUK1	guanylate kinase 1 [Source:HGNC Symbol;Acc:HGNC:4693]	1345.1	1137.0	**-1.18**	0.0001273
FAM149B1	family with sequence similarity 149 member B1 [Source:HGNC Symbol;Acc:HGNC:29162]	1759.0	1487.5	**-1.18**	0.000155
SELENOI	selenoprotein I [Source:HGNC Symbol;Acc:HGNC:29361]	1887.2	1610.8	**-1.18**	0.0001621
ZFP90	ZFP90 zinc finger protein [Source:HGNC Symbol;Acc:HGNC:23329]	919.1	780.6	**-1.18**	0.0002235
MRPL32	mitochondrial ribosomal protein L32 [Source:HGNC Symbol;Acc:HGNC:14035]	1121.7	948.5	**-1.18**	0.0003363
CLPB	ClpB homolog, mitochondrial AAA ATPase chaperonin [Source:HGNC Symbol;Acc:HGNC:30664]	616.8	525.1	**-1.18**	0.0004108
TICAM2	toll like receptor adaptor molecule 2 [Source:HGNC Symbol;Acc:HGNC:21354]	764.2	647.4	**-1.18**	0.0004236
SUPV3L1	Suv3 like RNA helicase [Source:HGNC Symbol;Acc:HGNC:11471]	526.8	447.2	**-1.18**	0.0004294
RFC5	replication factor C subunit 5 [Source:HGNC Symbol;Acc:HGNC:9973]	632.7	537.9	**-1.18**	0.00102
UBXN8	UBX domain protein 8 [Source:HGNC Symbol;Acc:HGNC:30307]	564.0	477.9	**-1.18**	0.001095
CCT2	chaperonin containing TCP1 subunit 2 [Source:HGNC Symbol;Acc:HGNC:1615]	3885.4	3285.1	**-1.18**	0.00128
JMY	junction mediating and regulatory protein, p53 cofactor [Source:HGNC Symbol;Acc:HGNC:28916]	1071.5	906.9	**-1.18**	0.001503
EARS2	glutamyl-tRNA synthetase 2, mitochondrial [Source:HGNC Symbol;Acc:HGNC:29419]	382.2	322.6	**-1.18**	0.002048
GDF9	growth differentiation factor 9 [Source:HGNC Symbol;Acc:HGNC:4224]	292.2	247.6	**-1.18**	0.002995
AEN	apoptosis enhancing nuclease [Source:HGNC Symbol;Acc:HGNC:25722]	515.0	438.6	**-1.18**	0.003204
EXOSC9	exosome component 9 [Source:HGNC Symbol;Acc:HGNC:9137]	977.8	832.5	**-1.18**	0.003633
COX18	COX18, cytochrome c oxidase assembly factor [Source:HGNC Symbol;Acc:HGNC:26801]	371.7	313.3	**-1.18**	0.003934
SH3BP5	SH3 domain binding protein 5 [Source:HGNC Symbol;Acc:HGNC:10827]	1810.9	1517.1	**-1.18**	0.004693
ALG1	ALG1, chitobiosyldiphosphodolichol beta-mannosyltransferase [Source:HGNC Symbol;Acc:HGNC:18294]	488.1	414.7	**-1.18**	0.004841
KDELC1	KDEL motif containing 1 [Source:HGNC Symbol;Acc:HGNC:19350]	352.8	300.9	**-1.18**	0.004912

PIH1D1	PIH1 domain containing 1 [Source:HGNC Symbol;Acc:HGNC:26075]	438.2	372.6	**-1.18**	0.006555
CAPRIN2	caprin family member 2 [Source:HGNC Symbol;Acc:HGNC:21259]	535.7	452.0	**-1.18**	0.006827
TEF	TEF, PAR bZIP transcription factor [Source:HGNC Symbol;Acc:HGNC:11722]	663.5	565.4	**-1.18**	0.01293
MRPL1	mitochondrial ribosomal protein L1 [Source:HGNC Symbol;Acc:HGNC:14275]	652.3	555.7	**-1.18**	0.01316
GLRX2	glutaredoxin 2 [Source:HGNC Symbol;Acc:HGNC:16065]	449.2	380.0	**-1.18**	0.01321
PFAS	phosphoribosylformylglycinamidine synthase [Source:HGNC Symbol;Acc:HGNC:8863]	240.8	203.5	**-1.18**	0.01327
ITFG2	integrin alpha FG-GAP repeat containing 2 [Source:HGNC Symbol;Acc:HGNC:30879]	296.0	248.8	**-1.18**	0.01507
NMI	N-myc and STAT interactor [Source:HGNC Symbol;Acc:HGNC:7854]	247.0	210.6	**-1.18**	0.01695
FAM118A	family with sequence similarity 118 member A [Source:HGNC Symbol;Acc:HGNC:1313]	219.3	186.5	**-1.18**	0.01904
MRPS11	mitochondrial ribosomal protein S11 [Source:HGNC Symbol;Acc:HGNC:14050]	344.9	292.8	**-1.18**	0.01986
STIM2	stromal interaction molecule 2 [Source:HGNC Symbol;Acc:HGNC:19205]	376.6	317.3	**-1.18**	0.02193
ZHX2	zinc fingers and homeoboxes 2 [Source:HGNC Symbol;Acc:HGNC:18513]	499.9	422.7	**-1.18**	0.02261
PANK4	pantothenate kinase 4 [Source:HGNC Symbol;Acc:HGNC:19366]	214.6	181.4	**-1.18**	0.02386
CYTH2	cytohesin 2 [Source:HGNC Symbol;Acc:HGNC:9502]	298.2	252.3	**-1.18**	0.0286
MRPL54	mitochondrial ribosomal protein L54 [Source:HGNC Symbol;Acc:HGNC:16685]	245.5	208.5	**-1.18**	0.03385
LINC01116	long intergenic non-protein coding RNA 1116 [Source:HGNC Symbol;Acc:HGNC:49259]	232.1	196.8	**-1.18**	0.04082
TPCN1	two pore segment channel 1 [Source:HGNC Symbol;Acc:HGNC:18182]	215.4	182.5	**-1.18**	0.04708
PDF	peptide deformylase, mitochondrial [Source:HGNC Symbol;Acc:HGNC:30012]	206.7	175.3	**-1.18**	0.04755
ARMCX4	armadillo repeat containing X-linked 4 [Source:HGNC Symbol;Acc:HGNC:28615]	166.2	140.7	**-1.18**	0.04873
ZNF772	zinc finger protein 772 [Source:HGNC Symbol;Acc:HGNC:33106]	192.1	162.0	**-1.18**	0.04883
PJA2	praja ring finger ubiquitin ligase 2 [Source:HGNC Symbol;Acc:HGNC:17481]	11021.2	9269.5	**-1.19**	2.67E-14
LMNA	lamin A/C [Source:HGNC Symbol;Acc:HGNC:6636]	8914.4	7516.4	**-1.19**	1.02E-10
SPPL2A	signal peptide peptidase like 2A [Source:HGNC Symbol;Acc:HGNC:30227]	7853.8	6572.9	**-1.19**	1.55E-10
DLG1	discs large MAGUK scaffold protein 1 [Source:HGNC Symbol;Acc:HGNC:2900]	3661.5	3081.7	**-1.19**	2.57E-08
SNHG14	small nucleolar RNA host gene 14 [Source:HGNC Symbol;Acc:HGNC:37462]	1369.8	1146.1	**-1.19**	1.32E-07
VIM	vimentin [Source:HGNC Symbol;Acc:HGNC:12692]	70738.9	59702.5	**-1.19**	1.64E-07
ARL1	ADP ribosylation factor like GTPase 1 [Source:HGNC Symbol;Acc:HGNC:692]	6333.4	5304.3	**-1.19**	1.74E-07
PSMB5	proteasome subunit beta 5 [Source:HGNC Symbol;Acc:HGNC:9542]	2225.9	1869.1	**-1.19**	8.93E-07
SULF2	sulfatase 2 [Source:HGNC Symbol;Acc:HGNC:20392]	2879.9	2426.0	**-1.19**	1.42E-06
PSD3	pleckstrin and Sec7 domain containing 3 [Source:HGNC Symbol;Acc:HGNC:19093]	14652.1	12371.4	**-1.19**	2.17E-06
RXRA	retinoid X receptor alpha [Source:HGNC Symbol;Acc:HGNC:10477]	1159.0	972.7	**-1.19**	4.82E-06
NT5E	5'-nucleotidase ecto [Source:HGNC Symbol;Acc:HGNC:8021]	68241.2	57170.8	**-1.19**	5.13E-06
SNRPN	small nuclear ribonucleoprotein polypeptide N [Source:HGNC Symbol;Acc:HGNC:11164]	1582.5	1334.6	**-1.19**	5.61E-06
NF2	neurofibromin 2 [Source:HGNC Symbol;Acc:HGNC:7773]	1625.4	1367.4	**-1.19**	7.41E-06
SNX27	sorting nexin family member 27 [Source:HGNC Symbol;Acc:HGNC:20073]	1059.4	892.3	**-1.19**	9.64E-06
GTF2I	general transcription factor IIi [Source:HGNC Symbol;Acc:HGNC:4659]	1654.4	1398.5	**-1.19**	1.16E-05
KHDRBS1	KH RNA binding domain containing, signal transduction associated 1 [Source:HGNC Symbol;Acc:HGNC:1	2677.2	2254.1	**-1.19**	5.35E-05
PTS	6-pyruvoyltetrahydropterin synthase [Source:HGNC Symbol;Acc:HGNC:9689]	988.6	828.4	**-1.19**	5.71E-05
SLC30A6	solute carrier family 30 member 6 [Source:HGNC Symbol;Acc:HGNC:19305]	1445.1	1213.6	**-1.19**	7.27E-05
ISCU	iron-sulfur cluster assembly enzyme [Source:HGNC Symbol;Acc:HGNC:29882]	1608.0	1357.8	**-1.19**	0.0001041
ORAI2	ORAI calcium release-activated calcium modulator 2 [Source:HGNC Symbol;Acc:HGNC:21667]	491.1	414.1	**-1.19**	0.0001485
ADNP	activity dependent neuroprotector homeobox [Source:HGNC Symbol;Acc:HGNC:15766]	946.1	797.9	**-1.19**	0.0001519
USP37	ubiquitin specific peptidase 37 [Source:HGNC Symbol;Acc:HGNC:20063]	972.6	824.1	**-1.19**	0.0001667
MAD1L1	mitotic arrest deficient 1 like 1 [Source:HGNC Symbol;Acc:HGNC:6762]	565.7	475.8	**-1.19**	0.0001804
PPA2	pyrophosphatase (inorganic) 2 [Source:HGNC Symbol;Acc:HGNC:28883]	1202.3	1014.9	**-1.19**	0.0001952
RNF144B	ring finger protein 144B [Source:HGNC Symbol;Acc:HGNC:21578]	2138.7	1788.7	**-1.19**	0.0002196
OARD1	O-acyl-ADP-ribose deacylase 1 [Source:HGNC Symbol;Acc:HGNC:21257]	687.9	576.2	**-1.19**	0.0002207
RBAK	RB associated KRAB zinc finger [Source:HGNC Symbol;Acc:HGNC:17680]	849.1	708.6	**-1.19**	0.0003163
C1orf52	chromosome 1 open reading frame 52 [Source:HGNC Symbol;Acc:HGNC:24871]	429.2	359.9	**-1.19**	0.0004384
AP2S1	adaptor related protein complex 2 subunit sigma 1 [Source:HGNC Symbol;Acc:HGNC:565]	466.5	390.7	**-1.19**	0.0004439
CSTF3	cleavage stimulation factor subunit 3 [Source:HGNC Symbol;Acc:HGNC:2485]	982.7	831.7	**-1.19**	0.0005912
EEF1E1-BLOC1S5	EEF1E1-BLOC1S5 readthrough (NMD candidate) [Source:HGNC Symbol;Acc:HGNC:49187]	714.5	599.1	**-1.19**	0.0006283
THAP9-AS1	THAP9 antisense RNA 1 [Source:HGNC Symbol;Acc:HGNC:44172]	815.1	682.2	**-1.19**	0.0007337
HDGFL2	HDGF like 2 [Source:HGNC Symbol;Acc:HGNC:14680]	596.0	500.7	**-1.19**	0.0009346
NDUFS6	NADH:ubiquinone oxidoreductase subunit S6 [Source:HGNC Symbol;Acc:HGNC:7713]	643.5	540.1	**-1.19**	0.001118
ARHGAP1	Rho GTPase activating protein 1 [Source:HGNC Symbol;Acc:HGNC:673]	644.5	541.8	**-1.19**	0.001406
BORCS7-ASMT	BORCS7-ASMT readthrough (NMD candidate) [Source:HGNC Symbol;Acc:HGNC:49183]	461.3	389.9	**-1.19**	0.001787
P2RX4	purinergic receptor P2X 4 [Source:HGNC Symbol;Acc:HGNC:8535]	331.4	278.4	**-1.19**	0.003163
ZNF583	zinc finger protein 583 [Source:HGNC Symbol;Acc:HGNC:26427]	336.4	281.9	**-1.19**	0.003243
MGME1	mitochondrial genome maintenance exonuclease 1 [Source:HGNC Symbol;Acc:HGNC:16205]	328.4	277.2	**-1.19**	0.003339
DCLRE1A	DNA cross-link repair 1A [Source:HGNC Symbol;Acc:HGNC:17660]	517.9	438.2	**-1.19**	0.003521
APOL6	apolipoprotein L6 [Source:HGNC Symbol;Acc:HGNC:14870]	833.9	707.3	**-1.19**	0.003817
ZFYVE19	zinc finger FYVE-type containing 19 [Source:HGNC Symbol;Acc:HGNC:20758]	313.1	263.1	**-1.19**	0.004952
ATG9A	autophagy related 9A [Source:HGNC Symbol;Acc:HGNC:22408]	590.2	497.4	**-1.19**	0.005393
RNF5	ring finger protein 5 [Source:HGNC Symbol;Acc:HGNC:10068]	277.5	232.8	**-1.19**	0.00583
NDUFAF8	NADH:ubiquinone oxidoreductase complex assembly factor 8 [Source:HGNC Symbol;Acc:HGNC:33551]	399.7	337.9	**-1.19**	0.007234
RTEL1	regulator of telomere elongation helicase 1 [Source:HGNC Symbol;Acc:HGNC:15888]	245.3	205.6	**-1.19**	0.00823
VIL1	villin 1 [Source:HGNC Symbol;Acc:HGNC:12690]	341.0	288.8	**-1.19**	0.01062
LINC00888	long intergenic non-protein coding RNA 888 [Source:HGNC Symbol;Acc:HGNC:48575]	278.3	232.9	**-1.19**	0.01727
HOXC10	homeobox C10 [Source:HGNC Symbol;Acc:HGNC:5122]	275.1	231.6	**-1.19**	0.02365
KCTD11	potassium channel tetramerization domain containing 11 [Source:HGNC Symbol;Acc:HGNC:21302]	189.8	158.7	**-1.19**	0.02906
HSPB1	heat shock protein family B (small) member 1 [Source:HGNC Symbol;Acc:HGNC:5246]	468.0	389.0	**-1.19**	0.02932

UBE2M	ubiquitin conjugating enzyme E2 M [Source:HGNC Symbol;Acc:HGNC:12491]	339.1	283.7	**-1.19**	0.03168
ZNF250	zinc finger protein 250 [Source:HGNC Symbol;Acc:HGNC:13044]	264.8	223.3	**-1.19**	0.03654
ZNF550	zinc finger protein 550 [Source:HGNC Symbol;Acc:HGNC:28643]	192.3	160.3	**-1.19**	0.0416
ERMAP	erythroblast membrane associated protein (Scianna blood group) [Source:HGNC Symbol;Acc:HGNC:157	480.1	413.1	**-1.19**	0.04503
FOXRED1	FAD dependent oxidoreductase domain containing 1 [Source:HGNC Symbol;Acc:HGNC:26927]	174.0	146.4	**-1.19**	0.04719
SERBP1	SERPINE1 mRNA binding protein 1 [Source:HGNC Symbol;Acc:HGNC:17860]	10542.6	8810.6	**-1.20**	1.66E-14
RCN3	reticulocalbin 3 [Source:HGNC Symbol;Acc:HGNC:21145]	3662.2	3076.2	**-1.20**	3.47E-14
EXOC7	exocyst complex component 7 [Source:HGNC Symbol;Acc:HGNC:23214]	1989.4	1661.6	**-1.20**	3.98E-11
NCL	nucleolin [Source:HGNC Symbol;Acc:HGNC:7667]	13834.0	11627.1	**-1.20**	2.28E-08
JKAMP	JNK1/MAPK8 associated membrane protein [Source:HGNC Symbol;Acc:HGNC:20184]	1585.1	1318.6	**-1.20**	3.87E-08
UBA6	ubiquitin like modifier activating enzyme 6 [Source:HGNC Symbol;Acc:HGNC:25581]	2927.0	2451.0	**-1.20**	3.90E-07
PDCD6	programmed cell death 6 [Source:HGNC Symbol;Acc:HGNC:8765]	1958.8	1628.8	**-1.20**	5.42E-07
USP5	ubiquitin specific peptidase 5 [Source:HGNC Symbol;Acc:HGNC:12628]	1419.1	1188.0	**-1.20**	5.55E-07
RBM8A	RNA binding motif protein 8A [Source:HGNC Symbol;Acc:HGNC:9905]	2733.7	2276.8	**-1.20**	1.43E-06
HOXA10	homeobox A10 [Source:HGNC Symbol;Acc:HGNC:5100]	701.2	586.1	**-1.20**	1.86E-06
SLC35G2	solute carrier family 35 member G2 [Source:HGNC Symbol;Acc:HGNC:28480]	701.7	584.0	**-1.20**	3.47E-06
PSMG1	proteasome assembly chaperone 1 [Source:HGNC Symbol;Acc:HGNC:3043]	1169.9	981.5	**-1.20**	5.77E-06
KPNA5	karyopherin subunit alpha 5 [Source:HGNC Symbol;Acc:HGNC:6398]	799.9	668.5	**-1.20**	7.20E-06
INPPL1	inositol polyphosphate phosphatase like 1 [Source:HGNC Symbol;Acc:HGNC:6080]	855.1	710.9	**-1.20**	1.84E-05
EIF4E	eukaryotic translation initiation factor 4E [Source:HGNC Symbol;Acc:HGNC:3287]	2137.1	1779.0	**-1.20**	1.90E-05
MAPK9	mitogen-activated protein kinase 9 [Source:HGNC Symbol;Acc:HGNC:6886]	730.4	609.9	**-1.20**	3.85E-05
FBXL17	F-box and leucine rich repeat protein 17 [Source:HGNC Symbol;Acc:HGNC:13615]	835.9	698.9	**-1.20**	5.25E-05
C3orf58	chromosome 3 open reading frame 58 [Source:HGNC Symbol;Acc:HGNC:28490]	654.6	546.6	**-1.20**	5.28E-05
MTHFD2	methylenetetrahydrofolate dehydrogenase (NADP+ dependent) 2, methenyltetrahydrofolate cyclohydr	5287.1	4415.1	**-1.20**	5.72E-05
ERCC1	ERCC excision repair 1, endonuclease non-catalytic subunit [Source:HGNC Symbol;Acc:HGNC:3433]	909.3	753.6	**-1.20**	7.49E-05
SMAD3	SMAD family member 3 [Source:HGNC Symbol;Acc:HGNC:6769]	1007.2	839.7	**-1.20**	9.87E-05
FCHSD2	FCH and double SH3 domains 2 [Source:HGNC Symbol;Acc:HGNC:29114]	1415.8	1185.5	**-1.20**	0.0001559
ASS1	argininosuccinate synthase 1 [Source:HGNC Symbol;Acc:HGNC:758]	1429.6	1210.4	**-1.20**	0.0001627
GALE	UDP-galactose-4-epimerase [Source:HGNC Symbol;Acc:HGNC:4116]	770.0	642.1	**-1.20**	0.0002697
HLCS	holocarboxylase synthetase [Source:HGNC Symbol;Acc:HGNC:4976]	921.4	767.0	**-1.20**	0.0004437
WDR26	WD repeat domain 26 [Source:HGNC Symbol;Acc:HGNC:21208]	1714.2	1438.7	**-1.20**	0.000487
ZNHIT3	zinc finger HIT-type containing 3 [Source:HGNC Symbol;Acc:HGNC:12309]	456.0	380.8	**-1.20**	0.0005326
SELENOH	selenoprotein H [Source:HGNC Symbol;Acc:HGNC:18251]	430.1	356.7	**-1.20**	0.0006699
C1QBP	complement C1q binding protein [Source:HGNC Symbol;Acc:HGNC:1243]	3264.9	2743.4	**-1.20**	0.0007917
COL9A3	collagen type IX alpha 3 chain [Source:HGNC Symbol;Acc:HGNC:2219]	2746.7	2328.8	**-1.20**	0.001266
SYTL4	synaptotagmin like 4 [Source:HGNC Symbol;Acc:HGNC:15588]	687.0	571.5	**-1.20**	0.001419
PLAC9	placenta specific 9 [Source:HGNC Symbol;Acc:HGNC:19255]	504.7	424.2	**-1.20**	0.001437
ZNF184	zinc finger protein 184 [Source:HGNC Symbol;Acc:HGNC:12975]	393.8	328.3	**-1.20**	0.001551
TMEM220	transmembrane protein 220 [Source:HGNC Symbol;Acc:HGNC:33757]	307.9	258.2	**-1.20**	0.002486
R3HDM2	R3H domain containing 2 [Source:HGNC Symbol;Acc:HGNC:29167]	714.8	595.7	**-1.20**	0.002721
REPIN1	replication initiator 1 [Source:HGNC Symbol;Acc:HGNC:17922]	230.8	192.8	**-1.20**	0.003437
SAMD1	sterile alpha motif domain containing 1 [Source:HGNC Symbol;Acc:HGNC:17958]	267.8	224.2	**-1.20**	0.004184
GLB1L	galactosidase beta 1 like [Source:HGNC Symbol;Acc:HGNC:28129]	280.4	234.6	**-1.20**	0.005461
COLEC12	collectin subfamily member 12 [Source:HGNC Symbol;Acc:HGNC:16016]	2279.8	1908.5	**-1.20**	0.006103
MBTPS2	membrane bound transcription factor peptidase, site 2 [Source:HGNC Symbol;Acc:HGNC:15455]	748.1	623.5	**-1.20**	0.01073
RUBCNL	RUN and cysteine rich domain containing beclin 1 interacting protein like [Source:HGNC Symbol;Acc:HG	386.3	325.9	**-1.20**	0.01075
NR2F6	nuclear receptor subfamily 2 group F member 6 [Source:HGNC Symbol;Acc:HGNC:7977]	209.0	174.4	**-1.20**	0.01742
IQCK	IQ motif containing K [Source:HGNC Symbol;Acc:HGNC:28556]	234.1	194.2	**-1.20**	0.02226
SPRYD4	SPRY domain containing 4 [Source:HGNC Symbol;Acc:HGNC:27468]	221.3	186.5	**-1.20**	0.04521
FAM114A1	family with sequence similarity 114 member A1 [Source:HGNC Symbol;Acc:HGNC:25087]	8314.0	6914.3	**-1.21**	4.67E-14
EIF2AK2	eukaryotic translation initiation factor 2 alpha kinase 2 [Source:HGNC Symbol;Acc:HGNC:9437]	2023.9	1677.4	**-1.21**	8.32E-11
TSPAN3	tetraspanin 3 [Source:HGNC Symbol;Acc:HGNC:17752]	6247.0	5194.0	**-1.21**	8.71E-11
ALDH18A1	aldehyde dehydrogenase 18 family member A1 [Source:HGNC Symbol;Acc:HGNC:9722]	2450.8	2034.4	**-1.21**	3.90E-09
ITGB5	integrin subunit beta 5 [Source:HGNC Symbol;Acc:HGNC:6160]	31043.0	25903.6	**-1.21**	6.71E-08
HMGCR	3-hydroxy-3-methylglutaryl-CoA reductase [Source:HGNC Symbol;Acc:HGNC:5006]	4171.6	3472.0	**-1.21**	1.05E-07
FAM168A	family with sequence similarity 168 member A [Source:HGNC Symbol;Acc:HGNC:28999]	1109.4	916.3	**-1.21**	1.42E-07
RHOC	ras homolog family member C [Source:HGNC Symbol;Acc:HGNC:669]	4600.4	3770.7	**-1.21**	1.70E-07
PTDSS1	phosphatidylserine synthase 1 [Source:HGNC Symbol;Acc:HGNC:9587]	1639.6	1356.0	**-1.21**	2.36E-07
CBX6	chromobox 6 [Source:HGNC Symbol;Acc:HGNC:1556]	1157.9	962.3	**-1.21**	3.53E-07
GTF3C6	general transcription factor IIIC subunit 6 [Source:HGNC Symbol;Acc:HGNC:20872]	2514.5	2075.2	**-1.21**	1.07E-06
DZIP3	DAZ interacting zinc finger protein 3 [Source:HGNC Symbol;Acc:HGNC:30938]	1073.9	893.8	**-1.21**	1.45E-06
GNL1	G protein nucleolar 1 (putative) [Source:HGNC Symbol;Acc:HGNC:4413]	811.7	671.7	**-1.21**	1.47E-06
ACTN1	actinin alpha 1 [Source:HGNC Symbol;Acc:HGNC:163]	3808.4	3169.3	**-1.21**	1.54E-06
PRPSAP2	phosphoribosyl pyrophosphate synthetase associated protein 2 [Source:HGNC Symbol;Acc:HGNC:9467]	637.5	529.6	**-1.21**	2.07E-06
FANCL	Fanconi anemia complementation group L [Source:HGNC Symbol;Acc:HGNC:20748]	1211.2	1004.1	**-1.21**	4.89E-06
MED14	mediator complex subunit 14 [Source:HGNC Symbol;Acc:HGNC:2370]	820.9	682.2	**-1.21**	2.49E-05
CEP135	centrosomal protein 135 [Source:HGNC Symbol;Acc:HGNC:29086]	523.2	432.5	**-1.21**	2.72E-05
FUNDC1	FUN14 domain containing 1 [Source:HGNC Symbol;Acc:HGNC:28746]	733.4	603.1	**-1.21**	3.04E-05
ETFB	electron transfer flavoprotein subunit beta [Source:HGNC Symbol;Acc:HGNC:3482]	675.4	560.4	**-1.21**	3.61E-05
TBC1D22A	TBC1 domain family member 22A [Source:HGNC Symbol;Acc:HGNC:1309]	575.1	476.6	**-1.21**	4.76E-05
TMEM237	transmembrane protein 237 [Source:HGNC Symbol;Acc:HGNC:14432]	983.8	813.6	**-1.21**	6.72E-05
VIM-AS1	VIM antisense RNA 1 [Source:HGNC Symbol;Acc:HGNC:44879]	11684.6	9728.2	**-1.21**	7.59E-05

SIRPA	signal regulatory protein alpha [Source:HGNC Symbol;Acc:HGNC:9662]	978.0	813.8	**-1.21**	0.0001012
NUP62	nucleoporin 62 [Source:HGNC Symbol;Acc:HGNC:8066]	489.7	405.1	**-1.21**	0.0001129
ING3	inhibitor of growth family member 3 [Source:HGNC Symbol;Acc:HGNC:14587]	510.6	425.0	**-1.21**	0.0001349
ATXN1	ataxin 1 [Source:HGNC Symbol;Acc:HGNC:10548]	1328.8	1106.4	**-1.21**	0.00017
FGD6	FYVE, RhoGEF and PH domain containing 6 [Source:HGNC Symbol;Acc:HGNC:21740]	577.5	475.5	**-1.21**	0.0001988
SMAD6	SMAD family member 6 [Source:HGNC Symbol;Acc:HGNC:6772]	1309.9	1087.5	**-1.21**	0.0003746
SIAH1	siah E3 ubiquitin protein ligase 1 [Source:HGNC Symbol;Acc:HGNC:10857]	532.5	439.5	**-1.21**	0.000472
HPS4	HPS4, biogenesis of lysosomal organelles complex 3 subunit 2 [Source:HGNC Symbol;Acc:HGNC:15844]	547.2	453.8	**-1.21**	0.0005446
C12orf4	chromosome 12 open reading frame 4 [Source:HGNC Symbol;Acc:HGNC:1184]	789.4	660.1	**-1.21**	0.0005869
BCOR	BCL6 corepressor [Source:HGNC Symbol;Acc:HGNC:20893]	586.1	483.3	**-1.21**	0.0009675
VAV2	vav guanine nucleotide exchange factor 2 [Source:HGNC Symbol;Acc:HGNC:12658]	533.8	442.1	**-1.21**	0.001021
BTG3	BTG anti-proliferation factor 3 [Source:HGNC Symbol;Acc:HGNC:1132]	426.2	354.0	**-1.21**	0.001287
DPH5	diphthamide biosynthesis 5 [Source:HGNC Symbol;Acc:HGNC:24270]	670.6	557.0	**-1.21**	0.00166
NARF	nuclear prelamin A recognition factor [Source:HGNC Symbol;Acc:HGNC:29916]	350.7	293.1	**-1.21**	0.00197
PSPH	phosphoserine phosphatase [Source:HGNC Symbol;Acc:HGNC:9577]	432.8	361.2	**-1.21**	0.002302
MPP1	membrane palmitoylated protein 1 [Source:HGNC Symbol;Acc:HGNC:7219]	380.6	316.2	**-1.21**	0.002677
BCAR1	BCAR1, Cas family scaffold protein [Source:HGNC Symbol;Acc:HGNC:971]	498.0	416.8	**-1.21**	0.0027
PCGF6	polycomb group ring finger 6 [Source:HGNC Symbol;Acc:HGNC:21156]	271.3	224.3	**-1.21**	0.003365
FAAP20	Fanconi anemia core complex associated protein 20 [Source:HGNC Symbol;Acc:HGNC:26428]	305.0	253.1	**-1.21**	0.005256
SCML1	Scm polycomb group protein like 1 [Source:HGNC Symbol;Acc:HGNC:10580]	379.9	315.1	**-1.21**	0.005516
HMGB1P6	high mobility group box 1 pseudogene 6 [Source:HGNC Symbol;Acc:HGNC:4998]	308.9	254.8	**-1.21**	0.006027
LINC00909	long intergenic non-protein coding RNA 909 [Source:HGNC Symbol;Acc:HGNC:44331]	336.5	277.5	**-1.21**	0.006693
CEP41	centrosomal protein 41 [Source:HGNC Symbol;Acc:HGNC:12370]	298.0	247.2	**-1.21**	0.007382
PXDN	peroxidasin [Source:HGNC Symbol;Acc:HGNC:14966]	2118.0	1756.1	**-1.21**	0.007618
ARL16	ADP ribosylation factor like GTPase 16 [Source:HGNC Symbol;Acc:HGNC:27902]	328.9	272.0	**-1.21**	0.007956
ZFP14	ZFP14 zinc finger protein [Source:HGNC Symbol;Acc:HGNC:29312]	290.8	241.3	**-1.21**	0.01461
B4GAT1	beta-1,4-glucuronyltransferase 1 [Source:HGNC Symbol;Acc:HGNC:15685]	177.5	146.3	**-1.21**	0.01623
GOLGA8A	golgin A8 family member A [Source:HGNC Symbol;Acc:HGNC:31972]	252.7	211.4	**-1.21**	0.01664
DUS4L	dihydrouridine synthase 4 like [Source:HGNC Symbol;Acc:HGNC:21517]	186.5	154.7	**-1.21**	0.02937
SIPA1L3	signal induced proliferation associated 1 like 3 [Source:HGNC Symbol;Acc:HGNC:23801]	280.3	234.1	**-1.21**	0.03398
CDC14A	cell division cycle 14A [Source:HGNC Symbol;Acc:HGNC:1718]	176.9	145.3	**-1.21**	0.03978
RTN4IP1	reticulon 4 interacting protein 1 [Source:HGNC Symbol;Acc:HGNC:18647]	187.4	154.3	**-1.21**	0.04626
SMIM37	small integral membrane protein 37 [Source:HGNC Symbol;Acc:HGNC:27339]	126.0	104.3	**-1.21**	0.04716
ZDHHC15	zinc finger DHHC-type containing 15 [Source:HGNC Symbol;Acc:HGNC:20342]	209.2	173.6	**-1.21**	0.04772
CLTC	clathrin heavy chain [Source:HGNC Symbol;Acc:HGNC:2092]	24663.2	20300.2	**-1.21**	1.02E-16
H2AFV	H2A histone family member V [Source:HGNC Symbol;Acc:HGNC:20664]	3803.5	3132.1	**-1.21**	3.84E-13
DARS	aspartyl-tRNA synthetase [Source:HGNC Symbol;Acc:HGNC:2678]	5204.4	4272.5	**-1.21**	1.51E-12
ESYT1	extended synaptotagmin 1 [Source:HGNC Symbol;Acc:HGNC:29534]	2345.6	1939.9	**-1.21**	2.39E-12
SPATS2L	spermatogenesis associated serine rich 2 like [Source:HGNC Symbol;Acc:HGNC:24574]	2625.7	2151.7	**-1.21**	5.08E-10
RBPJ	recombination signal binding protein for immunoglobulin kappa J region [Source:HGNC Symbol;Acc:HGI	2055.0	1697.2	**-1.21**	8.08E-09
ZMPSTE24	zinc metallopeptidase STE24 [Source:HGNC Symbol;Acc:HGNC:12877]	5444.4	4485.3	**-1.21**	1.47E-08
SLC31A1	solute carrier family 31 member 1 [Source:HGNC Symbol;Acc:HGNC:11016]	1868.0	1530.9	**-1.21**	4.08E-08
GALNT7	polypeptide N-acetylgalactosaminyltransferase 7 [Source:HGNC Symbol;Acc:HGNC:4129]	2033.5	1667.2	**-1.21**	4.48E-08
CYB5R1	cytochrome b5 reductase 1 [Source:HGNC Symbol;Acc:HGNC:13397]	997.7	821.4	**-1.21**	6.39E-08
TKT	transketolase [Source:HGNC Symbol;Acc:HGNC:11834]	1547.1	1270.5	**-1.21**	2.47E-07
RND3	Rho family GTPase 3 [Source:HGNC Symbol;Acc:HGNC:671]	3449.1	2817.7	**-1.21**	5.41E-07
AARS	alanyl-tRNA synthetase [Source:HGNC Symbol;Acc:HGNC:20]	5139.5	4220.3	**-1.21**	1.37E-06
S100A13	S100 calcium binding protein A13 [Source:HGNC Symbol;Acc:HGNC:10490]	6331.3	5313.8	**-1.21**	1.60E-06
SPRED1	sprouty related EVH1 domain containing 1 [Source:HGNC Symbol;Acc:HGNC:20249]	3136.2	2586.8	**-1.21**	3.17E-06
FBXO9	F-box protein 9 [Source:HGNC Symbol;Acc:HGNC:13588]	1006.6	829.4	**-1.21**	3.38E-06
NSFL1C	NSFL1 cofactor [Source:HGNC Symbol;Acc:HGNC:15912]	1189.5	988.1	**-1.21**	5.49E-06
HTRA2	HtrA serine peptidase 2 [Source:HGNC Symbol;Acc:HGNC:14348]	814.8	673.9	**-1.21**	6.55E-06
PCNT	pericentrin [Source:HGNC Symbol;Acc:HGNC:16068]	1272.6	1053.2	**-1.21**	7.35E-06
DIS3L	DIS3 like exosome 3'-5' exoribonuclease [Source:HGNC Symbol;Acc:HGNC:28698]	917.7	757.2	**-1.21**	1.86E-05
STK35	serine/threonine kinase 35 [Source:HGNC Symbol;Acc:HGNC:16254]	444.4	365.8	**-1.21**	3.03E-05
RAD1	RAD1 checkpoint DNA exonuclease [Source:HGNC Symbol;Acc:HGNC:9806]	1051.5	866.2	**-1.21**	5.19E-05
HSPD1	heat shock protein family D (Hsp60) member 1 [Source:HGNC Symbol;Acc:HGNC:5261]	8477.4	7022.7	**-1.21**	5.62E-05
MIF-AS1	MIF antisense RNA 1 [Source:HGNC Symbol;Acc:HGNC:27669]	766.3	633.4	**-1.21**	6.93E-05
RNF2	ring finger protein 2 [Source:HGNC Symbol;Acc:HGNC:10061]	663.1	547.1	**-1.21**	7.12E-05
MIF	macrophage migration inhibitory factor [Source:HGNC Symbol;Acc:HGNC:7097]	760.9	629.7	**-1.21**	7.98E-05
GIN1	gypsy retrotransposon integrase 1 [Source:HGNC Symbol;Acc:HGNC:25959]	471.3	387.9	**-1.21**	0.0001155
CCDC142	coiled-coil domain containing 142 [Source:HGNC Symbol;Acc:HGNC:25889]	474.1	392.7	**-1.21**	0.0002276
POLR3B	RNA polymerase III subunit B [Source:HGNC Symbol;Acc:HGNC:30348]	590.0	489.2	**-1.21**	0.0002421
ZCCHC24	zinc finger CCHC-type containing 24 [Source:HGNC Symbol;Acc:HGNC:26911]	436.3	359.9	**-1.21**	0.0003441
SLC2A1	solute carrier family 2 member 1 [Source:HGNC Symbol;Acc:HGNC:11005]	1160.8	950.8	**-1.21**	0.0004532
ABHD10	abhydrolase domain containing 10 [Source:HGNC Symbol;Acc:HGNC:25656]	971.3	796.5	**-1.21**	0.0005524
OGFOD3	2-oxoglutarate and iron dependent oxygenase domain containing 3 [Source:HGNC Symbol;Acc:HGNC:2(	438.9	361.6	**-1.21**	0.000718
LANCL2	LanC like 2 [Source:HGNC Symbol;Acc:HGNC:6509]	322.1	264.7	**-1.21**	0.000734
PFDN4	prefoldin subunit 4 [Source:HGNC Symbol;Acc:HGNC:8868]	740.7	617.0	**-1.21**	0.0009213
MAPK3	mitogen-activated protein kinase 3 [Source:HGNC Symbol;Acc:HGNC:6877]	483.6	396.2	**-1.21**	0.00136
EHD1	EH domain containing 1 [Source:HGNC Symbol;Acc:HGNC:3242]	412.9	341.2	**-1.21**	0.002118
SNHG1	small nucleolar RNA host gene 1 [Source:HGNC Symbol;Acc:HGNC:32688]	607.4	504.5	**-1.21**	0.002895

ENTPD1	ectonucleoside triphosphate diphosphohydrolase 1 [Source:HGNC Symbol;Acc:HGNC:3363]	451.9	373.8	**-1.21**	0.00334
SPDL1	spindle apparatus coiled-coil protein 1 [Source:HGNC Symbol;Acc:HGNC:26010]	560.9	465.9	**-1.21**	0.00451
SLC4A5	solute carrier family 4 member 5 [Source:HGNC Symbol;Acc:HGNC:18168]	262.3	215.4	**-1.21**	0.008092
STOML1	stomatin like 1 [Source:HGNC Symbol;Acc:HGNC:14560]	213.4	176.0	**-1.21**	0.01058
B4GALT3	beta-1,4-galactosyltransferase 3 [Source:HGNC Symbol;Acc:HGNC:926]	228.0	185.3	**-1.21**	0.01137
MAOB	monoamine oxidase B [Source:HGNC Symbol;Acc:HGNC:6834]	876.2	721.1	**-1.21**	0.01216
RXYLT1-AS1	RXYLT1 antisense RNA 1 [Source:HGNC Symbol;Acc:HGNC:48910]	958.7	819.4	**-1.21**	0.01602
CCNK	cyclin K [Source:HGNC Symbol;Acc:HGNC:1596]	256.9	213.3	**-1.21**	0.01842
PPP2R5B	protein phosphatase 2 regulatory subunit B'beta [Source:HGNC Symbol;Acc:HGNC:9310]	198.3	164.0	**-1.21**	0.01912
DPY19L2	dpy-19 like 2 [Source:HGNC Symbol;Acc:HGNC:19414]	138.6	114.2	**-1.21**	0.02574
CYP27C1	cytochrome P450 family 27 subfamily C member 1 [Source:HGNC Symbol;Acc:HGNC:33480]	502.0	417.2	**-1.21**	0.03548
CD47	CD47 molecule [Source:HGNC Symbol;Acc:HGNC:1682]	3447.4	2815.0	**-1.22**	3.00E-13
CHD4	chromodomain helicase DNA binding protein 4 [Source:HGNC Symbol;Acc:HGNC:1919]	5669.1	4644.3	**-1.22**	1.76E-12
ITGB1BP1	integrin subunit beta 1 binding protein 1 [Source:HGNC Symbol;Acc:HGNC:23927]	2636.7	2167.7	**-1.22**	5.86E-12
RRBP1	ribosome binding protein 1 [Source:HGNC Symbol;Acc:HGNC:10448]	10721.6	8769.8	**-1.22**	4.08E-11
GOLGA2	golgin A2 [Source:HGNC Symbol;Acc:HGNC:4425]	4115.1	3378.1	**-1.22**	4.63E-11
DOCK7	dedicator of cytokinesis 7 [Source:HGNC Symbol;Acc:HGNC:19190]	2653.6	2174.0	**-1.22**	5.00E-10
POFUT1	protein O-fucosyltransferase 1 [Source:HGNC Symbol;Acc:HGNC:14988]	2596.8	2131.7	**-1.22**	1.28E-09
TRPC1	transient receptor potential cation channel subfamily C member 1 [Source:HGNC Symbol;Acc:HGNC:123	960.3	782.1	**-1.22**	2.20E-09
SPIN1	spindlin 1 [Source:HGNC Symbol;Acc:HGNC:11243]	2171.4	1782.2	**-1.22**	8.51E-09
ATP2A2	ATPase sarcoplasmic/endoplasmic reticulum Ca2+ transporting 2 [Source:HGNC Symbol;Acc:HGNC:812]	3547.3	2927.2	**-1.22**	4.68E-08
ARL6IP4	ADP ribosylation factor like GTPase 6 interacting protein 4 [Source:HGNC Symbol;Acc:HGNC:18076]	1872.9	1542.1	**-1.22**	1.02E-07
BACE1	beta-secretase 1 [Source:HGNC Symbol;Acc:HGNC:933]	959.7	787.5	**-1.22**	2.63E-07
CLCN4	chloride voltage-gated channel 4 [Source:HGNC Symbol;Acc:HGNC:2022]	902.4	739.7	**-1.22**	2.08E-06
CHPF2	chondroitin polymerizing factor 2 [Source:HGNC Symbol;Acc:HGNC:29270]	1204.6	989.7	**-1.22**	2.75E-06
MEGF8	multiple EGF like domains 8 [Source:HGNC Symbol;Acc:HGNC:3233]	812.1	664.1	**-1.22**	3.78E-06
INPP4A	inositol polyphosphate-4-phosphatase type I A [Source:HGNC Symbol;Acc:HGNC:6074]	815.6	665.7	**-1.22**	1.18E-05
NADK2	NAD kinase 2, mitochondrial [Source:HGNC Symbol;Acc:HGNC:26404]	1458.9	1197.7	**-1.22**	1.75E-05
FKRP	fukutin related protein [Source:HGNC Symbol;Acc:HGNC:17997]	498.5	405.9	**-1.22**	2.19E-05
GADD45GIP1	GADD45G interacting protein 1 [Source:HGNC Symbol;Acc:HGNC:29996]	806.8	661.4	**-1.22**	2.93E-05
CEP57L1	centrosomal protein 57 like 1 [Source:HGNC Symbol;Acc:HGNC:21561]	565.6	463.7	**-1.22**	3.64E-05
EXOSC8	exosome component 8 [Source:HGNC Symbol;Acc:HGNC:17035]	680.8	559.2	**-1.22**	3.75E-05
HSPE1-MOB4	HSPE1-MOB4 readthrough [Source:HGNC Symbol;Acc:HGNC:49184]	2584.3	2138.8	**-1.22**	4.50E-05
ASB1	ankyrin repeat and SOCS box containing 1 [Source:HGNC Symbol;Acc:HGNC:16011]	641.6	521.7	**-1.22**	5.53E-05
OSGEP	O-sialoglycoprotein endopeptidase [Source:HGNC Symbol;Acc:HGNC:18028]	421.2	345.1	**-1.22**	0.0001755
CEP152	centrosomal protein 152 [Source:HGNC Symbol;Acc:HGNC:29298]	417.5	341.5	**-1.22**	0.00018
SLC35B4	solute carrier family 35 member B4 [Source:HGNC Symbol;Acc:HGNC:20584]	1048.2	868.1	**-1.22**	0.0001887
LDLRAP1	low density lipoprotein receptor adaptor protein 1 [Source:HGNC Symbol;Acc:HGNC:18640]	482.9	394.1	**-1.22**	0.0001957
PXN	paxillin [Source:HGNC Symbol;Acc:HGNC:9718]	311.9	253.1	**-1.22**	0.0004082
HEATR3	HEAT repeat containing 3 [Source:HGNC Symbol;Acc:HGNC:26087]	530.3	430.9	**-1.22**	0.0004385
FANCM	Fanconi anemia complementation group M [Source:HGNC Symbol;Acc:HGNC:23168]	422.3	347.5	**-1.22**	0.0005751
ZSWIM6	zinc finger SWIM-type containing 6 [Source:HGNC Symbol;Acc:HGNC:29316]	554.6	458.6	**-1.22**	0.0006011
MRPL53	mitochondrial ribosomal protein L53 [Source:HGNC Symbol;Acc:HGNC:16684]	396.0	325.2	**-1.22**	0.0006508
SLC46A1	solute carrier family 46 member 1 [Source:HGNC Symbol;Acc:HGNC:30521]	324.8	264.6	**-1.22**	0.0007993
GAPDHP1	glyceraldehyde-3-phosphate dehydrogenase pseudogene 1 [Source:HGNC Symbol;Acc:HGNC:4159]	330.9	272.2	**-1.22**	0.001796
FLI1	Fli-1 proto-oncogene, ETS transcription factor [Source:HGNC Symbol;Acc:HGNC:3749]	239.2	194.1	**-1.22**	0.001839
AS3MT	arsenite methyltransferase [Source:HGNC Symbol;Acc:HGNC:17452]	363.4	298.3	**-1.22**	0.002497
NAA40	N(alpha)-acetyltransferase 40, NatD catalytic subunit [Source:HGNC Symbol;Acc:HGNC:25845]	279.6	228.4	**-1.22**	0.002898
TMEM173	transmembrane protein 173 [Source:HGNC Symbol;Acc:HGNC:27962]	441.8	362.9	**-1.22**	0.004185
POLE4	DNA polymerase epsilon 4, accessory subunit [Source:HGNC Symbol;Acc:HGNC:18755]	235.9	193.5	**-1.22**	0.004473
C9	complement C9 [Source:HGNC Symbol;Acc:HGNC:1358]	236.0	191.4	**-1.22**	0.004705
SARM1	sterile alpha and TIR motif containing 1 [Source:HGNC Symbol;Acc:HGNC:17074]	298.2	243.6	**-1.22**	0.005117
IFITM1	interferon induced transmembrane protein 1 [Source:HGNC Symbol;Acc:HGNC:5412]	344.7	287.1	**-1.22**	0.0069
POC5	POC5 centriolar protein [Source:HGNC Symbol;Acc:HGNC:26658]	215.1	176.0	**-1.22**	0.007675
PSMB8	proteasome subunit beta 8 [Source:HGNC Symbol;Acc:HGNC:9545]	181.8	149.2	**-1.22**	0.01592
PNPLA4	patatin like phospholipase domain containing 4 [Source:HGNC Symbol;Acc:HGNC:24887]	221.2	180.6	**-1.22**	0.01625
ACYP2	acylphosphatase 2 [Source:HGNC Symbol;Acc:HGNC:180]	319.5	261.0	**-1.22**	0.01882
ZNF527	zinc finger protein 527 [Source:HGNC Symbol;Acc:HGNC:29385]	152.2	123.8	**-1.22**	0.02039
DTX3	deltex E3 ubiquitin ligase 3 [Source:HGNC Symbol;Acc:HGNC:24457]	126.9	103.7	**-1.22**	0.02294
TSGA10	testis specific 10 [Source:HGNC Symbol;Acc:HGNC:14927]	155.3	126.4	**-1.22**	0.02399
THOC6	THO complex 6 [Source:HGNC Symbol;Acc:HGNC:28369]	149.3	122.5	**-1.22**	0.03377
EIF4G2	eukaryotic translation initiation factor 4 gamma 2 [Source:HGNC Symbol;Acc:HGNC:3297]	23758.0	19285.2	**-1.23**	4.86E-17
FIP1L1	factor interacting with PAPOLA and CPSF1 [Source:HGNC Symbol;Acc:HGNC:19124]	3175.0	2588.0	**-1.23**	7.65E-14
HSP90AB1	heat shock protein 90 alpha family class B member 1 [Source:HGNC Symbol;Acc:HGNC:5258]	25696.4	20845.5	**-1.23**	2.16E-12
AHI1	Abelson helper integration site 1 [Source:HGNC Symbol;Acc:HGNC:21575]	2038.2	1650.3	**-1.23**	9.24E-11
SUMO2	small ubiquitin-like modifier 2 [Source:HGNC Symbol;Acc:HGNC:11125]	3031.6	2458.8	**-1.23**	1.17E-10
KCNQ5	potassium voltage-gated channel subfamily Q member 5 [Source:HGNC Symbol;Acc:HGNC:6299]	2824.2	2296.9	**-1.23**	1.88E-09
NDRG1	N-myc downstream regulated 1 [Source:HGNC Symbol;Acc:HGNC:7679]	5010.9	4031.1	**-1.23**	9.05E-09
NOC2L	NOC2 like nucleolar associated transcriptional repressor [Source:HGNC Symbol;Acc:HGNC:24517]	1329.1	1077.3	**-1.23**	5.49E-08
SPATA5	spermatogenesis associated 5 [Source:HGNC Symbol;Acc:HGNC:18119]	1010.8	821.7	**-1.23**	2.96E-07
PTPN4	protein tyrosine phosphatase, non-receptor type 4 [Source:HGNC Symbol;Acc:HGNC:9656]	992.4	805.0	**-1.23**	3.28E-07
HAT1	histone acetyltransferase 1 [Source:HGNC Symbol;Acc:HGNC:4821]	2586.3	2117.2	**-1.23**	3.48E-07

C12orf65	chromosome 12 open reading frame 65 [Source:HGNC Symbol;Acc:HGNC:26784]	478.9	387.5	**-1.23**	8.67E-06
INTS6L	integrator complex subunit 6 like [Source:HGNC Symbol;Acc:HGNC:27334]	645.8	521.7	**-1.23**	1.81E-05
ETV5	ETS variant 5 [Source:HGNC Symbol;Acc:HGNC:3494]	767.8	624.3	**-1.23**	2.05E-05
APEH	acylaminoacyl-peptide hydrolase [Source:HGNC Symbol;Acc:HGNC:586]	710.4	575.2	**-1.23**	2.08E-05
NIPAL3	NIPA like domain containing 3 [Source:HGNC Symbol;Acc:HGNC:25233]	942.9	761.4	**-1.23**	3.10E-05
PTPRE	protein tyrosine phosphatase, receptor type E [Source:HGNC Symbol;Acc:HGNC:9669]	1727.9	1388.8	**-1.23**	4.96E-05
TBC1D8B	TBC1 domain family member 8B [Source:HGNC Symbol;Acc:HGNC:24715]	937.8	761.4	**-1.23**	5.71E-05
TFB1M	transcription factor B1, mitochondrial [Source:HGNC Symbol;Acc:HGNC:17037]	403.9	329.8	**-1.23**	7.79E-05
KAT6B	lysine acetyltransferase 6B [Source:HGNC Symbol;Acc:HGNC:17582]	678.0	549.9	**-1.23**	0.0001701
ANGPTL1	angiopoietin like 1 [Source:HGNC Symbol;Acc:HGNC:489]	326.5	263.9	**-1.23**	0.0003155
VLDLR	very low density lipoprotein receptor [Source:HGNC Symbol;Acc:HGNC:12698]	1069.6	884.0	**-1.23**	0.0003251
NCAPD3	non-SMC condensin II complex subunit D3 [Source:HGNC Symbol;Acc:HGNC:28952]	891.9	732.9	**-1.23**	0.0005222
MPDU1	mannose-P-dolichol utilization defect 1 [Source:HGNC Symbol;Acc:HGNC:7207]	406.4	329.4	**-1.23**	0.0008237
APIP	APAF1 interacting protein [Source:HGNC Symbol;Acc:HGNC:17581]	397.2	323.4	**-1.23**	0.0008823
ITGB3BP	integrin subunit beta 3 binding protein [Source:HGNC Symbol;Acc:HGNC:6157]	779.5	643.0	**-1.23**	0.0009663
RTTN	rotatin [Source:HGNC Symbol;Acc:HGNC:18654]	321.6	262.6	**-1.23**	0.001464
UBE2D4	ubiquitin conjugating enzyme E2 D4 (putative) [Source:HGNC Symbol;Acc:HGNC:21647]	349.6	284.0	**-1.23**	0.001781
CSMD2	CUB and Sushi multiple domains 2 [Source:HGNC Symbol;Acc:HGNC:19290]	324.6	263.9	**-1.23**	0.001941
LDB1	LIM domain binding 1 [Source:HGNC Symbol;Acc:HGNC:6532]	310.1	251.4	**-1.23**	0.001964
MTBP	MDM2 binding protein [Source:HGNC Symbol;Acc:HGNC:7417]	340.7	277.5	**-1.23**	0.002873
CAD	carbamoyl-phosphate synthetase 2, aspartate transcarbamylase, and dihydroorotase [Source:HGNC Syr	594.0	484.5	**-1.23**	0.003275
PSTPIP2	proline-serine-threonine phosphatase interacting protein 2 [Source:HGNC Symbol;Acc:HGNC:9581]	333.4	269.0	**-1.23**	0.005716
TLR3	toll like receptor 3 [Source:HGNC Symbol;Acc:HGNC:11849]	220.8	180.9	**-1.23**	0.005879
NPRL2	NPR2 like, GATOR1 complex subunit [Source:HGNC Symbol;Acc:HGNC:24969]	185.8	150.3	**-1.23**	0.006428
KLF11	Kruppel like factor 11 [Source:HGNC Symbol;Acc:HGNC:11811]	228.1	185.9	**-1.23**	0.006611
ZBTB46	zinc finger and BTB domain containing 46 [Source:HGNC Symbol;Acc:HGNC:16094]	257.2	206.1	**-1.23**	0.008159
TRIM6	tripartite motif containing 6 [Source:HGNC Symbol;Acc:HGNC:16277]	164.5	133.6	**-1.23**	0.01408
XRCC3	X-ray repair cross complementing 3 [Source:HGNC Symbol;Acc:HGNC:12830]	150.4	122.4	**-1.23**	0.01902
TMTC4	transmembrane and tetratricopeptide repeat containing 4 [Source:HGNC Symbol;Acc:HGNC:25904]	179.0	144.6	**-1.23**	0.02053
WDYHV1	WDYHV motif containing 1 [Source:HGNC Symbol;Acc:HGNC:25490]	172.8	140.7	**-1.23**	0.02129
NDUFAF6	NADH:ubiquinone oxidoreductase complex assembly factor 6 [Source:HGNC Symbol;Acc:HGNC:28625]	226.0	183.5	**-1.23**	0.02569
CYB5A	cytochrome b5 type A [Source:HGNC Symbol;Acc:HGNC:2570]	251.8	207.3	**-1.23**	0.02823
TTLL3	tubulin tyrosine ligase like 3 [Source:HGNC Symbol;Acc:HGNC:24483]	145.9	118.5	**-1.23**	0.03015
WDFY3-AS2	WDFY3 antisense RNA 2 [Source:HGNC Symbol;Acc:HGNC:21603]	214.4	176.2	**-1.23**	0.03149
SLC7A5	solute carrier family 7 member 5 [Source:HGNC Symbol;Acc:HGNC:11063]	615.2	507.4	**-1.23**	0.03193
FBXO2	F-box protein 2 [Source:HGNC Symbol;Acc:HGNC:13581]	442.0	355.0	**-1.23**	0.03384
DCPS	decapping enzyme, scavenger [Source:HGNC Symbol;Acc:HGNC:29812]	109.7	89.6	**-1.23**	0.03401
KIF24	kinesin family member 24 [Source:HGNC Symbol;Acc:HGNC:19916]	150.8	121.4	**-1.23**	0.04056
PTGES	prostaglandin E synthase [Source:HGNC Symbol;Acc:HGNC:9599]	298.9	242.1	**-1.23**	0.04056
KRR1	KRR1, small subunit processome component homolog [Source:HGNC Symbol;Acc:HGNC:5176]	4189.3	3397.8	**-1.24**	1.51E-14
XPOT	exportin for tRNA [Source:HGNC Symbol;Acc:HGNC:12826]	7925.5	6360.0	**-1.24**	1.82E-14
HMGB1	high mobility group box 1 [Source:HGNC Symbol;Acc:HGNC:4983]	9309.8	7511.3	**-1.24**	2.77E-13
GALNT10	polypeptide N-acetylgalactosaminyltransferase 10 [Source:HGNC Symbol;Acc:HGNC:19873]	4205.6	3403.4	**-1.24**	6.65E-11
MLEC	malectin [Source:HGNC Symbol;Acc:HGNC:28973]	7633.1	6148.0	**-1.24**	5.37E-10
ZRANB2	zinc finger RANBP2-type containing 2 [Source:HGNC Symbol;Acc:HGNC:13058]	2853.3	2312.7	**-1.24**	9.89E-10
MFSD11	major facilitator superfamily domain containing 11 [Source:HGNC Symbol;Acc:HGNC:25458]	1249.9	1008.7	**-1.24**	2.21E-09
ATP2B1	ATPase plasma membrane Ca2+ transporting 1 [Source:HGNC Symbol;Acc:HGNC:814]	6323.9	5036.3	**-1.24**	1.55E-07
APOL2	apolipoprotein L2 [Source:HGNC Symbol;Acc:HGNC:619]	862.6	696.2	**-1.24**	1.68E-07
ENPP1	ectonucleotide pyrophosphatase/phosphodiesterase 1 [Source:HGNC Symbol;Acc:HGNC:3356]	13986.8	11315.6	**-1.24**	6.95E-07
PPIL3	peptidylprolyl isomerase like 3 [Source:HGNC Symbol;Acc:HGNC:9262]	733.5	587.0	**-1.24**	4.83E-06
ENOPH1	enolase-phosphatase 1 [Source:HGNC Symbol;Acc:HGNC:24599]	1017.9	824.0	**-1.24**	5.04E-06
ECHS1	enoyl-CoA hydratase, short chain 1 [Source:HGNC Symbol;Acc:HGNC:3151]	827.8	669.4	**-1.24**	5.89E-06
IFRD1	interferon related developmental regulator 1 [Source:HGNC Symbol;Acc:HGNC:5456]	2055.1	1647.1	**-1.24**	7.77E-06
SLC3A1	solute carrier family 3 member 1 [Source:HGNC Symbol;Acc:HGNC:11025]	768.9	621.5	**-1.24**	9.04E-06
HIBCH	3-hydroxyisobutyryl-CoA hydrolase [Source:HGNC Symbol;Acc:HGNC:4908]	549.2	442.4	**-1.24**	2.38E-05
EPB41L2	erythrocyte membrane protein band 4.1 like 2 [Source:HGNC Symbol;Acc:HGNC:3379]	5441.7	4391.8	**-1.24**	2.83E-05
SNRPF	small nuclear ribonucleoprotein polypeptide F [Source:HGNC Symbol;Acc:HGNC:11162]	1297.5	1056.1	**-1.24**	3.16E-05
CALD1	caldesmon 1 [Source:HGNC Symbol;Acc:HGNC:1441]	5852.0	4719.3	**-1.24**	5.88E-05
TAF1A	TATA-box binding protein associated factor, RNA polymerase I subunit A [Source:HGNC Symbol;Acc:HGI	525.0	422.4	**-1.24**	6.07E-05
ZNF248	zinc finger protein 248 [Source:HGNC Symbol;Acc:HGNC:13041]	388.7	313.6	**-1.24**	6.80E-05
SLC39A11	solute carrier family 39 member 11 [Source:HGNC Symbol;Acc:HGNC:14463]	377.0	302.4	**-1.24**	0.0001402
FAM173B	family with sequence similarity 173 member B [Source:HGNC Symbol;Acc:HGNC:27029]	441.8	357.4	**-1.24**	0.0002635
PPAT	phosphoribosyl pyrophosphate amidotransferase [Source:HGNC Symbol;Acc:HGNC:9238]	486.8	396.9	**-1.24**	0.000435
CCDC171	coiled-coil domain containing 171 [Source:HGNC Symbol;Acc:HGNC:29828]	323.0	259.7	**-1.24**	0.0006963
EEF1AKMT1	EEF1A lysine methyltransferase 1 [Source:HGNC Symbol;Acc:HGNC:27351]	283.6	229.3	**-1.24**	0.0007936
TP53I13	tumor protein p53 inducible protein 13 [Source:HGNC Symbol;Acc:HGNC:25102]	281.2	225.9	**-1.24**	0.0008136
RIPK2	receptor interacting serine/threonine kinase 2 [Source:HGNC Symbol;Acc:HGNC:10020]	410.8	330.7	**-1.24**	0.001129
KALRN	kalirin RhoGEF kinase [Source:HGNC Symbol;Acc:HGNC:4814]	1593.9	1290.2	**-1.24**	0.001229
ELP6	elongator acetyltransferase complex subunit 6 [Source:HGNC Symbol;Acc:HGNC:25976]	320.2	259.3	**-1.24**	0.001537
RGPD8	RANBP2-like and GRIP domain containing 8 [Source:HGNC Symbol;Acc:HGNC:9849]	302.2	242.0	**-1.24**	0.001774
TMEM231	transmembrane protein 231 [Source:HGNC Symbol;Acc:HGNC:37234]	197.8	159.7	**-1.24**	0.00261
GPX7	glutathione peroxidase 7 [Source:HGNC Symbol;Acc:HGNC:4559]	212.2	170.7	**-1.24**	0.002617

STX16-NPEPL1	STX16-NPEPL1 readthrough (NMD candidate) [Source:HGNC Symbol;Acc:HGNC:41993]	250.3	201.5	**-1.24**	0.003714
GSE1	Gse1 coiled-coil protein [Source:HGNC Symbol;Acc:HGNC:28979]	295.4	237.6	**-1.24**	0.003878
GCA	grancalcin [Source:HGNC Symbol;Acc:HGNC:15990]	193.5	156.4	**-1.24**	0.004073
SLC25A5-AS1	SLC25A5 antisense RNA 1 [Source:HGNC Symbol;Acc:HGNC:43438]	149.8	120.5	**-1.24**	0.005071
CHCHD4	coiled-coil-helix-coiled-coil-helix domain containing 4 [Source:HGNC Symbol;Acc:HGNC:26467]	271.8	219.0	**-1.24**	0.00517
ZKSCAN2	zinc finger with KRAB and SCAN domains 2 [Source:HGNC Symbol;Acc:HGNC:25677]	210.3	169.7	**-1.24**	0.01186
BCKDHA	branched chain keto acid dehydrogenase E1, alpha polypeptide [Source:HGNC Symbol;Acc:HGNC:986]	222.7	181.4	**-1.24**	0.01677
ZNF304	zinc finger protein 304 [Source:HGNC Symbol;Acc:HGNC:13505]	251.5	202.3	**-1.24**	0.01687
C17orf58	chromosome 17 open reading frame 58 [Source:HGNC Symbol;Acc:HGNC:27568]	168.8	136.0	**-1.24**	0.01862
SDHAF3	succinate dehydrogenase complex assembly factor 3 [Source:HGNC Symbol;Acc:HGNC:21752]	201.0	163.6	**-1.24**	0.02011
EFNB2	ephrin B2 [Source:HGNC Symbol;Acc:HGNC:3227]	204.2	170.9	**-1.24**	0.03338
NIPSNAP3B	nipsnap homolog 3B [Source:HGNC Symbol;Acc:HGNC:23641]	113.2	91.2	**-1.24**	0.03963
ZNF574	zinc finger protein 574 [Source:HGNC Symbol;Acc:HGNC:26166]	100.1	81.4	**-1.24**	0.04854
C8orf37	chromosome 8 open reading frame 37 [Source:HGNC Symbol;Acc:HGNC:27232]	110.7	88.6	**-1.24**	0.0499
ZEB1	zinc finger E-box binding homeobox 1 [Source:HGNC Symbol;Acc:HGNC:11642]	5378.6	4315.5	**-1.25**	7.59E-21
HSPA13	heat shock protein family A (Hsp70) member 13 [Source:HGNC Symbol;Acc:HGNC:11375]	5158.8	4119.2	**-1.25**	5.33E-19
LRP11	LDL receptor related protein 11 [Source:HGNC Symbol;Acc:HGNC:16936]	2388.9	1916.0	**-1.25**	4.57E-16
FAM177A1	family with sequence similarity 177 member A1 [Source:HGNC Symbol;Acc:HGNC:19829]	3353.3	2683.5	**-1.25**	4.31E-15
SLC39A7	solute carrier family 39 member 7 [Source:HGNC Symbol;Acc:HGNC:4927]	4931.9	3981.9	**-1.25**	1.88E-14
PRKCA	protein kinase C alpha [Source:HGNC Symbol;Acc:HGNC:9393]	2922.7	2344.7	**-1.25**	1.45E-12
TXNDC5	thioredoxin domain containing 5 [Source:HGNC Symbol;Acc:HGNC:21073]	9110.4	7300.1	**-1.25**	6.19E-11
EML4	echinoderm microtubule associated protein like 4 [Source:HGNC Symbol;Acc:HGNC:1316]	1152.5	922.4	**-1.25**	4.71E-10
PSMD10	proteasome 26S subunit, non-ATPase 10 [Source:HGNC Symbol;Acc:HGNC:9555]	1335.4	1064.6	**-1.25**	6.91E-09
PLOD1	procollagen-lysine,2-oxoglutarate 5-dioxygenase 1 [Source:HGNC Symbol;Acc:HGNC:9081]	2526.6	2022.3	**-1.25**	1.07E-08
FTL	ferritin light chain [Source:HGNC Symbol;Acc:HGNC:3999]	12123.3	9792.0	**-1.25**	1.03E-07
SRXN1	sulfiredoxin 1 [Source:HGNC Symbol;Acc:HGNC:16132]	1127.7	913.0	**-1.25**	6.16E-07
CLVS1	clavesin 1 [Source:HGNC Symbol;Acc:HGNC:23139]	835.8	665.7	**-1.25**	6.72E-07
BLOC1S5	biogenesis of lysosomal organelles complex 1 subunit 5 [Source:HGNC Symbol;Acc:HGNC:18561]	1229.5	984.3	**-1.25**	7.19E-07
GSTM3	glutathione S-transferase mu 3 [Source:HGNC Symbol;Acc:HGNC:4635]	669.0	536.5	**-1.25**	8.69E-07
MSH6	mutS homolog 6 [Source:HGNC Symbol;Acc:HGNC:7329]	3859.1	3116.2	**-1.25**	1.43E-06
LYSMD4	LysM domain containing 4 [Source:HGNC Symbol;Acc:HGNC:26571]	453.3	363.3	**-1.25**	3.74E-06
BTN2A1	butyrophilin subfamily 2 member A1 [Source:HGNC Symbol;Acc:HGNC:1136]	590.5	472.1	**-1.25**	4.69E-06
ZNF436	zinc finger protein 436 [Source:HGNC Symbol;Acc:HGNC:20814]	626.5	499.7	**-1.25**	9.03E-06
TBL1X	transducin beta like 1 X-linked [Source:HGNC Symbol;Acc:HGNC:11585]	509.3	403.9	**-1.25**	9.09E-06
PRKRIP1	PRKR interacting protein 1 [Source:HGNC Symbol;Acc:HGNC:21894]	435.2	349.2	**-1.25**	1.06E-05
GTDC1	glycosyltransferase like domain containing 1 [Source:HGNC Symbol;Acc:HGNC:20887]	572.0	456.4	**-1.25**	1.09E-05
CYC1	cytochrome c1 [Source:HGNC Symbol;Acc:HGNC:2579]	658.9	528.7	**-1.25**	1.13E-05
GLIS3	GLIS family zinc finger 3 [Source:HGNC Symbol;Acc:HGNC:28510]	420.3	334.9	**-1.25**	1.36E-05
ABHD5	abhydrolase domain containing 5 [Source:HGNC Symbol;Acc:HGNC:21396]	407.3	324.1	**-1.25**	3.05E-05
PPM1F	protein phosphatase, Mg2+/Mn2+ dependent 1F [Source:HGNC Symbol;Acc:HGNC:19388]	407.5	327.0	**-1.25**	3.92E-05
RBM4B	RNA binding motif protein 4B [Source:HGNC Symbol;Acc:HGNC:28842]	376.2	300.7	**-1.25**	7.15E-05
CHPF	chondroitin polymerizing factor [Source:HGNC Symbol;Acc:HGNC:24291]	1211.1	977.1	**-1.25**	8.22E-05
DAB2	DAB2, clathrin adaptor protein [Source:HGNC Symbol;Acc:HGNC:2662]	5175.3	4063.6	**-1.25**	8.54E-05
HINT3	histidine triad nucleotide binding protein 3 [Source:HGNC Symbol;Acc:HGNC:18468]	663.2	532.9	**-1.25**	0.0001565
SEMA4F	ssemaphorin 4F [Source:HGNC Symbol;Acc:HGNC:10734]	255.0	203.6	**-1.25**	0.0001827
SLC25A11	solute carrier family 25 member 11 [Source:HGNC Symbol;Acc:HGNC:10981]	372.1	299.0	**-1.25**	0.0002285
SLC25A26	solute carrier family 25 member 26 [Source:HGNC Symbol;Acc:HGNC:20661]	514.5	416.5	**-1.25**	0.000293
TACC2	transforming acidic coiled-coil containing protein 2 [Source:HGNC Symbol;Acc:HGNC:11523]	395.7	317.5	**-1.25**	0.0003165
REL	REL proto-oncogene, NF-kB subunit [Source:HGNC Symbol;Acc:HGNC:9954]	328.9	262.5	**-1.25**	0.000602
STEAP2	STEAP2 metalloreductase [Source:HGNC Symbol;Acc:HGNC:17885]	1074.9	866.8	**-1.25**	0.0006843
ARHGEF10L	Rho guanine nucleotide exchange factor 10 like [Source:HGNC Symbol;Acc:HGNC:25540]	248.7	198.0	**-1.25**	0.0007013
ZFP1	ZFP1 zinc finger protein [Source:HGNC Symbol;Acc:HGNC:23328]	355.8	285.6	**-1.25**	0.0009346
FAM214B	family with sequence similarity 214 member B [Source:HGNC Symbol;Acc:HGNC:25666]	242.9	195.0	**-1.25**	0.001299
TEX264	testis expressed 264 [Source:HGNC Symbol;Acc:HGNC:30247]	463.3	369.9	**-1.25**	0.00141
RFC2	replication factor C subunit 2 [Source:HGNC Symbol;Acc:HGNC:9970]	398.9	321.4	**-1.25**	0.001802
IDH2	isocitrate dehydrogenase (NADP(+)) 2, mitochondrial [Source:HGNC Symbol;Acc:HGNC:5383]	583.1	466.4	**-1.25**	0.002118
NCOA7	nuclear receptor coactivator 7 [Source:HGNC Symbol;Acc:HGNC:21081]	963.8	756.3	**-1.25**	0.00215
ZCCHC4	zinc finger CCHC-type containing 4 [Source:HGNC Symbol;Acc:HGNC:22917]	283.4	226.2	**-1.25**	0.00226
BOLA3	bolA family member 3 [Source:HGNC Symbol;Acc:HGNC:24415]	523.1	425.3	**-1.25**	0.002945
SKP2	S-phase kinase associated protein 2 [Source:HGNC Symbol;Acc:HGNC:10901]	385.7	313.6	**-1.25**	0.004268
ZFP64	ZFP64 zinc finger protein [Source:HGNC Symbol;Acc:HGNC:15940]	211.1	168.9	**-1.25**	0.005501
TTC30A	tetratricopeptide repeat domain 30A [Source:HGNC Symbol;Acc:HGNC:25853]	430.3	349.1	**-1.25**	0.006667
SETMAR	SET domain and mariner transposase fusion gene [Source:HGNC Symbol;Acc:HGNC:10762]	181.0	145.3	**-1.25**	0.007187
ZNF43	zinc finger protein 43 [Source:HGNC Symbol;Acc:HGNC:13109]	247.5	199.7	**-1.25**	0.009843
MTSS1L	MTSS1L, I-BAR domain containing [Source:HGNC Symbol;Acc:HGNC:25094]	339.8	265.5	**-1.25**	0.01171
GPR27	G protein-coupled receptor 27 [Source:HGNC Symbol;Acc:HGNC:4482]	141.2	114.1	**-1.25**	0.01571
PPT2	palmitoyl-protein thioesterase 2 [Source:HGNC Symbol;Acc:HGNC:9326]	201.7	159.8	**-1.25**	0.0189
ELOVL6	ELOVL fatty acid elongase 6 [Source:HGNC Symbol;Acc:HGNC:15829]	342.8	281.5	**-1.25**	0.02092
ACOX3	acyl-CoA oxidase 3, pristanoyl [Source:HGNC Symbol;Acc:HGNC:121]	153.8	123.0	**-1.25**	0.02366
BMP2	bone morphogenetic protein 2 [Source:HGNC Symbol;Acc:HGNC:1069]	8588.5	6758.4	**-1.25**	0.02446
BAX	BCL2 associated X, apoptosis regulator [Source:HGNC Symbol;Acc:HGNC:959]	112.8	89.5	**-1.25**	0.02451
PRAP1	proline rich acidic protein 1 [Source:HGNC Symbol;Acc:HGNC:23304]	150.3	119.0	**-1.25**	0.02696

FILIP1	filamin A interacting protein 1 [Source:HGNC Symbol;Acc:HGNC:21015]	130.5	103.8	**-1.25**	0.04927
NUCKS1	nuclear casein kinase and cyclin dependent kinase substrate 1 [Source:HGNC Symbol;Acc:HGNC:29923]	11912.2	9493.3	**-1.26**	2.04E-18
TMEM30A	transmembrane protein 30A [Source:HGNC Symbol;Acc:HGNC:16667]	11953.0	9524.1	**-1.26**	5.09E-18
PREPL	prolyl endopeptidase like [Source:HGNC Symbol;Acc:HGNC:30228]	3042.4	2423.6	**-1.26**	1.94E-16
SRPX2	sushi repeat containing protein X-linked 2 [Source:HGNC Symbol;Acc:HGNC:30668]	4875.0	3901.4	**-1.26**	4.56E-15
ARRDC3	arrestin domain containing 3 [Source:HGNC Symbol;Acc:HGNC:29263]	9935.0	7922.5	**-1.26**	2.26E-14
ERLIN1	ER lipid raft associated 1 [Source:HGNC Symbol;Acc:HGNC:16947]	1754.2	1397.7	**-1.26**	1.01E-12
BLOC1S5-TXNDC5	BLOC1S5-TXNDC5 readthrough (NMD candidate) [Source:HGNC Symbol;Acc:HGNC:42001]	9739.4	7750.8	**-1.26**	6.59E-12
TPM2	tropomyosin 2 [Source:HGNC Symbol;Acc:HGNC:12011]	2723.2	2173.5	**-1.26**	8.75E-12
NRK	Nik related kinase [Source:HGNC Symbol;Acc:HGNC:25391]	1736.5	1386.7	**-1.26**	3.46E-11
POFUT2	protein O-fucosyltransferase 2 [Source:HGNC Symbol;Acc:HGNC:14683]	1269.9	1011.8	**-1.26**	6.23E-11
IDH1	isocitrate dehydrogenase (NADP(+)) 1, cytosolic [Source:HGNC Symbol;Acc:HGNC:5382]	4134.3	3294.5	**-1.26**	1.54E-10
TMX1	thioredoxin related transmembrane protein 1 [Source:HGNC Symbol;Acc:HGNC:15487]	4432.2	3541.2	**-1.26**	8.96E-10
NSL1	NSL1, MIS12 kinetochore complex component [Source:HGNC Symbol;Acc:HGNC:24548]	729.6	579.6	**-1.26**	2.48E-09
PIEZO1	piezo type mechanosensitive ion channel component 1 [Source:HGNC Symbol;Acc:HGNC:28993]	3110.6	2483.5	**-1.26**	2.73E-09
MLH1	mutL homolog 1 [Source:HGNC Symbol;Acc:HGNC:7127]	1852.9	1479.7	**-1.26**	4.09E-09
MOCS2	molybdenum cofactor synthesis 2 [Source:HGNC Symbol;Acc:HGNC:7193]	972.7	775.2	**-1.26**	4.45E-09
P3H1	prolyl 3-hydroxylase 1 [Source:HGNC Symbol;Acc:HGNC:19316]	1553.8	1234.1	**-1.26**	8.82E-09
GJA1	gap junction protein alpha 1 [Source:HGNC Symbol;Acc:HGNC:4274]	8301.7	6606.2	**-1.26**	2.86E-08
UXS1	UDP-glucuronate decarboxylase 1 [Source:HGNC Symbol;Acc:HGNC:17729]	2360.9	1901.9	**-1.26**	3.10E-08
KIAA1841	KIAA1841 [Source:HGNC Symbol;Acc:HGNC:29387]	771.6	612.1	**-1.26**	2.13E-07
COPS6	COP9 signalosome subunit 6 [Source:HGNC Symbol;Acc:HGNC:21749]	770.1	612.1	**-1.26**	2.48E-07
ECI2	enoyl-CoA delta isomerase 2 [Source:HGNC Symbol;Acc:HGNC:14601]	1055.4	830.1	**-1.26**	3.47E-07
ZSCAN30	zinc finger and SCAN domain containing 30 [Source:HGNC Symbol;Acc:HGNC:33517]	866.6	690.8	**-1.26**	3.93E-07
POMGNT1	protein O-linked mannose N-acetylglucosaminyltransferase 1 (beta 1,2-) [Source:HGNC Symbol;Acc:HGN	953.9	754.4	**-1.26**	9.22E-07
TRIP6	thyroid hormone receptor interactor 6 [Source:HGNC Symbol;Acc:HGNC:12311]	841.3	669.7	**-1.26**	1.71E-06
OSGIN2	oxidative stress induced growth inhibitor family member 2 [Source:HGNC Symbol;Acc:HGNC:1355]	1621.7	1270.8	**-1.26**	2.01E-06
RFWD3	ring finger and WD repeat domain 3 [Source:HGNC Symbol;Acc:HGNC:25539]	895.5	716.2	**-1.26**	2.55E-06
COQ7	coenzyme Q7, hydroxylase [Source:HGNC Symbol;Acc:HGNC:2244]	434.2	344.4	**-1.26**	2.58E-06
LMNB2	lamin B2 [Source:HGNC Symbol;Acc:HGNC:6638]	1687.6	1345.8	**-1.26**	5.13E-06
TNS2	tensin 2 [Source:HGNC Symbol;Acc:HGNC:19737]	885.8	698.1	**-1.26**	5.18E-05
ERI2	ERI1 exoribonuclease family member 2 [Source:HGNC Symbol;Acc:HGNC:30541]	497.1	392.8	**-1.26**	0.0001017
MEIS2	Meis homeobox 2 [Source:HGNC Symbol;Acc:HGNC:7001]	250.8	198.9	**-1.26**	0.0003298
PIP4P2	phosphatidylinositol-4,5-bisphosphate 4-phosphatase 2 [Source:HGNC Symbol;Acc:HGNC:25452]	393.2	314.1	**-1.26**	0.000334
SCFD2	sec1 family domain containing 2 [Source:HGNC Symbol;Acc:HGNC:30676]	490.0	394.7	**-1.26**	0.0005707
GPSM1	G protein signaling modulator 1 [Source:HGNC Symbol;Acc:HGNC:17858]	597.4	486.8	**-1.26**	0.0006965
ZNF738	zinc finger protein 738 [Source:HGNC Symbol;Acc:HGNC:32469]	256.8	204.4	**-1.26**	0.0007187
LRRC8C	leucine rich repeat containing 8 VRAC subunit C [Source:HGNC Symbol;Acc:HGNC:25075]	286.7	232.3	**-1.26**	0.0009507
YPEL2	yippee like 2 [Source:HGNC Symbol;Acc:HGNC:18326]	792.4	631.1	**-1.26**	0.001068
DCAF4	DDB1 and CUL4 associated factor 4 [Source:HGNC Symbol;Acc:HGNC:20229]	293.3	233.9	**-1.26**	0.001076
HYI	hydroxypyruvate isomerase (putative) [Source:HGNC Symbol;Acc:HGNC:26948]	326.8	260.8	**-1.26**	0.001365
HOXA13	homeobox A13 [Source:HGNC Symbol;Acc:HGNC:5102]	215.1	171.6	**-1.26**	0.002113
CC2D1A	coiled-coil and C2 domain containing 1A [Source:HGNC Symbol;Acc:HGNC:30237]	224.0	178.3	**-1.26**	0.002493
ZNF793	zinc finger protein 793 [Source:HGNC Symbol;Acc:HGNC:33115]	196.3	155.4	**-1.26**	0.003838
MGMT	O-6-methylguanine-DNA methyltransferase [Source:HGNC Symbol;Acc:HGNC:7059]	170.9	134.7	**-1.26**	0.005146
C12orf66	chromosome 12 open reading frame 66 [Source:HGNC Symbol;Acc:HGNC:26517]	226.3	181.3	**-1.26**	0.005819
METTL4	methyltransferase like 4 [Source:HGNC Symbol;Acc:HGNC:24726]	253.8	204.4	**-1.26**	0.008419
NXT1	nuclear transport factor 2 like export factor 1 [Source:HGNC Symbol;Acc:HGNC:15913]	147.6	116.4	**-1.26**	0.01507
C2	complement C2 [Source:HGNC Symbol;Acc:HGNC:1248]	105.2	84.4	**-1.26**	0.01886
LYRM9	LYR motif containing 9 [Source:HGNC Symbol;Acc:HGNC:27314]	150.4	119.2	**-1.26**	0.01926
LTBP4	latent transforming growth factor beta binding protein 4 [Source:HGNC Symbol;Acc:HGNC:6717]	167.4	132.6	**-1.26**	0.02048
POMGNT2	protein O-linked mannose N-acetylglucosaminyltransferase 2 (beta 1,4-) [Source:HGNC Symbol;Acc:HGN	110.3	87.3	**-1.26**	0.02159
HSPA5	heat shock protein family A (Hsp70) member 5 [Source:HGNC Symbol;Acc:HGNC:5238]	27246.1	21847.4	**-1.26**	0.02461
FAM98C	family with sequence similarity 98 member C [Source:HGNC Symbol;Acc:HGNC:27119]	78.2	61.5	**-1.26**	0.02945
HAUS5	HAUS augmin like complex subunit 5 [Source:HGNC Symbol;Acc:HGNC:29130]	160.7	128.6	**-1.26**	0.04004
LINC00476	long intergenic non-protein coding RNA 476 [Source:HGNC Symbol;Acc:HGNC:27858]	86.9	68.2	**-1.26**	0.04649
RDH11	retinol dehydrogenase 11 [Source:HGNC Symbol;Acc:HGNC:17964]	2995.9	2371.7	**-1.27**	2.75E-16
ACADVL	acyl-CoA dehydrogenase very long chain [Source:HGNC Symbol;Acc:HGNC:92]	3674.9	2904.4	**-1.27**	8.58E-16
HSP90AA1	heat shock protein 90 alpha family class A member 1 [Source:HGNC Symbol;Acc:HGNC:5253]	43105.3	34252.6	**-1.27**	6.28E-15
CNIH1	cornichon family AMPA receptor auxiliary protein 1 [Source:HGNC Symbol;Acc:HGNC:19431]	2876.2	2264.5	**-1.27**	1.10E-14
CMAHP	cytidine monophospho-N-acetylneuraminic acid hydroxylase, pseudogene [Source:HGNC Symbol;Acc:H	2887.8	2273.4	**-1.27**	1.82E-14
RCN2	reticulocalbin 2 [Source:HGNC Symbol;Acc:HGNC:9935]	5142.3	4056.1	**-1.27**	7.66E-13
PTPN14	protein tyrosine phosphatase, non-receptor type 14 [Source:HGNC Symbol;Acc:HGNC:9647]	4722.5	3738.2	**-1.27**	3.52E-12
ASPH	aspartate beta-hydroxylase [Source:HGNC Symbol;Acc:HGNC:757]	15424.4	12048.6	**-1.27**	6.29E-12
CWC22	CWC22 spliceosome associated protein homolog [Source:HGNC Symbol;Acc:HGNC:29322]	1617.1	1280.7	**-1.27**	6.02E-11
ZFAND1	zinc finger AN1-type containing 1 [Source:HGNC Symbol;Acc:HGNC:25858]	957.8	758.8	**-1.27**	6.72E-10
PLCE1	phospholipase C epsilon 1 [Source:HGNC Symbol;Acc:HGNC:17175]	758.9	598.0	**-1.27**	1.15E-08
HYPK	huntingtin interacting protein K [Source:HGNC Symbol;Acc:HGNC:18418]	1064.7	840.9	**-1.27**	4.20E-08
ARHGDIB	Rho GDP dissociation inhibitor beta [Source:HGNC Symbol;Acc:HGNC:679]	916.8	704.3	**-1.27**	2.22E-07
MAGI1	membrane associated guanylate kinase, WW and PDZ domain containing 1 [Source:HGNC Symbol;Acc:H	1123.0	886.1	**-1.27**	4.26E-07
GDAP1	ganglioside induced differentiation associated protein 1 [Source:HGNC Symbol;Acc:HGNC:15968]	641.2	509.4	**-1.27**	1.44E-06
COPRS	coordinator of PRMT5 and differentiation stimulator [Source:HGNC Symbol;Acc:HGNC:28848]	1030.5	816.5	**-1.27**	2.57E-06

ARID1B	AT-rich interaction domain 1B [Source:HGNC Symbol;Acc:HGNC:18040]	1141.7	906.5	**-1.27**	7.49E-06
ZBTB14	zinc finger and BTB domain containing 14 [Source:HGNC Symbol;Acc:HGNC:12860]	496.7	392.3	**-1.27**	8.54E-06
SRSF2	serine and arginine rich splicing factor 2 [Source:HGNC Symbol;Acc:HGNC:10783]	1388.9	1113.4	**-1.27**	1.27E-05
SLC50A1	solute carrier family 50 member 1 [Source:HGNC Symbol;Acc:HGNC:30657]	327.1	257.9	**-1.27**	2.28E-05
ZMIZ1	zinc finger MIZ-type containing 1 [Source:HGNC Symbol;Acc:HGNC:16493]	802.4	629.6	**-1.27**	0.0001329
EXO5	exonuclease 5 [Source:HGNC Symbol;Acc:HGNC:26115]	241.8	191.2	**-1.27**	0.0005895
ANAPC15	anaphase promoting complex subunit 15 [Source:HGNC Symbol;Acc:HGNC:24531]	198.5	156.4	**-1.27**	0.0008605
KBTBD8	kelch repeat and BTB domain containing 8 [Source:HGNC Symbol;Acc:HGNC:30691]	349.0	271.5	**-1.27**	0.0009466
ALDH7A1	aldehyde dehydrogenase 7 family member A1 [Source:HGNC Symbol;Acc:HGNC:877]	315.3	249.3	**-1.27**	0.001275
CDKN2D	cyclin dependent kinase inhibitor 2D [Source:HGNC Symbol;Acc:HGNC:1790]	300.5	236.6	**-1.27**	0.001311
HOXA9	homeobox A9 [Source:HGNC Symbol;Acc:HGNC:5109]	195.7	154.1	**-1.27**	0.001457
SNRNP25	small nuclear ribonucleoprotein U11/U12 subunit 25 [Source:HGNC Symbol;Acc:HGNC:14161]	232.3	181.6	**-1.27**	0.001713
PRMT6	protein arginine methyltransferase 6 [Source:HGNC Symbol;Acc:HGNC:18241]	192.2	150.8	**-1.27**	0.002424
TBC1D9	TBC1 domain family member 9 [Source:HGNC Symbol;Acc:HGNC:21710]	403.8	325.5	**-1.27**	0.004163
BAIAP2	BAI1 associated protein 2 [Source:HGNC Symbol;Acc:HGNC:947]	206.2	162.1	**-1.27**	0.004236
EPHA2	EPH receptor A2 [Source:HGNC Symbol;Acc:HGNC:3386]	491.1	389.2	**-1.27**	0.005307
CEP128	centrosomal protein 128 [Source:HGNC Symbol;Acc:HGNC:20359]	236.3	187.5	**-1.27**	0.005348
DHX34	DExH-box helicase 34 [Source:HGNC Symbol;Acc:HGNC:16719]	170.5	134.0	**-1.27**	0.005596
FDX2	ferredoxin 2 [Source:HGNC Symbol;Acc:HGNC:30546]	182.4	143.1	**-1.27**	0.00872
NAP1L5	nucleosome assembly protein 1 like 5 [Source:HGNC Symbol;Acc:HGNC:19968]	223.3	175.7	**-1.27**	0.0126
MYORG	myogenesis regulating glycosidase (putative) [Source:HGNC Symbol;Acc:HGNC:19918]	248.7	199.8	**-1.27**	0.01738
RHBDD3	rhomboid domain containing 3 [Source:HGNC Symbol;Acc:HGNC:1308]	90.7	71.1	**-1.27**	0.02749
HELB	DNA helicase B [Source:HGNC Symbol;Acc:HGNC:17196]	202.5	163.4	**-1.27**	0.02895
PSMB10	proteasome subunit beta 10 [Source:HGNC Symbol;Acc:HGNC:9538]	92.6	73.1	**-1.27**	0.02999
ANKRD18EP	ankyrin repeat domain 18E, pseudogene [Source:HGNC Symbol;Acc:HGNC:43609]	90.5	71.1	**-1.27**	0.03613
SGMS1-AS1	SGMS1 antisense RNA 1 [Source:HGNC Symbol;Acc:HGNC:49683]	127.9	102.2	**-1.27**	0.04677
OSMR	oncostatin M receptor [Source:HGNC Symbol;Acc:HGNC:8507]	6696.3	5268.7	**-1.27**	1.19E-16
OGFRL1	opioid growth factor receptor like 1 [Source:HGNC Symbol;Acc:HGNC:21378]	2846.4	2236.7	**-1.27**	3.96E-14
PPFIBP1	PPFIA binding protein 1 [Source:HGNC Symbol;Acc:HGNC:9249]	11893.0	9278.4	**-1.27**	4.52E-14
NEO1	neogenin 1 [Source:HGNC Symbol;Acc:HGNC:7754]	1499.1	1170.6	**-1.27**	1.62E-11
MELTF	melanotransferrin [Source:HGNC Symbol;Acc:HGNC:7037]	3866.1	3026.4	**-1.27**	4.14E-11
PLXNB2	plexin B2 [Source:HGNC Symbol;Acc:HGNC:9104]	1587.0	1235.0	**-1.27**	5.28E-10
MRPL42	mitochondrial ribosomal protein L42 [Source:HGNC Symbol;Acc:HGNC:14493]	2097.9	1647.3	**-1.27**	6.82E-10
PHTF1	putative homeodomain transcription factor 1 [Source:HGNC Symbol;Acc:HGNC:8939]	937.0	736.0	**-1.27**	2.00E-09
LINC01578	long intergenic non-protein coding RNA 1578 [Source:HGNC Symbol;Acc:HGNC:48626]	1821.7	1422.5	**-1.27**	3.10E-09
FNBP1	formin binding protein 1 [Source:HGNC Symbol;Acc:HGNC:17069]	798.3	625.1	**-1.27**	3.23E-09
RIN2	Ras and Rab interactor 2 [Source:HGNC Symbol;Acc:HGNC:18750]	1102.6	873.6	**-1.27**	1.15E-08
NR1D2	nuclear receptor subfamily 1 group D member 2 [Source:HGNC Symbol;Acc:HGNC:7963]	4418.6	3461.7	**-1.27**	1.45E-08
INTS4	integrator complex subunit 4 [Source:HGNC Symbol;Acc:HGNC:25048]	639.6	505.2	**-1.27**	3.09E-08
AK4	adenylate kinase 4 [Source:HGNC Symbol;Acc:HGNC:363]	2334.3	1822.7	**-1.27**	3.20E-08
WARS	tryptophanyl-tRNA synthetase [Source:HGNC Symbol;Acc:HGNC:12729]	4334.8	3389.1	**-1.27**	5.12E-08
MPZL3	myelin protein zero like 3 [Source:HGNC Symbol;Acc:HGNC:27279]	606.2	476.2	**-1.27**	1.06E-07
ATP10A	ATPase phospholipid transporting 10A (putative) [Source:HGNC Symbol;Acc:HGNC:13542]	1092.8	857.7	**-1.27**	2.00E-06
TFDP1	transcription factor Dp-1 [Source:HGNC Symbol;Acc:HGNC:11749]	1338.2	1065.2	**-1.27**	2.49E-06
KPNA2	karyopherin subunit alpha 2 [Source:HGNC Symbol;Acc:HGNC:6395]	1041.9	830.7	**-1.27**	3.98E-06
PKD1	polycystin 1, transient receptor potential channel interacting [Source:HGNC Symbol;Acc:HGNC:9008]	851.1	665.7	**-1.27**	6.57E-06
HSPE1	heat shock protein family E (Hsp10) member 1 [Source:HGNC Symbol;Acc:HGNC:5269]	1935.6	1540.5	**-1.27**	1.90E-05
SMARCB1	SWI/SNF related, matrix associated, actin dependent regulator of chromatin, subfamily b, member 1 [Sc	569.6	444.6	**-1.27**	0.0001264
BANF1	barrier to autointegration factor 1 [Source:HGNC Symbol;Acc:HGNC:17397]	331.0	261.0	**-1.27**	0.0003935
SYCP2	synaptonemal complex protein 2 [Source:HGNC Symbol;Acc:HGNC:11490]	304.0	237.9	**-1.27**	0.001442
ARID1A	AT-rich interaction domain 1A [Source:HGNC Symbol;Acc:HGNC:11110]	365.9	286.3	**-1.27**	0.0022
ACKR3	atypical chemokine receptor 3 [Source:HGNC Symbol;Acc:HGNC:23692]	461.6	374.7	**-1.27**	0.002607
FAM174A	family with sequence similarity 174 member A [Source:HGNC Symbol;Acc:HGNC:24943]	213.5	168.2	**-1.27**	0.002923
PCDHB14	protocadherin beta 14 [Source:HGNC Symbol;Acc:HGNC:8685]	206.7	161.6	**-1.27**	0.003083
FGD1	FYVE, RhoGEF and PH domain containing 1 [Source:HGNC Symbol;Acc:HGNC:3663]	166.4	129.0	**-1.27**	0.004454
PTRHD1	peptidyl-tRNA hydrolase domain containing 1 [Source:HGNC Symbol;Acc:HGNC:33782]	161.3	125.6	**-1.27**	0.009004
RGS5	regulator of G protein signaling 5 [Source:HGNC Symbol;Acc:HGNC:10001]	136.8	105.6	**-1.27**	0.009993
PIWIL2	piwi like RNA-mediated gene silencing 2 [Source:HGNC Symbol;Acc:HGNC:17644]	170.5	127.3	**-1.27**	0.01022
CBR3-AS1	CBR3 antisense RNA 1 [Source:HGNC Symbol;Acc:HGNC:43664]	140.3	110.7	**-1.27**	0.01123
RPL23AP7	ribosomal protein L23a pseudogene 7 [Source:HGNC Symbol;Acc:HGNC:17336]	171.6	132.6	**-1.27**	0.01241
MRGBP	MRG domain binding protein [Source:HGNC Symbol;Acc:HGNC:15866]	159.5	124.1	**-1.27**	0.02183
ABHD14A-ACY1	ABHD14A-ACY1 readthrough [Source:HGNC Symbol;Acc:HGNC:38856]	117.8	92.1	**-1.27**	0.02276
EXOC6	exocyst complex component 6 [Source:HGNC Symbol;Acc:HGNC:23196]	106.0	83.7	**-1.27**	0.02772
ANKMY1	ankyrin repeat and MYND domain containing 1 [Source:HGNC Symbol;Acc:HGNC:20987]	95.9	75.5	**-1.27**	0.02884
AFMID	arylformamidase [Source:HGNC Symbol;Acc:HGNC:20910]	133.2	105.2	**-1.27**	0.02976
MACC1	MACC1, MET transcriptional regulator [Source:HGNC Symbol;Acc:HGNC:30215]	153.8	115.2	**-1.27**	0.03318
KRBOX1	KRAB box domain containing 1 [Source:HGNC Symbol;Acc:HGNC:38708]	110.8	87.1	**-1.27**	0.0353
C19orf66	chromosome 19 open reading frame 66 [Source:HGNC Symbol;Acc:HGNC:25649]	102.3	80.2	**-1.27**	0.03533
OPN1SW	opsin 1, short wave sensitive [Source:HGNC Symbol;Acc:HGNC:1012]	102.7	80.1	**-1.27**	0.03558
TEX41	testis expressed 41 (non-protein coding) [Source:HGNC Symbol;Acc:HGNC:48667]	81.4	62.9	**-1.27**	0.03721
HOXA5	homeobox A5 [Source:HGNC Symbol;Acc:HGNC:5106]	81.8	64.3	**-1.27**	0.03943
ARNTL2-AS1	ARNTL2 antisense RNA 1 [Source:HGNC Symbol;Acc:HGNC:49892]	102.1	79.5	**-1.27**	0.04724

RBBP7	RB binding protein 7, chromatin remodeling factor [Source:HGNC Symbol;Acc:HGNC:9890]	2722.6	2123.7	**-1.28**	4.86E-17
TCEA1	transcription elongation factor A1 [Source:HGNC Symbol;Acc:HGNC:11612]	5524.1	4277.5	**-1.28**	3.84E-16
ALKBH5	alkB homolog 5, RNA demethylase [Source:HGNC Symbol;Acc:HGNC:25996]	2169.1	1681.7	**-1.28**	2.62E-14
SNAPC3	small nuclear RNA activating complex polypeptide 3 [Source:HGNC Symbol;Acc:HGNC:11136]	1372.5	1066.0	**-1.28**	1.46E-13
USP11	ubiquitin specific peptidase 11 [Source:HGNC Symbol;Acc:HGNC:12609]	1246.2	972.8	**-1.28**	2.53E-10
CETN3	centrin 3 [Source:HGNC Symbol;Acc:HGNC:1868]	1231.6	961.8	**-1.28**	2.28E-09
ICK	intestinal cell kinase [Source:HGNC Symbol;Acc:HGNC:21219]	764.8	592.6	**-1.28**	2.53E-09
NDC1	NDC1 transmembrane nucleoporin [Source:HGNC Symbol;Acc:HGNC:25525]	1288.6	1012.7	**-1.28**	3.82E-09
SUMO3	small ubiquitin-like modifier 3 [Source:HGNC Symbol;Acc:HGNC:11124]	1410.8	1100.2	**-1.28**	7.76E-09
PDCL3	phosducin like 3 [Source:HGNC Symbol;Acc:HGNC:28860]	516.1	401.8	**-1.28**	8.81E-08
NUDT6	nudix hydrolase 6 [Source:HGNC Symbol;Acc:HGNC:8053]	1481.8	1158.5	**-1.28**	9.95E-08
MICA	MHC class I polypeptide-related sequence A [Source:HGNC Symbol;Acc:HGNC:7090]	1187.8	932.1	**-1.28**	1.12E-07
B4GALT2	beta-1,4-galactosyltransferase 2 [Source:HGNC Symbol;Acc:HGNC:925]	783.3	611.2	**-1.28**	1.75E-07
LDHB	lactate dehydrogenase B [Source:HGNC Symbol;Acc:HGNC:6541]	4614.2	3630.3	**-1.28**	1.74E-06
SGO2	shugoshin 2 [Source:HGNC Symbol;Acc:HGNC:30812]	655.8	515.3	**-1.28**	9.04E-06
HSPB11	heat shock protein family B (small) member 11 [Source:HGNC Symbol;Acc:HGNC:25019]	455.1	357.6	**-1.28**	2.79E-05
NEMP2	nuclear envelope integral membrane protein 2 [Source:HGNC Symbol;Acc:HGNC:33700]	253.6	197.2	**-1.28**	4.42E-05
ING4	inhibitor of growth family member 4 [Source:HGNC Symbol;Acc:HGNC:19423]	260.7	202.1	**-1.28**	0.0002144
CCDC144CP	coiled-coil domain containing 144C, pseudogene [Source:HGNC Symbol;Acc:HGNC:29073]	237.8	183.7	**-1.28**	0.0002736
MIR497HG	mir-497-195 cluster host gene [Source:HGNC Symbol;Acc:HGNC:39523]	229.1	179.8	**-1.28**	0.001195
FMO4	flavin containing monooxygenase 4 [Source:HGNC Symbol;Acc:HGNC:3772]	245.9	191.6	**-1.28**	0.001585
PRIM2	DNA primase subunit 2 [Source:HGNC Symbol;Acc:HGNC:9370]	487.6	385.0	**-1.28**	0.002044
PXN-AS1	PXN antisense RNA 1 [Source:HGNC Symbol;Acc:HGNC:44123]	152.9	119.2	**-1.28**	0.002718
PTGER2	prostaglandin E receptor 2 [Source:HGNC Symbol;Acc:HGNC:9594]	524.2	409.1	**-1.28**	0.005649
FAM104B	family with sequence similarity 104 member B [Source:HGNC Symbol;Acc:HGNC:25085]	208.7	161.8	**-1.28**	0.008507
RUSC1	RUN and SH3 domain containing 1 [Source:HGNC Symbol;Acc:HGNC:17153]	110.1	85.5	**-1.28**	0.01485
PPP1R3E	protein phosphatase 1 regulatory subunit 3E [Source:HGNC Symbol;Acc:HGNC:14943]	122.6	95.1	**-1.28**	0.01777
DNPH1	2'-deoxynucleoside 5'-phosphate N-hydrolase 1 [Source:HGNC Symbol;Acc:HGNC:21218]	98.9	77.3	**-1.28**	0.02884
COL8A1	collagen type VIII alpha 1 chain [Source:HGNC Symbol;Acc:HGNC:2215]	106.4	84.5	**-1.28**	0.04987
GAPDH	glyceraldehyde-3-phosphate dehydrogenase [Source:HGNC Symbol;Acc:HGNC:4141]	29461.7	22816.9	**-1.29**	3.95E-33
BNIP3L	BCL2 interacting protein 3 like [Source:HGNC Symbol;Acc:HGNC:1085]	6036.1	4665.6	**-1.29**	2.36E-14
SFT2D2	SFT2 domain containing 2 [Source:HGNC Symbol;Acc:HGNC:25140]	6272.8	4823.3	**-1.29**	6.82E-14
SNTB2	syntrophin beta 2 [Source:HGNC Symbol;Acc:HGNC:11169]	3980.4	3104.6	**-1.29**	8.58E-14
SLC35B1	solute carrier family 35 member B1 [Source:HGNC Symbol;Acc:HGNC:20798]	1141.3	880.5	**-1.29**	1.96E-13
NBN	nibrin [Source:HGNC Symbol;Acc:HGNC:7652]	3376.3	2621.6	**-1.29**	1.18E-12
TSEN15	tRNA splicing endonuclease subunit 15 [Source:HGNC Symbol;Acc:HGNC:16791]	1414.7	1098.4	**-1.29**	5.25E-12
HIBADH	3-hydroxyisobutyrate dehydrogenase [Source:HGNC Symbol;Acc:HGNC:4907]	722.5	560.6	**-1.29**	3.54E-11
FGF2	fibroblast growth factor 2 [Source:HGNC Symbol;Acc:HGNC:3676]	9832.2	7670.3	**-1.29**	7.20E-11
PRRX1	paired related homeobox 1 [Source:HGNC Symbol;Acc:HGNC:9142]	12216.8	9470.0	**-1.29**	1.06E-09
NR3C1	nuclear receptor subfamily 3 group C member 1 [Source:HGNC Symbol;Acc:HGNC:7978]	6709.8	5174.0	**-1.29**	2.84E-09
ARSB	arylsulfatase B [Source:HGNC Symbol;Acc:HGNC:714]	5674.2	4312.8	**-1.29**	7.00E-09
SRSF7	serine and arginine rich splicing factor 7 [Source:HGNC Symbol;Acc:HGNC:10789]	2570.7	2009.2	**-1.29**	8.69E-09
CASD1	CAS1 domain containing 1 [Source:HGNC Symbol;Acc:HGNC:16014]	1137.4	878.8	**-1.29**	1.40E-08
ANKRD36BP1	ankyrin repeat domain 36B pseudogene 1 [Source:HGNC Symbol;Acc:HGNC:28169]	750.4	579.3	**-1.29**	4.95E-08
LYRM7	LYR motif containing 7 [Source:HGNC Symbol;Acc:HGNC:28072]	1121.5	868.7	**-1.29**	4.99E-08
RWDD2B	RWD domain containing 2B [Source:HGNC Symbol;Acc:HGNC:1302]	562.1	434.2	**-1.29**	2.35E-07
PHF19	PHD finger protein 19 [Source:HGNC Symbol;Acc:HGNC:24566]	576.4	446.6	**-1.29**	4.07E-07
ATP1B1	ATPase Na+/K+ transporting subunit beta 1 [Source:HGNC Symbol;Acc:HGNC:804]	10235.3	8188.9	**-1.29**	8.09E-07
VRK1	vaccinia related kinase 1 [Source:HGNC Symbol;Acc:HGNC:12718]	609.3	470.4	**-1.29**	2.06E-06
CRY1	cryptochrome circadian regulator 1 [Source:HGNC Symbol;Acc:HGNC:2384]	850.4	654.2	**-1.29**	3.04E-06
ZCCHC14	zinc finger CCHC-type containing 14 [Source:HGNC Symbol;Acc:HGNC:24134]	923.9	720.2	**-1.29**	3.61E-06
POGLUT1	protein O-glucosyltransferase 1 [Source:HGNC Symbol;Acc:HGNC:22954]	857.6	659.4	**-1.29**	4.82E-06
TOR3A	torsin family 3 member A [Source:HGNC Symbol;Acc:HGNC:11997]	333.7	258.8	**-1.29**	1.22E-05
CLN8	CLN8, transmembrane ER and ERGIC protein [Source:HGNC Symbol;Acc:HGNC:2079]	377.2	292.4	**-1.29**	1.39E-05
GPR155	G protein-coupled receptor 155 [Source:HGNC Symbol;Acc:HGNC:22951]	312.4	240.3	**-1.29**	3.67E-05
FAM83G	family with sequence similarity 83 member G [Source:HGNC Symbol;Acc:HGNC:32554]	308.4	240.8	**-1.29**	7.03E-05
SLC47A1	solute carrier family 47 member 1 [Source:HGNC Symbol;Acc:HGNC:25588]	266.2	207.7	**-1.29**	0.0001321
XRCC1	X-ray repair cross complementing 1 [Source:HGNC Symbol;Acc:HGNC:12828]	388.9	303.1	**-1.29**	0.0001328
DZIP1L	DAZ interacting zinc finger protein 1 like [Source:HGNC Symbol;Acc:HGNC:26551]	301.5	232.4	**-1.29**	0.0001959
TMEM63B	transmembrane protein 63B [Source:HGNC Symbol;Acc:HGNC:17735]	300.0	229.7	**-1.29**	0.0002375
CDRT4	CMT1A duplicated region transcript 4 [Source:HGNC Symbol;Acc:HGNC:14383]	298.0	231.5	**-1.29**	0.0003371
ARHGEF17	Rho guanine nucleotide exchange factor 17 [Source:HGNC Symbol;Acc:HGNC:21726]	518.2	402.1	**-1.29**	0.0003936
ILVBL	ilvB acetolactate synthase like [Source:HGNC Symbol;Acc:HGNC:6041]	234.8	180.3	**-1.29**	0.0004806
TDP1	tyrosyl-DNA phosphodiesterase 1 [Source:HGNC Symbol;Acc:HGNC:18884]	199.3	153.9	**-1.29**	0.0005485
CTPS1	CTP synthase 1 [Source:HGNC Symbol;Acc:HGNC:2519]	392.3	307.5	**-1.29**	0.0005506
ZNF826P	zinc finger protein 826, pseudogene [Source:HGNC Symbol;Acc:HGNC:33875]	187.1	144.3	**-1.29**	0.0006349
DNMT3A	DNA methyltransferase 3 alpha [Source:HGNC Symbol;Acc:HGNC:2978]	235.1	183.4	**-1.29**	0.0007568
GSTZ1	glutathione S-transferase zeta 1 [Source:HGNC Symbol;Acc:HGNC:4643]	231.3	178.8	**-1.29**	0.001091
HIST2H2AB	histone cluster 2 H2A family member b [Source:HGNC Symbol;Acc:HGNC:20508]	259.6	200.9	**-1.29**	0.001362
STIL	STIL, centriolar assembly protein [Source:HGNC Symbol;Acc:HGNC:10879]	255.9	204.2	**-1.29**	0.002044
TSEN34	tRNA splicing endonuclease subunit 34 [Source:HGNC Symbol;Acc:HGNC:15506]	196.6	150.4	**-1.29**	0.002122
GATB	glutamyl-tRNA amidotransferase subunit B [Source:HGNC Symbol;Acc:HGNC:8849]	281.6	216.0	**-1.29**	0.002131

LRR1	leucine rich repeat protein 1 [Source:HGNC Symbol;Acc:HGNC:19742]	284.2	223.5	**-1.29**	0.002595
DHODH	dihydroorotate dehydrogenase (quinone) [Source:HGNC Symbol;Acc:HGNC:2867]	184.6	140.7	**-1.29**	0.004452
ADAMTS2	ADAM metallopeptidase with thrombospondin type 1 motif 2 [Source:HGNC Symbol;Acc:HGNC:218]	205.4	162.4	**-1.29**	0.005175
CNKSR2	connector enhancer of kinase suppressor of Ras 2 [Source:HGNC Symbol;Acc:HGNC:19701]	133.4	101.8	**-1.29**	0.005258
PCOLCE-AS1	PCOLCE antisense RNA 1 [Source:HGNC Symbol;Acc:HGNC:40430]	161.3	123.1	**-1.29**	0.00588
RAP2C-AS1	RAP2C antisense RNA 1 [Source:HGNC Symbol;Acc:HGNC:40957]	125.5	96.9	**-1.29**	0.006048
CERS4	ceramide synthase 4 [Source:HGNC Symbol;Acc:HGNC:23747]	115.2	87.3	**-1.29**	0.01107
FAM117B	family with sequence similarity 117 member B [Source:HGNC Symbol;Acc:HGNC:14440]	80.2	61.5	**-1.29**	0.03918
SEMA4G	semaphorin 4G [Source:HGNC Symbol;Acc:HGNC:10735]	92.6	72.3	**-1.29**	0.04185
EIF5	eukaryotic translation initiation factor 5 [Source:HGNC Symbol;Acc:HGNC:3299]	9650.3	7429.6	**-1.30**	1.14E-26
GALNT2	polypeptide N-acetylgalactosaminyltransferase 2 [Source:HGNC Symbol;Acc:HGNC:4124]	11935.9	9147.8	**-1.30**	1.14E-25
LAPTM4A	lysosomal protein transmembrane 4 alpha [Source:HGNC Symbol;Acc:HGNC:6924]	8756.9	6729.4	**-1.30**	9.21E-20
SGCB	sarcoglycan beta [Source:HGNC Symbol;Acc:HGNC:10806]	2819.2	2174.1	**-1.30**	1.96E-17
CRIM1	cysteine rich transmembrane BMP regulator 1 [Source:HGNC Symbol;Acc:HGNC:2359]	16213.4	12439.3	**-1.30**	1.48E-15
USP6NL	USP6 N-terminal like [Source:HGNC Symbol;Acc:HGNC:16858]	1694.1	1303.7	**-1.30**	3.79E-13
SLC25A5	solute carrier family 25 member 5 [Source:HGNC Symbol;Acc:HGNC:10991]	1708.5	1317.0	**-1.30**	1.23E-11
FOCAD	focadhesin [Source:HGNC Symbol;Acc:HGNC:23377]	1587.2	1216.4	**-1.30**	2.79E-11
DARS-AS1	DARS antisense RNA 1 [Source:HGNC Symbol;Acc:HGNC:40170]	522.6	402.1	**-1.30**	1.69E-09
CREB3L2	cAMP responsive element binding protein 3 like 2 [Source:HGNC Symbol;Acc:HGNC:23720]	2357.7	1809.9	**-1.30**	4.55E-09
CCDC85A	coiled-coil domain containing 85A [Source:HGNC Symbol;Acc:HGNC:29400]	1156.4	882.0	**-1.30**	6.23E-09
SLC43A3	solute carrier family 43 member 3 [Source:HGNC Symbol;Acc:HGNC:17466]	1588.8	1232.5	**-1.30**	7.04E-09
CISD2	CDGSH iron sulfur domain 2 [Source:HGNC Symbol;Acc:HGNC:24212]	614.3	472.2	**-1.30**	9.86E-09
AGPAT4	1-acylglycerol-3-phosphate O-acyltransferase 4 [Source:HGNC Symbol;Acc:HGNC:20885]	623.2	483.0	**-1.30**	1.03E-07
RNF121	ring finger protein 121 [Source:HGNC Symbol;Acc:HGNC:21070]	551.2	423.6	**-1.30**	2.15E-07
PAGR1	PAXIP1 associated glutamate rich protein 1 [Source:HGNC Symbol;Acc:HGNC:28707]	616.3	473.6	**-1.30**	3.40E-07
TRIM2	tripartite motif containing 2 [Source:HGNC Symbol;Acc:HGNC:15974]	1017.5	781.6	**-1.30**	4.76E-07
UBXN11	UBX domain protein 11 [Source:HGNC Symbol;Acc:HGNC:30600]	610.7	468.5	**-1.30**	5.33E-07
NEK11	NIMA related kinase 11 [Source:HGNC Symbol;Acc:HGNC:18593]	544.1	416.6	**-1.30**	8.96E-07
LMO7	LIM domain 7 [Source:HGNC Symbol;Acc:HGNC:6646]	3102.7	2412.4	**-1.30**	2.00E-06
NTRK2	neurotrophic receptor tyrosine kinase 2 [Source:HGNC Symbol;Acc:HGNC:8032]	587.1	446.8	**-1.30**	3.25E-06
TMEM106A	transmembrane protein 106A [Source:HGNC Symbol;Acc:HGNC:28288]	339.1	260.8	**-1.30**	4.58E-06
CFAP20	cilia and flagella associated protein 20 [Source:HGNC Symbol;Acc:HGNC:29523]	687.3	529.4	**-1.30**	5.27E-06
MLST8	MTOR associated protein, LST8 homolog [Source:HGNC Symbol;Acc:HGNC:24825]	263.7	201.3	**-1.30**	1.03E-05
PARP3	poly(ADP-ribose) polymerase family member 3 [Source:HGNC Symbol;Acc:HGNC:273]	408.8	312.9	**-1.30**	1.17E-05
EPS8L2	EPS8 like 2 [Source:HGNC Symbol;Acc:HGNC:21296]	653.9	499.6	**-1.30**	1.55E-05
TUBG1	tubulin gamma 1 [Source:HGNC Symbol;Acc:HGNC:12417]	569.3	440.5	**-1.30**	3.02E-05
BRMS1L	breast cancer metastasis-suppressor 1 like [Source:HGNC Symbol;Acc:HGNC:20512]	331.7	255.2	**-1.30**	3.20E-05
SP140L	SP140 nuclear body protein like [Source:HGNC Symbol;Acc:HGNC:25105]	262.2	201.4	**-1.30**	4.96E-05
ARMC5	armadillo repeat containing 5 [Source:HGNC Symbol;Acc:HGNC:25781]	236.5	181.4	**-1.30**	9.07E-05
EPB41L4A-AS1	EPB41L4A antisense RNA 1 [Source:HGNC Symbol;Acc:HGNC:30749]	496.4	385.5	**-1.30**	0.0001733
MCM3	minichromosome maintenance complex component 3 [Source:HGNC Symbol;Acc:HGNC:6945]	2534.3	2021.6	**-1.30**	0.0002127
NAALADL2	N-acetylated alpha-linked acidic dipeptidase like 2 [Source:HGNC Symbol;Acc:HGNC:23219]	244.5	185.8	**-1.30**	0.000243
TRDMT1	tRNA aspartic acid methyltransferase 1 [Source:HGNC Symbol;Acc:HGNC:2977]	288.1	220.7	**-1.30**	0.0002699
EML2	echinoderm microtubule associated protein like 2 [Source:HGNC Symbol;Acc:HGNC:18035]	270.6	207.9	**-1.30**	0.0002725
ZNF511	zinc finger protein 511 [Source:HGNC Symbol;Acc:HGNC:28445]	242.4	185.5	**-1.30**	0.0002807
C17orf49	chromosome 17 open reading frame 49 [Source:HGNC Symbol;Acc:HGNC:28737]	244.8	189.1	**-1.30**	0.000335
WBP1	WW domain binding protein 1 [Source:HGNC Symbol;Acc:HGNC:12737]	198.7	152.1	**-1.30**	0.000386
C3orf18	chromosome 3 open reading frame 18 [Source:HGNC Symbol;Acc:HGNC:24837]	234.4	179.5	**-1.30**	0.0005387
SPP1	secreted phosphoprotein 1 [Source:HGNC Symbol;Acc:HGNC:11255]	52263.3	37784.5	**-1.30**	0.0006229
LRRC28	leucine rich repeat containing 28 [Source:HGNC Symbol;Acc:HGNC:28355]	162.3	125.0	**-1.30**	0.0008169
ZNF300P1	zinc finger protein 300 pseudogene 1 [Source:HGNC Symbol;Acc:HGNC:27032]	187.0	145.6	**-1.30**	0.0008556
CRIP2	cysteine rich protein 2 [Source:HGNC Symbol;Acc:HGNC:2361]	422.3	318.4	**-1.30**	0.001071
RNASEH1-AS1	RNASEH1 antisense RNA 1 [Source:HGNC Symbol;Acc:HGNC:49289]	130.6	98.2	**-1.30**	0.003829
NBPF3	NBPF member 3 [Source:HGNC Symbol;Acc:HGNC:25076]	162.8	124.9	**-1.30**	0.004083
ACSM3	acyl-CoA synthetase medium chain family member 3 [Source:HGNC Symbol;Acc:HGNC:10522]	113.5	86.4	**-1.30**	0.005359
ATF3	activating transcription factor 3 [Source:HGNC Symbol;Acc:HGNC:785]	167.9	128.7	**-1.30**	0.006463
RITA1	RBPJ interacting and tubulin associated 1 [Source:HGNC Symbol;Acc:HGNC:25925]	124.4	96.0	**-1.30**	0.01658
ZNF273	zinc finger protein 273 [Source:HGNC Symbol;Acc:HGNC:13067]	118.1	89.5	**-1.30**	0.01721
TCEA1P2	transcription elongation factor A1 pseudogene 2 [Source:HGNC Symbol;Acc:HGNC:29891]	71.8	54.3	**-1.30**	0.03312
ZNF30	zinc finger protein 30 [Source:HGNC Symbol;Acc:HGNC:13090]	82.5	62.8	**-1.30**	0.03439
FAM174B	family with sequence similarity 174 member B [Source:HGNC Symbol;Acc:HGNC:34339]	85.8	65.6	**-1.30**	0.03754
SMARCA1	SWI/SNF related, matrix associated, actin dependent regulator of chromatin, subfamily a, member 1 [Sc	3897.1	2987.4	**-1.31**	8.53E-29
PTPA	protein phosphatase 2 phosphatase activator [Source:HGNC Symbol;Acc:HGNC:9308]	1039.6	793.6	**-1.31**	2.87E-14
B3GLCT	beta 3-glucosyltransferase [Source:HGNC Symbol;Acc:HGNC:20207]	1526.3	1167.7	**-1.31**	1.06E-13
SLC12A2	solute carrier family 12 member 2 [Source:HGNC Symbol;Acc:HGNC:10911]	1193.5	910.7	**-1.31**	1.36E-13
SRSF6	serine and arginine rich splicing factor 6 [Source:HGNC Symbol;Acc:HGNC:10788]	4045.9	3093.7	**-1.31**	1.36E-13
SH3BGRL3	SH3 domain binding glutamate rich protein like 3 [Source:HGNC Symbol;Acc:HGNC:15568]	1994.8	1513.3	**-1.31**	4.04E-13
SLC23A2	solute carrier family 23 member 2 [Source:HGNC Symbol;Acc:HGNC:10973]	2050.8	1567.2	**-1.31**	4.80E-13
PELI1	pellino E3 ubiquitin protein ligase 1 [Source:HGNC Symbol;Acc:HGNC:8827]	1105.6	837.9	**-1.31**	7.22E-13
LIMD1	LIM domains containing 1 [Source:HGNC Symbol;Acc:HGNC:6612]	972.6	739.7	**-1.31**	1.05E-11
GSN	gelsolin [Source:HGNC Symbol;Acc:HGNC:4620]	4377.6	3364.8	**-1.31**	1.35E-11
OXCT1	3-oxoacid CoA-transferase 1 [Source:HGNC Symbol;Acc:HGNC:8527]	2746.1	2080.6	**-1.31**	1.92E-11

ZNF608	zinc finger protein 608 [Source:HGNC Symbol;Acc:HGNC:29238]	760.1	579.4	**-1.31**	4.50E-10
ANTXR2	anthrax toxin receptor 2 [Source:HGNC Symbol;Acc:HGNC:21732]	1737.3	1308.8	**-1.31**	2.65E-09
RBBP9	RB binding protein 9, serine hydrolase [Source:HGNC Symbol;Acc:HGNC:9892]	666.6	506.0	**-1.31**	3.12E-09
P3H4	prolyl 3-hydroxylase family member 4 (non-enzymatic) [Source:HGNC Symbol;Acc:HGNC:16946]	1356.8	1036.5	**-1.31**	8.80E-09
PLCD1	phospholipase C delta 1 [Source:HGNC Symbol;Acc:HGNC:9060]	1621.5	1242.3	**-1.31**	2.53E-08
PRKCZ	protein kinase C zeta [Source:HGNC Symbol;Acc:HGNC:9412]	841.8	646.2	**-1.31**	3.10E-08
SLC1A5	solute carrier family 1 member 5 [Source:HGNC Symbol;Acc:HGNC:10943]	2706.5	2052.2	**-1.31**	3.37E-08
DCHS1	dachsous cadherin-related 1 [Source:HGNC Symbol;Acc:HGNC:13681]	1354.3	1037.0	**-1.31**	5.51E-08
NAPG	NSF attachment protein gamma [Source:HGNC Symbol;Acc:HGNC:7642]	5933.2	4485.7	**-1.31**	6.39E-08
SEMA3C	semaphorin 3C [Source:HGNC Symbol;Acc:HGNC:10725]	5192.8	3926.5	**-1.31**	3.83E-07
JUN	Jun proto-oncogene, AP-1 transcription factor subunit [Source:HGNC Symbol;Acc:HGNC:6204]	1082.0	827.2	**-1.31**	6.41E-07
A2M	alpha-2-macroglobulin [Source:HGNC Symbol;Acc:HGNC:7]	12674.7	9931.8	**-1.31**	7.33E-07
CASP8	caspase 8 [Source:HGNC Symbol;Acc:HGNC:1509]	349.8	266.2	**-1.31**	1.09E-06
CMTM3	CKLF like MARVEL transmembrane domain containing 3 [Source:HGNC Symbol;Acc:HGNC:19174]	289.1	220.1	**-1.31**	2.26E-06
CCDC58	coiled-coil domain containing 58 [Source:HGNC Symbol;Acc:HGNC:31136]	377.0	287.4	**-1.31**	1.86E-05
CRADD	CASP2 and RIPK1 domain containing adaptor with death domain [Source:HGNC Symbol;Acc:HGNC:2340	222.3	170.1	**-1.31**	4.78E-05
ZNF180	zinc finger protein 180 [Source:HGNC Symbol;Acc:HGNC:12970]	260.2	199.5	**-1.31**	8.93E-05
FANCC	Fanconi anemia complementation group C [Source:HGNC Symbol;Acc:HGNC:3584]	270.8	206.1	**-1.31**	0.000257
CKLF	chemokine like factor [Source:HGNC Symbol;Acc:HGNC:13253]	287.4	218.3	**-1.31**	0.0002903
ZC3HAV1L	zinc finger CCCH-type containing, antiviral 1 like [Source:HGNC Symbol;Acc:HGNC:22423]	203.2	155.4	**-1.31**	0.000299
UBA6-AS1	UBA6 antisense RNA 1 (head to head) [Source:HGNC Symbol;Acc:HGNC:49083]	205.1	155.1	**-1.31**	0.0005641
ID3	inhibitor of DNA binding 3, HLH protein [Source:HGNC Symbol;Acc:HGNC:5362]	544.5	425.2	**-1.31**	0.0008623
ZFP82	ZFP82 zinc finger protein [Source:HGNC Symbol;Acc:HGNC:28682]	140.5	106.8	**-1.31**	0.002441
MIS18A	MIS18 kinetochore protein A [Source:HGNC Symbol;Acc:HGNC:1286]	139.9	106.6	**-1.31**	0.002526
RALGDS	ral guanine nucleotide dissociation stimulator [Source:HGNC Symbol;Acc:HGNC:9842]	132.4	101.7	**-1.31**	0.003272
SIPA1	signal-induced proliferation-associated 1 [Source:HGNC Symbol;Acc:HGNC:10885]	131.7	100.6	**-1.31**	0.003721
GRAMD1A	GRAM domain containing 1A [Source:HGNC Symbol;Acc:HGNC:29305]	131.9	101.3	**-1.31**	0.004473
KCNK2	potassium two pore domain channel subfamily K member 2 [Source:HGNC Symbol;Acc:HGNC:6277]	94.5	70.7	**-1.31**	0.0324
BLOC1S4	biogenesis of lysosomal organelles complex 1 subunit 4 [Source:HGNC Symbol;Acc:HGNC:24206]	85.5	65.7	**-1.31**	0.04438
CEP170	centrosomal protein 170 [Source:HGNC Symbol;Acc:HGNC:28920]	1568.9	1187.8	**-1.32**	5.47E-20
SDC2	syndecan 2 [Source:HGNC Symbol;Acc:HGNC:10659]	15540.4	11636.7	**-1.32**	3.43E-15
MAGEF1	MAGE family member F1 [Source:HGNC Symbol;Acc:HGNC:29639]	1176.8	890.6	**-1.32**	3.55E-14
CACYBP	calcyclin binding protein [Source:HGNC Symbol;Acc:HGNC:30423]	3195.6	2429.3	**-1.32**	5.68E-13
LYPLAL1	lysophospholipase like 1 [Source:HGNC Symbol;Acc:HGNC:20440]	984.0	746.3	**-1.32**	7.00E-13
TTC39B	tetratricopeptide repeat domain 39B [Source:HGNC Symbol;Acc:HGNC:23704]	688.2	519.5	**-1.32**	1.13E-09
TGFB1	transforming growth factor beta 1 [Source:HGNC Symbol;Acc:HGNC:11766]	704.9	531.5	**-1.32**	1.28E-09
ATP5IF1	ATP synthase inhibitory factor subunit 1 [Source:HGNC Symbol;Acc:HGNC:871]	920.3	698.0	**-1.32**	3.76E-09
PPP1R14B	protein phosphatase 1 regulatory inhibitor subunit 14B [Source:HGNC Symbol;Acc:HGNC:9057]	1179.0	889.6	**-1.32**	5.60E-09
SLC41A1	solute carrier family 41 member 1 [Source:HGNC Symbol;Acc:HGNC:19429]	815.3	617.6	**-1.32**	6.16E-09
FRMD4B	FERM domain containing 4B [Source:HGNC Symbol;Acc:HGNC:24886]	2077.3	1592.7	**-1.32**	1.60E-08
PTPRS	protein tyrosine phosphatase, receptor type S [Source:HGNC Symbol;Acc:HGNC:9681]	948.0	721.4	**-1.32**	1.99E-08
CNTROB	centrobin, centriole duplication and spindle assembly protein [Source:HGNC Symbol;Acc:HGNC:29616]	460.2	348.5	**-1.32**	2.79E-08
RAD51D	RAD51 paralog D [Source:HGNC Symbol;Acc:HGNC:9823]	391.1	296.2	**-1.32**	3.56E-08
TCEAL2	transcription elongation factor A like 2 [Source:HGNC Symbol;Acc:HGNC:29818]	772.0	580.4	**-1.32**	5.98E-08
RGL1	ral guanine nucleotide dissociation stimulator like 1 [Source:HGNC Symbol;Acc:HGNC:30281]	349.7	263.9	**-1.32**	6.16E-07
SLC25A40	solute carrier family 25 member 40 [Source:HGNC Symbol;Acc:HGNC:29680]	342.2	260.8	**-1.32**	1.08E-06
C6orf201	chromosome 6 open reading frame 201 [Source:HGNC Symbol;Acc:HGNC:21620]	352.9	266.5	**-1.32**	7.74E-06
MRM2	mitochondrial rRNA methyltransferase 2 [Source:HGNC Symbol;Acc:HGNC:16352]	554.1	421.7	**-1.32**	1.03E-05
SLC9A3R1	SLC9A3 regulator 1 [Source:HGNC Symbol;Acc:HGNC:11075]	333.9	251.8	**-1.32**	1.68E-05
KLC4	kinesin light chain 4 [Source:HGNC Symbol;Acc:HGNC:21624]	263.4	200.4	**-1.32**	1.94E-05
B3GAT2	beta-1,3-glucuronyltransferase 2 [Source:HGNC Symbol;Acc:HGNC:922]	698.2	527.6	**-1.32**	2.15E-05
HOXA10-AS	HOXA10 antisense RNA [Source:HGNC Symbol;Acc:HGNC:40281]	308.8	234.8	**-1.32**	2.84E-05
PAQR8	progestin and adipoQ receptor family member 8 [Source:HGNC Symbol;Acc:HGNC:15708]	187.3	142.1	**-1.32**	5.53E-05
ETV6	ETS variant 6 [Source:HGNC Symbol;Acc:HGNC:3495]	272.3	204.5	**-1.32**	8.00E-05
ZBTB22	zinc finger and BTB domain containing 22 [Source:HGNC Symbol;Acc:HGNC:13085]	184.5	139.6	**-1.32**	0.0001082
SMG9	SMG9, nonsense mediated mRNA decay factor [Source:HGNC Symbol;Acc:HGNC:25763]	279.4	211.6	**-1.32**	0.0001111
MAP2K3	mitogen-activated protein kinase kinase 3 [Source:HGNC Symbol;Acc:HGNC:6843]	177.8	134.8	**-1.32**	0.0001988
SMARCAL1	SWI/SNF related, matrix associated, actin dependent regulator of chromatin, subfamily a like 1 [Source:I	264.4	199.3	**-1.32**	0.000218
GAS2L3	growth arrest specific 2 like 3 [Source:HGNC Symbol;Acc:HGNC:27475]	271.9	213.0	**-1.32**	0.0002514
PLPP1	phospholipid phosphatase 1 [Source:HGNC Symbol;Acc:HGNC:9228]	1069.9	787.5	**-1.32**	0.0004165
FAM50B	family with sequence similarity 50 member B [Source:HGNC Symbol;Acc:HGNC:18789]	189.0	143.7	**-1.32**	0.001593
ST8SIA1	ST8 alpha-N-acetyl-neuraminide alpha-2,8-sialyltransferase 1 [Source:HGNC Symbol;Acc:HGNC:10869]	180.9	140.5	**-1.32**	0.003908
AMER1	APC membrane recruitment protein 1 [Source:HGNC Symbol;Acc:HGNC:26837]	133.1	101.6	**-1.32**	0.00588
COQ8A	coenzyme Q8A [Source:HGNC Symbol;Acc:HGNC:16812]	159.2	120.2	**-1.32**	0.01029
RABGGTA	Rab geranylgeranyltransferase subunit alpha [Source:HGNC Symbol;Acc:HGNC:9795]	90.9	68.3	**-1.32**	0.02167
MUM1L1	MUM1 like 1 [Source:HGNC Symbol;Acc:HGNC:26583]	104.0	78.5	**-1.32**	0.02837
SUSD5	sushi domain containing 5 [Source:HGNC Symbol;Acc:HGNC:29061]	6932.3	5197.6	**-1.33**	7.31E-34
TMEM87B	transmembrane protein 87B [Source:HGNC Symbol;Acc:HGNC:25913]	1795.2	1349.4	**-1.33**	3.61E-18
ZDHHC20	zinc finger DHHC-type containing 20 [Source:HGNC Symbol;Acc:HGNC:20749]	3503.6	2632.7	**-1.33**	6.84E-18
PTGFRN	prostaglandin F2 receptor inhibitor [Source:HGNC Symbol;Acc:HGNC:9601]	1466.6	1101.6	**-1.33**	3.92E-17
ZEB2	zinc finger E-box binding homeobox 2 [Source:HGNC Symbol;Acc:HGNC:14881]	1040.4	775.0	**-1.33**	5.41E-15
BNIP3	BCL2 interacting protein 3 [Source:HGNC Symbol;Acc:HGNC:1084]	3854.9	2874.1	**-1.33**	3.00E-14

PDCD4	programmed cell death 4 [Source:HGNC Symbol;Acc:HGNC:8763]	5327.0	3979.9	**-1.33**	6.72E-14
PDE4D	phosphodiesterase 4D [Source:HGNC Symbol;Acc:HGNC:8783]	1507.2	1138.4	**-1.33**	1.52E-10
MAGEH1	MAGE family member H1 [Source:HGNC Symbol;Acc:HGNC:24092]	532.7	400.4	**-1.33**	5.17E-10
FAM213A	family with sequence similarity 213 member A [Source:HGNC Symbol;Acc:HGNC:28651]	460.2	344.8	**-1.33**	5.33E-10
RPAP3	RNA polymerase II associated protein 3 [Source:HGNC Symbol;Acc:HGNC:26151]	1021.8	770.1	**-1.33**	8.86E-10
DST	dystonin [Source:HGNC Symbol;Acc:HGNC:1090]	31527.7	23886.0	**-1.33**	1.32E-09
CEP78	centrosomal protein 78 [Source:HGNC Symbol;Acc:HGNC:25740]	911.6	689.2	**-1.33**	8.27E-08
PFKM	phosphofructokinase, muscle [Source:HGNC Symbol;Acc:HGNC:8877]	1075.0	811.7	**-1.33**	4.64E-07
HENMT1	HEN methyltransferase 1 [Source:HGNC Symbol;Acc:HGNC:26400]	320.3	238.7	**-1.33**	1.23E-06
HIST2H2BE	histone cluster 2 H2B family member e [Source:HGNC Symbol;Acc:HGNC:4760]	436.4	334.2	**-1.33**	3.77E-06
NME1	NME/NM23 nucleoside diphosphate kinase 1 [Source:HGNC Symbol;Acc:HGNC:7849]	1832.4	1395.1	**-1.33**	4.02E-06
NTMT1	N-terminal Xaa-Pro-Lys N-methyltransferase 1 [Source:HGNC Symbol;Acc:HGNC:23373]	315.5	236.8	**-1.33**	1.18E-05
CSK	C-terminal Src kinase [Source:HGNC Symbol;Acc:HGNC:2444]	272.5	206.3	**-1.33**	1.63E-05
PHPT1	phosphohistidine phosphatase 1 [Source:HGNC Symbol;Acc:HGNC:30033]	322.5	241.8	**-1.33**	2.33E-05
MYO19	myosin XIX [Source:HGNC Symbol;Acc:HGNC:26234]	585.6	441.3	**-1.33**	2.75E-05
PRMT7	protein arginine methyltransferase 7 [Source:HGNC Symbol;Acc:HGNC:25557]	277.5	206.5	**-1.33**	6.57E-05
METTL1	methyltransferase like 1 [Source:HGNC Symbol;Acc:HGNC:7030]	215.4	162.2	**-1.33**	0.0001426
CKLF-CMTM1	CKLF-CMTM1 readthrough [Source:HGNC Symbol;Acc:HGNC:39977]	262.0	196.9	**-1.33**	0.0003389
HS6ST1	heparan sulfate 6-O-sulfotransferase 1 [Source:HGNC Symbol;Acc:HGNC:5201]	162.6	120.9	**-1.33**	0.0003608
ATG16L2	autophagy related 16 like 2 [Source:HGNC Symbol;Acc:HGNC:25464]	249.1	187.6	**-1.33**	0.0004429
DNA2	DNA replication helicase/nuclease 2 [Source:HGNC Symbol;Acc:HGNC:2939]	237.7	180.7	**-1.33**	0.0004895
ZRANB2-AS1	ZRANB2 antisense RNA 1 [Source:HGNC Symbol;Acc:HGNC:43594]	275.3	207.6	**-1.33**	0.0008891
DTWD2	DTW domain containing 2 [Source:HGNC Symbol;Acc:HGNC:19334]	144.5	107.8	**-1.33**	0.001011
KLF3-AS1	KLF3 antisense RNA 1 [Source:HGNC Symbol;Acc:HGNC:25796]	130.4	97.2	**-1.33**	0.001117
RPP40	ribonuclease P/MRP subunit p40 [Source:HGNC Symbol;Acc:HGNC:20992]	184.8	138.6	**-1.33**	0.001152
AMN1	antagonist of mitotic exit network 1 homolog [Source:HGNC Symbol;Acc:HGNC:27281]	139.3	105.9	**-1.33**	0.002044
ZNF714	zinc finger protein 714 [Source:HGNC Symbol;Acc:HGNC:27124]	146.5	112.5	**-1.33**	0.006231
ZNF85	zinc finger protein 85 [Source:HGNC Symbol;Acc:HGNC:13160]	135.3	101.6	**-1.33**	0.008445
ANKRD23	ankyrin repeat domain 23 [Source:HGNC Symbol;Acc:HGNC:24470]	87.4	65.2	**-1.33**	0.009679
TNFRSF11B	TNF receptor superfamily member 11b [Source:HGNC Symbol;Acc:HGNC:11909]	71281.8	51916.0	**-1.33**	0.01017
CCNF	cyclin F [Source:HGNC Symbol;Acc:HGNC:1591]	105.5	80.6	**-1.33**	0.01165
TEDC1	tubulin epsilon and delta complex 1 [Source:HGNC Symbol;Acc:HGNC:20127]	57.0	42.5	**-1.33**	0.02351
GOLGA8N	golgin A8 family member N [Source:HGNC Symbol;Acc:HGNC:44405]	72.4	54.2	**-1.33**	0.03531
PMCH	pro-melanin concentrating hormone [Source:HGNC Symbol;Acc:HGNC:9109]	63.2	46.7	**-1.33**	0.03692
LINC00857	long intergenic non-protein coding RNA 857 [Source:HGNC Symbol;Acc:HGNC:45114]	53.0	39.8	**-1.33**	0.04123
MEF2A	myocyte enhancer factor 2A [Source:HGNC Symbol;Acc:HGNC:6993]	5134.4	3838.5	**-1.34**	2.66E-32
SERPINA5	serpin family A member 5 [Source:HGNC Symbol;Acc:HGNC:8723]	2904.8	2152.2	**-1.34**	3.28E-20
HACD3	3-hydroxyacyl-CoA dehydratase 3 [Source:HGNC Symbol;Acc:HGNC:24175]	3634.0	2730.6	**-1.34**	6.51E-17
GPI	glucose-6-phosphate isomerase [Source:HGNC Symbol;Acc:HGNC:4458]	2397.6	1791.3	**-1.34**	8.32E-16
ANKMY2	ankyrin repeat and MYND domain containing 2 [Source:HGNC Symbol;Acc:HGNC:25370]	728.9	543.8	**-1.34**	1.91E-13
RAB34	RAB34, member RAS oncogene family [Source:HGNC Symbol;Acc:HGNC:16519]	762.8	571.7	**-1.34**	1.34E-11
HMGCS1	3-hydroxy-3-methylglutaryl-CoA synthase 1 [Source:HGNC Symbol;Acc:HGNC:5007]	6095.4	4562.4	**-1.34**	2.15E-11
CD3EAP	CD3e molecule associated protein [Source:HGNC Symbol;Acc:HGNC:24219]	449.9	335.3	**-1.34**	1.81E-09
DGKE	diacylglycerol kinase epsilon [Source:HGNC Symbol;Acc:HGNC:2852]	409.8	306.5	**-1.34**	4.35E-09
ALG14	ALG14, UDP-N-acetylglucosaminyltransferase subunit [Source:HGNC Symbol;Acc:HGNC:28287]	377.2	281.5	**-1.34**	4.26E-08
DUSP14	dual specificity phosphatase 14 [Source:HGNC Symbol;Acc:HGNC:17007]	1014.1	755.9	**-1.34**	5.12E-08
OBSL1	obscurin like 1 [Source:HGNC Symbol;Acc:HGNC:29092]	748.2	560.5	**-1.34**	2.74E-07
UST	uronyl 2-sulfotransferase [Source:HGNC Symbol;Acc:HGNC:17223]	1086.3	807.4	**-1.34**	1.22E-06
LARP6	La ribonucleoprotein domain family member 6 [Source:HGNC Symbol;Acc:HGNC:24012]	742.6	561.4	**-1.34**	1.46E-06
PIGW	phosphatidylinositol glycan anchor biosynthesis class W [Source:HGNC Symbol;Acc:HGNC:23213]	529.5	398.5	**-1.34**	1.54E-06
PGPEP1	pyroglutamyl-peptidase I [Source:HGNC Symbol;Acc:HGNC:13568]	291.6	219.5	**-1.34**	3.15E-06
POMK	protein-O-mannose kinase [Source:HGNC Symbol;Acc:HGNC:26267]	314.9	234.6	**-1.34**	4.18E-06
PLGRKT	plasminogen receptor with a C-terminal lysine [Source:HGNC Symbol;Acc:HGNC:23633]	273.6	202.5	**-1.34**	4.31E-06
NEK6	NIMA related kinase 6 [Source:HGNC Symbol;Acc:HGNC:7749]	458.1	343.5	**-1.34**	5.41E-06
LETM2	leucine zipper and EF-hand containing transmembrane protein 2 [Source:HGNC Symbol;Acc:HGNC:1464	244.9	183.6	**-1.34**	6.94E-06
ZNF175	zinc finger protein 175 [Source:HGNC Symbol;Acc:HGNC:12964]	309.0	229.2	**-1.34**	1.89E-05
C16orf87	chromosome 16 open reading frame 87 [Source:HGNC Symbol;Acc:HGNC:33754]	310.9	232.6	**-1.34**	0.0001055
RAD51C	RAD51 paralog C [Source:HGNC Symbol;Acc:HGNC:9820]	392.7	289.6	**-1.34**	0.0002905
TMTC2	transmembrane and tetratricopeptide repeat containing 2 [Source:HGNC Symbol;Acc:HGNC:25440]	193.3	144.4	**-1.34**	0.0003572
COX19	COX19, cytochrome c oxidase assembly factor [Source:HGNC Symbol;Acc:HGNC:28074]	155.2	114.0	**-1.34**	0.00442
HIST1H4I	histone cluster 1 H4 family member i [Source:HGNC Symbol;Acc:HGNC:4793]	111.3	82.2	**-1.34**	0.004784
SLC37A4	solute carrier family 37 member 4 [Source:HGNC Symbol;Acc:HGNC:4061]	99.5	73.6	**-1.34**	0.005653
NICN1	nicolin 1 [Source:HGNC Symbol;Acc:HGNC:18317]	102.6	76.6	**-1.34**	0.005687
C19orf48	chromosome 19 open reading frame 48 [Source:HGNC Symbol;Acc:HGNC:29667]	96.5	72.5	**-1.34**	0.009822
CCDC89	coiled-coil domain containing 89 [Source:HGNC Symbol;Acc:HGNC:26762]	109.6	82.2	**-1.34**	0.01085
FYB1	FYN binding protein 1 [Source:HGNC Symbol;Acc:HGNC:4036]	147.7	107.9	**-1.34**	0.01107
DIRAS3	DIRAS family GTPase 3 [Source:HGNC Symbol;Acc:HGNC:687]	83.3	63.7	**-1.34**	0.01153
DXO	decapping exoribonuclease [Source:HGNC Symbol;Acc:HGNC:2992]	94.6	70.5	**-1.34**	0.01182
FAM111A-DT	FAM111A divergent transcript [Source:HGNC Symbol;Acc:HGNC:53752]	95.3	71.1	**-1.34**	0.01503
LINC00854	long intergenic non-protein coding RNA 854 [Source:HGNC Symbol;Acc:HGNC:43658]	65.8	48.6	**-1.34**	0.0156
EPHX1	epoxide hydrolase 1 [Source:HGNC Symbol;Acc:HGNC:3401]	79.9	59.0	**-1.34**	0.02375
ANKRD31	ankyrin repeat domain 31 [Source:HGNC Symbol;Acc:HGNC:26853]	66.2	49.3	**-1.34**	0.02714

HR	HR, lysine demethylase and nuclear receptor corepressor [Source:HGNC Symbol;Acc:HGNC:5172]	193.3	145.8	**-1.34**	0.03067
PVT1	Pvt1 oncogene (non-protein coding) [Source:HGNC Symbol;Acc:HGNC:9709]	63.0	46.2	**-1.34**	0.03384
SARS2	seryl-tRNA synthetase 2, mitochondrial [Source:HGNC Symbol;Acc:HGNC:17697]	50.2	37.0	**-1.34**	0.03832
TMEM206	transmembrane protein 206 [Source:HGNC Symbol;Acc:HGNC:25593]	99.9	72.9	**-1.34**	0.04013
TIAM2	T cell lymphoma invasion and metastasis 2 [Source:HGNC Symbol;Acc:HGNC:11806]	4120.8	3067.8	**-1.35**	6.59E-23
CDK5RAP2	CDK5 regulatory subunit associated protein 2 [Source:HGNC Symbol;Acc:HGNC:18672]	2112.6	1567.9	**-1.35**	3.38E-21
COG6	component of oligomeric golgi complex 6 [Source:HGNC Symbol;Acc:HGNC:18621]	2688.5	1986.6	**-1.35**	1.12E-19
TMEM41B	transmembrane protein 41B [Source:HGNC Symbol;Acc:HGNC:28948]	1572.6	1169.4	**-1.35**	6.86E-19
NSD2	nuclear receptor binding SET domain protein 2 [Source:HGNC Symbol;Acc:HGNC:12766]	1886.8	1402.9	**-1.35**	2.43E-18
TRAF3	TNF receptor associated factor 3 [Source:HGNC Symbol;Acc:HGNC:12033]	789.3	586.1	**-1.35**	2.82E-13
SLC2A10	solute carrier family 2 member 10 [Source:HGNC Symbol;Acc:HGNC:13444]	865.3	641.2	**-1.35**	7.54E-13
MTAP	methylthioadenosine phosphorylase [Source:HGNC Symbol;Acc:HGNC:7413]	1612.7	1187.2	**-1.35**	1.01E-11
PSMB6	proteasome subunit beta 6 [Source:HGNC Symbol;Acc:HGNC:9543]	1136.6	843.3	**-1.35**	5.12E-11
MZT1	mitotic spindle organizing protein 1 [Source:HGNC Symbol;Acc:HGNC:33830]	1686.8	1253.1	**-1.35**	2.04E-10
TMED1	transmembrane p24 trafficking protein 1 [Source:HGNC Symbol;Acc:HGNC:17291]	377.8	279.1	**-1.35**	7.68E-10
LCORL	ligand dependent nuclear receptor corepressor like [Source:HGNC Symbol;Acc:HGNC:30776]	541.4	404.6	**-1.35**	8.44E-09
RANBP1	RAN binding protein 1 [Source:HGNC Symbol;Acc:HGNC:9847]	1464.1	1091.7	**-1.35**	3.26E-08
EVC	EvC ciliary complex subunit 1 [Source:HGNC Symbol;Acc:HGNC:3497]	471.4	349.6	**-1.35**	1.01E-07
INSIG1	insulin induced gene 1 [Source:HGNC Symbol;Acc:HGNC:6083]	4108.4	3072.4	**-1.35**	2.12E-07
C11orf24	chromosome 11 open reading frame 24 [Source:HGNC Symbol;Acc:HGNC:1174]	678.2	502.7	**-1.35**	1.03E-06
OGN	osteoglycin [Source:HGNC Symbol;Acc:HGNC:8126]	6674.9	4729.2	**-1.35**	1.68E-06
ALAD	aminolevulinate dehydratase [Source:HGNC Symbol;Acc:HGNC:395]	400.9	298.7	**-1.35**	2.21E-06
TOE1	target of EGR1, exonuclease [Source:HGNC Symbol;Acc:HGNC:15954]	281.9	209.2	**-1.35**	1.51E-05
TNFAIP6	TNF alpha induced protein 6 [Source:HGNC Symbol;Acc:HGNC:11898]	1054.4	772.3	**-1.35**	3.81E-05
LIMD1-AS1	LIMD1 antisense RNA 1 [Source:HGNC Symbol;Acc:HGNC:44107]	252.2	185.6	**-1.35**	4.74E-05
TMEM170B	transmembrane protein 170B [Source:HGNC Symbol;Acc:HGNC:34244]	281.7	207.4	**-1.35**	7.15E-05
ADAMTS17	ADAM metallopeptidase with thrombospondin type 1 motif 17 [Source:HGNC Symbol;Acc:HGNC:17109]	193.4	144.1	**-1.35**	0.0002316
KRT18	keratin 18 [Source:HGNC Symbol;Acc:HGNC:6430]	303.5	228.6	**-1.35**	0.0004823
CDIP1	cell death inducing p53 target 1 [Source:HGNC Symbol;Acc:HGNC:13234]	184.7	137.2	**-1.35**	0.0006783
CBR3	carbonyl reductase 3 [Source:HGNC Symbol;Acc:HGNC:1549]	187.8	139.5	**-1.35**	0.0007651
CRMP1	collapsin response mediator protein 1 [Source:HGNC Symbol;Acc:HGNC:2365]	140.2	103.7	**-1.35**	0.0008115
XYLB	xylulokinase [Source:HGNC Symbol;Acc:HGNC:12839]	164.9	122.8	**-1.35**	0.0008786
NINL	ninein like [Source:HGNC Symbol;Acc:HGNC:29163]	113.4	83.6	**-1.35**	0.001665
HAUS8	HAUS augmin like complex subunit 8 [Source:HGNC Symbol;Acc:HGNC:30532]	148.0	110.4	**-1.35**	0.001734
SCRN2	secernin 2 [Source:HGNC Symbol;Acc:HGNC:30381]	111.5	82.0	**-1.35**	0.002386
APOO	apolipoprotein O [Source:HGNC Symbol;Acc:HGNC:28727]	172.1	128.4	**-1.35**	0.002424
SLCO3A1	solute carrier organic anion transporter family member 3A1 [Source:HGNC Symbol;Acc:HGNC:10952]	170.0	124.4	**-1.35**	0.002847
ALDH5A1	aldehyde dehydrogenase 5 family member A1 [Source:HGNC Symbol;Acc:HGNC:408]	152.6	113.8	**-1.35**	0.006185
RPL39L	ribosomal protein L39 like [Source:HGNC Symbol;Acc:HGNC:17094]	123.1	91.1	**-1.35**	0.008516
PRH1	proline rich protein HaeIII subfamily 1 [Source:HGNC Symbol;Acc:HGNC:9366]	84.5	62.0	**-1.35**	0.01396
PSTK	phosphoseryl-tRNA kinase [Source:HGNC Symbol;Acc:HGNC:28578]	69.3	51.0	**-1.35**	0.01807
HMGN5	high mobility group nucleosome binding domain 5 [Source:HGNC Symbol;Acc:HGNC:8013]	71.5	52.4	**-1.35**	0.01876
WEE2-AS1	WEE2 antisense RNA 1 [Source:HGNC Symbol;Acc:HGNC:48669]	74.6	55.3	**-1.35**	0.01952
ZRANB2-AS2	ZRANB2 antisense RNA 2 (head to head) [Source:HGNC Symbol;Acc:HGNC:43595]	60.8	45.0	**-1.35**	0.0249
BMP5	bone morphogenetic protein 5 [Source:HGNC Symbol;Acc:HGNC:1072]	99.8	72.7	**-1.35**	0.03417
FAM110B	family with sequence similarity 110 member B [Source:HGNC Symbol;Acc:HGNC:28587]	113.8	85.1	**-1.35**	0.03582
MYOC	myocilin [Source:HGNC Symbol;Acc:HGNC:7610]	79.5	63.5	**-1.35**	0.04167
GPR135	G protein-coupled receptor 135 [Source:HGNC Symbol;Acc:HGNC:19991]	43.0	31.6	**-1.35**	0.04269
CBX1	chromobox 1 [Source:HGNC Symbol;Acc:HGNC:1551]	2644.4	1961.0	**-1.36**	3.54E-17
C6orf48	chromosome 6 open reading frame 48 [Source:HGNC Symbol;Acc:HGNC:19078]	899.0	665.4	**-1.36**	1.49E-15
CPPED1	calcineurin like phosphoesterase domain containing 1 [Source:HGNC Symbol;Acc:HGNC:25632]	786.5	576.9	**-1.36**	5.55E-15
LONP1	lon peptidase 1, mitochondrial [Source:HGNC Symbol;Acc:HGNC:9479]	1142.0	842.1	**-1.36**	7.22E-15
COPG2	coatomer protein complex subunit gamma 2 [Source:HGNC Symbol;Acc:HGNC:2237]	979.9	722.8	**-1.36**	1.36E-11
TBCD	tubulin folding cofactor D [Source:HGNC Symbol;Acc:HGNC:11581]	592.3	434.9	**-1.36**	1.76E-11
MAZ	MYC associated zinc finger protein [Source:HGNC Symbol;Acc:HGNC:6914]	779.9	581.1	**-1.36**	3.49E-11
SLC9A7	solute carrier family 9 member A7 [Source:HGNC Symbol;Acc:HGNC:17123]	1084.9	808.9	**-1.36**	5.86E-10
CES2	carboxylesterase 2 [Source:HGNC Symbol;Acc:HGNC:1864]	512.9	378.9	**-1.36**	3.23E-09
OAZ2	ornithine decarboxylase antizyme 2 [Source:HGNC Symbol;Acc:HGNC:8096]	507.7	375.6	**-1.36**	7.98E-09
MASTL	microtubule associated serine/threonine kinase like [Source:HGNC Symbol;Acc:HGNC:19042]	854.7	636.3	**-1.36**	1.98E-08
C16orf70	chromosome 16 open reading frame 70 [Source:HGNC Symbol;Acc:HGNC:29564]	381.0	278.7	**-1.36**	8.82E-08
PTCD2	pentatricopeptide repeat domain 2 [Source:HGNC Symbol;Acc:HGNC:25734]	411.4	305.1	**-1.36**	9.45E-08
ARAP2	ArfGAP with RhoGAP domain, ankyrin repeat and PH domain 2 [Source:HGNC Symbol;Acc:HGNC:16924]	604.1	450.4	**-1.36**	1.90E-07
RRM1-AS1	RRM1 antisense RNA 1 [Source:HGNC Symbol;Acc:HGNC:40512]	571.8	428.7	**-1.36**	4.54E-07
KNSTRN	kinetochore localized astrin (SPAG5) binding protein [Source:HGNC Symbol;Acc:HGNC:30767]	430.2	319.3	**-1.36**	9.09E-07
TIFA	TRAF interacting protein with forkhead associated domain [Source:HGNC Symbol;Acc:HGNC:19075]	435.9	324.0	**-1.36**	1.47E-06
CENPS	centromere protein S [Source:HGNC Symbol;Acc:HGNC:23163]	324.9	238.6	**-1.36**	1.51E-06
NUAK1	NUAK family kinase 1 [Source:HGNC Symbol;Acc:HGNC:14311]	690.0	510.1	**-1.36**	1.10E-05
KIF7	kinesin family member 7 [Source:HGNC Symbol;Acc:HGNC:30497]	246.7	182.1	**-1.36**	1.47E-05
REX1BD	required for excision 1-B domain containing [Source:HGNC Symbol;Acc:HGNC:26098]	213.8	156.5	**-1.36**	1.47E-05
ST3GAL4	ST3 beta-galactoside alpha-2,3-sialyltransferase 4 [Source:HGNC Symbol;Acc:HGNC:10864]	153.7	112.2	**-1.36**	0.0001179
SMYD3	SET and MYND domain containing 3 [Source:HGNC Symbol;Acc:HGNC:15513]	196.3	143.3	**-1.36**	0.0003774
NUDT1	nudix hydrolase 1 [Source:HGNC Symbol;Acc:HGNC:8048]	159.7	118.3	**-1.36**	0.0005095

FOSL1	FOS like 1, AP-1 transcription factor subunit [Source:HGNC Symbol;Acc:HGNC:13718]	399.8	293.5	**-1.36**	0.0005895
WWOX	WW domain containing oxidoreductase [Source:HGNC Symbol;Acc:HGNC:12799]	167.5	122.5	**-1.36**	0.0006801
A2M-AS1	A2M antisense RNA 1 [Source:HGNC Symbol;Acc:HGNC:27057]	202.8	156.3	**-1.36**	0.0007511
THOC3	THO complex 3 [Source:HGNC Symbol;Acc:HGNC:19072]	165.5	122.0	**-1.36**	0.0009497
SLC22A17	solute carrier family 22 member 17 [Source:HGNC Symbol;Acc:HGNC:23095]	151.5	111.0	**-1.36**	0.001126
IRF2BP1	interferon regulatory factor 2 binding protein 1 [Source:HGNC Symbol;Acc:HGNC:21728]	140.9	103.8	**-1.36**	0.001811
TRIM16	tripartite motif containing 16 [Source:HGNC Symbol;Acc:HGNC:17241]	124.1	92.3	**-1.36**	0.002664
COL3A1	collagen type III alpha 1 chain [Source:HGNC Symbol;Acc:HGNC:2201]	12511.3	9334.8	**-1.36**	0.002724
PRKCD	protein kinase C delta [Source:HGNC Symbol;Acc:HGNC:9399]	139.7	101.4	**-1.36**	0.002957
CDK20	cyclin dependent kinase 20 [Source:HGNC Symbol;Acc:HGNC:21420]	88.9	64.7	**-1.36**	0.004958
PHF7	PHD finger protein 7 [Source:HGNC Symbol;Acc:HGNC:18458]	88.5	65.2	**-1.36**	0.006054
NAT2	N-acetyltransferase 2 [Source:HGNC Symbol;Acc:HGNC:7646]	91.2	66.2	**-1.36**	0.008237
FIGN	fidgetin, microtubule severing factor [Source:HGNC Symbol;Acc:HGNC:13285]	110.6	82.2	**-1.36**	0.009438
NECTIN1	nectin cell adhesion molecule 1 [Source:HGNC Symbol;Acc:HGNC:9706]	79.9	57.8	**-1.36**	0.01057
MN1	MN1 proto-oncogene, transcriptional regulator [Source:HGNC Symbol;Acc:HGNC:7180]	132.7	95.3	**-1.36**	0.016
EID3	EP300 interacting inhibitor of differentiation 3 [Source:HGNC Symbol;Acc:HGNC:32961]	107.0	78.4	**-1.36**	0.02161
BASP1	brain abundant membrane attached signal protein 1 [Source:HGNC Symbol;Acc:HGNC:957]	64.9	46.7	**-1.36**	0.02525
ABHD14A	abhydrolase domain containing 14A [Source:HGNC Symbol;Acc:HGNC:24538]	62.6	45.3	**-1.36**	0.03692
SNX10	sorting nexin 10 [Source:HGNC Symbol;Acc:HGNC:14974]	76.8	58.7	**-1.36**	0.03747
MAP1B	microtubule associated protein 1B [Source:HGNC Symbol;Acc:HGNC:6836]	1381.8	994.3	**-1.36**	0.04587
SLC39A8	solute carrier family 39 member 8 [Source:HGNC Symbol;Acc:HGNC:20862]	2153.4	1576.1	**-1.37**	2.05E-30
FUT11	fucosyltransferase 11 [Source:HGNC Symbol;Acc:HGNC:19233]	2104.1	1544.9	**-1.37**	9.61E-23
PFKL	phosphofructokinase, liver type [Source:HGNC Symbol;Acc:HGNC:8876]	1329.8	971.9	**-1.37**	6.57E-18
C4orf3	chromosome 4 open reading frame 3 [Source:HGNC Symbol;Acc:HGNC:19225]	3930.5	2849.5	**-1.37**	3.96E-15
TMEM18	transmembrane protein 18 [Source:HGNC Symbol;Acc:HGNC:25257]	910.9	667.8	**-1.37**	1.57E-13
EFEMP2	EGF containing fibulin extracellular matrix protein 2 [Source:HGNC Symbol;Acc:HGNC:3219]	1042.5	768.3	**-1.37**	1.03E-11
EFEMP1	EGF containing fibulin extracellular matrix protein 1 [Source:HGNC Symbol;Acc:HGNC:3218]	8871.0	6615.3	**-1.37**	5.16E-11
RRM1	ribonucleotide reductase catalytic subunit M1 [Source:HGNC Symbol;Acc:HGNC:10451]	4065.9	3027.4	**-1.37**	6.95E-11
ANKRD10	ankyrin repeat domain 10 [Source:HGNC Symbol;Acc:HGNC:20265]	1103.4	797.7	**-1.37**	7.58E-11
POLA1	DNA polymerase alpha 1, catalytic subunit [Source:HGNC Symbol;Acc:HGNC:9173]	1147.3	848.5	**-1.37**	2.54E-09
RIMKLB	ribosomal modification protein rimK like family member B [Source:HGNC Symbol;Acc:HGNC:29228]	433.1	318.2	**-1.37**	1.15E-08
CDKN2B	cyclin dependent kinase inhibitor 2B [Source:HGNC Symbol;Acc:HGNC:1788]	2719.4	1985.9	**-1.37**	8.67E-08
SUSD6	sushi domain containing 6 [Source:HGNC Symbol;Acc:HGNC:19956]	420.8	307.2	**-1.37**	1.80E-07
THNSL1	threonine synthase like 1 [Source:HGNC Symbol;Acc:HGNC:26160]	622.6	456.2	**-1.37**	1.81E-07
TREH	trehalase [Source:HGNC Symbol;Acc:HGNC:12266]	391.6	288.5	**-1.37**	2.51E-07
TPK1	thiamin pyrophosphokinase 1 [Source:HGNC Symbol;Acc:HGNC:17358]	266.9	196.7	**-1.37**	4.43E-07
CENPS-CORT	CENPS-CORT readthrough [Source:HGNC Symbol;Acc:HGNC:38843]	304.9	221.6	**-1.37**	7.97E-07
NEIL2	nei like DNA glycosylase 2 [Source:HGNC Symbol;Acc:HGNC:18956]	367.3	267.8	**-1.37**	1.70E-06
CKS2	CDC28 protein kinase regulatory subunit 2 [Source:HGNC Symbol;Acc:HGNC:2000]	427.8	319.2	**-1.37**	3.30E-06
CDC7	cell division cycle 7 [Source:HGNC Symbol;Acc:HGNC:1745]	483.7	363.5	**-1.37**	1.66E-05
SV2A	synaptic vesicle glycoprotein 2A [Source:HGNC Symbol;Acc:HGNC:20566]	184.8	133.8	**-1.37**	1.75E-05
PIK3R2	phosphoinositide-3-kinase regulatory subunit 2 [Source:HGNC Symbol;Acc:HGNC:8980]	238.0	175.2	**-1.37**	1.77E-05
CNMD	chondromodulin [Source:HGNC Symbol;Acc:HGNC:17005]	891.5	657.3	**-1.37**	2.84E-05
GEN1	GEN1, Holliday junction 5' flap endonuclease [Source:HGNC Symbol;Acc:HGNC:26881]	337.7	251.6	**-1.37**	4.89E-05
BNC2	basonuclin 2 [Source:HGNC Symbol;Acc:HGNC:30988]	427.1	309.4	**-1.37**	7.65E-05
ZNF512B	zinc finger protein 512B [Source:HGNC Symbol;Acc:HGNC:29212]	141.6	103.1	**-1.37**	0.0001676
ITPKB	inositol-trisphosphate 3-kinase B [Source:HGNC Symbol;Acc:HGNC:6179]	199.8	146.8	**-1.37**	0.0008576
APOBEC3C	apolipoprotein B mRNA editing enzyme catalytic subunit 3C [Source:HGNC Symbol;Acc:HGNC:17353]	164.0	121.1	**-1.37**	0.001343
PODNL1	podocan like 1 [Source:HGNC Symbol;Acc:HGNC:26275]	341.1	248.0	**-1.37**	0.001797
HPCAL1	hippocalcin like 1 [Source:HGNC Symbol;Acc:HGNC:5145]	171.5	125.3	**-1.37**	0.002327
CLDN23	claudin 23 [Source:HGNC Symbol;Acc:HGNC:17591]	136.0	101.1	**-1.37**	0.002683
STK32A	serine/threonine kinase 32A [Source:HGNC Symbol;Acc:HGNC:28317]	204.2	149.4	**-1.37**	0.003691
GTPBP3	GTP binding protein 3, mitochondrial [Source:HGNC Symbol;Acc:HGNC:14880]	72.5	52.5	**-1.37**	0.01032
FUOM	fucose mutarotase [Source:HGNC Symbol;Acc:HGNC:24733]	64.2	46.5	**-1.37**	0.01316
NPEPL1	aminopeptidase like 1 [Source:HGNC Symbol;Acc:HGNC:16244]	77.8	56.5	**-1.37**	0.01333
PABPC4L	poly(A) binding protein cytoplasmic 4 like [Source:HGNC Symbol;Acc:HGNC:31955]	68.4	51.1	**-1.37**	0.01857
MB21D2	Mab-21 domain containing 2 [Source:HGNC Symbol;Acc:HGNC:30438]	67.3	48.4	**-1.37**	0.02001
SPOCK3	SPARC (osteonectin), cwcv and kazal like domains proteoglycan 3 [Source:HGNC Symbol;Acc:HGNC:135(	122.1	84.6	**-1.37**	0.02298
APBA1	amyloid beta precursor protein binding family A member 1 [Source:HGNC Symbol;Acc:HGNC:578]	57.0	41.5	**-1.37**	0.04107
HTRA1	HtrA serine peptidase 1 [Source:HGNC Symbol;Acc:HGNC:9476]	11964.4	8763.4	**-1.38**	2.38E-33
RRM2B	ribonucleotide reductase regulatory TP53 inducible subunit M2B [Source:HGNC Symbol;Acc:HGNC:1729	2540.9	1846.6	**-1.38**	3.20E-23
ARMCX2	armadillo repeat containing X-linked 2 [Source:HGNC Symbol;Acc:HGNC:16869]	1619.4	1174.9	**-1.38**	3.65E-17
MAP7D3	MAP7 domain containing 3 [Source:HGNC Symbol;Acc:HGNC:25742]	6165.4	4461.8	**-1.38**	9.24E-17
COPZ2	coatomer protein complex subunit zeta 2 [Source:HGNC Symbol;Acc:HGNC:19356]	1095.5	789.9	**-1.38**	1.16E-15
HLTF	helicase like transcription factor [Source:HGNC Symbol;Acc:HGNC:11099]	3379.1	2469.1	**-1.38**	7.10E-14
MEA1	male-enhanced antigen 1 [Source:HGNC Symbol;Acc:HGNC:6986]	1326.2	967.6	**-1.38**	2.39E-13
AMPD2	adenosine monophosphate deaminase 2 [Source:HGNC Symbol;Acc:HGNC:469]	610.2	440.0	**-1.38**	4.30E-11
MPHOSPH9	M-phase phosphoprotein 9 [Source:HGNC Symbol;Acc:HGNC:7215]	990.0	725.2	**-1.38**	5.93E-11
COL9A2	collagen type IX alpha 2 chain [Source:HGNC Symbol;Acc:HGNC:2218]	1195.8	869.6	**-1.38**	3.83E-10
AXL	AXL receptor tyrosine kinase [Source:HGNC Symbol;Acc:HGNC:905]	3536.9	2588.3	**-1.38**	3.27E-08
TRIM25	tripartite motif containing 25 [Source:HGNC Symbol;Acc:HGNC:12932]	446.6	323.3	**-1.38**	9.54E-08
SLC16A3	solute carrier family 16 member 3 [Source:HGNC Symbol;Acc:HGNC:10924]	973.3	714.1	**-1.38**	1.05E-07

ZBTB2	zinc finger and BTB domain containing 2 [Source:HGNC Symbol;Acc:HGNC:20868]	349.3	254.5	**-1.38**	1.18E-07
CFI	complement factor I [Source:HGNC Symbol;Acc:HGNC:5394]	338.1	242.7	**-1.38**	1.51E-07
HYAL1	hyaluronoglucosaminidase 1 [Source:HGNC Symbol;Acc:HGNC:5320]	473.6	344.8	**-1.38**	5.65E-07
ST6GALNAC4	ST6 N-acetylgalactosaminide alpha-2,6-sialyltransferase 4 [Source:HGNC Symbol;Acc:HGNC:17846]	398.8	289.5	**-1.38**	1.65E-06
GGH	gamma-glutamyl hydrolase [Source:HGNC Symbol;Acc:HGNC:4248]	537.4	407.3	**-1.38**	1.97E-06
HEXIM1	hexamethylene bisacetamide inducible 1 [Source:HGNC Symbol;Acc:HGNC:24953]	782.0	566.8	**-1.38**	3.75E-06
SOGA1	suppressor of glucose, autophagy associated 1 [Source:HGNC Symbol;Acc:HGNC:16111]	369.4	270.5	**-1.38**	4.77E-06
SASS6	SAS-6 centriolar assembly protein [Source:HGNC Symbol;Acc:HGNC:25403]	233.1	169.0	**-1.38**	6.05E-06
PIM1	Pim-1 proto-oncogene, serine/threonine kinase [Source:HGNC Symbol;Acc:HGNC:8986]	260.3	187.4	**-1.38**	6.10E-06
DSN1	DSN1 homolog, MIS12 kinetochore complex component [Source:HGNC Symbol;Acc:HGNC:16165]	533.5	393.7	**-1.38**	4.80E-05
TRAF1	TNF receptor associated factor 1 [Source:HGNC Symbol;Acc:HGNC:12031]	196.1	143.3	**-1.38**	0.0001414
L3HYPDH	trans-L-3-hydroxyproline dehydratase [Source:HGNC Symbol;Acc:HGNC:20488]	164.8	119.9	**-1.38**	0.0001588
MYLIP	myosin regulatory light chain interacting protein [Source:HGNC Symbol;Acc:HGNC:21155]	162.3	118.9	**-1.38**	0.0004105
SNHG7	small nucleolar RNA host gene 7 [Source:HGNC Symbol;Acc:HGNC:28254]	173.3	127.2	**-1.38**	0.00106
CRYBB2P1	crystallin beta B2 pseudogene 1 [Source:HGNC Symbol;Acc:HGNC:2399]	158.7	115.0	**-1.38**	0.001692
TUBG2	tubulin gamma 2 [Source:HGNC Symbol;Acc:HGNC:12419]	87.1	63.0	**-1.38**	0.002879
CAPS2	calcyphosine 2 [Source:HGNC Symbol;Acc:HGNC:16471]	128.9	95.1	**-1.38**	0.003284
BMP6	bone morphogenetic protein 6 [Source:HGNC Symbol;Acc:HGNC:1073]	1810.2	1348.4	**-1.38**	0.003341
C3orf33	chromosome 3 open reading frame 33 [Source:HGNC Symbol;Acc:HGNC:26434]	74.8	54.2	**-1.38**	0.01264
ULBP3	UL16 binding protein 3 [Source:HGNC Symbol;Acc:HGNC:14895]	62.8	45.5	**-1.38**	0.01638
AHDC1	AT-hook DNA binding motif containing 1 [Source:HGNC Symbol;Acc:HGNC:25230]	82.1	58.3	**-1.38**	0.02587
FAM86C1	family with sequence similarity 86 member C1 [Source:HGNC Symbol;Acc:HGNC:25561]	69.6	50.4	**-1.38**	0.03482
UACA	uveal autoantigen with coiled-coil domains and ankyrin repeats [Source:HGNC Symbol;Acc:HGNC:15947	6308.9	4558.3	**-1.39**	1.22E-29
GLIPR1	GLI pathogenesis related 1 [Source:HGNC Symbol;Acc:HGNC:17001]	5224.4	3784.0	**-1.39**	4.30E-27
GLT8D2	glycosyltransferase 8 domain containing 2 [Source:HGNC Symbol;Acc:HGNC:24890]	1070.8	773.3	**-1.39**	7.25E-24
LTBP1	latent transforming growth factor beta binding protein 1 [Source:HGNC Symbol;Acc:HGNC:6714]	10069.0	7297.6	**-1.39**	3.70E-21
NEK7	NIMA related kinase 7 [Source:HGNC Symbol;Acc:HGNC:13386]	3712.3	2662.4	**-1.39**	1.31E-16
PLPP5	phospholipid phosphatase 5 [Source:HGNC Symbol;Acc:HGNC:25026]	877.3	634.1	**-1.39**	2.50E-15
JPX	JPX transcript, XIST activator (non-protein coding) [Source:HGNC Symbol;Acc:HGNC:37191]	613.2	442.6	**-1.39**	5.31E-11
NDRG3	NDRG family member 3 [Source:HGNC Symbol;Acc:HGNC:14462]	475.7	343.6	**-1.39**	7.74E-10
UNKL	unkempt family like zinc finger [Source:HGNC Symbol;Acc:HGNC:14184]	380.6	274.6	**-1.39**	2.23E-09
TMEM9	transmembrane protein 9 [Source:HGNC Symbol;Acc:HGNC:18823]	666.0	481.7	**-1.39**	4.80E-09
C1GALT1	core 1 synthase, glycoprotein-N-acetylgalactosamine 3-beta-galactosyltransferase 1 [Source:HGNC Syml	2099.8	1511.3	**-1.39**	7.31E-09
CCDC85C	coiled-coil domain containing 85C [Source:HGNC Symbol;Acc:HGNC:35459]	397.6	287.6	**-1.39**	1.16E-08
PBX2	PBX homeobox 2 [Source:HGNC Symbol;Acc:HGNC:8633]	462.8	334.5	**-1.39**	2.50E-08
MANEA	mannosidase endo-alpha [Source:HGNC Symbol;Acc:HGNC:21072]	782.6	569.3	**-1.39**	2.90E-08
PARP2	poly(ADP-ribose) polymerase 2 [Source:HGNC Symbol;Acc:HGNC:272]	373.9	271.7	**-1.39**	3.35E-08
GTPBP2	GTP binding protein 2 [Source:HGNC Symbol;Acc:HGNC:4670]	648.4	465.3	**-1.39**	6.23E-08
RFX7	regulatory factor X7 [Source:HGNC Symbol;Acc:HGNC:25777]	299.8	216.2	**-1.39**	6.85E-08
DGKD	diacylglycerol kinase delta [Source:HGNC Symbol;Acc:HGNC:2851]	292.5	210.1	**-1.39**	6.63E-07
PPARD	peroxisome proliferator activated receptor delta [Source:HGNC Symbol;Acc:HGNC:9235]	247.1	175.9	**-1.39**	2.35E-06
CHST14	carbohydrate sulfotransferase 14 [Source:HGNC Symbol;Acc:HGNC:24464]	217.1	155.6	**-1.39**	5.06E-06
PRKCH	protein kinase C eta [Source:HGNC Symbol;Acc:HGNC:9403]	191.1	136.8	**-1.39**	4.06E-05
MEGF10	multiple EGF like domains 10 [Source:HGNC Symbol;Acc:HGNC:29634]	250.9	178.0	**-1.39**	4.50E-05
VEGFB	vascular endothelial growth factor B [Source:HGNC Symbol;Acc:HGNC:12681]	236.9	172.1	**-1.39**	7.61E-05
ZMYND19	zinc finger MYND-type containing 19 [Source:HGNC Symbol;Acc:HGNC:21146]	254.6	184.3	**-1.39**	0.0001519
PRDM11	PR/SET domain 11 [Source:HGNC Symbol;Acc:HGNC:13996]	156.2	111.8	**-1.39**	0.0001871
KCNN4	potassium calcium-activated channel subfamily N member 4 [Source:HGNC Symbol;Acc:HGNC:6293]	179.6	128.0	**-1.39**	0.0002199
POLD1	DNA polymerase delta 1, catalytic subunit [Source:HGNC Symbol;Acc:HGNC:9175]	167.6	119.9	**-1.39**	0.0002201
NPM3	nucleophosmin/nucleoplasmin 3 [Source:HGNC Symbol;Acc:HGNC:7931]	220.2	159.4	**-1.39**	0.0005776
ZNF667	zinc finger protein 667 [Source:HGNC Symbol;Acc:HGNC:28854]	100.9	71.7	**-1.39**	0.002399
ATP6V1E2	ATPase H+ transporting V1 subunit E2 [Source:HGNC Symbol;Acc:HGNC:18125]	122.8	86.9	**-1.39**	0.002998
CXCL8	C-X-C motif chemokine ligand 8 [Source:HGNC Symbol;Acc:HGNC:6025]	264.9	214.2	**-1.39**	0.004737
PLPPR2	phospholipid phosphatase related 2 [Source:HGNC Symbol;Acc:HGNC:29566]	130.6	93.4	**-1.39**	0.004841
TAS2R14	taste 2 receptor member 14 [Source:HGNC Symbol;Acc:HGNC:14920]	100.8	72.2	**-1.39**	0.005048
MAP4K2	mitogen-activated protein kinase kinase kinase kinase 2 [Source:HGNC Symbol;Acc:HGNC:6864]	72.3	52.0	**-1.39**	0.005515
PLK1	polo like kinase 1 [Source:HGNC Symbol;Acc:HGNC:9077]	90.9	66.6	**-1.39**	0.005858
BOLA2-SMG1P6	BOLA2-SMG1P6 readthrough [Source:HGNC Symbol;Acc:HGNC:53563]	62.6	44.6	**-1.39**	0.01902
IQCC	IQ motif containing C [Source:HGNC Symbol;Acc:HGNC:25545]	75.9	53.7	**-1.39**	0.03316
PRELID3A	PRELI domain containing 3A [Source:HGNC Symbol;Acc:HGNC:24639]	58.1	41.6	**-1.39**	0.03842
ITGB7	integrin subunit beta 7 [Source:HGNC Symbol;Acc:HGNC:6162]	41.1	29.4	**-1.39**	0.04868
ALDOA	aldolase, fructose-bisphosphate A [Source:HGNC Symbol;Acc:HGNC:414]	4484.0	3231.3	**-1.39**	4.92E-32
CDK2AP1	cyclin dependent kinase 2 associated protein 1 [Source:HGNC Symbol;Acc:HGNC:14002]	5673.9	4070.6	**-1.39**	1.40E-29
BEX4	brain expressed X-linked 4 [Source:HGNC Symbol;Acc:HGNC:25475]	931.9	670.6	**-1.39**	2.02E-17
DNMT1	DNA methyltransferase 1 [Source:HGNC Symbol;Acc:HGNC:2976]	1888.1	1364.1	**-1.39**	6.60E-17
SCD	stearoyl-CoA desaturase [Source:HGNC Symbol;Acc:HGNC:10571]	7335.4	5307.0	**-1.39**	5.50E-13
ANO6	anoctamin 6 [Source:HGNC Symbol;Acc:HGNC:25240]	1800.0	1297.0	**-1.39**	1.32E-12
MEG8	maternally expressed 8, small nucleolar RNA host gene [Source:HGNC Symbol;Acc:HGNC:14574]	693.7	508.2	**-1.39**	6.09E-12
ALKBH8	alkB homolog 8, tRNA methyltransferase [Source:HGNC Symbol;Acc:HGNC:25189]	543.8	387.9	**-1.39**	2.11E-11
OLFM1	olfactomedin 1 [Source:HGNC Symbol;Acc:HGNC:17187]	749.4	538.1	**-1.39**	3.96E-11
CARNMT1	carnosine N-methyltransferase 1 [Source:HGNC Symbol;Acc:HGNC:23435]	974.6	702.2	**-1.39**	1.39E-10
HIST1H2BD	histone cluster 1 H2B family member d [Source:HGNC Symbol;Acc:HGNC:4747]	1029.5	739.8	**-1.39**	1.62E-10

APEX1	apurinic/apyrimidinic endodeoxyribonuclease 1 [Source:HGNC Symbol;Acc:HGNC:587]	1831.5	1324.9	**-1.39**	1.96E-10
NASP	nuclear autoantigenic sperm protein [Source:HGNC Symbol;Acc:HGNC:7644]	1603.2	1165.4	**-1.39**	6.42E-09
PRDX2	peroxiredoxin 2 [Source:HGNC Symbol;Acc:HGNC:9353]	746.6	540.7	**-1.39**	6.44E-09
F8	coagulation factor VIII [Source:HGNC Symbol;Acc:HGNC:3546]	494.1	354.2	**-1.39**	8.51E-09
CAPS	calcyphosine [Source:HGNC Symbol;Acc:HGNC:1487]	462.4	333.0	**-1.39**	1.18E-08
PLAUR	plasminogen activator, urokinase receptor [Source:HGNC Symbol;Acc:HGNC:9053]	1489.5	1022.4	**-1.39**	1.86E-08
CCNB1	cyclin B1 [Source:HGNC Symbol;Acc:HGNC:1579]	1350.9	1041.6	**-1.39**	5.71E-08
TREX2	three prime repair exonuclease 2 [Source:HGNC Symbol;Acc:HGNC:12270]	259.2	185.9	**-1.39**	2.92E-06
PLD1	phospholipase D1 [Source:HGNC Symbol;Acc:HGNC:9067]	529.7	379.9	**-1.39**	4.84E-06
PTPRF	protein tyrosine phosphatase, receptor type F [Source:HGNC Symbol;Acc:HGNC:9670]	533.9	379.7	**-1.39**	5.74E-06
PRPF40B	pre-mRNA processing factor 40 homolog B [Source:HGNC Symbol;Acc:HGNC:25031]	227.9	162.0	**-1.39**	5.82E-06
SH3D21	SH3 domain containing 21 [Source:HGNC Symbol;Acc:HGNC:26236]	310.6	222.8	**-1.39**	1.91E-05
WDHD1	WD repeat and HMG-box DNA binding protein 1 [Source:HGNC Symbol;Acc:HGNC:23170]	847.7	631.4	**-1.39**	5.62E-05
CAMKK1	calcium/calmodulin dependent protein kinase kinase 1 [Source:HGNC Symbol;Acc:HGNC:1469]	327.6	230.7	**-1.39**	8.58E-05
FRK	fyn related Src family tyrosine kinase [Source:HGNC Symbol;Acc:HGNC:3955]	257.3	185.2	**-1.39**	0.0004565
FLG-AS1	FLG antisense RNA 1 [Source:HGNC Symbol;Acc:HGNC:27913]	142.3	104.1	**-1.39**	0.0005866
ACACB	acetyl-CoA carboxylase beta [Source:HGNC Symbol;Acc:HGNC:85]	192.8	139.3	**-1.39**	0.0006438
PCDHB7	protocadherin beta 7 [Source:HGNC Symbol;Acc:HGNC:8692]	120.0	82.4	**-1.39**	0.002985
NFKBIE	NFKB inhibitor epsilon [Source:HGNC Symbol;Acc:HGNC:7799]	85.9	61.3	**-1.39**	0.004883
FAM83D	family with sequence similarity 83 member D [Source:HGNC Symbol;Acc:HGNC:16122]	119.1	86.0	**-1.39**	0.005623
TBKBP1	TBK1 binding protein 1 [Source:HGNC Symbol;Acc:HGNC:30140]	74.9	53.2	**-1.39**	0.006096
CENPA	centromere protein A [Source:HGNC Symbol;Acc:HGNC:1851]	91.2	66.5	**-1.39**	0.01303
C5orf30	chromosome 5 open reading frame 30 [Source:HGNC Symbol;Acc:HGNC:25052]	64.9	46.4	**-1.39**	0.0273
LINC00624	long intergenic non-protein coding RNA 624 [Source:HGNC Symbol;Acc:HGNC:44254]	48.4	34.4	**-1.39**	0.02865
CMIP	c-Maf inducing protein [Source:HGNC Symbol;Acc:HGNC:24319]	65.9	47.6	**-1.39**	0.03386
RFX2	regulatory factor X2 [Source:HGNC Symbol;Acc:HGNC:9983]	51.0	35.9	**-1.39**	0.0344
RNF212	ring finger protein 212 [Source:HGNC Symbol;Acc:HGNC:27729]	64.3	44.4	**-1.39**	0.04022
ZNHIT6	zinc finger HIT-type containing 6 [Source:HGNC Symbol;Acc:HGNC:26089]	2465.6	1762.1	**-1.40**	6.76E-25
P3H2	prolyl 3-hydroxylase 2 [Source:HGNC Symbol;Acc:HGNC:19317]	23211.8	16599.2	**-1.40**	1.03E-23
QSOX2	quiescin sulfhydryl oxidase 2 [Source:HGNC Symbol;Acc:HGNC:30249]	747.1	531.3	**-1.40**	1.20E-16
CENPO	centromere protein O [Source:HGNC Symbol;Acc:HGNC:28152]	652.2	464.9	**-1.40**	4.18E-16
KIFC3	kinesin family member C3 [Source:HGNC Symbol;Acc:HGNC:6326]	696.2	494.7	**-1.40**	9.24E-16
GORAB	golgin, RAB6 interacting [Source:HGNC Symbol;Acc:HGNC:25676]	833.9	592.4	**-1.40**	1.30E-15
ITM2A	integral membrane protein 2A [Source:HGNC Symbol;Acc:HGNC:6173]	1431.6	1028.5	**-1.40**	9.94E-14
EIF4EBP1	eukaryotic translation initiation factor 4E binding protein 1 [Source:HGNC Symbol;Acc:HGNC:3288]	516.2	367.9	**-1.40**	8.18E-13
SDC3	syndecan 3 [Source:HGNC Symbol;Acc:HGNC:10660]	753.0	532.3	**-1.40**	3.31E-12
NCAPD2	non-SMC condensin I complex subunit D2 [Source:HGNC Symbol;Acc:HGNC:24305]	1090.8	792.7	**-1.40**	2.84E-11
ZWILCH	zwilch kinetochore protein [Source:HGNC Symbol;Acc:HGNC:25468]	1133.0	814.7	**-1.40**	1.45E-10
DENND1B	DENN domain containing 1B [Source:HGNC Symbol;Acc:HGNC:28404]	759.1	537.4	**-1.40**	3.54E-10
NT5DC1	5'-nucleotidase domain containing 1 [Source:HGNC Symbol;Acc:HGNC:21556]	681.5	488.0	**-1.40**	5.65E-10
RTEL1-TNFRSF6B	RTEL1-TNFRSF6B readthrough (NMD candidate) [Source:HGNC Symbol;Acc:HGNC:44095]	307.6	217.7	**-1.40**	6.39E-09
GAREM1	GRB2 associated regulator of MAPK1 subtype 1 [Source:HGNC Symbol;Acc:HGNC:26136]	820.5	577.8	**-1.40**	5.92E-08
CMC2	C-X9-C motif containing 2 [Source:HGNC Symbol;Acc:HGNC:24447]	342.6	243.4	**-1.40**	7.22E-08
C18orf54	chromosome 18 open reading frame 54 [Source:HGNC Symbol;Acc:HGNC:13796]	247.8	174.6	**-1.40**	8.35E-08
TBC1D2	TBC1 domain family member 2 [Source:HGNC Symbol;Acc:HGNC:18026]	228.8	161.2	**-1.40**	5.63E-07
C1orf122	chromosome 1 open reading frame 122 [Source:HGNC Symbol;Acc:HGNC:24789]	182.2	128.6	**-1.40**	1.00E-06
GPR161	G protein-coupled receptor 161 [Source:HGNC Symbol;Acc:HGNC:23694]	252.4	180.7	**-1.40**	1.47E-06
HAUS7	HAUS augmin like complex subunit 7 [Source:HGNC Symbol;Acc:HGNC:32979]	264.2	188.3	**-1.40**	1.67E-06
IFT27	intraflagellar transport 27 [Source:HGNC Symbol;Acc:HGNC:18626]	185.6	131.0	**-1.40**	2.51E-06
TARBP1	TAR (HIV-1) RNA binding protein 1 [Source:HGNC Symbol;Acc:HGNC:11568]	340.9	243.2	**-1.40**	5.84E-06
TMEM25	transmembrane protein 25 [Source:HGNC Symbol;Acc:HGNC:25890]	280.5	199.7	**-1.40**	9.93E-06
EPHB4	EPH receptor B4 [Source:HGNC Symbol;Acc:HGNC:3395]	283.1	204.3	**-1.40**	6.43E-05
MREG	melanoregulin [Source:HGNC Symbol;Acc:HGNC:25478]	223.3	162.2	**-1.40**	0.0001702
KCNJ15	potassium voltage-gated channel subfamily J member 15 [Source:HGNC Symbol;Acc:HGNC:6261]	135.4	95.2	**-1.40**	0.0002819
LIMK1	LIM domain kinase 1 [Source:HGNC Symbol;Acc:HGNC:6613]	132.7	93.4	**-1.40**	0.0006385
ZNF239	zinc finger protein 239 [Source:HGNC Symbol;Acc:HGNC:13031]	129.4	92.7	**-1.40**	0.001709
MEOX2	mesenchyme homeobox 2 [Source:HGNC Symbol;Acc:HGNC:7014]	209.3	153.6	**-1.40**	0.001924
ATF5	activating transcription factor 5 [Source:HGNC Symbol;Acc:HGNC:790]	137.0	94.9	**-1.40**	0.002955
NRM	nurim [Source:HGNC Symbol;Acc:HGNC:8003]	94.8	67.3	**-1.40**	0.004473
ADAM21	ADAM metallopeptidase domain 21 [Source:HGNC Symbol;Acc:HGNC:200]	52.4	37.1	**-1.40**	0.02294
PRX	periaxin [Source:HGNC Symbol;Acc:HGNC:13797]	52.3	36.6	**-1.40**	0.02482
ZNF792	zinc finger protein 792 [Source:HGNC Symbol;Acc:HGNC:24751]	54.0	38.5	**-1.40**	0.0289
CHCHD10	coiled-coil-helix-coiled-coil-helix domain containing 10 [Source:HGNC Symbol;Acc:HGNC:15559]	54.3	38.0	**-1.40**	0.02902
ZBTB20-AS1	ZBTB20 antisense RNA 1 [Source:HGNC Symbol;Acc:HGNC:40640]	44.0	31.2	**-1.40**	0.03261
GPD2	glycerol-3-phosphate dehydrogenase 2 [Source:HGNC Symbol;Acc:HGNC:4456]	5020.9	3521.5	**-1.41**	4.30E-22
LTBP2	latent transforming growth factor beta binding protein 2 [Source:HGNC Symbol;Acc:HGNC:6715]	9228.6	6644.6	**-1.41**	1.96E-17
MRC2	mannose receptor C type 2 [Source:HGNC Symbol;Acc:HGNC:16875]	3333.9	2404.3	**-1.41**	1.49E-16
PCDHGB4	protocadherin gamma subfamily B, 4 [Source:HGNC Symbol;Acc:HGNC:8711]	1877.2	1324.6	**-1.41**	9.27E-12
ADPRHL2	ADP-ribosylhydrolase like 2 [Source:HGNC Symbol;Acc:HGNC:21304]	455.4	323.6	**-1.41**	2.01E-11
PCDHGB6	protocadherin gamma subfamily B, 6 [Source:HGNC Symbol;Acc:HGNC:8713]	1647.7	1157.8	**-1.41**	5.57E-11
PPP1R14C	protein phosphatase 1 regulatory inhibitor subunit 14C [Source:HGNC Symbol;Acc:HGNC:14952]	871.8	623.1	**-1.41**	1.25E-10
CENPL	centromere protein L [Source:HGNC Symbol;Acc:HGNC:17879]	359.5	255.7	**-1.41**	2.13E-09

RPL23AP82	ribosomal protein L23a pseudogene 82 [Source:HGNC Symbol;Acc:HGNC:33730]	382.3	271.2	**-1.41**	7.04E-09
CMSS1	cms1 ribosomal small subunit homolog (yeast) [Source:HGNC Symbol;Acc:HGNC:28666]	710.8	505.3	**-1.41**	9.06E-09
DNAJC9	DnaJ heat shock protein family (Hsp40) member C9 [Source:HGNC Symbol;Acc:HGNC:19123]	1480.8	1067.7	**-1.41**	1.11E-08
EFS	embryonal Fyn-associated substrate [Source:HGNC Symbol;Acc:HGNC:16898]	265.7	188.8	**-1.41**	3.28E-08
MRPL48	mitochondrial ribosomal protein L48 [Source:HGNC Symbol;Acc:HGNC:16653]	361.9	258.1	**-1.41**	6.50E-08
PIGU	phosphatidylinositol glycan anchor biosynthesis class U [Source:HGNC Symbol;Acc:HGNC:15791]	270.6	191.8	**-1.41**	1.07E-07
OSBPL3	oxysterol binding protein like 3 [Source:HGNC Symbol;Acc:HGNC:16370]	392.2	283.3	**-1.41**	2.26E-07
SLC36A1	solute carrier family 36 member 1 [Source:HGNC Symbol;Acc:HGNC:18761]	313.1	221.5	**-1.41**	3.43E-07
PCYOX1L	prenylcysteine oxidase 1 like [Source:HGNC Symbol;Acc:HGNC:28477]	172.9	122.4	**-1.41**	4.07E-06
TLE4	transducin like enhancer of split 4 [Source:HGNC Symbol;Acc:HGNC:11840]	188.0	132.2	**-1.41**	1.36E-05
PIR	pirin [Source:HGNC Symbol;Acc:HGNC:30048]	237.9	171.4	**-1.41**	5.85E-05
CHRNE	cholinergic receptor nicotinic epsilon subunit [Source:HGNC Symbol;Acc:HGNC:1966]	206.0	143.5	**-1.41**	0.0001033
OGFOD2	2-oxoglutarate and iron dependent oxygenase domain containing 2 [Source:HGNC Symbol;Acc:HGNC:2!	111.6	78.1	**-1.41**	0.0001284
ZMYM3	zinc finger MYM-type containing 3 [Source:HGNC Symbol;Acc:HGNC:13054]	166.6	118.4	**-1.41**	0.0001702
TRIM65	tripartite motif containing 65 [Source:HGNC Symbol;Acc:HGNC:27316]	159.6	113.1	**-1.41**	0.0004081
SIM2	SIM bHLH transcription factor 2 [Source:HGNC Symbol;Acc:HGNC:10883]	127.3	90.3	**-1.41**	0.0004212
TRIM16L	tripartite motif containing 16 like [Source:HGNC Symbol;Acc:HGNC:32670]	147.6	105.1	**-1.41**	0.0005693
ANKDD1A	ankyrin repeat and death domain containing 1A [Source:HGNC Symbol;Acc:HGNC:28002]	100.5	70.5	**-1.41**	0.0007396
BTN3A1	butyrophilin subfamily 3 member A1 [Source:HGNC Symbol;Acc:HGNC:1138]	119.9	82.8	**-1.41**	0.001446
SLC14A1	solute carrier family 14 member 1 (Kidd blood group) [Source:HGNC Symbol;Acc:HGNC:10918]	216.5	150.2	**-1.41**	0.002948
ADAM22	ADAM metallopeptidase domain 22 [Source:HGNC Symbol;Acc:HGNC:201]	109.5	77.2	**-1.41**	0.003589
TMEM39B	transmembrane protein 39B [Source:HGNC Symbol;Acc:HGNC:25510]	92.9	65.0	**-1.41**	0.004108
TMEM91	transmembrane protein 91 [Source:HGNC Symbol;Acc:HGNC:32393]	69.8	48.4	**-1.41**	0.005886
HRCT1	histidine rich carboxyl terminus 1 [Source:HGNC Symbol;Acc:HGNC:33872]	140.3	98.8	**-1.41**	0.006555
HMBS	hydroxymethylbilane synthase [Source:HGNC Symbol;Acc:HGNC:4982]	83.8	58.4	**-1.41**	0.009065
KIAA1024	KIAA1024 [Source:HGNC Symbol;Acc:HGNC:29172]	56.0	39.4	**-1.41**	0.01358
ZNF165	zinc finger protein 165 [Source:HGNC Symbol;Acc:HGNC:12953]	42.4	29.2	**-1.41**	0.02029
BEND3	BEN domain containing 3 [Source:HGNC Symbol;Acc:HGNC:23040]	81.0	57.4	**-1.41**	0.02222
C1QTNF7	C1q and TNF related 7 [Source:HGNC Symbol;Acc:HGNC:14342]	56.7	40.3	**-1.41**	0.03884
MDFIC	MyoD family inhibitor domain containing [Source:HGNC Symbol;Acc:HGNC:28870]	6217.4	4364.9	**-1.42**	1.21E-35
GLS	glutaminase [Source:HGNC Symbol;Acc:HGNC:4331]	10983.1	7737.8	**-1.42**	1.51E-31
CHD1L	chromodomain helicase DNA binding protein 1 like [Source:HGNC Symbol;Acc:HGNC:1916]	1924.4	1348.2	**-1.42**	4.03E-30
IARS	isoleucyl-tRNA synthetase [Source:HGNC Symbol;Acc:HGNC:5330]	8945.9	6301.3	**-1.42**	4.67E-27
DKK3	dickkopf WNT signaling pathway inhibitor 3 [Source:HGNC Symbol;Acc:HGNC:2893]	1823.0	1278.2	**-1.42**	2.31E-25
CEP97	centrosomal protein 97 [Source:HGNC Symbol;Acc:HGNC:26244]	1263.5	891.6	**-1.42**	3.27E-21
TFRC	transferrin receptor [Source:HGNC Symbol;Acc:HGNC:11763]	16460.2	11583.5	**-1.42**	2.42E-20
HIST1H2AC	histone cluster 1 H2A family member c [Source:HGNC Symbol;Acc:HGNC:4733]	1084.6	770.5	**-1.42**	4.93E-20
EHBP1L1	EH domain binding protein 1 like 1 [Source:HGNC Symbol;Acc:HGNC:30682]	3012.7	2134.1	**-1.42**	8.42E-20
PTMA	prothymosin alpha [Source:HGNC Symbol;Acc:HGNC:9623]	21149.6	14991.7	**-1.42**	1.62E-17
GPRASP1	G protein-coupled receptor associated sorting protein 1 [Source:HGNC Symbol;Acc:HGNC:24834]	917.8	647.0	**-1.42**	1.33E-16
SMC4	structural maintenance of chromosomes 4 [Source:HGNC Symbol;Acc:HGNC:14013]	3440.2	2464.1	**-1.42**	6.42E-14
STMP1	short transmembrane mitochondrial protein 1 [Source:HGNC Symbol;Acc:HGNC:41909]	1051.0	744.0	**-1.42**	4.51E-13
S100A10	S100 calcium binding protein A10 [Source:HGNC Symbol;Acc:HGNC:10487]	3199.8	2234.2	**-1.42**	1.19E-12
PCDHGA8	protocadherin gamma subfamily A, 8 [Source:HGNC Symbol;Acc:HGNC:8706]	1665.6	1166.2	**-1.42**	5.95E-12
RPA3	replication protein A3 [Source:HGNC Symbol;Acc:HGNC:10291]	880.8	618.3	**-1.42**	7.73E-12
PLEKHA4	pleckstrin homology domain containing A4 [Source:HGNC Symbol;Acc:HGNC:14339]	595.9	417.5	**-1.42**	2.79E-11
SDHA	succinate dehydrogenase complex flavoprotein subunit A [Source:HGNC Symbol;Acc:HGNC:10680]	3179.9	2181.8	**-1.42**	2.91E-11
TSPAN13	tetraspanin 13 [Source:HGNC Symbol;Acc:HGNC:21643]	1137.2	794.0	**-1.42**	4.95E-11
ENOSF1	enolase superfamily member 1 [Source:HGNC Symbol;Acc:HGNC:30365]	865.6	609.7	**-1.42**	8.25E-11
PTPRK	protein tyrosine phosphatase, receptor type K [Source:HGNC Symbol;Acc:HGNC:9674]	651.8	452.3	**-1.42**	1.01E-10
AKR1B1	aldo-keto reductase family 1 member B [Source:HGNC Symbol;Acc:HGNC:381]	509.7	358.6	**-1.42**	1.39E-09
HK2	hexokinase 2 [Source:HGNC Symbol;Acc:HGNC:4923]	1150.5	812.5	**-1.42**	2.84E-08
RAP2B	RAP2B, member of RAS oncogene family [Source:HGNC Symbol;Acc:HGNC:9862]	475.7	331.6	**-1.42**	7.23E-08
CIC	capicua transcriptional repressor [Source:HGNC Symbol;Acc:HGNC:14214]	398.8	277.8	**-1.42**	1.49E-06
ASPM	abnormal spindle microtubule assembly [Source:HGNC Symbol;Acc:HGNC:19048]	1274.4	988.7	**-1.42**	6.60E-06
ARL4A	ADP ribosylation factor like GTPase 4A [Source:HGNC Symbol;Acc:HGNC:695]	187.5	130.7	**-1.42**	1.36E-05
IL17RD	interleukin 17 receptor D [Source:HGNC Symbol;Acc:HGNC:17616]	109.2	75.9	**-1.42**	6.72E-05
CEACAM19	carcinoembryonic antigen related cell adhesion molecule 19 [Source:HGNC Symbol;Acc:HGNC:31951]	168.2	118.0	**-1.42**	7.03E-05
CACNA1D	calcium voltage-gated channel subunit alpha1 D [Source:HGNC Symbol;Acc:HGNC:1391]	74.3	51.1	**-1.42**	0.00111
GSTM4	glutathione S-transferase mu 4 [Source:HGNC Symbol;Acc:HGNC:4636]	97.9	68.5	**-1.42**	0.001665
RNF207	ring finger protein 207 [Source:HGNC Symbol;Acc:HGNC:32947]	85.0	58.9	**-1.42**	0.002718
DHRS1	dehydrogenase/reductase 1 [Source:HGNC Symbol;Acc:HGNC:16445]	75.0	52.2	**-1.42**	0.00482
LAGE3	L antigen family member 3 [Source:HGNC Symbol;Acc:HGNC:26058]	83.5	58.4	**-1.42**	0.005002
B3GNTL1	UDP-GlcNAc:betaGal beta-1,3-N-acetylglucosaminyltransferase like 1 [Source:HGNC Symbol;Acc:HGNC::	80.9	55.7	**-1.42**	0.01301
CHRNA5	cholinergic receptor nicotinic alpha 5 subunit [Source:HGNC Symbol;Acc:HGNC:1959]	63.1	45.4	**-1.42**	0.02037
EXOSC5	exosome component 5 [Source:HGNC Symbol;Acc:HGNC:24662]	55.9	38.7	**-1.42**	0.02367
FAM66C	family with sequence similarity 66 member C [Source:HGNC Symbol;Acc:HGNC:21644]	54.6	37.2	**-1.42**	0.02743
PTMAP2	prothymosin alpha pseudogene 2 [Source:HGNC Symbol;Acc:HGNC:9625]	49.2	34.0	**-1.42**	0.02812
INPP5F	inositol polyphosphate-5-phosphatase F [Source:HGNC Symbol;Acc:HGNC:17054]	1807.9	1258.9	**-1.43**	8.58E-32
RAP2C	RAP2C, member of RAS oncogene family [Source:HGNC Symbol;Acc:HGNC:21165]	1431.2	996.8	**-1.43**	4.99E-31
UBB	ubiquitin B [Source:HGNC Symbol;Acc:HGNC:12463]	8672.8	6057.7	**-1.43**	8.30E-30
ZNF521	zinc finger protein 521 [Source:HGNC Symbol;Acc:HGNC:24605]	1177.0	820.7	**-1.43**	1.33E-24

METRNL	meteorin like, glial cell differentiation regulator [Source:HGNC Symbol;Acc:HGNC:27584]	1445.4	1005.5	**-1.43**	1.75E-23
NQO2	N-ribosyldihydronicotinamide:quinone reductase 2 [Source:HGNC Symbol;Acc:HGNC:7856]	1933.5	1344.8	**-1.43**	4.33E-20
LIMA1	LIM domain and actin binding 1 [Source:HGNC Symbol;Acc:HGNC:24636]	4556.8	3239.2	**-1.43**	1.18E-17
P3H3	prolyl 3-hydroxylase 3 [Source:HGNC Symbol;Acc:HGNC:19318]	766.1	532.1	**-1.43**	2.93E-16
PHLDB1	pleckstrin homology like domain family B member 1 [Source:HGNC Symbol;Acc:HGNC:23697]	2493.5	1756.7	**-1.43**	2.74E-15
CLIP2	CAP-Gly domain containing linker protein 2 [Source:HGNC Symbol;Acc:HGNC:2586]	833.2	582.6	**-1.43**	4.00E-15
TMSB4X	thymosin beta 4 X-linked [Source:HGNC Symbol;Acc:HGNC:11881]	4269.5	2988.5	**-1.43**	4.48E-15
TMEM65	transmembrane protein 65 [Source:HGNC Symbol;Acc:HGNC:25203]	725.2	505.0	**-1.43**	7.01E-14
MLF2	myeloid leukemia factor 2 [Source:HGNC Symbol;Acc:HGNC:7126]	580.2	403.3	**-1.43**	1.24E-13
ME1	malic enzyme 1 [Source:HGNC Symbol;Acc:HGNC:6983]	736.4	510.2	**-1.43**	1.80E-11
CUTA	cutA divalent cation tolerance homolog [Source:HGNC Symbol;Acc:HGNC:21101]	346.3	242.4	**-1.43**	7.65E-11
TRIM59	tripartite motif containing 59 [Source:HGNC Symbol;Acc:HGNC:30834]	956.4	685.2	**-1.43**	1.08E-10
PCDHGB5	protocadherin gamma subfamily B, 5 [Source:HGNC Symbol;Acc:HGNC:8712]	1646.0	1142.6	**-1.43**	1.41E-10
XYLT2	xylosyltransferase 2 [Source:HGNC Symbol;Acc:HGNC:15517]	350.9	245.1	**-1.43**	4.58E-10
SPC24	SPC24, NDC80 kinetochore complex component [Source:HGNC Symbol;Acc:HGNC:26913]	438.7	310.6	**-1.43**	1.55E-09
NPR2	natriuretic peptide receptor 2 [Source:HGNC Symbol;Acc:HGNC:7944]	815.8	556.5	**-1.43**	6.47E-09
DAAM1	dishevelled associated activator of morphogenesis 1 [Source:HGNC Symbol;Acc:HGNC:18142]	462.8	324.2	**-1.43**	6.87E-09
TIPIN	TIMELESS interacting protein [Source:HGNC Symbol;Acc:HGNC:30750]	589.1	414.1	**-1.43**	7.56E-07
RFC4	replication factor C subunit 4 [Source:HGNC Symbol;Acc:HGNC:9972]	605.1	429.5	**-1.43**	1.70E-06
FANCA	Fanconi anemia complementation group A [Source:HGNC Symbol;Acc:HGNC:3582]	226.1	159.5	**-1.43**	2.36E-05
ISPD	isoprenoid synthase domain containing [Source:HGNC Symbol;Acc:HGNC:37276]	123.9	86.1	**-1.43**	2.82E-05
PAQR7	progestin and adipoQ receptor family member 7 [Source:HGNC Symbol;Acc:HGNC:23146]	182.7	126.8	**-1.43**	0.0001103
ENO1P4	enolase 1 pseudogene 4 [Source:HGNC Symbol;Acc:HGNC:37945]	108.7	75.6	**-1.43**	0.0001312
PDLIM3	PDZ and LIM domain 3 [Source:HGNC Symbol;Acc:HGNC:20767]	150.6	107.4	**-1.43**	0.0002464
ZIM2	zinc finger imprinted 2 [Source:HGNC Symbol;Acc:HGNC:12875]	102.0	70.2	**-1.43**	0.0008706
ZNF853	zinc finger protein 853 [Source:HGNC Symbol;Acc:HGNC:21767]	102.3	70.0	**-1.43**	0.001217
TENM1	teneurin transmembrane protein 1 [Source:HGNC Symbol;Acc:HGNC:8117]	82.7	57.3	**-1.43**	0.002038
TIAF1	TGFB1-induced anti-apoptotic factor 1 [Source:HGNC Symbol;Acc:HGNC:11803]	83.3	57.0	**-1.43**	0.003971
CCDC170	coiled-coil domain containing 170 [Source:HGNC Symbol;Acc:HGNC:21177]	69.1	46.2	**-1.43**	0.005469
PCDHB12	protocadherin beta 12 [Source:HGNC Symbol;Acc:HGNC:8683]	55.2	37.6	**-1.43**	0.007499
TMEM8B	transmembrane protein 8B [Source:HGNC Symbol;Acc:HGNC:21427]	73.1	50.3	**-1.43**	0.01896
CASC10	cancer susceptibility 10 [Source:HGNC Symbol;Acc:HGNC:31448]	54.7	38.6	**-1.43**	0.02388
MAP6D1	MAP6 domain containing 1 [Source:HGNC Symbol;Acc:HGNC:25753]	43.0	28.9	**-1.43**	0.04707
HMGN2	high mobility group nucleosomal binding domain 2 [Source:HGNC Symbol;Acc:HGNC:4986]	3805.4	2647.0	**-1.44**	3.82E-40
RNF145	ring finger protein 145 [Source:HGNC Symbol;Acc:HGNC:20853]	2846.5	1963.9	**-1.44**	3.65E-34
CTSO	cathepsin O [Source:HGNC Symbol;Acc:HGNC:2542]	1410.6	972.0	**-1.44**	1.17E-26
TMX3	thioredoxin related transmembrane protein 3 [Source:HGNC Symbol;Acc:HGNC:24718]	5590.6	3896.7	**-1.44**	4.57E-26
NAP1L3	nucleosome assembly protein 1 like 3 [Source:HGNC Symbol;Acc:HGNC:7639]	908.2	628.8	**-1.44**	1.75E-18
PCDHGA2	protocadherin gamma subfamily A, 2 [Source:HGNC Symbol;Acc:HGNC:8700]	1758.8	1207.8	**-1.44**	7.27E-16
DYNLL1	dynein light chain LC8-type 1 [Source:HGNC Symbol;Acc:HGNC:15476]	3750.0	2625.4	**-1.44**	6.43E-14
ITGA11	integrin subunit alpha 11 [Source:HGNC Symbol;Acc:HGNC:6136]	4016.1	2752.0	**-1.44**	1.55E-13
C11orf95	chromosome 11 open reading frame 95 [Source:HGNC Symbol;Acc:HGNC:28449]	565.9	390.8	**-1.44**	3.39E-13
DUT	deoxyuridine triphosphatase [Source:HGNC Symbol;Acc:HGNC:3078]	1929.6	1355.2	**-1.44**	3.54E-13
HIST1H2BK	histone cluster 1 H2B family member k [Source:HGNC Symbol;Acc:HGNC:13954]	567.0	392.9	**-1.44**	1.74E-12
PCDHGA12	protocadherin gamma subfamily A, 12 [Source:HGNC Symbol;Acc:HGNC:8699]	1515.0	1043.7	**-1.44**	8.70E-12
PCDHGB2	protocadherin gamma subfamily B, 2 [Source:HGNC Symbol;Acc:HGNC:8709]	1525.3	1046.7	**-1.44**	2.21E-11
PCDHGA5	protocadherin gamma subfamily A, 5 [Source:HGNC Symbol;Acc:HGNC:8703]	1540.4	1062.0	**-1.44**	3.62E-11
PCDHGA4	protocadherin gamma subfamily A, 4 [Source:HGNC Symbol;Acc:HGNC:8702]	1524.3	1046.8	**-1.44**	1.07E-10
AUTS2	AUTS2, activator of transcription and developmental regulator [Source:HGNC Symbol;Acc:HGNC:14262]	592.0	402.2	**-1.44**	2.55E-10
IER2	immediate early response 2 [Source:HGNC Symbol;Acc:HGNC:28871]	479.4	332.2	**-1.44**	7.14E-10
PTMAP5	prothymosin alpha pseudogene 5 [Source:HGNC Symbol;Acc:HGNC:9628]	208.6	144.7	**-1.44**	2.12E-08
CGAS	cyclic GMP-AMP synthase [Source:HGNC Symbol;Acc:HGNC:21367]	209.7	144.7	**-1.44**	8.28E-08
EHMT2	euchromatic histone lysine methyltransferase 2 [Source:HGNC Symbol;Acc:HGNC:14129]	370.0	255.4	**-1.44**	5.10E-07
MYH14	myosin heavy chain 14 [Source:HGNC Symbol;Acc:HGNC:23212]	182.0	124.8	**-1.44**	1.75E-06
DLGAP5	DLG associated protein 5 [Source:HGNC Symbol;Acc:HGNC:16864]	545.2	404.2	**-1.44**	1.89E-06
GSDMD	gasdermin D [Source:HGNC Symbol;Acc:HGNC:25697]	183.1	127.8	**-1.44**	1.43E-05
CCHCR1	coiled-coil alpha-helical rod protein 1 [Source:HGNC Symbol;Acc:HGNC:13930]	243.0	168.6	**-1.44**	3.40E-05
CBX4	chromobox 4 [Source:HGNC Symbol;Acc:HGNC:1554]	114.1	78.1	**-1.44**	7.50E-05
SMARCD3	SWI/SNF related, matrix associated, actin dependent regulator of chromatin, subfamily d, member 3 [Sc	144.9	100.1	**-1.44**	8.39E-05
SLC2A4RG	SLC2A4 regulator [Source:HGNC Symbol;Acc:HGNC:15930]	164.2	112.9	**-1.44**	0.0002187
STXBP5-AS1	STXBP5 antisense RNA 1 [Source:HGNC Symbol;Acc:HGNC:44183]	160.7	107.3	**-1.44**	0.0002264
MIEF2	mitochondrial elongation factor 2 [Source:HGNC Symbol;Acc:HGNC:17920]	152.2	103.6	**-1.44**	0.0003978
INO80C	INO80 complex subunit C [Source:HGNC Symbol;Acc:HGNC:26994]	98.4	69.2	**-1.44**	0.001244
TICRR	TOPBP1 interacting checkpoint and replication regulator [Source:HGNC Symbol;Acc:HGNC:28704]	83.6	57.4	**-1.44**	0.003551
HCLS1	hematopoietic cell-specific Lyn substrate 1 [Source:HGNC Symbol;Acc:HGNC:4844]	80.1	54.8	**-1.44**	0.004123
MYEOV	myeloma overexpressed [Source:HGNC Symbol;Acc:HGNC:7563]	78.8	52.3	**-1.44**	0.005916
SPAAR	small regulatory polypeptide of amino acid response [Source:HGNC Symbol;Acc:HGNC:27244]	63.3	44.5	**-1.44**	0.009018
PWWP2B	PWWP domain containing 2B [Source:HGNC Symbol;Acc:HGNC:25150]	54.8	37.8	**-1.44**	0.01233
LHX9	LIM homeobox 9 [Source:HGNC Symbol;Acc:HGNC:14222]	76.5	52.7	**-1.44**	0.01506
CA11	carbonic anhydrase 11 [Source:HGNC Symbol;Acc:HGNC:1370]	60.9	43.0	**-1.44**	0.01783
AFAP1L2	actin filament associated protein 1 like 2 [Source:HGNC Symbol;Acc:HGNC:25901]	44.4	29.7	**-1.44**	0.01966
SEPT6	septin 6 [Source:HGNC Symbol;Acc:HGNC:15848]	959.5	661.5	**-1.45**	2.28E-20

DHRS3	dehydrogenase/reductase 3 [Source:HGNC Symbol;Acc:HGNC:17693]	1237.9	849.7	**-1.45**	7.95E-19
PRMT1	protein arginine methyltransferase 1 [Source:HGNC Symbol;Acc:HGNC:5187]	1088.7	743.4	**-1.45**	2.97E-17
DRAP1	DR1 associated protein 1 [Source:HGNC Symbol;Acc:HGNC:3019]	2352.2	1609.5	**-1.45**	7.99E-17
TUBA1B	tubulin alpha 1b [Source:HGNC Symbol;Acc:HGNC:18809]	10995.4	7671.5	**-1.45**	2.15E-16
LZTS2	leucine zipper tumor suppressor 2 [Source:HGNC Symbol;Acc:HGNC:29381]	562.0	385.0	**-1.45**	7.36E-16
LGR4	leucine rich repeat containing G protein-coupled receptor 4 [Source:HGNC Symbol;Acc:HGNC:13299]	1561.6	1076.7	**-1.45**	6.69E-15
MMP14	matrix metallopeptidase 14 [Source:HGNC Symbol;Acc:HGNC:7160]	8308.4	5615.3	**-1.45**	5.47E-13
PCDHGA10	protocadherin gamma subfamily A, 10 [Source:HGNC Symbol;Acc:HGNC:8697]	1571.2	1074.6	**-1.45**	3.36E-12
NT5DC2	5'-nucleotidase domain containing 2 [Source:HGNC Symbol;Acc:HGNC:25717]	615.2	420.8	**-1.45**	3.66E-12
PCDHGA6	protocadherin gamma subfamily A, 6 [Source:HGNC Symbol;Acc:HGNC:8704]	1578.0	1081.2	**-1.45**	5.19E-12
PCDHGB7	protocadherin gamma subfamily B, 7 [Source:HGNC Symbol;Acc:HGNC:8714]	1650.3	1127.8	**-1.45**	5.24E-12
PCDHGC4	protocadherin gamma subfamily C, 4 [Source:HGNC Symbol;Acc:HGNC:8717]	1490.1	1014.6	**-1.45**	7.95E-12
PCDHGA9	protocadherin gamma subfamily A, 9 [Source:HGNC Symbol;Acc:HGNC:8707]	1546.4	1057.1	**-1.45**	8.20E-12
PCDHGA3	protocadherin gamma subfamily A, 3 [Source:HGNC Symbol;Acc:HGNC:8701]	1528.1	1047.3	**-1.45**	8.66E-12
PCDHGA7	protocadherin gamma subfamily A, 7 [Source:HGNC Symbol;Acc:HGNC:8705]	1519.6	1039.9	**-1.45**	8.75E-12
PCDHGB1	protocadherin gamma subfamily B, 1 [Source:HGNC Symbol;Acc:HGNC:8708]	1500.1	1026.1	**-1.45**	1.08E-11
PCDHGB3	protocadherin gamma subfamily B, 3 [Source:HGNC Symbol;Acc:HGNC:8710]	1512.0	1033.1	**-1.45**	1.09E-11
PCDHGA1	protocadherin gamma subfamily A, 1 [Source:HGNC Symbol;Acc:HGNC:8696]	1497.7	1020.2	**-1.45**	1.54E-11
KNTC1	kinetochore associated 1 [Source:HGNC Symbol;Acc:HGNC:17255]	1148.1	805.8	**-1.45**	2.51E-10
GRK3	G protein-coupled receptor kinase 3 [Source:HGNC Symbol;Acc:HGNC:290]	359.6	246.6	**-1.45**	4.18E-10
LAYN	layilin [Source:HGNC Symbol;Acc:HGNC:29471]	352.7	240.9	**-1.45**	1.70E-09
TECR	trans-2,3-enoyl-CoA reductase [Source:HGNC Symbol;Acc:HGNC:4551]	424.1	292.6	**-1.45**	1.43E-08
DTYMK	deoxythymidylate kinase [Source:HGNC Symbol;Acc:HGNC:3061]	273.3	187.9	**-1.45**	3.95E-08
GPRC5B	G protein-coupled receptor class C group 5 member B [Source:HGNC Symbol;Acc:HGNC:13308]	289.0	197.5	**-1.45**	8.11E-08
AURKA	aurora kinase A [Source:HGNC Symbol;Acc:HGNC:11393]	305.0	218.6	**-1.45**	5.60E-07
PDE3B	phosphodiesterase 3B [Source:HGNC Symbol;Acc:HGNC:8779]	310.2	215.4	**-1.45**	9.31E-07
LONRF2	LON peptidase N-terminal domain and ring finger 2 [Source:HGNC Symbol;Acc:HGNC:24788]	256.7	176.0	**-1.45**	1.34E-06
MYLK	myosin light chain kinase [Source:HGNC Symbol;Acc:HGNC:7590]	393.7	261.4	**-1.45**	9.55E-06
ZBTB7C	zinc finger and BTB domain containing 7C [Source:HGNC Symbol;Acc:HGNC:31700]	120.2	81.9	**-1.45**	0.0002703
IKBKE	inhibitor of nuclear factor kappa B kinase subunit epsilon [Source:HGNC Symbol;Acc:HGNC:14552]	112.3	76.2	**-1.45**	0.0002924
AR	androgen receptor [Source:HGNC Symbol;Acc:HGNC:644]	227.1	154.8	**-1.45**	0.000327
PLCB4	phospholipase C beta 4 [Source:HGNC Symbol;Acc:HGNC:9059]	177.6	121.4	**-1.45**	0.0003736
CYP26B1	cytochrome P450 family 26 subfamily B member 1 [Source:HGNC Symbol;Acc:HGNC:20581]	270.0	188.7	**-1.45**	0.0006006
SEMA3B	semaphorin 3B [Source:HGNC Symbol;Acc:HGNC:10724]	98.2	65.6	**-1.45**	0.001356
AP1S3	adaptor related protein complex 1 subunit sigma 3 [Source:HGNC Symbol;Acc:HGNC:18971]	115.4	78.3	**-1.45**	0.002714
PDE10A	phosphodiesterase 10A [Source:HGNC Symbol;Acc:HGNC:8772]	110.3	72.0	**-1.45**	0.004137
RPS6KA1	ribosomal protein S6 kinase A1 [Source:HGNC Symbol;Acc:HGNC:10430]	100.2	66.6	**-1.45**	0.006999
OSGIN1	oxidative stress induced growth inhibitor 1 [Source:HGNC Symbol;Acc:HGNC:30093]	82.3	55.5	**-1.45**	0.008429
LPCAT4	lysophosphatidylcholine acyltransferase 4 [Source:HGNC Symbol;Acc:HGNC:30059]	53.9	36.8	**-1.45**	0.008792
UBTD1	ubiquitin domain containing 1 [Source:HGNC Symbol;Acc:HGNC:25683]	76.0	50.8	**-1.45**	0.01551
FBXO41	F-box protein 41 [Source:HGNC Symbol;Acc:HGNC:29409]	35.5	24.0	**-1.45**	0.01618
LRRC37A7P	leucine rich repeat containing 37 member A7, pseudogene [Source:HGNC Symbol;Acc:HGNC:43792]	43.1	28.7	**-1.45**	0.0236
PKM	pyruvate kinase M1/2 [Source:HGNC Symbol;Acc:HGNC:9021]	47215.8	32348.1	**-1.46**	8.71E-79
HSPA9	heat shock protein family A (Hsp70) member 9 [Source:HGNC Symbol;Acc:HGNC:5244]	15078.7	10359.3	**-1.46**	4.29E-71
SAMD4A	sterile alpha motif domain containing 4A [Source:HGNC Symbol;Acc:HGNC:23023]	4610.0	3150.3	**-1.46**	2.29E-42
SLC4A2	solute carrier family 4 member 2 [Source:HGNC Symbol;Acc:HGNC:11028]	1347.4	922.7	**-1.46**	3.01E-37
ETS1	ETS proto-oncogene 1, transcription factor [Source:HGNC Symbol;Acc:HGNC:3488]	6400.3	4342.3	**-1.46**	2.51E-24
ERG	ERG, ETS transcription factor [Source:HGNC Symbol;Acc:HGNC:3446]	1994.4	1361.1	**-1.46**	3.31E-23
S100B	S100 calcium binding protein B [Source:HGNC Symbol;Acc:HGNC:10500]	2133.8	1460.0	**-1.46**	8.28E-19
GPX8	glutathione peroxidase 8 (putative) [Source:HGNC Symbol;Acc:HGNC:33100]	3752.3	2583.9	**-1.46**	4.59E-18
FAP	fibroblast activation protein alpha [Source:HGNC Symbol;Acc:HGNC:3590]	5319.0	3745.2	**-1.46**	4.69E-16
CUL7	cullin 7 [Source:HGNC Symbol;Acc:HGNC:21024]	987.8	672.8	**-1.46**	2.00E-15
CNPY4	canopy FGF signaling regulator 4 [Source:HGNC Symbol;Acc:HGNC:28631]	712.5	490.3	**-1.46**	2.74E-15
PCDHGA11	protocadherin gamma subfamily A, 11 [Source:HGNC Symbol;Acc:HGNC:8698]	1624.2	1101.3	**-1.46**	3.02E-13
CDH19	cadherin 19 [Source:HGNC Symbol;Acc:HGNC:1758]	851.7	582.4	**-1.46**	6.26E-13
IRS1	insulin receptor substrate 1 [Source:HGNC Symbol;Acc:HGNC:6125]	1015.6	692.9	**-1.46**	7.78E-12
TRPV4	transient receptor potential cation channel subfamily V member 4 [Source:HGNC Symbol;Acc:HGNC:18(	1748.0	1207.2	**-1.46**	1.50E-11
PDE7B	phosphodiesterase 7B [Source:HGNC Symbol;Acc:HGNC:8792]	777.8	535.4	**-1.46**	7.80E-11
ARHGEF9	Cdc42 guanine nucleotide exchange factor 9 [Source:HGNC Symbol;Acc:HGNC:14561]	451.0	307.6	**-1.46**	4.36E-10
PGAP1	post-GPI attachment to proteins 1 [Source:HGNC Symbol;Acc:HGNC:25712]	353.7	243.2	**-1.46**	2.21E-09
BPHL	biphenyl hydrolase like [Source:HGNC Symbol;Acc:HGNC:1094]	320.6	219.0	**-1.46**	4.25E-09
MRPL14	mitochondrial ribosomal protein L14 [Source:HGNC Symbol;Acc:HGNC:14279]	291.3	197.7	**-1.46**	8.42E-07
TMEM182	transmembrane protein 182 [Source:HGNC Symbol;Acc:HGNC:26391]	148.6	100.9	**-1.46**	1.12E-06
SCML2	Scm polycomb group protein like 2 [Source:HGNC Symbol;Acc:HGNC:10581]	171.2	116.9	**-1.46**	1.14E-06
B9D1	B9 domain containing 1 [Source:HGNC Symbol;Acc:HGNC:24123]	166.0	113.2	**-1.46**	6.03E-06
RAD51B	RAD51 paralog B [Source:HGNC Symbol;Acc:HGNC:9822]	129.8	88.1	**-1.46**	6.26E-06
VIT	vitrin [Source:HGNC Symbol;Acc:HGNC:12697]	206.5	145.9	**-1.46**	2.30E-05
GPR162	G protein-coupled receptor 162 [Source:HGNC Symbol;Acc:HGNC:16693]	105.7	72.1	**-1.46**	0.0007584
LHPP	phospholysine phosphohistidine inorganic pyrophosphate phosphatase [Source:HGNC Symbol;Acc:HGN	84.9	58.2	**-1.46**	0.001754
ABCD1	ATP binding cassette subfamily D member 1 [Source:HGNC Symbol;Acc:HGNC:61]	80.2	54.2	**-1.46**	0.002556
DGLUCY	D-glutamate cyclase [Source:HGNC Symbol;Acc:HGNC:20498]	105.7	73.1	**-1.46**	0.004586
PLEKHN1	pleckstrin homology domain containing N1 [Source:HGNC Symbol;Acc:HGNC:25284]	48.0	32.4	**-1.46**	0.008391

LINC01515	long intergenic non-protein coding RNA 1515 [Source:HGNC Symbol;Acc:HGNC:51210]	53.2	35.8	**-1.46**	0.02287
CATSPER2	cation channel sperm associated 2 [Source:HGNC Symbol;Acc:HGNC:18810]	48.2	32.5	**-1.46**	0.04056
FAM20B	FAM20B, glycosaminoglycan xylosylkinase [Source:HGNC Symbol;Acc:HGNC:23017]	4969.6	3366.6	**-1.47**	2.09E-34
PVR	poliovirus receptor [Source:HGNC Symbol;Acc:HGNC:9705]	3204.5	2172.4	**-1.47**	6.14E-25
SIPA1L1	signal induced proliferation associated 1 like 1 [Source:HGNC Symbol;Acc:HGNC:20284]	3753.6	2527.2	**-1.47**	2.27E-23
PLXNA1	plexin A1 [Source:HGNC Symbol;Acc:HGNC:9099]	1194.8	805.7	**-1.47**	5.78E-23
PCDHGC3	protocadherin gamma subfamily C, 3 [Source:HGNC Symbol;Acc:HGNC:8716]	2841.6	1919.8	**-1.47**	3.27E-19
ALDOC	aldolase, fructose-bisphosphate C [Source:HGNC Symbol;Acc:HGNC:418]	896.7	607.3	**-1.47**	3.08E-18
VCAN	versican [Source:HGNC Symbol;Acc:HGNC:2464]	1815.0	1207.9	**-1.47**	2.25E-16
STK17A	serine/threonine kinase 17a [Source:HGNC Symbol;Acc:HGNC:11395]	6530.3	4357.5	**-1.47**	7.38E-16
SH3YL1	SH3 and SYLF domain containing 1 [Source:HGNC Symbol;Acc:HGNC:29546]	591.0	398.1	**-1.47**	1.29E-15
TNS3	tensin 3 [Source:HGNC Symbol;Acc:HGNC:21616]	5464.7	3768.9	**-1.47**	2.29E-15
PCOLCE	procollagen C-endopeptidase enhancer [Source:HGNC Symbol;Acc:HGNC:8738]	447.4	302.1	**-1.47**	2.99E-15
TTC8	tetratricopeptide repeat domain 8 [Source:HGNC Symbol;Acc:HGNC:20087]	604.7	409.1	**-1.47**	7.64E-15
IQGAP3	IQ motif containing GTPase activating protein 3 [Source:HGNC Symbol;Acc:HGNC:20669]	573.6	394.0	**-1.47**	1.18E-13
CSGALNACT1	chondroitin sulfate N-acetylgalactosaminyltransferase 1 [Source:HGNC Symbol;Acc:HGNC:24290]	6368.3	4336.8	**-1.47**	2.82E-12
PCDHGC5	protocadherin gamma subfamily C, 5 [Source:HGNC Symbol;Acc:HGNC:8718]	1502.0	1015.7	**-1.47**	3.52E-12
ZNF624	zinc finger protein 624 [Source:HGNC Symbol;Acc:HGNC:29254]	320.1	217.0	**-1.47**	8.75E-11
CCNA2	cyclin A2 [Source:HGNC Symbol;Acc:HGNC:1578]	921.6	664.1	**-1.47**	9.32E-09
MOCOS	molybdenum cofactor sulfurase [Source:HGNC Symbol;Acc:HGNC:18234]	314.1	207.6	**-1.47**	1.56E-08
HMGN2P5	high mobility group nucleosomal binding domain 2 pseudogene 5 [Source:HGNC Symbol;Acc:HGNC:335	239.0	163.4	**-1.47**	1.87E-08
HSPG2	heparan sulfate proteoglycan 2 [Source:HGNC Symbol;Acc:HGNC:5273]	2193.0	1490.0	**-1.47**	3.49E-08
PCK2	phosphoenolpyruvate carboxykinase 2, mitochondrial [Source:HGNC Symbol;Acc:HGNC:8725]	549.2	358.8	**-1.47**	2.70E-07
SSBP4	single stranded DNA binding protein 4 [Source:HGNC Symbol;Acc:HGNC:15676]	164.7	111.6	**-1.47**	5.19E-07
HINT2	histidine triad nucleotide binding protein 2 [Source:HGNC Symbol;Acc:HGNC:18344]	161.7	109.8	**-1.47**	1.29E-06
PRKAA2	protein kinase AMP-activated catalytic subunit alpha 2 [Source:HGNC Symbol;Acc:HGNC:9377]	214.7	146.7	**-1.47**	1.32E-06
MOV10	Mov10 RISC complex RNA helicase [Source:HGNC Symbol;Acc:HGNC:7200]	197.9	133.1	**-1.47**	1.74E-06
CAMTA2	calmodulin binding transcription activator 2 [Source:HGNC Symbol;Acc:HGNC:18807]	247.6	165.4	**-1.47**	1.73E-05
CENPW	centromere protein W [Source:HGNC Symbol;Acc:HGNC:21488]	184.4	128.1	**-1.47**	2.06E-05
DDR1	discoidin domain receptor tyrosine kinase 1 [Source:HGNC Symbol;Acc:HGNC:2730]	249.0	169.9	**-1.47**	2.65E-05
DCLRE1B	DNA cross-link repair 1B [Source:HGNC Symbol;Acc:HGNC:17641]	227.6	154.3	**-1.47**	2.70E-05
LDLRAD2	low density lipoprotein receptor class A domain containing 2 [Source:HGNC Symbol;Acc:HGNC:32071]	258.0	174.7	**-1.47**	3.11E-05
BGN	biglycan [Source:HGNC Symbol;Acc:HGNC:1044]	1658.8	1105.5	**-1.47**	0.000147
ZBED5-AS1	ZBED5 antisense RNA 1 [Source:HGNC Symbol;Acc:HGNC:48646]	102.4	67.6	**-1.47**	0.0002388
FAM81A	family with sequence similarity 81 member A [Source:HGNC Symbol;Acc:HGNC:28379]	100.2	68.7	**-1.47**	0.0002838
CKB	creatine kinase B [Source:HGNC Symbol;Acc:HGNC:1991]	127.2	83.5	**-1.47**	0.000299
SCARF2	scavenger receptor class F member 2 [Source:HGNC Symbol;Acc:HGNC:19869]	81.0	53.1	**-1.47**	0.00343
TAF1A-AS1	TAF1A antisense RNA 1 [Source:HGNC Symbol;Acc:HGNC:40573]	68.1	46.1	**-1.47**	0.003509
KLF16	Kruppel like factor 16 [Source:HGNC Symbol;Acc:HGNC:16857]	73.0	48.9	**-1.47**	0.004065
HMGN2P3	high mobility group nucleosomal binding domain 2 pseudogene 3 [Source:HGNC Symbol;Acc:HGNC:335	64.9	43.4	**-1.47**	0.006274
FAM86C2P	family with sequence similarity 86 member C2, pseudogene [Source:HGNC Symbol;Acc:HGNC:42392]	46.5	30.7	**-1.47**	0.01996
ZNF732	zinc finger protein 732 [Source:HGNC Symbol;Acc:HGNC:37138]	62.3	44.8	**-1.47**	0.03361
ZNF93	zinc finger protein 93 [Source:HGNC Symbol;Acc:HGNC:13169]	45.8	30.8	**-1.47**	0.03432
ADORA2B	adenosine A2b receptor [Source:HGNC Symbol;Acc:HGNC:264]	43.6	28.9	**-1.47**	0.04525
EFHB	EF-hand domain family member B [Source:HGNC Symbol;Acc:HGNC:26330]	38.9	25.4	**-1.47**	0.04628
C5orf15	chromosome 5 open reading frame 15 [Source:HGNC Symbol;Acc:HGNC:20656]	4237.8	2852.3	**-1.48**	3.40E-28
KDELR3	KDEL endoplasmic reticulum protein retention receptor 3 [Source:HGNC Symbol;Acc:HGNC:6306]	2105.9	1409.7	**-1.48**	8.23E-28
SNAPC1	small nuclear RNA activating complex polypeptide 1 [Source:HGNC Symbol;Acc:HGNC:11134]	4122.2	2792.6	**-1.48**	4.15E-24
HIST1H1E	histone cluster 1 H1 family member e [Source:HGNC Symbol;Acc:HGNC:4718]	1341.2	903.5	**-1.48**	3.91E-23
FSCN1	fascin actin-bundling protein 1 [Source:HGNC Symbol;Acc:HGNC:11148]	889.2	601.1	**-1.48**	6.07E-20
SLC9A3R2	SLC9A3 regulator 2 [Source:HGNC Symbol;Acc:HGNC:11076]	889.1	600.7	**-1.48**	1.01E-17
ENAH	ENAH, actin regulator [Source:HGNC Symbol;Acc:HGNC:18271]	8300.8	5552.4	**-1.48**	1.10E-17
SLC16A1	solute carrier family 16 member 1 [Source:HGNC Symbol;Acc:HGNC:10922]	2899.6	1980.8	**-1.48**	5.31E-16
SPON1	spondin 1 [Source:HGNC Symbol;Acc:HGNC:11252]	460.1	312.4	**-1.48**	1.58E-14
MAGOHB	mago homolog B, exon junction complex subunit [Source:HGNC Symbol;Acc:HGNC:25504]	880.3	594.6	**-1.48**	2.96E-13
PEG3	paternally expressed 3 [Source:HGNC Symbol;Acc:HGNC:8826]	842.8	560.3	**-1.48**	1.74E-12
CREB5	cAMP responsive element binding protein 5 [Source:HGNC Symbol;Acc:HGNC:16844]	1023.7	686.6	**-1.48**	2.49E-12
HFE	homeostatic iron regulator [Source:HGNC Symbol;Acc:HGNC:4886]	356.9	238.5	**-1.48**	2.49E-12
THAP2	THAP domain containing 2 [Source:HGNC Symbol;Acc:HGNC:20854]	326.6	218.8	**-1.48**	6.17E-12
ECT2	epithelial cell transforming 2 [Source:HGNC Symbol;Acc:HGNC:3155]	1132.5	786.6	**-1.48**	1.25E-10
NOCT	nocturnin [Source:HGNC Symbol;Acc:HGNC:14254]	217.0	146.3	**-1.48**	4.75E-10
CENPE	centromere protein E [Source:HGNC Symbol;Acc:HGNC:1856]	815.0	581.3	**-1.48**	1.05E-09
MGAT4A	mannosyl (alpha-1,3-)-glycoprotein beta-1,4-N-acetylglucosaminyltransferase, isozyme A [Source:HGNC	217.0	146.1	**-1.48**	7.95E-09
SERPINB9	serpin family B member 9 [Source:HGNC Symbol;Acc:HGNC:8955]	305.2	206.8	**-1.48**	4.31E-08
FAM86DP	family with sequence similarity 86 member D, pseudogene [Source:HGNC Symbol;Acc:HGNC:32659]	227.4	152.3	**-1.48**	2.15E-07
TMEM200B	transmembrane protein 200B [Source:HGNC Symbol;Acc:HGNC:33785]	222.9	152.7	**-1.48**	5.69E-07
MMS22L	MMS22 like, DNA repair protein [Source:HGNC Symbol;Acc:HGNC:21475]	501.7	343.7	**-1.48**	1.04E-06
NDUFS8	NADH:ubiquinone oxidoreductase core subunit S8 [Source:HGNC Symbol;Acc:HGNC:7715]	138.5	92.9	**-1.48**	3.51E-05
KCNC4	potassium voltage-gated channel subfamily C member 4 [Source:HGNC Symbol;Acc:HGNC:6236]	105.1	70.4	**-1.48**	0.0005695
CD37	CD37 molecule [Source:HGNC Symbol;Acc:HGNC:1666]	69.5	46.4	**-1.48**	0.0006252
UAP1L1	UDP-N-acetylglucosamine pyrophosphorylase 1 like 1 [Source:HGNC Symbol;Acc:HGNC:28082]	106.0	70.8	**-1.48**	0.0008371
THSD7A	thrombospondin type 1 domain containing 7A [Source:HGNC Symbol;Acc:HGNC:22207]	137.0	92.7	**-1.48**	0.003991

TFR2	transferrin receptor 2 [Source:HGNC Symbol;Acc:HGNC:11762]	36.8	24.9	**-1.48**	0.01652
KHDC1	KH domain containing 1 [Source:HGNC Symbol;Acc:HGNC:21366]	52.5	33.8	**-1.48**	0.02199
SPON1-AS1	SPON1 antisense RNA 1 [Source:HGNC Symbol;Acc:HGNC:53117]	33.8	22.2	**-1.48**	0.02851
ARSJ	arylsulfatase family member J [Source:HGNC Symbol;Acc:HGNC:26286]	4686.8	3133.8	**-1.49**	2.67E-29
HMGN1	high mobility group nucleosome binding domain 1 [Source:HGNC Symbol;Acc:HGNC:4984]	3065.6	2068.5	**-1.49**	2.75E-29
TEX9	testis expressed 9 [Source:HGNC Symbol;Acc:HGNC:29585]	884.5	590.0	**-1.49**	1.53E-27
PFKFB3	6-phosphofructo-2-kinase/fructose-2,6-biphosphatase 3 [Source:HGNC Symbol;Acc:HGNC:8874]	1723.8	1153.9	**-1.49**	3.60E-26
SMAD7	SMAD family member 7 [Source:HGNC Symbol;Acc:HGNC:6773]	824.2	549.8	**-1.49**	1.14E-23
ROBO1	roundabout guidance receptor 1 [Source:HGNC Symbol;Acc:HGNC:10249]	2159.2	1441.0	**-1.49**	2.65E-21
GCLM	glutamate-cysteine ligase modifier subunit [Source:HGNC Symbol;Acc:HGNC:4312]	1531.4	1016.7	**-1.49**	2.04E-20
SAMD9L	sterile alpha motif domain containing 9 like [Source:HGNC Symbol;Acc:HGNC:1349]	1556.6	1036.5	**-1.49**	1.43E-17
ARMCX6	armadillo repeat containing X-linked 6 [Source:HGNC Symbol;Acc:HGNC:26094]	547.8	366.4	**-1.49**	1.23E-16
PPP4R4	protein phosphatase 4 regulatory subunit 4 [Source:HGNC Symbol;Acc:HGNC:23788]	750.5	504.8	**-1.49**	2.83E-14
SOX6	SRY-box 6 [Source:HGNC Symbol;Acc:HGNC:16421]	627.8	414.9	**-1.49**	3.89E-13
TBC1D24	TBC1 domain family member 24 [Source:HGNC Symbol;Acc:HGNC:29203]	280.8	187.6	**-1.49**	3.42E-12
ARL10	ADP ribosylation factor like GTPase 10 [Source:HGNC Symbol;Acc:HGNC:22042]	455.9	307.8	**-1.49**	5.28E-12
KIF20B	kinesin family member 20B [Source:HGNC Symbol;Acc:HGNC:7212]	945.6	661.4	**-1.49**	6.82E-11
LLGL1	LLGL1, scribble cell polarity complex component [Source:HGNC Symbol;Acc:HGNC:6628]	296.2	196.0	**-1.49**	6.50E-10
ARHGAP22	Rho GTPase activating protein 22 [Source:HGNC Symbol;Acc:HGNC:30320]	692.6	467.6	**-1.49**	2.33E-09
APOL3	apolipoprotein L3 [Source:HGNC Symbol;Acc:HGNC:14868]	292.5	196.4	**-1.49**	4.90E-09
DISP1	dispatched RND transporter family member 1 [Source:HGNC Symbol;Acc:HGNC:19711]	270.5	178.1	**-1.49**	1.56E-08
ADCY7	adenylate cyclase 7 [Source:HGNC Symbol;Acc:HGNC:238]	364.3	242.2	**-1.49**	1.93E-08
PRELID2	PRELI domain containing 2 [Source:HGNC Symbol;Acc:HGNC:28306]	217.8	143.7	**-1.49**	3.90E-07
PCDHB5	protocadherin beta 5 [Source:HGNC Symbol;Acc:HGNC:8690]	161.7	108.0	**-1.49**	4.47E-07
RCCD1	RCC1 domain containing 1 [Source:HGNC Symbol;Acc:HGNC:30457]	202.1	135.2	**-1.49**	1.19E-06
CLN6	CLN6, transmembrane ER protein [Source:HGNC Symbol;Acc:HGNC:2077]	182.8	122.5	**-1.49**	1.63E-06
KIF2C	kinesin family member 2C [Source:HGNC Symbol;Acc:HGNC:6393]	288.4	201.3	**-1.49**	4.57E-06
AEBP1	AE binding protein 1 [Source:HGNC Symbol;Acc:HGNC:303]	4800.5	3212.4	**-1.49**	8.97E-06
SLC16A9	solute carrier family 16 member 9 [Source:HGNC Symbol;Acc:HGNC:23520]	169.0	112.1	**-1.49**	1.20E-05
PLEKHG4	pleckstrin homology and RhoGEF domain containing G4 [Source:HGNC Symbol;Acc:HGNC:24501]	91.0	59.7	**-1.49**	0.0005289
PEG10	paternally expressed 10 [Source:HGNC Symbol;Acc:HGNC:14005]	73.5	48.3	**-1.49**	0.00132
LDOC1	LDOC1, regulator of NFKB signaling [Source:HGNC Symbol;Acc:HGNC:6548]	63.9	41.6	**-1.49**	0.001387
KIF18A	kinesin family member 18A [Source:HGNC Symbol;Acc:HGNC:29441]	153.2	105.6	**-1.49**	0.002373
ATP2B1-AS1	ATP2B1 antisense RNA 1 [Source:HGNC Symbol;Acc:HGNC:27883]	71.6	46.7	**-1.49**	0.005447
D2HGDH	D-2-hydroxyglutarate dehydrogenase [Source:HGNC Symbol;Acc:HGNC:28358]	67.2	44.9	**-1.49**	0.009518
SNORD86	small nucleolar RNA, C/D box 86 [Source:HGNC Symbol;Acc:HGNC:32745]	51.0	34.1	**-1.49**	0.02425
BSN	bassoon presynaptic cytomatrix protein [Source:HGNC Symbol;Acc:HGNC:1117]	31.4	20.1	**-1.49**	0.04183
RBM38	RNA binding motif protein 38 [Source:HGNC Symbol;Acc:HGNC:15818]	26.2	16.8	**-1.49**	0.04657
MAGED2	MAGE family member D2 [Source:HGNC Symbol;Acc:HGNC:16353]	10661.6	7090.6	**-1.51**	1.25E-48
PENK	proenkephalin [Source:HGNC Symbol;Acc:HGNC:8831]	4479.2	2981.9	**-1.51**	6.42E-28
TUBB	tubulin beta class I [Source:HGNC Symbol;Acc:HGNC:20778]	11479.6	7698.7	**-1.51**	2.35E-22
PEX13	peroxisomal biogenesis factor 13 [Source:HGNC Symbol;Acc:HGNC:8855]	2462.0	1607.7	**-1.51**	2.44E-19
BARD1	BRCA1 associated RING domain 1 [Source:HGNC Symbol;Acc:HGNC:952]	962.2	647.3	**-1.51**	3.61E-18
H2AFZ	H2A histone family member Z [Source:HGNC Symbol;Acc:HGNC:4741]	2515.7	1681.2	**-1.51**	1.17E-17
KBTBD7	kelch repeat and BTB domain containing 7 [Source:HGNC Symbol;Acc:HGNC:25266]	773.8	514.7	**-1.51**	2.53E-14
HSPA4L	heat shock protein family A (Hsp70) member 4 like [Source:HGNC Symbol;Acc:HGNC:17041]	385.1	258.0	**-1.51**	2.95E-11
CCDC8	coiled-coil domain containing 8 [Source:HGNC Symbol;Acc:HGNC:25367]	493.6	329.7	**-1.51**	2.05E-10
GYPC	glycophorin C (Gerbich blood group) [Source:HGNC Symbol;Acc:HGNC:4704]	700.0	460.5	**-1.51**	6.81E-10
TMPO-AS1	TMPO antisense RNA 1 [Source:HGNC Symbol;Acc:HGNC:44158]	267.9	177.6	**-1.51**	6.38E-09
CHEK1	checkpoint kinase 1 [Source:HGNC Symbol;Acc:HGNC:1925]	742.3	510.4	**-1.51**	1.72E-08
RNASEH2A	ribonuclease H2 subunit A [Source:HGNC Symbol;Acc:HGNC:18518]	388.6	259.3	**-1.51**	2.89E-08
CHAF1A	chromatin assembly factor 1 subunit A [Source:HGNC Symbol;Acc:HGNC:1910]	368.3	249.6	**-1.51**	3.69E-07
SH3BGRL2	SH3 domain binding glutamate rich protein like 2 [Source:HGNC Symbol;Acc:HGNC:15567]	132.1	86.4	**-1.51**	6.76E-07
TCEAL6	transcription elongation factor A like 6 [Source:HGNC Symbol;Acc:HGNC:24553]	133.8	87.4	**-1.51**	2.60E-06
EVA1B	eva-1 homolog B [Source:HGNC Symbol;Acc:HGNC:25558]	266.1	175.8	**-1.51**	3.50E-06
EFNB1	ephrin B1 [Source:HGNC Symbol;Acc:HGNC:3226]	106.1	69.8	**-1.51**	0.0001109
TSPY26P	testis specific protein Y-linked 26, pseudogene [Source:HGNC Symbol;Acc:HGNC:16256]	103.9	69.1	**-1.51**	0.0001194
FDXACB1	ferredoxin-fold anticodon binding domain containing 1 [Source:HGNC Symbol;Acc:HGNC:25110]	79.1	52.5	**-1.51**	0.0007249
CLEC11A	C-type lectin domain containing 11A [Source:HGNC Symbol;Acc:HGNC:10576]	68.7	45.6	**-1.51**	0.0108
ALDH3A1	aldehyde dehydrogenase 3 family member A1 [Source:HGNC Symbol;Acc:HGNC:405]	35.4	22.3	**-1.51**	0.02954
PSIP1	PC4 and SFRS1 interacting protein 1 [Source:HGNC Symbol;Acc:HGNC:9527]	2227.4	1472.2	**-1.52**	1.90E-36
KDELC2	KDEL motif containing 2 [Source:HGNC Symbol;Acc:HGNC:28496]	4428.9	2951.8	**-1.52**	1.04E-33
LDLRAD4	low density lipoprotein receptor class A domain containing 4 [Source:HGNC Symbol;Acc:HGNC:1224]	1076.7	713.9	**-1.52**	3.09E-26
DZIP1	DAZ interacting zinc finger protein 1 [Source:HGNC Symbol;Acc:HGNC:20908]	1024.1	670.6	**-1.52**	2.39E-25
STK26	serine/threonine kinase 26 [Source:HGNC Symbol;Acc:HGNC:18174]	16209.9	10679.0	**-1.52**	1.45E-24
CTSC	cathepsin C [Source:HGNC Symbol;Acc:HGNC:2528]	1663.5	1085.1	**-1.52**	2.41E-21
DLEU2	deleted in lymphocytic leukemia 2 (non-protein coding) [Source:HGNC Symbol;Acc:HGNC:13748]	357.4	236.3	**-1.52**	6.71E-14
RFX5	regulatory factor X5 [Source:HGNC Symbol;Acc:HGNC:9986]	319.0	208.7	**-1.52**	4.69E-12
MIR222HG	MIR222 host gene [Source:HGNC Symbol;Acc:HGNC:49555]	460.7	313.2	**-1.52**	8.52E-12
SUV39H2	suppressor of variegation 3-9 homolog 2 [Source:HGNC Symbol;Acc:HGNC:17287]	388.2	258.7	**-1.52**	2.56E-09
RBL1	RB transcriptional corepressor like 1 [Source:HGNC Symbol;Acc:HGNC:9893]	986.6	669.3	**-1.52**	3.87E-08
CCNB2	cyclin B2 [Source:HGNC Symbol;Acc:HGNC:1580]	288.3	197.2	**-1.52**	1.61E-07

MBLAC2	metallo-beta-lactamase domain containing 2 [Source:HGNC Symbol;Acc:HGNC:33711]	230.6	151.8	**-1.52**	2.94E-07
AADAT	aminoadipate aminotransferase [Source:HGNC Symbol;Acc:HGNC:17929]	146.7	97.4	**-1.52**	5.84E-06
MBOAT7	membrane bound O-acyltransferase domain containing 7 [Source:HGNC Symbol;Acc:HGNC:15505]	88.4	57.2	**-1.52**	0.0001846
DLG4	discs large MAGUK scaffold protein 4 [Source:HGNC Symbol;Acc:HGNC:2903]	94.6	60.1	**-1.52**	0.0001874
SERTAD4-AS1	SERTAD4 antisense RNA 1 [Source:HGNC Symbol;Acc:HGNC:32019]	84.5	55.1	**-1.52**	0.0002059
DOK5	docking protein 5 [Source:HGNC Symbol;Acc:HGNC:16173]	71.5	46.9	**-1.52**	0.0006194
THEM5	thioesterase superfamily member 5 [Source:HGNC Symbol;Acc:HGNC:26755]	76.3	49.6	**-1.52**	0.0008805
FKBPL	FK506 binding protein like [Source:HGNC Symbol;Acc:HGNC:13949]	76.1	49.6	**-1.52**	0.00233
ZNF682	zinc finger protein 682 [Source:HGNC Symbol;Acc:HGNC:28857]	84.5	56.0	**-1.52**	0.003077
CD274	CD274 molecule [Source:HGNC Symbol;Acc:HGNC:17635]	52.3	33.6	**-1.52**	0.01285
THAP8	THAP domain containing 8 [Source:HGNC Symbol;Acc:HGNC:23191]	46.8	29.9	**-1.52**	0.01443
C12orf40	chromosome 12 open reading frame 40 [Source:HGNC Symbol;Acc:HGNC:26846]	28.8	18.6	**-1.52**	0.02486
SLC16A14	solute carrier family 16 member 14 [Source:HGNC Symbol;Acc:HGNC:26417]	31.8	20.2	**-1.52**	0.02913
LINC01011	long intergenic non-protein coding RNA 1011 [Source:HGNC Symbol;Acc:HGNC:33812]	30.1	19.4	**-1.52**	0.04715
FGFRL1	fibroblast growth factor receptor like 1 [Source:HGNC Symbol;Acc:HGNC:3693]	6999.0	4600.2	**-1.53**	4.45E-50
KLHL5	kelch like family member 5 [Source:HGNC Symbol;Acc:HGNC:6356]	992.6	652.3	**-1.53**	6.43E-30
SH3RF1	SH3 domain containing ring finger 1 [Source:HGNC Symbol;Acc:HGNC:17650]	1037.8	676.3	**-1.53**	2.68E-29
CPEB4	cytoplasmic polyadenylation element binding protein 4 [Source:HGNC Symbol;Acc:HGNC:21747]	2534.1	1660.4	**-1.53**	4.24E-26
RARG	retinoic acid receptor gamma [Source:HGNC Symbol;Acc:HGNC:9866]	1730.2	1137.5	**-1.53**	1.20E-25
SLC35B2	solute carrier family 35 member B2 [Source:HGNC Symbol;Acc:HGNC:16872]	895.4	582.6	**-1.53**	1.20E-17
EXTL2	exostosin like glycosyltransferase 2 [Source:HGNC Symbol;Acc:HGNC:3516]	1228.4	801.8	**-1.53**	1.20E-17
NFIX	nuclear factor I X [Source:HGNC Symbol;Acc:HGNC:7788]	1143.0	751.6	**-1.53**	1.14E-16
TSPAN5	tetraspanin 5 [Source:HGNC Symbol;Acc:HGNC:17753]	1105.5	733.3	**-1.53**	3.79E-16
UBE2S	ubiquitin conjugating enzyme E2 S [Source:HGNC Symbol;Acc:HGNC:17895]	351.1	228.4	**-1.53**	1.38E-14
ARNTL2	aryl hydrocarbon receptor nuclear translocator like 2 [Source:HGNC Symbol;Acc:HGNC:18984]	1048.1	683.9	**-1.53**	5.19E-13
PMF1-BGLAP	PMF1-BGLAP readthrough [Source:HGNC Symbol;Acc:HGNC:42953]	329.9	215.4	**-1.53**	1.18E-12
RGP1	RGP1 homolog, RAB6A GEF complex partner 1 [Source:HGNC Symbol;Acc:HGNC:21965]	3347.3	2230.4	**-1.53**	3.92E-12
LRP5	LDL receptor related protein 5 [Source:HGNC Symbol;Acc:HGNC:6697]	337.3	221.2	**-1.53**	6.15E-12
NFAT5	nuclear factor of activated T cells 5 [Source:HGNC Symbol;Acc:HGNC:7774]	2459.0	1595.8	**-1.53**	3.73E-11
HYAL2	hyaluronoglucosaminidase 2 [Source:HGNC Symbol;Acc:HGNC:5321]	387.5	254.4	**-1.53**	5.69E-11
ITGA6	integrin subunit alpha 6 [Source:HGNC Symbol;Acc:HGNC:6142]	2382.8	1534.4	**-1.53**	5.85E-11
PDE4B	phosphodiesterase 4B [Source:HGNC Symbol;Acc:HGNC:8781]	1911.5	1261.8	**-1.53**	8.13E-09
RHNO1	RAD9-HUS1-RAD1 interacting nuclear orphan 1 [Source:HGNC Symbol;Acc:HGNC:28206]	239.6	159.0	**-1.53**	6.48E-08
POLA2	DNA polymerase alpha 2, accessory subunit [Source:HGNC Symbol;Acc:HGNC:30073]	214.7	141.6	**-1.53**	2.07E-07
NEK2	NIMA related kinase 2 [Source:HGNC Symbol;Acc:HGNC:7745]	303.7	212.6	**-1.53**	5.90E-07
PECR	peroxisomal trans-2-enoyl-CoA reductase [Source:HGNC Symbol;Acc:HGNC:18281]	256.0	164.2	**-1.53**	7.68E-07
FOXP4	forkhead box P4 [Source:HGNC Symbol;Acc:HGNC:20842]	138.7	90.6	**-1.53**	1.11E-06
GALNT12	polypeptide N-acetylgalactosaminyltransferase 12 [Source:HGNC Symbol;Acc:HGNC:19877]	276.0	174.8	**-1.53**	2.12E-06
ZNF204P	zinc finger protein 204, pseudogene [Source:HGNC Symbol;Acc:HGNC:12995]	134.7	89.0	**-1.53**	0.0001007
ABCA13	ATP binding cassette subfamily A member 13 [Source:HGNC Symbol;Acc:HGNC:14638]	177.2	111.9	**-1.53**	0.0001534
ANKRD39	ankyrin repeat domain 39 [Source:HGNC Symbol;Acc:HGNC:28640]	92.7	60.3	**-1.53**	0.0004116
PMAIP1	phorbol-12-myristate-13-acetate-induced protein 1 [Source:HGNC Symbol;Acc:HGNC:9108]	72.3	47.5	**-1.53**	0.002209
CPT1C	carnitine palmitoyltransferase 1C [Source:HGNC Symbol;Acc:HGNC:18540]	73.4	47.4	**-1.53**	0.002209
FAM169A	family with sequence similarity 169 member A [Source:HGNC Symbol;Acc:HGNC:29138]	65.4	42.3	**-1.53**	0.005094
LSR	lipolysis stimulated lipoprotein receptor [Source:HGNC Symbol;Acc:HGNC:29572]	30.5	19.2	**-1.53**	0.01287
DEF6	DEF6, guanine nucleotide exchange factor [Source:HGNC Symbol;Acc:HGNC:2760]	58.6	37.2	**-1.53**	0.0166
CD4	CD4 molecule [Source:HGNC Symbol;Acc:HGNC:1678]	38.8	25.1	**-1.53**	0.02063
ARHGAP39	Rho GTPase activating protein 39 [Source:HGNC Symbol;Acc:HGNC:29351]	28.9	18.2	**-1.53**	0.02407
ACER2	alkaline ceramidase 2 [Source:HGNC Symbol;Acc:HGNC:23675]	25.2	15.7	**-1.53**	0.03634
MAPRE2	microtubule associated protein RP/EB family member 2 [Source:HGNC Symbol;Acc:HGNC:6891]	1501.9	979.9	**-1.54**	8.71E-28
MAGED1	MAGE family member D1 [Source:HGNC Symbol;Acc:HGNC:6813]	2821.8	1830.1	**-1.54**	8.63E-27
MYO18A	myosin XVIIIA [Source:HGNC Symbol;Acc:HGNC:31104]	931.9	605.9	**-1.54**	1.15E-23
ACVR1	activin A receptor type 1 [Source:HGNC Symbol;Acc:HGNC:171]	2187.0	1431.9	**-1.54**	1.18E-23
HNMT	histamine N-methyltransferase [Source:HGNC Symbol;Acc:HGNC:5028]	1319.4	875.3	**-1.54**	4.28E-18
NEMP1	nuclear envelope integral membrane protein 1 [Source:HGNC Symbol;Acc:HGNC:29001]	890.4	588.9	**-1.54**	2.16E-16
PMF1	polyamine modulated factor 1 [Source:HGNC Symbol;Acc:HGNC:9112]	362.8	235.8	**-1.54**	8.50E-16
LAPTM4B	lysosomal protein transmembrane 4 beta [Source:HGNC Symbol;Acc:HGNC:13646]	997.3	645.5	**-1.54**	8.81E-16
ZNF704	zinc finger protein 704 [Source:HGNC Symbol;Acc:HGNC:32291]	1708.2	1070.9	**-1.54**	1.06E-13
MXRA8	matrix remodeling associated 8 [Source:HGNC Symbol;Acc:HGNC:7542]	1073.2	702.0	**-1.54**	7.93E-12
FAM120C	family with sequence similarity 120C [Source:HGNC Symbol;Acc:HGNC:16949]	403.9	265.5	**-1.54**	1.28E-09
FAM216A	family with sequence similarity 216 member A [Source:HGNC Symbol;Acc:HGNC:30180]	245.0	156.5	**-1.54**	5.75E-09
SPTLC3	serine palmitoyltransferase long chain base subunit 3 [Source:HGNC Symbol;Acc:HGNC:16253]	459.3	308.4	**-1.54**	9.45E-09
BPI	bactericidal permeability increasing protein [Source:HGNC Symbol;Acc:HGNC:1095]	189.9	123.7	**-1.54**	3.17E-08
IL11RA	interleukin 11 receptor subunit alpha [Source:HGNC Symbol;Acc:HGNC:5967]	136.3	88.1	**-1.54**	2.59E-07
SSBP3	single stranded DNA binding protein 3 [Source:HGNC Symbol;Acc:HGNC:15674]	153.1	99.0	**-1.54**	3.89E-07
BAIAP2-DT	BAIAP2 divergent transcript [Source:HGNC Symbol;Acc:HGNC:44342]	206.2	162.1	**-1.54**	8.66E-07
RP9P	RP9 pseudogene [Source:HGNC Symbol;Acc:HGNC:33969]	146.1	95.0	**-1.54**	2.59E-06
SLC9A1	solute carrier family 9 member A1 [Source:HGNC Symbol;Acc:HGNC:11071]	162.0	104.2	**-1.54**	1.39E-05
SAC3D1	SAC3 domain containing 1 [Source:HGNC Symbol;Acc:HGNC:30179]	129.4	84.2	**-1.54**	1.47E-05
VWC2	von Willebrand factor C domain containing 2 [Source:HGNC Symbol;Acc:HGNC:30200]	76.7	49.2	**-1.54**	0.0002441
RAET1G	retinoic acid early transcript 1G [Source:HGNC Symbol;Acc:HGNC:16795]	85.7	55.1	**-1.54**	0.0004102
ARHGEF25	Rho guanine nucleotide exchange factor 25 [Source:HGNC Symbol;Acc:HGNC:30275]	90.9	58.4	**-1.54**	0.0006143

GLIS2	GLIS family zinc finger 2 [Source:HGNC Symbol;Acc:HGNC:29450]	65.3	41.9	**-1.54**	0.001157
SELL	selectin L [Source:HGNC Symbol;Acc:HGNC:10720]	58.9	37.9	**-1.54**	0.002481
NOTCH3	notch 3 [Source:HGNC Symbol;Acc:HGNC:7883]	55.5	35.5	**-1.54**	0.002498
CCDC136	coiled-coil domain containing 136 [Source:HGNC Symbol;Acc:HGNC:22225]	60.5	38.2	**-1.54**	0.002603
PCDH12	protocadherin 12 [Source:HGNC Symbol;Acc:HGNC:8657]	57.3	36.1	**-1.54**	0.003571
NINJ2	ninjurin 2 [Source:HGNC Symbol;Acc:HGNC:7825]	46.4	29.2	**-1.54**	0.007333
FAM43B	family with sequence similarity 43 member B [Source:HGNC Symbol;Acc:HGNC:31791]	41.3	26.4	**-1.54**	0.01415
PKDREJ	polycystin family receptor for egg jelly [Source:HGNC Symbol;Acc:HGNC:9015]	30.1	18.7	**-1.54**	0.0328
PDZD4	PDZ domain containing 4 [Source:HGNC Symbol;Acc:HGNC:21167]	24.4	15.3	**-1.54**	0.04184
FAT1	FAT atypical cadherin 1 [Source:HGNC Symbol;Acc:HGNC:3595]	14257.4	9202.3	**-1.55**	5.69E-47
P4HA1	prolyl 4-hydroxylase subunit alpha 1 [Source:HGNC Symbol;Acc:HGNC:8546]	16644.2	10720.0	**-1.55**	3.79E-39
EMILIN1	elastin microfibril interfacer 1 [Source:HGNC Symbol;Acc:HGNC:19880]	3288.3	2138.7	**-1.55**	1.35E-24
DDR2	discoidin domain receptor tyrosine kinase 2 [Source:HGNC Symbol;Acc:HGNC:2731]	15905.5	10417.8	**-1.55**	3.26E-24
CTSD	cathepsin D [Source:HGNC Symbol;Acc:HGNC:2529]	5250.5	3451.9	**-1.55**	1.83E-19
ASNS	asparagine synthetase (glutamine-hydrolyzing) [Source:HGNC Symbol;Acc:HGNC:753]	2468.0	1568.9	**-1.55**	1.22E-14
PSMC3IP	PSMC3 interacting protein [Source:HGNC Symbol;Acc:HGNC:17928]	652.9	424.9	**-1.55**	1.55E-14
DMPK	DM1 protein kinase [Source:HGNC Symbol;Acc:HGNC:2933]	873.1	568.6	**-1.55**	9.26E-10
CRYZL2P	crystallin zeta like 2, pseudogene [Source:HGNC Symbol;Acc:HGNC:52164]	184.2	119.0	**-1.55**	6.14E-09
C17orf107	chromosome 17 open reading frame 107 [Source:HGNC Symbol;Acc:HGNC:37238]	233.0	149.5	**-1.55**	3.19E-08
PDLIM2	PDZ and LIM domain 2 [Source:HGNC Symbol;Acc:HGNC:13992]	177.9	112.9	**-1.55**	6.84E-08
CCDC134	coiled-coil domain containing 134 [Source:HGNC Symbol;Acc:HGNC:26185]	125.8	80.8	**-1.55**	9.42E-07
HSPA12A	heat shock protein family A (Hsp70) member 12A [Source:HGNC Symbol;Acc:HGNC:19022]	119.1	75.9	**-1.55**	2.47E-06
LINC01943	long intergenic non-protein coding RNA 1943 [Source:HGNC Symbol;Acc:HGNC:52767]	121.3	78.7	**-1.55**	5.97E-06
PPP2R3B	protein phosphatase 2 regulatory subunit B''beta [Source:HGNC Symbol;Acc:HGNC:13417]	95.9	62.0	**-1.55**	2.70E-05
THSD1	thrombospondin type 1 domain containing 1 [Source:HGNC Symbol;Acc:HGNC:17754]	88.5	56.7	**-1.55**	2.97E-05
PLEKHG1	pleckstrin homology and RhoGEF domain containing G1 [Source:HGNC Symbol;Acc:HGNC:20884]	103.1	67.1	**-1.55**	3.41E-05
FANCB	Fanconi anemia complementation group B [Source:HGNC Symbol;Acc:HGNC:3583]	101.5	65.3	**-1.55**	5.36E-05
IL27RA	interleukin 27 receptor subunit alpha [Source:HGNC Symbol;Acc:HGNC:17290]	95.8	61.1	**-1.55**	0.0001144
LRRC45	leucine rich repeat containing 45 [Source:HGNC Symbol;Acc:HGNC:28302]	102.0	63.2	**-1.55**	0.0004849
RELT	RELT, TNF receptor [Source:HGNC Symbol;Acc:HGNC:13764]	86.4	55.2	**-1.55**	0.000545
PLD6	phospholipase D family member 6 [Source:HGNC Symbol;Acc:HGNC:30447]	75.0	46.4	**-1.55**	0.0007347
GTSE1	G2 and S-phase expressed 1 [Source:HGNC Symbol;Acc:HGNC:13698]	103.0	68.0	**-1.55**	0.0009279
APOBEC3G	apolipoprotein B mRNA editing enzyme catalytic subunit 3G [Source:HGNC Symbol;Acc:HGNC:17357]	69.8	44.1	**-1.55**	0.001065
SOX11	SRY-box 11 [Source:HGNC Symbol;Acc:HGNC:11191]	76.4	45.9	**-1.55**	0.00136
LAPTM5	lysosomal protein transmembrane 5 [Source:HGNC Symbol;Acc:HGNC:29612]	52.3	33.1	**-1.55**	0.003365
OLMALINC	oligodendrocyte maturation-associated long intergenic non-coding RNA [Source:HGNC Symbol;Acc:HGN	34.1	21.6	**-1.55**	0.008242
CAMK4	calcium/calmodulin dependent protein kinase IV [Source:HGNC Symbol;Acc:HGNC:1464]	37.1	24.0	**-1.55**	0.02287
RAB9B	RAB9B, member RAS oncogene family [Source:HGNC Symbol;Acc:HGNC:14090]	42.3	27.6	**-1.55**	0.02698
ASTN2	astrotactin 2 [Source:HGNC Symbol;Acc:HGNC:17021]	27.2	16.6	**-1.55**	0.04563
RPS6KA3	ribosomal protein S6 kinase A3 [Source:HGNC Symbol;Acc:HGNC:10432]	3243.0	2080.7	**-1.56**	9.25E-90
HDAC9	histone deacetylase 9 [Source:HGNC Symbol;Acc:HGNC:14065]	1305.2	838.2	**-1.56**	1.01E-36
KIRREL1	kirre like nephrin family adhesion molecule 1 [Source:HGNC Symbol;Acc:HGNC:15734]	2660.0	1704.1	**-1.56**	7.42E-36
DYRK2	dual specificity tyrosine phosphorylation regulated kinase 2 [Source:HGNC Symbol;Acc:HGNC:3093]	1199.1	766.9	**-1.56**	1.60E-34
PGAM1	phosphoglycerate mutase 1 [Source:HGNC Symbol;Acc:HGNC:8888]	1904.1	1222.6	**-1.56**	7.74E-26
B4GALT6	beta-1,4-galactosyltransferase 6 [Source:HGNC Symbol;Acc:HGNC:929]	1018.9	653.4	**-1.56**	2.42E-19
MATN3	matrilin 3 [Source:HGNC Symbol;Acc:HGNC:6909]	1883.2	1198.1	**-1.56**	3.91E-18
SYNM	synemin [Source:HGNC Symbol;Acc:HGNC:24466]	4579.6	2958.0	**-1.56**	4.04E-15
TWISTNB	TWIST neighbor [Source:HGNC Symbol;Acc:HGNC:18027]	2671.4	1686.4	**-1.56**	1.11E-14
GOT1	glutamic-oxaloacetic transaminase 1 [Source:HGNC Symbol;Acc:HGNC:4432]	1030.3	646.5	**-1.56**	1.24E-14
DDAH2	dimethylarginine dimethylaminohydrolase 2 [Source:HGNC Symbol;Acc:HGNC:2716]	342.1	219.7	**-1.56**	4.51E-11
TBPL1	TATA-box binding protein like 1 [Source:HGNC Symbol;Acc:HGNC:11589]	695.9	427.5	**-1.56**	9.94E-11
BOC	BOC cell adhesion associated, oncogene regulated [Source:HGNC Symbol;Acc:HGNC:17173]	815.0	544.3	**-1.56**	1.54E-10
CD55	CD55 molecule (Cromer blood group) [Source:HGNC Symbol;Acc:HGNC:2665]	22856.9	14240.9	**-1.56**	1.02E-08
ERCC2	ERCC excision repair 2, TFIIH core complex helicase subunit [Source:HGNC Symbol;Acc:HGNC:3434]	136.7	87.2	**-1.56**	1.12E-08
LIN9	lin-9 DREAM MuvB core complex component [Source:HGNC Symbol;Acc:HGNC:30830]	346.4	227.9	**-1.56**	1.21E-08
UTS2	urotensin 2 [Source:HGNC Symbol;Acc:HGNC:12636]	146.1	93.7	**-1.56**	2.72E-08
C4orf46	chromosome 4 open reading frame 46 [Source:HGNC Symbol;Acc:HGNC:27320]	386.3	253.8	**-1.56**	5.60E-08
ZNF713	zinc finger protein 713 [Source:HGNC Symbol;Acc:HGNC:22043]	153.7	98.3	**-1.56**	2.65E-06
DCAF15	DDB1 and CUL4 associated factor 15 [Source:HGNC Symbol;Acc:HGNC:25095]	138.6	87.9	**-1.56**	6.40E-06
GJC1	gap junction protein gamma 1 [Source:HGNC Symbol;Acc:HGNC:4280]	85.7	54.4	**-1.56**	0.0007465
TSPOAP1	TSPO associated protein 1 [Source:HGNC Symbol;Acc:HGNC:16831]	67.6	42.4	**-1.56**	0.0007507
ACOT11	acyl-CoA thioesterase 11 [Source:HGNC Symbol;Acc:HGNC:18156]	72.9	45.3	**-1.56**	0.004602
AMT	aminomethyltransferase [Source:HGNC Symbol;Acc:HGNC:473]	44.9	27.7	**-1.56**	0.005162
PSMB9	proteasome subunit beta 9 [Source:HGNC Symbol;Acc:HGNC:9546]	27.4	16.8	**-1.56**	0.0261
TTC22	tetratricopeptide repeat domain 22 [Source:HGNC Symbol;Acc:HGNC:26067]	24.2	14.7	**-1.56**	0.02965
MOXD1	monooxygenase DBH like 1 [Source:HGNC Symbol;Acc:HGNC:21063]	5638.2	3596.4	**-1.57**	4.35E-38
HHIPL1	HHIP like 1 [Source:HGNC Symbol;Acc:HGNC:19710]	829.0	527.8	**-1.57**	2.86E-23
ANKRD50	ankyrin repeat domain 50 [Source:HGNC Symbol;Acc:HGNC:29223]	2982.9	1877.2	**-1.57**	7.09E-22
TMPO	thymopoietin [Source:HGNC Symbol;Acc:HGNC:11875]	3170.3	2069.5	**-1.57**	1.30E-19
PKD2	polycystin 2, transient receptor potential cation channel [Source:HGNC Symbol;Acc:HGNC:9009]	3909.9	2462.5	**-1.57**	5.55E-19
SREBF1	sterol regulatory element binding transcription factor 1 [Source:HGNC Symbol;Acc:HGNC:11289]	349.0	221.4	**-1.57**	4.98E-14
RACGAP1	Rac GTPase activating protein 1 [Source:HGNC Symbol;Acc:HGNC:9804]	1439.2	955.4	**-1.57**	5.39E-14

MCM6	minichromosome maintenance complex component 6 [Source:HGNC Symbol;Acc:HGNC:6949]	2095.9	1370.7	**-1.57**	1.33E-12
CD44-AS1	CD44 antisense RNA 1 [Source:HGNC Symbol;Acc:HGNC:40133]	534.1	337.4	**-1.57**	6.51E-11
TGFA	transforming growth factor alpha [Source:HGNC Symbol;Acc:HGNC:11765]	586.3	368.0	**-1.57**	9.06E-11
HIST2H2BF	histone cluster 2 H2B family member f [Source:HGNC Symbol;Acc:HGNC:24700]	202.3	128.2	**-1.57**	1.02E-09
HIST1H4C	histone cluster 1 H4 family member c [Source:HGNC Symbol;Acc:HGNC:4787]	511.2	333.6	**-1.57**	9.27E-09
PTGFR	prostaglandin F receptor [Source:HGNC Symbol;Acc:HGNC:9600]	287.0	182.1	**-1.57**	1.28E-07
MTMR11	myotubularin related protein 11 [Source:HGNC Symbol;Acc:HGNC:24307]	240.5	151.0	**-1.57**	3.00E-07
ZSCAN2	zinc finger and SCAN domain containing 2 [Source:HGNC Symbol;Acc:HGNC:20994]	112.8	70.8	**-1.57**	1.47E-06
PGBD2	piggyBac transposable element derived 2 [Source:HGNC Symbol;Acc:HGNC:19399]	132.5	84.0	**-1.57**	1.88E-06
CLSTN3	calsyntenin 3 [Source:HGNC Symbol;Acc:HGNC:18371]	162.1	102.0	**-1.57**	1.98E-06
RMI1	RecQ mediated genome instability 1 [Source:HGNC Symbol;Acc:HGNC:25764]	90.6	57.2	**-1.57**	9.34E-05
PCDHB13	protocadherin beta 13 [Source:HGNC Symbol;Acc:HGNC:8684]	82.9	51.4	**-1.57**	0.0001593
ZMYND12	zinc finger MYND-type containing 12 [Source:HGNC Symbol;Acc:HGNC:21192]	79.1	50.2	**-1.57**	0.0008901
MDH1B	malate dehydrogenase 1B [Source:HGNC Symbol;Acc:HGNC:17836]	108.3	67.6	**-1.57**	0.002129
PODXL2	podocalyxin like 2 [Source:HGNC Symbol;Acc:HGNC:17936]	53.7	33.1	**-1.57**	0.002983
FAM84A	family with sequence similarity 84 member A [Source:HGNC Symbol;Acc:HGNC:20743]	43.0	27.9	**-1.57**	0.01873
DMD	dystrophin [Source:HGNC Symbol;Acc:HGNC:2928]	48.2	29.0	**-1.57**	0.04389
MTHFD1L	methylenetetrahydrofolate dehydrogenase (NADP+ dependent) 1 like [Source:HGNC Symbol;Acc:HGNC	1892.1	1199.0	**-1.58**	2.55E-56
TMEM45A	transmembrane protein 45A [Source:HGNC Symbol;Acc:HGNC:25480]	3901.1	2444.5	**-1.58**	5.11E-37
CLMAT3	colorectal liver metastasis associated transcript 3 [Source:HGNC Symbol;Acc:HGNC:52287]	1954.4	1234.9	**-1.58**	1.24E-35
GALNT5	polypeptide N-acetylgalactosaminyltransferase 5 [Source:HGNC Symbol;Acc:HGNC:4127]	2508.3	1579.4	**-1.58**	1.59E-32
MRPL17	mitochondrial ribosomal protein L17 [Source:HGNC Symbol;Acc:HGNC:14053]	1800.2	1143.0	**-1.58**	1.14E-28
PAICS	phosphoribosylaminoimidazole carboxylase and phosphoribosylaminoimidazolesuccinocarboxamide syı	2857.9	1831.6	**-1.58**	9.18E-27
SLC2A13	solute carrier family 2 member 13 [Source:HGNC Symbol;Acc:HGNC:15956]	892.0	562.1	**-1.58**	1.08E-22
SEMA4C	semaphorin 4C [Source:HGNC Symbol;Acc:HGNC:10731]	423.9	269.1	**-1.58**	4.17E-18
FBXO5	F-box protein 5 [Source:HGNC Symbol;Acc:HGNC:13584]	460.0	297.9	**-1.58**	1.39E-10
ZFP69B	ZFP69 zinc finger protein B [Source:HGNC Symbol;Acc:HGNC:28053]	148.2	93.0	**-1.58**	2.12E-09
SH3BGR	SH3 domain binding glutamate rich protein [Source:HGNC Symbol;Acc:HGNC:10822]	140.4	87.2	**-1.58**	6.68E-09
MAPK10	mitogen-activated protein kinase 10 [Source:HGNC Symbol;Acc:HGNC:6872]	140.4	87.2	**-1.58**	1.38E-06
TMEM268	transmembrane protein 268 [Source:HGNC Symbol;Acc:HGNC:24513]	105.9	66.1	**-1.58**	8.43E-06
CDC20	cell division cycle 20 [Source:HGNC Symbol;Acc:HGNC:1723]	195.1	129.9	**-1.58**	1.29E-05
PRR11	proline rich 11 [Source:HGNC Symbol;Acc:HGNC:25619]	188.7	124.2	**-1.58**	0.000128
TPI1P1	triosephosphate isomerase 1 pseudogene 1 [Source:HGNC Symbol;Acc:HGNC:35449]	112.3	70.3	**-1.58**	0.0005239
KCNG1	potassium voltage-gated channel modifier subfamily G member 1 [Source:HGNC Symbol;Acc:HGNC:624	50.8	32.0	**-1.58**	0.001324
LIN7B	lin-7 homolog B, crumbs cell polarity complex component [Source:HGNC Symbol;Acc:HGNC:17788]	37.0	22.7	**-1.58**	0.007228
BEND3P3	BEN domain containing 3 pseudogene 3 [Source:HGNC Symbol;Acc:HGNC:45016]	34.8	21.3	**-1.58**	0.01137
HLTF-AS1	HLTF antisense RNA 1 [Source:HGNC Symbol;Acc:HGNC:40554]	27.5	17.0	**-1.58**	0.01342
EPHB6	EPH receptor B6 [Source:HGNC Symbol;Acc:HGNC:3396]	18.2	11.1	**-1.58**	0.03868
PGK1	phosphoglycerate kinase 1 [Source:HGNC Symbol;Acc:HGNC:8896]	12358.2	7736.0	**-1.59**	1.48E-28
FRMD6	FERM domain containing 6 [Source:HGNC Symbol;Acc:HGNC:19839]	8561.6	5509.3	**-1.59**	2.34E-22
PYCR1	pyrroline-5-carboxylate reductase 1 [Source:HGNC Symbol;Acc:HGNC:9721]	917.2	575.7	**-1.59**	3.32E-19
DDX11	DEAD/H-box helicase 11 [Source:HGNC Symbol;Acc:HGNC:2736]	389.7	246.2	**-1.59**	7.60E-16
CDCA4	cell division cycle associated 4 [Source:HGNC Symbol;Acc:HGNC:14625]	436.1	277.7	**-1.59**	5.11E-13
SLC37A2	solute carrier family 37 member 2 [Source:HGNC Symbol;Acc:HGNC:20644]	356.0	224.0	**-1.59**	4.12E-12
GXYLT2	glucoside xylosyltransferase 2 [Source:HGNC Symbol;Acc:HGNC:33383]	291.4	180.7	**-1.59**	7.19E-12
CDCA7L	cell division cycle associated 7 like [Source:HGNC Symbol;Acc:HGNC:30777]	542.1	352.8	**-1.59**	5.55E-09
SELP	selectin P [Source:HGNC Symbol;Acc:HGNC:10721]	194.2	122.6	**-1.59**	7.02E-08
DSCC1	DNA replication and sister chromatid cohesion 1 [Source:HGNC Symbol;Acc:HGNC:24453]	316.5	204.5	**-1.59**	2.27E-07
METRN	meteorin, glial cell differentiation regulator [Source:HGNC Symbol;Acc:HGNC:14151]	125.3	78.6	**-1.59**	4.01E-07
RIMKLA	ribosomal modification protein rimK like family member A [Source:HGNC Symbol;Acc:HGNC:28725]	105.0	66.4	**-1.59**	1.62E-05
RAB15	RAB15, member RAS oncogene family [Source:HGNC Symbol;Acc:HGNC:20150]	164.6	104.7	**-1.59**	1.66E-05
GSTM2	glutathione S-transferase mu 2 [Source:HGNC Symbol;Acc:HGNC:4634]	84.3	52.6	**-1.59**	0.0008943
SLC4A8	solute carrier family 4 member 8 [Source:HGNC Symbol;Acc:HGNC:11034]	63.8	39.9	**-1.59**	0.004059
NAT14	N-acetyltransferase 14 (putative) [Source:HGNC Symbol;Acc:HGNC:28918]	37.7	22.5	**-1.59**	0.009004
MC1R	melanocortin 1 receptor [Source:HGNC Symbol;Acc:HGNC:6929]	30.5	18.4	**-1.59**	0.00955
SH3BP1	SH3 domain binding protein 1 [Source:HGNC Symbol;Acc:HGNC:10824]	56.5	34.4	**-1.59**	0.01047
LINC01096	long intergenic non-protein coding RNA 1096 [Source:HGNC Symbol;Acc:HGNC:27739]	22.2	13.1	**-1.59**	0.03445
GASAL1	growth arrest associated lncRNA 1 [Source:HGNC Symbol;Acc:HGNC:53461]	24.1	14.4	**-1.59**	0.04157
CBR1	carbonyl reductase 1 [Source:HGNC Symbol;Acc:HGNC:1548]	979.2	614.1	**-1.60**	6.22E-30
PTK7	protein tyrosine kinase 7 (inactive) [Source:HGNC Symbol;Acc:HGNC:9618]	641.1	404.2	**-1.60**	1.08E-20
ATRNL1	attractin like 1 [Source:HGNC Symbol;Acc:HGNC:29063]	1142.1	699.0	**-1.60**	2.02E-19
FZD7	frizzled class receptor 7 [Source:HGNC Symbol;Acc:HGNC:4045]	287.5	178.7	**-1.60**	3.08E-11
FAM49A	family with sequence similarity 49 member A [Source:HGNC Symbol;Acc:HGNC:25373]	354.7	229.1	**-1.60**	1.11E-10
CIT	citron rho-interacting serine/threonine kinase [Source:HGNC Symbol;Acc:HGNC:1985]	244.9	153.0	**-1.60**	2.14E-09
DLGAP1-AS1	DLGAP1 antisense RNA 1 [Source:HGNC Symbol;Acc:HGNC:31676]	123.9	76.1	**-1.60**	6.96E-06
PLEKHH3	pleckstrin homology, MyTH4 and FERM domain containing H3 [Source:HGNC Symbol;Acc:HGNC:26105]	97.7	59.9	**-1.60**	2.08E-05
LMF1	lipase maturation factor 1 [Source:HGNC Symbol;Acc:HGNC:14154]	122.0	76.0	**-1.60**	3.54E-05
CDK5	cyclin dependent kinase 5 [Source:HGNC Symbol;Acc:HGNC:1774]	91.0	55.9	**-1.60**	0.0001314
TMEM144	transmembrane protein 144 [Source:HGNC Symbol;Acc:HGNC:25633]	130.8	81.4	**-1.60**	0.0001327
CORO2B	coronin 2B [Source:HGNC Symbol;Acc:HGNC:2256]	93.7	59.0	**-1.60**	0.001145
SPNS2	sphingolipid transporter 2 [Source:HGNC Symbol;Acc:HGNC:26992]	66.1	38.7	**-1.60**	0.003301
PHEX	phosphate regulating endopeptidase homolog X-linked [Source:HGNC Symbol;Acc:HGNC:8918]	51.3	30.3	**-1.60**	0.008954

C4A	complement C4A (Rodgers blood group) [Source:HGNC Symbol;Acc:HGNC:1323]	33.0	20.3	**-1.60**	0.009521
HIVEP3	human immunodeficiency virus type I enhancer binding protein 3 [Source:HGNC Symbol;Acc:HGNC:135	42.0	25.6	**-1.60**	0.009644
NCAM2	neural cell adhesion molecule 2 [Source:HGNC Symbol;Acc:HGNC:7657]	56.2	31.2	**-1.60**	0.02244
APOBEC3D	apolipoprotein B mRNA editing enzyme catalytic subunit 3D [Source:HGNC Symbol;Acc:HGNC:17354]	28.3	17.0	**-1.60**	0.02294
FAR2P2	fatty acyl-CoA reductase 2 pseudogene 2 [Source:HGNC Symbol;Acc:HGNC:49279]	42.1	25.1	**-1.60**	0.02396
GLP2R	glucagon like peptide 2 receptor [Source:HGNC Symbol;Acc:HGNC:4325]	40.5	23.7	**-1.60**	0.04668
PRKG1	protein kinase cGMP-dependent 1 [Source:HGNC Symbol;Acc:HGNC:9414]	813.2	494.7	**-1.61**	2.46E-19
PBX1	PBX homeobox 1 [Source:HGNC Symbol;Acc:HGNC:8632]	728.0	454.8	**-1.61**	4.77E-19
TNFRSF10D	TNF receptor superfamily member 10d [Source:HGNC Symbol;Acc:HGNC:11907]	3385.8	2010.0	**-1.61**	1.97E-17
PITX1	paired like homeodomain 1 [Source:HGNC Symbol;Acc:HGNC:9004]	317.8	192.1	**-1.61**	8.45E-11
AMOT	angiomotin [Source:HGNC Symbol;Acc:HGNC:17810]	274.9	169.0	**-1.61**	1.43E-10
WDR76	WD repeat domain 76 [Source:HGNC Symbol;Acc:HGNC:25773]	963.8	628.2	**-1.61**	3.66E-10
DMRTA1	DMRT like family A1 [Source:HGNC Symbol;Acc:HGNC:13826]	362.8	219.9	**-1.61**	4.91E-09
FEN1	flap structure-specific endonuclease 1 [Source:HGNC Symbol;Acc:HGNC:3650]	456.4	287.8	**-1.61**	1.15E-08
PARPBP	PARP1 binding protein [Source:HGNC Symbol;Acc:HGNC:26074]	199.7	125.6	**-1.61**	9.45E-08
NCAPH	non-SMC condensin I complex subunit H [Source:HGNC Symbol;Acc:HGNC:1112]	289.0	188.1	**-1.61**	7.87E-07
ESPL1	extra spindle pole bodies like 1, separase [Source:HGNC Symbol;Acc:HGNC:16856]	119.2	73.2	**-1.61**	1.27E-06
NLGN4X	neuroligin 4 X-linked [Source:HGNC Symbol;Acc:HGNC:14287]	129.9	78.6	**-1.61**	1.26E-05
FAM171A1	family with sequence similarity 171 member A1 [Source:HGNC Symbol;Acc:HGNC:23522]	84.5	51.2	**-1.61**	1.52E-05
TPRG1	tumor protein p63 regulated 1 [Source:HGNC Symbol;Acc:HGNC:24759]	126.1	74.7	**-1.61**	3.81E-05
LINC00471	long intergenic non-protein coding RNA 471 [Source:HGNC Symbol;Acc:HGNC:28668]	52.5	32.0	**-1.61**	0.0009031
NPTX2	neuronal pentraxin 2 [Source:HGNC Symbol;Acc:HGNC:7953]	55.8	34.4	**-1.61**	0.002176
CAPN6	calpain 6 [Source:HGNC Symbol;Acc:HGNC:1483]	76.4	44.8	**-1.61**	0.003849
PCDH10	protocadherin 10 [Source:HGNC Symbol;Acc:HGNC:13404]	46.0	26.6	**-1.61**	0.008703
TMEM143	transmembrane protein 143 [Source:HGNC Symbol;Acc:HGNC:25603]	31.1	18.3	**-1.61**	0.01107
INAFM2	InaF motif containing 2 [Source:HGNC Symbol;Acc:HGNC:35165]	38.0	22.9	**-1.61**	0.01137
PLCE1-AS1	PLCE1 antisense RNA 1 [Source:HGNC Symbol;Acc:HGNC:45193]	34.7	20.7	**-1.61**	0.01466
SPIRE2	spire type actin nucleation factor 2 [Source:HGNC Symbol;Acc:HGNC:30623]	21.9	12.6	**-1.61**	0.02366
ARHGAP25	Rho GTPase activating protein 25 [Source:HGNC Symbol;Acc:HGNC:28951]	18.4	10.8	**-1.61**	0.02719
NFATC4	nuclear factor of activated T cells 4 [Source:HGNC Symbol;Acc:HGNC:7778]	22.2	13.2	**-1.61**	0.02753
ID2-AS1	ID2 antisense RNA 1 [Source:HGNC Symbol;Acc:HGNC:51103]	19.9	11.7	**-1.61**	0.04208
SNX18	sorting nexin 18 [Source:HGNC Symbol;Acc:HGNC:19245]	1512.9	931.9	**-1.62**	1.62E-52
C9orf3	chromosome 9 open reading frame 3 [Source:HGNC Symbol;Acc:HGNC:1361]	3363.6	2059.0	**-1.62**	1.20E-32
ADIRF-AS1	ADIRF antisense RNA 1 [Source:HGNC Symbol;Acc:HGNC:45127]	616.1	377.7	**-1.62**	4.89E-27
STARD4	StAR related lipid transfer domain containing 4 [Source:HGNC Symbol;Acc:HGNC:18058]	1389.8	867.2	**-1.62**	8.62E-26
STMN1	stathmin 1 [Source:HGNC Symbol;Acc:HGNC:6510]	1472.8	974.7	**-1.62**	1.85E-17
VWA5A	von Willebrand factor A domain containing 5A [Source:HGNC Symbol;Acc:HGNC:6658]	522.5	324.6	**-1.62**	1.27E-15
MCM8	minichromosome maintenance 8 homologous recombination repair factor [Source:HGNC Symbol;Acc:H	769.0	486.6	**-1.62**	8.11E-11
RNASET2	ribonuclease T2 [Source:HGNC Symbol;Acc:HGNC:21686]	229.4	141.5	**-1.62**	5.25E-10
AL358075.4	-PIK3R3 readthrough [Source:NCBI gene;Acc:110117499]	258.6	155.4	**-1.62**	6.43E-09
CCDC3	coiled-coil domain containing 3 [Source:HGNC Symbol;Acc:HGNC:23813]	187.8	111.9	**-1.62**	1.83E-07
HECW1	HECT, C2 and WW domain containing E3 ubiquitin protein ligase 1 [Source:HGNC Symbol;Acc:HGNC:221	189.8	116.6	**-1.62**	7.01E-07
PRKAR1B	protein kinase cAMP-dependent type I regulatory subunit beta [Source:HGNC Symbol;Acc:HGNC:9390]	122.6	74.7	**-1.62**	1.23E-06
AKNA	AT-hook transcription factor [Source:HGNC Symbol;Acc:HGNC:24108]	158.7	94.6	**-1.62**	1.71E-06
AFAP1-AS1	AFAP1 antisense RNA 1 [Source:HGNC Symbol;Acc:HGNC:28141]	107.6	64.9	**-1.62**	4.37E-05
KIF26A	kinesin family member 26A [Source:HGNC Symbol;Acc:HGNC:20226]	66.1	40.7	**-1.62**	0.0003689
KIAA1522	KIAA1522 [Source:HGNC Symbol;Acc:HGNC:29301]	31.4	18.4	**-1.62**	0.006089
ABCB1	ATP binding cassette subfamily B member 1 [Source:HGNC Symbol;Acc:HGNC:40]	39.0	23.2	**-1.62**	0.01334
ERICH2	glutamate rich 2 [Source:HGNC Symbol;Acc:HGNC:44395]	31.2	17.9	**-1.62**	0.01625
TP53I11	tumor protein p53 inducible protein 11 [Source:HGNC Symbol;Acc:HGNC:16842]	33.1	19.5	**-1.62**	0.02225
AQP1	aquaporin 1 (Colton blood group) [Source:HGNC Symbol;Acc:HGNC:633]	42.1	27.5	**-1.62**	0.02304
TNFRSF12A	TNF receptor superfamily member 12A [Source:HGNC Symbol;Acc:HGNC:18152]	2411.4	1456.0	**-1.64**	1.97E-34
RCC2	regulator of chromosome condensation 2 [Source:HGNC Symbol;Acc:HGNC:30297]	859.8	523.9	**-1.64**	4.94E-30
ANTXR1	anthrax toxin receptor 1 [Source:HGNC Symbol;Acc:HGNC:21014]	1648.2	1010.9	**-1.64**	4.21E-27
PPP2R3A	protein phosphatase 2 regulatory subunit B''alpha [Source:HGNC Symbol;Acc:HGNC:9307]	1263.3	758.0	**-1.64**	1.42E-21
LGALS1	galectin 1 [Source:HGNC Symbol;Acc:HGNC:6561]	2948.5	1851.9	**-1.64**	1.64E-21
PDGFRL	platelet derived growth factor receptor like [Source:HGNC Symbol;Acc:HGNC:8805]	480.4	291.9	**-1.64**	5.92E-14
TEAD2	TEA domain transcription factor 2 [Source:HGNC Symbol;Acc:HGNC:11715]	171.1	104.1	**-1.64**	2.18E-11
THBS2	thrombospondin 2 [Source:HGNC Symbol;Acc:HGNC:11786]	1473.6	935.9	**-1.64**	2.46E-11
GSDME	gasdermin E [Source:HGNC Symbol;Acc:HGNC:2810]	322.8	195.8	**-1.64**	1.18E-09
SHISA4	shisa family member 4 [Source:HGNC Symbol;Acc:HGNC:27139]	367.7	221.3	**-1.64**	1.67E-09
COL11A2	collagen type XI alpha 2 chain [Source:HGNC Symbol;Acc:HGNC:2187]	15200.0	9574.8	**-1.64**	3.13E-09
CDNF	cerebral dopamine neurotrophic factor [Source:HGNC Symbol;Acc:HGNC:24913]	125.4	75.5	**-1.64**	2.82E-08
NOL4L	nucleolar protein 4 like [Source:HGNC Symbol;Acc:HGNC:16106]	172.9	103.9	**-1.64**	8.92E-07
PLEKHA2	pleckstrin homology domain containing A2 [Source:HGNC Symbol;Acc:HGNC:14336]	188.7	115.8	**-1.64**	4.03E-06
EVI2A	ecotropic viral integration site 2A [Source:HGNC Symbol;Acc:HGNC:3499]	1961.4	1181.3	**-1.64**	4.08E-06
NID1	nidogen 1 [Source:HGNC Symbol;Acc:HGNC:7821]	91.6	56.0	**-1.64**	2.30E-05
CACNB1	calcium voltage-gated channel auxiliary subunit beta 1 [Source:HGNC Symbol;Acc:HGNC:1401]	97.5	58.0	**-1.64**	4.64E-05
SPRED3	sprouty related EVH1 domain containing 3 [Source:HGNC Symbol;Acc:HGNC:31041]	65.3	38.0	**-1.64**	0.0008249
MANEAL	mannosidase endo-alpha like [Source:HGNC Symbol;Acc:HGNC:26452]	54.8	32.6	**-1.64**	0.001352
OIP5	Opa interacting protein 5 [Source:HGNC Symbol;Acc:HGNC:20300]	57.1	35.4	**-1.64**	0.002969
TMEM169	transmembrane protein 169 [Source:HGNC Symbol;Acc:HGNC:25130]	45.1	25.5	**-1.64**	0.004753

USP18	ubiquitin specific peptidase 18 [Source:HGNC Symbol;Acc:HGNC:12616]	31.7	18.7	**-1.64**	0.01007
COL19A1	collagen type XIX alpha 1 chain [Source:HGNC Symbol;Acc:HGNC:2196]	39.6	22.7	**-1.64**	0.01962
ROPN1B	rhophilin associated tail protein 1B [Source:HGNC Symbol;Acc:HGNC:31927]	28.4	16.0	**-1.64**	0.03174
EPOR	erythropoietin receptor [Source:HGNC Symbol;Acc:HGNC:3416]	24.6	13.9	**-1.64**	0.03307
TRIM61	tripartite motif containing 61 [Source:HGNC Symbol;Acc:HGNC:24339]	23.1	13.3	**-1.64**	0.04194
MIR100HG	mir-100-let-7a-2-mir-125b-1 cluster host gene [Source:HGNC Symbol;Acc:HGNC:39522]	7031.0	4292.0	**-1.65**	1.46E-45
SKA2	spindle and kinetochore associated complex subunit 2 [Source:HGNC Symbol;Acc:HGNC:28006]	1580.0	965.6	**-1.65**	5.54E-33
TMTC1	transmembrane and tetratricopeptide repeat containing 1 [Source:HGNC Symbol;Acc:HGNC:24099]	9111.1	5478.4	**-1.65**	4.45E-31
SMO	smoothened, frizzled class receptor [Source:HGNC Symbol;Acc:HGNC:11119]	380.4	231.4	**-1.65**	2.44E-20
RTKN	rhotekin [Source:HGNC Symbol;Acc:HGNC:10466]	464.2	283.3	**-1.65**	8.27E-17
LYNX1	Ly6/neurotoxin 1 [Source:HGNC Symbol;Acc:HGNC:29604]	896.2	547.8	**-1.65**	2.65E-15
CIP2A	cell proliferation regulating inhibitor of protein phosphatase 2A [Source:HGNC Symbol;Acc:HGNC:29302	597.9	363.6	**-1.65**	1.58E-13
SPATA20	spermatogenesis associated 20 [Source:HGNC Symbol;Acc:HGNC:26125]	332.4	204.2	**-1.65**	3.95E-12
MCM5	minichromosome maintenance complex component 5 [Source:HGNC Symbol;Acc:HGNC:6948]	991.3	614.2	**-1.65**	1.74E-10
CCDC85B	coiled-coil domain containing 85B [Source:HGNC Symbol;Acc:HGNC:24926]	366.4	216.3	**-1.65**	2.70E-10
CYGB	cytoglobin [Source:HGNC Symbol;Acc:HGNC:16505]	206.1	124.2	**-1.65**	1.24E-08
HIST1H4B	histone cluster 1 H4 family member b [Source:HGNC Symbol;Acc:HGNC:4789]	286.4	174.4	**-1.65**	1.37E-07
DPY19L2P1	DPY19L2 pseudogene 1 [Source:HGNC Symbol;Acc:HGNC:22305]	111.0	67.5	**-1.65**	9.42E-07
CCDC28B	coiled-coil domain containing 28B [Source:HGNC Symbol;Acc:HGNC:28163]	75.6	44.9	**-1.65**	2.90E-05
MSH5-SAPCD1	MSH5-SAPCD1 readthrough (NMD candidate) [Source:HGNC Symbol;Acc:HGNC:41994]	50.8	30.3	**-1.65**	0.0003984
SSTR5-AS1	SSTR5 antisense RNA 1 [Source:HGNC Symbol;Acc:HGNC:26502]	108.3	65.2	**-1.65**	0.000426
ANGPT2	angiopoietin 2 [Source:HGNC Symbol;Acc:HGNC:485]	213.3	129.8	**-1.65**	0.001045
TBX19	T-box 19 [Source:HGNC Symbol;Acc:HGNC:11596]	39.3	22.6	**-1.65**	0.02385
JAG1	jagged 1 [Source:HGNC Symbol;Acc:HGNC:6188]	4398.5	2631.8	**-1.66**	1.06E-31
PDK1	pyruvate dehydrogenase kinase 1 [Source:HGNC Symbol;Acc:HGNC:8809]	1538.9	925.8	**-1.66**	1.59E-31
FHL2	four and a half LIM domains 2 [Source:HGNC Symbol;Acc:HGNC:3703]	1583.1	959.4	**-1.66**	4.38E-31
TPI1	triosephosphate isomerase 1 [Source:HGNC Symbol;Acc:HGNC:12009]	9017.1	5450.2	**-1.66**	2.37E-28
ADIRF	adipogenesis regulatory factor [Source:HGNC Symbol;Acc:HGNC:24043]	580.5	348.3	**-1.66**	3.84E-28
SYNC	syncoilin, intermediate filament protein [Source:HGNC Symbol;Acc:HGNC:28897]	697.5	413.1	**-1.66**	1.36E-20
SH2B3	SH2B adaptor protein 3 [Source:HGNC Symbol;Acc:HGNC:29605]	693.7	417.6	**-1.66**	2.27E-17
ROR2	receptor tyrosine kinase like orphan receptor 2 [Source:HGNC Symbol;Acc:HGNC:10257]	827.1	492.0	**-1.66**	2.46E-17
LRIG1	leucine rich repeats and immunoglobulin like domains 1 [Source:HGNC Symbol;Acc:HGNC:17360]	832.3	501.9	**-1.66**	7.24E-17
S100A2	S100 calcium binding protein A2 [Source:HGNC Symbol;Acc:HGNC:10492]	357.3	216.4	**-1.66**	3.52E-15
HIVEP1	human immunodeficiency virus type I enhancer binding protein 1 [Source:HGNC Symbol;Acc:HGNC:492(	505.0	297.0	**-1.66**	1.82E-14
NCS1	neuronal calcium sensor 1 [Source:HGNC Symbol;Acc:HGNC:3953]	302.7	182.1	**-1.66**	6.34E-14
FIGNL1	fidgetin like 1 [Source:HGNC Symbol;Acc:HGNC:13286]	1122.9	694.6	**-1.66**	3.67E-13
XXYLT1	xyloside xylosyltransferase 1 [Source:HGNC Symbol;Acc:HGNC:26639]	280.1	167.5	**-1.66**	4.02E-13
CKS1B	CDC28 protein kinase regulatory subunit 1B [Source:HGNC Symbol;Acc:HGNC:19083]	344.4	208.7	**-1.66**	6.39E-13
DNAJC6	DnaJ heat shock protein family (Hsp40) member C6 [Source:HGNC Symbol;Acc:HGNC:15469]	220.4	131.5	**-1.66**	5.26E-11
SCARB1	scavenger receptor class B member 1 [Source:HGNC Symbol;Acc:HGNC:1664]	231.7	138.5	**-1.66**	3.06E-10
ATAD5	ATPase family, AAA domain containing 5 [Source:HGNC Symbol;Acc:HGNC:25752]	307.0	190.7	**-1.66**	7.68E-10
ISOC2	isochorismatase domain containing 2 [Source:HGNC Symbol;Acc:HGNC:26278]	131.9	79.3	**-1.66**	1.43E-06
FLNB-AS1	FLNB antisense RNA 1 [Source:HGNC Symbol;Acc:HGNC:40239]	80.7	48.6	**-1.66**	6.03E-05
LURAP1L	leucine rich adaptor protein 1 like [Source:HGNC Symbol;Acc:HGNC:31452]	111.7	66.5	**-1.66**	0.0002428
SLC27A3	solute carrier family 27 member 3 [Source:HGNC Symbol;Acc:HGNC:10997]	63.6	37.4	**-1.66**	0.0002692
MTFR2	mitochondrial fission regulator 2 [Source:HGNC Symbol;Acc:HGNC:21115]	61.9	36.2	**-1.66**	0.0004589
CREB3L1	cAMP responsive element binding protein 3 like 1 [Source:HGNC Symbol;Acc:HGNC:18856]	42.4	24.9	**-1.66**	0.0006782
MAGIX	MAGI family member, X-linked [Source:HGNC Symbol;Acc:HGNC:30006]	55.2	32.2	**-1.66**	0.001698
TMCC1-AS1	TMCC1 antisense RNA 1 (head to head) [Source:HGNC Symbol;Acc:HGNC:49060]	44.3	25.9	**-1.66**	0.002264
DLG1-AS1	DLG1 antisense RNA 1 [Source:HGNC Symbol;Acc:HGNC:44154]	28.3	16.3	**-1.66**	0.005676
C4B	complement C4B (Chido blood group) [Source:HGNC Symbol;Acc:HGNC:1324]	22.9	13.5	**-1.66**	0.02963
EGR2	early growth response 2 [Source:HGNC Symbol;Acc:HGNC:3239]	39.4	22.0	**-1.66**	0.04085
LOXL3	lysyl oxidase like 3 [Source:HGNC Symbol;Acc:HGNC:13869]	10236.0	6124.7	**-1.67**	1.30E-56
SPARC	secreted protein acidic and cysteine rich [Source:HGNC Symbol;Acc:HGNC:11219]	37978.8	22842.5	**-1.67**	5.25E-47
FGFR1	fibroblast growth factor receptor 1 [Source:HGNC Symbol;Acc:HGNC:3688]	6894.2	4115.2	**-1.67**	1.36E-43
TUBB2A	tubulin beta 2A class IIa [Source:HGNC Symbol;Acc:HGNC:12412]	702.6	417.3	**-1.67**	1.20E-14
SPATA18	spermatogenesis associated 18 [Source:HGNC Symbol;Acc:HGNC:29579]	221.3	130.5	**-1.67**	5.43E-12
DPY19L2P2	DPY19L2 pseudogene 2 [Source:HGNC Symbol;Acc:HGNC:21764]	197.2	119.2	**-1.67**	6.57E-09
DM1-AS	DM1 locus antisense RNA [Source:HGNC Symbol;Acc:HGNC:53125]	114.7	68.9	**-1.67**	8.43E-08
UBE2C	ubiquitin conjugating enzyme E2 C [Source:HGNC Symbol;Acc:HGNC:15937]	181.0	112.8	**-1.67**	1.73E-07
SPAG8	sperm associated antigen 8 [Source:HGNC Symbol;Acc:HGNC:14105]	101.9	59.2	**-1.67**	2.87E-07
THBS1	thrombospondin 1 [Source:HGNC Symbol;Acc:HGNC:11785]	42257.5	25654.6	**-1.67**	3.47E-07
RNF152	ring finger protein 152 [Source:HGNC Symbol;Acc:HGNC:26811]	1462.5	877.6	**-1.67**	7.67E-06
PLEKHA8P1	pleckstrin homology domain containing A8 pseudogene 1 [Source:HGNC Symbol;Acc:HGNC:30222]	48.2	28.2	**-1.67**	0.0006172
HIST1H3I	histone cluster 1 H3 family member i [Source:HGNC Symbol;Acc:HGNC:4771]	63.2	39.7	**-1.67**	0.00112
EPYC	epiphycan [Source:HGNC Symbol;Acc:HGNC:3053]	46.3	27.1	**-1.67**	0.002131
GUCY1B1	guanylate cyclase 1 soluble subunit beta 1 [Source:HGNC Symbol;Acc:HGNC:4687]	53.5	32.1	**-1.67**	0.002631
GRAMD2A	GRAM domain containing 2A [Source:HGNC Symbol;Acc:HGNC:27287]	45.4	25.8	**-1.67**	0.00334
SLC15A2	solute carrier family 15 member 2 [Source:HGNC Symbol;Acc:HGNC:10921]	36.8	20.8	**-1.67**	0.006264
TTYH3	tweety family member 3 [Source:HGNC Symbol;Acc:HGNC:22222]	26.4	15.0	**-1.67**	0.0196
LINC02256	long intergenic non-protein coding RNA 2256 [Source:HGNC Symbol;Acc:HGNC:53157]	21.7	12.2	**-1.67**	0.02306
SLC10A5	solute carrier family 10 member 5 [Source:HGNC Symbol;Acc:HGNC:22981]	20.6	11.4	**-1.67**	0.04255

NCAPG2	non-SMC condensin II complex subunit G2 [Source:HGNC Symbol;Acc:HGNC:21904]	962.7	574.7	**-1.68**	5.72E-32
CHST6	carbohydrate sulfotransferase 6 [Source:HGNC Symbol;Acc:HGNC:6938]	1257.8	743.0	**-1.68**	2.59E-27
ANOS1	anosmin 1 [Source:HGNC Symbol;Acc:HGNC:6211]	903.6	530.6	**-1.68**	5.80E-23
MYO1B	myosin IB [Source:HGNC Symbol;Acc:HGNC:7596]	3760.8	2266.3	**-1.68**	1.92E-18
MSH2	mutS homolog 2 [Source:HGNC Symbol;Acc:HGNC:7325]	1446.7	860.8	**-1.68**	2.54E-18
EHD3	EH domain containing 3 [Source:HGNC Symbol;Acc:HGNC:3244]	606.6	356.4	**-1.68**	7.28E-18
FZD4	frizzled class receptor 4 [Source:HGNC Symbol;Acc:HGNC:4042]	787.1	471.3	**-1.68**	8.06E-15
PALM2	paralemmin 2 [Source:HGNC Symbol;Acc:HGNC:15845]	264.3	155.2	**-1.68**	9.00E-14
CENPN	centromere protein N [Source:HGNC Symbol;Acc:HGNC:30873]	401.0	244.3	**-1.68**	3.53E-13
KNL1	kinetochore scaffold 1 [Source:HGNC Symbol;Acc:HGNC:24054]	1064.0	686.0	**-1.68**	4.64E-12
CDCA2	cell division cycle associated 2 [Source:HGNC Symbol;Acc:HGNC:14623]	311.9	193.0	**-1.68**	1.04E-11
GDF10	growth differentiation factor 10 [Source:HGNC Symbol;Acc:HGNC:4215]	738.5	453.3	**-1.68**	1.54E-10
KIF14	kinesin family member 14 [Source:HGNC Symbol;Acc:HGNC:19181]	524.9	336.0	**-1.68**	2.84E-10
LBH	limb bud and heart development [Source:HGNC Symbol;Acc:HGNC:29532]	2819.9	1684.0	**-1.68**	2.13E-08
HILPDA	hypoxia inducible lipid droplet associated [Source:HGNC Symbol;Acc:HGNC:28859]	158.4	94.6	**-1.68**	5.29E-08
SCN8A	sodium voltage-gated channel alpha subunit 8 [Source:HGNC Symbol;Acc:HGNC:10596]	191.8	112.4	**-1.68**	1.21E-07
ZNF774	zinc finger protein 774 [Source:HGNC Symbol;Acc:HGNC:33108]	88.0	51.8	**-1.68**	2.19E-06
SPIN4	spindlin family member 4 [Source:HGNC Symbol;Acc:HGNC:27040]	122.2	74.5	**-1.68**	1.09E-05
RASSF5	Ras association domain family member 5 [Source:HGNC Symbol;Acc:HGNC:17609]	61.4	36.8	**-1.68**	0.0002615
LINC01588	long intergenic non-protein coding RNA 1588 [Source:HGNC Symbol;Acc:HGNC:27503]	26.4	14.9	**-1.68**	0.007728
CNIH3	cornichon family AMPA receptor auxiliary protein 3 [Source:HGNC Symbol;Acc:HGNC:26802]	36.9	21.5	**-1.68**	0.008511
DPY19L2P3	DPY19L2 pseudogene 3 [Source:HGNC Symbol;Acc:HGNC:22367]	22.2	12.7	**-1.68**	0.01231
LIPT2	lipoyl(octanoyl) transferase 2 [Source:HGNC Symbol;Acc:HGNC:37216]	19.6	10.9	**-1.68**	0.01902
PAMR1	peptidase domain containing associated with muscle regeneration 1 [Source:HGNC Symbol;Acc:HGNC:2	20.7	12.1	**-1.68**	0.03507
TMEM106C	transmembrane protein 106C [Source:HGNC Symbol;Acc:HGNC:28775]	1416.8	843.4	**-1.69**	6.16E-46
TRUB1	TruB pseudouridine synthase family member 1 [Source:HGNC Symbol;Acc:HGNC:16060]	1656.5	977.3	**-1.69**	1.65E-41
GPRASP2	G protein-coupled receptor associated sorting protein 2 [Source:HGNC Symbol;Acc:HGNC:25169]	748.8	444.2	**-1.69**	1.98E-37
WWP2	WW domain containing E3 ubiquitin protein ligase 2 [Source:HGNC Symbol;Acc:HGNC:16804]	5497.8	3210.7	**-1.69**	9.75E-30
FXYD6	FXYD domain containing ion transport regulator 6 [Source:HGNC Symbol;Acc:HGNC:4030]	813.4	484.1	**-1.69**	1.08E-29
PER3	period circadian regulator 3 [Source:HGNC Symbol;Acc:HGNC:8847]	1066.2	623.5	**-1.69**	3.60E-24
ARHGAP11A	Rho GTPase activating protein 11A [Source:HGNC Symbol;Acc:HGNC:15783]	1392.0	859.1	**-1.69**	2.44E-20
MRPS6	mitochondrial ribosomal protein S6 [Source:HGNC Symbol;Acc:HGNC:14051]	6191.9	3546.3	**-1.69**	3.81E-19
COTL1	coactosin like F-actin binding protein 1 [Source:HGNC Symbol;Acc:HGNC:18304]	600.5	350.1	**-1.69**	7.24E-19
AFAP1	actin filament associated protein 1 [Source:HGNC Symbol;Acc:HGNC:24017]	679.1	396.1	**-1.69**	8.27E-19
SCHIP1	schwannomin interacting protein 1 [Source:HGNC Symbol;Acc:HGNC:15678]	418.5	247.9	**-1.69**	1.27E-16
DIAPH3	diaphanous related formin 3 [Source:HGNC Symbol;Acc:HGNC:15480]	662.5	416.2	**-1.69**	5.79E-11
KIF4A	kinesin family member 4A [Source:HGNC Symbol;Acc:HGNC:13339]	433.4	271.9	**-1.69**	7.38E-11
CHCHD6	coiled-coil-helix-coiled-coil-helix domain containing 6 [Source:HGNC Symbol;Acc:HGNC:28184]	113.5	67.0	**-1.69**	1.13E-08
RARA	retinoic acid receptor alpha [Source:HGNC Symbol;Acc:HGNC:9864]	158.3	93.6	**-1.69**	2.31E-08
FOXRED2	FAD dependent oxidoreductase domain containing 2 [Source:HGNC Symbol;Acc:HGNC:26264]	103.9	60.6	**-1.69**	7.49E-08
IL4R	interleukin 4 receptor [Source:HGNC Symbol;Acc:HGNC:6015]	84.2	48.8	**-1.69**	5.62E-06
DGKI	diacylglycerol kinase iota [Source:HGNC Symbol;Acc:HGNC:2855]	105.7	59.7	**-1.69**	1.37E-05
PHLDA2	pleckstrin homology like domain family A member 2 [Source:HGNC Symbol;Acc:HGNC:12385]	78.7	46.5	**-1.69**	2.51E-05
TONSL	tonsoku like, DNA repair protein [Source:HGNC Symbol;Acc:HGNC:7801]	74.9	44.2	**-1.69**	3.29E-05
PNMA8B	PNMA family member 8B [Source:HGNC Symbol;Acc:HGNC:29206]	66.6	38.8	**-1.69**	4.99E-05
SFN	stratifin [Source:HGNC Symbol;Acc:HGNC:10773]	84.1	49.6	**-1.69**	0.0001194
ZNF74	zinc finger protein 74 [Source:HGNC Symbol;Acc:HGNC:13144]	78.6	45.8	**-1.69**	0.0001718
SLC25A10	solute carrier family 25 member 10 [Source:HGNC Symbol;Acc:HGNC:10980]	36.6	20.8	**-1.69**	0.005311
GPR146	G protein-coupled receptor 146 [Source:HGNC Symbol;Acc:HGNC:21718]	22.0	12.4	**-1.69**	0.021
EFNA3	ephrin A3 [Source:HGNC Symbol;Acc:HGNC:3223]	19.4	10.8	**-1.69**	0.02638
SMKR1	small lysine rich protein 1 [Source:HGNC Symbol;Acc:HGNC:43561]	15.2	8.3	**-1.69**	0.04974
CD44	CD44 molecule (Indian blood group) [Source:HGNC Symbol;Acc:HGNC:1681]	10254.5	6019.0	**-1.71**	6.10E-42
SMC2	structural maintenance of chromosomes 2 [Source:HGNC Symbol;Acc:HGNC:14011]	2420.2	1466.4	**-1.71**	5.39E-24
PWAR5	Prader Willi/Angelman region RNA 5 [Source:HGNC Symbol;Acc:HGNC:30090]	470.0	274.3	**-1.71**	1.92E-18
FXYD6-FXYD2	FXYD6-FXYD2 readthrough [Source:HGNC Symbol;Acc:HGNC:39978]	325.7	192.4	**-1.71**	1.86E-17
PCNA	proliferating cell nuclear antigen [Source:HGNC Symbol;Acc:HGNC:8729]	2971.3	1796.8	**-1.71**	4.24E-16
PIK3R3	phosphoinositide-3-kinase regulatory subunit 3 [Source:HGNC Symbol;Acc:HGNC:8981]	438.0	250.7	**-1.71**	6.81E-13
FAM102B	family with sequence similarity 102 member B [Source:HGNC Symbol;Acc:HGNC:27637]	560.7	329.7	**-1.71**	1.06E-12
EPHX2	epoxide hydrolase 2 [Source:HGNC Symbol;Acc:HGNC:3402]	260.4	152.9	**-1.71**	8.49E-12
NME4	NME/NM23 nucleoside diphosphate kinase 4 [Source:HGNC Symbol;Acc:HGNC:7852]	252.7	148.6	**-1.71**	5.29E-11
IFFO2	intermediate filament family orphan 2 [Source:HGNC Symbol;Acc:HGNC:27006]	186.4	109.0	**-1.71**	5.36E-11
ITPR1	inositol 1,4,5-trisphosphate receptor type 1 [Source:HGNC Symbol;Acc:HGNC:6180]	653.1	373.9	**-1.71**	3.47E-09
TRAF4	TNF receptor associated factor 4 [Source:HGNC Symbol;Acc:HGNC:12034]	215.4	126.3	**-1.71**	5.43E-09
FAT4	FAT atypical cadherin 4 [Source:HGNC Symbol;Acc:HGNC:23109]	1831.1	1046.6	**-1.71**	1.27E-08
NIPAL1	NIPA like domain containing 1 [Source:HGNC Symbol;Acc:HGNC:27194]	165.3	97.6	**-1.71**	3.36E-07
CEP55	centrosomal protein 55 [Source:HGNC Symbol;Acc:HGNC:1161]	815.6	515.8	**-1.71**	2.65E-05
CABLES2	Cdk5 and Abl enzyme substrate 2 [Source:HGNC Symbol;Acc:HGNC:16143]	72.1	41.5	**-1.71**	2.90E-05
TMEM201	transmembrane protein 201 [Source:HGNC Symbol;Acc:HGNC:33719]	64.5	37.6	**-1.71**	3.55E-05
CDC25C	cell division cycle 25C [Source:HGNC Symbol;Acc:HGNC:1727]	65.4	38.4	**-1.71**	9.74E-05
PRDM6	PR/SET domain 6 [Source:HGNC Symbol;Acc:HGNC:9350]	78.4	45.0	**-1.71**	0.000191
MSH5	mutS homolog 5 [Source:HGNC Symbol;Acc:HGNC:7328]	52.4	30.4	**-1.71**	0.0002693
SCAMP5	secretory carrier membrane protein 5 [Source:HGNC Symbol;Acc:HGNC:30386]	26.1	14.4	**-1.71**	0.009573

LINC01465	long intergenic non-protein coding RNA 1465 [Source:HGNC Symbol;Acc:HGNC:26364]	28.9	15.7	**-1.71**	0.01514
KNDC1	kinase non-catalytic C-lobe domain containing 1 [Source:HGNC Symbol;Acc:HGNC:29374]	17.4	9.9	**-1.71**	0.03119
HHIPL2	HHIP like 2 [Source:HGNC Symbol;Acc:HGNC:25842]	528.1	306.3	**-1.72**	1.15E-24
CDKN2C	cyclin dependent kinase inhibitor 2C [Source:HGNC Symbol;Acc:HGNC:1789]	705.7	422.4	**-1.72**	4.15E-24
PHGDH	phosphoglycerate dehydrogenase [Source:HGNC Symbol;Acc:HGNC:8923]	2141.6	1235.9	**-1.72**	5.52E-21
ADGRA3	adhesion G protein-coupled receptor A3 [Source:HGNC Symbol;Acc:HGNC:13839]	839.9	486.9	**-1.72**	1.28E-20
PSME2	proteasome activator subunit 2 [Source:HGNC Symbol;Acc:HGNC:9569]	578.8	339.5	**-1.72**	1.81E-16
ZGRF1	zinc finger GRF-type containing 1 [Source:HGNC Symbol;Acc:HGNC:25654]	466.1	275.0	**-1.72**	4.56E-13
ST5	suppression of tumorigenicity 5 [Source:HGNC Symbol;Acc:HGNC:11350]	307.4	178.0	**-1.72**	1.72E-11
ZNF365	zinc finger protein 365 [Source:HGNC Symbol;Acc:HGNC:18194]	171.0	99.0	**-1.72**	8.30E-11
KIF22	kinesin family member 22 [Source:HGNC Symbol;Acc:HGNC:6391]	366.5	219.0	**-1.72**	9.61E-11
UBA7	ubiquitin like modifier activating enzyme 7 [Source:HGNC Symbol;Acc:HGNC:12471]	145.7	84.7	**-1.72**	7.95E-09
MCM4	minichromosome maintenance complex component 4 [Source:HGNC Symbol;Acc:HGNC:6947]	2015.6	1229.6	**-1.72**	8.97E-08
ANLN	anillin actin binding protein [Source:HGNC Symbol;Acc:HGNC:14082]	2801.7	1775.6	**-1.72**	1.51E-06
C3orf67	chromosome 3 open reading frame 67 [Source:HGNC Symbol;Acc:HGNC:24763]	74.3	42.4	**-1.72**	2.21E-05
ROR1	receptor tyrosine kinase like orphan receptor 1 [Source:HGNC Symbol;Acc:HGNC:10256]	64.6	37.5	**-1.72**	0.0008449
ZNF90	zinc finger protein 90 [Source:HGNC Symbol;Acc:HGNC:13165]	48.4	27.4	**-1.72**	0.001057
FBLN2	fibulin 2 [Source:HGNC Symbol;Acc:HGNC:3601]	47.1	25.6	**-1.72**	0.002293
CYP46A1	cytochrome P450 family 46 subfamily A member 1 [Source:HGNC Symbol;Acc:HGNC:2641]	33.5	18.8	**-1.72**	0.004889
ZNF815P	zinc finger protein 815, pseudogene [Source:HGNC Symbol;Acc:HGNC:22029]	19.2	10.2	**-1.72**	0.03408
ATP2A3	ATPase sarcoplasmic/endoplasmic reticulum Ca2+ transporting 3 [Source:HGNC Symbol;Acc:HGNC:813]	17.3	9.0	**-1.72**	0.04494
GALNT18	polypeptide N-acetylgalactosaminyltransferase 18 [Source:HGNC Symbol;Acc:HGNC:30488]	1969.1	1143.2	**-1.73**	5.44E-35
MCOLN2	mucolipin 2 [Source:HGNC Symbol;Acc:HGNC:13357]	547.0	314.2	**-1.73**	9.50E-22
EGLN3	egl-9 family hypoxia inducible factor 3 [Source:HGNC Symbol;Acc:HGNC:14661]	326.9	187.2	**-1.73**	9.39E-19
HYLS1	HYLS1, centriolar and ciliogenesis associated [Source:HGNC Symbol;Acc:HGNC:26558]	243.3	142.6	**-1.73**	9.34E-13
REXO5	RNA exonuclease 5 [Source:HGNC Symbol;Acc:HGNC:24661]	172.7	99.4	**-1.73**	1.07E-09
SSTR5	somatostatin receptor 5 [Source:HGNC Symbol;Acc:HGNC:11334]	96.3	55.5	**-1.73**	9.54E-08
CRIP1	cysteine rich protein 1 [Source:HGNC Symbol;Acc:HGNC:2360]	159.9	95.1	**-1.73**	2.05E-07
TRAIP	TRAF interacting protein [Source:HGNC Symbol;Acc:HGNC:30764]	82.2	48.1	**-1.73**	6.76E-06
SLC2A6	solute carrier family 2 member 6 [Source:HGNC Symbol;Acc:HGNC:11011]	100.0	57.1	**-1.73**	7.53E-06
HIST1H4D	histone cluster 1 H4 family member d [Source:HGNC Symbol;Acc:HGNC:4782]	105.6	61.3	**-1.73**	3.07E-05
CDK5R1	cyclin dependent kinase 5 regulatory subunit 1 [Source:HGNC Symbol;Acc:HGNC:1775]	43.2	24.6	**-1.73**	0.0004651
SPRY4-AS1	SPRY4 antisense RNA 1 [Source:HGNC Symbol;Acc:HGNC:53465]	29.9	16.1	**-1.73**	0.01211
C1orf226	chromosome 1 open reading frame 226 [Source:HGNC Symbol;Acc:HGNC:34351]	15.9	8.4	**-1.73**	0.0286
NRP2	neuropilin 2 [Source:HGNC Symbol;Acc:HGNC:8005]	5725.7	3219.8	**-1.74**	7.54E-34
ZC3H8	zinc finger CCCH-type containing 8 [Source:HGNC Symbol;Acc:HGNC:30941]	852.6	489.3	**-1.74**	4.41E-29
ERF	ETS2 repressor factor [Source:HGNC Symbol;Acc:HGNC:3444]	535.5	307.1	**-1.74**	1.01E-28
GSTA4	glutathione S-transferase alpha 4 [Source:HGNC Symbol;Acc:HGNC:4629]	731.6	424.6	**-1.74**	1.10E-28
HSPB8	heat shock protein family B (small) member 8 [Source:HGNC Symbol;Acc:HGNC:30171]	573.4	327.3	**-1.74**	9.36E-26
DNAJC18	DnaJ heat shock protein family (Hsp40) member C18 [Source:HGNC Symbol;Acc:HGNC:28429]	405.3	232.0	**-1.74**	2.07E-20
LNP1	leukemia NUP98 fusion partner 1 [Source:HGNC Symbol;Acc:HGNC:28014]	246.4	140.4	**-1.74**	3.46E-16
PTTG1	pituitary tumor-transforming 1 [Source:HGNC Symbol;Acc:HGNC:9690]	373.1	221.2	**-1.74**	5.77E-14
ROM1	retinal outer segment membrane protein 1 [Source:HGNC Symbol;Acc:HGNC:10254]	230.3	132.4	**-1.74**	6.18E-13
DEPDC1	DEP domain containing 1 [Source:HGNC Symbol;Acc:HGNC:22949]	683.6	427.3	**-1.74**	1.43E-11
ARL4C	ADP ribosylation factor like GTPase 4C [Source:HGNC Symbol;Acc:HGNC:698]	439.7	242.9	**-1.74**	3.31E-10
ITIH6	inter-alpha-trypsin inhibitor heavy chain family member 6 [Source:HGNC Symbol;Acc:HGNC:28907]	3053.9	1763.7	**-1.74**	3.31E-10
RGS2	regulator of G protein signaling 2 [Source:HGNC Symbol;Acc:HGNC:9998]	4148.6	2390.7	**-1.74**	8.91E-10
CCNE2	cyclin E2 [Source:HGNC Symbol;Acc:HGNC:1590]	473.6	282.2	**-1.74**	8.99E-10
FAM149A	family with sequence similarity 149 member A [Source:HGNC Symbol;Acc:HGNC:24527]	132.4	76.0	**-1.74**	6.79E-07
TMC7	transmembrane channel like 7 [Source:HGNC Symbol;Acc:HGNC:23000]	113.0	65.3	**-1.74**	2.85E-06
THEM6	thioesterase superfamily member 6 [Source:HGNC Symbol;Acc:HGNC:29656]	72.3	40.6	**-1.74**	1.43E-05
CDC42EP1	CDC42 effector protein 1 [Source:HGNC Symbol;Acc:HGNC:17014]	71.2	40.2	**-1.74**	2.68E-05
BCL3	B cell CLL/lymphoma 3 [Source:HGNC Symbol;Acc:HGNC:998]	48.6	27.4	**-1.74**	8.47E-05
MILR1	mast cell immunoglobulin like receptor 1 [Source:HGNC Symbol;Acc:HGNC:27570]	48.3	26.3	**-1.74**	0.0003388
BEX2	brain expressed X-linked 2 [Source:HGNC Symbol;Acc:HGNC:30933]	88.9	48.4	**-1.74**	0.0006532
IFI44	interferon induced protein 44 [Source:HGNC Symbol;Acc:HGNC:16938]	46.4	25.2	**-1.74**	0.003146
PCDH18	protocadherin 18 [Source:HGNC Symbol;Acc:HGNC:14268]	59.1	29.8	**-1.74**	0.003482
LINC01705	long intergenic non-protein coding RNA 1705 [Source:HGNC Symbol;Acc:HGNC:52493]	54.9	28.6	**-1.74**	0.003825
C19orf57	chromosome 19 open reading frame 57 [Source:HGNC Symbol;Acc:HGNC:28153]	34.3	18.6	**-1.74**	0.005577
SMIM31	small integral membrane protein 31 [Source:HGNC Symbol;Acc:HGNC:49638]	27.7	15.5	**-1.74**	0.015
FAIM2	Fas apoptotic inhibitory molecule 2 [Source:HGNC Symbol;Acc:HGNC:17067]	21.0	10.9	**-1.74**	0.01587
ROBO3	roundabout guidance receptor 3 [Source:HGNC Symbol;Acc:HGNC:13433]	22.2	11.6	**-1.74**	0.02949
ZNF835	zinc finger protein 835 [Source:HGNC Symbol;Acc:HGNC:34332]	13.2	6.9	**-1.74**	0.03862
CBX5	chromobox 5 [Source:HGNC Symbol;Acc:HGNC:1555]	14679.7	8371.5	**-1.75**	4.71E-100
ARHGEF2	Rho/Rac guanine nucleotide exchange factor 2 [Source:HGNC Symbol;Acc:HGNC:682]	2594.4	1469.3	**-1.75**	2.25E-45
PRKACB	protein kinase cAMP-activated catalytic subunit beta [Source:HGNC Symbol;Acc:HGNC:9381]	1336.1	760.7	**-1.75**	2.28E-36
VASN	vasorin [Source:HGNC Symbol;Acc:HGNC:18517]	1366.7	770.1	**-1.75**	1.46E-24
MIR4435-2HG	MIR4435-2 host gene [Source:HGNC Symbol;Acc:HGNC:35163]	605.3	345.2	**-1.75**	2.62E-24
MIR99AHG	mir-99a-let-7c cluster host gene [Source:HGNC Symbol;Acc:HGNC:1274]	517.3	297.6	**-1.75**	4.14E-21
SYNPO	synaptopodin [Source:HGNC Symbol;Acc:HGNC:30672]	671.6	381.3	**-1.75**	1.62E-20
CELSR1	cadherin EGF LAG seven-pass G-type receptor 1 [Source:HGNC Symbol;Acc:HGNC:1850]	567.9	320.1	**-1.75**	6.08E-18
GRAMD4	GRAM domain containing 4 [Source:HGNC Symbol;Acc:HGNC:29113]	653.9	365.6	**-1.75**	7.05E-16

HTATSF1P2	HIV-1 Tat specific factor 1 pseudogene 2 [Source:HGNC Symbol;Acc:HGNC:38586]	234.6	134.8	**-1.75**	3.36E-15
KIF20A	kinesin family member 20A [Source:HGNC Symbol;Acc:HGNC:9787]	679.5	425.8	**-1.75**	9.87E-13
EFCAB11	EF-hand calcium binding domain 11 [Source:HGNC Symbol;Acc:HGNC:20357]	261.0	148.9	**-1.75**	2.66E-12
TUBA4A	tubulin alpha 4a [Source:HGNC Symbol;Acc:HGNC:12407]	286.2	165.2	**-1.75**	1.19E-11
CDCA8	cell division cycle associated 8 [Source:HGNC Symbol;Acc:HGNC:14629]	257.6	151.9	**-1.75**	6.79E-11
CENPF	centromere protein F [Source:HGNC Symbol;Acc:HGNC:1857]	2599.8	1594.0	**-1.75**	9.28E-09
SERP2	stress associated endoplasmic reticulum protein family member 2 [Source:HGNC Symbol;Acc:HGNC:206	116.8	65.7	**-1.75**	1.44E-08
TMEM56	transmembrane protein 56 [Source:HGNC Symbol;Acc:HGNC:26477]	166.9	93.4	**-1.75**	2.65E-08
MEF2C-AS2	MEF2C antisense RNA 2 [Source:HGNC Symbol;Acc:HGNC:53115]	112.3	62.8	**-1.75**	4.38E-08
GRIK3	glutamate ionotropic receptor kainate type subunit 3 [Source:HGNC Symbol;Acc:HGNC:4581]	109.0	61.7	**-1.75**	5.52E-07
CYS1	cystin 1 [Source:HGNC Symbol;Acc:HGNC:18525]	117.2	66.3	**-1.75**	5.57E-07
VCAN-AS1	VCAN antisense RNA 1 [Source:HGNC Symbol;Acc:HGNC:40163]	93.7	53.4	**-1.75**	5.64E-06
CHTF18	chromosome transmission fidelity factor 18 [Source:HGNC Symbol;Acc:HGNC:18435]	84.6	47.2	**-1.75**	8.19E-06
CCDC141	coiled-coil domain containing 141 [Source:HGNC Symbol;Acc:HGNC:26821]	203.7	112.3	**-1.75**	8.71E-06
GPER1	G protein-coupled estrogen receptor 1 [Source:HGNC Symbol;Acc:HGNC:4485]	112.7	62.3	**-1.75**	9.49E-06
EAF2	ELL associated factor 2 [Source:HGNC Symbol;Acc:HGNC:23115]	106.0	59.1	**-1.75**	2.86E-05
ADAMTS10	ADAM metallopeptidase with thrombospondin type 1 motif 10 [Source:HGNC Symbol;Acc:HGNC:13201]	60.7	33.5	**-1.75**	0.0001649
FBXO43	F-box protein 43 [Source:HGNC Symbol;Acc:HGNC:28521]	22.6	12.2	**-1.75**	0.005443
CTF1	cardiotrophin 1 [Source:HGNC Symbol;Acc:HGNC:2499]	35.7	18.9	**-1.75**	0.009594
CMTM1	CKLF like MARVEL transmembrane domain containing 1 [Source:HGNC Symbol;Acc:HGNC:19172]	19.3	10.0	**-1.75**	0.02305
CNGA1	cyclic nucleotide gated channel alpha 1 [Source:HGNC Symbol;Acc:HGNC:2148]	13.9	7.1	**-1.75**	0.04685
SHMT2	serine hydroxymethyltransferase 2 [Source:HGNC Symbol;Acc:HGNC:10852]	2703.2	1523.2	**-1.77**	7.77E-76
EMP3	epithelial membrane protein 3 [Source:HGNC Symbol;Acc:HGNC:3335]	3414.9	1950.5	**-1.77**	2.09E-48
FKBP14	FK506 binding protein 14 [Source:HGNC Symbol;Acc:HGNC:18625]	2272.1	1295.8	**-1.77**	4.86E-35
SOCS2	suppressor of cytokine signaling 2 [Source:HGNC Symbol;Acc:HGNC:19382]	858.9	487.2	**-1.77**	1.24E-28
NBPF1	NBPF member 1 [Source:HGNC Symbol;Acc:HGNC:26088]	371.9	209.7	**-1.77**	5.32E-18
UNG	uracil DNA glycosylase [Source:HGNC Symbol;Acc:HGNC:12572]	580.3	332.6	**-1.77**	2.54E-14
AC093616.1	anaphase-promoting complex subunit 1-like [Source:NCBI gene;Acc:730268]	138.0	77.2	**-1.77**	7.55E-12
CMBL	carboxymethylenebutenolidase homolog [Source:HGNC Symbol;Acc:HGNC:25090]	174.5	98.6	**-1.77**	1.69E-11
FST	follistatin [Source:HGNC Symbol;Acc:HGNC:3971]	2090.0	1087.9	**-1.77**	3.84E-09
ARRB1	arrestin beta 1 [Source:HGNC Symbol;Acc:HGNC:711]	148.7	82.4	**-1.77**	2.91E-08
POLE2	DNA polymerase epsilon 2, accessory subunit [Source:HGNC Symbol;Acc:HGNC:9178]	195.0	113.4	**-1.77**	6.23E-07
MVB12B	multivesicular body subunit 12B [Source:HGNC Symbol;Acc:HGNC:23368]	52.0	28.9	**-1.77**	6.38E-05
ELN-AS1	ELN antisense RNA 1 [Source:HGNC Symbol;Acc:HGNC:40212]	36.0	19.5	**-1.77**	0.001669
SLC9A7P1	solute carrier family 9 member 7 pseudogene 1 [Source:HGNC Symbol;Acc:HGNC:32679]	35.3	18.7	**-1.77**	0.005113
MYEF2	myelin expression factor 2 [Source:HGNC Symbol;Acc:HGNC:17940]	23.1	12.2	**-1.77**	0.02189
NPTX1	neuronal pentraxin 1 [Source:HGNC Symbol;Acc:HGNC:7952]	16.4	8.2	**-1.77**	0.02407
MEIOB	meiosis specific with OB domains [Source:HGNC Symbol;Acc:HGNC:28569]	16.6	8.4	**-1.77**	0.02668
PRDM16	PR/SET domain 16 [Source:HGNC Symbol;Acc:HGNC:14000]	22.1	11.1	**-1.77**	0.03328
GPR156	G protein-coupled receptor 156 [Source:HGNC Symbol;Acc:HGNC:20844]	24.1	12.6	**-1.77**	0.03606
PPP1R3B	protein phosphatase 1 regulatory subunit 3B [Source:HGNC Symbol;Acc:HGNC:14942]	1653.0	927.9	**-1.78**	9.02E-41
FMNL3	formin like 3 [Source:HGNC Symbol;Acc:HGNC:23698]	796.1	445.5	**-1.78**	3.51E-40
FZD1	frizzled class receptor 1 [Source:HGNC Symbol;Acc:HGNC:4038]	724.0	407.4	**-1.78**	3.40E-39
MEF2C	myocyte enhancer factor 2C [Source:HGNC Symbol;Acc:HGNC:6996]	2024.8	1140.8	**-1.78**	3.73E-25
PER2	period circadian regulator 2 [Source:HGNC Symbol;Acc:HGNC:8846]	411.7	233.0	**-1.78**	1.73E-23
CYTOR	cytoskeleton regulator RNA [Source:HGNC Symbol;Acc:HGNC:28717]	270.3	152.2	**-1.78**	3.99E-17
ABCA2	ATP binding cassette subfamily A member 2 [Source:HGNC Symbol;Acc:HGNC:32]	266.9	148.8	**-1.78**	1.84E-15
DLEU1	deleted in lymphocytic leukemia 1 (non-protein coding) [Source:HGNC Symbol;Acc:HGNC:13747]	257.7	144.0	**-1.78**	4.11E-14
GCNT4	glucosaminyl (N-acetyl) transferase 4, core 2 [Source:HGNC Symbol;Acc:HGNC:17973]	189.8	107.0	**-1.78**	7.28E-12
CARD9	caspase recruitment domain family member 9 [Source:HGNC Symbol;Acc:HGNC:16391]	158.5	88.9	**-1.78**	1.49E-11
DMC1	DNA meiotic recombinase 1 [Source:HGNC Symbol;Acc:HGNC:2927]	150.8	84.7	**-1.78**	1.57E-11
ABCB6	ATP binding cassette subfamily B member 6 (Langereis blood group) [Source:HGNC Symbol;Acc:HGNC:4	355.4	201.7	**-1.78**	6.50E-11
C1QTNF8	C1q and TNF related 8 [Source:HGNC Symbol;Acc:HGNC:31374]	172.5	94.2	**-1.78**	3.88E-10
GINS3	GINS complex subunit 3 [Source:HGNC Symbol;Acc:HGNC:25851]	173.4	98.0	**-1.78**	2.29E-08
TCEAL7	transcription elongation factor A like 7 [Source:HGNC Symbol;Acc:HGNC:28336]	121.4	67.3	**-1.78**	3.04E-08
GALNT16	polypeptide N-acetylgalactosaminyltransferase 16 [Source:HGNC Symbol;Acc:HGNC:23233]	145.1	78.8	**-1.78**	1.45E-07
PSRC1	proline and serine rich coiled-coil 1 [Source:HGNC Symbol;Acc:HGNC:24472]	154.2	88.5	**-1.78**	1.82E-07
ZNF726	zinc finger protein 726 [Source:HGNC Symbol;Acc:HGNC:32462]	81.9	46.4	**-1.78**	3.72E-06
LINC01852	long intergenic non-protein coding RNA 1852 [Source:HGNC Symbol;Acc:HGNC:52668]	43.9	23.7	**-1.78**	0.0001236
TNFRSF21	TNF receptor superfamily member 21 [Source:HGNC Symbol;Acc:HGNC:13469]	34.8	18.8	**-1.78**	0.0007245
HSD17B8	hydroxysteroid 17-beta dehydrogenase 8 [Source:HGNC Symbol;Acc:HGNC:3554]	19.9	10.5	**-1.78**	0.01064
MIR155HG	MIR155 host gene [Source:HGNC Symbol;Acc:HGNC:35460]	19.9	10.3	**-1.78**	0.01595
HUNK	hormonally up-regulated Neu-associated kinase [Source:HGNC Symbol;Acc:HGNC:13326]	15.0	7.7	**-1.78**	0.01989
LMO3	LIM domain only 3 [Source:HGNC Symbol;Acc:HGNC:6643]	17.3	8.5	**-1.78**	0.03085
UCP2	uncoupling protein 2 [Source:HGNC Symbol;Acc:HGNC:12518]	14.3	7.1	**-1.78**	0.03249
RN7SL471P	RNA, 7SL, cytoplasmic 471, pseudogene [Source:HGNC Symbol;Acc:HGNC:46487]	18.7	9.1	**-1.78**	0.03688
MICAL2	microtubule associated monooxygenase, calponin and LIM domain containing 2 [Source:HGNC Symbol;/	1143.1	640.8	**-1.79**	6.39E-29
KIF23	kinesin family member 23 [Source:HGNC Symbol;Acc:HGNC:6392]	1186.3	700.5	**-1.79**	7.38E-16
BUB1	BUB1 mitotic checkpoint serine/threonine kinase [Source:HGNC Symbol;Acc:HGNC:1148]	608.6	363.9	**-1.79**	1.88E-15
FSBP	fibrinogen silencer binding protein [Source:HGNC Symbol;Acc:HGNC:43653]	179.0	100.5	**-1.79**	1.72E-11
RAD54B	RAD54 homolog B [Source:HGNC Symbol;Acc:HGNC:17228]	219.8	124.7	**-1.79**	2.56E-11
PFKFB4	6-phosphofructo-2-kinase/fructose-2,6-biphosphatase 4 [Source:HGNC Symbol;Acc:HGNC:8875]	173.5	95.7	**-1.79**	3.10E-11

PDE1C	phosphodiesterase 1C [Source:HGNC Symbol;Acc:HGNC:8776]	133.9	74.5	**-1.79**	2.26E-10
RBM43	RNA binding motif protein 43 [Source:HGNC Symbol;Acc:HGNC:24790]	134.4	74.4	**-1.79**	5.94E-10
HMMR	hyaluronan mediated motility receptor [Source:HGNC Symbol;Acc:HGNC:5012]	1466.6	890.7	**-1.79**	9.58E-08
AGAP11	ArfGAP with GTPase domain, ankyrin repeat and PH domain 11 [Source:HGNC Symbol;Acc:HGNC:29421]	86.9	47.7	**-1.79**	9.79E-08
RIN1	Ras and Rab interactor 1 [Source:HGNC Symbol;Acc:HGNC:18749]	123.8	67.6	**-1.79**	1.19E-07
SGO1	shugoshin 1 [Source:HGNC Symbol;Acc:HGNC:25088]	112.0	61.9	**-1.79**	9.53E-07
MSMP	microseminoprotein, prostate associated [Source:HGNC Symbol;Acc:HGNC:29663]	2346.9	1327.9	**-1.79**	1.63E-06
GLI2	GLI family zinc finger 2 [Source:HGNC Symbol;Acc:HGNC:4318]	73.2	39.9	**-1.79**	2.69E-06
MAP3K14	mitogen-activated protein kinase kinase kinase 14 [Source:HGNC Symbol;Acc:HGNC:6853]	111.7	63.3	**-1.79**	1.35E-05
MFAP2	microfibril associated protein 2 [Source:HGNC Symbol;Acc:HGNC:7033]	25.1	13.2	**-1.79**	0.002807
FAM78B	family with sequence similarity 78 member B [Source:HGNC Symbol;Acc:HGNC:13495]	18.2	8.8	**-1.79**	0.03478
RNU12	RNA, U12 small nuclear [Source:HGNC Symbol;Acc:HGNC:19380]	10.2	4.9	**-1.79**	0.04544
PRSS23	serine protease 23 [Source:HGNC Symbol;Acc:HGNC:14370]	15630.8	8720.5	**-1.80**	4.27E-71
SULF1	sulfatase 1 [Source:HGNC Symbol;Acc:HGNC:20391]	27348.2	15445.7	**-1.80**	1.37E-56
SYNJ2	synaptojanin 2 [Source:HGNC Symbol;Acc:HGNC:11504]	1911.4	1049.9	**-1.80**	1.83E-45
NDUFA4L2	NDUFA4, mitochondrial complex associated like 2 [Source:HGNC Symbol;Acc:HGNC:29836]	2034.8	1119.5	**-1.80**	2.81E-31
PRC1-AS1	PRC1 antisense RNA 1 [Source:HGNC Symbol;Acc:HGNC:48587]	310.3	181.7	**-1.80**	4.03E-09
PRR5L	proline rich 5 like [Source:HGNC Symbol;Acc:HGNC:25878]	110.6	59.0	**-1.80**	2.23E-07
NEURL1B	neuralized E3 ubiquitin protein ligase 1B [Source:HGNC Symbol;Acc:HGNC:35422]	64.4	33.8	**-1.80**	0.0001952
PRKG1-AS1	PRKG1 antisense RNA 1 [Source:HGNC Symbol;Acc:HGNC:45029]	36.7	19.3	**-1.80**	0.0003857
PAOX	polyamine oxidase [Source:HGNC Symbol;Acc:HGNC:20837]	28.1	14.5	**-1.80**	0.001715
CCR10	C-C motif chemokine receptor 10 [Source:HGNC Symbol;Acc:HGNC:4474]	18.5	9.5	**-1.80**	0.006182
VNN2	vanin 2 [Source:HGNC Symbol;Acc:HGNC:12706]	25.7	12.8	**-1.80**	0.008705
CLEC18A	C-type lectin domain family 18 member A [Source:HGNC Symbol;Acc:HGNC:30388]	22.5	11.5	**-1.80**	0.01084
CACNA2D4	calcium voltage-gated channel auxiliary subunit alpha2delta 4 [Source:HGNC Symbol;Acc:HGNC:20202]	18.2	9.2	**-1.80**	0.02236
SERPIND1	serpin family D member 1 [Source:HGNC Symbol;Acc:HGNC:4838]	20.2	9.7	**-1.80**	0.03307
DEFB109F	defensin beta 109F (pseudogene) [Source:HGNC Symbol;Acc:HGNC:38066]	13.2	6.3	**-1.80**	0.03526
RND1	Rho family GTPase 1 [Source:HGNC Symbol;Acc:HGNC:18314]	16.8	8.4	**-1.80**	0.04174
GAS1	growth arrest specific 1 [Source:HGNC Symbol;Acc:HGNC:4165]	2381.9	1317.7	**-1.82**	2.07E-39
HMOX1	heme oxygenase 1 [Source:HGNC Symbol;Acc:HGNC:5013]	3579.7	2007.3	**-1.82**	4.39E-30
NFATC2	nuclear factor of activated T cells 2 [Source:HGNC Symbol;Acc:HGNC:7776]	342.9	188.9	**-1.82**	1.66E-22
CPED1	cadherin like and PC-esterase domain containing 1 [Source:HGNC Symbol;Acc:HGNC:26159]	653.2	364.4	**-1.82**	3.82E-21
BRCA1	BRCA1, DNA repair associated [Source:HGNC Symbol;Acc:HGNC:1100]	1377.9	790.3	**-1.82**	9.63E-19
DYSF	dysferlin [Source:HGNC Symbol;Acc:HGNC:3097]	310.4	171.4	**-1.82**	8.51E-14
C11orf96	chromosome 11 open reading frame 96 [Source:HGNC Symbol;Acc:HGNC:38675]	234.5	132.8	**-1.82**	1.79E-12
HMMR-AS1	HMMR antisense RNA 1 [Source:HGNC Symbol;Acc:HGNC:49149]	147.3	85.5	**-1.82**	1.09E-05
SLC9A5	solute carrier family 9 member A5 [Source:HGNC Symbol;Acc:HGNC:11078]	27.1	14.2	**-1.82**	0.003285
CAVIN4	caveolae associated protein 4 [Source:HGNC Symbol;Acc:HGNC:33742]	27.5	13.9	**-1.82**	0.01048
ACOX2	acyl-CoA oxidase 2 [Source:HGNC Symbol;Acc:HGNC:120]	15.9	7.7	**-1.82**	0.026
RNU6-146P	RNA, U6 small nuclear 146, pseudogene [Source:HGNC Symbol;Acc:HGNC:47109]	9.7	4.5	**-1.82**	0.03929
RFPL1S	RFPL1 antisense RNA 1 [Source:HGNC Symbol;Acc:HGNC:9978]	18.0	8.7	**-1.82**	0.04242
ISM1	isthmin 1 [Source:HGNC Symbol;Acc:HGNC:16213]	5671.3	3108.5	**-1.83**	1.46E-46
GLDN	gliomedin [Source:HGNC Symbol;Acc:HGNC:29514]	10073.6	5368.2	**-1.83**	1.02E-44
SGCD	sarcoglycan delta [Source:HGNC Symbol;Acc:HGNC:10807]	2145.5	1186.2	**-1.83**	1.87E-27
CTHRC1	collagen triple helix repeat containing 1 [Source:HGNC Symbol;Acc:HGNC:18831]	360.4	198.1	**-1.83**	1.19E-22
MKI67	marker of proliferation Ki-67 [Source:HGNC Symbol;Acc:HGNC:7107]	1363.2	783.2	**-1.83**	5.60E-16
GRIA3	glutamate ionotropic receptor AMPA type subunit 3 [Source:HGNC Symbol;Acc:HGNC:4573]	261.2	143.6	**-1.83**	8.56E-16
NUSAP1	nucleolar and spindle associated protein 1 [Source:HGNC Symbol;Acc:HGNC:18538]	1067.1	635.9	**-1.83**	1.55E-13
H2AFX	H2A histone family member X [Source:HGNC Symbol;Acc:HGNC:4739]	231.5	127.1	**-1.83**	4.76E-13
TOP2A	DNA topoisomerase II alpha [Source:HGNC Symbol;Acc:HGNC:11989]	4241.1	2515.4	**-1.83**	2.08E-10
PRKX	protein kinase X-linked [Source:HGNC Symbol;Acc:HGNC:9441]	103.8	56.6	**-1.83**	7.75E-09
SFRP1	secreted frizzled related protein 1 [Source:HGNC Symbol;Acc:HGNC:10776]	75.8	40.5	**-1.83**	1.23E-06
GPR153	G protein-coupled receptor 153 [Source:HGNC Symbol;Acc:HGNC:23618]	77.8	41.6	**-1.83**	7.73E-06
HEXIM2	hexamethylene bisacetamide inducible 2 [Source:HGNC Symbol;Acc:HGNC:28591]	56.7	30.5	**-1.83**	1.21E-05
FANCD2OS	FANCD2 opposite strand [Source:HGNC Symbol;Acc:HGNC:28623]	60.8	32.5	**-1.83**	4.55E-05
ARAP3	ArfGAP with RhoGAP domain, ankyrin repeat and PH domain 3 [Source:HGNC Symbol;Acc:HGNC:24097]	55.3	29.4	**-1.83**	0.0001925
GPR137C	G protein-coupled receptor 137C [Source:HGNC Symbol;Acc:HGNC:25445]	37.2	19.8	**-1.83**	0.0002147
NEURL1	neuralized E3 ubiquitin protein ligase 1 [Source:HGNC Symbol;Acc:HGNC:7761]	13.7	6.4	**-1.83**	0.03238
VN1R1	vomeronasal 1 receptor 1 [Source:HGNC Symbol;Acc:HGNC:13548]	11.0	5.2	**-1.83**	0.03703
USP2-AS1	USP2 antisense RNA 1 (head to head) [Source:HGNC Symbol;Acc:HGNC:48673]	11.2	5.3	**-1.83**	0.0434
TRPS1	transcriptional repressor GATA binding 1 [Source:HGNC Symbol;Acc:HGNC:12340]	17282.2	9380.8	**-1.84**	3.79E-107
DOK1	docking protein 1 [Source:HGNC Symbol;Acc:HGNC:2990]	2053.4	1118.7	**-1.84**	3.68E-56
ZNF385D	zinc finger protein 385D [Source:HGNC Symbol;Acc:HGNC:26191]	3450.4	1878.1	**-1.84**	1.18E-50
FLNB	filamin B [Source:HGNC Symbol;Acc:HGNC:3755]	2499.5	1368.0	**-1.84**	7.83E-41
HIST1H4H	histone cluster 1 H4 family member h [Source:HGNC Symbol;Acc:HGNC:4788]	875.1	483.5	**-1.84**	3.65E-29
GPT2	glutamic--pyruvic transaminase 2 [Source:HGNC Symbol;Acc:HGNC:18062]	903.5	482.2	**-1.84**	2.67E-28
CCDC34	coiled-coil domain containing 34 [Source:HGNC Symbol;Acc:HGNC:25079]	319.2	174.8	**-1.84**	2.71E-15
NDC80	NDC80, kinetochore complex component [Source:HGNC Symbol;Acc:HGNC:16909]	519.6	305.5	**-1.84**	4.70E-14
ULBP2	UL16 binding protein 2 [Source:HGNC Symbol;Acc:HGNC:14894]	276.1	145.5	**-1.84**	3.39E-12
PRC1	protein regulator of cytokinesis 1 [Source:HGNC Symbol;Acc:HGNC:9341]	1825.1	1069.5	**-1.84**	1.22E-09
POC1A	POC1 centriolar protein A [Source:HGNC Symbol;Acc:HGNC:24488]	105.1	57.4	**-1.84**	1.95E-06
HSD17B6	hydroxysteroid 17-beta dehydrogenase 6 [Source:HGNC Symbol;Acc:HGNC:23316]	73.2	40.2	**-1.84**	2.39E-05

CYP7B1	cytochrome P450 family 7 subfamily B member 1 [Source:HGNC Symbol;Acc:HGNC:2652]	98.6	53.3	**-1.84**	8.58E-05
EGF	epidermal growth factor [Source:HGNC Symbol;Acc:HGNC:3229]	35.6	18.9	**-1.84**	0.0006503
GPRIN3	GPRIN family member 3 [Source:HGNC Symbol;Acc:HGNC:27733]	29.3	15.0	**-1.84**	0.0008222
ANO8	anoctamin 8 [Source:HGNC Symbol;Acc:HGNC:29329]	31.6	16.4	**-1.84**	0.005302
TMEM74	transmembrane protein 74 [Source:HGNC Symbol;Acc:HGNC:26409]	16.6	7.8	**-1.84**	0.02104
RNU7-1	RNA, U7 small nuclear 1 [Source:HGNC Symbol;Acc:HGNC:34033]	15.3	7.2	**-1.84**	0.02758
SYT2	synaptotagmin 2 [Source:HGNC Symbol;Acc:HGNC:11510]	19.8	9.6	**-1.84**	0.02961
POU2F2	POU class 2 homeobox 2 [Source:HGNC Symbol;Acc:HGNC:9213]	9.6	4.3	**-1.84**	0.04764
MPO	myeloperoxidase [Source:HGNC Symbol;Acc:HGNC:7218]	8.8	4.0	**-1.84**	0.04914
BCL2	BCL2, apoptosis regulator [Source:HGNC Symbol;Acc:HGNC:990]	1625.6	880.3	**-1.85**	6.39E-53
FAM107B	family with sequence similarity 107 member B [Source:HGNC Symbol;Acc:HGNC:23726]	1477.2	794.6	**-1.85**	7.71E-38
SMTN	smoothelin [Source:HGNC Symbol;Acc:HGNC:11126]	814.0	440.6	**-1.85**	6.13E-30
LINC00346	long intergenic non-protein coding RNA 346 [Source:HGNC Symbol;Acc:HGNC:27492]	405.7	218.5	**-1.85**	2.34E-25
HJURP	Holliday junction recognition protein [Source:HGNC Symbol;Acc:HGNC:25444]	598.9	335.9	**-1.85**	6.96E-19
RFC3	replication factor C subunit 3 [Source:HGNC Symbol;Acc:HGNC:9971]	415.3	230.8	**-1.85**	1.28E-12
DDIAS	DNA damage induced apoptosis suppressor [Source:HGNC Symbol;Acc:HGNC:26351]	164.3	92.2	**-1.85**	1.60E-08
SYNE1-AS1	SYNE1 antisense RNA 1 [Source:HGNC Symbol;Acc:HGNC:40793]	107.2	55.7	**-1.85**	5.36E-07
SPTBN4	spectrin beta, non-erythrocytic 4 [Source:HGNC Symbol;Acc:HGNC:14896]	33.6	17.5	**-1.85**	0.000738
MTMR9LP	myotubularin related protein 9-like, pseudogene [Source:HGNC Symbol;Acc:HGNC:27920]	31.8	16.3	**-1.85**	0.0007816
DCHS2	dachsous cadherin-related 2 [Source:HGNC Symbol;Acc:HGNC:23111]	40.4	21.1	**-1.85**	0.005246
FGFR2	fibroblast growth factor receptor 2 [Source:HGNC Symbol;Acc:HGNC:3689]	3062.6	1637.6	**-1.87**	1.64E-82
PLOD2	procollagen-lysine,2-oxoglutarate 5-dioxygenase 2 [Source:HGNC Symbol;Acc:HGNC:9082]	35062.8	18787.1	**-1.87**	2.10E-58
SH3BP2	SH3 domain binding protein 2 [Source:HGNC Symbol;Acc:HGNC:10825]	910.9	484.0	**-1.87**	4.10E-41
TIMELESS	timeless circadian regulator [Source:HGNC Symbol;Acc:HGNC:11813]	769.6	419.3	**-1.87**	1.60E-28
CABCOCO1	ciliary associated calcium binding coiled-coil 1 [Source:HGNC Symbol;Acc:HGNC:28678]	253.5	133.7	**-1.87**	3.63E-21
CST6	cystatin E/M [Source:HGNC Symbol;Acc:HGNC:2478]	477.9	255.6	**-1.87**	4.52E-20
B3GALNT1	beta-1,3-N-acetylgalactosaminyltransferase 1 (globoside blood group) [Source:HGNC Symbol;Acc:HGNC	759.1	407.6	**-1.87**	2.99E-18
KCNA6	potassium voltage-gated channel subfamily A member 6 [Source:HGNC Symbol;Acc:HGNC:6225]	425.6	224.2	**-1.87**	7.75E-17
SCN1B	sodium voltage-gated channel beta subunit 1 [Source:HGNC Symbol;Acc:HGNC:10586]	280.9	152.4	**-1.87**	3.66E-11
CHAF1B	chromatin assembly factor 1 subunit B [Source:HGNC Symbol;Acc:HGNC:1911]	197.8	108.2	**-1.87**	6.66E-10
ZNF724	zinc finger protein 724 [Source:HGNC Symbol;Acc:HGNC:32460]	217.7	120.9	**-1.87**	5.15E-08
SNORD108	small nucleolar RNA, C/D box 108 [Source:HGNC Symbol;Acc:HGNC:32772]	61.5	32.4	**-1.87**	8.95E-07
ZNF469	zinc finger protein 469 [Source:HGNC Symbol;Acc:HGNC:23216]	96.5	48.8	**-1.87**	3.33E-06
APCDD1L-DT	APCDD1L divergent transcript [Source:HGNC Symbol;Acc:HGNC:27152]	185.2	59.0	**-1.87**	1.15E-05
TAC1	tachykinin precursor 1 [Source:HGNC Symbol;Acc:HGNC:11517]	43.7	22.8	**-1.87**	4.62E-05
ZNF280B	zinc finger protein 280B [Source:HGNC Symbol;Acc:HGNC:23022]	38.7	19.8	**-1.87**	0.0001268
BMS1P3	BMS1, ribosome biogenesis factor pseudogene 3 [Source:HGNC Symbol;Acc:HGNC:23651]	35.9	18.6	**-1.87**	0.0002266
MEX3A	mex-3 RNA binding family member A [Source:HGNC Symbol;Acc:HGNC:33482]	27.5	13.6	**-1.87**	0.002276
MCAM	melanoma cell adhesion molecule [Source:HGNC Symbol;Acc:HGNC:6934]	40.1	23.4	**-1.87**	0.005516
PLEKHG5	pleckstrin homology and RhoGEF domain containing G5 [Source:HGNC Symbol;Acc:HGNC:29105]	19.2	9.0	**-1.87**	0.01593
LAX1	lymphocyte transmembrane adaptor 1 [Source:HGNC Symbol;Acc:HGNC:26005]	12.6	5.8	**-1.87**	0.02529
ANKH	ANKH inorganic pyrophosphate transport regulator [Source:HGNC Symbol;Acc:HGNC:15492]	10392.6	5497.4	**-1.88**	3.45E-96
MYO10	myosin X [Source:HGNC Symbol;Acc:HGNC:7593]	2614.6	1388.8	**-1.88**	5.40E-65
FAM162A	family with sequence similarity 162 member A [Source:HGNC Symbol;Acc:HGNC:17865]	992.8	525.2	**-1.88**	2.20E-36
SSX2IP	SSX family member 2 interacting protein [Source:HGNC Symbol;Acc:HGNC:16509]	1002.3	535.2	**-1.88**	2.96E-34
GALNT8	polypeptide N-acetylgalactosaminyltransferase 8 [Source:HGNC Symbol;Acc:HGNC:4130]	713.3	370.8	**-1.88**	1.90E-20
LOXL1-AS1	LOXL1 antisense RNA 1 [Source:HGNC Symbol;Acc:HGNC:44169]	122.9	65.8	**-1.88**	2.37E-09
XRCC2	X-ray repair cross complementing 2 [Source:HGNC Symbol;Acc:HGNC:12829]	201.6	112.1	**-1.88**	1.07E-08
TNXB	tenascin XB [Source:HGNC Symbol;Acc:HGNC:11976]	1329.5	709.8	**-1.88**	2.24E-08
SLC8A3	solute carrier family 8 member A3 [Source:HGNC Symbol;Acc:HGNC:11070]	52.2	26.8	**-1.88**	5.13E-06
ROBO2	roundabout guidance receptor 2 [Source:HGNC Symbol;Acc:HGNC:10250]	88.4	43.9	**-1.88**	9.07E-06
FILIP1L	filamin A interacting protein 1 like [Source:HGNC Symbol;Acc:HGNC:24589]	62.2	32.7	**-1.88**	1.11E-05
DNM3	dynamin 3 [Source:HGNC Symbol;Acc:HGNC:29125]	91.5	47.6	**-1.88**	1.29E-05
FGF11	fibroblast growth factor 11 [Source:HGNC Symbol;Acc:HGNC:3667]	45.4	23.6	**-1.88**	2.04E-05
BUB1B-PAK6	BUB1B-PAK6 readthrough [Source:HGNC Symbol;Acc:HGNC:52276]	46.8	24.2	**-1.88**	0.0002191
CASP1	caspase 1 [Source:HGNC Symbol;Acc:HGNC:1499]	33.2	17.0	**-1.88**	0.001115
PARP15	poly(ADP-ribose) polymerase family member 15 [Source:HGNC Symbol;Acc:HGNC:26876]	12.8	5.8	**-1.88**	0.02819
ZNF462	zinc finger protein 462 [Source:HGNC Symbol;Acc:HGNC:21684]	1010.7	535.1	**-1.89**	1.00E-37
VSTM4	V-set and transmembrane domain containing 4 [Source:HGNC Symbol;Acc:HGNC:26470]	664.4	349.0	**-1.89**	2.27E-34
DDIT3	DNA damage inducible transcript 3 [Source:HGNC Symbol;Acc:HGNC:2726]	727.9	378.4	**-1.89**	1.84E-26
CKAP2	cytoskeleton associated protein 2 [Source:HGNC Symbol;Acc:HGNC:1990]	1159.3	654.4	**-1.89**	1.12E-18
FANCD2	Fanconi anemia complementation group D2 [Source:HGNC Symbol;Acc:HGNC:3585]	469.6	259.1	**-1.89**	1.54E-16
GSN-AS1	GSN antisense RNA 1 [Source:HGNC Symbol;Acc:HGNC:23372]	180.4	94.5	**-1.89**	1.57E-16
NXN	nucleoredoxin [Source:HGNC Symbol;Acc:HGNC:18008]	120.9	63.1	**-1.89**	9.44E-14
KLHL13	kelch like family member 13 [Source:HGNC Symbol;Acc:HGNC:22931]	204.8	105.8	**-1.89**	3.78E-13
TMEM17	transmembrane protein 17 [Source:HGNC Symbol;Acc:HGNC:26623]	109.4	56.2	**-1.89**	4.52E-09
CENPI	centromere protein I [Source:HGNC Symbol;Acc:HGNC:3968]	220.6	121.2	**-1.89**	4.74E-09
ME3	malic enzyme 3 [Source:HGNC Symbol;Acc:HGNC:6985]	77.3	40.3	**-1.89**	9.03E-07
APCDD1	APC down-regulated 1 [Source:HGNC Symbol;Acc:HGNC:15718]	43.9	22.4	**-1.89**	4.69E-05
TTYH2	tweety family member 2 [Source:HGNC Symbol;Acc:HGNC:13877]	38.2	19.6	**-1.89**	9.29E-05
TRIM45	tripartite motif containing 45 [Source:HGNC Symbol;Acc:HGNC:19018]	36.6	18.2	**-1.89**	0.0003407
SPAG4	sperm associated antigen 4 [Source:HGNC Symbol;Acc:HGNC:11214]	25.1	12.3	**-1.89**	0.003928

RIMS3	regulating synaptic membrane exocytosis 3 [Source:HGNC Symbol;Acc:HGNC:21292]	20.9	9.9	**-1.89**	0.006186
DPF3	double PHD fingers 3 [Source:HGNC Symbol;Acc:HGNC:17427]	17.9	8.5	**-1.89**	0.01489
FOXP4-AS1	FOXP4 antisense RNA 1 [Source:HGNC Symbol;Acc:HGNC:50332]	11.8	5.4	**-1.89**	0.01844
BOK-AS1	BOK antisense RNA 1 [Source:HGNC Symbol;Acc:HGNC:35125]	10.3	4.8	**-1.89**	0.01995
TSPAN32	tetraspanin 32 [Source:HGNC Symbol;Acc:HGNC:13410]	11.4	4.8	**-1.89**	0.0377
CCDC80	coiled-coil domain containing 80 [Source:HGNC Symbol;Acc:HGNC:30649]	24419.6	12728.1	**-1.91**	6.25E-60
HIST1H2BC	histone cluster 1 H2B family member c [Source:HGNC Symbol;Acc:HGNC:4757]	968.3	519.1	**-1.91**	1.50E-33
BPGM	bisphosphoglycerate mutase [Source:HGNC Symbol;Acc:HGNC:1093]	1123.8	568.2	**-1.91**	8.32E-32
PHLDA3	pleckstrin homology like domain family A member 3 [Source:HGNC Symbol;Acc:HGNC:8934]	287.6	151.7	**-1.91**	1.42E-20
SESN2	sestrin 2 [Source:HGNC Symbol;Acc:HGNC:20746]	395.8	201.3	**-1.91**	1.31E-18
TTK	TTK protein kinase [Source:HGNC Symbol;Acc:HGNC:12401]	426.0	230.8	**-1.91**	6.38E-13
MX1	MX dynamin like GTPase 1 [Source:HGNC Symbol;Acc:HGNC:7532]	232.7	117.1	**-1.91**	1.37E-09
CD83	CD83 molecule [Source:HGNC Symbol;Acc:HGNC:1703]	95.2	49.2	**-1.91**	4.16E-09
RGN	regucalcin [Source:HGNC Symbol;Acc:HGNC:9989]	121.1	62.9	**-1.91**	9.80E-09
ERCC6L	ERCC excision repair 6 like, spindle assembly checkpoint helicase [Source:HGNC Symbol;Acc:HGNC:2079	245.8	136.7	**-1.91**	6.13E-08
SKA3	spindle and kinetochore associated complex subunit 3 [Source:HGNC Symbol;Acc:HGNC:20262]	213.6	122.0	**-1.91**	1.03E-07
CDCA3	cell division cycle associated 3 [Source:HGNC Symbol;Acc:HGNC:14624]	129.0	68.5	**-1.91**	1.12E-07
ISYNA1	inositol-3-phosphate synthase 1 [Source:HGNC Symbol;Acc:HGNC:29821]	69.1	35.7	**-1.91**	2.58E-07
PDCD1LG2	programmed cell death 1 ligand 2 [Source:HGNC Symbol;Acc:HGNC:18731]	149.6	76.7	**-1.91**	4.96E-07
MAPK8IP1	mitogen-activated protein kinase 8 interacting protein 1 [Source:HGNC Symbol;Acc:HGNC:6882]	62.4	31.5	**-1.91**	5.62E-07
RAPGEF4	Rap guanine nucleotide exchange factor 4 [Source:HGNC Symbol;Acc:HGNC:16626]	54.9	27.5	**-1.91**	3.79E-05
ADM2	adrenomedullin 2 [Source:HGNC Symbol;Acc:HGNC:28898]	40.7	19.8	**-1.91**	0.001545
ECSCR	endothelial cell surface expressed chemotaxis and apoptosis regulator [Source:HGNC Symbol;Acc:HGNC	14.8	6.4	**-1.91**	0.02833
PPIAP90	peptidylprolyl isomerase A pseudogene 90 [Source:HGNC Symbol;Acc:HGNC:53714]	10.5	4.7	**-1.91**	0.02849
IL36B	interleukin 36 beta [Source:HGNC Symbol;Acc:HGNC:15564]	16.6	7.6	**-1.91**	0.02916
LYPD6	LY6/PLAUR domain containing 6 [Source:HGNC Symbol;Acc:HGNC:28751]	14.8	6.6	**-1.91**	0.03654
PHTF2	putative homeodomain transcription factor 2 [Source:HGNC Symbol;Acc:HGNC:13411]	3569.3	1854.4	**-1.92**	1.76E-83
MALL	mal, T cell differentiation protein like [Source:HGNC Symbol;Acc:HGNC:6818]	1041.3	543.1	**-1.92**	2.01E-35
DBN1	drebrin 1 [Source:HGNC Symbol;Acc:HGNC:2695]	749.8	390.5	**-1.92**	1.69E-29
ADSSL1	adenylosuccinate synthase like 1 [Source:HGNC Symbol;Acc:HGNC:20093]	278.6	145.5	**-1.92**	1.56E-21
FANCI	Fanconi anemia complementation group I [Source:HGNC Symbol;Acc:HGNC:25568]	1031.3	564.7	**-1.92**	3.08E-20
CENPH	centromere protein H [Source:HGNC Symbol;Acc:HGNC:17268]	389.6	203.2	**-1.92**	3.91E-19
NRSN2	neurensin 2 [Source:HGNC Symbol;Acc:HGNC:16229]	238.9	124.0	**-1.92**	1.98E-18
HTRA3	HtrA serine peptidase 3 [Source:HGNC Symbol;Acc:HGNC:30406]	160.0	81.3	**-1.92**	1.87E-11
MCM2	minichromosome maintenance complex component 2 [Source:HGNC Symbol;Acc:HGNC:6944]	841.5	456.8	**-1.92**	9.24E-08
VEGFC	vascular endothelial growth factor C [Source:HGNC Symbol;Acc:HGNC:12682]	158.8	80.7	**-1.92**	1.33E-07
TMEM134	transmembrane protein 134 [Source:HGNC Symbol;Acc:HGNC:26142]	49.6	25.7	**-1.92**	4.97E-06
KAZN	kazrin, periplakin interacting protein [Source:HGNC Symbol;Acc:HGNC:29173]	54.1	27.4	**-1.92**	3.82E-05
C21orf58	chromosome 21 open reading frame 58 [Source:HGNC Symbol;Acc:HGNC:1300]	35.1	17.4	**-1.92**	0.0002257
SLC4A3	solute carrier family 4 member 3 [Source:HGNC Symbol;Acc:HGNC:11029]	43.3	21.2	**-1.92**	0.001419
C17orf53	chromosome 17 open reading frame 53 [Source:HGNC Symbol;Acc:HGNC:28460]	22.8	11.0	**-1.92**	0.00364
TPPP3	tubulin polymerization promoting protein family member 3 [Source:HGNC Symbol;Acc:HGNC:24162]	23.1	10.8	**-1.92**	0.00658
EPHB1	EPH receptor B1 [Source:HGNC Symbol;Acc:HGNC:3392]	25.2	12.0	**-1.92**	0.006592
CENPM	centromere protein M [Source:HGNC Symbol;Acc:HGNC:18352]	30.0	14.7	**-1.92**	0.007144
FCRLA	Fc receptor like A [Source:HGNC Symbol;Acc:HGNC:18504]	12.9	5.2	**-1.92**	0.04294
CALCA	calcitonin related polypeptide alpha [Source:HGNC Symbol;Acc:HGNC:1437]	7.8	3.2	**-1.92**	0.04753
IQSEC1	IQ motif and Sec7 domain 1 [Source:HGNC Symbol;Acc:HGNC:29112]	685.8	354.9	**-1.93**	4.92E-35
MATN2	matrilin 2 [Source:HGNC Symbol;Acc:HGNC:6908]	1052.6	540.7	**-1.93**	7.01E-23
KIF11	kinesin family member 11 [Source:HGNC Symbol;Acc:HGNC:6388]	1464.7	811.2	**-1.93**	7.63E-23
SCUBE3	signal peptide, CUB domain and EGF like domain containing 3 [Source:HGNC Symbol;Acc:HGNC:13655]	559.4	287.1	**-1.93**	2.82E-21
BOK	BOK, BCL2 family apoptosis regulator [Source:HGNC Symbol;Acc:HGNC:1087]	317.4	162.7	**-1.93**	6.86E-20
TP53	tumor protein p53 [Source:HGNC Symbol;Acc:HGNC:11998]	387.4	198.2	**-1.93**	2.61E-17
FOXM1	forkhead box M1 [Source:HGNC Symbol;Acc:HGNC:3818]	209.8	113.8	**-1.93**	1.16E-09
DLX3	distal-less homeobox 3 [Source:HGNC Symbol;Acc:HGNC:2916]	75.8	38.1	**-1.93**	9.44E-08
NRIP3	nuclear receptor interacting protein 3 [Source:HGNC Symbol;Acc:HGNC:1167]	77.1	39.8	**-1.93**	2.47E-07
LINC01140	long intergenic non-protein coding RNA 1140 [Source:HGNC Symbol;Acc:HGNC:27922]	110.0	53.7	**-1.93**	6.03E-07
ADAMTSL1	ADAMTS like 1 [Source:HGNC Symbol;Acc:HGNC:14632]	95.4	49.0	**-1.93**	1.59E-06
TNFSF10	TNF superfamily member 10 [Source:HGNC Symbol;Acc:HGNC:11925]	62.5	29.6	**-1.93**	3.53E-05
GSTM1	glutathione S-transferase mu 1 [Source:HGNC Symbol;Acc:HGNC:4632]	29.6	13.1	**-1.93**	0.002067
CES4A	carboxylesterase 4A [Source:HGNC Symbol;Acc:HGNC:26741]	22.7	10.8	**-1.93**	0.002607
PHYHIP	phytanoyl-CoA 2-hydroxylase interacting protein [Source:HGNC Symbol;Acc:HGNC:16865]	18.7	8.5	**-1.93**	0.01139
KLF2P2	Kruppel like factor 2 pseudogene 2 [Source:HGNC Symbol;Acc:HGNC:49281]	8.0	3.2	**-1.93**	0.04011
LINC00211	long intergenic non-protein coding RNA 211 [Source:HGNC Symbol;Acc:HGNC:37459]	8.8	3.6	**-1.93**	0.0449
HIF1A	hypoxia inducible factor 1 subunit alpha [Source:HGNC Symbol;Acc:HGNC:4910]	28876.8	15036.6	**-1.95**	5.77E-79
NIPSNAP1	nipsnap homolog 1 [Source:HGNC Symbol;Acc:HGNC:7827]	568.6	292.1	**-1.95**	1.00E-31
LMCD1	LIM and cysteine rich domains 1 [Source:HGNC Symbol;Acc:HGNC:6633]	490.6	252.2	**-1.95**	1.39E-28
EPHA3	EPH receptor A3 [Source:HGNC Symbol;Acc:HGNC:3387]	240.0	121.4	**-1.95**	1.49E-17
TRIP13	thyroid hormone receptor interactor 13 [Source:HGNC Symbol;Acc:HGNC:12307]	295.5	158.1	**-1.95**	3.76E-10
CDC25A	cell division cycle 25A [Source:HGNC Symbol;Acc:HGNC:1725]	246.4	129.7	**-1.95**	7.75E-09
ZNF821	zinc finger protein 821 [Source:HGNC Symbol;Acc:HGNC:28043]	69.7	34.6	**-1.95**	8.05E-08
CALHM6	calcium homeostasis modulator family member 6 [Source:HGNC Symbol;Acc:HGNC:33391]	54.2	26.5	**-1.95**	4.95E-06
CDT1	chromatin licensing and DNA replication factor 1 [Source:HGNC Symbol;Acc:HGNC:24576]	66.8	34.3	**-1.95**	1.82E-05

MIR210HG	MIR210 host gene [Source:HGNC Symbol;Acc:HGNC:39524]	62.1	30.9	**-1.95**	2.93E-05
CAPN5	calpain 5 [Source:HGNC Symbol;Acc:HGNC:1482]	48.9	24.0	**-1.95**	4.54E-05
MID1	midline 1 [Source:HGNC Symbol;Acc:HGNC:7095]	37.5	18.1	**-1.95**	0.0002187
TMEM171	transmembrane protein 171 [Source:HGNC Symbol;Acc:HGNC:27031]	23.8	11.2	**-1.95**	0.003809
HIST1H2AD	histone cluster 1 H2A family member d [Source:HGNC Symbol;Acc:HGNC:4729]	13.4	5.6	**-1.95**	0.03965
NEGR1	neuronal growth regulator 1 [Source:HGNC Symbol;Acc:HGNC:17302]	2346.9	1199.2	**-1.96**	1.80E-62
AK1	adenylate kinase 1 [Source:HGNC Symbol;Acc:HGNC:361]	3624.4	1831.6	**-1.96**	2.40E-62
SEPT11	septin 11 [Source:HGNC Symbol;Acc:HGNC:25589]	9026.4	4559.3	**-1.96**	2.61E-45
HIF1A-AS1	HIF1A antisense RNA 1 [Source:HGNC Symbol;Acc:HGNC:43014]	451.2	229.7	**-1.96**	7.16E-33
CDKN3	cyclin dependent kinase inhibitor 3 [Source:HGNC Symbol;Acc:HGNC:1791]	409.1	214.2	**-1.96**	1.06E-25
TPX2	TPX2, microtubule nucleation factor [Source:HGNC Symbol;Acc:HGNC:1249]	1225.3	680.3	**-1.96**	1.81E-19
NCAPG	non-SMC condensin I complex subunit G [Source:HGNC Symbol;Acc:HGNC:24304]	1072.9	592.8	**-1.96**	3.63E-17
ADAMTS5	ADAM metallopeptidase with thrombospondin type 1 motif 5 [Source:HGNC Symbol;Acc:HGNC:221]	908.8	465.8	**-1.96**	4.51E-15
TACC3	transforming acidic coiled-coil containing protein 3 [Source:HGNC Symbol;Acc:HGNC:11524]	327.8	170.4	**-1.96**	6.04E-15
PRIM1	DNA primase subunit 1 [Source:HGNC Symbol;Acc:HGNC:9369]	357.8	189.8	**-1.96**	2.59E-14
MNS1	meiosis specific nuclear structural 1 [Source:HGNC Symbol;Acc:HGNC:29636]	253.0	129.7	**-1.96**	9.21E-14
GALNT17	polypeptide N-acetylgalactosaminyltransferase 17 [Source:HGNC Symbol;Acc:HGNC:16347]	163.5	82.2	**-1.96**	3.93E-12
SHISA2	shisa family member 2 [Source:HGNC Symbol;Acc:HGNC:20366]	187.6	96.5	**-1.96**	4.80E-12
MCM7	minichromosome maintenance complex component 7 [Source:HGNC Symbol;Acc:HGNC:6950]	1993.0	1053.8	**-1.96**	1.37E-11
ROGDI	rogdi homolog [Source:HGNC Symbol;Acc:HGNC:29478]	122.2	60.2	**-1.96**	1.46E-09
GADD45G	growth arrest and DNA damage inducible gamma [Source:HGNC Symbol;Acc:HGNC:4097]	22.8	10.7	**-1.96**	0.0007017
RASGEF1A	RasGEF domain family member 1A [Source:HGNC Symbol;Acc:HGNC:24246]	31.5	14.7	**-1.96**	0.001223
MYOM3	myomesin 3 [Source:HGNC Symbol;Acc:HGNC:26679]	19.8	9.1	**-1.96**	0.003876
KIF5A	kinesin family member 5A [Source:HGNC Symbol;Acc:HGNC:6323]	16.4	7.5	**-1.96**	0.005604
LINC01605	long intergenic non-protein coding RNA 1605 [Source:HGNC Symbol;Acc:HGNC:51654]	44.7	20.7	**-1.96**	0.005951
SNAI3	snail family transcriptional repressor 3 [Source:HGNC Symbol;Acc:HGNC:18411]	15.6	7.0	**-1.96**	0.009879
MEX3B	mex-3 RNA binding family member B [Source:HGNC Symbol;Acc:HGNC:25297]	11.4	5.0	**-1.96**	0.01847
RHBDL3	rhomboid like 3 [Source:HGNC Symbol;Acc:HGNC:16502]	11.6	4.8	**-1.96**	0.02484
SLC8A1	solute carrier family 8 member A1 [Source:HGNC Symbol;Acc:HGNC:11068]	2257.5	1147.7	**-1.97**	2.89E-61
UNC5C	unc-5 netrin receptor C [Source:HGNC Symbol;Acc:HGNC:12569]	982.9	494.8	**-1.97**	1.39E-60
ITGB8	integrin subunit beta 8 [Source:HGNC Symbol;Acc:HGNC:6163]	13901.7	7024.7	**-1.97**	2.71E-35
CERCAM	cerebral endothelial cell adhesion molecule [Source:HGNC Symbol;Acc:HGNC:23723]	769.5	386.8	**-1.97**	3.45E-30
NOVA1	NOVA alternative splicing regulator 1 [Source:HGNC Symbol;Acc:HGNC:7886]	615.0	308.6	**-1.97**	9.21E-30
HES1	hes family bHLH transcription factor 1 [Source:HGNC Symbol;Acc:HGNC:5192]	338.2	170.7	**-1.97**	7.54E-24
CRYZL2P-SEC16B	CRYZL2P-SEC16B readthrough [Source:HGNC Symbol;Acc:HGNC:53757]	304.7	151.7	**-1.97**	3.35E-22
SPAG5	sperm associated antigen 5 [Source:HGNC Symbol;Acc:HGNC:13452]	686.0	365.3	**-1.97**	2.04E-20
MELK	maternal embryonic leucine zipper kinase [Source:HGNC Symbol;Acc:HGNC:16870]	598.3	329.6	**-1.97**	1.74E-14
NR3C2	nuclear receptor subfamily 3 group C member 2 [Source:HGNC Symbol;Acc:HGNC:7979]	230.2	112.4	**-1.97**	2.10E-14
NUF2	NUF2, NDC80 kinetochore complex component [Source:HGNC Symbol;Acc:HGNC:14621]	514.2	278.0	**-1.97**	2.58E-13
LRRN4CL	LRRN4 C-terminal like [Source:HGNC Symbol;Acc:HGNC:33724]	129.6	64.9	**-1.97**	8.21E-12
TM4SF1	transmembrane 4 L six family member 1 [Source:HGNC Symbol;Acc:HGNC:11853]	13823.5	7276.6	**-1.97**	1.64E-11
P2RY2	purinergic receptor P2Y2 [Source:HGNC Symbol;Acc:HGNC:8541]	88.0	43.8	**-1.97**	2.43E-10
PITPNM3	PITPNM family member 3 [Source:HGNC Symbol;Acc:HGNC:21043]	69.8	35.0	**-1.97**	7.33E-06
TROAP	trophinin associated protein [Source:HGNC Symbol;Acc:HGNC:12327]	54.2	27.3	**-1.97**	8.97E-05
HSF5	heat shock transcription factor 5 [Source:HGNC Symbol;Acc:HGNC:26862]	24.8	11.5	**-1.97**	0.001671
CASP12	caspase 12 (gene/pseudogene) [Source:HGNC Symbol;Acc:HGNC:19004]	31.3	14.7	**-1.97**	0.003248
FER1L4	fer-1 like family member 4, pseudogene [Source:HGNC Symbol;Acc:HGNC:15801]	15.9	7.2	**-1.97**	0.005286
ROCR	regulator of chondrogenesis RNA [Source:HGNC Symbol;Acc:HGNC:52946]	26.6	11.7	**-1.97**	0.00821
SNORD114-10	small nucleolar RNA, C/D box 114-10 [Source:HGNC Symbol;Acc:HGNC:32998]	15.6	6.7	**-1.97**	0.008224
LINC01106	long intergenic non-protein coding RNA 1106 [Source:HGNC Symbol;Acc:HGNC:26769]	13.1	5.7	**-1.97**	0.01396
MYH16	myosin heavy chain 16 pseudogene [Source:HGNC Symbol;Acc:HGNC:31038]	12.4	5.2	**-1.97**	0.02493
AC240274.1	neuroblastoma breakpoint family member 1 [Source:NCBI gene;Acc:102724250]	330.7	165.6	**-1.99**	6.70E-35
CHI3L1	chitinase 3 like 1 [Source:HGNC Symbol;Acc:HGNC:1932]	43725.4	22629.6	**-1.99**	6.16E-20
CCDC15	coiled-coil domain containing 15 [Source:HGNC Symbol;Acc:HGNC:25798]	208.3	107.4	**-1.99**	3.06E-13
UBE2T	ubiquitin conjugating enzyme E2 T [Source:HGNC Symbol;Acc:HGNC:25009]	352.9	184.9	**-1.99**	5.17E-13
RAD54L	RAD54 like [Source:HGNC Symbol;Acc:HGNC:9826]	90.8	45.5	**-1.99**	1.22E-09
RNF112	ring finger protein 112 [Source:HGNC Symbol;Acc:HGNC:12968]	45.1	22.1	**-1.99**	1.73E-06
SPSB4	splA/ryanodine receptor domain and SOCS box containing 4 [Source:HGNC Symbol;Acc:HGNC:30630]	60.6	28.9	**-1.99**	9.42E-06
ARMC4	armadillo repeat containing 4 [Source:HGNC Symbol;Acc:HGNC:25583]	54.8	26.5	**-1.99**	1.20E-05
OLFML3	olfactomedin like 3 [Source:HGNC Symbol;Acc:HGNC:24956]	72.8	36.0	**-1.99**	1.96E-05
GJB2	gap junction protein beta 2 [Source:HGNC Symbol;Acc:HGNC:4284]	94.7	45.1	**-1.99**	6.73E-05
LRP4-AS1	LRP4 antisense RNA 1 [Source:HGNC Symbol;Acc:HGNC:44128]	32.2	15.1	**-1.99**	0.0001599
DCLK2	doublecortin like kinase 2 [Source:HGNC Symbol;Acc:HGNC:19002]	37.5	17.8	**-1.99**	0.0002881
P2RX7	purinergic receptor P2X 7 [Source:HGNC Symbol;Acc:HGNC:8537]	24.2	11.1	**-1.99**	0.0009392
SLC18B1	solute carrier family 18 member B1 [Source:HGNC Symbol;Acc:HGNC:21573]	19.4	8.9	**-1.99**	0.002807
SIGLEC15	sialic acid binding Ig like lectin 15 [Source:HGNC Symbol;Acc:HGNC:27596]	15.0	5.8	**-1.99**	0.03045
SOX5	SRY-box 5 [Source:HGNC Symbol;Acc:HGNC:11201]	10957.8	5498.5	**-2.00**	5.09E-88
KIF13B	kinesin family member 13B [Source:HGNC Symbol;Acc:HGNC:14405]	4531.7	2252.9	**-2.00**	9.15E-50
FAM180A	family with sequence similarity 180 member A [Source:HGNC Symbol;Acc:HGNC:33773]	738.2	376.0	**-2.00**	2.15E-38
COL8A2	collagen type VIII alpha 2 chain [Source:HGNC Symbol;Acc:HGNC:2216]	1254.8	622.0	**-2.00**	1.19E-36
B3GNT9	UDP-GlcNAc:betaGal beta-1,3-N-acetylglucosaminyltransferase 9 [Source:HGNC Symbol;Acc:HGNC:2871	701.0	347.1	**-2.00**	3.17E-36
CAMK1D	calcium/calmodulin dependent protein kinase ID [Source:HGNC Symbol;Acc:HGNC:19341]	574.5	288.0	**-2.00**	3.04E-30

GLRB	glycine receptor beta [Source:HGNC Symbol;Acc:HGNC:4329]	174.3	86.0	**-2.00**	9.50E-15
PAQR4	progestin and adipoQ receptor family member 4 [Source:HGNC Symbol;Acc:HGNC:26386]	190.5	98.4	**-2.00**	1.38E-14
HIST1H2BN	histone cluster 1 H2B family member n [Source:HGNC Symbol;Acc:HGNC:4749]	210.6	108.4	**-2.00**	2.97E-14
RAB38	RAB38, member RAS oncogene family [Source:HGNC Symbol;Acc:HGNC:9776]	187.9	94.7	**-2.00**	2.01E-11
HIST1H3H	histone cluster 1 H3 family member h [Source:HGNC Symbol;Acc:HGNC:4775]	99.9	50.4	**-2.00**	6.59E-10
SPON2	spondin 2 [Source:HGNC Symbol;Acc:HGNC:11253]	79.7	38.6	**-2.00**	1.05E-07
SYNGR1	synaptogyrin 1 [Source:HGNC Symbol;Acc:HGNC:11498]	63.7	30.1	**-2.00**	3.84E-05
CPEB1	cytoplasmic polyadenylation element binding protein 1 [Source:HGNC Symbol;Acc:HGNC:21744]	29.0	13.4	**-2.00**	0.0003883
LYPD5	LY6/PLAUR domain containing 5 [Source:HGNC Symbol;Acc:HGNC:26397]	34.8	16.9	**-2.00**	0.001385
PXDNL	peroxidasin like [Source:HGNC Symbol;Acc:HGNC:26359]	25.9	11.4	**-2.00**	0.001982
SCN2A	sodium voltage-gated channel alpha subunit 2 [Source:HGNC Symbol;Acc:HGNC:10588]	25.5	11.5	**-2.00**	0.003757
SV2B	synaptic vesicle glycoprotein 2B [Source:HGNC Symbol;Acc:HGNC:16874]	40.0	18.1	**-2.00**	0.004945
LMNTD2	lamin tail domain containing 2 [Source:HGNC Symbol;Acc:HGNC:28561]	7.4	2.7	**-2.00**	0.0395
PDLIM7	PDZ and LIM domain 7 [Source:HGNC Symbol;Acc:HGNC:22958]	356.4	179.6	**-2.01**	9.91E-25
SLC5A3	solute carrier family 5 member 3 [Source:HGNC Symbol;Acc:HGNC:11038]	21780.2	10505.7	**-2.01**	8.67E-21
ADAMTSL2	ADAMTS like 2 [Source:HGNC Symbol;Acc:HGNC:14631]	270.6	134.8	**-2.01**	8.83E-15
FANCG	Fanconi anemia complementation group G [Source:HGNC Symbol;Acc:HGNC:3588]	161.2	80.1	**-2.01**	1.20E-13
USP51	ubiquitin specific peptidase 51 [Source:HGNC Symbol;Acc:HGNC:23086]	171.2	84.1	**-2.01**	4.69E-13
RHOJ	ras homolog family member J [Source:HGNC Symbol;Acc:HGNC:688]	111.8	54.8	**-2.01**	1.34E-11
STAB1	stabilin 1 [Source:HGNC Symbol;Acc:HGNC:18628]	109.5	52.7	**-2.01**	4.33E-11
RMI2	RecQ mediated genome instability 2 [Source:HGNC Symbol;Acc:HGNC:28349]	137.7	70.0	**-2.01**	4.62E-08
MMRN2	multimerin 2 [Source:HGNC Symbol;Acc:HGNC:19888]	100.8	49.3	**-2.01**	3.97E-07
SGIP1	SH3 domain GRB2 like endophilin interacting protein 1 [Source:HGNC Symbol;Acc:HGNC:25412]	61.6	29.5	**-2.01**	7.48E-07
CDKN1C	cyclin dependent kinase inhibitor 1C [Source:HGNC Symbol;Acc:HGNC:1786]	65.7	30.6	**-2.01**	3.99E-05
FABP5P7	fatty acid binding protein 5 pseudogene 7 [Source:HGNC Symbol;Acc:HGNC:31070]	43.1	19.9	**-2.01**	6.39E-05
TRIM22	tripartite motif containing 22 [Source:HGNC Symbol;Acc:HGNC:16379]	37.1	17.0	**-2.01**	0.0005145
CLIC3	chloride intracellular channel 3 [Source:HGNC Symbol;Acc:HGNC:2064]	32.9	15.7	**-2.01**	0.00207
JAKMIP3	Janus kinase and microtubule interacting protein 3 [Source:HGNC Symbol;Acc:HGNC:23523]	16.1	6.9	**-2.01**	0.004573
EPN3	epsin 3 [Source:HGNC Symbol;Acc:HGNC:18235]	9.7	3.8	**-2.01**	0.02902
LRP12	LDL receptor related protein 12 [Source:HGNC Symbol;Acc:HGNC:31708]	1796.3	881.7	**-2.03**	1.26E-82
TNFRSF10B	TNF receptor superfamily member 10b [Source:HGNC Symbol;Acc:HGNC:11905]	1039.0	508.6	**-2.03**	1.48E-70
KAZALD1	Kazal type serine peptidase inhibitor domain 1 [Source:HGNC Symbol;Acc:HGNC:25460]	492.4	243.6	**-2.03**	1.57E-46
FAM111A	family with sequence similarity 111 member A [Source:HGNC Symbol;Acc:HGNC:24725]	1458.1	737.4	**-2.03**	5.11E-42
SDK2	sidekick cell adhesion molecule 2 [Source:HGNC Symbol;Acc:HGNC:19308]	1850.6	908.6	**-2.03**	1.14E-34
PUS10	pseudouridylate synthase 10 [Source:HGNC Symbol;Acc:HGNC:26505]	636.3	308.7	**-2.03**	1.81E-34
DNM1	dynamin 1 [Source:HGNC Symbol;Acc:HGNC:2972]	229.3	111.9	**-2.03**	2.86E-24
BANK1	B cell scaffold protein with ankyrin repeats 1 [Source:HGNC Symbol;Acc:HGNC:18233]	526.3	271.3	**-2.03**	6.26E-19
CDK1	cyclin dependent kinase 1 [Source:HGNC Symbol;Acc:HGNC:1722]	993.2	530.5	**-2.03**	1.40E-17
PKMYT1	protein kinase, membrane associated tyrosine/threonine 1 [Source:HGNC Symbol;Acc:HGNC:29650]	162.4	83.7	**-2.03**	2.06E-11
RAPGEF3	Rap guanine nucleotide exchange factor 3 [Source:HGNC Symbol;Acc:HGNC:16629]	85.8	41.3	**-2.03**	3.39E-10
HASPIN	histone H3 associated protein kinase [Source:HGNC Symbol;Acc:HGNC:19682]	64.7	32.9	**-2.03**	5.88E-07
CSRNP3	cysteine and serine rich nuclear protein 3 [Source:HGNC Symbol;Acc:HGNC:30729]	76.2	34.3	**-2.03**	5.37E-06
ABCA12	ATP binding cassette subfamily A member 12 [Source:HGNC Symbol;Acc:HGNC:14637]	23.4	10.4	**-2.03**	0.001627
KCTD16	potassium channel tetramerization domain containing 16 [Source:HGNC Symbol;Acc:HGNC:29244]	22.5	9.6	**-2.03**	0.006484
PPP1R26P1	protein phosphatase 1 regulatory subunit 26 pseudogene 1 [Source:HGNC Symbol;Acc:HGNC:42015]	7.9	2.8	**-2.03**	0.03077
B3GALT1	beta-1,3-galactosyltransferase 1 [Source:HGNC Symbol;Acc:HGNC:916]	173.0	84.6	**-2.04**	1.42E-18
BIRC5	baculoviral IAP repeat containing 5 [Source:HGNC Symbol;Acc:HGNC:593]	427.4	224.2	**-2.04**	1.38E-14
RAD51	RAD51 recombinase [Source:HGNC Symbol;Acc:HGNC:9817]	181.7	90.6	**-2.04**	2.26E-13
SNAI1	snail family transcriptional repressor 1 [Source:HGNC Symbol;Acc:HGNC:11128]	101.4	48.4	**-2.04**	1.32E-12
INHBA-AS1	INHBA antisense RNA 1 [Source:HGNC Symbol;Acc:HGNC:40303]	156.9	79.5	**-2.04**	3.03E-11
PNMA8A	PNMA family member 8A [Source:HGNC Symbol;Acc:HGNC:25578]	114.6	54.2	**-2.04**	1.45E-08
SOCS2-AS1	SOCS2 antisense RNA 1 [Source:HGNC Symbol;Acc:HGNC:27054]	85.1	39.9	**-2.04**	1.53E-07
PKDCC	protein kinase domain containing, cytoplasmic [Source:HGNC Symbol;Acc:HGNC:25123]	91.9	44.3	**-2.04**	1.69E-07
MPP2	membrane palmitoylated protein 2 [Source:HGNC Symbol;Acc:HGNC:7220]	48.5	23.1	**-2.04**	7.72E-07
SLC2A5	solute carrier family 2 member 5 [Source:HGNC Symbol;Acc:HGNC:11010]	44.4	21.2	**-2.04**	1.66E-05
CA9	carbonic anhydrase 9 [Source:HGNC Symbol;Acc:HGNC:1383]	27.0	11.7	**-2.04**	0.001632
PPFIA3	PTPRF interacting protein alpha 3 [Source:HGNC Symbol;Acc:HGNC:9247]	25.7	11.2	**-2.04**	0.002688
C15orf59	chromosome 15 open reading frame 59 [Source:HGNC Symbol;Acc:HGNC:33753]	12.5	5.2	**-2.04**	0.005305
CYSRT1	cysteine rich tail 1 [Source:HGNC Symbol;Acc:HGNC:30529]	11.0	4.1	**-2.04**	0.02038
PXYLP1	2-phosphoxylose phosphatase 1 [Source:HGNC Symbol;Acc:HGNC:26303]	2873.2	1387.0	**-2.06**	1.85E-55
MLLT3	MLLT3, super elongation complex subunit [Source:HGNC Symbol;Acc:HGNC:7136]	257.5	124.1	**-2.06**	7.15E-21
MALINC1	mitosis associated long intergenic non-coding RNA 1 [Source:HGNC Symbol;Acc:HGNC:49009]	129.1	61.6	**-2.06**	7.56E-14
HIST1H2AK	histone cluster 1 H2A family member k [Source:HGNC Symbol;Acc:HGNC:4726]	65.0	31.2	**-2.06**	5.23E-07
ADAM19	ADAM metallopeptidase domain 19 [Source:HGNC Symbol;Acc:HGNC:197]	54.4	25.0	**-2.06**	1.55E-06
PBK	PDZ binding kinase [Source:HGNC Symbol;Acc:HGNC:18282]	945.2	486.5	**-2.06**	3.29E-06
AGRN	agrin [Source:HGNC Symbol;Acc:HGNC:329]	52.0	24.2	**-2.06**	6.53E-06
SAPCD2	suppressor APC domain containing 2 [Source:HGNC Symbol;Acc:HGNC:28055]	34.9	15.5	**-2.06**	5.01E-05
ALX4	ALX homeobox 4 [Source:HGNC Symbol;Acc:HGNC:450]	33.4	15.1	**-2.06**	9.43E-05
MAMLD1	mastermind like domain containing 1 [Source:HGNC Symbol;Acc:HGNC:2568]	37.0	17.5	**-2.06**	0.0001045
DMGDH	dimethylglycine dehydrogenase [Source:HGNC Symbol;Acc:HGNC:24475]	23.2	10.0	**-2.06**	0.001814
NRGN	neurogranin [Source:HGNC Symbol;Acc:HGNC:8000]	13.0	4.5	**-2.06**	0.01967
IGSF5	immunoglobulin superfamily member 5 [Source:HGNC Symbol;Acc:HGNC:5952]	7.5	2.6	**-2.06**	0.02983

COLGALT2	collagen beta(1-O)galactosyltransferase 2 [Source:HGNC Symbol;Acc:HGNC:16790]	4016.7	1938.1	**-2.07**	2.94E-91
SEC16B	SEC16 homolog B, endoplasmic reticulum export factor [Source:HGNC Symbol;Acc:HGNC:30301]	266.5	126.6	**-2.07**	1.48E-21
BUB1B	BUB1 mitotic checkpoint serine/threonine kinase B [Source:HGNC Symbol;Acc:HGNC:1149]	535.5	270.3	**-2.07**	9.82E-19
OBSCN	obscurin, cytoskeletal calmodulin and titin-interacting RhoGEF [Source:HGNC Symbol;Acc:HGNC:15719]	235.0	111.8	**-2.07**	1.46E-16
PPM1H	protein phosphatase, Mg2+/Mn2+ dependent 1H [Source:HGNC Symbol;Acc:HGNC:18583]	114.1	53.5	**-2.07**	2.13E-13
DLGAP1-AS2	DLGAP1 antisense RNA 2 [Source:HGNC Symbol;Acc:HGNC:28146]	72.4	33.4	**-2.07**	2.53E-09
GSTM5	glutathione S-transferase mu 5 [Source:HGNC Symbol;Acc:HGNC:4637]	78.2	35.8	**-2.07**	2.87E-09
BHLHE40-AS1	BHLHE40 antisense RNA 1 [Source:HGNC Symbol;Acc:HGNC:44471]	82.4	38.6	**-2.07**	7.44E-09
TMEM196	transmembrane protein 196 [Source:HGNC Symbol;Acc:HGNC:22431]	100.5	46.6	**-2.07**	1.94E-07
NEIL3	nei like DNA glycosylase 3 [Source:HGNC Symbol;Acc:HGNC:24573]	77.4	38.1	**-2.07**	4.02E-07
EBF3	early B cell factor 3 [Source:HGNC Symbol;Acc:HGNC:19087]	23.4	10.3	**-2.07**	0.0001952
EREG	epiregulin [Source:HGNC Symbol;Acc:HGNC:3443]	34.7	15.4	**-2.07**	0.0003848
RNF150	ring finger protein 150 [Source:HGNC Symbol;Acc:HGNC:23138]	22.5	9.6	**-2.07**	0.004403
ZFHX4-AS1	ZFHX4 antisense RNA 1 [Source:HGNC Symbol;Acc:HGNC:44165]	21.4	8.8	**-2.07**	0.004904
CARNS1	carnosine synthase 1 [Source:HGNC Symbol;Acc:HGNC:29268]	16.9	7.1	**-2.07**	0.005508
NPNT	nephronectin [Source:HGNC Symbol;Acc:HGNC:27405]	13.3	5.1	**-2.07**	0.0129
RPL29P19	ribosomal protein L29 pseudogene 19 [Source:HGNC Symbol;Acc:HGNC:35508]	11.0	4.2	**-2.07**	0.01326
B4GALNT3	beta-1,4-N-acetyl-galactosaminyltransferase 3 [Source:HGNC Symbol;Acc:HGNC:24137]	1655.8	795.5	**-2.08**	1.53E-105
CXXC5	CXXC finger protein 5 [Source:HGNC Symbol;Acc:HGNC:26943]	674.5	321.9	**-2.08**	1.27E-38
MPP7	membrane palmitoylated protein 7 [Source:HGNC Symbol;Acc:HGNC:26542]	424.1	202.2	**-2.08**	2.45E-38
NCAM1	neural cell adhesion molecule 1 [Source:HGNC Symbol;Acc:HGNC:7656]	367.3	175.7	**-2.08**	4.80E-21
FERMT1	fermitin family member 1 [Source:HGNC Symbol;Acc:HGNC:15889]	299.6	136.5	**-2.08**	5.80E-21
GINS2	GINS complex subunit 2 [Source:HGNC Symbol;Acc:HGNC:24575]	232.5	116.1	**-2.08**	7.54E-11
CDC6	cell division cycle 6 [Source:HGNC Symbol;Acc:HGNC:1744]	948.5	470.1	**-2.08**	1.47E-10
FRMPD4	FERM and PDZ domain containing 4 [Source:HGNC Symbol;Acc:HGNC:29007]	86.0	39.4	**-2.08**	5.03E-10
CENPK	centromere protein K [Source:HGNC Symbol;Acc:HGNC:29479]	583.2	289.6	**-2.08**	1.20E-08
GDF5OS	growth differentiation factor 5 opposite strand [Source:HGNC Symbol;Acc:HGNC:33435]	122.8	59.6	**-2.08**	9.32E-08
MGAT3	mannosyl (beta-1,4-)-glycoprotein beta-1,4-N-acetylglucosaminyltransferase [Source:HGNC Symbol;Acc:	71.1	30.8	**-2.08**	2.73E-06
BBC3	BCL2 binding component 3 [Source:HGNC Symbol;Acc:HGNC:17868]	34.1	15.4	**-2.08**	3.84E-05
KLLN	killin, p53 regulated DNA replication inhibitor [Source:HGNC Symbol;Acc:HGNC:37212]	32.5	14.5	**-2.08**	0.0001421
ZNF887P	zinc finger protein 887, pseudogene [Source:HGNC Symbol;Acc:HGNC:38700]	19.3	8.3	**-2.08**	0.002115
BCL6B	B cell CLL/lymphoma 6B [Source:HGNC Symbol;Acc:HGNC:1002]	8.0	2.5	**-2.08**	0.03056
TCEA3	transcription elongation factor A3 [Source:HGNC Symbol;Acc:HGNC:11615]	1303.2	620.8	**-2.10**	1.85E-47
EPB41L1	erythrocyte membrane protein band 4.1 like 1 [Source:HGNC Symbol;Acc:HGNC:3378]	529.5	251.3	**-2.10**	2.31E-40
TRO	trophinin [Source:HGNC Symbol;Acc:HGNC:12326]	274.0	127.9	**-2.10**	8.63E-30
DLG3	discs large MAGUK scaffold protein 3 [Source:HGNC Symbol;Acc:HGNC:2902]	353.0	168.0	**-2.10**	1.15E-24
ARHGAP5-AS1	ARHGAP5 antisense RNA 1 (head to head) [Source:HGNC Symbol;Acc:HGNC:20279]	113.7	53.6	**-2.10**	1.75E-16
HIST1H2BE	histone cluster 1 H2B family member e [Source:HGNC Symbol;Acc:HGNC:4753]	194.6	97.3	**-2.10**	5.15E-14
NHS	NHS actin remodeling regulator [Source:HGNC Symbol;Acc:HGNC:7820]	175.1	81.1	**-2.10**	4.19E-13
IGFBP4	insulin like growth factor binding protein 4 [Source:HGNC Symbol;Acc:HGNC:5473]	193.8	92.1	**-2.10**	5.61E-12
DOC2B	double C2 domain beta [Source:HGNC Symbol;Acc:HGNC:2986]	86.1	39.5	**-2.10**	1.17E-09
BAHCC1	BAH domain and coiled-coil containing 1 [Source:HGNC Symbol;Acc:HGNC:29279]	84.9	40.4	**-2.10**	2.99E-08
WNT3	Wnt family member 3 [Source:HGNC Symbol;Acc:HGNC:12782]	42.5	19.1	**-2.10**	4.05E-05
RDH10-AS1	RDH10 antisense RNA 1 [Source:HGNC Symbol;Acc:HGNC:51658]	23.8	9.9	**-2.10**	0.003783
HAO1	hydroxyacid oxidase 1 [Source:HGNC Symbol;Acc:HGNC:4809]	16.6	6.6	**-2.10**	0.003809
IQSEC3	IQ motif and Sec7 domain 3 [Source:HGNC Symbol;Acc:HGNC:29193]	9.8	3.7	**-2.10**	0.01348
AC129507.1	uncharacterized LOC100506388 [Source:NCBI gene;Acc:100506388]	12.7	4.6	**-2.10**	0.01755
SCARA3	scavenger receptor class A member 3 [Source:HGNC Symbol;Acc:HGNC:19000]	5025.5	2373.6	**-2.11**	4.80E-83
CAB39L	calcium binding protein 39 like [Source:HGNC Symbol;Acc:HGNC:20290]	1461.9	694.6	**-2.11**	1.53E-60
SERPINH1	serpin family H member 1 [Source:HGNC Symbol;Acc:HGNC:1546]	2782.5	1311.0	**-2.11**	1.77E-55
COL1A2	collagen type I alpha 2 chain [Source:HGNC Symbol;Acc:HGNC:2198]	5477.4	2601.9	**-2.11**	3.08E-53
PLEKHG3	pleckstrin homology and RhoGEF domain containing G3 [Source:HGNC Symbol;Acc:HGNC:20364]	410.4	192.1	**-2.11**	8.39E-34
DUSP10	dual specificity phosphatase 10 [Source:HGNC Symbol;Acc:HGNC:3065]	197.6	91.4	**-2.11**	3.51E-21
PTGS1	prostaglandin-endoperoxide synthase 1 [Source:HGNC Symbol;Acc:HGNC:9604]	492.6	242.9	**-2.11**	1.03E-18
GDF5	growth differentiation factor 5 [Source:HGNC Symbol;Acc:HGNC:4220]	474.1	229.3	**-2.11**	2.93E-17
MAD2L1	mitotic arrest deficient 2 like 1 [Source:HGNC Symbol;Acc:HGNC:6763]	536.0	268.6	**-2.11**	5.47E-17
CNTNAP1	contactin associated protein 1 [Source:HGNC Symbol;Acc:HGNC:8011]	139.2	64.5	**-2.11**	4.70E-13
ATP8B4	ATPase phospholipid transporting 8B4 (putative) [Source:HGNC Symbol;Acc:HGNC:13536]	115.8	51.8	**-2.11**	2.46E-09
KIF18B	kinesin family member 18B [Source:HGNC Symbol;Acc:HGNC:27102]	70.7	33.0	**-2.11**	8.06E-08
FAM155A	family with sequence similarity 155 member A [Source:HGNC Symbol;Acc:HGNC:33877]	59.1	26.9	**-2.11**	1.74E-06
FDXR	ferredoxin reductase [Source:HGNC Symbol;Acc:HGNC:3642]	46.3	20.7	**-2.11**	1.58E-05
SYT16	synaptotagmin 16 [Source:HGNC Symbol;Acc:HGNC:23142]	38.9	17.5	**-2.11**	1.81E-05
SRPK3	SRSF protein kinase 3 [Source:HGNC Symbol;Acc:HGNC:11402]	41.7	18.8	**-2.11**	2.58E-05
LINC00601	long intergenic non-protein coding RNA 601 [Source:HGNC Symbol;Acc:HGNC:43916]	10.0	3.8	**-2.11**	0.01012
LRP4	LDL receptor related protein 4 [Source:HGNC Symbol;Acc:HGNC:6696]	508.8	239.7	**-2.13**	1.10E-44
ADTRP	androgen dependent TFPI regulating protein [Source:HGNC Symbol;Acc:HGNC:21214]	651.0	304.0	**-2.13**	2.50E-27
FABP5	fatty acid binding protein 5 [Source:HGNC Symbol;Acc:HGNC:3560]	1357.8	634.5	**-2.13**	3.29E-27
ADORA1	adenosine A1 receptor [Source:HGNC Symbol;Acc:HGNC:262]	289.0	135.1	**-2.13**	7.71E-22
CENPU	centromere protein U [Source:HGNC Symbol;Acc:HGNC:21348]	886.9	450.8	**-2.13**	6.68E-21
LINC01915	long intergenic non-protein coding RNA 1915 [Source:HGNC Symbol;Acc:HGNC:52734]	85.0	38.3	**-2.13**	5.06E-08
GSC	goosecoid homeobox [Source:HGNC Symbol;Acc:HGNC:4612]	32.7	14.5	**-2.13**	7.09E-05
MMEL1	membrane metalloendopeptidase like 1 [Source:HGNC Symbol;Acc:HGNC:14668]	17.5	7.2	**-2.13**	0.001628

LINC01579	long intergenic non-protein coding RNA 1579 [Source:HGNC Symbol;Acc:HGNC:27519]	10.2	3.6	**-2.13**	0.01101
LINC01770	long intergenic non-protein coding RNA 1770 [Source:HGNC Symbol;Acc:HGNC:52560]	8.1	2.6	**-2.13**	0.02342
ICAM1	intercellular adhesion molecule 1 [Source:HGNC Symbol;Acc:HGNC:5344]	1590.9	782.4	**-2.14**	8.37E-44
BAALC	BAALC, MAP3K1 and KLF4 binding [Source:HGNC Symbol;Acc:HGNC:14333]	1038.9	488.8	**-2.14**	3.72E-30
HIST1H2BJ	histone cluster 1 H2B family member j [Source:HGNC Symbol;Acc:HGNC:4761]	974.5	468.8	**-2.14**	6.63E-25
ATAD2	ATPase family, AAA domain containing 2 [Source:HGNC Symbol;Acc:HGNC:30123]	2122.2	1037.7	**-2.14**	3.05E-12
PIP5KL1	phosphatidylinositol-4-phosphate 5-kinase like 1 [Source:HGNC Symbol;Acc:HGNC:28711]	69.5	31.4	**-2.14**	1.57E-08
TMEM200A	transmembrane protein 200A [Source:HGNC Symbol;Acc:HGNC:21075]	91.0	41.6	**-2.14**	4.02E-08
WDR62	WD repeat domain 62 [Source:HGNC Symbol;Acc:HGNC:24502]	120.1	57.1	**-2.14**	5.15E-08
TMC6	transmembrane channel like 6 [Source:HGNC Symbol;Acc:HGNC:18021]	25.2	10.6	**-2.14**	0.0005084
TSPAN6	tetraspanin 6 [Source:HGNC Symbol;Acc:HGNC:11858]	3335.7	1542.6	**-2.16**	9.25E-132
LRIG3	leucine rich repeats and immunoglobulin like domains 3 [Source:HGNC Symbol;Acc:HGNC:30991]	1910.8	866.5	**-2.16**	8.56E-49
TGFB2-AS1	TGFB2 antisense RNA 1 (head to head) [Source:HGNC Symbol;Acc:HGNC:50628]	231.0	105.2	**-2.16**	1.58E-19
COL10A1	collagen type X alpha 1 chain [Source:HGNC Symbol;Acc:HGNC:2185]	2211.9	1059.0	**-2.16**	1.80E-11
LMNB1	lamin B1 [Source:HGNC Symbol;Acc:HGNC:6637]	883.8	441.3	**-2.16**	9.18E-11
SCG5	secretogranin V [Source:HGNC Symbol;Acc:HGNC:10816]	116.7	52.5	**-2.16**	5.25E-10
REEP1	receptor accessory protein 1 [Source:HGNC Symbol;Acc:HGNC:25786]	48.6	21.4	**-2.16**	1.02E-07
MND1	meiotic nuclear divisions 1 [Source:HGNC Symbol;Acc:HGNC:24839]	104.9	48.5	**-2.16**	6.65E-07
SERPINA6	serpin family A member 6 [Source:HGNC Symbol;Acc:HGNC:1540]	39.8	17.2	**-2.16**	1.12E-06
NUDT8	nudix hydrolase 8 [Source:HGNC Symbol;Acc:HGNC:8055]	20.6	8.6	**-2.16**	0.0004345
LINGO1	leucine rich repeat and Ig domain containing 1 [Source:HGNC Symbol;Acc:HGNC:21205]	30.2	12.3	**-2.16**	0.00299
ST6GAL2	ST6 beta-galactoside alpha-2,6-sialyltransferase 2 [Source:HGNC Symbol;Acc:HGNC:10861]	16.8	6.5	**-2.16**	0.006115
PGK1P2	phosphoglycerate kinase 1, pseudogene 2 [Source:HGNC Symbol;Acc:HGNC:8899]	8.0	2.7	**-2.16**	0.01608
HPDL	4-hydroxyphenylpyruvate dioxygenase like [Source:HGNC Symbol;Acc:HGNC:28242]	12.7	3.8	**-2.16**	0.02356
SEPT5	septin 5 [Source:HGNC Symbol;Acc:HGNC:9164]	1415.3	640.8	**-2.17**	3.18E-74
ENO2	enolase 2 [Source:HGNC Symbol;Acc:HGNC:3353]	722.7	329.5	**-2.17**	4.27E-57
BRCA2	BRCA2, DNA repair associated [Source:HGNC Symbol;Acc:HGNC:1101]	969.5	471.5	**-2.17**	1.32E-25
ETV1	ETS variant 1 [Source:HGNC Symbol;Acc:HGNC:3490]	562.4	254.4	**-2.17**	6.95E-25
PLCG2	phospholipase C gamma 2 [Source:HGNC Symbol;Acc:HGNC:9066]	351.0	158.5	**-2.17**	1.15E-21
FAM213B	family with sequence similarity 213 member B [Source:HGNC Symbol;Acc:HGNC:28390]	101.5	45.8	**-2.17**	4.09E-12
CKAP2L	cytoskeleton associated protein 2 like [Source:HGNC Symbol;Acc:HGNC:26877]	615.7	295.8	**-2.17**	1.15E-09
ORC1	origin recognition complex subunit 1 [Source:HGNC Symbol;Acc:HGNC:8487]	181.5	87.6	**-2.17**	3.62E-09
AURKB	aurora kinase B [Source:HGNC Symbol;Acc:HGNC:11390]	122.1	58.6	**-2.17**	2.38E-07
LINC00900	long intergenic non-protein coding RNA 900 [Source:HGNC Symbol;Acc:HGNC:27444]	22.4	9.3	**-2.17**	0.0006381
VWA1	von Willebrand factor A domain containing 1 [Source:HGNC Symbol;Acc:HGNC:30910]	618.8	282.0	**-2.19**	7.84E-65
DTWD1	DTW domain containing 1 [Source:HGNC Symbol;Acc:HGNC:30926]	1591.0	717.5	**-2.19**	1.02E-40
CABLES1	Cdk5 and Abl enzyme substrate 1 [Source:HGNC Symbol;Acc:HGNC:25097]	305.9	137.3	**-2.19**	5.10E-26
DNAJC22	DnaJ heat shock protein family (Hsp40) member C22 [Source:HGNC Symbol;Acc:HGNC:25802]	201.1	90.9	**-2.19**	7.80E-17
TGFBI	transforming growth factor beta induced [Source:HGNC Symbol;Acc:HGNC:11771]	3222.9	1495.3	**-2.19**	3.63E-16
SKA1	spindle and kinetochore associated complex subunit 1 [Source:HGNC Symbol;Acc:HGNC:28109]	195.5	91.0	**-2.19**	3.11E-15
NT5DC4	5'-nucleotidase domain containing 4 [Source:HGNC Symbol;Acc:HGNC:27678]	69.3	32.0	**-2.19**	2.08E-07
KIF21B	kinesin family member 21B [Source:HGNC Symbol;Acc:HGNC:29442]	32.4	13.8	**-2.19**	6.95E-06
RASL12	RAS like family 12 [Source:HGNC Symbol;Acc:HGNC:30289]	24.2	10.3	**-2.19**	0.0001306
MEG9	maternally expressed 9 (non-protein coding) [Source:HGNC Symbol;Acc:HGNC:43874]	19.6	7.8	**-2.19**	0.002344
UROC1	urocanate hydratase 1 [Source:HGNC Symbol;Acc:HGNC:26444]	14.3	5.2	**-2.19**	0.004527
FMNL1	formin like 1 [Source:HGNC Symbol;Acc:HGNC:1212]	9.6	3.2	**-2.19**	0.01173
SLC8A1-AS1	SLC8A1 antisense RNA 1 [Source:HGNC Symbol;Acc:HGNC:44102]	265.5	120.3	**-2.20**	6.93E-26
STK32B	serine/threonine kinase 32B [Source:HGNC Symbol;Acc:HGNC:14217]	180.1	82.3	**-2.20**	1.46E-14
MICALL2	MICAL like 2 [Source:HGNC Symbol;Acc:HGNC:29672]	120.2	52.9	**-2.20**	3.75E-12
HELLS	helicase, lymphoid specific [Source:HGNC Symbol;Acc:HGNC:4861]	1119.9	539.8	**-2.20**	3.37E-10
PAXX	PAXX, non-homologous end joining factor [Source:HGNC Symbol;Acc:HGNC:27849]	66.7	29.2	**-2.20**	1.62E-09
RECQL4	RecQ like helicase 4 [Source:HGNC Symbol;Acc:HGNC:9949]	90.0	39.9	**-2.20**	1.04E-08
INSC	INSC, spindle orientation adaptor protein [Source:HGNC Symbol;Acc:HGNC:33116]	119.2	55.5	**-2.20**	1.57E-07
APOBEC3B-AS1	APOBEC3B antisense RNA 1 [Source:HGNC Symbol;Acc:HGNC:43836]	33.1	13.5	**-2.20**	1.93E-05
FAM198B-AS1	FAM198B antisense RNA 1 [Source:HGNC Symbol;Acc:HGNC:53132]	23.2	9.1	**-2.20**	0.00366
MGAT5	mannosyl (alpha-1,6-)-glycoprotein beta-1,6-N-acetyl-glucosaminyltransferase [Source:HGNC Symbol;Ac	1581.3	712.0	**-2.22**	3.44E-108
COL5A2	collagen type V alpha 2 chain [Source:HGNC Symbol;Acc:HGNC:2210]	19215.6	8756.4	**-2.22**	5.34E-72
SSPN	sarcospan [Source:HGNC Symbol;Acc:HGNC:11322]	1077.3	485.3	**-2.22**	3.90E-70
MKX	mohawk homeobox [Source:HGNC Symbol;Acc:HGNC:23729]	376.1	166.1	**-2.22**	2.29E-27
GNG2	G protein subunit gamma 2 [Source:HGNC Symbol;Acc:HGNC:4404]	264.6	116.1	**-2.22**	2.96E-21
PLK4	polo like kinase 4 [Source:HGNC Symbol;Acc:HGNC:11397]	312.1	144.7	**-2.22**	3.06E-16
RIPK4	receptor interacting serine/threonine kinase 4 [Source:HGNC Symbol;Acc:HGNC:496]	39.8	17.9	**-2.22**	4.69E-05
TRDC	T cell receptor delta constant [Source:HGNC Symbol;Acc:HGNC:12253]	30.4	11.2	**-2.22**	0.0004502
PICSAR	P38 inhibited cutaneous squamous cell carcinoma associated lincRNA [Source:HGNC Symbol;Acc:HGNC:	22.1	9.0	**-2.22**	0.0005283
TEDC2	tubulin epsilon and delta complex 2 [Source:HGNC Symbol;Acc:HGNC:25849]	26.5	10.8	**-2.22**	0.001663
AC099489.1	vitellogenin [Source:NCBI gene;Acc:400499]	19.4	7.2	**-2.22**	0.003034
DERL3	derlin 3 [Source:HGNC Symbol;Acc:HGNC:14236]	9.3	3.2	**-2.22**	0.006056
MAMSTR	MEF2 activating motif and SAP domain containing transcriptional regulator [Source:HGNC Symbol;Acc:H	13.5	4.7	**-2.22**	0.006103
LINC01759	long intergenic non-protein coding RNA 1759 [Source:HGNC Symbol;Acc:HGNC:52548]	8.3	2.5	**-2.22**	0.01508
LUM	lumican [Source:HGNC Symbol;Acc:HGNC:6724]	311312.2	141123.3	**-2.23**	1.23E-69
BEND6	BEN domain containing 6 [Source:HGNC Symbol;Acc:HGNC:20871]	742.4	337.3	**-2.23**	8.36E-39
RGMA	repulsive guidance molecule BMP co-receptor a [Source:HGNC Symbol;Acc:HGNC:30308]	235.5	103.1	**-2.23**	2.35E-17

CASC15	cancer susceptibility 15 (non-protein coding) [Source:HGNC Symbol;Acc:HGNC:28245]	64.4	26.9	**-2.23**	5.95E-09
ZNF648	zinc finger protein 648 [Source:HGNC Symbol;Acc:HGNC:18190]	69.0	29.9	**-2.23**	1.44E-08
CNTFR	ciliary neurotrophic factor receptor [Source:HGNC Symbol;Acc:HGNC:2170]	55.8	23.5	**-2.23**	8.81E-08
NR4A3	nuclear receptor subfamily 4 group A member 3 [Source:HGNC Symbol;Acc:HGNC:7982]	48.9	20.8	**-2.23**	1.51E-06
AGMAT	agmatinase [Source:HGNC Symbol;Acc:HGNC:18407]	39.1	16.5	**-2.23**	7.35E-06
RSAD2	radical S-adenosyl methionine domain containing 2 [Source:HGNC Symbol;Acc:HGNC:30908]	31.0	12.7	**-2.23**	7.37E-06
PRR7	proline rich 7, synaptic [Source:HGNC Symbol;Acc:HGNC:28130]	12.8	4.8	**-2.23**	0.002072
TBXA2R	thromboxane A2 receptor [Source:HGNC Symbol;Acc:HGNC:11608]	12.9	4.6	**-2.23**	0.004167
FNDC5	fibronectin type III domain containing 5 [Source:HGNC Symbol;Acc:HGNC:20240]	11.9	4.2	**-2.23**	0.004185
LRP8	LDL receptor related protein 8 [Source:HGNC Symbol;Acc:HGNC:6700]	2553.7	1137.9	**-2.25**	2.55E-68
VDR	vitamin D receptor [Source:HGNC Symbol;Acc:HGNC:12679]	317.2	140.8	**-2.25**	8.93E-32
CGNL1	cingulin like 1 [Source:HGNC Symbol;Acc:HGNC:25931]	1458.3	666.5	**-2.25**	2.00E-30
KIAA1549	KIAA1549 [Source:HGNC Symbol;Acc:HGNC:22219]	482.8	209.2	**-2.25**	4.68E-27
RERG	RAS like estrogen regulated growth inhibitor [Source:HGNC Symbol;Acc:HGNC:15980]	1279.6	551.0	**-2.25**	8.81E-26
EZH2	enhancer of zeste 2 polycomb repressive complex 2 subunit [Source:HGNC Symbol;Acc:HGNC:3527]	460.8	214.5	**-2.25**	2.52E-23
KIFC1	kinesin family member C1 [Source:HGNC Symbol;Acc:HGNC:6389]	376.0	176.0	**-2.25**	4.06E-17
MCM10	minichromosome maintenance 10 replication initiation factor [Source:HGNC Symbol;Acc:HGNC:18043]	330.4	154.8	**-2.25**	1.26E-10
PIMREG	PICALM interacting mitotic regulator [Source:HGNC Symbol;Acc:HGNC:25483]	148.6	67.8	**-2.25**	5.46E-09
TCEAL5	transcription elongation factor A like 5 [Source:HGNC Symbol;Acc:HGNC:22282]	26.5	10.7	**-2.25**	3.49E-05
CERKL	ceramide kinase like [Source:HGNC Symbol;Acc:HGNC:21699]	17.8	6.7	**-2.25**	0.001168
ADRA2A	adrenoceptor alpha 2A [Source:HGNC Symbol;Acc:HGNC:281]	16.1	5.5	**-2.25**	0.006703
ARHGEF40	Rho guanine nucleotide exchange factor 40 [Source:HGNC Symbol;Acc:HGNC:25516]	873.5	385.2	**-2.27**	1.56E-88
MEG3	maternally expressed 3 (non-protein coding) [Source:HGNC Symbol;Acc:HGNC:14575]	704.8	310.3	**-2.27**	1.13E-37
AJUBA	ajuba LIM protein [Source:HGNC Symbol;Acc:HGNC:20250]	825.2	360.0	**-2.27**	2.51E-30
LCTL	lactase like [Source:HGNC Symbol;Acc:HGNC:15583]	340.4	150.1	**-2.27**	1.55E-26
NFASC	neurofascin [Source:HGNC Symbol;Acc:HGNC:29866]	247.6	107.8	**-2.27**	4.24E-22
CDCA5	cell division cycle associated 5 [Source:HGNC Symbol;Acc:HGNC:14626]	225.1	102.7	**-2.27**	6.28E-16
SMOX	spermine oxidase [Source:HGNC Symbol;Acc:HGNC:15862]	403.7	172.8	**-2.27**	1.09E-13
SH3RF2	SH3 domain containing ring finger 2 [Source:HGNC Symbol;Acc:HGNC:26299]	149.3	64.4	**-2.27**	1.77E-13
CCNE1	cyclin E1 [Source:HGNC Symbol;Acc:HGNC:1589]	73.3	31.1	**-2.27**	1.14E-07
S100A3	S100 calcium binding protein A3 [Source:HGNC Symbol;Acc:HGNC:10493]	83.6	35.7	**-2.27**	3.30E-07
DNAH9	dynein axonemal heavy chain 9 [Source:HGNC Symbol;Acc:HGNC:2953]	50.6	21.5	**-2.27**	1.10E-06
HSPB6	heat shock protein family B (small) member 6 [Source:HGNC Symbol;Acc:HGNC:26511]	12.4	4.5	**-2.27**	0.00273
CDC42EP3	CDC42 effector protein 3 [Source:HGNC Symbol;Acc:HGNC:16943]	2768.5	1219.7	**-2.28**	7.18E-65
TBX4	T-box 4 [Source:HGNC Symbol;Acc:HGNC:11603]	697.9	309.3	**-2.28**	1.04E-45
COL2A1	collagen type II alpha 1 chain [Source:HGNC Symbol;Acc:HGNC:2200]	130240.1	56195.3	**-2.28**	2.51E-31
NYNRIN	NYN domain and retroviral integrase containing [Source:HGNC Symbol;Acc:HGNC:20165]	215.0	91.4	**-2.28**	1.59E-27
DHFR	dihydrofolate reductase [Source:HGNC Symbol;Acc:HGNC:2861]	1204.2	560.0	**-2.28**	6.05E-27
SHCBP1	SHC binding and spindle associated 1 [Source:HGNC Symbol;Acc:HGNC:29547]	1072.0	499.9	**-2.28**	9.96E-26
ENC1	ectodermal-neural cortex 1 [Source:HGNC Symbol;Acc:HGNC:3345]	211.1	91.2	**-2.28**	8.16E-23
PSAT1	phosphoserine aminotransferase 1 [Source:HGNC Symbol;Acc:HGNC:19129]	1799.9	760.7	**-2.28**	9.95E-17
KIF15	kinesin family member 15 [Source:HGNC Symbol;Acc:HGNC:17273]	469.7	223.7	**-2.28**	2.14E-16
POLQ	DNA polymerase theta [Source:HGNC Symbol;Acc:HGNC:9186]	279.8	128.4	**-2.28**	1.03E-15
ADCY5	adenylate cyclase 5 [Source:HGNC Symbol;Acc:HGNC:236]	58.4	24.1	**-2.28**	2.92E-10
ARVCF	ARVCF, delta catenin family member [Source:HGNC Symbol;Acc:HGNC:728]	55.2	22.1	**-2.28**	3.89E-08
TYMSOS	TYMS opposite strand [Source:HGNC Symbol;Acc:HGNC:29553]	41.5	17.3	**-2.28**	2.25E-07
TNFRSF14	TNF receptor superfamily member 14 [Source:HGNC Symbol;Acc:HGNC:11912]	35.8	14.0	**-2.28**	5.38E-05
APOBEC3A	apolipoprotein B mRNA editing enzyme catalytic subunit 3A [Source:HGNC Symbol;Acc:HGNC:17343]	26.6	10.2	**-2.28**	5.67E-05
BEND3P1	BEN domain containing 3 pseudogene 1 [Source:HGNC Symbol;Acc:HGNC:45014]	13.9	5.1	**-2.28**	0.001585
PTN	pleiotrophin [Source:HGNC Symbol;Acc:HGNC:9630]	222.4	97.6	**-2.30**	6.58E-29
SERTAD4	SERTA domain containing 4 [Source:HGNC Symbol;Acc:HGNC:25236]	301.7	129.6	**-2.30**	4.69E-18
BRIP1	BRCA1 interacting protein C-terminal helicase 1 [Source:HGNC Symbol;Acc:HGNC:20473]	261.1	124.5	**-2.30**	3.47E-12
JAK3	Janus kinase 3 [Source:HGNC Symbol;Acc:HGNC:6193]	113.7	49.4	**-2.30**	5.28E-12
ASF1B	anti-silencing function 1B histone chaperone [Source:HGNC Symbol;Acc:HGNC:20996]	85.2	37.2	**-2.30**	5.68E-09
DEPDC1B	DEP domain containing 1B [Source:HGNC Symbol;Acc:HGNC:24902]	69.5	29.5	**-2.30**	2.29E-07
ANO1	anoctamin 1 [Source:HGNC Symbol;Acc:HGNC:21625]	65.9	27.2	**-2.30**	6.91E-07
SCG2	secretogranin II [Source:HGNC Symbol;Acc:HGNC:10575]	29.0	11.6	**-2.30**	6.96E-05
MAPK13	mitogen-activated protein kinase 13 [Source:HGNC Symbol;Acc:HGNC:6875]	35.5	13.4	**-2.30**	0.0002199
IFNE	interferon epsilon [Source:HGNC Symbol;Acc:HGNC:18163]	10.2	3.1	**-2.30**	0.008918
COL15A1	collagen type XV alpha 1 chain [Source:HGNC Symbol;Acc:HGNC:2192]	9376.8	4058.2	**-2.31**	1.82E-101
NTN1	netrin 1 [Source:HGNC Symbol;Acc:HGNC:8029]	2476.2	1072.3	**-2.31**	9.66E-77
BHLHE41	basic helix-loop-helix family member e41 [Source:HGNC Symbol;Acc:HGNC:16617]	878.2	377.0	**-2.31**	3.73E-57
HIST1H2BB	histone cluster 1 H2B family member b [Source:HGNC Symbol;Acc:HGNC:4751]	791.6	362.0	**-2.31**	1.28E-35
RAPGEF5	Rap guanine nucleotide exchange factor 5 [Source:HGNC Symbol;Acc:HGNC:16862]	270.1	112.8	**-2.31**	4.16E-21
XAF1	XIAP associated factor 1 [Source:HGNC Symbol;Acc:HGNC:30932]	96.0	39.7	**-2.31**	7.68E-12
FBXO32	F-box protein 32 [Source:HGNC Symbol;Acc:HGNC:16731]	1134.2	495.0	**-2.31**	2.03E-11
EDA2R	ectodysplasin A2 receptor [Source:HGNC Symbol;Acc:HGNC:17756]	74.2	29.4	**-2.31**	2.55E-09
NRN1	neuritin 1 [Source:HGNC Symbol;Acc:HGNC:17972]	6464.2	2719.3	**-2.33**	6.09E-74
SLC6A6	solute carrier family 6 member 6 [Source:HGNC Symbol;Acc:HGNC:11052]	352.8	151.6	**-2.33**	5.11E-35
NID2	nidogen 2 [Source:HGNC Symbol;Acc:HGNC:13389]	259.4	110.5	**-2.33**	2.18E-28
ITGB3	integrin subunit beta 3 [Source:HGNC Symbol;Acc:HGNC:6156]	639.0	268.6	**-2.33**	1.73E-24
HIST1H3A	histone cluster 1 H3 family member a [Source:HGNC Symbol;Acc:HGNC:4766]	289.3	127.6	**-2.33**	1.63E-15

FBN2	fibrillin 2 [Source:HGNC Symbol;Acc:HGNC:3604]	134.8	61.9	**-2.33**	1.60E-10
UHRF1	ubiquitin like with PHD and ring finger domains 1 [Source:HGNC Symbol;Acc:HGNC:12556]	1065.9	481.9	**-2.33**	4.32E-10
TSLP	thymic stromal lymphopoietin [Source:HGNC Symbol;Acc:HGNC:30743]	47.8	19.4	**-2.33**	8.60E-09
RTL1	retrotransposon Gag like 1 [Source:HGNC Symbol;Acc:HGNC:14665]	50.8	19.6	**-2.33**	4.91E-08
RASA4	RAS p21 protein activator 4 [Source:HGNC Symbol;Acc:HGNC:23181]	35.1	13.9	**-2.33**	5.97E-07
E2F8	E2F transcription factor 8 [Source:HGNC Symbol;Acc:HGNC:24727]	36.3	15.0	**-2.33**	2.90E-05
MRGPRF-AS1	MRGPRF antisense RNA 1 [Source:HGNC Symbol;Acc:HGNC:51140]	24.9	9.6	**-2.33**	9.40E-05
C15orf48	chromosome 15 open reading frame 48 [Source:HGNC Symbol;Acc:HGNC:29898]	32.1	11.4	**-2.33**	0.0001264
SOD2P1	superoxide dismutase 2 pseudogene 1 [Source:HGNC Symbol;Acc:HGNC:45268]	23.7	8.4	**-2.33**	0.00101
TIMP3	TIMP metallopeptidase inhibitor 3 [Source:HGNC Symbol;Acc:HGNC:11822]	14668.7	6429.5	**-2.35**	5.06E-85
S100A4	S100 calcium binding protein A4 [Source:HGNC Symbol;Acc:HGNC:10494]	756.8	318.6	**-2.35**	1.31E-72
FHOD1	formin homology 2 domain containing 1 [Source:HGNC Symbol;Acc:HGNC:17905]	155.2	64.4	**-2.35**	6.77E-25
PRTFDC1	phosphoribosyl transferase domain containing 1 [Source:HGNC Symbol;Acc:HGNC:23333]	201.4	85.3	**-2.35**	1.20E-19
ZNF367	zinc finger protein 367 [Source:HGNC Symbol;Acc:HGNC:18320]	2931.8	1346.7	**-2.35**	1.87E-16
HIST1H2BO	histone cluster 1 H2B family member o [Source:HGNC Symbol;Acc:HGNC:4758]	219.6	100.8	**-2.35**	2.65E-15
GRAMD1B	GRAM domain containing 1B [Source:HGNC Symbol;Acc:HGNC:29214]	98.2	41.3	**-2.35**	1.39E-12
AMIGO2	adhesion molecule with Ig like domain 2 [Source:HGNC Symbol;Acc:HGNC:24073]	74.1	30.5	**-2.35**	5.76E-10
SNORD114-1	small nucleolar RNA, C/D box 114-1 [Source:HGNC Symbol;Acc:HGNC:32989]	9.7	3.0	**-2.35**	0.004595
VEGFA	vascular endothelial growth factor A [Source:HGNC Symbol;Acc:HGNC:12680]	8007.4	3366.9	**-2.36**	1.57E-65
TUB	tubby bipartite transcription factor [Source:HGNC Symbol;Acc:HGNC:12406]	420.3	179.2	**-2.36**	1.51E-38
ARRDC4	arrestin domain containing 4 [Source:HGNC Symbol;Acc:HGNC:28087]	478.6	197.5	**-2.36**	1.93E-28
THBD	thrombomodulin [Source:HGNC Symbol;Acc:HGNC:11784]	1376.5	556.2	**-2.36**	4.48E-12
STARD8	StAR related lipid transfer domain containing 8 [Source:HGNC Symbol;Acc:HGNC:19161]	46.3	18.4	**-2.36**	2.80E-07
BEGAIN	brain enriched guanylate kinase associated [Source:HGNC Symbol;Acc:HGNC:24163]	35.7	14.2	**-2.36**	7.19E-07
ZBTB12	zinc finger and BTB domain containing 12 [Source:HGNC Symbol;Acc:HGNC:19066]	28.8	11.3	**-2.36**	1.17E-05
PIF1	PIF1 5'-to-3' DNA helicase [Source:HGNC Symbol;Acc:HGNC:26220]	26.8	10.2	**-2.36**	6.42E-05
TRHR	thyrotropin releasing hormone receptor [Source:HGNC Symbol;Acc:HGNC:12299]	10.7	2.8	**-2.36**	0.008786
IER5L	immediate early response 5 like [Source:HGNC Symbol;Acc:HGNC:23679]	1284.4	542.3	**-2.38**	2.54E-67
MAP1A	microtubule associated protein 1A [Source:HGNC Symbol;Acc:HGNC:6835]	832.1	352.2	**-2.38**	8.57E-38
MRAP2	melanocortin 2 receptor accessory protein 2 [Source:HGNC Symbol;Acc:HGNC:21232]	1243.4	494.9	**-2.38**	3.10E-28
ZWINT	ZW10 interacting kinetochore protein [Source:HGNC Symbol;Acc:HGNC:13195]	905.3	398.7	**-2.38**	1.89E-14
OSBPL7	oxysterol binding protein like 7 [Source:HGNC Symbol;Acc:HGNC:16387]	50.7	20.0	**-2.38**	6.63E-09
BEND5	BEN domain containing 5 [Source:HGNC Symbol;Acc:HGNC:25668]	22.1	8.0	**-2.38**	5.13E-05
COL16A1	collagen type XVI alpha 1 chain [Source:HGNC Symbol;Acc:HGNC:2193]	991.9	408.0	**-2.39**	6.01E-60
TMEFF1	transmembrane protein with EGF like and two follistatin like domains 1 [Source:HGNC Symbol;Acc:HGN	274.1	114.8	**-2.39**	1.07E-30
CRISPLD1	cysteine rich secretory protein LCCL domain containing 1 [Source:HGNC Symbol;Acc:HGNC:18206]	1014.9	408.6	**-2.39**	6.13E-25
SLC7A11	solute carrier family 7 member 11 [Source:HGNC Symbol;Acc:HGNC:11059]	3049.4	1187.0	**-2.39**	1.36E-12
CTSW	cathepsin W [Source:HGNC Symbol;Acc:HGNC:2546]	57.2	21.9	**-2.39**	2.16E-08
SMIM2-AS1	SMIM2 antisense RNA 1 [Source:HGNC Symbol;Acc:HGNC:42674]	46.1	17.3	**-2.39**	1.55E-05
PRR19	proline rich 19 [Source:HGNC Symbol;Acc:HGNC:33728]	22.9	8.3	**-2.39**	0.0001392
TCF4	transcription factor 4 [Source:HGNC Symbol;Acc:HGNC:11634]	3602.0	1473.3	**-2.41**	1.15E-55
SLC7A11-AS1	SLC7A11 antisense RNA 1 [Source:HGNC Symbol;Acc:HGNC:44064]	399.4	156.8	**-2.41**	3.02E-18
APOBEC3B	apolipoprotein B mRNA editing enzyme catalytic subunit 3B [Source:HGNC Symbol;Acc:HGNC:17352]	111.9	45.3	**-2.41**	3.20E-13
ZNF385C	zinc finger protein 385C [Source:HGNC Symbol;Acc:HGNC:33722]	109.7	43.5	**-2.41**	5.86E-13
PRAL	p53 regulation associated lncRNA [Source:HGNC Symbol;Acc:HGNC:52646]	79.1	31.2	**-2.41**	5.81E-10
IGDCC4	immunoglobulin superfamily DCC subclass member 4 [Source:HGNC Symbol;Acc:HGNC:13770]	33.9	12.8	**-2.41**	1.32E-05
ZNF295-AS1	ZNF295 antisense RNA 1 [Source:HGNC Symbol;Acc:HGNC:23130]	8.1	2.0	**-2.41**	0.007464
BHLHE40	basic helix-loop-helix family member e40 [Source:HGNC Symbol;Acc:HGNC:1046]	2800.9	1142.6	**-2.43**	1.36E-59
MSANTD3-TMEFF1	MSANTD3-TMEFF1 readthrough [Source:HGNC Symbol;Acc:HGNC:38838]	467.6	193.2	**-2.43**	7.97E-52
AFF3	AF4/FMR2 family member 3 [Source:HGNC Symbol;Acc:HGNC:6473]	397.1	158.4	**-2.43**	9.12E-34
TINAGL1	tubulointerstitial nephritis antigen like 1 [Source:HGNC Symbol;Acc:HGNC:19168]	188.4	75.8	**-2.43**	5.28E-15
NR4A2	nuclear receptor subfamily 4 group A member 2 [Source:HGNC Symbol;Acc:HGNC:7981]	114.7	44.2	**-2.43**	1.68E-10
EXO1	exonuclease 1 [Source:HGNC Symbol;Acc:HGNC:3511]	394.3	170.6	**-2.43**	3.67E-09
SOX12	SRY-box 12 [Source:HGNC Symbol;Acc:HGNC:11198]	46.6	17.8	**-2.43**	5.01E-09
ANO1-AS1	ANO1 antisense RNA 1 [Source:HGNC Symbol;Acc:HGNC:40016]	11.6	3.3	**-2.43**	0.004545
ENPP2	ectonucleotide pyrophosphatase/phosphodiesterase 2 [Source:HGNC Symbol;Acc:HGNC:3357]	1560.3	652.6	**-2.45**	3.15E-95
LPXN	leupaxin [Source:HGNC Symbol;Acc:HGNC:14061]	376.4	151.9	**-2.45**	8.40E-40
KIAA1211	KIAA1211 [Source:HGNC Symbol;Acc:HGNC:29219]	273.6	110.2	**-2.45**	6.11E-37
GINS4	GINS complex subunit 4 [Source:HGNC Symbol;Acc:HGNC:28226]	243.6	102.2	**-2.45**	2.08E-18
SLC4A4	solute carrier family 4 member 4 [Source:HGNC Symbol;Acc:HGNC:11030]	106.3	41.6	**-2.45**	7.06E-16
C1QTNF5	C1q and TNF related 5 [Source:HGNC Symbol;Acc:HGNC:14344]	118.4	47.6	**-2.45**	1.10E-13
PLEK	pleckstrin [Source:HGNC Symbol;Acc:HGNC:9070]	36.8	13.6	**-2.45**	2.35E-06
HIST1H2BM	histone cluster 1 H2B family member m [Source:HGNC Symbol;Acc:HGNC:4750]	57.1	25.4	**-2.45**	6.02E-06
RASA4B	RAS p21 protein activator 4B [Source:HGNC Symbol;Acc:HGNC:35202]	24.5	8.7	**-2.45**	3.30E-05
BHLHE22	basic helix-loop-helix family member e22 [Source:HGNC Symbol;Acc:HGNC:11963]	10.7	3.4	**-2.45**	0.001351
GAMT	guanidinoacetate N-methyltransferase [Source:HGNC Symbol;Acc:HGNC:4136]	23.5	7.3	**-2.45**	0.001747
KRT15	keratin 15 [Source:HGNC Symbol;Acc:HGNC:6421]	11.1	3.2	**-2.45**	0.003825
RGS3	regulator of G protein signaling 3 [Source:HGNC Symbol;Acc:HGNC:9999]	1964.2	774.3	**-2.46**	2.89E-78
PDGFRA	platelet derived growth factor receptor alpha [Source:HGNC Symbol;Acc:HGNC:8803]	14544.5	5868.8	**-2.46**	9.92E-76
PLCL2	phospholipase C like 2 [Source:HGNC Symbol;Acc:HGNC:9064]	278.0	112.6	**-2.46**	1.17E-30
LIG1	DNA ligase 1 [Source:HGNC Symbol;Acc:HGNC:6598]	369.0	151.3	**-2.46**	4.13E-27
TPM1-AS	TPM1 antisense RNA [Source:HGNC Symbol;Acc:HGNC:53635]	172.6	68.3	**-2.46**	9.87E-20

DACT1	dishevelled binding antagonist of beta catenin 1 [Source:HGNC Symbol;Acc:HGNC:17748]	416.3	165.8	**-2.46**	5.13E-15
TCF19	transcription factor 19 [Source:HGNC Symbol;Acc:HGNC:11629]	897.0	382.3	**-2.46**	5.24E-14
PDE1A	phosphodiesterase 1A [Source:HGNC Symbol;Acc:HGNC:8774]	174.2	69.1	**-2.46**	7.33E-11
SULT1A1	sulfotransferase family 1A member 1 [Source:HGNC Symbol;Acc:HGNC:11453]	24.1	8.2	**-2.46**	8.51E-05
EPHB2	EPH receptor B2 [Source:HGNC Symbol;Acc:HGNC:3393]	248.8	98.0	**-2.48**	7.16E-34
HBEGF	heparin binding EGF like growth factor [Source:HGNC Symbol;Acc:HGNC:3059]	172.0	67.5	**-2.48**	4.31E-25
SEMA7A	semaphorin 7A (John Milton Hagen blood group) [Source:HGNC Symbol;Acc:HGNC:10741]	213.4	83.1	**-2.48**	1.89E-21
HIST1H2AB	histone cluster 1 H2A family member b [Source:HGNC Symbol;Acc:HGNC:4734]	297.5	127.6	**-2.48**	7.66E-17
CHAC1	ChaC glutathione specific gamma-glutamylcyclotransferase 1 [Source:HGNC Symbol;Acc:HGNC:28680]	152.1	58.8	**-2.48**	3.43E-15
GNAZ	G protein subunit alpha z [Source:HGNC Symbol;Acc:HGNC:4395]	116.1	46.4	**-2.48**	3.16E-14
CMPK2	cytidine/uridine monophosphate kinase 2 [Source:HGNC Symbol;Acc:HGNC:27015]	40.7	15.3	**-2.48**	2.44E-06
E2F7	E2F transcription factor 7 [Source:HGNC Symbol;Acc:HGNC:23820]	56.6	21.6	**-2.48**	9.04E-06
DNM3OS	DNM3 opposite strand/antisense RNA [Source:HGNC Symbol;Acc:HGNC:41228]	1345.4	535.1	**-2.50**	1.44E-70
PDGFRB	platelet derived growth factor receptor beta [Source:HGNC Symbol;Acc:HGNC:8804]	524.0	209.9	**-2.50**	5.59E-25
MEGF6	multiple EGF like domains 6 [Source:HGNC Symbol;Acc:HGNC:3232]	244.3	97.1	**-2.50**	1.12E-22
HIST1H4L	histone cluster 1 H4 family member l [Source:HGNC Symbol;Acc:HGNC:4791]	165.4	73.9	**-2.50**	2.81E-13
PLCL2-AS1	PLCL2 antisense RNA 1 [Source:HGNC Symbol;Acc:HGNC:40449]	17.4	5.7	**-2.50**	0.000312
TMEM37	transmembrane protein 37 [Source:HGNC Symbol;Acc:HGNC:18216]	8.0	2.1	**-2.50**	0.004204
LNX1	ligand of numb-protein X 1 [Source:HGNC Symbol;Acc:HGNC:6657]	1880.6	746.8	**-2.51**	1.47E-80
SDC1	syndecan 1 [Source:HGNC Symbol;Acc:HGNC:10658]	1102.8	438.6	**-2.51**	1.43E-58
LMO4	LIM domain only 4 [Source:HGNC Symbol;Acc:HGNC:6644]	1724.5	670.9	**-2.51**	1.54E-44
TK1	thymidine kinase 1 [Source:HGNC Symbol;Acc:HGNC:11830]	355.4	147.1	**-2.51**	1.46E-24
GP1BB	glycoprotein Ib platelet subunit beta [Source:HGNC Symbol;Acc:HGNC:4440]	147.8	57.1	**-2.51**	9.72E-24
PCLAF	PCNA clamp associated factor [Source:HGNC Symbol;Acc:HGNC:28961]	383.0	171.4	**-2.51**	1.12E-15
OAS3	2'-5'-oligoadenylate synthetase 3 [Source:HGNC Symbol;Acc:HGNC:8088]	106.7	41.1	**-2.51**	2.59E-11
RCOR2	REST corepressor 2 [Source:HGNC Symbol;Acc:HGNC:27455]	27.9	9.8	**-2.51**	1.66E-05
ZNF423	zinc finger protein 423 [Source:HGNC Symbol;Acc:HGNC:16762]	29.5	10.2	**-2.51**	5.07E-05
RSPH14	radial spoke head 14 homolog [Source:HGNC Symbol;Acc:HGNC:13437]	17.6	5.9	**-2.51**	0.0002591
DLC1	DLC1 Rho GTPase activating protein [Source:HGNC Symbol;Acc:HGNC:2897]	3208.5	1267.0	**-2.53**	1.28E-155
KIAA1755	KIAA1755 [Source:HGNC Symbol;Acc:HGNC:29372]	443.9	174.9	**-2.53**	3.17E-19
RRM2	ribonucleotide reductase regulatory subunit M2 [Source:HGNC Symbol;Acc:HGNC:10452]	2026.6	860.1	**-2.53**	6.41E-17
CLSPN	claspin [Source:HGNC Symbol;Acc:HGNC:19715]	968.0	410.7	**-2.53**	6.82E-15
UBE2QL1	ubiquitin conjugating enzyme E2 Q family like 1 [Source:HGNC Symbol;Acc:HGNC:37269]	42.4	14.6	**-2.53**	1.57E-05
PDZRN3-AS1	PDZRN3 antisense RNA 1 [Source:HGNC Symbol;Acc:HGNC:40814]	8.6	1.6	**-2.53**	0.006264
NQO1	NAD(P)H quinone dehydrogenase 1 [Source:HGNC Symbol;Acc:HGNC:2874]	3720.3	1485.3	**-2.55**	2.55E-86
BFSP1	beaded filament structural protein 1 [Source:HGNC Symbol;Acc:HGNC:1040]	225.9	87.0	**-2.55**	1.94E-32
NKX3-2	NK3 homeobox 2 [Source:HGNC Symbol;Acc:HGNC:951]	161.1	61.5	**-2.55**	5.84E-23
GALM	galactose mutarotase [Source:HGNC Symbol;Acc:HGNC:24063]	271.7	108.4	**-2.55**	6.81E-22
LMO2	LIM domain only 2 [Source:HGNC Symbol;Acc:HGNC:6642]	80.7	29.8	**-2.55**	8.22E-12
SCX	scleraxis bHLH transcription factor [Source:HGNC Symbol;Acc:HGNC:32322]	32.4	11.7	**-2.55**	1.14E-07
LGI2	leucine rich repeat LGI family member 2 [Source:HGNC Symbol;Acc:HGNC:18710]	31.2	11.0	**-2.55**	1.34E-06
VSNL1	visinin like 1 [Source:HGNC Symbol;Acc:HGNC:12722]	21.8	6.7	**-2.55**	0.0003509
TMIE	transmembrane inner ear [Source:HGNC Symbol;Acc:HGNC:30800]	10.9	2.9	**-2.55**	0.002158
TGFB2	transforming growth factor beta 2 [Source:HGNC Symbol;Acc:HGNC:11768]	7649.3	2938.2	**-2.57**	1.44E-72
EXT1	exostosin glycosyltransferase 1 [Source:HGNC Symbol;Acc:HGNC:3512]	559.3	216.7	**-2.57**	2.48E-70
HIST1H1B	histone cluster 1 H1 family member b [Source:HGNC Symbol;Acc:HGNC:4719]	608.4	278.2	**-2.57**	8.14E-25
PTH1R	parathyroid hormone 1 receptor [Source:HGNC Symbol;Acc:HGNC:9608]	213.8	82.0	**-2.57**	1.67E-19
E2F1	E2F transcription factor 1 [Source:HGNC Symbol;Acc:HGNC:3113]	124.8	51.1	**-2.57**	1.73E-11
FHOD3	formin homology 2 domain containing 3 [Source:HGNC Symbol;Acc:HGNC:26178]	67.1	24.3	**-2.57**	2.65E-09
TPM1	tropomyosin 1 [Source:HGNC Symbol;Acc:HGNC:12010]	5207.5	2001.4	**-2.58**	1.02E-118
FABP3	fatty acid binding protein 3 [Source:HGNC Symbol;Acc:HGNC:3557]	624.2	241.1	**-2.58**	1.18E-48
SAMD11	sterile alpha motif domain containing 11 [Source:HGNC Symbol;Acc:HGNC:28706]	394.6	146.8	**-2.58**	4.97E-37
ARHGEF3	Rho guanine nucleotide exchange factor 3 [Source:HGNC Symbol;Acc:HGNC:683]	333.8	129.0	**-2.58**	2.87E-35
GINS1	GINS complex subunit 1 [Source:HGNC Symbol;Acc:HGNC:28980]	324.8	130.2	**-2.58**	6.72E-22
ORC6	origin recognition complex subunit 6 [Source:HGNC Symbol;Acc:HGNC:17151]	247.8	99.6	**-2.58**	2.21E-18
CDC45	cell division cycle 45 [Source:HGNC Symbol;Acc:HGNC:1739]	370.1	149.8	**-2.58**	1.26E-09
TMCC2	transmembrane and coiled-coil domain family 2 [Source:HGNC Symbol;Acc:HGNC:24239]	45.3	15.9	**-2.58**	1.17E-07
CX3CL1	C-X3-C motif chemokine ligand 1 [Source:HGNC Symbol;Acc:HGNC:10647]	30.1	10.6	**-2.58**	1.10E-06
ZMAT4	zinc finger matrin-type 4 [Source:HGNC Symbol;Acc:HGNC:25844]	25.7	8.9	**-2.58**	1.27E-05
KCNN3	potassium calcium-activated channel subfamily N member 3 [Source:HGNC Symbol;Acc:HGNC:6292]	37.5	12.0	**-2.58**	1.60E-05
IL11	interleukin 11 [Source:HGNC Symbol;Acc:HGNC:5966]	30.4	8.2	**-2.58**	5.76E-05
TMEM213	transmembrane protein 213 [Source:HGNC Symbol;Acc:HGNC:27220]	13.2	4.1	**-2.58**	0.0001572
TMEM132E	transmembrane protein 132E [Source:HGNC Symbol;Acc:HGNC:26991]	13.1	3.3	**-2.58**	0.002344
MSANTD3	Myb/SANT DNA binding domain containing 3 [Source:HGNC Symbol;Acc:HGNC:23370]	1232.5	475.8	**-2.60**	1.15E-97
SOX8	SRY-box 8 [Source:HGNC Symbol;Acc:HGNC:11203]	762.0	296.8	**-2.60**	1.04E-43
NLGN4Y	neuroligin 4 Y-linked [Source:HGNC Symbol;Acc:HGNC:15529]	281.3	97.1	**-2.60**	2.76E-24
PLEKHB1	pleckstrin homology domain containing B1 [Source:HGNC Symbol;Acc:HGNC:19079]	62.6	23.0	**-2.60**	1.31E-12
HIST1H3J	histone cluster 1 H3 family member j [Source:HGNC Symbol;Acc:HGNC:4774]	505.5	216.8	**-2.60**	1.03E-11
HIST1H2AM	histone cluster 1 H2A family member m [Source:HGNC Symbol;Acc:HGNC:4735]	482.0	207.2	**-2.60**	2.82E-11
PAFAH1B3	platelet activating factor acetylhydrolase 1b catalytic subunit 3 [Source:HGNC Symbol;Acc:HGNC:8576]	79.9	28.5	**-2.60**	1.39E-10
DTL	denticleless E3 ubiquitin protein ligase homolog [Source:HGNC Symbol;Acc:HGNC:30288]	580.3	238.5	**-2.60**	7.25E-10
FGF1	fibroblast growth factor 1 [Source:HGNC Symbol;Acc:HGNC:3665]	6015.9	2323.5	**-2.62**	1.50E-64

PTGER3	prostaglandin E receptor 3 [Source:HGNC Symbol;Acc:HGNC:9595]	485.3	183.4	**-2.62**	9.54E-41
ASPN	asporin [Source:HGNC Symbol;Acc:HGNC:14872]	1728.1	577.8	**-2.62**	3.65E-19
NXPH4	neurexophilin 4 [Source:HGNC Symbol;Acc:HGNC:8078]	56.2	19.4	**-2.62**	1.89E-06
ERICH3	glutamate rich 3 [Source:HGNC Symbol;Acc:HGNC:25346]	31.3	10.0	**-2.62**	0.0002506
TAGLN3	transgelin 3 [Source:HGNC Symbol;Acc:HGNC:29868]	13.1	2.3	**-2.62**	0.004181
F13A1	coagulation factor XIII A chain [Source:HGNC Symbol;Acc:HGNC:3531]	7760.9	2942.7	**-2.64**	1.54E-95
GAS7	growth arrest specific 7 [Source:HGNC Symbol;Acc:HGNC:4169]	1888.3	717.0	**-2.64**	1.59E-77
RANBP3L	RAN binding protein 3 like [Source:HGNC Symbol;Acc:HGNC:26353]	576.2	215.1	**-2.64**	8.38E-27
BCL2A1	BCL2 related protein A1 [Source:HGNC Symbol;Acc:HGNC:991]	108.8	35.8	**-2.64**	4.19E-13
PRDM8	PR/SET domain 8 [Source:HGNC Symbol;Acc:HGNC:13993]	62.9	22.6	**-2.64**	1.91E-10
LRRTM2	leucine rich repeat transmembrane neuronal 2 [Source:HGNC Symbol;Acc:HGNC:19409]	59.3	20.5	**-2.64**	1.06E-07
TNIK	TRAF2 and NCK interacting kinase [Source:HGNC Symbol;Acc:HGNC:30765]	1652.0	619.7	**-2.66**	4.06E-80
TGFB2-OT1	TGFB2 overlapping transcript 1 [Source:HGNC Symbol;Acc:HGNC:50629]	2173.0	808.0	**-2.66**	5.19E-63
TYMS	thymidylate synthetase [Source:HGNC Symbol;Acc:HGNC:12441]	703.2	273.9	**-2.66**	6.37E-32
HIST2H3D	histone cluster 2 H3 family member d [Source:HGNC Symbol;Acc:HGNC:25311]	118.5	43.4	**-2.66**	9.64E-15
GPR19	G protein-coupled receptor 19 [Source:HGNC Symbol;Acc:HGNC:4473]	67.0	24.0	**-2.66**	1.94E-11
TNFRSF1B	TNF receptor superfamily member 1B [Source:HGNC Symbol;Acc:HGNC:11917]	42.7	14.8	**-2.66**	1.50E-08
CD300C	CD300c molecule [Source:HGNC Symbol;Acc:HGNC:19320]	47.8	16.7	**-2.66**	4.61E-08
HCAR1	hydroxycarboxylic acid receptor 1 [Source:HGNC Symbol;Acc:HGNC:4532]	19.3	6.1	**-2.66**	1.90E-05
IL36RN	interleukin 36 receptor antagonist [Source:HGNC Symbol;Acc:HGNC:15561]	8.9	1.6	**-2.66**	0.003852
ADAM12	ADAM metallopeptidase domain 12 [Source:HGNC Symbol;Acc:HGNC:190]	1359.7	509.3	**-2.68**	1.13E-133
HCFC1R1	host cell factor C1 regulator 1 [Source:HGNC Symbol;Acc:HGNC:21198]	437.2	161.2	**-2.68**	1.81E-50
SPC25	SPC25, NDC80 kinetochore complex component [Source:HGNC Symbol;Acc:HGNC:24031]	236.4	89.9	**-2.68**	2.10E-29
SLC6A9	solute carrier family 6 member 9 [Source:HGNC Symbol;Acc:HGNC:11056]	156.4	53.7	**-2.68**	1.05E-11
PHYHIPL	phytanoyl-CoA 2-hydroxylase interacting protein like [Source:HGNC Symbol;Acc:HGNC:29378]	29.0	8.8	**-2.68**	2.29E-05
ABCA4	ATP binding cassette subfamily A member 4 [Source:HGNC Symbol;Acc:HGNC:34]	14.6	4.3	**-2.68**	0.0002287
ITGA3	integrin subunit alpha 3 [Source:HGNC Symbol;Acc:HGNC:6139]	2532.3	933.3	**-2.69**	4.83E-133
CHST11	carbohydrate sulfotransferase 11 [Source:HGNC Symbol;Acc:HGNC:17422]	771.5	274.9	**-2.69**	3.25E-84
COL14A1	collagen type XIV alpha 1 chain [Source:HGNC Symbol;Acc:HGNC:2191]	1474.2	562.4	**-2.69**	1.08E-49
PLXND1	plexin D1 [Source:HGNC Symbol;Acc:HGNC:9107]	448.4	166.1	**-2.69**	8.09E-41
UNC5B	unc-5 netrin receptor B [Source:HGNC Symbol;Acc:HGNC:12568]	642.7	230.3	**-2.69**	7.54E-36
ABCB4	ATP binding cassette subfamily B member 4 [Source:HGNC Symbol;Acc:HGNC:45]	163.0	57.2	**-2.69**	5.82E-16
RIBC2	RIB43A domain with coiled-coils 2 [Source:HGNC Symbol;Acc:HGNC:13241]	42.5	15.0	**-2.69**	1.76E-07
SMIM2	small integral membrane protein 2 [Source:HGNC Symbol;Acc:HGNC:28776]	31.0	9.6	**-2.69**	2.14E-05
SORL1	sortilin related receptor 1 [Source:HGNC Symbol;Acc:HGNC:11185]	590.3	213.8	**-2.71**	4.47E-59
SSC5D	scavenger receptor cysteine rich family member with 5 domains [Source:HGNC Symbol;Acc:HGNC:2664	44.1	15.1	**-2.71**	1.02E-10
TSPAN11	tetraspanin 11 [Source:HGNC Symbol;Acc:HGNC:30795]	53.3	18.2	**-2.71**	1.93E-10
BHMT2	betaine--homocysteine S-methyltransferase 2 [Source:HGNC Symbol;Acc:HGNC:1048]	48.9	16.9	**-2.71**	1.62E-09
SHISA9	shisa family member 9 [Source:HGNC Symbol;Acc:HGNC:37231]	46.1	15.2	**-2.71**	1.29E-08
SERPINI2	serpin family I member 2 [Source:HGNC Symbol;Acc:HGNC:8945]	12.3	2.9	**-2.71**	0.001415
LOXL2	lysyl oxidase like 2 [Source:HGNC Symbol;Acc:HGNC:6666]	694.4	248.2	**-2.73**	3.20E-65
HIST1H2BI	histone cluster 1 H2B family member i [Source:HGNC Symbol;Acc:HGNC:4756]	350.1	143.3	**-2.73**	4.61E-24
RCSD1	RCSD domain containing 1 [Source:HGNC Symbol;Acc:HGNC:28310]	157.6	54.9	**-2.73**	2.56E-14
RAB7B	RAB7B, member RAS oncogene family [Source:HGNC Symbol;Acc:HGNC:30513]	39.1	13.0	**-2.73**	1.04E-06
DACT3	dishevelled binding antagonist of beta catenin 3 [Source:HGNC Symbol;Acc:HGNC:30745]	28.1	9.0	**-2.73**	2.02E-06
TNFRSF10C	TNF receptor superfamily member 10c [Source:HGNC Symbol;Acc:HGNC:11906]	26.4	8.3	**-2.73**	4.88E-05
RCAN1	regulator of calcineurin 1 [Source:HGNC Symbol;Acc:HGNC:3040]	7643.3	2747.5	**-2.75**	1.33E-132
MAFB	MAF bZIP transcription factor B [Source:HGNC Symbol;Acc:HGNC:6408]	339.0	123.8	**-2.75**	1.11E-46
CYSLTR1	cysteinyl leukotriene receptor 1 [Source:HGNC Symbol;Acc:HGNC:17451]	120.9	42.8	**-2.75**	5.23E-12
WFDC1	WAP four-disulfide core domain 1 [Source:HGNC Symbol;Acc:HGNC:15466]	48.8	16.3	**-2.75**	3.19E-08
KCNH2	potassium voltage-gated channel subfamily H member 2 [Source:HGNC Symbol;Acc:HGNC:6251]	16.1	4.8	**-2.75**	1.50E-05
NCALD	neurocalcin delta [Source:HGNC Symbol;Acc:HGNC:7655]	15.8	3.9	**-2.75**	0.0009084
SYTL2	synaptotagmin like 2 [Source:HGNC Symbol;Acc:HGNC:15585]	304.0	106.9	**-2.77**	5.04E-35
RAD51AP1	RAD51 associated protein 1 [Source:HGNC Symbol;Acc:HGNC:16956]	371.0	142.6	**-2.77**	2.04E-27
HIST1H2AL	histone cluster 1 H2A family member l [Source:HGNC Symbol;Acc:HGNC:4730]	183.6	74.3	**-2.77**	6.66E-17
FAM111B	family with sequence similarity 111 member B [Source:HGNC Symbol;Acc:HGNC:24200]	1127.9	469.5	**-2.77**	3.69E-13
TMEM26-AS1	TMEM26 antisense RNA 1 [Source:HGNC Symbol;Acc:HGNC:51209]	30.9	9.9	**-2.77**	3.66E-06
SCARA5	scavenger receptor class A member 5 [Source:HGNC Symbol;Acc:HGNC:28701]	42.5	13.0	**-2.77**	4.90E-06
SLC29A1	solute carrier family 29 member 1 (Augustine blood group) [Source:HGNC Symbol;Acc:HGNC:11003]	3242.6	1149.1	**-2.79**	5.60E-78
PRICKLE1	prickle planar cell polarity protein 1 [Source:HGNC Symbol;Acc:HGNC:17019]	562.1	198.1	**-2.79**	7.73E-41
SBSPON	somatomedin B and thrombospondin type 1 domain containing [Source:HGNC Symbol;Acc:HGNC:30362	506.7	179.5	**-2.79**	1.36E-38
STMN3	stathmin 3 [Source:HGNC Symbol;Acc:HGNC:15926]	162.9	56.8	**-2.79**	1.88E-23
HIST1H2AH	histone cluster 1 H2A family member h [Source:HGNC Symbol;Acc:HGNC:13671]	729.8	280.7	**-2.79**	3.99E-17
GRIA2	glutamate ionotropic receptor AMPA type subunit 2 [Source:HGNC Symbol;Acc:HGNC:4572]	75.7	24.9	**-2.79**	4.00E-14
HIST1H3G	histone cluster 1 H3 family member g [Source:HGNC Symbol;Acc:HGNC:4772]	154.9	58.4	**-2.79**	1.54E-12
ESCO2	establishment of sister chromatid cohesion N-acetyltransferase 2 [Source:HGNC Symbol;Acc:HGNC:272	418.7	153.9	**-2.79**	3.00E-09
HIST1H2BL	histone cluster 1 H2B family member l [Source:HGNC Symbol;Acc:HGNC:4748]	67.5	26.6	**-2.79**	6.31E-08
LGR6	leucine rich repeat containing G protein-coupled receptor 6 [Source:HGNC Symbol;Acc:HGNC:19719]	15.2	3.1	**-2.79**	0.001286
SMOC1	SPARC related modular calcium binding 1 [Source:HGNC Symbol;Acc:HGNC:20318]	2975.0	1059.5	**-2.81**	8.34E-153
SHC4	SHC adaptor protein 4 [Source:HGNC Symbol;Acc:HGNC:16743]	902.4	328.6	**-2.81**	5.71E-44
HIST1H2AI	histone cluster 1 H2A family member i [Source:HGNC Symbol;Acc:HGNC:4725]	160.6	62.6	**-2.81**	2.08E-18
CCDC110	coiled-coil domain containing 110 [Source:HGNC Symbol;Acc:HGNC:28504]	154.1	53.3	**-2.81**	7.56E-16

DBP	D-box binding PAR bZIP transcription factor [Source:HGNC Symbol;Acc:HGNC:2697]	95.9	31.7	**-2.81**	4.43E-14
NUP210	nucleoporin 210 [Source:HGNC Symbol;Acc:HGNC:30052]	14.3	3.8	**-2.81**	0.0001117
GEM	GTP binding protein overexpressed in skeletal muscle [Source:HGNC Symbol;Acc:HGNC:4234]	1677.5	578.1	**-2.83**	6.09E-74
WTAPP1	Wilms tumor 1 associated protein pseudogene 1 [Source:HGNC Symbol;Acc:HGNC:44115]	7929.5	2843.0	**-2.83**	1.43E-27
ADAMTS3	ADAM metallopeptidase with thrombospondin type 1 motif 3 [Source:HGNC Symbol;Acc:HGNC:219]	207.5	70.6	**-2.83**	7.10E-26
GABRB1	gamma-aminobutyric acid type A receptor beta1 subunit [Source:HGNC Symbol;Acc:HGNC:4081]	79.5	26.4	**-2.83**	1.84E-13
PPP1R3G	protein phosphatase 1 regulatory subunit 3G [Source:HGNC Symbol;Acc:HGNC:14945]	23.8	6.9	**-2.83**	1.88E-05
RAB11FIP4	RAB11 family interacting protein 4 [Source:HGNC Symbol;Acc:HGNC:30267]	2354.2	817.4	**-2.85**	1.22E-113
MMP1	matrix metallopeptidase 1 [Source:HGNC Symbol;Acc:HGNC:7155]	26580.9	9513.7	**-2.85**	2.99E-32
TUBA8	tubulin alpha 8 [Source:HGNC Symbol;Acc:HGNC:12410]	90.3	30.6	**-2.85**	8.17E-17
ELF3	E74 like ETS transcription factor 3 [Source:HGNC Symbol;Acc:HGNC:3318]	95.2	33.1	**-2.85**	8.03E-10
FXYD2	FXYD domain containing ion transport regulator 2 [Source:HGNC Symbol;Acc:HGNC:4026]	25.3	7.5	**-2.85**	9.16E-06
AXIN2	axin 2 [Source:HGNC Symbol;Acc:HGNC:904]	750.0	260.7	**-2.87**	3.06E-72
SPX	spexin hormone [Source:HGNC Symbol;Acc:HGNC:28139]	307.6	98.2	**-2.87**	7.67E-16
PLXNA4	plexin A4 [Source:HGNC Symbol;Acc:HGNC:9102]	81.9	25.1	**-2.87**	6.59E-08
FBLN7	fibulin 7 [Source:HGNC Symbol;Acc:HGNC:26740]	167.6	55.9	**-2.89**	3.89E-23
KIF5C	kinesin family member 5C [Source:HGNC Symbol;Acc:HGNC:6325]	36.7	10.8	**-2.89**	1.34E-06
GFAP	glial fibrillary acidic protein [Source:HGNC Symbol;Acc:HGNC:4235]	31.4	9.1	**-2.89**	6.99E-06
TOX	thymocyte selection associated high mobility group box [Source:HGNC Symbol;Acc:HGNC:18988]	21.6	5.8	**-2.89**	4.39E-05
SYT8	synaptotagmin 8 [Source:HGNC Symbol;Acc:HGNC:19264]	12.3	2.6	**-2.89**	0.0005165
EFR3B	EFR3 homolog B [Source:HGNC Symbol;Acc:HGNC:29155]	246.8	83.2	**-2.91**	2.71E-48
HIST1H2AJ	histone cluster 1 H2A family member j [Source:HGNC Symbol;Acc:HGNC:4727]	486.5	193.2	**-2.91**	5.28E-26
RNF128	ring finger protein 128, E3 ubiquitin protein ligase [Source:HGNC Symbol;Acc:HGNC:21153]	97.6	29.8	**-2.91**	2.22E-14
FAM133A	family with sequence similarity 133 member A [Source:HGNC Symbol;Acc:HGNC:26748]	57.4	18.1	**-2.91**	4.18E-10
ID4	inhibitor of DNA binding 4, HLH protein [Source:HGNC Symbol;Acc:HGNC:5363]	1384.6	466.8	**-2.93**	1.10E-136
THY1	Thy-1 cell surface antigen [Source:HGNC Symbol;Acc:HGNC:11801]	1022.6	357.0	**-2.93**	1.61E-112
LOXL1	lysyl oxidase like 1 [Source:HGNC Symbol;Acc:HGNC:6665]	269.1	90.9	**-2.93**	9.09E-37
MRGPRF	MAS related GPR family member F [Source:HGNC Symbol;Acc:HGNC:24828]	168.7	54.0	**-2.93**	1.12E-18
COL5A1	collagen type V alpha 1 chain [Source:HGNC Symbol;Acc:HGNC:2209]	5372.5	1790.5	**-2.95**	1.06E-89
ENG	endoglin [Source:HGNC Symbol;Acc:HGNC:3349]	831.9	275.9	**-2.95**	6.32E-58
HIST1H3B	histone cluster 1 H3 family member b [Source:HGNC Symbol;Acc:HGNC:4776]	918.3	337.5	**-2.95**	1.50E-19
PKNOX2	PBX/knotted 1 homeobox 2 [Source:HGNC Symbol;Acc:HGNC:16714]	161.5	51.8	**-2.95**	4.81E-17
ADCY1	adenylate cyclase 1 [Source:HGNC Symbol;Acc:HGNC:232]	71.9	22.8	**-2.95**	8.56E-12
FAM13C	family with sequence similarity 13 member C [Source:HGNC Symbol;Acc:HGNC:19371]	52.3	15.0	**-2.95**	4.88E-09
BMF-AS1	BMF antisense RNA 1 [Source:HGNC Symbol;Acc:HGNC:53612]	21.0	5.9	**-2.95**	5.44E-06
TUBB2B	tubulin beta 2B class IIb [Source:HGNC Symbol;Acc:HGNC:30829]	940.4	315.2	**-2.97**	3.86E-70
EVA1A	eva-1 homolog A, regulator of programmed cell death [Source:HGNC Symbol;Acc:HGNC:25816]	70.4	21.9	**-2.97**	6.26E-10
RGS13	regulator of G protein signaling 13 [Source:HGNC Symbol;Acc:HGNC:9995]	13.6	3.3	**-2.97**	6.76E-05
HIST1H3C	histone cluster 1 H3 family member c [Source:HGNC Symbol;Acc:HGNC:4768]	707.8	252.2	**-3.01**	2.03E-18
PDZRN3	PDZ domain containing ring finger 3 [Source:HGNC Symbol;Acc:HGNC:17704]	675.0	230.0	**-3.03**	7.05E-39
HIST1H2AG	histone cluster 1 H2A family member g [Source:HGNC Symbol;Acc:HGNC:4737]	871.8	323.0	**-3.03**	1.83E-36
SPTB	spectrin beta, erythrocytic [Source:HGNC Symbol;Acc:HGNC:11274]	79.9	24.4	**-3.03**	2.16E-13
SHROOM3	shroom family member 3 [Source:HGNC Symbol;Acc:HGNC:30422]	499.7	162.1	**-3.05**	1.48E-56
APLN	apelin [Source:HGNC Symbol;Acc:HGNC:16665]	24.6	6.6	**-3.05**	1.34E-06
LINC00284	long intergenic non-protein coding RNA 284 [Source:HGNC Symbol;Acc:HGNC:26981]	19.2	4.9	**-3.05**	1.34E-05
LINC00163	long intergenic non-protein coding RNA 163 [Source:HGNC Symbol;Acc:HGNC:33165]	16.0	3.7	**-3.05**	4.03E-05
PTGIS	prostaglandin I2 synthase [Source:HGNC Symbol;Acc:HGNC:9603]	400.3	130.0	**-3.07**	1.27E-44
MSX1	msh homeobox 1 [Source:HGNC Symbol;Acc:HGNC:7391]	29.9	8.5	**-3.10**	1.19E-08
COL11A1	collagen type XI alpha 1 chain [Source:HGNC Symbol;Acc:HGNC:2186]	59852.0	19522.6	**-3.12**	4.32E-160
MMP16	matrix metallopeptidase 16 [Source:HGNC Symbol;Acc:HGNC:7162]	1004.9	323.5	**-3.12**	2.83E-75
COL1A1	collagen type I alpha 1 chain [Source:HGNC Symbol;Acc:HGNC:2197]	146.3	46.8	**-3.12**	6.02E-11
LRRN2	leucine rich repeat neuronal 2 [Source:HGNC Symbol;Acc:HGNC:16914]	33.2	9.3	**-3.12**	3.47E-09
RGS4	regulator of G protein signaling 4 [Source:HGNC Symbol;Acc:HGNC:10000]	28.0	7.5	**-3.12**	4.73E-08
FAM129A	family with sequence similarity 129 member A [Source:HGNC Symbol;Acc:HGNC:16784]	3935.8	1225.9	**-3.14**	1.58E-119
FLRT2	fibronectin leucine rich transmembrane protein 2 [Source:HGNC Symbol;Acc:HGNC:3761]	427.6	135.8	**-3.14**	3.22E-41
TLR5	toll like receptor 5 [Source:HGNC Symbol;Acc:HGNC:11851]	85.5	25.9	**-3.14**	5.10E-19
LAMA4	laminin subunit alpha 4 [Source:HGNC Symbol;Acc:HGNC:6484]	2794.7	875.5	**-3.16**	6.89E-126
CSF1	colony stimulating factor 1 [Source:HGNC Symbol;Acc:HGNC:2432]	3078.9	982.4	**-3.16**	1.60E-38
APCDD1L	APC down-regulated 1 like [Source:HGNC Symbol;Acc:HGNC:26892]	185.2	59.0	**-3.16**	1.95E-14
SLC6A17	solute carrier family 6 member 17 [Source:HGNC Symbol;Acc:HGNC:31399]	23.2	5.9	**-3.16**	1.92E-07
LIF	LIF, interleukin 6 family cytokine [Source:HGNC Symbol;Acc:HGNC:6596]	477.6	142.3	**-3.18**	7.19E-33
OLFML2B	olfactomedin like 2B [Source:HGNC Symbol;Acc:HGNC:24558]	98.9	29.0	**-3.18**	1.17E-19
SPEG	SPEG complex locus [Source:HGNC Symbol;Acc:HGNC:16901]	113.6	33.6	**-3.18**	9.34E-17
E2F2	E2F transcription factor 2 [Source:HGNC Symbol;Acc:HGNC:3114]	81.4	25.9	**-3.18**	5.81E-13
WISP1	WNT1 inducible signaling pathway protein 1 [Source:HGNC Symbol;Acc:HGNC:12769]	368.6	114.2	**-3.20**	2.51E-35
TRERF1	transcriptional regulating factor 1 [Source:HGNC Symbol;Acc:HGNC:18273]	87.1	26.1	**-3.20**	5.40E-23
CLEC3A	C-type lectin domain family 3 member A [Source:HGNC Symbol;Acc:HGNC:2052]	734.1	198.9	**-3.20**	1.35E-14
MYOZ3	myozenin 3 [Source:HGNC Symbol;Acc:HGNC:18565]	16.6	3.9	**-3.20**	6.54E-06
PGBD5	piggyBac transposable element derived 5 [Source:HGNC Symbol;Acc:HGNC:19405]	13.3	3.0	**-3.20**	1.57E-05
FOXA3	forkhead box A3 [Source:HGNC Symbol;Acc:HGNC:5023]	83.4	24.6	**-3.23**	2.29E-18
NANOS1	nanos C2HC-type zinc finger 1 [Source:HGNC Symbol;Acc:HGNC:23044]	93.3	24.3	**-3.25**	1.93E-13
HIST1H4A	histone cluster 1 H4 family member a [Source:HGNC Symbol;Acc:HGNC:4781]	53.8	15.5	**-3.25**	5.93E-12

AQP7	aquaporin 7 [Source:HGNC Symbol;Acc:HGNC:640]	67.4	18.6	**-3.25**	1.17E-11
FIBIN	fin bud initiation factor homolog (zebrafish) [Source:HGNC Symbol;Acc:HGNC:33747]	3967.8	1205.4	**-3.27**	1.54E-191
TSC22D1	TSC22 domain family member 1 [Source:HGNC Symbol;Acc:HGNC:16826]	12833.1	3877.7	**-3.27**	3.40E-134
LRRC15	leucine rich repeat containing 15 [Source:HGNC Symbol;Acc:HGNC:20818]	867.3	264.0	**-3.27**	1.13E-73
EXTL1	exostosin like glycosyltransferase 1 [Source:HGNC Symbol;Acc:HGNC:3515]	251.0	77.2	**-3.27**	7.68E-41
CD82	CD82 molecule [Source:HGNC Symbol;Acc:HGNC:6210]	307.3	92.8	**-3.27**	2.16E-38
CAP2	cyclase associated actin cytoskeleton regulatory protein 2 [Source:HGNC Symbol;Acc:HGNC:20039]	251.4	76.1	**-3.27**	1.05E-29
C11orf21	chromosome 11 open reading frame 21 [Source:HGNC Symbol;Acc:HGNC:13231]	25.7	6.0	**-3.27**	1.51E-06
RDH10	retinol dehydrogenase 10 [Source:HGNC Symbol;Acc:HGNC:19975]	2048.7	607.7	**-3.29**	1.24E-105
NIM1K	NIM1 serine/threonine protein kinase [Source:HGNC Symbol;Acc:HGNC:28646]	392.3	117.5	**-3.29**	4.25E-57
RTL3	retrotransposon Gag like 3 [Source:HGNC Symbol;Acc:HGNC:22997]	165.3	48.8	**-3.29**	2.05E-30
IER3-AS1	IER3 antisense RNA 1 [Source:HGNC Symbol;Acc:HGNC:53629]	381.7	113.6	**-3.32**	1.12E-40
LINC01415	long intergenic non-protein coding RNA 1415 [Source:HGNC Symbol;Acc:HGNC:50709]	59.3	15.6	**-3.34**	1.33E-12
WNT5A	Wnt family member 5A [Source:HGNC Symbol;Acc:HGNC:12784]	138.7	32.4	**-3.34**	2.19E-06
GPD1	glycerol-3-phosphate dehydrogenase 1 [Source:HGNC Symbol;Acc:HGNC:4455]	83.2	22.6	**-3.36**	4.96E-16
RAB39B	RAB39B, member RAS oncogene family [Source:HGNC Symbol;Acc:HGNC:16499]	38.2	9.8	**-3.36**	8.18E-09
SUSD4	sushi domain containing 4 [Source:HGNC Symbol;Acc:HGNC:25470]	21.8	4.8	**-3.36**	3.17E-06
TRIL	TLR4 interactor with leucine rich repeats [Source:HGNC Symbol;Acc:HGNC:22200]	15.8	2.3	**-3.36**	0.0001052
OAF	out at first homolog [Source:HGNC Symbol;Acc:HGNC:28752]	759.4	221.3	**-3.39**	1.50E-127
PDE5A	phosphodiesterase 5A [Source:HGNC Symbol;Acc:HGNC:8784]	708.5	199.9	**-3.39**	1.14E-113
CRACR2B	calcium release activated channel regulator 2B [Source:HGNC Symbol;Acc:HGNC:28703]	39.8	9.9	**-3.39**	3.15E-08
IGFBP1	insulin like growth factor binding protein 1 [Source:HGNC Symbol;Acc:HGNC:5469]	157.1	42.8	**-3.41**	2.88E-13
IER3	immediate early response 3 [Source:HGNC Symbol;Acc:HGNC:5392]	1019.7	294.5	**-3.43**	2.73E-71
TRIB3	tribbles pseudokinase 3 [Source:HGNC Symbol;Acc:HGNC:16228]	325.9	90.2	**-3.43**	1.19E-29
EGR3	early growth response 3 [Source:HGNC Symbol;Acc:HGNC:3240]	54.5	14.6	**-3.43**	3.54E-12
GLI1	GLI family zinc finger 1 [Source:HGNC Symbol;Acc:HGNC:4317]	24.9	5.7	**-3.43**	1.00E-08
FZD8	frizzled class receptor 8 [Source:HGNC Symbol;Acc:HGNC:4046]	5499.0	1599.1	**-3.48**	2.85E-278
SPTSSB	serine palmitoyltransferase small subunit B [Source:HGNC Symbol;Acc:HGNC:24045]	178.8	50.1	**-3.48**	3.19E-23
MYBL1	MYB proto-oncogene like 1 [Source:HGNC Symbol;Acc:HGNC:7547]	2195.0	641.5	**-3.48**	5.94E-23
CD79B	CD79b molecule [Source:HGNC Symbol;Acc:HGNC:1699]	90.2	24.9	**-3.48**	8.18E-22
FRMD7	FERM domain containing 7 [Source:HGNC Symbol;Acc:HGNC:8079]	42.4	9.4	**-3.48**	1.98E-07
TGFB3-AS1	TGFB3 antisense RNA 1 [Source:HGNC Symbol;Acc:HGNC:53144]	21.5	4.2	**-3.48**	4.53E-06
SERPINB9P1	serpin family B member 9 pseudogene 1 [Source:HGNC Symbol;Acc:HGNC:28590]	18.0	3.5	**-3.48**	5.42E-06
SSTR2	somatostatin receptor 2 [Source:HGNC Symbol;Acc:HGNC:11331]	87.3	22.9	**-3.51**	6.67E-21
BTBD16	BTB domain containing 16 [Source:HGNC Symbol;Acc:HGNC:26340]	23.6	4.7	**-3.53**	2.83E-06
SHISA3	shisa family member 3 [Source:HGNC Symbol;Acc:HGNC:25159]	485.0	134.5	**-3.56**	1.94E-78
MAF	MAF bZIP transcription factor [Source:HGNC Symbol;Acc:HGNC:6776]	2317.0	648.5	**-3.58**	5.42E-79
GRIN2A	glutamate ionotropic receptor NMDA type subunit 2A [Source:HGNC Symbol;Acc:HGNC:4585]	222.6	58.3	**-3.58**	9.36E-22
SHISAL1	shisa like 1 [Source:HGNC Symbol;Acc:HGNC:29335]	214.9	58.5	**-3.61**	8.13E-30
SMIM10L2A	small integral membrane protein 10 like 2A [Source:HGNC Symbol;Acc:HGNC:34499]	35.5	8.2	**-3.61**	1.17E-09
MYBL2	MYB proto-oncogene like 2 [Source:HGNC Symbol;Acc:HGNC:7548]	38.9	8.9	**-3.61**	7.08E-09
PHLDA1	pleckstrin homology like domain family A member 1 [Source:HGNC Symbol;Acc:HGNC:8933]	3810.6	1038.6	**-3.63**	1.41E-148
TMEM158	transmembrane protein 158 (gene/pseudogene) [Source:HGNC Symbol;Acc:HGNC:30293]	120.4	28.6	**-3.63**	1.18E-19
FAM198B	family with sequence similarity 198 member B [Source:HGNC Symbol;Acc:HGNC:25312]	148.2	39.5	**-3.63**	7.15E-16
COL9A1	collagen type IX alpha 1 chain [Source:HGNC Symbol;Acc:HGNC:2217]	678.5	176.2	**-3.66**	3.90E-71
BMF	Bcl2 modifying factor [Source:HGNC Symbol;Acc:HGNC:24132]	240.9	64.4	**-3.66**	1.50E-35
NDP-AS1	NDP antisense RNA 1 [Source:HGNC Symbol;Acc:HGNC:40395]	35.6	7.3	**-3.66**	1.79E-07
TUBB3	tubulin beta 3 class III [Source:HGNC Symbol;Acc:HGNC:20772]	249.5	66.2	**-3.68**	1.82E-29
GPR68	G protein-coupled receptor 68 [Source:HGNC Symbol;Acc:HGNC:4519]	91.0	23.5	**-3.68**	1.70E-20
ACTC1	actin, alpha, cardiac muscle 1 [Source:HGNC Symbol;Acc:HGNC:143]	255.1	65.2	**-3.71**	1.22E-27
ANGPTL2	angiopoietin like 2 [Source:HGNC Symbol;Acc:HGNC:490]	5717.0	1557.8	**-3.76**	7.12E-115
INHBE	inhibin subunit beta E [Source:HGNC Symbol;Acc:HGNC:24029]	22.9	4.7	**-3.76**	6.87E-09
CDH11	cadherin 11 [Source:HGNC Symbol;Acc:HGNC:1750]	3197.8	853.6	**-3.78**	5.42E-45
ELN	elastin [Source:HGNC Symbol;Acc:HGNC:3327]	377.4	99.1	**-3.78**	6.54E-36
KCNJ12	potassium voltage-gated channel subfamily J member 12 [Source:HGNC Symbol;Acc:HGNC:6258]	101.3	25.3	**-3.78**	6.08E-24
LRRC17	leucine rich repeat containing 17 [Source:HGNC Symbol;Acc:HGNC:16895]	53.8	12.2	**-3.78**	1.02E-13
TDO2	tryptophan 2,3-dioxygenase [Source:HGNC Symbol;Acc:HGNC:11708]	20.4	3.2	**-3.78**	2.58E-06
PIEZO2	piezo type mechanosensitive ion channel component 2 [Source:HGNC Symbol;Acc:HGNC:26270]	2106.9	548.9	**-3.81**	7.71E-117
IGFBP3	insulin like growth factor binding protein 3 [Source:HGNC Symbol;Acc:HGNC:5472]	89825.2	24371.5	**-3.81**	5.70E-75
EPHB3	EPH receptor B3 [Source:HGNC Symbol;Acc:HGNC:3394]	237.1	61.3	**-3.81**	1.67E-35
LINC01016	long intergenic non-protein coding RNA 1016 [Source:HGNC Symbol;Acc:HGNC:48991]	20.7	3.8	**-3.81**	2.05E-07
SEMA6D	semaphorin 6D [Source:HGNC Symbol;Acc:HGNC:16770]	275.0	70.4	**-3.84**	6.41E-63
BMPER	BMP binding endothelial regulator [Source:HGNC Symbol;Acc:HGNC:24154]	447.1	110.9	**-3.84**	2.59E-41
TNFRSF19	TNF receptor superfamily member 19 [Source:HGNC Symbol;Acc:HGNC:11915]	100.5	24.8	**-3.84**	9.43E-26
TMEM26	transmembrane protein 26 [Source:HGNC Symbol;Acc:HGNC:28550]	510.8	130.0	**-3.86**	1.28E-60
IL6	interleukin 6 [Source:HGNC Symbol;Acc:HGNC:6018]	25.8	4.0	**-3.86**	1.89E-07
GRIP2	glutamate receptor interacting protein 2 [Source:HGNC Symbol;Acc:HGNC:23841]	16.8	2.8	**-3.86**	9.00E-07
B3GNT7	UDP-GlcNAc:betaGal beta-1,3-N-acetylglucosaminyltransferase 7 [Source:HGNC Symbol;Acc:HGNC:1881	1331.8	325.1	**-3.89**	5.21E-27
PRAG1	PEAK1 related, kinase-activating pseudokinase 1 [Source:HGNC Symbol;Acc:HGNC:25438]	28.8	6.1	**-3.89**	6.59E-12
RAP1GAP	RAP1 GTPase activating protein [Source:HGNC Symbol;Acc:HGNC:9858]	30.6	6.5	**-3.89**	6.76E-12
IFITM10	interferon induced transmembrane protein 10 [Source:HGNC Symbol;Acc:HGNC:40022]	605.5	154.3	**-3.92**	8.02E-100
SLC38A3	solute carrier family 38 member 3 [Source:HGNC Symbol;Acc:HGNC:18044]	96.2	22.4	**-3.92**	1.12E-20

FGF7	fibroblast growth factor 7 [Source:HGNC Symbol;Acc:HGNC:3685]	173.3	39.5	**-3.92**	2.01E-20
LINC01105	long intergenic non-protein coding RNA 1105 [Source:HGNC Symbol;Acc:HGNC:26403]	66.7	15.9	**-3.94**	2.32E-20
TNFRSF6B	TNF receptor superfamily member 6b [Source:HGNC Symbol;Acc:HGNC:11921]	67.7	15.0	**-3.94**	3.21E-15
CDON	cell adhesion associated, oncogene regulated [Source:HGNC Symbol;Acc:HGNC:17104]	5052.9	1256.5	**-3.97**	8.15E-189
RFLNA	refilin A [Source:HGNC Symbol;Acc:HGNC:27051]	356.5	87.2	**-4.00**	1.09E-72
PPFIA4	PTPRF interacting protein alpha 4 [Source:HGNC Symbol;Acc:HGNC:9248]	370.0	90.4	**-4.00**	3.66E-65
PHOSPHO1	phosphoethanolamine/phosphocholine phosphatase [Source:HGNC Symbol;Acc:HGNC:16815]	18.6	3.0	**-4.00**	2.89E-07
GAPLINC	gastric adenocarcinoma associated, positive CD44 regulator, long intergenic non-coding RNA [Source:HG	10.7	1.1	**-4.00**	7.45E-06
FZD9	frizzled class receptor 9 [Source:HGNC Symbol;Acc:HGNC:4047]	327.9	80.2	**-4.03**	1.84E-53
WSCD2	WSC domain containing 2 [Source:HGNC Symbol;Acc:HGNC:29117]	673.1	164.7	**-4.06**	2.32E-56
BDKRB2	bradykinin receptor B2 [Source:HGNC Symbol;Acc:HGNC:1030]	333.1	81.8	**-4.06**	1.05E-39
NOV	nephroblastoma overexpressed [Source:HGNC Symbol;Acc:HGNC:7885]	330.9	79.3	**-4.08**	4.85E-60
HIST1H1A	histone cluster 1 H1 family member a [Source:HGNC Symbol;Acc:HGNC:4715]	663.6	173.1	**-4.08**	2.13E-23
SNCG	synuclein gamma [Source:HGNC Symbol;Acc:HGNC:11141]	72.9	16.4	**-4.08**	1.20E-20
MMP13	matrix metallopeptidase 13 [Source:HGNC Symbol;Acc:HGNC:7159]	2452.4	536.6	**-4.08**	2.31E-13
PLA2G4A	phospholipase A2 group IVA [Source:HGNC Symbol;Acc:HGNC:9035]	4572.1	1101.3	**-4.14**	1.68E-191
TGM3	transglutaminase 3 [Source:HGNC Symbol;Acc:HGNC:11779]	16.2	2.2	**-4.14**	9.94E-07
WWTR1	WW domain containing transcription regulator 1 [Source:HGNC Symbol;Acc:HGNC:24042]	8693.9	2054.1	**-4.20**	1.98E-304
PDE3A	phosphodiesterase 3A [Source:HGNC Symbol;Acc:HGNC:8778]	122.1	21.9	**-4.26**	3.72E-09
ADAMTS1	ADAM metallopeptidase with thrombospondin type 1 motif 1 [Source:HGNC Symbol;Acc:HGNC:217]	4476.2	1022.4	**-4.29**	2.15E-239
SEMA3D	semaphorin 3D [Source:HGNC Symbol;Acc:HGNC:10726]	5748.1	1338.0	**-4.29**	2.68E-175
PTGS2	prostaglandin-endoperoxide synthase 2 [Source:HGNC Symbol;Acc:HGNC:9605]	1232.1	280.9	**-4.29**	6.53E-142
PLK2	polo like kinase 2 [Source:HGNC Symbol;Acc:HGNC:19699]	494.3	114.5	**-4.29**	1.47E-80
H19	H19, imprinted maternally expressed transcript (non-protein coding) [Source:HGNC Symbol;Acc:HGNC:4	6887.1	1599.6	**-4.29**	6.31E-34
GRIN2C	glutamate ionotropic receptor NMDA type subunit 2C [Source:HGNC Symbol;Acc:HGNC:4587]	59.9	11.8	**-4.32**	8.03E-14
LINC01229	long intergenic non-protein coding RNA 1229 [Source:HGNC Symbol;Acc:HGNC:49682]	30.5	5.4	**-4.35**	8.80E-12
PART1	prostate androgen-regulated transcript 1 (non-protein coding) [Source:HGNC Symbol;Acc:HGNC:17263]	755.3	169.7	**-4.38**	2.04E-124
PDGFC	platelet derived growth factor C [Source:HGNC Symbol;Acc:HGNC:8801]	2136.5	485.4	**-4.38**	1.38E-119
MDFI	MyoD family inhibitor [Source:HGNC Symbol;Acc:HGNC:6967]	573.5	127.0	**-4.44**	1.46E-121
RGS16	regulator of G protein signaling 16 [Source:HGNC Symbol;Acc:HGNC:9997]	565.8	125.8	**-4.44**	6.14E-80
WWTR1-AS1	WWTR1 antisense RNA 1 [Source:HGNC Symbol;Acc:HGNC:41035]	222.2	48.5	**-4.44**	1.63E-62
FGFR3	fibroblast growth factor receptor 3 [Source:HGNC Symbol;Acc:HGNC:3690]	1524.2	338.9	**-4.50**	3.85E-123
LPAR4	lysophosphatidic acid receptor 4 [Source:HGNC Symbol;Acc:HGNC:4478]	347.6	73.2	**-4.53**	4.22E-57
ARHGAP28	Rho GTPase activating protein 28 [Source:HGNC Symbol;Acc:HGNC:25509]	167.8	33.4	**-4.56**	8.13E-35
SOX4	SRY-box 4 [Source:HGNC Symbol;Acc:HGNC:11200]	1548.9	339.8	**-4.59**	8.47E-204
CEMIP	cell migration inducing hyaluronidase 1 [Source:HGNC Symbol;Acc:HGNC:29213]	1005.5	226.6	**-4.59**	9.86E-155
DCSTAMP	dendrocyte expressed seven transmembrane protein [Source:HGNC Symbol;Acc:HGNC:18549]	10.0	0.8	**-4.59**	4.76E-07
TENM2	teneurin transmembrane protein 2 [Source:HGNC Symbol;Acc:HGNC:29943]	64.5	10.0	**-4.63**	4.76E-11
BIRC7	baculoviral IAP repeat containing 7 [Source:HGNC Symbol;Acc:HGNC:13702]	10.6	0.8	**-4.63**	5.04E-07
BTBD11	BTB domain containing 11 [Source:HGNC Symbol;Acc:HGNC:23844]	370.8	76.6	**-4.69**	8.00E-64
GPR88	G protein-coupled receptor 88 [Source:HGNC Symbol;Acc:HGNC:4539]	1502.1	314.8	**-4.69**	6.82E-37
HS3ST1	heparan sulfate-glucosamine 3-sulfotransferase 1 [Source:HGNC Symbol;Acc:HGNC:5194]	112.3	22.6	**-4.69**	3.51E-31
SCN2B	sodium voltage-gated channel beta subunit 2 [Source:HGNC Symbol;Acc:HGNC:10589]	203.8	41.3	**-4.69**	9.54E-31
DOK7	docking protein 7 [Source:HGNC Symbol;Acc:HGNC:26594]	27.3	4.0	**-4.69**	7.11E-10
NDP	NDP, norrin cystine knot growth factor [Source:HGNC Symbol;Acc:HGNC:7678]	259.2	51.5	**-4.72**	3.35E-41
IFNWP19	interferon omega 1 pseudogene 19 [Source:HGNC Symbol;Acc:HGNC:5451]	106.3	21.0	**-4.72**	6.12E-25
COL27A1	collagen type XXVII alpha 1 chain [Source:HGNC Symbol;Acc:HGNC:22986]	2220.9	468.6	**-4.76**	7.10E-164
IFIT1	interferon induced protein with tetratricopeptide repeats 1 [Source:HGNC Symbol;Acc:HGNC:5407]	233.3	47.2	**-4.76**	7.51E-68
CRABP2	cellular retinoic acid binding protein 2 [Source:HGNC Symbol;Acc:HGNC:2339]	27.2	4.0	**-4.79**	5.61E-11
TGFB3	transforming growth factor beta 3 [Source:HGNC Symbol;Acc:HGNC:11769]	318.8	63.1	**-4.86**	6.00E-70
LFNG	LFNG O-fucosylpeptide 3-beta-N-acetylglucosaminyltransferase [Source:HGNC Symbol;Acc:HGNC:6560]	38.9	6.5	**-4.86**	7.04E-14
CD34	CD34 molecule [Source:HGNC Symbol;Acc:HGNC:1662]	28.0	4.1	**-4.86**	7.65E-11
LINC01503	long intergenic non-protein coding RNA 1503 [Source:HGNC Symbol;Acc:HGNC:51184]	91.7	16.6	**-4.89**	4.16E-18
OSR1	odd-skipped related transciption factor 1 [Source:HGNC Symbol;Acc:HGNC:8111]	39.9	6.4	**-4.89**	1.26E-13
SEMA3A	semaphorin 3A [Source:HGNC Symbol;Acc:HGNC:10723]	12555.6	2538.6	**-4.92**	1.02E-223
SLC2A12	solute carrier family 2 member 12 [Source:HGNC Symbol;Acc:HGNC:18067]	742.4	136.7	**-4.92**	3.99E-82
KCNE4	potassium voltage-gated channel subfamily E regulatory subunit 4 [Source:HGNC Symbol;Acc:HGNC:624	289.2	57.0	**-4.92**	4.90E-76
RSPO2	R-spondin 2 [Source:HGNC Symbol;Acc:HGNC:28583]	2787.1	538.3	**-4.96**	2.83E-208
P2RX6	purinergic receptor P2X 6 [Source:HGNC Symbol;Acc:HGNC:8538]	46.6	7.2	**-4.96**	9.32E-13
KCNA1	potassium voltage-gated channel subfamily A member 1 [Source:HGNC Symbol;Acc:HGNC:6218]	161.8	30.6	**-4.99**	1.91E-45
COL13A1	collagen type XIII alpha 1 chain [Source:HGNC Symbol;Acc:HGNC:2190]	51.2	7.2	**-4.99**	2.24E-11
PMP2	peripheral myelin protein 2 [Source:HGNC Symbol;Acc:HGNC:9117]	24.2	2.4	**-4.99**	2.01E-08
PKP1	plakophilin 1 [Source:HGNC Symbol;Acc:HGNC:9023]	40.4	6.5	**-5.03**	4.17E-17
ATP6V0A4	ATPase H+ transporting V0 subunit a4 [Source:HGNC Symbol;Acc:HGNC:866]	29.8	3.8	**-5.03**	8.08E-11
PGF	placental growth factor [Source:HGNC Symbol;Acc:HGNC:8893]	73.6	11.4	**-5.13**	2.84E-17
DIO2	iodothyronine deiodinase 2 [Source:HGNC Symbol;Acc:HGNC:2884]	1663.5	311.0	**-5.21**	1.08E-109
CPA4	carboxypeptidase A4 [Source:HGNC Symbol;Acc:HGNC:15740]	808.1	149.6	**-5.21**	6.32E-93
LSP1	lymphocyte-specific protein 1 [Source:HGNC Symbol;Acc:HGNC:6707]	1714.7	326.1	**-5.28**	4.05E-55
MIR31HG	MIR31 host gene [Source:HGNC Symbol;Acc:HGNC:37187]	104.0	17.5	**-5.31**	2.78E-20
BDKRB1	bradykinin receptor B1 [Source:HGNC Symbol;Acc:HGNC:1029]	25.2	2.7	**-5.31**	4.53E-10
PSTPIP1	proline-serine-threonine phosphatase interacting protein 1 [Source:HGNC Symbol;Acc:HGNC:9580]	440.3	81.0	**-5.35**	1.83E-82
PODXL	podocalyxin like [Source:HGNC Symbol;Acc:HGNC:9171]	895.6	161.7	**-5.43**	2.29E-99

CCL2	C-C motif chemokine ligand 2 [Source:HGNC Symbol;Acc:HGNC:10618]	176.3	29.9	**-5.43**	6.24E-36
SYT13	synaptotagmin 13 [Source:HGNC Symbol;Acc:HGNC:14962]	106.9	16.6	**-5.43**	2.15E-25
TNNT3	troponin T3, fast skeletal type [Source:HGNC Symbol;Acc:HGNC:11950]	81.5	12.6	**-5.54**	7.56E-22
NOG	noggin [Source:HGNC Symbol;Acc:HGNC:7866]	316.5	52.3	**-5.78**	8.65E-43
GABRA4	gamma-aminobutyric acid type A receptor alpha4 subunit [Source:HGNC Symbol;Acc:HGNC:4078]	2490.7	410.8	**-5.82**	4.58E-42
CAMK2A	calcium/calmodulin dependent protein kinase II alpha [Source:HGNC Symbol;Acc:HGNC:1460]	39.2	5.3	**-5.82**	9.46E-18
LZTS1	leucine zipper tumor suppressor 1 [Source:HGNC Symbol;Acc:HGNC:13861]	166.3	26.4	**-5.86**	2.35E-46
IGFBP5	insulin like growth factor binding protein 5 [Source:HGNC Symbol;Acc:HGNC:5474]	2688.8	435.7	**-5.90**	2.69E-143
GDF6	growth differentiation factor 6 [Source:HGNC Symbol;Acc:HGNC:4221]	4986.7	754.5	**-5.98**	1.68E-37
TNFSF15	TNF superfamily member 15 [Source:HGNC Symbol;Acc:HGNC:11931]	50.5	6.0	**-5.98**	1.39E-14
GDF15	growth differentiation factor 15 [Source:HGNC Symbol;Acc:HGNC:30142]	141.2	21.9	**-6.06**	2.72E-59
MAP1LC3C	microtubule associated protein 1 light chain 3 gamma [Source:HGNC Symbol;Acc:HGNC:13353]	31.3	2.3	**-6.32**	3.60E-11
TRPM8	transient receptor potential cation channel subfamily M member 8 [Source:HGNC Symbol;Acc:HGNC:17	531.7	81.4	**-6.41**	2.64E-72
HAS2-AS1	HAS2 antisense RNA 1 [Source:HGNC Symbol;Acc:HGNC:34340]	106.5	14.5	**-6.59**	5.74E-38
C10orf55	chromosome 10 open reading frame 55 [Source:HGNC Symbol;Acc:HGNC:31008]	39.9	3.8	**-6.59**	5.40E-14
ESM1	endothelial cell specific molecule 1 [Source:HGNC Symbol;Acc:HGNC:3466]	290.0	31.8	**-6.63**	5.20E-17
MEST	mesoderm specific transcript [Source:HGNC Symbol;Acc:HGNC:7028]	57.1	5.0	**-6.82**	2.63E-15
NGF	nerve growth factor [Source:HGNC Symbol;Acc:HGNC:7808]	129.1	16.2	**-6.92**	7.82E-29
TSPAN2	tetraspanin 2 [Source:HGNC Symbol;Acc:HGNC:20659]	1192.6	154.4	**-7.11**	1.66E-66
MYBPH	myosin binding protein H [Source:HGNC Symbol;Acc:HGNC:7552]	57.6	5.7	**-7.21**	1.01E-20
MXRA5	matrix remodeling associated 5 [Source:HGNC Symbol;Acc:HGNC:7539]	1100.1	132.2	**-7.31**	4.87E-47
ARSI	arylsulfatase family member I [Source:HGNC Symbol;Acc:HGNC:32521]	102.6	11.7	**-7.46**	1.56E-32
SLC26A4	solute carrier family 26 member 4 [Source:HGNC Symbol;Acc:HGNC:8818]	1537.7	180.9	**-7.73**	2.50E-56
HAS2	hyaluronan synthase 2 [Source:HGNC Symbol;Acc:HGNC:4819]	1922.7	234.2	**-8.11**	3.32E-208
GREM1	gremlin 1, DAN family BMP antagonist [Source:HGNC Symbol;Acc:HGNC:2001]	6327.8	691.8	**-8.22**	1.55E-63
VCAM1	vascular cell adhesion molecule 1 [Source:HGNC Symbol;Acc:HGNC:12663]	24663.5	2854.2	**-8.51**	1.73E-99
P2RY6	pyrimidinergic receptor P2Y6 [Source:HGNC Symbol;Acc:HGNC:8543]	114.3	7.6	**-10.56**	7.18E-30
INHBA	inhibin subunit beta A [Source:HGNC Symbol;Acc:HGNC:6066]	26339.5	2220.8	**-11.71**	0
PLAU	plasminogen activator, urokinase [Source:HGNC Symbol;Acc:HGNC:9052]	169.7	9.5	**-14.03**	2.61E-51
